# Phytohormones and volatile organic compounds, like geosmin, in the ectomycorrhiza of *Tricholoma vaccinum* and Norway spruce (*Picea abies*)

**DOI:** 10.1007/s00572-020-01005-2

**Published:** 2020-11-18

**Authors:** Oluwatosin Abdulsalam, Katharina Wagner, Sophia Wirth, Maritta Kunert, Anja David, Mario Kallenbach, Wilhelm Boland, Erika Kothe, Katrin Krause

**Affiliations:** 1 Institute of Microbiology, Microbial Communication, Friedrich Schiller University Jena, Neugasse 25, 07743 Jena, Germany; 2 Max Planck Institute for Chemical Ecology, Hans-Knöll-Straße 8, 07745 Jena, Germany

**Keywords:** Ectomycorrhiza, *Tricholoma vaccinum*, Norway spruce, Volatile organic compounds, Geosmin, Germacradienol synthase, Phytohormones

## Abstract

The ectomycorrhizospheric habitat contains a diverse pool of organisms, including the host plant, mycorrhizal fungi, and other rhizospheric microorganisms. Different signaling molecules may influence the ectomycorrhizal symbiosis. Here, we investigated the potential of the basidiomycete *Tricholoma vaccinum* to produce communication molecules for the interaction with its coniferous host, Norway spruce (*Picea abies*). We focused on the production of volatile organic compounds and phytohormones in axenic *T. vaccinum* cultures, identified the potential biosynthesis genes, and investigated their expression by RNA-Seq analyses. *T. vaccinum* released volatiles not usually associated with fungi, like limonene and β-barbatene, and geosmin. Using stable isotope labeling, the biosynthesis of geosmin was elucidated. The geosmin biosynthesis gene *ges1* of *T. vaccinum* was identified, and up-regulation was scored during mycorrhiza, while a different regulation was seen with mycorrhizosphere bacteria. The fungus also released the volatile phytohormone ethylene and excreted salicylic and abscisic acid as well as jasmonates into the medium. The tree excreted the auxin, indole-3-acetic acid, and its biosynthesis intermediate, indole-3-acetamide, as well as salicylic acid with its root exudates. These compounds could be shown for the first time in exudates as well as in soil of a natural ectomycorrhizospheric habitat. The effects of phytohormones present in the mycorrhizosphere on hyphal branching of *T. vaccinum* were assessed. Salicylic and abscisic acid changed hyphal branching in a concentration-dependent manner. Since extensive branching is important for mycorrhiza establishment, a well-balanced level of mycorrhizospheric phytohormones is necessary. The regulation thus can be expected to contribute to an interkingdom language.

**Electronic supplementary material:**

The online version of this article (10.1007/s00572-020-01005-2) contains supplementary material, which is available to authorized users.

## Introduction

Communication is essential to establish interactions between different cells, individuals, species, kingdoms, and even domains of life in their respective habitats. Forests enhance their stress tolerance to biotic and abiotic factors via the formation of mutualistic mycorrhizal associations between mainly basidiomycete fungi and their host trees (Read et al. 2004). In the mycorrhizosphere, the mycorrhizal fungus interacts with the plant root and the released nutrients of this symbiosis influence the community of associated microorganisms in the soil (Timonen and Marschner 2006). Thus, the specific microbiome associated to a mycorrhizal root differs from that of the surrounding soil, which has led to the framing of this community as ectomycorrhizosphere. It can be accessed when soil adhering to an excavated mycorrhizal root is considered. The fungal partner within such an ectomycorrhizosphere is determined by the appearance of the mycorrhizal morphotype (Agerer 1987–2002).

The interaction in a specific ectomycorrhizal community is created by signals bi-directionally sent and perceived between the partners via the water phase as well as by air (Raudaskoski and Kothe 2015). Among those, phytohormones as well as volatile organic compounds (VOC) released to the environment can affect the symbiosis (Effmert et al. 2012). Phytohormones produced by ectomycorrhizal fungi can act in establishing mycorrhiza (Felten et al. 2009; 2012; Laurans et al. 2001; Luo et al. 2009; Splivallo et al. 2009). Evidence for mycorrhiza-modulating factors has been obtained for indole-3-acetic acid (IAA; Gea et al. 1994), salicylic acid (SA; Medina et al. 2003), jasmonates (JA; Hause et al. 2007), and strigolactones (Besserer et al. 2006), all known for their role as plant hormones and presence in fungal mycelia. Among those, SA is involved in plant-pathogen associations and can induce systemic acquired resistance (Audenaert et al. 2002). A volatile phytohormone, ethylene (ET), is a plant defense compound and stimulates lateral root development, mycorrhization, and IAA production in *Pinus contorta*,* Picea engelmannii*, and *Pseudotsuga menziesii* roots (Graham and Linderman 1980; Scagel and Linderman 1998; Splivallo et al. 2009). The production of IAA by *Tricholoma vaccinum* has been shown already (Krause et al. 2015).

Mono- and sesquiterpenes, aldehydes and alcohols, ketones, esters, organic acids, and aliphatic and aromatic hydrocarbons are represented among VOCs (Macías‐Rodríguez et al. 2015). A role for sesquiterpenes emitted by the ectomycorrhizal fungus *Laccaria bicolor* has been established: they stimulated lateral root growth of the host *Populus*, and also were able to stimulate root growth in the non-host plant *Arabidopsis* (Ditengou et al. 2015). Another volatile omnipresent in soils, geosmin, is a major contributor to the petrichor (earth smell) that is observed post-rain fall after a long spell of dryness. This volatile organic compound, trans-1,10-dimethyl-trans-9-decalol, is mainly produced by actinomycetes (Gerber and Lechevalier 1965). Reports of production by other organisms including mold fungi (Kikuchi et al. 1981; Mattheis and Roberts 1992), arthropods (Omura et al. 2002), and red beets (Lu et al. 2003) are available; fungi of the division Basidiomycota were not yet among those. The very low odor threshold (ca. 5–7 ng/L) of geosmin makes the compound to an important off-flavor contaminant of drinking water and bioaccumulates in fish and shellfish leading to a muddy taste (Dupuy et al. 1986).

The biosynthesis pathway of geosmin has been well characterized in bacteria, particularly in the actinobacterial genus *Streptomyces*. A bi-functional enzyme, germacradienol/geosmin synthase, has been identified to be specific for biosynthesis of geosmin. The N-terminal domain of the protein converts farnesyl diphosphate (FPP), the immediate precursor of cyclic sesquiterpenes, into germacradienol and germacrene D, while the C-terminal domain catalyzes the transformation of germacradienol to geosmin (Jiang et al. 2007). While the biosynthesis pathway has been settled for actinomycetes, eukaryotic pathways for geosmin biosynthesis are underrepresented. Siddique et al. (2012) reported a P450 monooxygenase (*gpe1*) involved in geosmin biosynthesis in *Penicillium expansum*. However, a germacradienol/geosmin synthase has not yet been reported from eukaryotes.

The effects of VOCs as signaling molecules in ectomycorrhizae have not largely been appreciated so far, and phytohormones have not yet been directly shown in a mycorrhizospheric habitat. We thus aimed at identifying plant hormones in the rhizosphere of Norway spruce (*Picea abies*) associated with the widespread ectomycorrhizal fungus *Tricholoma vaccinum* (Asiimwe et al. 2012). In addition, the potential of *T. vaccinum* to produce phytohormones and hormone-like substances, as well as other VOCs, was investigated and the biosynthetic pathway for the production of geosmin in the basidiomycete fungus has been identified. To show the relevance of putative volatile communication molecules in ectomycorrhiza formation and functioning, the effect of exogenous phytohormones was tested in fungal cultures.

## Material and methods

### Cultivation of *T. vaccinum*, mycorrhiza, preparation of soil extracts and root exudates

*T. vaccinum* GK6514 (FSU4731, Jena Microbial Resource Collection, Germany) grown in 50-mL liquid Modified Melin Nokrans b medium (1 L MMNb: 0.05 g CaCl_2_·2H_2_O, 0.025 g NaCl, 0.5 g KH_2_PO_4_, 0.25 g (NH_4_)_2_HPO_4_, 1 µg FeCl_3_·6H_2_O, 0.15 g MgSO_4_·7H_2_O, 10 g glucose, 5 g malt extract, 1 g peptone from casein, 10 mL Fortin solution (for 1 L: 3.728 g KCI, 1.546 g H_3_BO_3_, 0.845 g MgSO_4_·7H_2_O, 0.575 g ZnSO_4_·7H_2_O, 0.125 g CuSO_4_·5H_2_O), 83 µL thiamine hydrochloride (1.2 mg/mL stock solution) after Kottke et al. 1987) at room temperature in the dark without shaking for 4 weeks in three biological replicates was used for phytohormone and VOC measurements. Three technical replicates were performed for the measurements. Mycelial biomass (100 mg) of the grown culture from each of the three replicates was ground in liquid nitrogen and stored at − 80 °C. For spruce, *Picea abies* (Karst.) seeds (Landesforst Mecklenburg-Vorpommern, Germany) were soaked in tap water over night, sterilized in 30% H_2_O_2_, rinsed, germinated, and grown on germination medium as reported by Krause and Kothe (2006). Liquid MMNa medium (differs to MMNb with 0.5 g/L (NH_4_)_2_HPO_4_, 2 g/L glucose and without malt extract, modified after Kottke et al. 1987) was used in 50-mL Cellstar Cellreactor tubes with a 0.2-μm Ø pore-sized filter cap (Greiner Bio-One GmbH, Germany) for *P. abies* and *T. vaccinum* co-cultivation in a climate chamber with day-night cycle of 12 h at 23 °C (day) and 17 °C (night) and 80% humidity. Spruce root exudates were obtained after cultivation of 1-month-old seedlings in 100-mL 20% MMNa solution as described by Sammer et al. (2016).

Soil extracts were obtained from 100 mg mycorrhizospheric soil taken from around 1–10 cm depth in triplicates under *T. vaccinum* fruiting bodies growing in association with roots of spruce (located in a coniferous forest near Jena, Germany, 50.919674, 11.525103, see Wagner et al. 2019) showing the *T. vaccinum*–spruce morphotype of short roots (Agerer 1987–2002). Soil extract was prepared directly after sampling (1:1 *w*/*v* with water, vortexed 24 h at room temperature in the dark), sterile filtered, and frozen at − 20 °C.

For effects of phytohormones, *T. vaccinum* was cultivated in 4 replicates over 4 weeks on half concentrated MMNb plates supplemented with SA or ABA (4 nM, 4 µM, and 40 µM) in the dark. Stock solutions (10 mM) were dissolved in ethanol. Mycelial growth area and hyphal branching were checked (see Dor et al. 2011). Since experiments were performed in biological replicates, differences in branching of the control between experiments were observed. Thus, for different phytohormone concentrations, separate controls with ethanol were considered.

### Phytohormone measurement

The phytohormones abscisic acid (ABA), IAA, indole-3-acetyl-alanine (IA-Ala), indol-3-acetamide (IAM), indole-3-butyric acid (IBA), SA, JA, 12-oxo-phytodienoic acid (OPDA), and the derivates jasmonoyl-isoleucine (JA Ile), dicarboxy JA-Ile (COOH-JA-Ile), hydroxylated JA (OH-JA), and hydroxylated Ja Ile were measured (OH-JA-Ile; Schäfer et al. 2016) from ground mycelium, supernatant, soil extract, and root exudates. The samples were added in triplicate to 96-well BioTubes (VWR International GmbH, Germany) containing two steel balls and kept for 30 min at − 20 °C before incubation at − 20 °C overnight with extraction buffer (MeOH, milli-Q-H_2_O, HCOOH 16:4:1) spiked with standards [^2^H_3_]-DHZ 0.25 ng, [^2^H_6_]-IP 0.1 ng, [^2^H_5_]-IAA 3 ng, D_2_-dihydro-JA and D_5_-JA 100 ng, D_6_-ABA, D_4_-SA, and ^13^C_6_-JA-Ile 20 ng. After vortexing and centrifugation at 1.913 g for 20 min at 4 °C, the supernatant was kept at − 20 °C. A HR-X column was conditioned with MeOH followed by extraction buffer without standard. Flow-through was collected, the column washed with extraction buffer, and flow-through sampled again. MeOH was evaporated with nitrogen and 1 N HCOOH was added to the samples, vortexed, and centrifuged. Mass spectrometry analyses were performed (Bruker EvoQ-LC-QQQ-MS, Zorbax Eclipse XDB-C_18_ column: 50 × 3 mm, 1.8 µm, Agilent) in solvent A (0.05% formic acid; 0.1% acetonitrile in water) and solvent B (MeOH gradient: constant flow at 400 µL/min; time/%B, 0/5, 0.5/5, 0.6/50, 2.5/100, 3.5/100, 3.55/5, 4.5/5) with an injection volume of 1 µL. Multiple-reaction-monitoring mode was used (ESI positive and negative ionization mode, ion spray voltage 4500 eV, cone temperature 350 °C, cone gas flow 30 psi, heated probe temperature/flow 400 °C/40 psi, nebulizer gas flow 60 psi, collision gas 1.5 mTorr).

### VOC measurements

VOCs in the headspace of 4-week-old axenic *T. vaccinum* cultures were sampled over 24 h (for geosmin 48 h) via solid-phase microextraction (SPME; Supelco DVB/CAR/PDMS, Bellefonte, PA, USA; see Henke et al. 2015a), followed by GC-MS analyses (Finnigan Trace GC and Trace MS detector, Thermo Fisher Scientific, Dreieich, Germany) equipped with a ZB5 column (15 m × 0.25 mm × 0.25 µm) with 10 m Guardian End (Phenomenex, Aschaffenburg, Germany). Measurements were executed in electron impact (EI) mode with 70 eV at 1.5 mL/min helium. The GC injector (split ratio 1:7), transfer line, and ion source were set at 220 °C, 280 °C, and 200 °C, respectively. Spectra in the total-ion-current mode were taken with the programmed conditions from 40 °C followed by 10 °C/min to 200 °C. Spectra of the fungal volatiles geosmin, β-barbatene, and limonene were obtained using authentic standards. Standards of (±) geosmin and limonene were obtained from Sigma-Aldrich, Germany, and barbatene isomers were kindly provided by Stefan von Reuss (Neuchatel, Switzerland). For ethylene measurements, *T. vaccinum* was inoculated in 2 mL of half concentrated MMNb in 4-mL vials and incubated over 2 weeks without shaking before supplementation with 100 µL spruce root exudates or soil extract. MMNb medium with the supplements, but without the fungus, was used as control. For each treatment, three replicates were used. After 2 weeks of further incubation to achieve high VOC concentrations for the slow growing *T. vaccinum*, ethylene was measured with a photoacoustic laser spectrometer (Sensor Sense Nijwegen Netherlands; Wu et al. 2007).

### In silico analyses

The genome of *T. vaccinum* GK6514 is available on request via JGI IMG (http://img.jgi.doe.gov/) under the submission ID59348. The genome was scanned for genes involved in phytohormone or VOC biosynthesis using BLASTN search with software sequence server (http://www.sequenceserver.com) and NCBI database (http://blast.ncbi.nlm.nih.gov). Only experimentally verified protein sequences were used for the search. Further online tools of JGI IMG (http://img.jgi.doe.gov/) allowed to identify potential phytohormone and volatile biosynthesis proteins based on KEGG pathways and the identification of encoded proteins of the neighboring genes in the fully sequenced genome of *T. vaccinum*.

Using sesquiterpene synthase genes in *Coprinus cinerea okayama* (Agger et al. 2009), a gene with geosmin biosynthesis function was suggested. Sequences were analyzed and illustrated using BioEdit. The alignment was performed in MAFFT v7 (Katoh and Toh 2008) using BLOSUM80 and E-INS-I (Katoh and Standley 2013). Using Vector NTI software, multiple sequence alignments were performed using algorithms by Notredame et al. (2000).

### RNA-Seq analysis

Two different treatments were performed including *T. vaccinum* in pure MMNb medium and *T. vaccinum* spruce mycorrhiza in MMNa medium containing 2 g glucose/L, see also Material and methods/Cultivation of *T. vaccinum* and supplementary Fig. S1a. The slowly growing *T. vaccinum* needs long time to develop mature mycorrhiza. A liquid system was therefore used for the cultivation to allow for better long-term fitness of the plant and easier control of possible contamination. The samples were harvested for RNA-Seq after 8-month cultivation of *T. vaccinum* with and without spruce. Because only well-mycorrhized material should be used for the study, thin sections of the roots were produced and successful mycorrhiza formation was checked (Henke et al. 2015b, supplementary Fig. S1b, c). Predominantly fungal parts of mycorrhiza and fungal mycelia were harvested and ground with a pestle and mortar using liquid nitrogen. Total RNA was isolated (Erdmann et al. 2012) using RNeasy Plant Mini Kit (QIAGEN Hilden, Germany) with three biological replicates of cultures of each treatment. StarSEQ GmbH (Mainz, Germany) performed a check of RNA samples (Bioanalyzer, Qubit), isolated mRNA from total RNA, prepared a RNA library, performed sequencing of 2 × 150 nt with Illumina Next Seq 500, delivered data of FASTQ files (50 mio PE reads (2 × 150 nt, 7.5 Gb)/sample). Further RNA-Seq data were aligned to the genomes of *T. vaccinum* and *P. abies* 1.0 (https://jgi.doe.gov, http://congenie.org/blast., TopHat, FPKM/RPKM) and pairwise comparison with Cufflinks workflow was performed to show differential expression of genes (see at http://cole-trapnell-lab.github.io/cufflinks/cuffdiff/index.html).

### Deuterium-labeled substrate feeding experiments for volatile biosynthesis

Liquid cultures of *T. vaccinum* GK6514 in MMNb broth spiked with 3 mg deuterated mevalonolactone per 30 mL culture broth, a non-spiked control, and 3 mg 1-deoxy-D-xylulose 5-phosphate (DOX, a precursor of the non-mevalonate pathway) per 30 mL culture broth were used to confirm the secondary metabolite pathway in the synthesis of geosmin in *T. vaccinum.* After 4 weeks of growth, VOCs in the headspace of the axenic *T. vaccinum* cultures were sampled for 48 h via solid-phase microextraction, as described above. Conditioning of fibers followed the manufacturer’s instructions, followed by GC-MS analyses of the collected samples (Thermo Scientific MS ISQ LT and Trace 1310, Dreieich, Germany) using a ZB5 column (30 m × 0.25 mm × 0.25 µm) with 10 m Guardian End (Phenomenex, Aschaffenburg, Germany). Measurements were carried out in electron impact (EI) mode with 70 eV at 1.5 mL/min helium. The GC injector (split ratio 1:10), transfer line, and ion source were set at 230 °C, 280 °C, and 250 °C, respectively. Spectra in the total-ion-current mode were taken with the programmed conditions from 40 °C (2 min) followed by 10 °C/min to 230 °C and 50 °C/min to 300 °C.

### qRT-PCR and target gene expression analyses

Using 3-week-old *T. vaccinum* cultures, total RNA was isolated (Erdmann et al. 2012). To check the geosmin biosynthesis by the mevalonate pathway, 3 mg mevalonolactone dissolved in 100 µL sterile MMNb liquid broth was added to the culture and incubated for 6 h. For control, the same treatment without mevalonolactone was analyzed. To check the regulation of geosmin in the communication between the fungus and its plant host, 8-week-old co-cultures of *T. vaccinum* with *P. abies* were compared with axenic *T. vaccinum* grown under the same conditions. Regulation of *T. vaccinum* geosmin while interacting with selected bacteria isolated from the *T. vaccinum*–*P. abies* mycorrhizosphere (see Wagner et al. 2019) was also tested by 3-week-old *T. vaccinum* cultures with *Bacillus cereus*, *Bacillus zhangzhouensis*, and *Lysinibacillus* sp. in divided Petri dishes allowing VOC exchange for 72 h. After total RNA extraction, cDNA of all samples and replicates were synthesized using QuantiTect Reverse Transcription Kit (QIAGEN Hilden, Germany).

Gene expression changes for the candidate gene *ges1* (g5920) for geosmin production were observed using designed primers (forward: 5′CACTTCCCAAATACAGACCGTTCCC3′ and reverse 5′AAATCTTCGCTGGGTGCCCTCT3′) spanning an intron (primer synthesis Eurofins Genomics, Ebersberg, Germany). For reference, genes *act1*, *cis1*, and *tef1* coding for actin, citrate synthetase, and translation elongation factor (EF1α), respectively, were used with three technical replicates for each cDNA sample. The qPCR was carried out using Cepheid Thermocycler (Sunnyvale, USA) with SYBR Green fluorescence detection kit. Primer efficiencies were calculated using a dilution series of the cDNA and expression ratios normalized using the methods described by Pfaffl (2001).

## Results

### Contribution of *T. vaccinum* to phytohormones in mycorrhizosphere soil

**Fig. 1:**
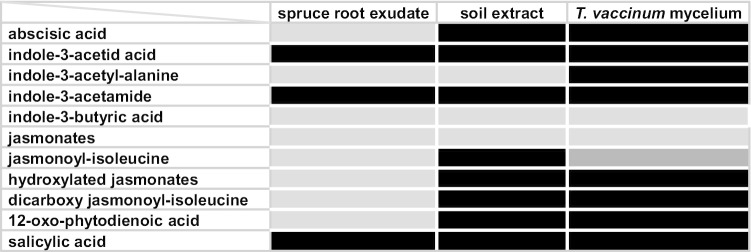
Qualitative analyses of phytohormones in spruce root exudates, soil extract from mycorrhizospheric sampling site, and *T. vaccinum* liquid cultures; light gray: no detection, black: production verified, dark gray: only found in supernatant not mycelium

*T. vaccinum* produced different phytohormones in liquid axenic cultures. Of those produced, IAA, IAM, SA, ABA, and JA-Ile were also found in soil extracts (Fig. 1). Spruce root exudates had a less diverse phytohormone pattern, including only IAA, IAM, and SA. However, the concentration of IAA and IAM was much higher in exudates (47.72 IAM area/standard; 136.54 IAA area/standard) than in soil (0.11 IAM area/standard; 0.16 IAA area/standard) or in *T. vaccinum* axenic cultures (0.31 IAM area/standard; 1.6 IAA area/standard). SA was found in all three environments, with 0.5 ng/mg around *T. vaccinum* mycelium, 0.9 ng/mg in the soil extract, and 1.6 ng/mg exuded by spruce roots (supplementary Fig. S2). In contrast, ABA was not excreted by the plant, but found in *T. vaccinum* cultures (0.1 ng/mg) and in mycorrhizosphere soil (0.01 ng/mg, supplementary Fig. S2). Like ABA, JA-Ile was measured in *T. vaccinum* cultures (0.004 ng/mg) and soil extract (0.009 ng/mg), albeit at very low concentrations, but was absent in root exudates.

### Effect of phytohormones on* T. vaccinum*

**Fig. 2:**
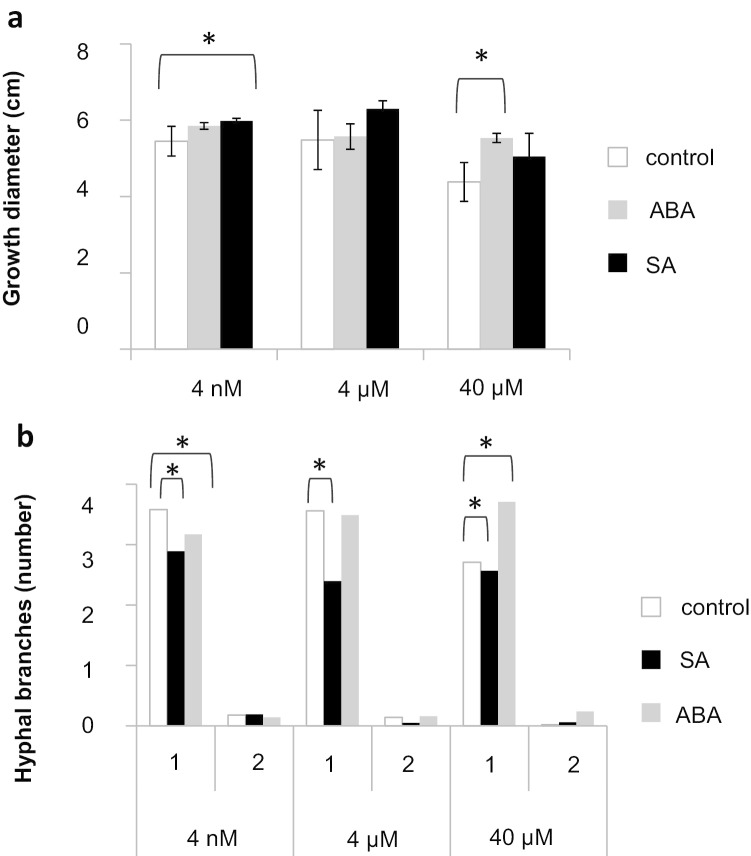
Effects of feeding of salicylic acid (SA) or abscisic acid (ABA) on growth (**a**) and branching (**b**) of *T. vaccinum.* Cultures were supplemented in a concentration gradient reflecting the naturally occurring phytohormone levels; 1: primary branch, 2: secondary branch, error bars indicate standard deviations, significant differences: *p < 0.05

Since phytohormones are major contributors to signaling compounds in ectomycorrhizal soil, their effect on both partners needs to be considered. While plant response to external phytohormones is well known, the effects on the fungal partner are less well described. Thus, prominent phytohormones found in the root exudates of *P. abies* (spruce) and soil extract were used to test for effects on radial growth and hyphal branching of *T. vaccinum*. Three concentrations of each phytohormone were tested. Cultures were supplemented in a concentration gradient reflecting the naturally occurring phytohormone levels found in this study. For the three different concentrations of SA, a corresponding increase in radial growth of *T. vaccinum* was observed (Fig. 2). Hyphal branching (both primary and secondary branching) was significantly reduced in all tested concentrations of SA. ABA inferred a considerable increase in the radial growth of *T. vaccinum* at 40 µM concentration and caused hyperbranching in the fungus (see Fig. 2). However, 40 µM is exceeding widely physiological concentrations.

### VOC production in *T. vaccinum*

**Fig. 3:**
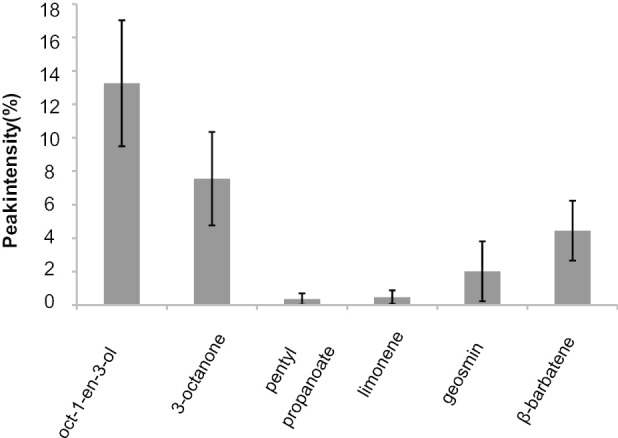
VOC identification and quantification from 4-week-old axenic liquid cultures of *T. vaccinum*; error bars indicate standard deviations

To test for potential signaling molecules, also volatile compounds were considered. *T. vaccinum* released different VOCs (Fig. 3; supplementary Table S1). The highest peak intensities belonged to the typical fungal VOCs oct-1-en-3-ol (13.3%) and 3-octanone (7.5%), known to correspond to the mushroom odor. Very low peak intensity indicated the presence of pentyl propanoate (0.4%) and limonene (0.5%) usually considered a plant VOC in cultures of the fungus without tree. Moreover, *T. vaccinum* produced the metabolite β-barbatene known from plants (4.4%) and the odor typical for streptomycetes, geosmin (2.0%, supplementary Fig. S3).

Ethylene concentrations in the headspace amounted to 5.7 nL/mg *T. vaccinum* biomass in axenic culture. The production was not significantly affected by supplementation with spruce exudates (4.1 ng/mg). However, significantly less ethylene was found in the presence of rhizospheric soil (0.4 ng/mg), likely due to microbial metabolism or signaling.

### In silico prediction of the potential biosynthesis genes

**Fig. 4:**
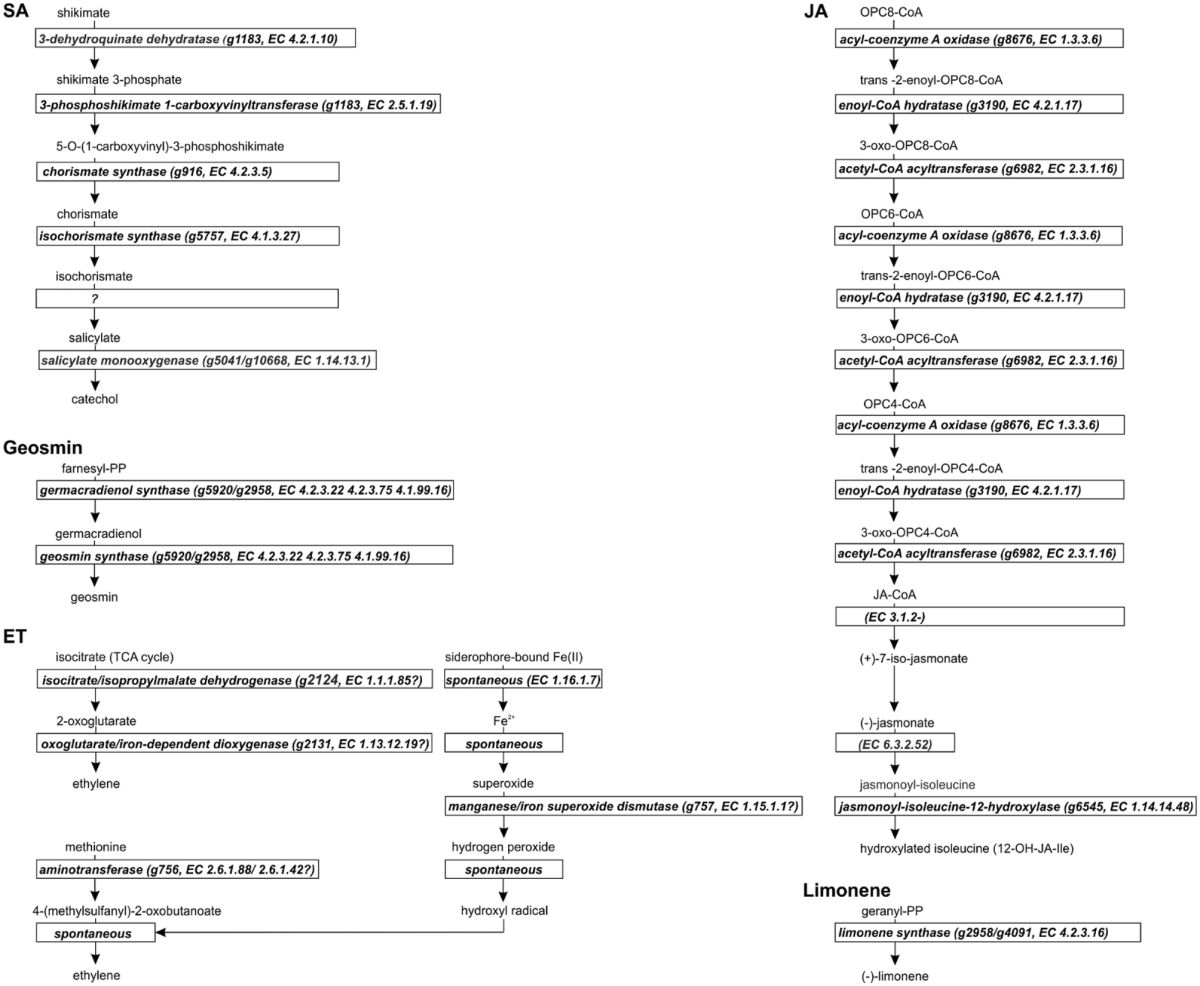
Potential phytohormone and volatile biosynthesis pathways, based on KEGG pathways of the fully sequenced genome of *T. vaccinum*, are presented with EC numbers and gene IDs. For salicylic acid (SA), a 3-dehydroquinate dehydratase (g1183), a 3-phosphoshikimate 1-carboxyvinyltransferase (g1183), a chorismate synthase (g916), an isochorismate synthase (g5757), and salicylate monooxygenases (e.g., g5041, g10668) were identified, but no isochorismate pyruvate lyase, which is thought to catalyze the last step to salicylic acid, was found. For jasmonic acid (JA), an acyl-coenzyme A oxidase (g8676), an enoyl-CoA hydratase (g3190), an acetyl-CoA acyltransferase (g6982), and Cytochrome P450 family 94 functioning as jasmonoyl-isoleucine-12-hydroxylase (g6545) were identified, but no enzymes catalyzing steps from JA-CoA to jasmonoyl-isoleucine, like jasmonoyl-L-amino acid synthetase, which is catalyzing the biological active jasmonoyl-isoleucine from jasmonate in *Arabidopsis thaliana*. For geosmin, g5920 and g2958 were identified to be germacradienol/geosmin synthases. For ethylene (ET) 4-(methylsulfanyl)-2-oxobutanoate (KMBA) pathway, methionine is catalyzed by an aminotransferase (g756) to 4-(methylsulfanyl)-2-oxobutanoate. A manganese/iron superoxide dismutase (g757) is catalyzing a precursor reaction resulting in a hydroxyl radical for final spontaneous reaction from 4-(methylsulfanyl)-2-oxobutanoate to ethylene. In ET EFE pathway, an isocitrate/isopropylmalate dehydrogenase (g2124) catalyzes the reaction from isocitrate of the TCA cycle to 2-oxoglutarate which is formed by an oxoglutarate/iron-dependent dioxygenase (g2131) to ethylene. For limonene, g2958 and g4091 forming limonene from geranyl diphosphate were identified

Since SA, JA, ET, geosmin, and limonene were produced by *T. vaccinum*, potential biosynthetic pathways were identified from the genome sequence. Here, only protein sequences of experimentally verified function from plants, fungi, and bacteria were considered which are associated with the corresponding function in KEGG pathways (Fig. 4). The comparison of SA and JA pathways in *T. vaccinum* with other organisms using KEGG maps and Blast analyses showed identity to plant pathways. The obtained SA pathway is linked to the isochorismate pathway in plants (Dempsey et al. 2011), but a gene encoding an isochorismate pyruvate lyase, which is discussed in plant biosynthesis pathways to catalyze the last step, was lacking from the fungal genome. A putative replacement was not identified.

Also, for JA biosynthesis pathway (Fig. 4), only one gene was missing. A jasmonate-amido synthetase catalyzing the formation of the biologically active jasmonoyl-isoleucine, known from *A. thaliana* with JAR1 (Guranowski et al. 2007), could not be identified from *T. vaccinum.* This corresponds to other fungi, where this gene is not present. However, some ectomycorrhizal basidiomycetes like *Boletus edulis* Bed1, *Suillus edulis* (protein ID 2761262324), and *Laccaria bicolor* (protein ID 2762831280) are available with this exact annotation lacking homologs in *T. vaccinum*.

ET biosynthesis enzymes of two microbial pathways were found in *T. vaccinum*; the 4-(methylsulfanyl)-2-oxobutanoate (KMBA) pathway starting with methionine is similar to that present in plants. The second pathway, the EFE pathway (Fig. 4), is very different from that present in plants (Groen and Whiteman 2014). No hits were identified for ABA biosynthesis genes using the available plant homologs.

We additionally checked for phytohormone synthesis islands in analyzing the neighboring genes of the identified phytohormone synthesis clusters (see supplementary Table S2, Figs. S4–S8), but could not verify linkage of the biosynthesis clusters. This corresponds also to the known pathway for synthesis of IAA (Krause et al. 2015; Henke et al. 2016). However, we found clusters for the two ET synthesis pathways. For the 4-(methylsulfanyl)-2-oxobutanoate (KMBA) pathway, next to an aminotransferase (g756), a manganese/iron superoxide dismutase (g757) is localized catalyzing a precursor reaction to obtain a hydroxyl radical for the final, spontaneous reaction from 4-(methylsulfanyl)-2-oxobutanoate to ethylene (supplementary Fig. S7). Also, for the isocitrate/isopropylmalate dehydrogenase (g2124), a linked gene for the enzyme catalyzing the reaction from isocitrate of the TCA cycle to 2-oxoglutarate was found (g2131). The latter is responsible for ethylene release by an oxoglutarate/iron-dependent dioxygenase. Next to that gene, an S-adenosyl-L-methionine-(SAM)-dependent methyltransferase (g2130) is localized, which is involved in plant ethylene production (Moffatt and Weretilnyk 2001). However, both pathways are not entirely assembled in a cluster that would suggest heterologous gene transfer for acquisition of ethylene biosynthesis.

As for the identified terpenoids, geosmin biosynthesis genes were detected with g2958 on contig 3429, a germacradienol/geosmin synthase, and a sesquiterpene synthase on contig 11698 (Fig. 4). This gene showed only low similarity to bacterial geosmin synthases, but was highly similar to Cop3 of *Coprinus cinereus* (Agger et al. 2009).

Possible limonene biosynthesis genes were identified with again g2958, the probable geosmin biosynthesis gene, and branching from that route g4091 coding for a limonene synthase responsible for the one-step cyclization of geranyl diphosphate into limonene (Fig. 4; see supplementary Table S2 and Fig. S8). Both are similar to fungal terpene synthases (see supplementary Table S3), g2958 to a protein of the ascomycete *Hypoxylon* sp., Hyp3, encoding a 1,8 cineole synthase producing 90% cineole and 10% limonene. The gene g4091 showed similarity to an aristolochene synthase from *Aspergillus terreus*, evolutionarily similar with cineole synthases (Shaw et al. 2015). Genes coding for enzymes associated with β-barbatene biosynthesis were not detected based on plant homologs.

### Unraveling the biosynthesis of the VOC geosmin

**Fig. 5:**
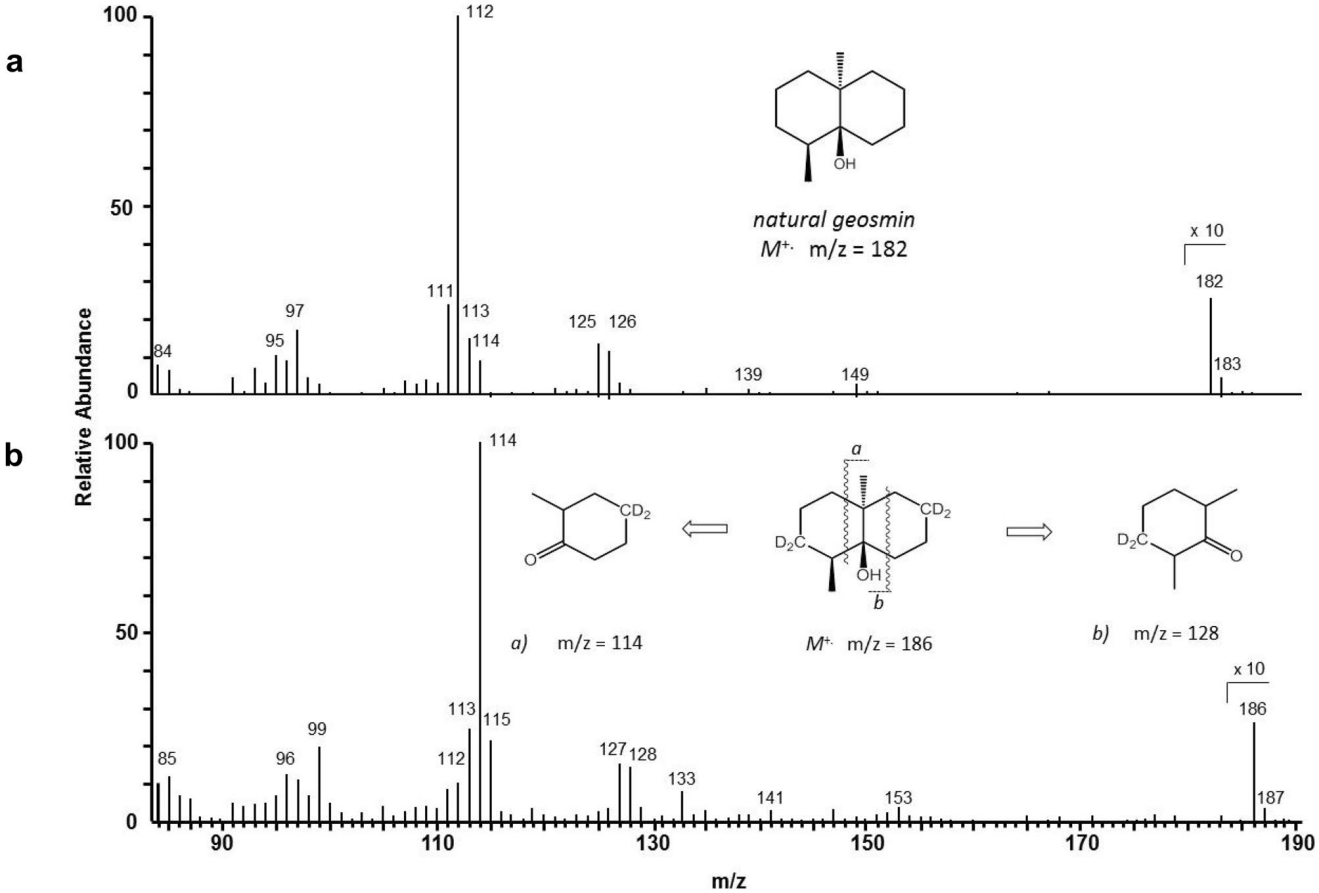
Mass spectra of natural geosmin (**a**, **b**) [^2^H_4_]-geosmin at m/z = 186 along with the two fragments at m/z = 114 and m/z = 128 indicating the presence of two deuterium atoms in each of the two fragments

The production of sesquiterpenes could be predicted to follow the mevalonate pathway. Therefore, *T. vaccinum* was grown in the presence of 3 mg ^2^H_2_-mevalonolactone for 4 weeks. Deuterium-labeled geosmin was seen with 15 to 29% label incorporation confirming the biosynthesis route (Fig. 5). To check for a contribution from the DOX pathway, *T. vaccinum* cultures spiked with 3 mg ^2^H_2_-DOX were analyzed. No incorporation of ^2^H_2_-DOX into geosmin was seen, which implies that geosmin biosynthesis in *T. vaccinum* exclusively proceeds via the classical mevalonate pathway and rules any contribution from bacteria (or endophytes) using the MEP-pathway. According to the high degree of deuterium labeling in [^2^H_4_]-geosmin (approx. 25%), the added [^2^H_2_]-mevalonolactone was efficiently converted into labeled farnesyl diphosphate [^2^H_6_]-FDP. The farnesyl diphosphate then was converted to the cyclic geosmin along a known sequence (Jiang and Cane 2008; Jiang et al. 2006, 2007; Spiteller et al. 2002), which involves a loss of a C_3_ segment with two deuterium atoms.

The mass spectrum of natural geosmin displays two even numbered fragments at m/z = 112 and 126 of high diagnostic value. Together with the molecular ion at m/z = 182, they allow the localization of the isotopes within the bicyclic skeleton of geosmin (Spiteller et al. 2002). As shown in Fig. 5, the fragment of m/z = 112 was shifted to m/z = 114, while at the same time, the second fragment at m/z = 126 was shifted to m/z = 128. The molecular ion raised from m/z = 182 to m/z = 186, indicating that in total, four deuterium atoms were present at the assigned positions in the geosmin molecule.

### Expression and regulation of the putative biosynthesis genes

By RNA-Seq analyses, pure fungal mycelium was compared with mycorrhizae to verify expression and regulation of genes involved in potential phytohormone or volatile biosynthesis pathways in mature mycorrhizae (see Table 1, compare supplementary B).

**Table 1:** Expression changes of *T. vaccinum* genes encoding proteins potentially involved in phytohormone and volatile biosynthesis based on RNA-Seq analyses with treatment pure fungal culture *versus* mycorrhiza

Gene ID	Function in biosynthesis pathway	log2 fold changes in RNA-Seq	Significance
g2124	ET: isocitrate/isopropylmalate dehydrogenase	0.0555816	No
g2131	ET: oxoglutarate/iron-dependent dioxygenase	0.355883	No
g756	ET: branched-chain amino acid aminotransferase	− 1.3683	Yes
g757	ET: Manganese/iron superoxide dismutase	0.449477	No
g1183	SA: 3-dehydroquinate dehydratase	− 0.378181	No
g916	SA: chorismate synthase	0.0602133	No
g5757	SA: isochorismate synthase	− 0.204483	No
g5041	SA: salicylate monooxygenase	1.87578	Yes
g10668	SA: salicylate monooxygenase	− 0.0884607	No
g8676	JA: acyl-coenzyme A oxidase	0.371859	No
g3190	JA: enoyl-CoA hydratase	− 0.664495	No
g6982	JA: acetyl-CoA acyltransferase	− 0.104792	No
g6545	JA: jasmonoyl-isoleucine-12-hydroxylase	− 0.712133	No
g5920	Geosmin: germacradienol/ geosmin synthases	− 0.0812449	No
g2958	Geosmin, Limonene: germacradienol/ geosmin/ limonene synthases	1.23004	No
g4091	Limonene: Geranyl diphosphate lyase (Limonene forming)	2.42992	No
g2731	IAA: tryptophan aminotransferase Tam1	1.11115	Yes
g4322	IAA: indole-3-pyruvic acid (IPA) decarboxylase Ipd1	− 0.810077	No
g7538	IAA: aldehyde dehydrogenase (NAD(P)+) Ald1	− 1.41141	No
g5206	IAA: aldehyde dehydrogenase (NAD(P)+) Ald2	− 1.91556	Yes
g6636	IAA: aldehyde dehydrogenase (NAD(P)+) Ald3	− 1.59273	Yes
g2541	IAA: aldehyde dehydrogenase (NAD(P)+) Ald4	− 1.59273	Yes
g6551	IAA: aldehyde dehydrogenase (NAD(P)+) Ald5	0.389237	No
g34	IAA: aldehyde dehydrogenase (NAD(P)+) Ald6	− 0.130813	No
g5201	IAA: aldehyde dehydrogenase (NAD(P)+) Ald7	− 0.359637	No
g2766	IAA: Prephenate dehydrogenase (NADP+) TyrA	− 0.67403	No
g7957	IAA: auxin efflux carrier transmembrane protein	3.27473	Yes

The expression values of three biological replicates of both treatments were used for log2 fold change calculation. Significant data show a *p* value ≥ 0.005

As for the phytohormones, tryptophan aminotransferase *tam1* for IAA biosynthesis as well as a gene encoding auxin efflux carrier transmembrane protein showed up-regulation in mycorrhiza. The aldehyde dehydrogenases *ald2-ald4* were downregulated, indicating that those genes are not specifically involved in IAA synthesis. In the SA pathway, up-regulation of the SA degrading enzyme salicylate monooxygenase was seen in mycorrhiza, indicative for SA being rather in excess during symbiosis. For JA, no significantly changed expression was observed. The ET (KMBA) pathway seems to be down-regulated during mycorrhization, as the aminotransferase showed significant repression.

**Fig. 6:**
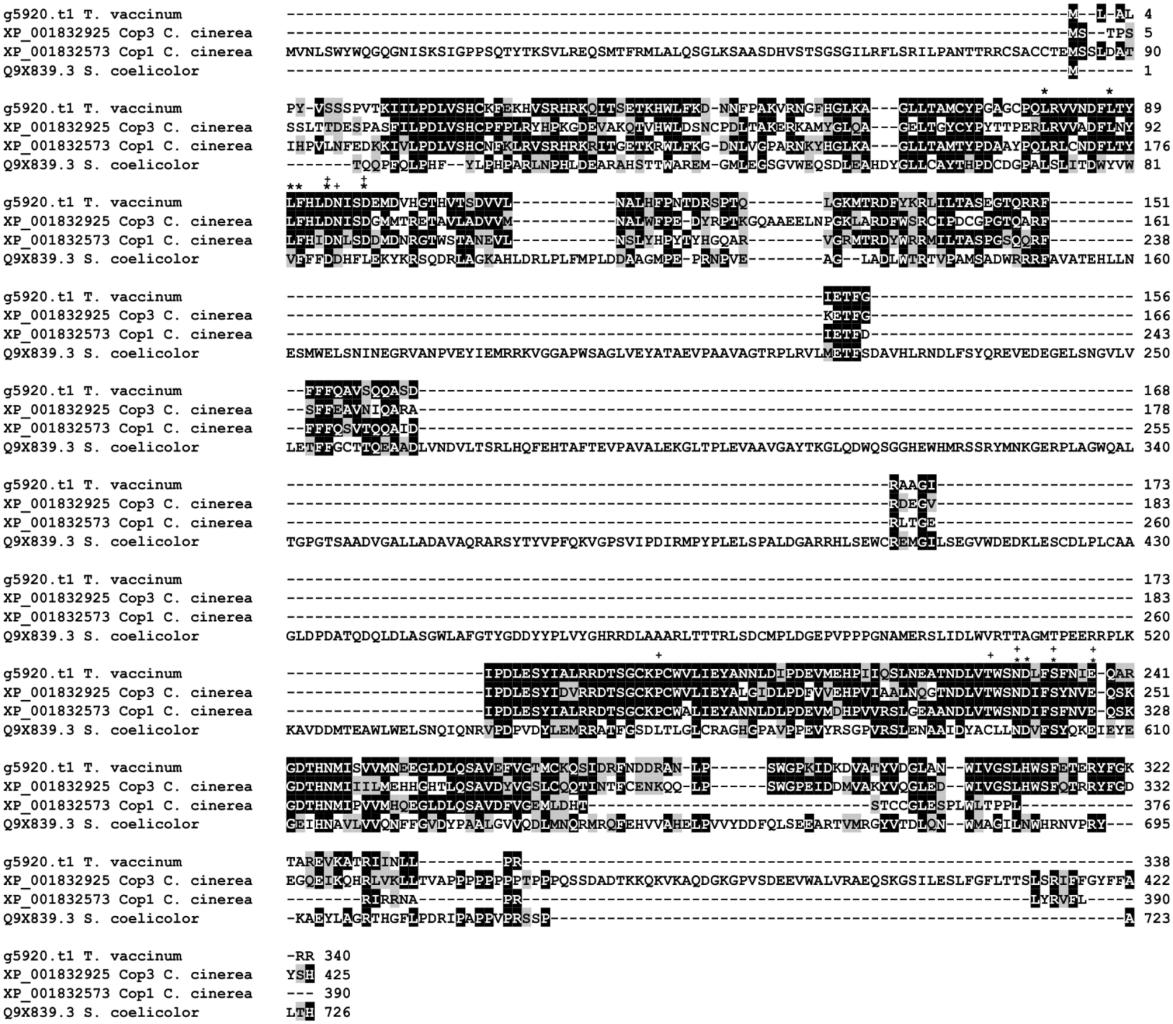
Multiple sequence alignment of the conceptually translated Ges1, putatively involved in geosmin biosynthesis, with fungal sesquiterpene synthases Cop1 and Cop3 from *C. cinerea okayama* and Cyc2, a bacterial germacradienol/geosmin synthase from *Streptomyces coelicolor*; *, substrate binding pocket; +, substrate-Mg^2+^ binding site

**Fig. 7:**
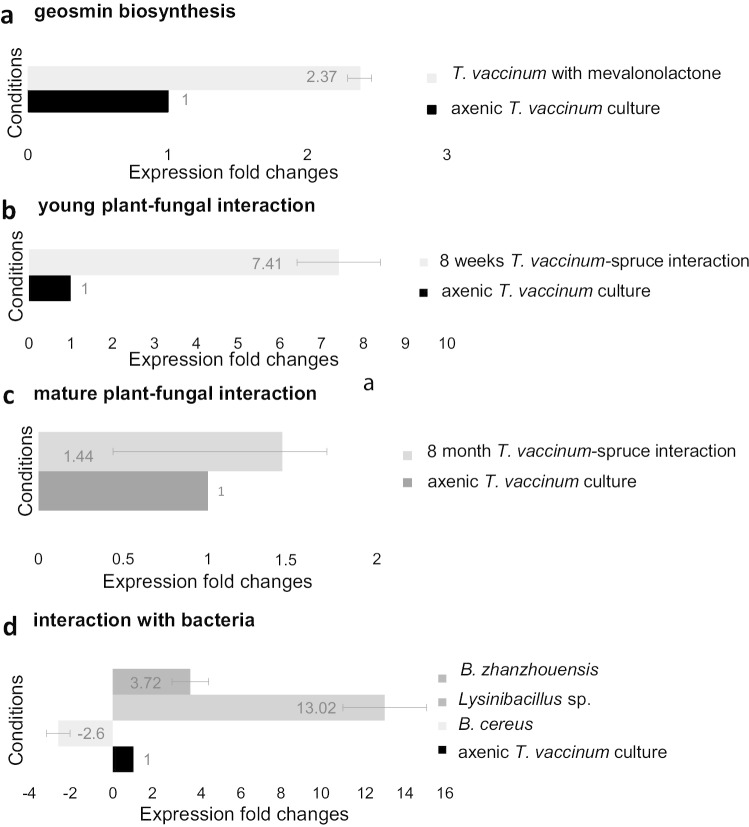
Expression analyses of *ges1* by qRT-PCR. **a** Mevalonolactone was added as a precursor in the classical mevalonate pathway at 3 mg ^2^H_2_-mevalonolactone in axenic culture medium to examine geosmin biosynthesis, **b** young mycorrhizal interaction with spruce, **c** mature mycorrhizal interaction with spruce and validation of RNA seq data, and **d** interaction of mycelium of *T. vaccinum* with selected bacteria from the mycorrhizosphere were tested

For the biosynthesis of the VOCs geosmin and limonene, no significant regulatory pattern was found. This prompted us to further investigate the geosmin biosynthesis genes for expression signals by qRT-PCR. The identified gene *ges1* (g5920) shows typical sequence motifs of sesquiterpene synthases, high sequence identity with fungal sesquiterpene synthases of *C. cinerea okayama*, and lower similarity with bacterial geosmin synthases (Fig. 6). Thus, the biosynthesis pathway was tested using potential precursors. The mevalonate pathway for secondary metabolism was tested feeding mevalonolactone to the fungal culture. A more than two-fold (2.37, Fig. 7) increase in *ges1* expression suggested involvement of this gene in the biosynthesis of geosmin. Expression of *ges1* in 8-week-old interaction with the plant host showed a 7.41-fold increase compared with axenic culture of the fungus (see Fig. 7), which further strengthened the notion of a role of geosmin in the communication between plant and fungus while mycorrhiza is still young. In validation of the RNAseq data, qRT-PCR was performed. As often observed, the amount of fold change was different as compared with RNA-Seq, but no regulation was seen (1.44-fold increase compared with axenic culture of the fungus in an 8-month-old plant-fungus interaction). A potential impact of ectomycorrhizosphere bacteria was tested by looking at *ges1* expression in interactions of fungal mycelium with volatiles of selected mycorrhizosphere bacteria. Indeed, the interactions with *B. zhangzhouensis* or *Lysinibacillus* sp. isolated from the ectomycorrhizosphere soil showed a 3.72- and 13.02-fold up-regulation, while interaction with *B. cereus* led to a 2.60-fold down-regulation (see Fig. 7). Thus, fine-tuning of VOC production in association with the co-occurring microbiome of a mycorrhiza could be verified.

## Discussion

Here, we analyzed the potential of the ectomycorrhizal fungus *T. vaccinum* to produce phytohormones and VOCs. We detected and quantified phytohormones excreted by the roots of the host tree *P. abies*, by axenic fungal cultures, and those present in the mycorrhizospheric soil. The VOC profile of axenically grown *T. vaccinum* mainly comprised the typical fungal VOCs, oct-1-en-3-ol and 3-octenone (Bäck et al. 2010; Fäldt et al. 1999; Müller et al. 2013). These compounds are known to attract insects and induce plant defense, which may be inferred from Kishimoto et al. (2007), where *A. thaliana* defense genes were induced by low concentrations of oct-1-en-3-ol. An induced plant resistance is also supported by the finding that the rate of plant pathogen attack on spruce was reduced in mycorrhizal spruce-*T. vaccinum* co-cultures (Wagner et al. 2019).

**Fig. 8:**
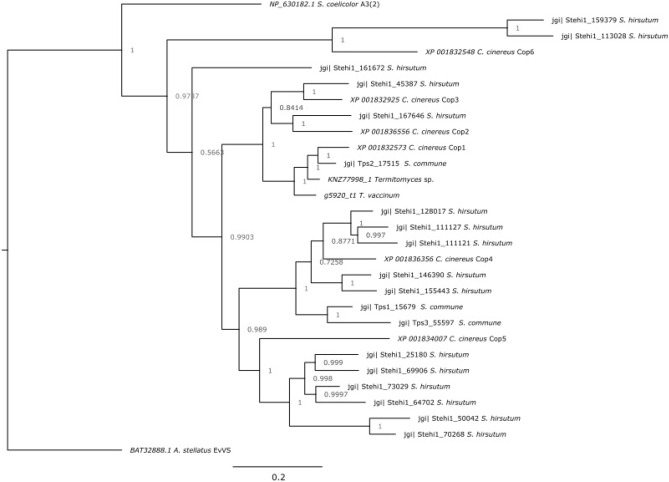
Phylogenetic tree showing clusters of different sesquiterpene synthases in basidiomycetes including g5290 from *T. vaccinum* clearly separated from the germacradienol/geosmin synthase of *Streptomyces coelicolor*. GenBank accession numbers and JGI IDs are given with the organism names

The identification of geosmin as a VOC produced by *T. vaccinum* shows that also soil basidiomycetes may contribute to the earthy smell. Geosmin, in addition to enhancing sporulation (Bentley and Meganathan 1981) and spore germination in arbuscular endomycorrhizal fungi (Carpenter-Boggs et al. 1995), thus might play a role also in ectomycorrhiza formation. Even if no direct effect on *T. vaccinum* was seen, promoting sporulation or germination of plant growth promoting bacteria and fungi in the spruce mycorrhizosphere might benefit the ectomycorrhiza formation. A route for the biosynthesis of the compound therefore was of interest. As no basidiomycetes had been reported to produce geosmin, the geosmin biosynthetic pathway was elucidated. *T. vaccinum* uses the classical mevalonate pathway. The alternative pathway known from many bacteria, plants, and apicomplexan protozoa, the 2-*C*-methyl-D-erythritol 4-phosphate/1-deoxy-D-xylulose 5-phosphate (MEP/DOXP) pathway (Spiteller et al. 2002) present in, e.g., *Streptomyces* sp., was not used by the basidiomycete. The only other reported potential biosynthesis gene in fungal geosmin production (*gpe1*) had been found with the ascomycete mold *Penicillium expansum* (Siddique et al. 2012). The geosmin biosynthetic gene in *T. vaccinum* was identified to be different from *gpe1*. A *Termitomyces* sp. germacradienol/germacrene D synthase-like gene was identified in the *T. vaccinum* genome instead. It is noteworthy, however, that the gene is dissimilar to the germacradienol/geosmin synthase reported from actinomycetes (Jiang et al. 2007), but clusters with *Coprinus cinereus* and *Schizophyllum commune* sesquiterpene synthases (Cop1 and Tps2, respectively; Fig. 8). Using transcript analyses, an increase in expression in the presence of the precursors, as well as in young interaction with the host plant, was shown. Specific regulation of *T. vaccinum ges1* was seen during interaction with co-occurring bacteria showing mycorrhiza-helper abilities (Wagner et al. 2019). Since up- versus down-regulation was species-dependent, no correlation with mycorrhiza-helper abilities was visible. However, the down-regulation of *ges1* observed in *B. cereus* might instead be linked to its increase in pigment production (Wagner et al. 2019).

Other VOCs produced by *T. vaccinum* included the monoterpene limonene and the sesquiterpene β-barbatene, both not usually considered typical for fungal volatiles. However, both of these classical plant metabolites were found also in fruiting bodies of the brown rot fungus *Fomitopsis pinicola* (Fäldt et al. 1999), while limonene typically is found in *Picea abies* needles (Persson et al. 1996). While VOC profiles of *T. vaccinum* and other ectomycorrhizal fungi, like *Paxillus involutus* or *Laccaria bicolor*, differ strikingly, a similarity to *F. pinicola* VOC pattern might suggest host range specificity as an evolutionary factor for VOC patterns. This seems to be the case irrespective of symbiotic versus pathogenic life styles (Müller et al. 2013), since *F. pinicola* has been shown to be most commonly found as conifer pathogen (and in very rare cases on birches). Two possible genes involved in limonene synthesis were identified in *T. vaccinum.* The terpenoid synthase g2958 showed similarity to proteins in *F. pinicola* and *P. involutus*, while g4091 shows similarity with two genes in *F. pinicola* and much less to *P. involutus* (supplementary Table S3). Probably due to limited available information on monoterpene synthases in fungi (Quin et al. 2014), KEGG analyses of genes in the monoterpenoid biosynthesis pathway did not yield a hit, even though all basidiomycete genomes available were screened (including, *F. pinicola*, *P. involutus*, and *L. bicolor*). Therefore, manual BLAST analysis of potential limonene synthase proteins was used to identify the genes as described above. Limonene displays antimicrobial activities especially against fungi (Duetz et al. 2003). In spruce roots, the composition of the monoterpenes changed during *Heterobasidion annosum* attack and increased in limonene concentration (Rieger 1995). The same pattern is seen during mycorrhization with *T. vaccinum*, where limonene is the major VOC compound of the spruce profile (Henke et al. 2015a). *T. vaccinum* is also sensitive to limonene, but only at high, non-natural concentrations (Schlunk et al. 2015). Since β-barbatene also shows antimicrobial activity, we assume that both are involved in plant defense of *P. abies* (Bukvicki et al. 2013). The fungal production thus could be connected to the protection against pathogens seen with mycorrhiza (Wagner et al. 2019).

Other typical plant metabolites produced by *T. vaccinum* are phytohormones. IAA and its intermediate IAM were detected in *T. vaccinum* rhizospheric soil and axenic cultures in addition to the expected presence in spruce root exudates. IAA and its precursors had already been shown to increase branching in *T. vaccinum* which is a pre-requisite to produce the hyphal mantle around the plant root (Krause et al. 2015). Fungal cultures compared with mycorrhiza revealed no clear up-regulation of the genes involved in IAA biosynthesis. Some aldehyde dehydrogenases are down-regulated, which might either be indicative of no involvement of these specific dehydrogenase genes in IAA synthesis, or it may be caused by the mature mycorrhiza stage used in this study. It has been proposed that at later stages, lower IAA levels than during initiation with massive re-structuring are necessary. Thus, IAA is involved in the formation of both mantle and Hartig’ net formation in early mycorrhiza (Henke et al. 2016). Nearly 75% of isolated fungi and bacteria from mycorrhizospheric soil samples were able to produce IAA (Wagner et al. 2019) and thus to stimulate plant growth (Davies 2010; Gea et al. 1994) providing more accessible short roots for mycorrhiza formation (Wagner et al., 2019).

An involvement of *T. vaccinum* in inducing plant defense is indicated by the production of JA derivates. The conjugate JA-Ile detected in *T. vaccinum* cultures and in the soil samples is known to be produced by plants after wounding (Gális et al. 2009) and thus may reinforce pest resilience in mycorrhized plants. Since exogenous application of methyl jasmonate to the stem of *P. abies* activated plant defense and reduced disease symptoms caused by *Ceratocystis polonica* (Krokene et al. 2008), it also may induce systemic pathogen resistance. Many fungi like *Coprinus* species, *Mycena galericulata*, or *Trametes versicolor* have been shown to produce JA (Miersch et al. 1993). The predicted biosynthesis genes in *T. vaccinum* show sequence similarity to a protein of *Aspergillus niger* with strong similarities to the oxophytodienoic acid reductase from plants, which is a key enzyme in JA biosynthesis (Taki et al. 2005).

SA is an effective substance in Norway spruce defense, e.g., against the pathogen *Heterobasidion parviporum* that causes stem and butt rot (Arnerup et al. 2013). For mycorrhiza, it has long been proposed that plant defense needs to be reduced to allow for colonization. Accordingly, down-regulation of gene expression in SA and ET biosynthesis was shown here. As an effect on the fungus, branching of *T. vaccinum* was reduced after SA application. Instead, the mycelium explored a larger surface area aiding in nutrient acquisition. Root-derived SA could thus re-direct *T. vaccinum* growth towards soil exploration. At earlier stages, SA close to the root might increase directional growth towards the root. Since the fungus could form SA, a role in re-directing growth might also be possible in saprotrophic growth. The biosynthetic polyketide synthase shows similarities to a 6-methyl salicylic acid synthase of *Penicillium griseofulvum* (Spencer and Jordan 1992).

ABA was not found in the spruce exudate, but its presence in root apices is well documented (Dunstan and Bock 1997). It positively influences phosphate utilization and nitrate uptake (Ullrich and Kunz 1984) as well as water balance in plants (Bradford 1983; Xu et al. 2013). We found ABA in *T. vaccinum* axenic cultures and in the soil samples*.* SA has been shown to enhance hyphal branching in AM mycorrhiza (Herrera-Medina et al. 2007). Changes in SA/ABA levels in the rhizosphere could modulate growth rate and branching in *T. vaccinum* and thus support adaptation to fluctuating environmental conditions and facilitate mycorrhization. The production in axenic fungal cultures would make the substances, known only for their phytohormone functions, to be systemic signals in fungi during growth in soil, which has not yet been reported.

In conclusion, the ectomycorrhizosphere *of T. vaccinum* and *P. abies* was shown to be a place of concomitant production of phytohormones as well as volatiles which then are available for exchange of dissolved or volatile signals. Both, VOCs and phytohormones could be shown to be produced by both partners, and for both mycorrhizal partners, a reaction to the compounds was verified. This establishes cross-kingdom signal exchange that might be necessary for optimal development of both symbionts in a fine-tuned manner. The biosynthesis by the basidiomycete fungus evidences co-evolution in this mutual symbiosis. In addition, the ectomycorrhizal microbiome modulates production. Appearance of the same molecules like SA, IAA, geosmin, or limonene in evolutionarily unrelated organisms stimulates the discussion to an “interkingdom language” in the ectomycorrhizosphere.

## Electronic supplementary material

Below is the link to the electronic supplementary material.

Supplementary file1 (DOCX 1691 KB)

Supplementary file2 (PDF 9514 KB)
