# Supplementary material for: Phytohormones and volatile organic compounds, like geosmin, in the ectomycorrhiza of Tricholoma vaccinum and Norway spruce (Picea abies)
Source: Mycorrhiza. 2020 Nov 18;31(2):173–88. doi: 10.1007/s00572-020-01005-2 (PMC7910269; doi:10.1007/s00572-020-01005-2)
Supplement: Supplementary file 1 — Supplementary file1 (DOCX 1691 KB) [file 572_2020_1005_MOESM1_ESM.docx]

**Supplementary Material**

**
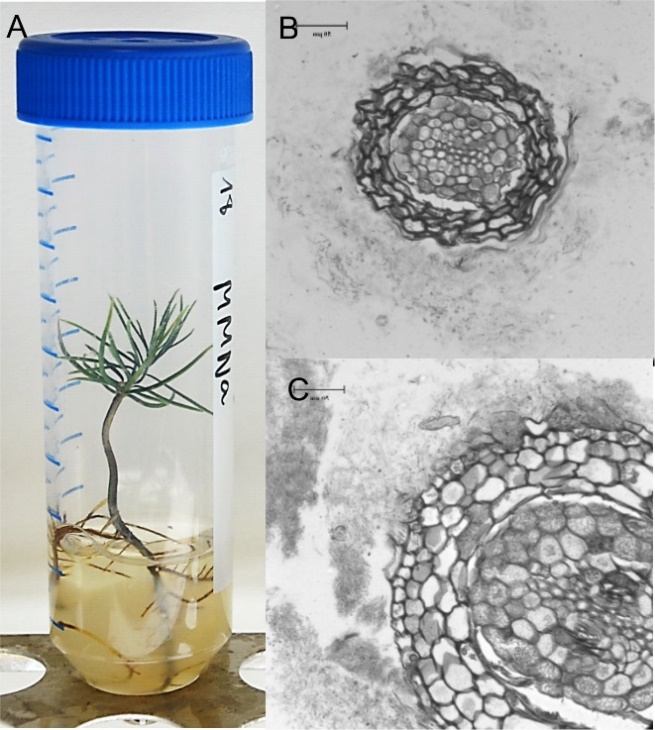
**

**Supplementary Fig. S1** 8 months old *T. vaccinum* spruce co-cultures grown in MMNa and used for RNA-Seq analyses. A) Liquid *T. vaccinum* spruce co-culture; B, C) toluidine-blue stained fungi in cross sections of selected spruce roots to show good mycorrhization.

**
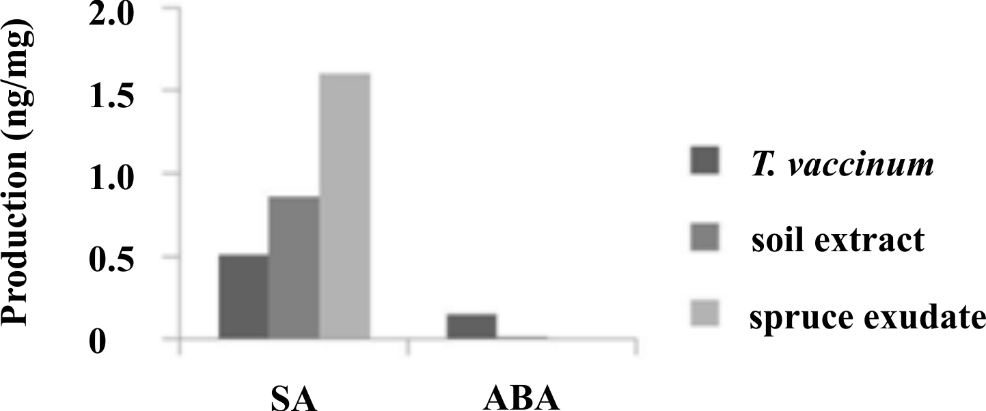
**

**Supplementary Fig. S2** Quantitative analyses of salicylic acid (SA) and abscisic acid (ABA) in spruce root exudates, soil extract from mycorrhizospheric sampling site and axenic *T. vaccinum* liquid cultures.

**Supplementary Table S1** VOCs identified after GC-MS after 24 h SPME sampling in the headspace of liquid *T. vaccinum* cultures (P1-P3) compared to the non-inoculated media (K1-K3) and analyzed with Massfinder against n-alkanes C8-C20.

| Treatment | Measured VOCs |
| --- | --- |
| K1 | Heptanone<5-methyl-3-> |
| K2 | Heptanone<5-methyl-3-> |
| K3 | Heptanone<5-methyl-3-> |
|  | Furfural<5-methyl-> |
|  | Benzene aldehyde |
|  | Hexenyl propionate<3Z> |
|  | Butanoate<3-methyl-2-butenyl 2-merhyl-> |
|  | Octenol acetate<2E> |
| P1 | Heptanone<5-methyl-3-> |
|  | Dimethyl-4-heptanone<3,5-> |
|  | Octen-3-ol<1-> |
|  | Octanone<3->Penthyl propanoate |
|  | Limonene |
|  | Geosmin |
|  | ß-barbatene |
| P2 | Heptanone<5-methyl-3-> |
|  | Octen-3-ol<1-> |
|  | Octanone<3->Penthyl furan<2-> |
|  | Geosmin |
|  | ß-Barbatene |
| P3 | Heptanone<5-methyl-3-> |
|  | 4-Methyl-3-heptanol |
|  | Octen-3-ol<1-> |
|  | Octanone<3->Pentyl propanoate |
|  | Limonene |
|  | Ethyl benzoate |
|  | Geosmin |
|  | ß-Barbatene |


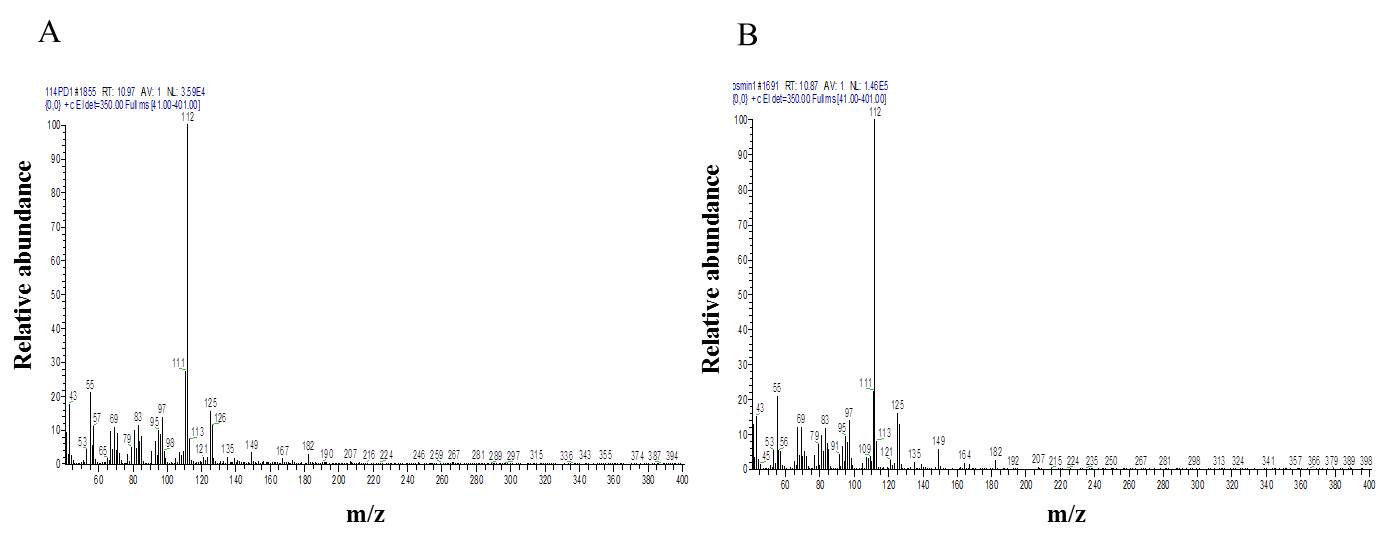

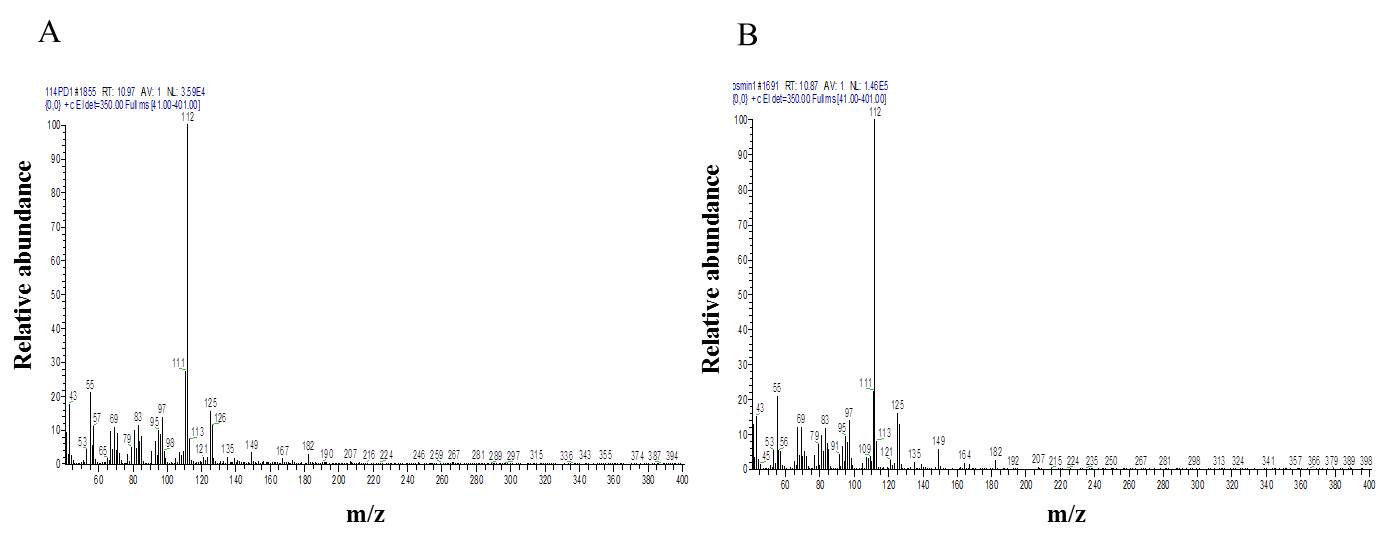


**A**

**B**

**Supplementary Fig. S3** Geosmin fragmentation profile after GC/MS measurements. Relative abundancies of fragments are shown correlated to their m/z ratio. A) SPME sampling in the headspace of *T. vaccinum* cultures; B) geosmin standard.

**Supplementary Table S2** Potential biosynthesis genes and its localization in the genome of *T. vaccinum*.

| **Gene ID** | **Function**  BlastP [Score, E value, Identity, Length] | | **Contig number** | **Proteins encoded by neighboring genes** |
| --- | --- | --- | --- | --- |
| **IAA biosynthesis pathway** | | | | |
| g2731 | IAA: tryptophan aminotransferase Tam1 | 2876 | g2730 Serine/threonine protein kinase, g2732 Galactokinase, g2733 Clathrin adaptor, g2734 Clavaminate synthase | |
| g4322 | IAA: indole-3-pyruvic acid (IPA) decarboxylase Ipd1 | 6326 | g4321 Cytochrome P450, g4323 Casein kinase, g4324 Nucleoside-diphosphate-sugar epimerase | |
| g7538 | IAA: aldehyde dehydrogenase (NAD(P)+) Ald1 | 21538 | g7539 hypothetical | |
| g5206 | IAA: aldehyde dehydrogenase (NAD(P)+) Ald2 | 8961 | g5207 Glyceraldehyde-3-phosphate dehydrogenase-like | |
| g6636 | IAA: aldehyde dehydrogenase (NAD(P)+) Ald3 | 15511 | g6635 hypothetical | |
| g2541 | IAA: aldehyde dehydrogenase (NAD(P)+) Ald4 | 2605 | g2542 4-hydroxybenzoate polyprenyltransferase, g2543 and g2544 hypothetical | |
| g6551 | IAA: aldehyde dehydrogenase (NAD(P)+) Ald5 | 15083 | g6548, g6549, g6550 and g6552 hypothetical, g6553 G protein alpha subunit | |
| g34 | IAA: aldehyde dehydrogenase (NAD(P)+) Ald6 | 38 | g31 Serine/threonine protein kinase, g32 hypothetical, g33 WD40-repeat, g35 hypothetical, g36 histone-lysine N-methyltransferase SETD1 | |
| g5201 | IAA: aldehyde dehydrogenase (NAD(P)+) Ald7 | 8957 | g5202 tRNA (C5-cytosine) methyltransferase, g5203 Mismatch repair ATPase, g5204 Histone deacetylase, g5205 Na+-driven multidrug efflux pump | |
| g2766 | IAA: Prephenate dehydrogenase (NADP+) TyrA | 2940 | g2760 FAD/NAD(P)-binding, g2761 and g2762 hypothetical, g2763 P-loop containing nucleoside triphosphate hydrolase, g2764 diacylglycerol acyltransferase, g2765 AAA family ATPase, g2767 Protein kinase, g2768 Aldehyde/histidinol dehydrogenase, g2769 and g2770 Glycoside hydrolase, | |
| g7957 | IAA: auxin efflux carrier transmembrane protein | 24939 | - | |
| **SA biosynthesis pathway** | | | | |
| g1183 | SA: 3-dehydroquinate dehydratase | 124744 | g1180 hypothetical, g1181 mitochondrial chaperone BCS1, g1182 ribose-5-phosphate isomerase, g1184 Regulator of G protein signalling superfamily, g1185, g1186 and g1187 hypothetical, g1188 SET, g1189 EF-hand, g1190 hypothetical, g1191 DNA-directed DNA polymerase | |
| g916 | SA: chorismate synthase | 855 | g913, g914 and g915 hypothetical, g916 Archaeal 2-phospho-L-lactate transferase, g917 hypothetical | |
| g5757 | SA: isochorismate synthase | 11124 | g5752 SPC24 kinetochore, g5753 CAF1A, g5754 Replication factor C, g5755 Heat shock protein 70, g5756 Ribosomal_S2 | |
| g5041 | SA: salicylate monooxygenases | 8474 | g5039 Protein kinase, g5041 Clavaminate synthase-like | |
| g10668 | SA: salicylate monooxygenases | 64374 | g10666 Protein disulfide-isomerase, g10667 HSP20 | |
| **JA biosynthesis pathway** | | | | |
| g8676 | JA: acyl-coenzyme A oxidase | 32070 | g8675 and g8677 hypothetical, g8678 Cytochrome P450 CYP2, g8679 Nuclear transport receptor LGL2, g8680 alpha/beta-Hydrolases, g8681 NADH dehydrogenase (ubiquinone) flavoprotein 1 | |
| g3190 | JA: enoyl-CoA hydratase | 3735 | g3189 hypothetical, g3191 Cytochrome P450, g3192 hypothetical, g3193 RhoGAP, g3194 Sterile alpha motif, g3195 hypothetical, g3196 Predicted phosphatase Zink finger, g3197 hypothetical | |
| g6982 | JA: acetyl-CoA acyltransferase | 17529 | g6981 RNA processing exonuclease, beta-lactamase fold | |
| g6545 | JA: jasmonoyl-isoleucine-12-hydroxylase | 15077 | g6543 and g6544 hypothetical | |
| **ET biosynthesis pathway** | | | | |
| g2131 | Oxoglutarate/iron-dependent dioxygenase ethylene forming enzyme *efe* of *Pseudomonas savastanoi pv. phaseolicola* P32021 [83.6, 9e-18, 81/310 26%, 1062] | 2100 | g2122 P-loop containing nucleoside triphosphate hydrolase, g2123 RNA-binding protein Nob1, g2124 Isocitrate/isopropylmalate dehydrogenase, g2125 1,4-alpha-glucan branching, g2126 Peroxisomal Protein Importer, g2127 Membrane coat complex Retromer Vps5, PX, g2128 ribA, RIB1 GTP cyclohydrolase II, g2129 hypothetical, g2130 S-adenosyl-L-methionine-dependent methyltransferase, g2132 hypothetical, g2133 Regulator of chromosome condensation, RCC1, g2134 RNI-like, g2135 Aldehyde dehydrogenase, g2136 Homoserine acetyltransferase, g2137 hypothetical, g2138 Vacuolar assembly/sorting protein Snf7, g2139 Inositol-1,4,5-triphosphate 5-phosphatase (synaptojanin), INP51/INP52/INP53 family | |
| g756 | branched-chain amino acid aminotransferase, Methionine aminotransferase BCAT4 *Arabidopsis thaliana* Q9LE06 [169, 3e-49  119/374 32%, 399] | 677 | g757 Manganese/iron superoxide dismutase, g758 Velvet, g759 UvrD-like Helicase | |
| **Geosmin biosynthesis pathway** | | | | |
| g5920 | Geosmin: germacradienol/ geosmin synthases | 11698 | g5921 mitochondrial protein MBA1, g5922 Uncharacterized conserved protein | |
| g2958 | Geosmin,,Limonene: germacradienol/ geosmin/ limonene synthases | 3429 | g2954 Mitochondrial ATP-dependent protease, g2955 NIPBL cohesin loading factor, g2956 Phosphatidylinositol transfer protein SEC14, g2957 Tetratricopeptide repeat, g2959 hypothetical, g2960 Ubiquitin activating E1, g2961 Metallo-dependent hydrolases, g2962 Uncharacterized conserved protein, g2963 Carnitine O-acetyltransferase, g2964 BTB/POZ fold | |
| **Limonene biosynthesis pathway** | | | | |
| g4091 | Limonene: Geranyl diphosphate lyase (limonene forming) | 5718 | - | |


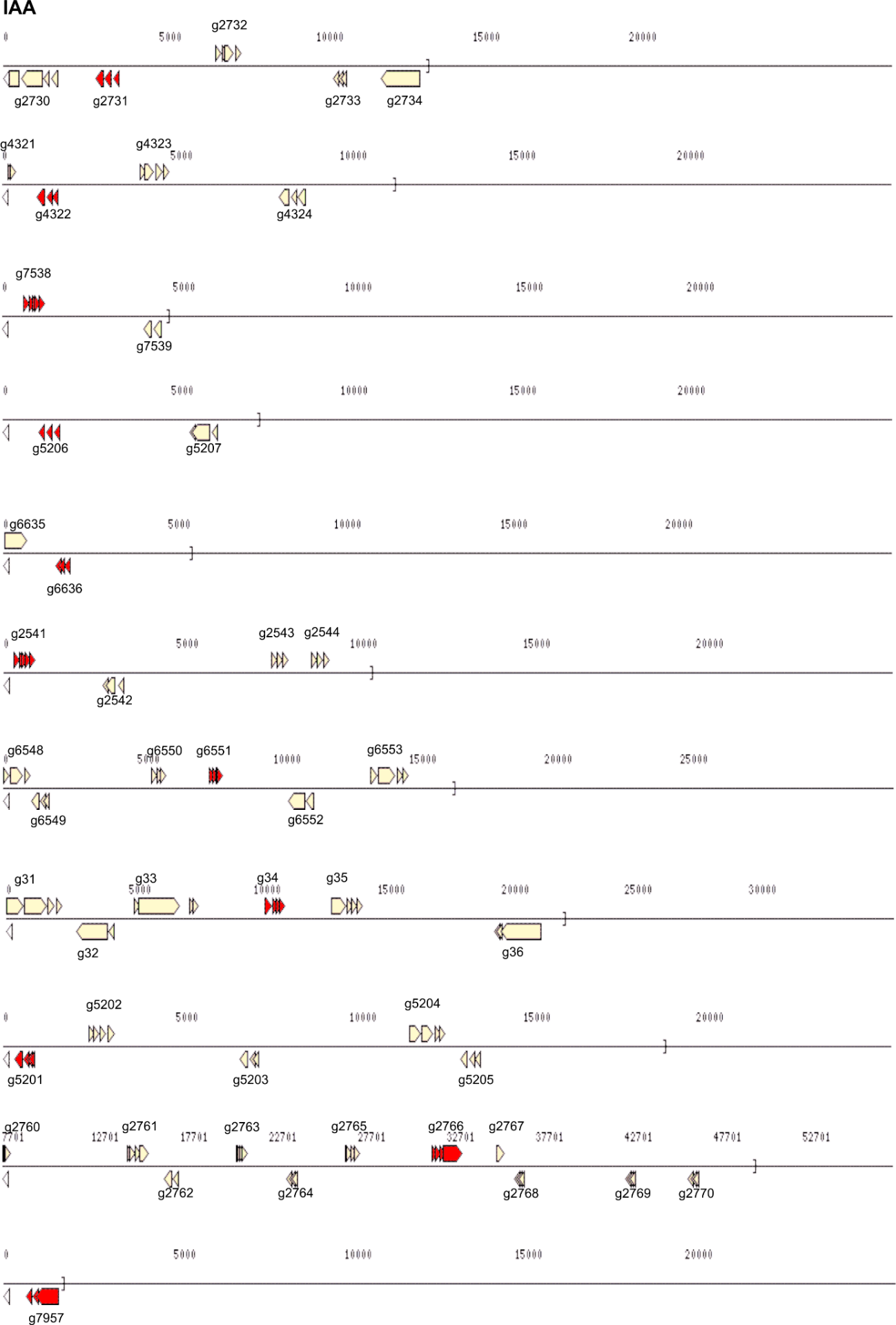


**Supplementary Fig. S4** Contigs of the *T. vaccinum* genome with potential biosynthesis genes (red arrows) of indole3-acetic acid (IAA) encoding g2731 the tryptophan aminotransferase Tam1, g4322 the indole-3-pyruvic acid (IPA) decarboxylase Ipd1, several aldehyde dehydrogenases with g7538 Ald1, g5206 Ald2, g6636 Ald3, g2541 Ald4, g6551 Ald5, g34 Ald6, g5201 Ald7, g2766 the prephenate dehydrogenase TyrA and g7957 an auxin efflux carrier transmembrane protein.

**
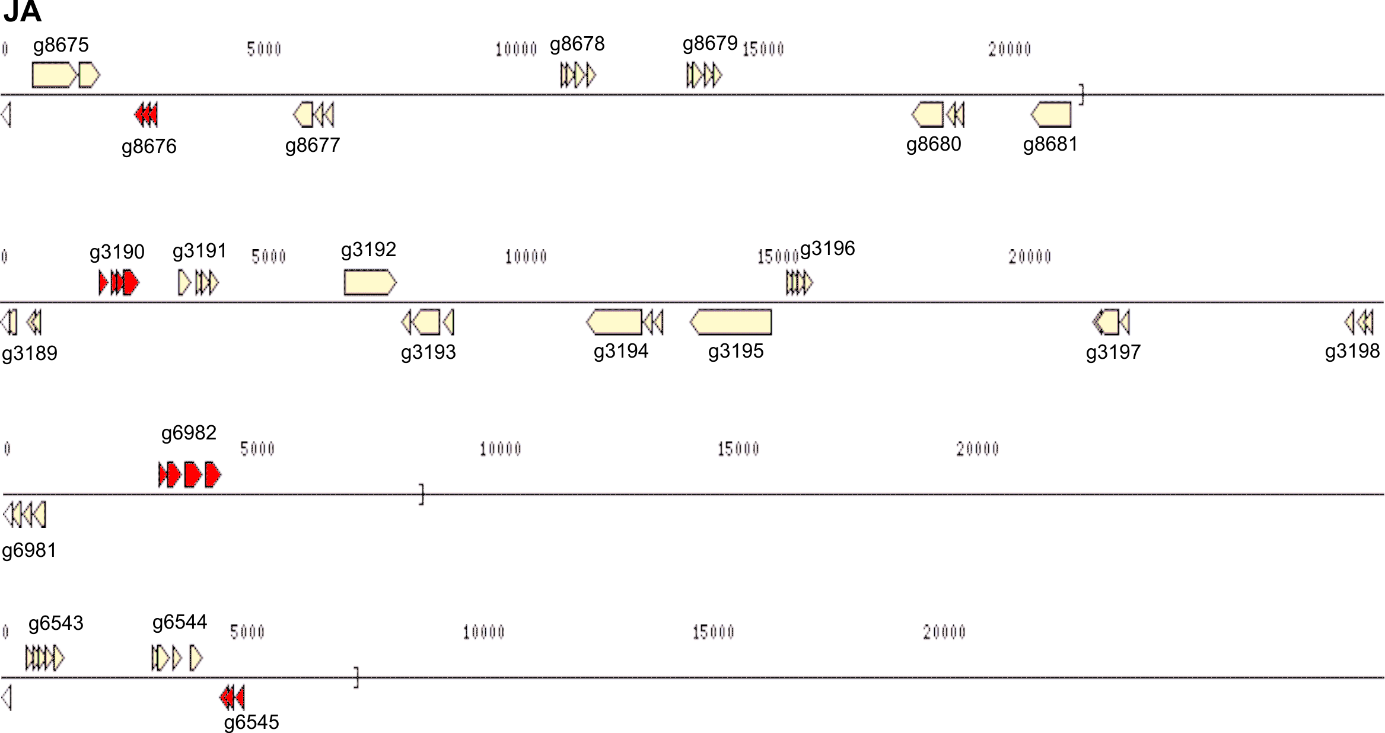
**

**Supplementary Fig. S5** Contigs of the *T. vaccinum* genome with potential biosynthesis genes (red arrows) involved in jasmonic acid (JA) biosynthesis. Gene g8676 codes for an acyl-coenzyme A oxidase, g3190 an enoyl-CoA hydratase, g6982 an acetyl-CoA acyltransferase and g6545 a jasmonoyl-isoleucine-12-hydroxylase.

**
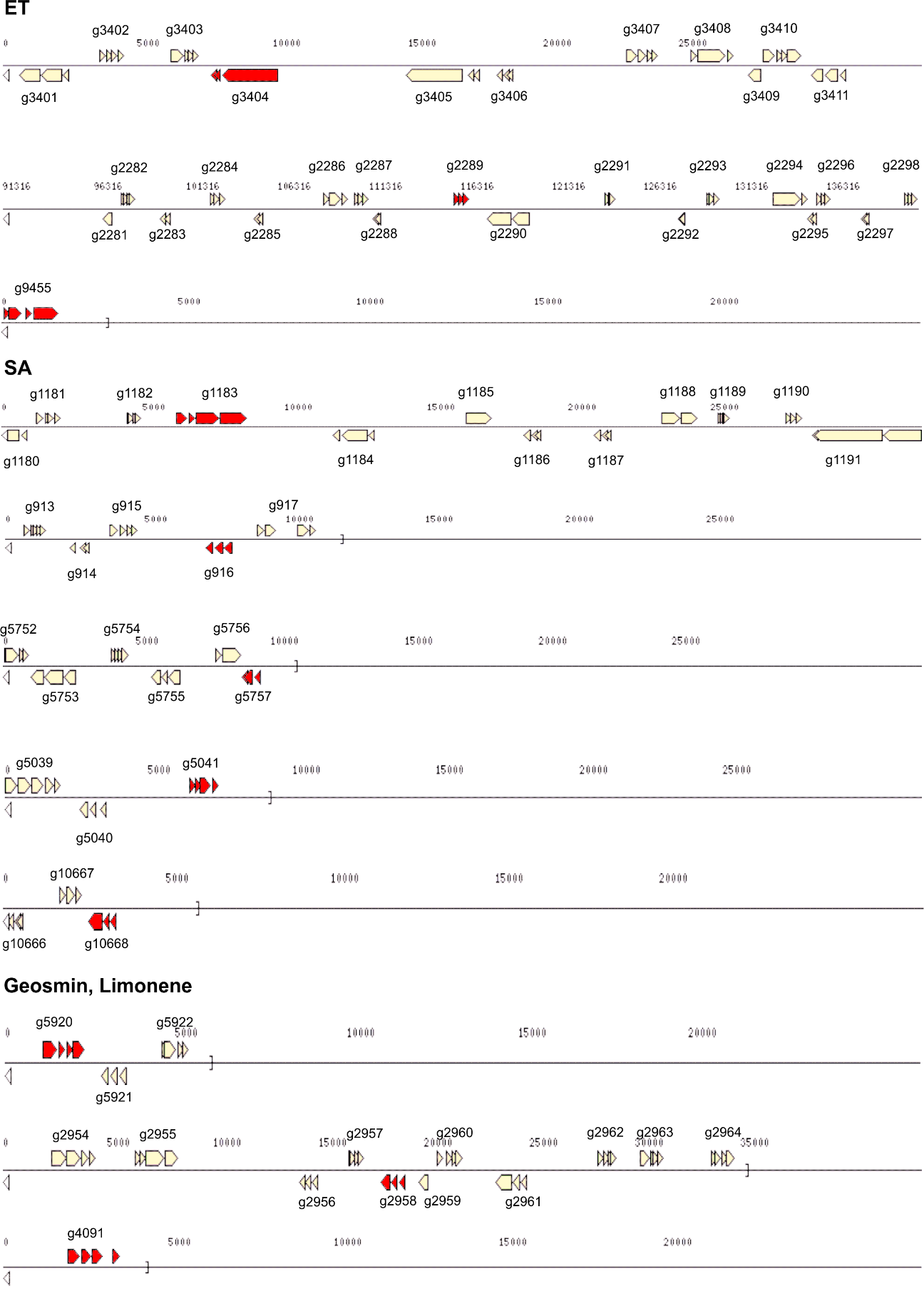
**

**Supplementary Fig. S6** Contigs of the *T. vaccinum* genome with potential biosynthesis genes (red arrows) for salicylic acid (SA) encoding g1183 3-dehydroquinate dehydratase, g916 a chorismate synthase, g5757 an isochorismate synthase, g5041 and g10668 both salicylate monooxygenases.

**
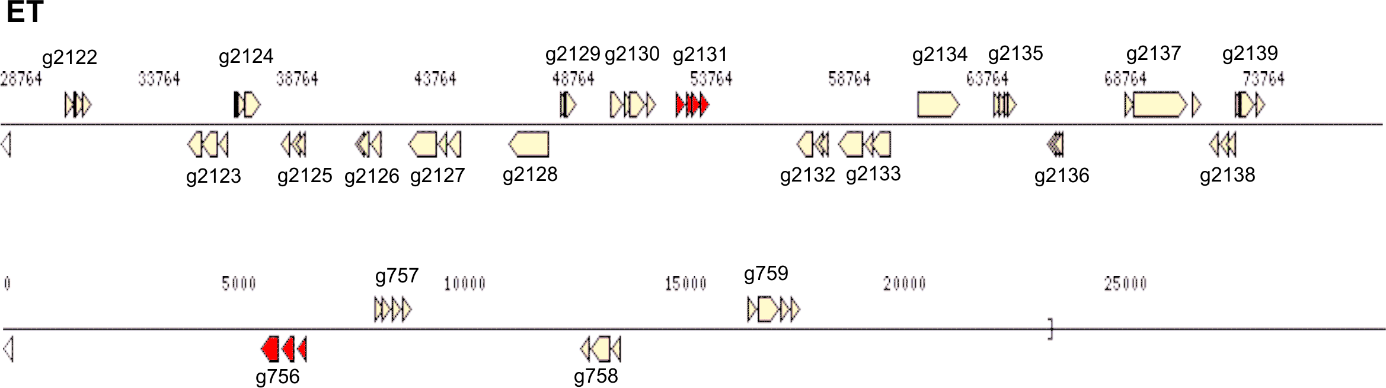
**

**Supplementary Fig. S7** Contigs of the *T. vaccinum* genome with potential biosynthesis genes (red arrows) for ethylene (ET) encoding g2131 an oxoglutarate/iron-dependent dioxygenase, and g756 encoding a branched-chain amino acid aminotransferase.

**
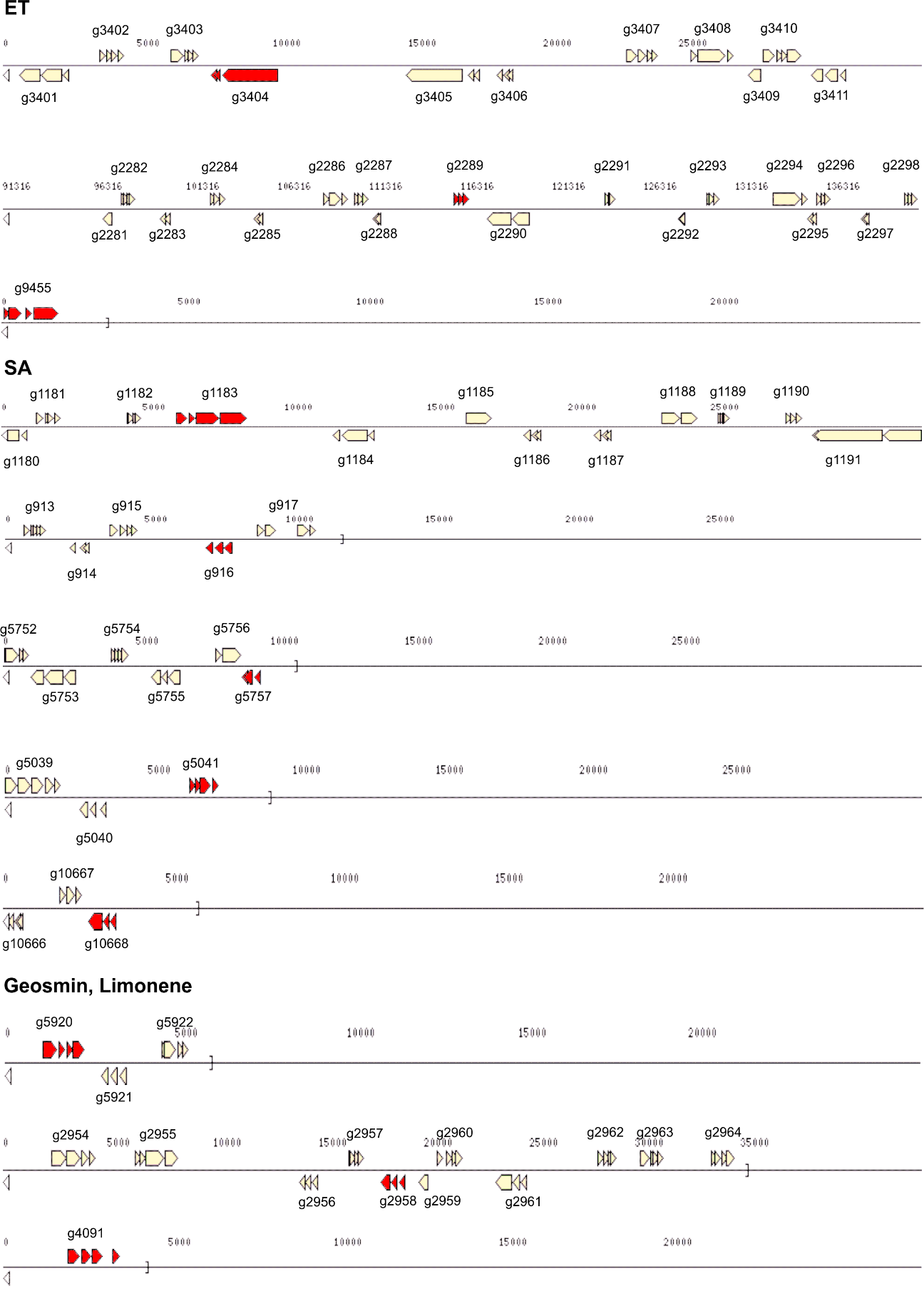
**

**Supplementary Fig. S8** Contigs of the *T. vaccinum* genome with potential biosynthesis genes (red arrows) for the volatile terpenoids geosmin and limonene encoding g5920 germacra­dienol/geosmin synthases, for geosmin and limonene g2958 germacradienol/ geosmin/limo­nene synthases and for limonene g4091 limonene forming geranyl diphosphate lyase.

**Supplementary Table S3** Blast analyses of possible limonene synthesis genes.

| Gene ID | Organism, protein name, accession number, score, identical amino acids |
| --- | --- |
| g2958 | *Hypoxylon sp.,* terpenoid synthase, OTA69335, Score=50.4, ID= 27% |
|  | *F. pinicola*, hypothetical protein, EPS98999.1, Score=57.4, ID= 29% |
|  | *P. involutus*, hypothetical protein, KIJ14442.1, Score=45.8, ID= 25% |
| g4091 | *A. terreus,* aristolochene synthase, AAF13263.1, Score=85.1, ID= 27% |
|  | *F. pinicola*, hypothetical protein, EPS98999.1, Score=41.2, ID= 24% |
|  | *F. pinicola*, hypothetical protein, KIJ18360.1, Score=29.3, ID= 34% |
|  | *P. involutus*, hypothetical protein, EPT02807.1, Score=29.3, ID= 40% |
