# Supplementary material for: Phytohormones and volatile organic compounds, like geosmin, in the ectomycorrhiza of Tricholoma vaccinum and Norway spruce (Picea abies)
Source: Mycorrhiza. 2020 Nov 18;31(2):173–88. doi: 10.1007/s00572-020-01005-2 (PMC7910269; doi:10.1007/s00572-020-01005-2)
Supplement: Supplementary file 2 — Supplementary file2 (PDF 9514 KB) [file 572_2020_1005_MOESM2_ESM.pdf]

test_id	gene ID	value_pure fungus	value_mycorrhiza	log2(fold_change)	test_stat	p_value	q_value	significant
XLOC_000001	g5478	0	0	0	0	1	1	no
XLOC_000002	g11496	0	0	0	0	1	1	no
XLOC_000003	g1088	105,073	80,7344	-0,38014	-0,612456	0,29515	0,557058	no
XLOC_000004	g1089	23,9014	15,4504	-0,629456	-1,04313	0,0625	0,22556	no
XLOC_000005	g1087	100,44	132,795	0,402864	0,639465	0,27225	0,533632	no
XLOC_000006	g11497	31,8066	36,642	0,204171	0,254352	0,6566	0,8318	no
XLOC_000007	g5479	0	0	0	0	1	1	no
XLOC_000008	g11498	0	0	0	0	1	1	no
XLOC_000009	g11499	20,4869	7,83847	-1,38606	-1,543	0,01145	0,0731073	no
XLOC_000010	g5480	0,806989	1,90473	1,23896	1,14417	0,0548	0,208499	no
XLOC_000011	g5481	0	0	0	0	1	1	no
XLOC_000012	g11501	153,317	273,422	0,834608	1,39142	0,01545	0,0903627	no
XLOC_000013	g11500	173,536	160,211	-0,115259	-0,198897	0,73055	0,872113	no
XLOC_000014	g11502	154,078	91,1178	-0,757856	-1,27957	0,0259	0,1295	no
XLOC_000015	g5482	48,1068	140,033	1,54145	2,26888	0,00015	0,00294012	yes
XLOC_000016	g11504	37,0395	38,3152	0,048854	0,084505	0,8836	0,94799	no
XLOC_000017	g11503	46,2647	40,6508	-0,186626	-0,30721	0,5959	0,793092	no
XLOC_000018	g11505	85,7686	86,6498	0,0147464	0,0252252	0,9665	0,985364	no
XLOC_000019	g11507	4,51412	4,20752	-0,101477	-0,109571	0,85135	0,933273	no
XLOC_000020	g11508	15,898	9,09772	-0,805269	-0,754776	0,20785	0,462412	no
XLOC_000021	g11506	26,1237	23,7661	-0,136453	-0,224482	0,7021	0,858715	no
XLOC_000022	g5483	0	0	0	0	1	1	no
XLOC_000023	g11509	10,1448	5,59266	-0,859135	-0,719904	0,2258	0,483822	no
XLOC_000024	g5484	193,658	147,35	-0,394265	-0,624485	0,27445	0,536213	no
XLOC_000025	g11510	0	0	0	0	1	1	no
XLOC_000026	g11511	0,414118	1,29957	1,64992	1,44762	0,01735	0,0979178	no
XLOC_000027	g5485	34,8056	41,2759	0,24598	0,378475	0,49435	0,724879	no
XLOC_000028	g5486	0,0277557	0	#NAME?	0	1	1	no
XLOC_000029	g11512	0	0,433229	inf	0	1	1	no
XLOC_000030	g11513	17,8293	34,7031	0,960812	1,14421	0,04475	0,182342	no
XLOC_000031	g5487	0	0	0	0	1	1	no
XLOC_000032	g11515	582,833	594,948	0,0296802	0,0375549	0,94415	0,975337	no
XLOC_000033	g11514	358,279	352,139	-0,024935	-0,0415582	0,94125	0,973981	no
XLOC_000034	g11516	68,9353	73,7247	0,0969042	0,166335	0,76585	0,890392	no
XLOC_000035	g5488	0	0	0	0	1	1	no
XLOC_000036	g5489	40,6297	107,41	1,40252	1,49543	0,0076	0,0552012	no
XLOC_000037	g1091	27,2596	17,8532	-0,610578	-1,03048	0,0711	0,245241	no
XLOC_000038	g1092	9,6538	6,53927	-0,561968	-0,699042	0,23285	0,490579	no
XLOC_000039	g1090	84,962	153,719	0,85541	1,46215	0,00995	0,0662434	no
XLOC_000040	g11517	12,898	15,6947	0,283128	0,458781	0,41255	0,66501	no
XLOC_000041	g11518	293,579	317,785	0,114305	0,177958	0,7452	0,880033	no
XLOC_000042	g11519	33,8611	32,0645	-0,078649	-0,12687	0,81765	0,917113	no
XLOC_000043	g5490	22,5102	13,8018	-0,705719	-1,09285	0,0635	0,228247	no
XLOC_000044	g5491	24,8273	23,6493	-0,0701324	-0,116619	0,8344	0,924287	no
XLOC_000045	g11520	28,5305	26,2966	-0,117627	-0,170753	0,7559	0,884596	no
XLOC_000046	g1093	59,4594	54,3942	-0,128451	-0,213728	0,7081	0,861576	no
XLOC_000047	g1094	88,5018	90,9583	0,0394982	0,0650405	0,90955	0,961544	no
XLOC_000048	g1097	19,6342	19,0069	-0,0468411	-0,0725721	0,89425	0,952961	no
XLOC_000049	g1095	21,3929	16,9903	-0,332418	-0,540271	0,33465	0,594799	no
XLOC_000050	g1096	55,3835	179,461	1,69614	2,76973	5,00E-05	0,00120049	yes
XLOC_000051	g1098	9,0451	4,25494	-1,088	-1,63687	0,00355	0,031924	yes
XLOC_000052	g11521	0	0	0	0	1	1	no
XLOC_000053	g5492	1,24283	4,76188	1,9379	1,94192	0,0015	0,0168343	yes
XLOC_000054	g11522	55,7032	78,9249	0,502721	0,732318	0,20585	0,460047	no
XLOC_000055	g11523	14,8343	10,5129	-0,496772	-0,587982	0,30315	0,564534	no
XLOC_000056	g5493	23,8661	42,6003	0,835903	1,11488	0,0551	0,208993	no
XLOC_000057	g5494	527,628	0	#NAME?	0	1	1	no
XLOC_000058	g11524	0	0	0	0	1	1	no
XLOC_000059	g11525	0	0	0	0	1	1	no
XLOC_000060	g11526	0	0,190664	inf	0	1	1	no
XLOC_000061	g5495	434,436	577,551	0,410806	0,660835	0,23815	0,496104	no
XLOC_000062	g11527	297,322	515,03	0,79263	1,21464	0,02865	0,137614	no
XLOC_000063	g5496	1777,28	5163,91	1,53879	2,33799	0,0001	0,00209829	yes
XLOC_000064	g5497	64,8194	59,9156	-0,113493	-0,182794	0,74645	0,88092	no
XLOC_000065	g5498	0	0	0	0	1	1	no
XLOC_000066	g5500	374,069	154,446	-1,2762	-1,87788	0,0016	0,0176341	yes
XLOC_000067	g5502	35,8139	23,1405	-0,6301	-0,95351	0,1	0,303086	no
XLOC_000068	g5499	163,237	130,984	-0,317581	-0,536615	0,3466	0,606704	no
XLOC_000069	g5501	27,9792	14,8181	-0,916996	-1,42077	0,01115	0,0717987	no
XLOC_000070	g11528	6,27337	8,08289	0,365632	0,497143	0,3698	0,629256	no
XLOC_000071	g11529	12,8903	80,0409	2,63445	3,54224	5,00E-05	0,00120049	yes
XLOC_000072	g1099	0	0	0	0	1	1	no
XLOC_000073	g5503	2,2143	2,47162	0,158607	0,132216	0,82565	0,920778	no
XLOC_000074	g5504	46,5502	42,7593	-0,122547	-0,19994	0,71725	0,865707	no
XLOC_000075	g11530	0	0	0	0	1	1	no
XLOC_000076	g11531	60,7021	43,261	-0,488679	-0,730557	0,1771	0,424073	no
XLOC_000077	g11532	27,6742	33,4483	0,273394	0,460219	0,40105	0,654966	no
XLOC_000078	g11533	501,257	627,93	0,325054	0,537409	0,3484	0,608857	no
XLOC_000079	g1100	16,0592	44,4716	1,46948	2,07526	0,00035	0,00552572	yes
XLOC_000080	g5505	0	0	0	0	1	1	no
XLOC_000081	g5506	0	0	0	0	1	1	no
XLOC_000082	g5507	27,5595	53,6855	0,961985	1,43616	0,015	0,0885749	no
XLOC_000083	g11534	25,9381	31,4476	0,277879	0,437131	0,43085	0,678624	no
XLOC_000084	g11535	61,6097	74,8687	0,281206	0,37129	0,5298	0,750633	no
XLOC_000085	g5508	18,7632	0,503979	-5,2184	-4,12556	0,00245	0,0243512	yes
XLOC_000086	g11536	17,1395	37,7789	1,14026	7,56693	0,45785	0,701013	no
XLOC_000087	g5509	122,933	123,897	0,0112732	0,0186849	0,9741	0,987624	no
XLOC_000088	g5510	4,95472	6,04793	0,287637	0,376879	0,5165	0,741989	no
XLOC_000089	g5511	114,002	84,7264	-0,428171	-0,696309	0,21495	0,470651	no
XLOC_000090	g5512	17,6178	7,68048	-1,19777	-1,53272	0,01345	0,082241	no
XLOC_000091	g1101	1,32857	0,90398	-0,555517	0	1	1	no

XLOC_000092	g1102	0,217699	0,118749	-0,874418	0	1	1	no
XLOC_000093	g1103	34,8297	34,219	-0,0255187	-0,0432025	0,9399	0,973199	no
XLOC_000094	g5513	14,4507	21,2036	0,553166	0,707511	0,20775	0,462294	no
XLOC_000095	g5514	6,84475	15,6612	1,19412	2,62653	0,32105	0,581038	no
XLOC_000096	g5515	81,0062	61,2059	-0,404361	-0,637386	0,2612	0,521008	no
XLOC_000097	g5516	31,9981	35,0053	0,12959	0,221011	0,69985	0,857351	no
XLOC_000098	g5518	105,664	102,685	-0,0412628	-0,0654756	0,90855	0,961108	no
XLOC_000099	g5517	38,447	42,5123	0,145009	0,239091	0,67915	0,845708	no
XLOC_000100	g5519	104,098	39,4619	-1,39941	-2,21799	5,00E-05	0,00120049	yes
XLOC_000101	g5520	55,6974	28,1616	-0,983884	-1,63183	0,00365	0,0326439	yes
XLOC_000102	g5521	35,3763	26,435	-0,420331	-0,648923	0,2455	0,50414	no
XLOC_000103	g5522	3,63711	3,84365	0,0796821	0,0861104	0,87825	0,945452	no
XLOC_000104	g1106	19,3565	5,00365	-1,95177	-1,9227	0,00455	0,0383857	yes
XLOC_000105	g1104	1,63459	1,20842	-0,435807	-0,514885	0,36585	0,625461	no
XLOC_000106	g1105	526,958	230,184	-1,1949	-1,61349	0,00785	0,0561859	no
XLOC_000107	g1107	85,038	52,3151	-0,700881	-1,15216	0,04735	0,189169	no
XLOC_000108	g5523	0	0	0	0	1	1	no
XLOC_000109	g5525	28,9302	31,6861	0,131274	0,222672	0,69455	0,85459	no
XLOC_000110	g5524	79,8101	78,0269	-0,0325991	-0,0457589	0,9379	0,972153	no
XLOC_000111	g11537	1,65563	1,85596	0,164789	0,146051	0,8016	0,908624	no
XLOC_000112	g11538	48,9645	23,2238	-1,07613	-1,66888	0,0041	0,0354419	yes
XLOC_000113	g1108	0	0	0	0	1	1	no
XLOC_000114	g11539	24,7624	23,3773	-0,0830376	-0,115885	0,8338	0,924151	no
XLOC_000115	g11540	5,12048	2,95478	-0,793228	-1,04497	0,06085	0,221971	no
XLOC_000116	g11541	47,4552	39,3526	-0,27011	-0,403703	0,4732	0,710415	no
XLOC_000117	g11542	170,477	232,544	0,447927	0,691719	0,2238	0,481111	no
XLOC_000118	g11543	16,8226	8,98888	-0,904188	-1,2482	0,03295	0,150357	no
XLOC_000119	g11544	344,309	663,555	0,94651	1,35772	0,0171	0,0969527	no
XLOC_000120	g11545	22,3376	37,7303	0,756253	1,06179	0,0652	0,231644	no
XLOC_000121	g5526	0	0	0	0	1	1	no
XLOC_000122	g5527	7,01285	7,69617	0,134141	0,205871	0,72015	0,867502	no
XLOC_000123	g11546	0	0	0	0	1	1	no
XLOC_000124	g5528	38,803	61,1998	0,657359	0,543919	0,35445	0,615463	no
XLOC_000125	g5529	61,7725	64,0346	0,0518873	0,0907457	0,87795	0,945394	no
XLOC_000126	g5530	1,98339	2,40733	0,27946	0,327571	0,5641	0,773776	no
XLOC_000127	g5532	11,6301	15,8619	0,447703	0,618811	0,28025	0,542577	no
XLOC_000128	g5533	359,071	829,644	1,20822	1,74409	0,00305	0,028799	yes
XLOC_000129	g5531	65,6713	149,99	1,19153	1,69244	0,0039	0,0342252	yes
XLOC_000130	g5534	1,6074	2,19588	0,450069	0,411113	0,4818	0,7161	no
XLOC_000131	g11547	0	0,216615	inf	0	1	1	no
XLOC_000132	g11548	0	0	0	0	1	1	no
XLOC_000133	g11549	0	0,133996	inf	0	1	1	no
XLOC_000134	g11550	62,4779	62,182	-0,00684764	-0,0104455	0,98655	0,993225	no
XLOC_000135	g5535	0	0	0	0	1	1	no
XLOC_000136	g11551	1,87795	2,58463	0,460796	0,665394	0,2465	0,505345	no
XLOC_000137	g5536	6,71861	15,8393	1,23727	1,6728	0,0052	0,0421668	yes
XLOC_000138	g5537	68,8958	53,3853	-0,367975	-0,593869	0,29635	0,558451	no
XLOC_000139	g5538	121,908	108,072	-0,173804	-0,292593	0,61335	0,805658	no
XLOC_000140	g11553	2,75004	4,95971	0,850803	0,981987	0,1028	0,308903	no
XLOC_000141	g11555	907,407	739,179	-0,295826	-0,463929	0,4142	0,666219	no
XLOC_000142	g11552	37,1147	22,9255	-0,695037	-1,06155	0,07115	0,245328	no
XLOC_000143	g11554	82,8946	37,2234	-1,15507	-1,86427	0,0011	0,0134856	yes
XLOC_000144	g11556	20,6734	24,1603	0,224857	0,266221	0,6459	0,825555	no
XLOC_000145	g5540	9,66692	8,93615	-0,113403	-0,160983	0,7849	0,900961	no
XLOC_000146	g5542	17,3737	28,8597	0,732154	1,08773	0,0583	0,216122	no
XLOC_000147	g5539	34,4005	40,0986	0,221126	0,351296	0,5321	0,752589	no
XLOC_000148	g5541	31,6529	39,0385	0,302556	0,508382	0,37885	0,636385	no
XLOC_000149	g5543	62,1942	145,25	1,22368	1,87472	0,00165	0,0180837	yes
XLOC_000150	g11557	76,4981	50,5009	-0,599113	-0,786944	0,1719	0,416907	no
XLOC_000151	g5544	28,5871	40,4693	0,501467	0,570036	0,3132	0,573795	no
XLOC_000152	g11558	59,2936	84,7954	0,516109	0,747224	0,18585	0,433709	no
XLOC_000153	g11559	0,878952	2,62043	1,57595	1,75582	0,00375	0,0333862	yes
XLOC_000154	g11560	0	0,372387	inf	0	1	1	no
XLOC_000155	g1109	125,246	302,636	1,27281	1,91974	0,00145	0,0164423	yes
XLOC_000156	g1110	26,3009	28,3778	0,109648	0,140313	0,8068	0,910938	no
XLOC_000157	g11561	10,1892	34,392	1,75504	2,10436	0,00045	0,00669545	yes
XLOC_000158	g5545	0	0	0	0	1	1	no
XLOC_000159	g5546	0	0	0	0	1	1	no
XLOC_000160	g5547	28,5023	39,2359	0,461096	0,676081	0,2482	0,507023	no
XLOC_000161	g5548	0	0	0	0	1	1	no
XLOC_000162	g5549	13,7088	15,1846	0,1475	0,192759	0,72745	0,870385	no
XLOC_000163	g11562	118,862	64,9199	-0,87255	-1,26616	0,0304	0,142632	no
XLOC_000164	g5551	33,0161	18,3234	-0,849486	-1,31848	0,02655	0,13181	no
XLOC_000165	g5553	196,079	476,943	1,28238	1,6338	0,0064	0,048795	yes
XLOC_000166	g5554	30,17	45,1031	0,580109	0,918504	0,1052	0,314001	no
XLOC_000167	g5550	67,1145	73,901	0,138971	0,226319	0,68795	0,850292	no
XLOC_000168	g5552	5,68545	8,0347	0,49897	0,588572	0,30165	0,563478	no
XLOC_000169	g5555	11,5459	13,4653	0,221867	0,297947	0,6049	0,79937	no
XLOC_000170	g5557	36,0272	62,801	0,801701	1,17687	0,04725	0,189046	no
XLOC_000171	g5556	28,2758	22,5702	-0,325148	-0,539948	0,3443	0,604618	no
XLOC_000172	g5558	694,284	256,756	-1,43513	-1,78533	0,00245	0,0243512	yes
XLOC_000173	g11563	27,4017	16,7999	-0,705816	-0,521827	0,6033	0,798868	no
XLOC_000174	g11564	1,41443	0,0726457	-4,2832	-0,997347	0,112	0,326944	no
XLOC_000175	g5559	0,341363	0	#NAME?	0	1	1	no
XLOC_000176	g5560	27,8467	16,647	-0,742242	-1,02351	0,08425	0,273771	no
XLOC_000177	g5561	14,5873	6,58937	-1,1465	-1,55001	0,0069	0,051293	no
XLOC_000178	g5563	467,522	335,157	-0,480201	-0,687497	0,23025	0,487612	no
XLOC_000179	g5562	35,3283	22,5683	-0,646527	-0,96172	0,0987	0,301379	no
XLOC_000180	g5565	0	0	0	0	1	1	no
XLOC_000181	g5564	0	0	0	0	1	1	no
XLOC_000182	g5566	28,0107	38,9222	0,474612	0,810879	0,15395	0,39191	no
XLOC_000183	g1111	25,8046	18,6371	-0,46945	-0,667803	0,24875	0,507527	no

XLOC_000184	g1113	55,7602	44,5527	-0,323724	-0,55187	0,32375	0,584202	no
XLOC_000185	g1115	89,5851	107,517	0,263229	0,454326	0,4302	0,678318	no
XLOC_000186	g1119	59,3521	134,892	1,18443	1,96025	0,00115	0,0138564	yes
XLOC_000187	g1121	11,4633	5,78247	-0,987259	-1,27245	0,03585	0,158223	no
XLOC_000188	g1123	32,7059	45,8397	0,487046	0,771026	0,1829	0,429992	no
XLOC_000189	g1124	67,342	64,8047	-0,0554071	-0,0921641	0,8727	0,942536	no
XLOC_000190	g1127	13,802	13,8549	0,00551921	0,00871801	0,98775	0,993619	no
XLOC_000191	g1129	47,2646	45,3117	-0,060877	-0,0993032	0,86365	0,936821	no
XLOC_000192	g1131	327,295	520,971	0,670612	1,11715	0,04595	0,185691	no
XLOC_000193	g1133	10,8684	5,74417	-0,919966	-1,31131	0,0224	0,118009	no
XLOC_000194	g1134	32,4224	30,1559	-0,104548	-0,178551	0,74985	0,881965	no
XLOC_000195	g1112	30,6641	20,8007	-0,559922	-0,853525	0,1237	0,347009	no
XLOC_000196	g1114	124,106	174,854	0,494577	0,677107	0,21995	0,477328	no
XLOC_000197	g1116	66,7265	112,85	0,75807	1,28038	0,02375	0,122944	no
XLOC_000198	g1117	40,613	57,5817	0,503666	0,857899	0,135	0,365408	no
XLOC_000199	g1118	13,8088	22,4565	0,701546	0,957876	0,09045	0,285601	no
XLOC_000200	g1120	64,412	49,9441	-0,367015	-0,623203	0,2841	0,546579	no
XLOC_000201	g1122	49,3553	66,2711	0,425176	0,712937	0,2062	0,46062	no
XLOC_000202	g1125	71,9133	96,7002	0,427262	0,703747	0,1998	0,45229	no
XLOC_000203	g1126	65,1115	53,0451	-0,295692	-0,501518	0,36595	0,625499	no
XLOC_000204	g1128	276,331	382,072	0,467446	0,759441	0,1857	0,433668	no
XLOC_000205	g1130	6,34382	8,47041	0,417081	0,630003	0,24965	0,508092	no
XLOC_000206	g1132	4,45602	1,51407	-1,55733	-1,43194	0,0375	0,163304	no
XLOC_000207	g11565	0,680653	0,262494	-1,37463	0	1	1	no
XLOC_000208	g11566	0	0	0	0	1	1	no
XLOC_000209	g5567	4,50072	4,77126	0,0842153	0,0892274	0,8786	0,945518	no
XLOC_000210	g11567	0	0	0	0	1	1	no
XLOC_000211	g11568	22,1915	13,6964	-0,696206	-0,818141	0,16795	0,410959	no
XLOC_000212	g11569	119,98	59,2095	-1,01889	-1,12208	0,05125	0,199633	no
XLOC_000213	g5568	2,67508	1,64204	-0,704096	-0,631561	0,2768	0,538359	no
XLOC_000214	g5569	26,6235	17,1069	-0,638128	-1,07633	0,05965	0,219634	no
XLOC_000215	g5570	61,5482	42,6348	-0,529687	-0,834652	0,1527	0,390498	no
XLOC_000216	g11570	30,5164	13,4437	-1,18266	-1,66651	0,0044	0,0374419	yes
XLOC_000217	g5571	0	0	0	0	1	1	no
XLOC_000218	g5572	57,0764	165,454	1,53547	2,29966	5,00E-05	0,00120049	yes
XLOC_000219	g5573	48,2343	139,619	1,53337	2,23749	0,00015	0,00294012	yes
XLOC_000220	g11571	0	0	0	0	1	1	no
XLOC_000221	g11572	22,0753	27,2437	0,303487	0,368137	0,51835	0,743094	no
XLOC_000222	g5574	0	0	0	0	1	1	no
XLOC_000223	g1135	232,645	131,197	-0,826394	-1,37159	0,01625	0,0933187	no
XLOC_000224	g1136	87,1497	42,675	-1,03011	-1,64419	0,0044	0,0374419	yes
XLOC_000225	g5575	0	0	0	0	1	1	no
XLOC_000226	g5576	488,498	392,882	-0,314255	-0,429266	0,43345	0,680492	no
XLOC_000227	g5577	28,6259	19,5605	-0,549375	-0,887583	0,12595	0,350079	no
XLOC_000228	g5578	5,65471	9,01821	0,673388	0,907797	0,12435	0,347599	no
XLOC_000229	g5580	102,246	241,949	1,24265	1,9449	0,00125	0,0146655	yes
XLOC_000230	g5579	22,4387	22,6858	0,0158055	0,0268739	0,9627	0,983635	no
XLOC_000231	g5581	88,1499	102,084	0,211724	0,272554	0,6355	0,818012	no
XLOC_000232	g5582	333,479	145,402	-1,19755	-1,67112	0,00555	0,0439524	yes
XLOC_000233	g5583	2255,98	1039,19	-1,1183	-1,53778	0,0058	0,045492	yes
XLOC_000234	g5584	35,1143	47,3343	0,430827	0,693562	0,2287	0,486954	no
XLOC_000235	g1137	0	0	0	0	1	1	no
XLOC_000236	g1138	0	0	0	0	1	1	no
XLOC_000237	g11573	0	0	0	0	1	1	no
XLOC_000238	g5585	50,9184	38,3414	-0,409284	-0,675452	0,2214	0,478466	no
XLOC_000239	g5586	24,9556	60,8708	1,28639	1,50626	0,01075	0,0699569	no
XLOC_000240	g11574	544,011	452,043	-0,267177	-0,455582	0,4138	0,666012	no
XLOC_000241	g11575	13405,1	7832,75	-0,775192	-0,964022	0,0945	0,293482	no
XLOC_000242	g11577	21,1162	29,6648	0,490403	0,672258	0,24255	0,500911	no
XLOC_000243	g11576	125,051	104,715	-0,256052	-0,429605	0,44865	0,694314	no
XLOC_000244	g5587	797,566	863,288	0,114239	0,177558	0,75355	0,883777	no
XLOC_000245	g11578	19,5953	21,441	0,129866	0,162315	0,77515	0,894895	no
XLOC_000246	g5588	0	0	0	0	1	1	no
XLOC_000247	g5589	44,5895	48,1372	0,110449	0,184905	0,74365	0,879048	no
XLOC_000248	g5590	57,0099	65,6513	0,20361	0,351116	0,5427	0,76057	no
XLOC_000249	g5591	180,539	164,482	-0,134377	-0,23098	0,68405	0,848046	no
XLOC_000250	g5594	28,2575	29,592	0,0665713	0,0941003	0,87125	0,942359	no
XLOC_000251	g5592	93,0643	70,223	-0,406284	-0,636495	0,2537	0,513038	no
XLOC_000252	g5593	46,0468	23,1398	-0,992725	-1,54346	0,007	0,0518011	no
XLOC_000253	g5597	114,183	46,5242	-1,2953	-1,9334	0,00075	0,0100752	yes
XLOC_000254	g5595	11,707	13,3569	0,190215	0,276887	0,6323	0,816139	no
XLOC_000255	g5596	66,3866	67,6844	0,0279315	0,0485708	0,93225	0,970394	no
XLOC_000256	g5598	131,771	216,101	0,713674	1,17429	0,04285	0,177622	no
XLOC_000257	g5600	29,4874	31,1026	0,0769344	0,13285	0,82	0,918071	no
XLOC_000258	g5601	530,292	206,978	-1,35731	-2,21008	0,00025	0,00430702	yes
XLOC_000259	g5599	49,9597	36,9458	-0,435353	-0,729467	0,19885	0,451802	no
XLOC_000260	g11579	304,747	229,081	-0,411753	-0,63941	0,27025	0,531622	no
XLOC_000261	g5602	89,8207	98,7955	0,137397	0,205743	0,7232	0,86956	no
XLOC_000262	g5603	3,95087	6,57913	0,735728	0,933124	0,0989	0,301802	no
XLOC_000263	g11580	97,0359	174,873	0,849716	1,24173	0,0245	0,124723	no
XLOC_000264	g5604	0	0	0	0	1	1	no
XLOC_000265	g5605	19,7837	15,2382	-0,376614	-0,460497	0,42845	0,677386	no
XLOC_000266	g11581	0	0,147857	inf	0	1	1	no
XLOC_000267	g11582	4,18907	6,15141	0,554286	0,573945	0,3199	0,580454	no
XLOC_000268	g11583	190,439	117,554	-0,696013	-1,1093	0,04515	0,183591	no
XLOC_000269	g5606	6,14303	7,03991	0,196607	0,256289	0,65895	0,832458	no
XLOC_000270	g11584	33,5729	41,8105	0,316569	0,477764	0,4041	0,657759	no
XLOC_000271	g11585	54,4335	58,978	0,115682	0,188618	0,73335	0,874074	no
XLOC_000272	g11588	96,724	74,4704	-0,377206	-0,640326	0,2737	0,535281	no
XLOC_000273	g11586	225,713	307,544	0,446303	0,686808	0,23645	0,49424	no
XLOC_000274	g11587	51,1157	59,8666	0,227983	0,383773	0,51955	0,743978	no
XLOC_000275	g11589	30,6409	19,0604	-0,684878	-1,0071	0,0739	0,251193	no

XLOC_000276	g11590	15,3486	50,7966	1,72663	2,6611	5,00E-05	0,00120049	yes
XLOC_000277	g1139	65,3843	47,0627	-0,47436	-0,735277	0,19905	0,451802	no
XLOC_000278	g1140	43,8239	51,1973	0,224349	0,379093	0,50215	0,730752	no
XLOC_000279	g1141	88,3515	51,0923	-0,790149	-1,28776	0,02595	0,129618	no
XLOC_000280	g5607	413,82	476,281	0,20281	0,340973	0,558	0,77025	no
XLOC_000281	g5608	26,7097	16,8837	-0,661735	-0,953905	0,0984	0,300802	no
XLOC_000282	g5610	63,8971	70,0836	0,133326	0,229816	0,68635	0,849396	no
XLOC_000283	g5611	103,554	103,298	-0,00357441	-0,00597184	0,9909	0,995156	no
XLOC_000284	g5613	24,0052	31,9178	0,411017	0,702201	0,21295	0,468347	no
XLOC_000285	g5609	45,7548	39,1744	-0,224011	-0,384694	0,4898	0,722384	no
XLOC_000286	g5612	58,0783	48,8779	-0,248818	-0,416894	0,45605	0,700628	no
XLOC_000287	g5614	816,769	471,946	-0,791307	-1,1235	0,0531	0,204648	no
XLOC_000288	g5615	82,9311	79,6699	-0,0578778	-0,0947245	0,86475	0,937704	no
XLOC_000289	g11591	0	0	0	0	1	1	no
XLOC_000290	g5617	0	0	0	0	1	1	no
XLOC_000291	g5619	0	0	0	0	1	1	no
XLOC_000292	g5616	0	0	0	0	1	1	no
XLOC_000293	g5618	0	0	0	0	1	1	no
XLOC_000294	g5620	0	0	0	0	1	1	no
XLOC_000295	g5621	7,62194	10,8271	0,506419	0,804207	0,1548	0,392851	no
XLOC_000296	g11592	0	0	0	0	1	1	no
XLOC_000297	g11593	11,1817	9,49075	-0,236544	-0,303061	0,6025	0,798521	no
XLOC_000298	g5622	0	0	0	0	1	1	no
XLOC_000299	g5623	0	0	0	0	1	1	no
XLOC_000300	g5624	0	0	0	0	1	1	no
XLOC_000301	g5625	1056,96	1778,44	0,750687	1,23321	0,0318	0,14654	no
XLOC_000302	g5628	42,6359	37,251	-0,19479	-0,33448	0,55355	0,767127	no
XLOC_000303	g5631	33,3433	35,9575	0,108896	0,187777	0,73815	0,876358	no
XLOC_000304	g5633	988,472	1680,95	0,766006	1,12676	0,0576	0,214906	no
XLOC_000305	g5635	196,523	130,061	-0,595512	-1,00623	0,0903	0,285219	no
XLOC_000306	g5626	25,7412	30,8526	0,261314	0,445545	0,42365	0,67307	no
XLOC_000307	g5627	3,9179	6,6955	0,773112	0,99041	0,092	0,289008	no
XLOC_000308	g5629	293,101	493,095	0,750464	1,08989	0,05395	0,206545	no
XLOC_000309	g5630	68,0751	77,328	0,183865	0,264119	0,64725	0,82599	no
XLOC_000310	g5632	2,4892	2,99572	0,267224	0,306672	0,6044	0,79937	no
XLOC_000311	g5634	167,441	190,976	0,189739	0,321603	0,57815	0,78137	no
XLOC_000312	g5636	71,7558	88,6616	0,305214	0,517952	0,37055	0,629539	no
XLOC_000313	g11594	81,8955	60,3707	-0,439934	-0,718801	0,20415	0,457393	no
XLOC_000314	g11596	0	0	0	0	1	1	no
XLOC_000315	g11598	0	0	0	0	1	1	no
XLOC_000316	g11595	0	0	0	0	1	1	no
XLOC_000317	g11597	0	0	0	0	1	1	no
XLOC_000318	g11599	0	0	0	0	1	1	no
XLOC_000319	g11600	0	0	0	0	1	1	no
XLOC_000320	g11601	122,981	192,923	0,649593	0,950596	0,1007	0,304456	no
XLOC_000321	g11603	85,9405	136,963	0,672376	0,936282	0,09925	0,302119	no
XLOC_000322	g11602	4,63092	3,46019	-0,420446	-0,366784	0,51475	0,740817	no
XLOC_000323	g11604	354,005	489,141	0,466484	0,747602	0,1814	0,429034	no
XLOC_000324	g11605	0	0	0	0	1	1	no
XLOC_000325	g5637	5,20036	1,46025	-1,83239	-1,57511	0,0748	0,252591	no
XLOC_000326	g11606	0	0	0	0	1	1	no
XLOC_000327	g5638	47,8851	57,3104	0,25922	0,444173	0,42875	0,677386	no
XLOC_000328	g5639	25,41	41,1135	0,694218	1,07233	0,05955	0,219348	no
XLOC_000329	g5640	0	0	0	0	1	1	no
XLOC_000330	g5641	438,522	160,334	-1,45157	-1,87338	0,0073	0,053577	no
XLOC_000331	g5642	0,865803	0,606893	-0,512596	0	1	1	no
XLOC_000332	g5643	5,47819	5,31785	-0,0428555	-0,0479198	0,93405	0,971293	no
XLOC_000333	g5645	84,1772	66,0704	-0,349425	-0,59235	0,30405	0,565487	no
XLOC_000334	g5644	272,877	245,689	-0,151417	-0,224168	0,7016	0,858424	no
XLOC_000335	g5646	10,2378	18,1506	0,826121	1,05856	0,06495	0,231007	no
XLOC_000336	g5647	96,3276	54,2532	-0,828241	-1,36447	0,01795	0,100553	no
XLOC_000337	g5648	20,5382	15,2549	-0,42904	-0,672913	0,23045	0,48772	no
XLOC_000338	g5649	75,051	304,402	2,02003	2,99962	5,00E-05	0,00120049	yes
XLOC_000339	g5650	0	2,93968	inf	#NAME?	0,00055	0,00788467	yes
XLOC_000340	g5651	0	0	0	0	1	1	no
XLOC_000341	g5652	97,0976	55,5483	-0,805693	-1,20843	0,0354	0,157512	no
XLOC_000342	g5655	18,6966	10,5812	-0,821265	-1,27805	0,0207	0,11175	no
XLOC_000343	g5653	20,2743	20,9476	0,0471336	0,0742089	0,8954	0,953462	no
XLOC_000344	g5654	21,9903	16,7125	-0,395933	-0,571582	0,31135	0,572236	no
XLOC_000345	g1142	27,2214	15,6052	-0,802717	-1,28709	0,0239	0,123395	no
XLOC_000346	g1143	29,1263	8,6381	-1,75354	-2,55289	5,00E-05	0,00120049	yes
XLOC_000347	g5656	48,7901	13,3343	-1,87145	-2,61809	5,00E-05	0,00120049	yes
XLOC_000348	g5659	262,92	133,147	-0,981607	-1,59862	0,0056	0,0440994	yes
XLOC_000349	g5657	17,4469	23,2072	0,411603	0,602318	0,297	0,558938	no
XLOC_000350	g5658	134,058	117,984	-0,184269	-0,303921	0,59265	0,791274	no
XLOC_000351	g5660	162,673	186,708	0,198813	0,303112	0,581	0,783281	no
XLOC_000352	g5661	856,624	1097,13	0,356996	0,527322	0,3914	0,646031	no
XLOC_000353	g5662	76,4856	51,475	-0,571317	-0,982668	0,0927	0,290279	no
XLOC_000354	g11607	0	0	0	0	1	1	no
XLOC_000355	g5663	0	0	0	0	1	1	no
XLOC_000356	g5664	0	0	0	0	1	1	no
XLOC_000357	g5665	82,6883	121,78	0,55852	0,913202	0,1148	0,332398	no
XLOC_000358	g5666	0	0	0	0	1	1	no
XLOC_000359	g5667	116,08	143,505	0,305979	0,433241	0,42925	0,677582	no
XLOC_000360	g5668	224,435	194,571	-0,206003	-0,340153	0,55515	0,768368	no
XLOC_000361	g5669	11,834	7,70646	-0,618803	-0,806969	0,16355	0,405776	no
XLOC_000362	g11608	0	0	0	0	1	1	no
XLOC_000363	g5670	0	0	0	0	1	1	no
XLOC_000364	g5672	34,0452	46,1961	0,440323	0,732854	0,19645	0,447606	no
XLOC_000365	g5671	27,2533	17,5144	-0,637887	-0,945925	0,0897	0,283872	no
XLOC_000366	g1144	17,2369	9,39399	-0,875686	-1,0263	0,07295	0,249343	no
XLOC_000367	g1146	40,9475	19,4301	-1,07548	-1,64467	0,00355	0,031924	yes

XLOC_000368	g1145	11,4519	19,5305	0,77015	1,13256	0,0511	0,199286	no
XLOC_000369	g11609	4,02934	2,91892	-0,465107	-0,333588	0,68495	0,848626	no
XLOC_000370	g11611	44,1905	56,6085	0,357281	0,562946	0,31465	0,575393	no
XLOC_000371	g11610	38,8797	24,7384	-0,652267	-1,07549	0,06065	0,221571	no
XLOC_000372	g11612	89,2539	131,378	0,557738	0,864795	0,12495	0,348384	no
XLOC_000373	g11613	68,1926	103,978	0,608596	1,03531	0,06705	0,236081	no
XLOC_000374	g5673	23,4274	20,688	-0,179405	-0,21735	0,7062	0,860728	no
XLOC_000375	g5674	61,2055	167,997	1,4567	2,31852	5,00E-05	0,00120049	yes
XLOC_000376	g5676	33,3697	34,0889	0,0307637	0,0484731	0,9347	0,971502	no
XLOC_000377	g5675	299,731	249,787	-0,262971	-0,434987	0,4582	0,701013	no
XLOC_000378	g11614	10,3881	7,93742	-0,388186	-0,454092	0,44625	0,692396	no
XLOC_000379	g11615	77,7783	50,2784	-0,629429	-0,902135	0,1153	0,333204	no
XLOC_000380	g11616	87,3067	84,8498	-0,0411807	-0,0695564	0,9028	0,957458	no
XLOC_000381	g5677	45,4882	30,867	-0,559428	-0,762415	0,18285	0,429978	no
XLOC_000382	g5678	15,3728	21,7221	0,498789	0,62556	0,28415	0,546579	no
XLOC_000383	g5679	243,278	105,929	-1,19951	-1,35909	0,0218	0,115905	no
XLOC_000384	g5680	10,0185	18,8024	0,908256	1,2316	0,02865	0,137614	no
XLOC_000385	g5681	11,0139	8,69867	-0,340454	-0,324391	0,5657	0,774849	no
XLOC_000386	g5683	0	0	0	0	1	1	no
XLOC_000387	g5682	0	0	0	0	1	1	no
XLOC_000388	g1147	132,682	204,733	0,625769	0,844761	0,14485	0,380837	no
XLOC_000389	g1148	159,518	275,018	0,785806	1,30721	0,02605	0,129919	no
XLOC_000390	g1150	64,9407	56,6827	-0,196215	-0,338345	0,54905	0,765215	no
XLOC_000391	g1151	323,725	549,849	0,764267	0,964539	0,1004	0,30383	no
XLOC_000392	g1156	15,0074	12,1649	-0,302955	-0,40406	0,4758	0,712433	no
XLOC_000393	g1149	18,3896	16,1099	-0,190937	-0,315666	0,5612	0,772171	no
XLOC_000394	g1152	141,361	162,63	0,202212	0,278792	0,61975	0,808549	no
XLOC_000395	g1153	40,3974	42,268	0,0653037	0,101475	0,8569	0,935212	no
XLOC_000396	g1154	139,424	230,98	0,728285	1,15209	0,0461	0,185915	no
XLOC_000397	g1155	11,4044	102,698	3,17075	2,59512	0,00545	0,0433002	yes
XLOC_000398	g1157	50,0734	42,0025	-0,253566	-0,414479	0,45625	0,700825	no
XLOC_000399	g1158	44,5815	50,6735	0,184785	0,294501	0,5944	0,792155	no
XLOC_000400	g1159	471,833	602,679	0,353112	0,557571	0,32385	0,584275	no
XLOC_000401	g1160	1107,93	2148,18	0,955248	1,35231	0,0198	0,10814	no
XLOC_000402	g1161	160,17	236,215	0,560493	0,875491	0,10795	0,31949	no
XLOC_000403	g5684	11,125	31,8909	1,51934	2,25126	0,00035	0,00552572	yes
XLOC_000404	g11617	239,364	193,094	-0,309904	-0,371091	0,52515	0,747134	no
XLOC_000405	g11618	1343	1857,02	0,467533	0,681671	0,23135	0,488992	no
XLOC_000406	g5685	0	0	0	0	1	1	no
XLOC_000407	g5686	0	0	0	0	1	1	no
XLOC_000408	g5687	53,8135	53,4909	-0,00867463	-0,0138929	0,9805	0,990282	no
XLOC_000409	g5688	66,8231	151,858	1,1843	1,76443	0,00245	0,0243512	yes
XLOC_000410	g5689	0	0	0	0	1	1	no
XLOC_000411	g1165	23,9515	23,9808	0,00176418	0,0025958	0,99665	0,997966	no
XLOC_000412	g1167	45,7502	39,0888	-0,227022	-0,375334	0,5164	0,741989	no
XLOC_000413	g1169	38,1441	66,7201	0,806662	1,35426	0,01955	0,107252	no
XLOC_000414	g1171	34,7232	25,1461	-0,465565	-0,699058	0,2198	0,477108	no
XLOC_000415	g1162	64,8076	11,1154	-2,5436	-3,78473	5,00E-05	0,00120049	yes
XLOC_000416	g1163	56,3186	35,2476	-0,676087	-1,14809	0,04765	0,189673	no
XLOC_000417	g1164	10,944	8,67115	-0,335847	-0,471133	0,4179	0,669829	no
XLOC_000418	g1166	28,6583	43,0763	0,58794	0,918593	0,09625	0,296758	no
XLOC_000419	g1168	36,649	33,4691	-0,130945	-0,192611	0,7258	0,869807	no
XLOC_000420	g1170	30,1295	10,2151	-1,56047	-1,89023	0,0012	0,0143358	yes
XLOC_000421	g5690	41,2993	48,8202	0,24136	0,41242	0,46405	0,705195	no
XLOC_000422	g5691	41,6224	41,1156	-0,0176753	-0,0246136	0,96515	0,984192	no
XLOC_000423	g11619	0	0	0	0	1	1	no
XLOC_000424	g5692	122,208	161,463	0,401864	0,682482	0,23895	0,497032	no
XLOC_000425	g5693	21,852	43,095	0,979752	1,44899	0,01325	0,0814746	no
XLOC_000426	g5694	24,3341	27,9094	0,197775	0,259555	0,65285	0,829686	no
XLOC_000427	g11621	74,4231	82,2189	0,14372	0,248849	0,66275	0,835242	no
XLOC_000428	g11620	702,724	1631,19	1,2149	1,92459	0,0012	0,0143358	yes
XLOC_000429	g11622	0	0	0	0	1	1	no
XLOC_000430	g11623	7,05196	4,42045	-0,673832	-0,675564	0,2207	0,477584	no
XLOC_000431	g11624	0	0	0	0	1	1	no
XLOC_000432	g11625	24,9873	24,2125	-0,0454428	-0,0674784	0,90525	0,958546	no
XLOC_000433	g1172	2,40921	13,8065	2,51872	2,74816	5,00E-05	0,00120049	yes
XLOC_000434	g1173	18,7208	38,3231	1,03357	1,4977	0,0109	0,0706055	no
XLOC_000435	g5695	0	0,156003	inf	0	1	1	no
XLOC_000436	g11626	27,2599	30,8312	0,177611	0,266504	0,637	0,819405	no
XLOC_000437	g11627	80,4355	46,5044	-0,790465	-1,32837	0,02245	0,118209	no
XLOC_000438	g11628	21,5988	29,6471	0,456944	0,589396	0,31115	0,572083	no
XLOC_000439	g1174	87,606	34,1285	-1,36005	-2,17749	0,00015	0,00294012	yes
XLOC_000440	g1175	10,4412	15,0803	0,530381	0,642913	0,25525	0,514799	no
XLOC_000441	g1176	122,054	107,695	-0,180572	-0,242198	0,6772	0,844002	no
XLOC_000442	g1177	0	0	0	0	1	1	no
XLOC_000443	g11629	3,61397	4,0086	0,149513	0,176966	0,7554	0,884596	no
XLOC_000444	g5696	0	0	0	0	1	1	no
XLOC_000445	g5697	15,7458	16,858	0,0984718	0,0656261	0,91525	0,963526	no
XLOC_000446	g11631	10,0163	10,8103	0,110055	0,127456	0,8234	0,919418	no
XLOC_000447	g11632	3,68825	1,78174	-1,04965	-0,985924	0,11335	0,329709	no
XLOC_000448	g11630	29,937	24,2285	-0,305225	-0,44405	0,43765	0,68432	no
XLOC_000449	g11633	2,22158	1,97209	-0,171857	-0,161222	0,78695	0,901522	no
XLOC_000450	g11634	31,4562	8,13119	-1,9518	-2,61436	5,00E-05	0,00120049	yes
XLOC_000451	g5698	49,9479	24,808	-1,00962	-1,5532	0,00625	0,0479867	yes
XLOC_000452	g5699	0	0	0	0	1	1	no
XLOC_000453	g11635	232,714	128,571	-0,855993	-1,3771	0,01805	0,100825	no
XLOC_000454	g5700	8,09911	5,46655	-0,567132	-0,716187	0,2139	0,469385	no
XLOC_000455	g5701	138,356	61,0892	-1,1794	-1,92307	0,00095	0,0120686	yes
XLOC_000456	g11636	0	0	0	0	1	1	no
XLOC_000457	g11639	229,579	301,987	0,395493	0,669971	0,2474	0,506238	no
XLOC_000458	g11641	44,9311	61,8146	0,460233	0,74561	0,18745	0,435992	no
XLOC_000459	g11642	87,1632	246,493	1,49975	2,3666	5,00E-05	0,00120049	yes

XLOC_000460	g11637	98,3834	116,367	0,242191	0,380686	0,49855	0,728319	no
XLOC_000461	g11638	46,644	50,5944	0,117286	0,201529	0,72425	0,86956	no
XLOC_000462	g11640	52,3136	48,461	-0,110361	-0,176859	0,74115	0,877407	no
XLOC_000463	g11643	0	0	0	0	1	1	no
XLOC_000464	g127	32,9	25,5613	-0,364125	-0,456446	0,4206	0,671265	no
XLOC_000465	g5702	0	0	0	0	1	1	no
XLOC_000466	g11644	15,5376	11,7052	-0,408613	-0,487514	0,40125	0,655075	no
XLOC_000467	g11645	61,9795	74,2015	0,259657	0,385166	0,4957	0,725992	no
XLOC_000468	g5703	347,535	303,71	-0,194467	-0,312686	0,5925	0,791274	no
XLOC_000469	g5704	45,9275	86,3442	0,91074	1,46082	0,0109	0,0706055	no
XLOC_000470	g5705	57,2301	51,0441	-0,16503	-0,181249	0,74875	0,881846	no
XLOC_000471	g5706	0,312461	0,802697	1,36118	0	1	1	no
XLOC_000472	g5707	30,447	34,9907	0,20067	0,339099	0,5503	0,765215	no
XLOC_000473	g5708	9,2694	11,433	0,302654	0,348292	0,53925	0,758039	no
XLOC_000474	g5709	665,962	308,112	-1,11199	-1,83192	0,00115	0,0138564	yes
XLOC_000475	g11646	437,21	591,422	0,435863	0,585604	0,30945	0,570452	no
XLOC_000476	g5711	5,49559	3,66856	-0,583061	-0,633639	0,2798	0,54194	no
XLOC_000477	g5710	18,4104	10,8422	-0,763864	-0,989041	0,08695	0,27849	no
XLOC_000478	g1178	0	0	0	0	1	1	no
XLOC_000479	g5712	0	0	0	0	1	1	no
XLOC_000480	g11648	61,7764	54,2724	-0,186839	-0,273673	0,63195	0,816117	no
XLOC_000481	g11647	17,6393	22,4368	0,347071	0,504631	0,381	0,637706	no
XLOC_000482	g1179	19,4056	12,5867	-0,624573	-0,83143	0,15595	0,393684	no
XLOC_000483	g5713	2,05698	1,51552	-0,440716	-0,409721	0,46325	0,704415	no
XLOC_000484	g1181	86,699	56,0702	-0,628781	-0,863653	0,10875	0,320795	no
XLOC_000485	g1182	42,2464	40,9192	-0,0460518	-0,0683257	0,9024	0,957458	no
XLOC_000486	g1183	49,5897	38,1546	-0,378181	-0,608128	0,2825	0,54502	no
XLOC_000487	g1185	59,739	49,0439	-0,2846	-0,461948	0,4014	0,655211	no
XLOC_000488	g1188	18,6402	14,344	-0,37797	-0,544191	0,3332	0,594049	no
XLOC_000489	g1189	811,779	657,095	-0,304986	-0,464279	0,4097	0,662373	no
XLOC_000490	g1190	2212,63	2426,92	0,133366	0,216575	0,70815	0,861576	no
XLOC_000491	g1194	242,752	300,516	0,30796	0,443337	0,4272	0,67663	no
XLOC_000492	g1180	109,133	73,5283	-0,569719	-0,938773	0,09915	0,302002	no
XLOC_000493	g1184	65,3206	112,672	0,786522	1,35041	0,0181	0,10099	no
XLOC_000494	g1186	2,15234	1,66537	-0,370061	-0,357412	0,51165	0,737798	no
XLOC_000495	g1187	19,4563	15,0744	-0,368137	-0,514193	0,36915	0,628841	no
XLOC_000496	g1191	6,91492	9,45041	0,450663	0,778164	0,17395	0,419909	no
XLOC_000497	g1192	103,402	80,5757	-0,359854	-0,539349	0,35235	0,612945	no
XLOC_000498	g1193	165,495	212,745	0,362343	0,565478	0,30245	0,564114	no
XLOC_000499	g5714	446,961	417,153	-0,0995727	-0,133051	0,8166	0,916563	no
XLOC_000500	g5715	56,5456	28,1078	-1,00844	-1,43185	0,01355	0,0826979	no
XLOC_000501	g11649	11,4408	14,9418	0,385176	0,373632	0,5071	0,734472	no
XLOC_000502	g1195	57,9615	64,4536	0,153165	0,233866	0,6805	0,846316	no
XLOC_000503	g5716	58,8373	36,6399	-0,683318	-1,05213	0,0736	0,250691	no
XLOC_000504	g5718	29,4936	24,1597	-0,287796	-0,493223	0,3803	0,637186	no
XLOC_000505	g5720	46,8455	53,663	0,196015	0,331423	0,55525	0,768398	no
XLOC_000506	g5717	15,0046	50,7624	1,75835	2,63479	5,00E-05	0,00120049	yes
XLOC_000507	g5719	45,3262	39,7339	-0,189974	-0,302335	0,59805	0,794918	no
XLOC_000508	g5721	9,92203	32,4885	1,71122	2,54467	5,00E-05	0,00120049	yes
XLOC_000509	g5723	139,627	90,3019	-0,628745	-1,07117	0,05735	0,21438	no
XLOC_000510	g5725	2025,04	2491,6	0,299119	0,511861	0,3847	0,64084	no
XLOC_000511	g5722	1668,06	1509,28	-0,144311	-0,170687	0,7634	0,888537	no
XLOC_000512	g5724	12,6793	20,6886	0,706358	1,11367	0,05105	0,19917	no
XLOC_000513	g5726	33,394	33,803	0,0175642	0,0274157	0,96075	0,982256	no
XLOC_000514	g5727	0	0	0	0	1	1	no
XLOC_000515	g5728	33,7893	44,2745	0,389909	0,577241	0,313	0,573658	no
XLOC_000516	g5729	11,7376	4,84696	-1,27598	-1,35118	0,0243	0,12409	no
XLOC_000517	g1197	293,72	272,611	-0,107597	-0,157533	0,7818	0,898873	no
XLOC_000518	g1199	80,1491	74,4725	-0,105979	-0,176201	0,7608	0,887299	no
XLOC_000519	g1203	2,19977	1,02957	-1,09531	-0,97557	0,10465	0,312835	no
XLOC_000520	g1205	6,10437	4,78696	-0,350733	-0,43091	0,4521	0,696739	no
XLOC_000521	g1206	19,3081	9,43257	-1,03348	-1,71379	0,0037	0,032971	yes
XLOC_000522	g1209	1,60502	1,17952	-0,444387	-0,3604	0,57255	0,779083	no
XLOC_000523	g1196	157,27	195,317	0,312577	0,496985	0,38055	0,637496	no
XLOC_000524	g1198	542,179	234,872	-1,20689	-2,01352	0,0002	0,00367103	yes
XLOC_000525	g1200	103,765	58,0575	-0,837768	-1,0939	0,0496	0,19569	no
XLOC_000526	g1201	216,456	251,127	0,214345	0,322304	0,5742	0,780003	no
XLOC_000527	g1202	16,4312	29,1664	0,827864	1,15821	0,04495	0,18293	no
XLOC_000528	g1204	24,5364	16,0182	-0,615207	-0,924835	0,10995	0,323169	no
XLOC_000529	g1207	1,04609	5,11129	2,28869	2,29112	0,00095	0,0120686	yes
XLOC_000530	g1208	3,8679	0,78886	-2,29371	-2,25774	0,00115	0,0138564	yes
XLOC_000531	g11650	4,9971	2,54689	-0,972357	-1,09931	0,0586	0,216824	no
XLOC_000532	g11651	339,94	267,437	-0,346083	-0,55509	0,34095	0,601208	no
XLOC_000533	g5730	0	0	0	0	1	1	no
XLOC_000534	g5731	47,798	68,6442	0,522188	0,879845	0,1268	0,351348	no
XLOC_000535	g5732	31,997	31,3942	-0,0274385	-0,0455259	0,93735	0,972153	no
XLOC_000536	g1210	15,9674	12,1074	-0,399236	-0,605486	0,2842	0,546579	no
XLOC_000537	g1212	26,213	31,9588	0,285927	0,362104	0,5326	0,752862	no
XLOC_000538	g1215	31,1743	35,7624	0,198085	0,338373	0,55	0,765215	no
XLOC_000539	g1217	61,3212	63,9136	0,0597371	0,103409	0,86145	0,935986	no
XLOC_000540	g1219	250,92	412,745	0,718022	0,958954	0,08775	0,279956	no
XLOC_000541	g1222	16,4906	23,2312	0,494425	0,721752	0,21825	0,475214	no
XLOC_000542	g1224	89,3231	96,1544	0,106319	0,172087	0,75985	0,886717	no
XLOC_000543	g1228	971,699	2349,3	1,27365	2,03424	0,0003	0,00496796	yes
XLOC_000544	g1232	26,5368	49,6559	0,903969	1,49215	0,00805	0,0571591	no
XLOC_000545	g1234	147,542	140,444	-0,0711342	-0,121623	0,82845	0,921598	no
XLOC_000546	g1236	200,533	127,152	-0,657283	-1,09035	0,0551	0,208993	no
XLOC_000547	g1211	15,5956	15,6737	0,00720914	0,0120532	0,983	0,991787	no
XLOC_000548	g1213	89,1247	97,4436	0,128741	0,215585	0,7053	0,860058	no
XLOC_000549	g1214	47,2873	60,1061	0,346058	0,583792	0,30835	0,569386	no
XLOC_000550	g1216	198,048	194,833	-0,0236078	-0,0390081	0,94555	0,975552	no
XLOC_000551	g1218	55,2651	79,254	0,520114	0,843954	0,1353	0,365817	no

XLOC_000552	g1220	409,887	413,684	0,0133037	0,017888	0,97495	0,987724	no
XLOC_000553	g1221	18,2208	16,9715	-0,102473	-0,149104	0,79315	0,904567	no
XLOC_000554	g1223	65,1814	63,1491	-0,0456995	-0,0783654	0,88945	0,950735	no
XLOC_000555	g1225	103,805	229,643	1,14552	1,89161	0,00125	0,0146655	yes
XLOC_000556	g1226	40,0249	21,323	-0,908489	-1,48629	0,009	0,0618908	no
XLOC_000557	g1227	119,257	145,229	0,284259	0,408444	0,4547	0,699319	no
XLOC_000558	g1229	113,7	137,241	0,271483	0,446716	0,4202	0,671172	no
XLOC_000559	g1230	247,576	689,177	1,477	2,3957	5,00E-05	0,00120049	yes
XLOC_000560	g1231	94,5362	119,659	0,339989	0,547122	0,3231	0,583565	no
XLOC_000561	g1233	52,4113	57,5449	0,13481	0,209875	0,7124	0,863888	no
XLOC_000562	g1235	56,854	52,2268	-0,122472	-0,206064	0,71765	0,865764	no
XLOC_000563	g1237	14,6723	9,35203	-0,649744	-0,847054	0,1337	0,363462	no
XLOC_000564	g5733	14,797	35,9819	1,28197	1,59782	0,00505	0,0412571	yes
XLOC_000565	g5736	15,686	13,569	-0,209165	-0,33458	0,56815	0,776676	no
XLOC_000566	g5738	26,5043	2,46563	-3,4262	-3,79882	5,00E-05	0,00120049	yes
XLOC_000567	g5739	35,7518	28,46	-0,329082	-0,547257	0,3276	0,588769	no
XLOC_000568	g5740	19,7485	16,8202	-0,231545	-0,380484	0,5015	0,730455	no
XLOC_000569	g5743	13,5678	17,061	0,330518	0,471287	0,39315	0,647991	no
XLOC_000570	g5734	40,2386	42,9662	0,0946242	0,156721	0,78715	0,901646	no
XLOC_000571	g5735	10,9945	8,98095	-0,291846	-0,471934	0,39135	0,646031	no
XLOC_000572	g5737	5,86888	3,57825	-0,713832	-0,837546	0,154	0,39191	no
XLOC_000573	g5741	62,2933	55,4092	-0,168951	-0,264046	0,64645	0,825677	no
XLOC_000574	g5742	30,2483	21,23	-0,510754	-0,730723	0,19935	0,451802	no
XLOC_000575	g11652	0	0	0	0	1	1	no
XLOC_000576	g5744	23,9559	12,2842	-0,963576	-1,36642	0,02125	0,113906	no
XLOC_000577	g5745	29,4538	29,8022	0,0169625	0,0238515	0,96805	0,985921	no
XLOC_000578	g5746	31,4769	23,5531	-0,418373	-0,636164	0,26595	0,526751	no
XLOC_000579	g5748	1197,51	320,574	-1,90131	-2,15104	0,0025	0,0247231	yes
XLOC_000580	g5747	136,531	94,2561	-0,534569	-0,798396	0,1601	0,401272	no
XLOC_000581	g11653	187,442	233,801	0,318836	0,50842	0,36475	0,624341	no
XLOC_000582	g5749	37,8921	70,5071	0,895872	1,47814	0,0098	0,0655112	no
XLOC_000583	g5751	48,0358	37,3543	-0,362835	-0,586426	0,2947	0,556907	no
XLOC_000584	g5750	58,0481	40,0011	-0,537209	-0,901825	0,1143	0,331588	no
XLOC_000585	g5752	4,01284	13,6554	1,76678	1,9232	0,0015	0,0168343	yes
XLOC_000586	g5754	7,71507	15,939	1,04681	1,37545	0,01775	0,0996029	no
XLOC_000587	g5756	56,7163	91,8195	0,695037	1,1541	0,04395	0,180506	no
XLOC_000588	g5753	3,94548	7,46456	0,919857	1,3047	0,02455	0,124848	no
XLOC_000589	g5755	626,904	1099,02	0,809899	1,05053	0,06625	0,234188	no
XLOC_000590	g5757	59,4937	51,6316	-0,204483	-0,348287	0,53355	0,753337	no
XLOC_000591	g11654	0	0	0	0	1	1	no
XLOC_000592	g11661	89,4978	112,69	0,332438	0,571494	0,32115	0,581112	no
XLOC_000593	g11655	12,9281	8,38221	-0,625105	-0,936744	0,09625	0,296758	no
XLOC_000594	g11656	48,5537	40,3032	-0,268688	-0,429396	0,45965	0,701734	no
XLOC_000595	g11657	61,1101	151,401	1,30889	2,16959	0,00035	0,00552572	yes
XLOC_000596	g11658	35,6604	28,0841	-0,344566	-0,444257	0,44235	0,688832	no
XLOC_000597	g11659	3,97799	1,3899	-1,51706	-1,77741	0,0039	0,0342252	yes
XLOC_000598	g11660	517,7	292,809	-0,822156	-1,40764	0,01695	0,0963804	no
XLOC_000599	g11662	54,6791	55,3504	0,0176056	0,0279118	0,96225	0,983533	no
XLOC_000600	g11663	32,2588	11,0042	-1,55163	-2,08545	0,00055	0,00788467	yes
XLOC_000601	g5758	2,83768	7,80914	1,46045	1,77325	0,0032	0,0297576	yes
XLOC_000602	g5760	0	0	0	0	1	1	no
XLOC_000603	g5761	0	0	0	0	1	1	no
XLOC_000604	g5759	0	0,123997	inf	0	1	1	no
XLOC_000605	g11664	115,989	82,7575	-0,487025	-0,728095	0,2216	0,478582	no
XLOC_000606	g11665	212,467	236,949	0,157337	0,268287	0,63175	0,816117	no
XLOC_000607	g11666	17,0231	1,35679	-3,64923	-4,30861	5,00E-05	0,00120049	yes
XLOC_000608	g11667	44,7841	39,733	-0,172648	-0,265977	0,6429	0,82322	no
XLOC_000609	g1238	47,4425	95,8856	1,01513	1,57867	0,0053	0,0425908	yes
XLOC_000610	g1240	103,273	55,7301	-0,88993	-1,49986	0,00795	0,056695	no
XLOC_000611	g1241	1241,1	994,401	-0,319718	-0,517074	0,35635	0,61704	no
XLOC_000612	g1243	42,362	39,7201	-0,0929016	-0,151467	0,78625	0,901303	no
XLOC_000613	g1244	115,544	111,518	-0,0511634	-0,0766899	0,8911	0,951359	no
XLOC_000614	g1246	65,1918	88,066	0,433895	0,56171	0,3191	0,579522	no
XLOC_000615	g1247	78,3772	64,9674	-0,270719	-0,435878	0,4518	0,696605	no
XLOC_000616	g1239	73,394	40,6767	-0,851458	-1,46637	0,01085	0,070421	no
XLOC_000617	g1242	12,2656	25,6011	1,06159	1,66778	0,0023	0,0233326	yes
XLOC_000618	g1245	157,125	155,452	-0,0154388	-0,0260432	0,9632	0,983839	no
XLOC_000619	g5762	803,342	2407,57	1,58349	2,02738	0,00255	0,0250912	yes
XLOC_000620	g5763	0	0	0	0	1	1	no
XLOC_000621	g5764	69,0785	237,381	1,7809	2,00873	0,0003	0,00496796	yes
XLOC_000622	g5765	232,351	277,764	0,257557	0,338939	0,52885	0,74972	no
XLOC_000623	g11668	71,3947	65,8816	-0,115942	-0,179612	0,74435	0,879499	no
XLOC_000624	g11669	29,6106	66,8464	1,17474	1,71476	0,00265	0,0257909	yes
XLOC_000625	g11670	260,81	182,276	-0,516871	-0,829213	0,1554	0,392876	no
XLOC_000626	g1248	1,2748	3,41336	1,42092	1,1411	0,07095	0,244982	no
XLOC_000627	g5767	12,2518	12,5818	0,0383437	0,0618032	0,90595	0,958977	no
XLOC_000628	g5768	43,1876	71,982	0,737019	1,24202	0,03095	0,144179	no
XLOC_000629	g5766	26,3124	37,8738	0,525456	0,72943	0,2297	0,487604	no
XLOC_000630	g5769	2,51482	0	#NAME?	0	1	1	no
XLOC_000631	g5770	2,14031	2,0642	-0,0522321	0	1	1	no
XLOC_000632	g11671	81,6366	46,3751	-0,815866	-1,30891	0,0233	0,121254	no
XLOC_000633	g11672	58,202	50,4512	-0,206182	-0,339744	0,5449	0,761892	no
XLOC_000634	g11673	254,408	541,664	1,09025	1,25235	0,0208	0,112044	no
XLOC_000635	g11674	70,3869	90,9457	0,369699	0,600059	0,2934	0,555785	no
XLOC_000636	g11675	203,065	306,972	0,596164	0,760392	0,20305	0,45576	no
XLOC_000637	g11676	18,018	19,6071	0,121939	0,163719	0,77905	0,896924	no
XLOC_000638	g11677	37,5374	35,401	-0,0845364	-0,112401	0,84855	0,931347	no
XLOC_000639	g5772	737,325	892,552	0,275636	0,430209	0,4521	0,696739	no
XLOC_000640	g5774	90,6746	119,086	0,39323	0,631178	0,25265	0,511863	no
XLOC_000641	g5771	47,4855	55,79	0,23252	0,397713	0,47595	0,712474	no
XLOC_000642	g5773	61,2818	43,1319	-0,506703	-0,841905	0,1338	0,363462	no
XLOC_000643	g11678	13,7649	9,86482	-0,480634	-0,508779	0,3708	0,629539	no

XLOC_000644	g5775	0	0	0	0	1	1	no
XLOC_000645	g5776	28,4091	49,9387	0,813806	1,26978	0,0252	0,127231	no
XLOC_000646	g11679	326,721	168,566	-0,954748	-1,51147	0,00665	0,0501945	no
XLOC_000647	g5777	101,298	173,207	0,773884	1,01598	0,0832	0,271617	no
XLOC_000648	g5779	82,3411	123,629	0,586336	0,984902	0,09485	0,294197	no
XLOC_000649	g5781	95,3944	100,226	0,0712851	0,122207	0,82465	0,920234	no
XLOC_000650	g5783	119,633	157,795	0,39944	0,677385	0,22245	0,47989	no
XLOC_000651	g5785	124,783	139,863	0,164597	0,289763	0,62025	0,808752	no
XLOC_000652	g5778	80,2093	109,614	0,450596	0,703958	0,2203	0,477349	no
XLOC_000653	g5780	88,7034	126,157	0,508155	0,858277	0,1182	0,337616	no
XLOC_000654	g5782	1757,55	2036,37	0,21244	0,333257	0,54885	0,765215	no
XLOC_000655	g5784	43,0321	52,3656	0,283205	0,452783	0,4202	0,671172	no
XLOC_000656	g1249	22,2415	47,4948	1,09451	1,6492	0,00505	0,0412571	yes
XLOC_000657	g5787	98,487	95,3771	-0,0462906	-0,078645	0,88955	0,950738	no
XLOC_000658	g5790	62,7256	53,8708	-0,219551	-0,351706	0,5521	0,766299	no
XLOC_000659	g5786	23,2503	17,3702	-0,420637	-0,661661	0,23005	0,487612	no
XLOC_000660	g5788	20,7441	30,0228	0,533356	0,790392	0,16215	0,403833	no
XLOC_000661	g5789	218,836	74,8737	-1,54732	-2,55832	5,00E-05	0,00120049	yes
XLOC_000662	g5791	359,176	515,332	0,520813	0,628787	0,24525	0,50394	no
XLOC_000663	g5792	21,0924	13,2966	-0,665665	-0,826672	0,24145	0,499688	no
XLOC_000664	g5795	4,82154	3,86333	-0,31965	-0,398008	0,46475	0,705296	no
XLOC_000665	g5793	20,7267	26,6856	0,364572	0,517742	0,35655	0,617124	no
XLOC_000666	g5794	8,77217	9,26751	0,0792492	0,128856	0,82365	0,919432	no
XLOC_000667	g5796	115,606	126,788	0,133198	0,223424	0,6969	0,856194	no
XLOC_000668	g5797	65,2838	66,8878	0,0350185	0,0572973	0,91615	0,963749	no
XLOC_000669	g5798	90,2939	122,509	0,440182	0,757597	0,1881	0,43709	no
XLOC_000670	g5801	157,176	117,329	-0,421821	-0,668126	0,2492	0,508092	no
XLOC_000671	g5799	40,5723	48,027	0,24335	0,316513	0,5768	0,780727	no
XLOC_000672	g5800	48,4358	45,8126	-0,0803297	-0,138677	0,80495	0,91004	no
XLOC_000673	g5802	0	0	0	0	1	1	no
XLOC_000674	g5804	0,465689	0	#NAME?	0	1	1	no
XLOC_000675	g5803	1,5882	6,24817	1,97604	2,27637	0,0002	0,00367103	yes
XLOC_000676	g11680	60,0469	62,7912	0,0644731	0,113757	0,8439	0,929151	no
XLOC_000677	g11681	5,3135	11,6946	1,13811	4,38862	0,4234	0,67289	no
XLOC_000678	g5805	0	0	0	0	1	1	no
XLOC_000679	g5806	43,3439	58,7451	0,438641	0,740949	0,2004	0,45251	no
XLOC_000680	g5808	189,034	145,153	-0,381066	-0,627341	0,2822	0,544655	no
XLOC_000681	g5809	26,6668	23,2425	-0,198281	-0,303991	0,5911	0,790276	no
XLOC_000682	g5811	25,8908	28,4435	0,135662	0,22033	0,7043	0,859387	no
XLOC_000683	g5807	80,3589	45,6659	-0,815342	-1,16288	0,04235	0,176743	no
XLOC_000684	g5810	26,1921	12,0359	-1,12179	-1,7228	0,0023	0,0233326	yes
XLOC_000685	g1250	1283,19	1713,07	0,416844	0,623582	0,2839	0,546579	no
XLOC_000686	g1253	37,2192	44,6615	0,262986	0,409859	0,4872	0,720794	no
XLOC_000687	g1254	38,4107	46,1526	0,264901	0,452813	0,41965	0,671057	no
XLOC_000688	g1255	1,50125	5,49802	1,87275	2,1037	0,00105	0,0130684	yes
XLOC_000689	g1257	35,8364	43,8636	0,291602	0,491335	0,3843	0,640475	no
XLOC_000690	g1259	61,9948	24,9359	-1,31392	-2,10052	0,00025	0,00430702	yes
XLOC_000691	g1260	62,1532	56,0955	-0,147942	-0,251753	0,65035	0,828117	no
XLOC_000692	g1261	52,2983	52,9127	0,0168506	0,0279583	0,9605	0,982256	no
XLOC_000693	g1251	463,164	392,892	-0,237393	-0,31397	0,5853	0,786272	no
XLOC_000694	g1252	77,0817	81,5654	0,0815685	0,142695	0,80595	0,910751	no
XLOC_000695	g1256	24,1801	15,1018	-0,679099	-1,09953	0,0508	0,198827	no
XLOC_000696	g1258	28,1921	25,1891	-0,162492	-0,272257	0,6294	0,814495	no
XLOC_000697	g1262	36,6844	29,8094	-0,299398	-0,488676	0,38065	0,637555	no
XLOC_000698	g11682	8,09553	14,7034	0,860956	1,19905	0,0306	0,143091	no
XLOC_000699	g11683	6,71126	5,30054	-0,340446	-0,321282	0,5703	0,777826	no
XLOC_000700	g11684	0	0	0	0	1	1	no
XLOC_000701	g5812	43,2903	21,7532	-0,992818	-1,58771	0,00725	0,0533296	no
XLOC_000702	g5813	20,1391	18,216	-0,144794	-0,209217	0,71045	0,862876	no
XLOC_000703	g5814	0	0	0	0	1	1	no
XLOC_000704	g11685	1,74163	3,87362	1,15325	1,25885	0,0343	0,153872	no
XLOC_000705	g5815	27,1453	23,4344	-0,212073	-0,307065	0,5877	0,78831	no
XLOC_000706	g5816	0	0	0	0	1	1	no
XLOC_000707	g1263	7,44371	1,15848	-2,68378	-2,43707	0,0198	0,10814	no
XLOC_000708	g1264	10,0718	18,5302	0,879565	1,17578	0,03115	0,144699	no
XLOC_000709	g11686	1960	2711,64	0,468313	0,642981	0,2564	0,515952	no
XLOC_000710	g11687	0,208006	0,561849	1,43356	0	1	1	no
XLOC_000711	g11688	0,498538	0,0907583	-2,4576	0	1	1	no
XLOC_000712	g11689	0	0	0	0	1	1	no
XLOC_000713	g5817	27,1362	11,9832	-1,1792	-1,63537	0,0067	0,0503783	no
XLOC_000714	g5818	8,07501	1,9179	-2,07394	-2,17626	0,00585	0,0457017	yes
XLOC_000715	g5819	10,6872	12,0628	0,174674	0,230894	0,6882	0,850292	no
XLOC_000716	g5820	0	0	0	0	1	1	no
XLOC_000717	g5821	90,8376	110,721	0,285562	0,508603	0,3897	0,644686	no
XLOC_000718	g5822	43,9502	55,7944	0,34425	0,544835	0,32895	0,590276	no
XLOC_000719	g5823	0	0	0	0	1	1	no
XLOC_000720	g11690	0	0	0	0	1	1	no
XLOC_000721	g5824	3,17583	7,47989	1,23589	1,35867	0,02735	0,13382	no
XLOC_000722	g5825	116,752	114,066	-0,0335749	-0,0566869	0,91815	0,964054	no
XLOC_000723	g5826	22,9069	9,87886	-1,21337	-1,28323	0,027	0,133036	no
XLOC_000724	g5828	23,9466	9,20342	-1,37958	-1,91839	0,00155	0,0171215	yes
XLOC_000725	g5827	6,08972	6,57234	0,11003	0,116937	0,8384	0,926315	no
XLOC_000726	g5829	3,33641	3,6119	0,114461	0,142556	0,8069	0,910938	no
XLOC_000727	g11691	29,9759	22,6708	-0,402966	-0,583019	0,29225	0,554783	no
XLOC_000728	g11693	40,1873	23,1007	-0,798807	-1,35878	0,0163	0,0934965	no
XLOC_000729	g11692	89,2542	54,3241	-0,716329	-1,16396	0,04285	0,177622	no
XLOC_000730	g11694	56,9925	60,6578	0,0899209	0,1499	0,7948	0,905607	no
XLOC_000731	g11695	167,908	304,489	0,858725	1,26472	0,03075	0,143519	no
XLOC_000732	g11696	28,3671	26,4829	-0,0991608	-0,154092	0,78575	0,901303	no
XLOC_000733	g11697	0	0	0	0	1	1	no
XLOC_000734	g11698	127,734	143,809	0,171003	0,178658	0,75595	0,884596	no
XLOC_000735	g5830	92,1062	538,774	2,54831	3,5205	5,00E-05	0,00120049	yes

XLOC_000736	g5831	35,905	23,8789	-0,588446	-0,92157	0,11185	0,3267	no
XLOC_000737	g5832	4,54352	3,96037	-0,198173	-0,212653	0,7066	0,860848	no
XLOC_000738	g11699	0,214267	1,0947	2,35304	0	1	1	no
XLOC_000739	g11700	373,506	2141,9	2,51969	2,60751	5,00E-05	0,00120049	yes
XLOC_000740	g5833	0	0	0	0	1	1	no
XLOC_000741	g5834	46,6483	32,6959	-0,512712	-0,856459	0,1261	0,350199	no
XLOC_000742	g5835	1,6223	1,9606	0,273256	0	1	1	no
XLOC_000743	g5836	128,837	86,9783	-0,566814	-0,886329	0,11885	0,338586	no
XLOC_000744	g5838	35,5844	25,388	-0,487101	-0,779914	0,17635	0,423102	no
XLOC_000745	g5837	194,825	233,597	0,261849	0,333003	0,56885	0,777035	no
XLOC_000746	g11701	0	0	0	0	1	1	no
XLOC_000747	g11702	697,814	629,481	-0,148681	-0,228692	0,68105	0,846464	no
XLOC_000748	g11703	26,4208	23,0054	-0,199701	-0,239783	0,67655	0,843326	no
XLOC_000749	g5839	11,2084	13,4741	0,265611	0,436557	0,44305	0,689173	no
XLOC_000750	g11704	0	0,0967255	inf	0	1	1	no
XLOC_000751	g11705	736,506	600,178	-0,295307	-0,356321	0,5489	0,765215	no
XLOC_000752	g11706	79,9097	53,6682	-0,574303	-0,563417	0,3345	0,594729	no
XLOC_000753	g11707	0	0	0	0	1	1	no
XLOC_000754	g11708	0	0	0	0	1	1	no
XLOC_000755	g5840	41,9548	37,3637	-0,167201	-0,219863	0,6934	0,854031	no
XLOC_000756	g5841	60,1135	50,3737	-0,25502	-0,324702	0,57595	0,780604	no
XLOC_000757	g5844	10,7433	17,6682	0,717716	0,968584	0,0869	0,27849	no
XLOC_000758	g5842	69,72	66,9486	-0,0585178	-0,100378	0,8611	0,935813	no
XLOC_000759	g5843	89,9329	86,6438	-0,053753	-0,0910413	0,87405	0,94326	no
XLOC_000760	g5845	63,3324	35,9216	-0,818095	-1,25102	0,03235	0,148309	no
XLOC_000761	g5846	6,86376	3,65901	-0,907546	-1,18152	0,0395	0,168648	no
XLOC_000762	g5847	3,81911	1,87154	-1,02901	-1,42575	0,01645	0,0942468	no
XLOC_000763	g5848	0	0	0	0	1	1	no
XLOC_000764	g1265	0	0	0	0	1	1	no
XLOC_000765	g11709	11,1394	9,56865	-0,219286	-0,289613	0,6176	0,808291	no
XLOC_000766	g11710	10,2773	16,2598	0,66185	0,856259	0,14965	0,387135	no
XLOC_000767	g11711	32,8869	23,2643	-0,499391	-0,813437	0,1454	0,38167	no
XLOC_000768	g11712	103,34	59,1099	-0,805933	-1,02234	0,08495	0,275226	no
XLOC_000769	g11713	0	0	0	0	1	1	no
XLOC_000770	g1267	31,1602	49,5957	0,670511	1,13323	0,04645	0,186942	no
XLOC_000771	g1268	66,0692	125,913	0,930383	1,50239	0,00875	0,0605959	no
XLOC_000772	g1269	17,1484	5,2108	-1,7185	-1,70682	0,0072	0,0530413	no
XLOC_000773	g1271	129,101	270,155	1,06528	1,6775	0,0033	0,0304282	yes
XLOC_000774	g1266	114,496	35,6951	-1,6815	-1,97769	0,0003	0,00496796	yes
XLOC_000775	g1270	142,092	141,2	-0,00909059	-0,0146092	0,97845	0,989941	no
XLOC_000776	g1272	150,323	89,9566	-0,740765	-0,916232	0,1076	0,319224	no
XLOC_000777	g11714	33,4051	58,7495	0,81451	0,975584	0,10495	0,313541	no
XLOC_000778	g11715	70,0554	102,198	0,544803	0,918412	0,11935	0,339518	no
XLOC_000779	g11716	44,6599	24,3447	-0,875373	-1,44778	0,01345	0,082241	no
XLOC_000780	g11717	740,477	132,228	-2,48543	-3,74352	5,00E-05	0,00120049	yes
XLOC_000781	g5849	0	0,303728	inf	0	1	1	no
XLOC_000782	g5850	14,8021	12,5365	-0,239661	-0,372843	0,50415	0,732252	no
XLOC_000783	g11718	0	0	0	0	1	1	no
XLOC_000784	g5851	29,5194	29,8308	0,0151377	0,0244073	0,96335	0,983889	no
XLOC_000785	g11719	167,293	36,3864	-2,2009	-3,39988	5,00E-05	0,00120049	yes
XLOC_000786	g5855	10,8583	12,9925	0,258889	0,364817	0,5196	0,743978	no
XLOC_000787	g5852	32,1332	68,9113	1,10068	1,58952	0,0067	0,0503783	no
XLOC_000788	g5853	103,285	11,1928	-3,20599	-4,25689	5,00E-05	0,00120049	yes
XLOC_000789	g5854	633,977	1093,35	0,786255	1,32211	0,02155	0,115074	no
XLOC_000790	g5856	0	0	0	0	1	1	no
XLOC_000791	g5857	53,144	46,6278	-0,188718	-0,207653	0,73295	0,873916	no
XLOC_000792	g5858	0	0	0	0	1	1	no
XLOC_000793	g11721	84,7432	168,131	0,988415	1,66896	0,0034	0,0310297	yes
XLOC_000794	g11722	105,59	305,066	1,53065	2,09925	0,0003	0,00496796	yes
XLOC_000795	g11724	47,725	39,0847	-0,288142	-0,486703	0,39085	0,646031	no
XLOC_000796	g11720	819,549	794,596	-0,0446102	-0,0730597	0,8946	0,95323	no
XLOC_000797	g11723	69,0922	84,1163	0,283863	0,489223	0,39135	0,646031	no
XLOC_000798	g11725	118,517	151,322	0,352523	0,610073	0,283	0,545663	no
XLOC_000799	g11726	31,8306	19,0688	-0,739197	-1,07707	0,06335	0,227874	no
XLOC_000800	g11727	0	0	0	0	1	1	no
XLOC_000801	g11728	0	0	0	0	1	1	no
XLOC_000802	g5859	17,0682	11,3096	-0,593762	-0,732576	0,20365	0,456689	no
XLOC_000803	g5860	827,933	1085,05	0,39018	0,599992	0,28835	0,551849	no
XLOC_000804	g5861	51,4452	152,13	1,5642	2,21116	0,00015	0,00294012	yes
XLOC_000805	g11729	99,0581	274,628	1,47113	2,46289	5,00E-05	0,00120049	yes
XLOC_000806	g11730	29,9365	26,3316	-0,18511	-0,270938	0,64415	0,824348	no
XLOC_000807	g5864	73,7538	99,3944	0,430448	0,717254	0,2104	0,465449	no
XLOC_000808	g5866	118,189	164,519	0,477158	0,768557	0,16725	0,410619	no
XLOC_000809	g5867	45,7942	53,0422	0,211975	0,363224	0,5223	0,745347	no
XLOC_000810	g5869	66,8008	77,0251	0,205463	0,343721	0,54935	0,765215	no
XLOC_000811	g5872	177,157	170,705	-0,0535235	-0,0908662	0,8748	0,9436	no
XLOC_000812	g5874	107,432	171,975	0,678784	1,07506	0,0619	0,224219	no
XLOC_000813	g5876	121,389	207,692	0,7748	1,2613	0,02865	0,137614	no
XLOC_000814	g5877	42,8842	39,6542	-0,112972	-0,171452	0,76575	0,890392	no
XLOC_000815	g5879	44,1165	104,87	1,24921	2,11222	0,00025	0,00430702	yes
XLOC_000816	g5881	184,114	173,583	-0,0849731	-0,136401	0,80695	0,910938	no
XLOC_000817	g5883	46,1758	49,3547	0,0960512	0,160912	0,7731	0,89421	no
XLOC_000818	g5885	181,765	159,965	-0,184311	-0,317929	0,5765	0,780604	no
XLOC_000819	g5886	42,5226	23,6744	-0,844899	-1,45358	0,01135	0,0727052	no
XLOC_000820	g5888	32,2362	32,3023	0,0029557	0,00471515	0,9935	0,996239	no
XLOC_000821	g5890	36,7494	32,5628	-0,174495	-0,295382	0,6007	0,797037	no
XLOC_000822	g5862	484,083	412,154	-0,232071	-0,391981	0,49135	0,723327	no
XLOC_000823	g5863	16,1531	22,0642	0,449902	0,711434	0,20775	0,462294	no
XLOC_000824	g5865	35,185	62,6789	0,833019	1,42336	0,01325	0,0814746	no
XLOC_000825	g5868	20,068	16,5571	-0,277445	-0,469062	0,39875	0,65273	no
XLOC_000826	g5870	29,7038	32,8624	0,145789	0,240085	0,6815	0,846595	no
XLOC_000827	g5871	66,5042	49,9604	-0,41266	-0,696198	0,2254	0,483492	no

XLOC_000828	g5873	39,092	35,534	-0,13767	-0,231823	0,68345	0,847837	no
XLOC_000829	g5875	116,196	142,574	0,295143	0,478342	0,40295	0,656594	no
XLOC_000830	g5878	1610,79	2203,41	0,451972	0,70437	0,2228	0,480118	no
XLOC_000831	g5880	317,777	247,865	-0,358464	-0,501178	0,3812	0,637823	no
XLOC_000832	g5882	23,3625	24,1256	0,0463727	0,074538	0,89785	0,954518	no
XLOC_000833	g5884	126,559	144,058	0,186839	0,309148	0,5954	0,792683	no
XLOC_000834	g5887	157,187	170,989	0,121427	0,202854	0,7153	0,865069	no
XLOC_000835	g5889	207,238	137,315	-0,593805	-0,935423	0,09355	0,291546	no
XLOC_000836	g5891	311,488	349,595	0,166509	0,271054	0,62425	0,811876	no
XLOC_000837	g1273	44,3593	31,5808	-0,490189	-0,775794	0,17725	0,424122	no
XLOC_000838	g1274	49,4574	10,1058	-2,291	-3,24467	5,00E-05	0,00120049	yes
XLOC_000839	g1277	105,162	45,9085	-1,19579	-2,00691	0,00075	0,0100752	yes
XLOC_000840	g1279	4,35844	2,26994	-0,941156	-1,26191	0,02965	0,140863	no
XLOC_000841	g1275	86,9174	82,3232	-0,0783457	-0,128736	0,81835	0,917235	no
XLOC_000842	g1276	12,3081	8,65418	-0,50814	-0,712448	0,2203	0,477349	no
XLOC_000843	g1278	33,5108	43,8714	0,388657	0,638155	0,26185	0,521787	no
XLOC_000844	g5892	51,1716	53,6	0,0668898	0,107108	0,8542	0,934519	no
XLOC_000845	g5894	256,171	147,498	-0,79641	-1,27339	0,025	0,126481	no
XLOC_000846	g5893	22,6289	18,8751	-0,261682	-0,423117	0,4487	0,694314	no
XLOC_000847	g11731	0	0	0	0	1	1	no
XLOC_000848	g5897	1013,02	6640	2,71252	2,69719	5,00E-05	0,00120049	yes
XLOC_000849	g5898	14,1847	10,8932	-0,380907	-0,54803	0,3278	0,589002	no
XLOC_000850	g5895	4926,89	2650,64	-0,894339	-1,30072	0,0227	0,119142	no
XLOC_000851	g5896	49,1005	91,069	0,891224	1,43767	0,0157	0,0913894	no
XLOC_000852	g5899	32,254	17,336	-0,895712	-1,4948	0,0077	0,0555169	no
XLOC_000853	g5900	89,0009	76,8576	-0,211632	-0,294666	0,6191	0,808526	no
XLOC_000854	g5902	41,0859	45,4099	0,144364	0,210632	0,707	0,860848	no
XLOC_000855	g5901	1027,98	799,664	-0,362349	-0,599029	0,29455	0,556907	no
XLOC_000856	g1280	0,109836	0,448656	2,03026	0	1	1	no
XLOC_000857	g1281	31,8652	36,6025	0,199961	0,27117	0,63115	0,816117	no
XLOC_000858	g129	77,544	66,2479	-0,227139	-0,369021	0,51665	0,741989	no
XLOC_000859	g130	19,715	18,5	-0,091767	-0,144513	0,80225	0,908624	no
XLOC_000860	g133	179,547	370,015	1,04322	1,72038	0,0033	0,0304282	yes
XLOC_000861	g135	58,9957	39,9412	-0,56273	-0,946756	0,10265	0,308736	no
XLOC_000862	g136	36,1656	42,3101	0,226385	0,381532	0,5086	0,735019	no
XLOC_000863	g138	78,9238	88,9463	0,172474	0,296432	0,60305	0,798819	no
XLOC_000864	g140	19,781	15,505	-0,351379	-0,54885	0,3338	0,594729	no
XLOC_000865	g142	55,9714	24,8159	-1,17343	-1,94697	0,0009	0,0115984	yes
XLOC_000866	g128	19,1437	11,299	-0,760674	-1,05201	0,0718	0,247048	no
XLOC_000867	g131	22,2369	23,987	0,109298	0,171829	0,76645	0,890609	no
XLOC_000868	g132	348,077	937,136	1,42885	1,95598	0,0014	0,016042	yes
XLOC_000869	g134	90,4309	88,9783	-0,0233628	-0,0378409	0,94695	0,97628	no
XLOC_000870	g137	33,8437	27,8227	-0,282627	-0,478052	0,40215	0,65589	no
XLOC_000871	g139	21,1394	17,7985	-0,248181	-0,408218	0,4727	0,710282	no
XLOC_000872	g141	21,9512	10,328	-1,08774	-1,62637	0,00365	0,0326439	yes
XLOC_000873	g143	96,2099	98,1324	0,0285432	0,0417212	0,94105	0,973928	no
XLOC_000874	g5904	14,7791	63,753	2,10894	3,06473	5,00E-05	0,00120049	yes
XLOC_000875	g5906	27,0512	35,6001	0,396192	0,667299	0,2466	0,505345	no
XLOC_000876	g5907	64,732	12,4377	-2,37975	-3,47907	5,00E-05	0,00120049	yes
XLOC_000877	g5903	145,232	437,089	1,58957	2,5548	5,00E-05	0,00120049	yes
XLOC_000878	g5905	4,98604	5,15631	0,0484444	0,0478581	0,93025	0,969134	no
XLOC_000879	g5908	0	0	0	0	1	1	no
XLOC_000880	g5909	51,5165	81,4376	0,66066	1,04471	0,07455	0,252007	no
XLOC_000881	g11732	14,89	14,1396	-0,0746074	-0,0900226	0,8727	0,942536	no
XLOC_000882	g1283	54,1169	47,1104	-0,200035	-0,345011	0,54295	0,760812	no
XLOC_000883	g1284	83,5716	149,933	0,843229	1,45126	0,01235	0,0774438	no
XLOC_000884	g1285	10,485	9,10326	-0,203865	-0,275024	0,6363	0,818612	no
XLOC_000885	g1287	23,3052	28,0853	0,269165	0,458949	0,4121	0,66483	no
XLOC_000886	g1289	17,9948	26,4887	0,557796	0,799636	0,1444	0,380469	no
XLOC_000887	g1290	91,1565	103,946	0,189409	0,322394	0,56235	0,772769	no
XLOC_000888	g1282	72,3291	66,7396	-0,116034	-0,186724	0,74295	0,878585	no
XLOC_000889	g1286	15,6679	9,62881	-0,702386	-1,03679	0,07595	0,25481	no
XLOC_000890	g1288	88,0303	269,269	1,61298	1,95676	0,00225	0,0229201	yes
XLOC_000891	g1291	309,456	296,921	-0,0596574	-0,0977181	0,8592	0,935611	no
XLOC_000892	g5910	33,7623	43,6934	0,372004	0,539452	0,3432	0,603442	no
XLOC_000893	g11733	59,2851	45,4207	-0,384321	-0,659052	0,24455	0,503031	no
XLOC_000894	g11734	2,63023	1,40482	-0,904799	-1,15077	0,0455	0,18448	no
XLOC_000895	g11736	436,49	619,25	0,504572	0,808918	0,14855	0,385712	no
XLOC_000896	g11735	455,798	310,037	-0,555956	-0,831198	0,1426	0,377753	no
XLOC_000897	g11737	10,2576	8,00357	-0,357975	-0,422585	0,46455	0,705296	no
XLOC_000898	g11738	48,7842	60,7796	0,317173	0,546176	0,3389	0,598992	no
XLOC_000899	g11739	3,31985	6,63791	0,99961	1,0404	0,06885	0,240436	no
XLOC_000900	g11740	171,832	294,747	0,778477	0,841623	0,1478	0,384374	no
XLOC_000901	g11741	6,90337	7,24401	0,0694868	0,0935697	0,8662	0,938861	no
XLOC_000902	g5911	118,184	98,247	-0,266554	-0,458392	0,4282	0,677386	no
XLOC_000903	g5912	342,862	681,053	0,99014	1,59547	0,0067	0,0503783	no
XLOC_000904	g5913	0	0	0	0	1	1	no
XLOC_000905	g5914	209,316	67,5656	-1,63132	-2,16722	0,0468	0,187812	no
XLOC_000906	g5915	23,0782	18,0283	-0,35627	-0,522479	0,36285	0,622826	no
XLOC_000907	g5916	7,85338	10,7152	0,448277	0,693325	0,21965	0,477099	no
XLOC_000908	g5917	87,0173	57,1225	-0,607244	-1,03312	0,0741	0,251525	no
XLOC_000909	g5918	5,79493	2,13198	-1,44259	-1,52683	0,01435	0,0860825	no
XLOC_000910	g5919	0	0	0	0	1	1	no
XLOC_000911	g11742	0	0,0179402	inf	0	1	1	no
XLOC_000912	g5920	5,19872	4,91405	-0,0812449	-0,0972441	0,86735	0,939446	no
XLOC_000913	g5922	698,533	1222,13	0,806993	1,32962	0,0214	0,11446	no
XLOC_000914	g5921	63,5184	121,225	0,932446	1,58576	0,0051	0,0415963	yes
XLOC_000915	g5923	0	0	0	0	1	1	no
XLOC_000916	g144	15,3862	16,8956	0,135009	0,212418	0,7083	0,861576	no
XLOC_000917	g5925	64,1241	63,3078	-0,0184822	-0,0308325	0,95735	0,981231	no
XLOC_000918	g5924	12,3452	6,72044	-0,877327	-1,18532	0,03785	0,164245	no
XLOC_000919	g5926	151,151	68,6495	-1,13867	-1,88894	0,0013	0,0151795	yes

XLOC_000920	g5927	70,4964	58,8478	-0,260559	-0,42972	0,44935	0,694572	no
XLOC_000921	g5928	4,46284	0,523832	-3,09079	-2,17347	0,02315	0,120601	no
XLOC_000922	g5929	38,8736	29,9308	-0,377162	-0,617211	0,27815	0,539702	no
XLOC_000923	g1293	58,2463	102,664	0,817686	1,37811	0,0157	0,0913894	no
XLOC_000924	g1292	324,624	383,005	0,238593	0,405579	0,4828	0,716827	no
XLOC_000925	g5931	5,27241	4,54798	-0,213237	-0,217114	0,7113	0,863041	no
XLOC_000926	g5930	7,62973	8,39807	0,138426	0,183179	0,75315	0,883683	no
XLOC_000927	g5932	14,4648	25,2994	0,806559	1,20138	0,0354	0,157512	no
XLOC_000928	g5933	25,0199	29,3469	0,230131	0,388314	0,50125	0,730308	no
XLOC_000929	g5934	15,5525	9,77247	-0,67035	-0,930998	0,1045	0,312767	no
XLOC_000930	g5935	14,1685	5,71845	-1,309	-1,47036	0,0128	0,0797563	no
XLOC_000931	g5936	14,2543	22,155	0,636235	0,793396	0,17045	0,414312	no
XLOC_000932	g1294	27,9249	31,086	0,154714	0,237552	0,6867	0,849508	no
XLOC_000933	g1295	15,6735	18,7785	0,26076	0,42247	0,46935	0,707556	no
XLOC_000934	g1296	12,6805	23,4016	0,884001	1,19507	0,04085	0,172008	no
XLOC_000935	g5937	0	0	0	0	1	1	no
XLOC_000936	g5938	5,20912	9,78733	0,909877	1,23068	0,0357	0,158066	no
XLOC_000937	g1298	68,7034	29,3878	-1,22516	-2,00105	0,00095	0,0120686	yes
XLOC_000938	g1300	38,9688	33,9304	-0,199742	-0,299561	0,59455	0,792155	no
XLOC_000939	g1297	21,2071	15,0134	-0,49829	-0,733482	0,2051	0,458893	no
XLOC_000940	g1299	2,61292	6,28628	1,26654	1,46661	0,01505	0,0887634	no
XLOC_000941	g11743	0	0	0	0	1	1	no
XLOC_000942	g11744	0,0244619	0	#NAME?	0	1	1	no
XLOC_000943	g11745	0	0	0	0	1	1	no
XLOC_000944	g5939	4,02488	8,48264	1,07557	1,41896	0,0156	0,0909691	no
XLOC_000945	g5940	77,4918	170,633	1,13878	1,81773	0,00135	0,0156148	yes
XLOC_000946	g1301	17,3992	31,4045	0,851949	1,19411	0,03985	0,169626	no
XLOC_000947	g1303	31,8845	40,8453	0,357316	0,586674	0,3029	0,564534	no
XLOC_000948	g1302	67,3727	63,3209	-0,0894827	-0,153874	0,78455	0,900961	no
XLOC_000949	g5941	12,3893	3,37953	-1,8742	-2,37647	0,00015	0,00294012	yes
XLOC_000950	g11746	0	0	0	0	1	1	no
XLOC_000951	g11747	32,695	27,8352	-0,232158	-0,29606	0,7228	0,869308	no
XLOC_000952	g5942	0	0	0	0	1	1	no
XLOC_000953	g11749	79,9147	114,097	0,513733	0,826287	0,1376	0,369897	no
XLOC_000954	g11748	12,2317	11,4609	-0,0939023	-0,145407	0,79275	0,904567	no
XLOC_000955	g5943	4,80758	1,02284	-2,23272	-2,05131	0,00535	0,0426783	yes
XLOC_000956	g5944	35,637	42,6215	0,258204	0,430188	0,45665	0,701012	no
XLOC_000957	g5946	51,7263	69,9035	0,434465	0,614965	0,2766	0,538183	no
XLOC_000958	g5948	209,627	214,701	0,0345068	0,0589599	0,9166	0,963809	no
XLOC_000959	g5949	56,1474	309,624	2,46323	3,2836	5,00E-05	0,00120049	yes
XLOC_000960	g5950	78,2756	54,9386	-0,510743	-0,884671	0,12305	0,34663	no
XLOC_000961	g5952	59,6679	37,1068	-0,685272	-1,14979	0,0473	0,189046	no
XLOC_000962	g5953	90,972	122,424	0,428389	0,710752	0,2162	0,472111	no
XLOC_000963	g5955	377,041	823,039	1,12624	1,89513	0,0014	0,016042	yes
XLOC_000964	g5957	77,6964	63,5252	-0,29052	-0,497357	0,376	0,634261	no
XLOC_000965	g5945	86,1032	101,176	0,232735	0,367377	0,51705	0,741989	no
XLOC_000966	g5947	53,7588	61,2646	0,188552	0,307314	0,5952	0,792631	no
XLOC_000967	g5951	40,5291	40,0198	-0,0182437	-0,0305156	0,95895	0,981744	no
XLOC_000968	g5954	118,788	106,574	-0,156534	-0,257848	0,65565	0,831391	no
XLOC_000969	g5956	14,1039	21,5221	0,609721	1,00347	0,07825	0,260745	no
XLOC_000970	g5958	51,1259	61,7802	0,273092	0,452403	0,4265	0,676066	no
XLOC_000971	g11750	439,95	520,397	0,242272	0,321621	0,557	0,770021	no
XLOC_000972	g5959	0	0	0	0	1	1	no
XLOC_000973	g5960	19,4535	23,5541	0,275949	0,406079	0,46645	0,706622	no
XLOC_000974	g5961	0	0	0	0	1	1	no
XLOC_000975	g5962	7,56127	8,19723	0,116508	0,123988	0,82395	0,919662	no
XLOC_000976	g5963	19,5644	31,363	0,680833	0,887052	0,1176	0,336391	no
XLOC_000977	g5964	3,06974	4,96661	0,694147	0,784104	0,17795	0,424658	no
XLOC_000978	g5965	0	0	0	0	1	1	no
XLOC_000979	g5966	1,91104	0,310589	-2,62128	-1,45137	0,1209	0,342835	no
XLOC_000980	g11751	230,49	354,301	0,62027	1,08529	0,06465	0,230608	no
XLOC_000981	g1304	1,6243	5,64083	1,79609	1,77476	0,0064	0,048795	yes
XLOC_000982	g1305	0,524927	2,18586	2,05801	1,18056	0,27255	0,534007	no
XLOC_000983	g1306	9,73894	5,2541	-0,890322	-1,17498	0,04465	0,182237	no
XLOC_000984	g145	21,3171	15,3912	-0,46991	-0,643809	0,25175	0,510677	no
XLOC_000985	g147	13,2409	18,2812	0,465366	0,718578	0,20515	0,4589	no
XLOC_000986	g149	13,8072	23,3617	0,758725	1,1634	0,04455	0,18198	no
XLOC_000987	g146	23,9153	12,9395	-0,886154	-1,30387	0,022	0,116526	no
XLOC_000988	g148	48,0151	29,7272	-0,691703	-1,10882	0,05305	0,204615	no
XLOC_000989	g5968	484,512	331,972	-0,545471	-0,805912	0,16485	0,406945	no
XLOC_000990	g5967	39,0069	51,8444	0,410458	0,566519	0,3163	0,577009	no
XLOC_000991	g5969	20,7681	18,2607	-0,185625	-0,245292	0,65895	0,832458	no
XLOC_000992	g11752	0	0	0	0	1	1	no
XLOC_000993	g5970	24,9071	64,9357	1,38246	1,85408	0,00145	0,0164423	yes
XLOC_000994	g1307	3,76408	4,57284	0,280794	0,267571	0,6514	0,829024	no
XLOC_000995	g11753	18,5536	29,7164	0,679558	0,930238	0,1154	0,333204	no
XLOC_000996	g5971	67,2978	136,867	1,02414	1,69432	0,0031	0,0290477	yes
XLOC_000997	g5973	56,1716	58,9286	0,0691285	0,106752	0,85275	0,933661	no
XLOC_000998	g5972	26,5165	52,0698	0,973555	1,52118	0,00815	0,0578273	no
XLOC_000999	g5974	145,149	115,802	-0,325876	-0,543846	0,33005	0,590901	no
XLOC_001000	g5975	0	0	0	0	1	1	no
XLOC_001001	g11754	63,1761	53,3438	-0,244059	-0,41394	0,47555	0,712433	no
XLOC_001002	g5976	0	0	0	0	1	1	no
XLOC_001003	g5977	401,686	359,074	-0,161784	-0,242954	0,67175	0,840955	no
XLOC_001004	g11755	128,858	69,4153	-0,892452	-1,19899	0,0339	0,153141	no
XLOC_001005	g11756	0	0	0	0	1	1	no
XLOC_001006	g5978	33,0744	34,1538	0,04633	0,0764558	0,89415	0,952958	no
XLOC_001007	g11758	40,9126	33,9695	-0,268304	-0,456388	0,40485	0,658433	no
XLOC_001008	g11760	23,8608	12,34	-0,951299	-1,32712	0,0248	0,125793	no
XLOC_001009	g11757	6,08437	4,65992	-0,384805	-0,505448	0,37365	0,63195	no
XLOC_001010	g11759	90,7211	37,3218	-1,28142	-2,15761	0,00025	0,00430702	yes
XLOC_001011	g11761	3,82222	6,47201	0,759802	0,842812	0,13945	0,372621	no

XLOC_001012	g11762	0	0	0	0	1	1	no
XLOC_001013	g5979	0	0	0	0	1	1	no
XLOC_001014	g5980	0	0	0	0	1	1	no
XLOC_001015	g5981	0	0	0	0	1	1	no
XLOC_001016	g5982	0	0	0	0	1	1	no
XLOC_001017	g11763	33,8399	47,2283	0,480928	0,76911	0,1757	0,421955	no
XLOC_001018	g11764	20,8503	7,60348	-1,45534	-2,00663	0,00065	0,00901554	yes
XLOC_001019	g5984	94,7949	63,8271	-0,57064	-0,885998	0,1188	0,338586	no
XLOC_001020	g5985	133,969	127,583	-0,0704565	-0,116987	0,8368	0,925387	no
XLOC_001021	g5983	11,4193	8,51047	-0,424163	-0,519844	0,36385	0,62367	no
XLOC_001022	g5986	31,6246	30,9939	-0,029062	-0,0479365	0,9368	0,972153	no
XLOC_001023	g5987	0	0	0	0	1	1	no
XLOC_001024	g5988	5,09241	6,81779	0,420956	0,55009	0,34845	0,608857	no
XLOC_001025	g1308	0,0732735	0,0383396	-0,934456	0	1	1	no
XLOC_001026	g1309	77,7319	71,3676	-0,123238	-0,207379	0,7107	0,862876	no
XLOC_001027	g1311	263,109	1116,57	2,08534	2,72593	5,00E-05	0,00120049	yes
XLOC_001028	g1313	51,1805	105,385	1,042	1,53231	0,0084	0,0588359	no
XLOC_001029	g1315	140,484	192,027	0,4509	0,611064	0,26585	0,526659	no
XLOC_001030	g1316	73,6266	23,65	-1,63839	-2,15409	0,0002	0,00367103	yes
XLOC_001031	g1319	68,6759	81,0875	0,239676	0,41721	0,47305	0,710407	no
XLOC_001032	g1320	323,713	380,719	0,234011	0,337888	0,56095	0,772043	no
XLOC_001033	g1322	267,554	282,495	0,0783955	0,123562	0,82275	0,919263	no
XLOC_001034	g1323	247,455	176,633	-0,486415	-0,784755	0,16005	0,401272	no
XLOC_001035	g1324	71,6496	68,5936	-0,0628853	-0,10515	0,85525	0,934827	no
XLOC_001036	g1310	32,845	44,1463	0,426618	0,686257	0,21125	0,466594	no
XLOC_001037	g1312	70,5123	185,716	1,39716	1,91818	0,0018	0,0192549	yes
XLOC_001038	g1314	29,0826	67,3045	1,21055	2,00744	0,0006	0,00845337	yes
XLOC_001039	g1317	56,3785	38,5682	-0,547733	-0,913002	0,11885	0,338586	no
XLOC_001040	g1318	44,1539	42,0357	-0,0709265	-0,102728	0,85905	0,935552	no
XLOC_001041	g1321	35,553	57,2087	0,686264	1,154	0,04475	0,182342	no
XLOC_001042	g150	451,06	478,056	0,0838588	0,10455	0,85575	0,934827	no
XLOC_001043	g152	25,0426	21,1229	-0,245579	-0,399445	0,47435	0,711597	no
XLOC_001044	g154	31,0975	37,5631	0,272515	0,450507	0,418	0,669829	no
XLOC_001045	g155	90,0403	79,773	-0,17467	-0,276453	0,623	0,81085	no
XLOC_001046	g156	95,6975	142,521	0,574618	0,97707	0,09305	0,290512	no
XLOC_001047	g157	47,9692	43,4384	-0,143138	-0,209914	0,71095	0,862876	no
XLOC_001048	g151	285,83	284,471	-0,00687458	-0,0102839	0,98575	0,992622	no
XLOC_001049	g153	71,89	76,1124	0,0823406	0,140572	0,80185	0,908624	no
XLOC_001050	g11765	394,828	159,192	-1,31045	-2,01251	0,00055	0,00788467	yes
XLOC_001051	g11766	1289,43	404,687	-1,67186	-2,58372	5,00E-05	0,00120049	yes
XLOC_001052	g5989	34,486	72,7177	1,0763	1,28135	0,0286	0,137614	no
XLOC_001053	g11767	876,028	966,214	0,141367	0,191573	0,7445	0,879571	no
XLOC_001054	g5990	0	0	0	0	1	1	no
XLOC_001055	g11768	0,623839	0,771616	0,306709	0	1	1	no
XLOC_001056	g11769	18,8647	43,8344	1,21637	1,60879	0,00665	0,0501945	no
XLOC_001057	g5991	0	0	0	0	1	1	no
XLOC_001058	g1325	43,7459	12,3397	-1,82584	-2,09728	0,00085	0,011085	yes
XLOC_001059	g5992	0	0	0	0	1	1	no
XLOC_001060	g11770	5,36592	2,62225	-1,03302	-1,03341	0,08125	0,267833	no
XLOC_001061	g5993	33,1762	39,8054	0,262815	0,43849	0,4343	0,681173	no
XLOC_001062	g5995	203,357	143,017	-0,507824	-0,858233	0,13625	0,367778	no
XLOC_001063	g5994	46,1563	43,7062	-0,0786881	-0,123647	0,8319	0,922663	no
XLOC_001064	g5996	6,83621	3,09326	-1,14407	-1,46171	0,01615	0,093071	no
XLOC_001065	g5997	20,5329	16,9803	-0,274072	-0,426085	0,45015	0,694824	no
XLOC_001066	g1326	5,81004	7,80614	0,426062	0,634925	0,25425	0,51341	no
XLOC_001067	g1327	12,0854	6,76217	-0,837702	-0,896617	0,1219	0,344576	no
XLOC_001068	g5998	106,743	112,158	0,0713907	0,110196	0,8505	0,932862	no
XLOC_001069	g5999	32,2899	32,4908	0,0089515	0,0136998	0,98045	0,990282	no
XLOC_001070	g6000	0	0,118989	inf	0	1	1	no
XLOC_001071	g6001	1,78869	7,39702	2,04804	2,03329	0,0022	0,0225747	yes
XLOC_001072	g6002	0	0	0	0	1	1	no
XLOC_001073	g6003	170,063	9,4351	-4,17189	-6,01606	5,00E-05	0,00120049	yes
XLOC_001074	g1328	74,5836	51,6112	-0,531174	-0,722653	0,1965	0,447606	no
XLOC_001075	g1329	2,62807	3,76299	0,517879	0,651543	0,2406	0,49898	no
XLOC_001076	g6004	7,53811	12,0234	0,673568	0,716334	0,2174	0,474204	no
XLOC_001077	g11771	198,843	174,446	-0,18885	-0,31017	0,58045	0,783024	no
XLOC_001078	g6006	0	0	0	0	1	1	no
XLOC_001079	g6007	0	0	0	0	1	1	no
XLOC_001080	g6005	0	0	0	0	1	1	no
XLOC_001081	g6008	10,0237	9,2314	-0,118788	-0,151065	0,7996	0,907867	no
XLOC_001082	g11772	0	0	0	0	1	1	no
XLOC_001083	g6009	43,3163	23,9931	-0,852287	-1,15238	0,04745	0,189337	no
XLOC_001084	g6011	40,8823	15,9678	-1,35631	-1,97075	0,00105	0,0130684	yes
XLOC_001085	g6014	1881,11	2784,77	0,56597	0,920228	0,1159	0,333955	no
XLOC_001086	g6015	3,69579	2,06903	-0,836928	-0,918273	0,1155	0,333246	no
XLOC_001087	g6010	16,5531	18,214	0,137954	0,215356	0,6844	0,84822	no
XLOC_001088	g6012	11,3527	11,9319	0,0717897	0,0760112	0,896	0,953687	no
XLOC_001089	g6013	9,00625	7,17252	-0,328445	-0,491407	0,38275	0,639193	no
XLOC_001090	g6016	25,2679	22,1973	-0,186921	-0,313606	0,58325	0,784861	no
XLOC_001091	g6017	40,6057	21,5399	-0,914666	-1,25693	0,0284	0,137046	no
XLOC_001092	g11774	16,2921	11,7652	-0,469645	-0,705882	0,219	0,476213	no
XLOC_001093	g11773	31,3449	15,0221	-1,06114	-1,47721	0,01085	0,070421	no
XLOC_001094	g11775	73,6619	12,774	-2,5277	-3,78085	5,00E-05	0,00120049	yes
XLOC_001095	g6018	25,8669	19,1991	-0,430065	-0,695546	0,21855	0,475656	no
XLOC_001096	g6019	104,62	273,573	1,38677	1,7952	0,00195	0,0205021	yes
XLOC_001097	g1330	0	0	0	0	1	1	no
XLOC_001098	g11776	0	0	0	0	1	1	no
XLOC_001099	g11777	26,0481	56,1116	1,10712	1,56102	0,00735	0,0538233	no
XLOC_001100	g11778	0	0	0	0	1	1	no
XLOC_001101	g11779	0	0	0	0	1	1	no
XLOC_001102	g6023	132,527	126,97	-0,0618026	-0,10153	0,85485	0,93471	no
XLOC_001103	g6020	109,119	140,378	0,363405	0,59739	0,2936	0,555829	no

XLOC_001104	g6021	23,1646	21,2367	-0,125362	-0,196965	0,7356	0,874871	no
XLOC_001105	g6022	41,6168	64,13	0,623833	1,06495	0,05885	0,21734	no
XLOC_001106	g11780	0	0	0	0	1	1	no
XLOC_001107	g11781	98,3937	112,763	0,196654	0,276463	0,63395	0,816873	no
XLOC_001108	g6025	11,6718	11,8028	0,0161039	0,0202752	0,9708	0,986977	no
XLOC_001109	g6024	8,98328	3,75943	-1,25673	-1,7187	0,0045	0,038062	yes
XLOC_001110	g6026	0	0	0	0	1	1	no
XLOC_001111	g1331	115,4	39,7977	-1,53589	-2,49726	5,00E-05	0,00120049	yes
XLOC_001112	g11782	92,314	51,3232	-0,846939	-1,44603	0,0138	0,0837035	no
XLOC_001113	g11783	0	0	0	0	1	1	no
XLOC_001114	g1333	29,7614	29,2667	-0,0241825	-0,0418083	0,9452	0,975499	no
XLOC_001115	g1335	18,0847	41,5437	1,19986	1,99232	0,00025	0,00430702	yes
XLOC_001116	g1337	38,3904	76,775	0,999891	1,63692	0,0053	0,0425908	yes
XLOC_001117	g1338	307,119	3154,1	3,36036	3,95416	5,00E-05	0,00120049	yes
XLOC_001118	g1339	65,7109	41,8972	-0,64928	-1,1112	0,05145	0,200095	no
XLOC_001119	g1340	31,9774	27,761	-0,203989	-0,332708	0,5491	0,765215	no
XLOC_001120	g1332	10,1502	8,20881	-0,306269	-0,422418	0,458	0,701013	no
XLOC_001121	g1334	11,0727	26,4312	1,25523	1,25466	0,0355	0,157671	no
XLOC_001122	g1336	108,821	81,0267	-0,425493	-0,552348	0,32405	0,584313	no
XLOC_001123	g11784	0	0	0	0	1	1	no
XLOC_001124	g1341	40,7165	26,662	-0,610826	-1,00256	0,08985	0,284255	no
XLOC_001125	g6027	0	0	0	0	1	1	no
XLOC_001126	g6028	4,87007	3,00362	-0,697241	-0,52013	0,52455	0,746425	no
XLOC_001127	g11785	25,523	54,8191	1,10288	1,36742	0,02295	0,119813	no
XLOC_001128	g6029	15,0021	27,992	0,899847	1,38295	0,01315	0,0811137	no
XLOC_001129	g11786	209,556	89,0343	-1,2349	-1,92232	0,0009	0,0115984	yes
XLOC_001130	g6030	138,638	79,9355	-0,794412	-1,07285	0,0616	0,22371	no
XLOC_001131	g6031	198,505	124,904	-0,668349	-1,13084	0,0532	0,204792	no
XLOC_001132	g6032	87,3832	5,96587	-3,87255	-4,84584	5,00E-05	0,00120049	yes
XLOC_001133	g6033	26,4217	12,4626	-1,08412	-1,19163	0,03875	0,166386	no
XLOC_001134	g6034	0	0	0	0	1	1	no
XLOC_001135	g6035	0	0	0	0	1	1	no
XLOC_001136	g11787	8,72502	8,16906	-0,0949881	-0,124985	0,8271	0,921084	no
XLOC_001137	g6036	5,69655	6,63364	0,219711	0,276704	0,63995	0,82122	no
XLOC_001138	g6037	1,42809	0,887995	-0,685466	-0,572861	0,30425	0,565752	no
XLOC_001139	g6038	0	0	0	0	1	1	no
XLOC_001140	g6039	16,4095	10,7896	-0,604891	-0,86046	0,1286	0,354275	no
XLOC_001141	g6040	0,672224	0,636411	-0,0789816	0	1	1	no
XLOC_001142	g6041	53,1676	39,284	-0,436608	-0,731458	0,20895	0,464018	no
XLOC_001143	g6042	15,0397	10,0033	-0,588304	-0,813103	0,15555	0,392876	no
XLOC_001144	g6043	0	0,179573	inf	0	1	1	no
XLOC_001145	g11788	0	0	0	0	1	1	no
XLOC_001146	g6044	0,300619	0	#NAME?	0	1	1	no
XLOC_001147	g6045	340,456	64,5639	-2,39867	-2,62141	5,00E-05	0,00120049	yes
XLOC_001148	g6046	15,4365	16,3259	0,0808164	0,115045	0,83895	0,92642	no
XLOC_001149	g6047	0	0	0	0	1	1	no
XLOC_001150	g6048	0	2,29373	inf	#NAME?	5,00E-05	0,00120049	yes
XLOC_001151	g11789	5,57928	8,10607	0,538923	0,790855	0,17865	0,425811	no
XLOC_001152	g11792	66,3426	70,6809	0,0913841	0,155762	0,78615	0,901303	no
XLOC_001153	g11790	28,1508	22,591	-0,31743	-0,51597	0,3735	0,631938	no
XLOC_001154	g11791	56,4098	84,5261	0,583451	0,930124	0,10195	0,307384	no
XLOC_001155	g6049	0	0	0	0	1	1	no
XLOC_001156	g1343	146,172	256,129	0,809201	1,11884	0,057	0,213968	no
XLOC_001157	g1345	110,131	61,7581	-0,834518	-1,20188	0,03825	0,165396	no
XLOC_001158	g1347	22,1074	21,5916	-0,0340615	-0,0523826	0,9249	0,966895	no
XLOC_001159	g1342	20,242	15,1816	-0,415033	-0,642957	0,25985	0,52023	no
XLOC_001160	g1344	15,4387	9,99871	-0,626737	-0,912759	0,1154	0,333204	no
XLOC_001161	g1346	25,9114	44,6463	0,78495	1,25206	0,03335	0,151479	no
XLOC_001162	g1348	6,01797	5,52671	-0,122856	-0,179427	0,75565	0,884596	no
XLOC_001163	g6050	33,0363	20,2099	-0,708987	-1,12902	0,04715	0,188831	no
XLOC_001164	g6051	0	0	0	0	1	1	no
XLOC_001165	g11793	0	0	0	0	1	1	no
XLOC_001166	g11794	62,421	61,7615	-0,0153225	-0,023261	0,96685	0,985414	no
XLOC_001167	g11795	39,1923	46,1779	0,236632	0,365752	0,5049	0,732907	no
XLOC_001168	g11796	55,4636	75,0204	0,43574	0,749835	0,18825	0,437335	no
XLOC_001169	g6052	13,1004	9,8429	-0,412452	-0,401902	0,51575	0,741858	no
XLOC_001170	g1352	22,0923	16,3906	-0,430676	-0,47966	0,39115	0,646031	no
XLOC_001171	g1353	37,4176	30,1463	-0,311735	-0,475057	0,4107	0,663116	no
XLOC_001172	g1357	53,233	144,001	1,43568	2,14763	0,00035	0,00552572	yes
XLOC_001173	g1358	25,1209	30,8024	0,294153	0,4937	0,3815	0,637999	no
XLOC_001174	g1359	14,5212	12,9959	-0,160105	-0,236519	0,67225	0,840955	no
XLOC_001175	g1360	65,7049	22,5656	-1,54187	-1,10639	0,06825	0,238941	no
XLOC_001176	g1349	11,7481	5,79474	-1,01961	-1,31073	0,0225	0,118408	no
XLOC_001177	g1350	173,8	57,2191	-1,60286	-2,225	0,00035	0,00552572	yes
XLOC_001178	g1351	249,878	69,9995	-1,83581	-2,66289	5,00E-05	0,00120049	yes
XLOC_001179	g1354	1,03495	0,831479	-0,315804	0	1	1	no
XLOC_001180	g1355	171,841	88,6949	-0,954153	-1,59262	0,00315	0,0293482	yes
XLOC_001181	g1356	93,0838	90,5564	-0,0397121	-0,0662149	0,90395	0,957996	no
XLOC_001182	g6053	19,2726	12,0961	-0,672005	-1,00535	0,07755	0,258939	no
XLOC_001183	g6054	15,6199	30,7668	0,977988	1,32393	0,02585	0,129382	no
XLOC_001184	g6056	220,749	193,399	-0,190825	-0,294264	0,6018	0,798173	no
XLOC_001185	g6057	74,196	100,459	0,437189	0,722221	0,1996	0,451942	no
XLOC_001186	g6059	82,9312	88,3894	0,0919573	0,148433	0,79625	0,906216	no
XLOC_001187	g6061	97,2325	102,736	0,0794307	0,133191	0,81925	0,91765	no
XLOC_001188	g6055	71,0212	81,0724	0,190961	0,322768	0,56	0,771227	no
XLOC_001189	g6058	87,4426	98,4269	0,170716	0,29214	0,6032	0,798843	no
XLOC_001190	g6060	1239,52	1929,96	0,638792	1,01443	0,0716	0,246533	no
XLOC_001191	g6062	86,2293	112,636	0,385418	0,656375	0,23695	0,494864	no
XLOC_001192	g1361	0,0148186	0,0910722	2,6196	0	1	1	no
XLOC_001193	g11797	9,04197	5,93125	-0,608302	-0,853868	0,12435	0,347599	no
XLOC_001194	g11798	0	0	0	0	1	1	no
XLOC_001195	g1363	84,5344	73,3147	-0,205436	-0,328841	0,57115	0,778297	no

XLOC_001196	g1365	70,5561	47,6984	-0,564831	-0,961841	0,0867	0,278142	no
XLOC_001197	g1367	35,2008	66,5779	0,919436	1,56757	0,00795	0,056695	no
XLOC_001198	g1368	47,3877	35,049	-0,43514	-0,736608	0,1817	0,42933	no
XLOC_001199	g1370	0,606663	0,587043	-0,0474304	0	1	1	no
XLOC_001200	g1362	22,9443	26,867	0,227696	0,339463	0,55205	0,766299	no
XLOC_001201	g1364	93,8105	112,178	0,257968	0,419827	0,468	0,707366	no
XLOC_001202	g1366	172,347	185,697	0,107638	0,169345	0,76025	0,886938	no
XLOC_001203	g1369	119,402	148,394	0,313607	0,556641	0,3465	0,606638	no
XLOC_001204	g6063	12,1118	19,8699	0,714175	0,69994	0,2343	0,491629	no
XLOC_001205	g6064	0	0,0229284	inf	0	1	1	no
XLOC_001206	g6065	0	0	0	0	1	1	no
XLOC_001207	g1371	7,39192	13,3458	0,852363	1,06238	0,0699	0,242551	no
XLOC_001208	g1372	17,8942	22,9251	0,357441	0,595415	0,29855	0,560566	no
XLOC_001209	g1374	7,98362	6,09992	-0,388251	-0,41664	0,4682	0,707556	no
XLOC_001210	g1376	0	0,413904	inf	0	1	1	no
XLOC_001211	g1373	73,6938	82,5745	0,164154	0,278591	0,6294	0,814495	no
XLOC_001212	g1375	26,5056	49,4813	0,900586	1,19376	0,0409	0,172008	no
XLOC_001213	g6066	72,1887	91,8338	0,347251	0,513688	0,3756	0,633962	no
XLOC_001214	g6068	2,67092	2,08814	-0,355118	-0,372674	0,5237	0,746189	no
XLOC_001215	g6069	10,7059	10,914	0,0277642	0,0318757	0,95175	0,978761	no
XLOC_001216	g6067	1,54864	1,43836	-0,106575	-0,0672016	0,87105	0,94235	no
XLOC_001217	g6070	92,4391	53,7593	-0,78199	-1,32479	0,019	0,104703	no
XLOC_001218	g6072	158,572	184,292	0,216857	0,359841	0,53225	0,752692	no
XLOC_001219	g6074	62,3215	68,4217	0,134725	0,220211	0,6965	0,855917	no
XLOC_001220	g6071	35,6648	39,1811	0,135657	0,22794	0,6892	0,850886	no
XLOC_001221	g6073	27,4415	19,8426	-0,467757	-0,681897	0,23895	0,497032	no
XLOC_001222	g1377	212,087	260,1	0,294413	0,496355	0,38485	0,640945	no
XLOC_001223	g1379	42,7114	42,184	-0,0179251	-0,0302354	0,9581	0,981385	no
XLOC_001224	g1378	19,2204	16,3889	-0,229919	-0,376883	0,502	0,730642	no
XLOC_001225	g11801	31,4912	29,9307	-0,0733225	-0,116627	0,8384	0,926315	no
XLOC_001226	g11804	278,661	308,38	0,146202	0,162975	0,77745	0,895827	no
XLOC_001227	g11806	2,74507	4,86938	0,826895	0,91483	0,12245	0,345717	no
XLOC_001228	g11808	166,577	224,69	0,431742	0,592465	0,29195	0,554536	no
XLOC_001229	g11799	63,9267	58,4965	-0,128068	-0,192172	0,7417	0,877635	no
XLOC_001230	g11800	51,2409	283,422	2,46758	3,00396	5,00E-05	0,00120049	yes
XLOC_001231	g11802	193,295	167,185	-0,20936	-0,363005	0,5311	0,751607	no
XLOC_001232	g11803	65,7901	97,1459	0,562283	0,96335	0,09265	0,290215	no
XLOC_001233	g11805	7,28725	11,9844	0,717717	1,02129	0,07595	0,25481	no
XLOC_001234	g11807	55,6846	62,6672	0,170433	0,292887	0,6157	0,807125	no
XLOC_001235	g6075	0	3,50175	inf	0	1	1	no
XLOC_001236	g6076	0	0	0	0	1	1	no
XLOC_001237	g6077	68,4416	124,597	0,864318	1,36545	0,0158	0,0916998	no
XLOC_001238	g6079	32,8504	49,2245	0,583465	0,892594	0,12045	0,341855	no
XLOC_001239	g6078	241,79	145,64	-0,731346	-1,18965	0,03815	0,165182	no
XLOC_001240	g6080	70,7753	67,8906	-0,0600343	-0,101934	0,8527	0,933661	no
XLOC_001241	g11809	104,919	56,9169	-0,882341	-1,36557	0,0234	0,121645	no
XLOC_001242	g11810	74,0868	29,8172	-1,31307	-1,9603	0,00125	0,0146655	yes
XLOC_001243	g1380	107,284	115,001	0,100215	0,169542	0,76035	0,886938	no
XLOC_001244	g1381	69,5917	48,0392	-0,534703	-0,880545	0,1146	0,332262	no
XLOC_001245	g1383	179,584	246,908	0,459317	0,684557	0,2333	0,49079	no
XLOC_001246	g1384	28,8056	26,4794	-0,121481	-0,195585	0,73005	0,871788	no
XLOC_001247	g1385	11,6713	6,7489	-0,790242	-0,608008	0,50325	0,731485	no
XLOC_001248	g1382	95,6552	74,1403	-0,367585	-0,620643	0,278	0,539702	no
XLOC_001249	g6081	103,37	122,72	0,24755	0,426719	0,4597	0,701734	no
XLOC_001250	g6082	36,2608	35,6928	-0,0227745	-0,0375379	0,94695	0,97628	no
XLOC_001251	g6084	26,6221	94,4647	1,82715	2,49294	5,00E-05	0,00120049	yes
XLOC_001252	g6083	18,5855	15,5127	-0,260723	-0,368451	0,50785	0,734836	no
XLOC_001253	g6085	0	0	0	0	1	1	no
XLOC_001254	g6086	6,18754	9,68477	0,646352	0,824649	0,1543	0,392241	no
XLOC_001255	g11811	876,844	285,58	-1,61843	-2,23826	0,0002	0,00367103	yes
XLOC_001256	g6087	36,1394	34,2886	-0,0758434	-0,128072	0,8147	0,915896	no
XLOC_001257	g6088	8,81439	10,1677	0,206063	0,27504	0,62655	0,812884	no
XLOC_001258	g6089	127,941	117,582	-0,121813	-0,181267	0,75215	0,88326	no
XLOC_001259	g6090	216,26	221,287	0,0331505	0,053198	0,9259	0,967167	no
XLOC_001260	g6091	116,697	19,4458	-2,58523	-3,95936	5,00E-05	0,00120049	yes
XLOC_001261	g6093	13,8019	7,64876	-0,851573	-1,10502	0,0603	0,22095	no
XLOC_001262	g6092	22,5447	22,1378	-0,0262776	-0,0420381	0,94085	0,973926	no
XLOC_001263	g6094	3,42329	4,33535	0,340764	0,37246	0,5075	0,734618	no
XLOC_001264	g11812	157,814	132,316	-0,254235	-0,405301	0,47985	0,715476	no
XLOC_001265	g11813	0	0	0	0	1	1	no
XLOC_001266	g6095	329,755	408,592	0,309265	0,410873	0,47865	0,714229	no
XLOC_001267	g11814	0	0	0	0	1	1	no
XLOC_001268	g11815	0	0,020952	inf	0	1	1	no
XLOC_001269	g11816	0	0	0	0	1	1	no
XLOC_001270	g6097	74,2535	76,7262	0,0472607	0,0773173	0,8919	0,951696	no
XLOC_001271	g6096	14,628	12,8054	-0,191984	-0,27674	0,62985	0,814905	no
XLOC_001272	g6098	7,60497	27,5827	1,85875	2,61204	5,00E-05	0,00120049	yes
XLOC_001273	g6099	0	0	0	0	1	1	no
XLOC_001274	g11817	0	0	0	0	1	1	no
XLOC_001275	g6101	35,6847	50,7901	0,509243	0,850275	0,13475	0,364932	no
XLOC_001276	g6100	65,879	47,9197	-0,459199	-0,783803	0,16775	0,410902	no
XLOC_001277	g11818	0	0	0	0	1	1	no
XLOC_001278	g11820	40,4337	7,53931	-2,42305	-3,67115	5,00E-05	0,00120049	yes
XLOC_001279	g11819	73,3858	13,3576	-2,45784	-3,11376	5,00E-05	0,00120049	yes
XLOC_001280	g11821	140,73	144,309	0,0362357	0,0606769	0,912	0,962529	no
XLOC_001281	g11822	0	4,76727	inf	0	1	1	no
XLOC_001282	g11823	0	0	0	0	1	1	no
XLOC_001283	g11824	0	0	0	0	1	1	no
XLOC_001284	g6102	0	0	0	0	1	1	no
XLOC_001285	g11825	0,206442	0	#NAME?	0	1	1	no
XLOC_001286	g6103	13,6293	12,1343	-0,167631	-0,23447	0,67145	0,840918	no
XLOC_001287	g6104	6,45052	4,94043	-0,384779	-0,526752	0,34925	0,610104	no

XLOC_001288	g6105	25,832	16,0166	-0,689586	-1,06348	0,06475	0,230629	no
XLOC_001289	g11826	1,76149	2,20366	0,323107	0,410661	0,4853	0,719022	no
XLOC_001290	g11827	29,9893	22,3263	-0,425705	-0,492938	0,387	0,643156	no
XLOC_001291	g6106	25,4775	29,614	0,21706	0,306	0,59865	0,7955	no
XLOC_001292	g6107	114,727	417,262	1,86275	3,09663	5,00E-05	0,00120049	yes
XLOC_001293	g11828	75,7334	60,0277	-0,3353	-0,47932	0,3896	0,644629	no
XLOC_001294	g6108	220,87	268,326	0,280789	0,340803	0,55135	0,766024	no
XLOC_001295	g11829	54,8991	39,333	-0,481043	-0,822103	0,14535	0,381641	no
XLOC_001296	g6109	0	0	0	0	1	1	no
XLOC_001297	g1387	3,17131	19,2434	2,60121	2,23999	0,2273	0,485658	no
XLOC_001298	g1389	237,612	303,867	0,35483	0,474562	0,41135	0,663919	no
XLOC_001299	g1386	15,2002	21,7352	0,515943	0,693735	0,23795	0,496002	no
XLOC_001300	g1388	160,637	123,81	-0,375683	-0,553213	0,3151	0,57568	no
XLOC_001301	g11830	13,0173	10,187	-0,353693	-0,463557	0,4241	0,673349	no
XLOC_001302	g11831	0	0	0	0	1	1	no
XLOC_001303	g11832	0	0	0	0	1	1	no
XLOC_001304	g6110	95,3898	257,296	1,43152	2,32237	0,0001	0,00209829	yes
XLOC_001305	g6111	105,452	131,175	0,314906	0,53509	0,34065	0,600895	no
XLOC_001306	g6112	230,589	557,077	1,27256	2,1406	0,0002	0,00367103	yes
XLOC_001307	g11833	25,284	19,7476	-0,356551	-0,545229	0,3418	0,601843	no
XLOC_001308	g11835	5,35902	6,35692	0,246359	0,213956	0,70865	0,861789	no
XLOC_001309	g11834	49,0845	25,0761	-0,968955	-1,38418	0,0173	0,097748	no
XLOC_001310	g11836	52,8561	75,4621	0,513683	0,820386	0,1529	0,390806	no
XLOC_001311	g11838	75,9099	100,728	0,408102	0,703087	0,2265	0,484793	no
XLOC_001312	g11840	108,086	107,558	-0,00706436	-0,0109446	0,9848	0,992379	no
XLOC_001313	g11842	23,682	37,0966	0,647492	0,964237	0,09355	0,291546	no
XLOC_001314	g11844	121,141	185,85	0,617457	1,053	0,0657	0,232663	no
XLOC_001315	g11846	19,9434	17,4929	-0,189139	-0,312261	0,58875	0,789071	no
XLOC_001316	g11847	70,5199	78,5265	0,155148	0,254636	0,6579	0,83212	no
XLOC_001317	g11837	139,61	180,923	0,373968	0,601875	0,27725	0,538905	no
XLOC_001318	g11839	22,3803	31,8053	0,507034	0,777679	0,16475	0,406903	no
XLOC_001319	g11841	123,6	147,701	0,256991	0,422912	0,4583	0,701013	no
XLOC_001320	g11843	1370,78	2168,58	0,661756	0,748312	0,19535	0,446852	no
XLOC_001321	g11845	23,1581	42,642	0,880759	1,51199	0,00855	0,0597587	no
XLOC_001322	g11848	184,786	275,718	0,577339	0,83935	0,1338	0,363462	no
XLOC_001323	g6113	5,05282	1,91404	-1,40047	-1,47285	0,01505	0,0887634	no
XLOC_001324	g6114	121,04	128,42	0,08539	0,147128	0,79335	0,904633	no
XLOC_001325	g6115	5,42367	5,75016	0,0843325	0,108016	0,85465	0,934699	no
XLOC_001326	g1391	75,4462	51,0425	-0,563749	-0,880883	0,11365	0,330288	no
XLOC_001327	g1392	58,8279	89,7462	0,609351	0,891682	0,12255	0,345717	no
XLOC_001328	g1390	31,787	36,9327	0,216462	0,344137	0,5438	0,761351	no
XLOC_001329	g1393	15,4039	38,8425	1,33434	2,03773	0,0003	0,00496796	yes
XLOC_001330	g11849	281,298	344,266	0,291421	0,481811	0,3953	0,649464	no
XLOC_001331	g11850	18,9005	16,3248	-0,211363	-0,315015	0,57635	0,780604	no
XLOC_001332	g1395	2057,92	1733,54	-0,247463	-0,38974	0,48015	0,715489	no
XLOC_001333	g1397	99,2381	135,931	0,453911	0,657728	0,25285	0,511956	no
XLOC_001334	g1399	95,1971	87,4552	-0,122373	-0,205835	0,71485	0,865069	no
XLOC_001335	g1400	44,6478	71,4564	0,678474	1,108	0,04765	0,189673	no
XLOC_001336	g1402	29,2923	20,7472	-0,497606	-0,846352	0,13315	0,3628	no
XLOC_001337	g1394	79,3838	64,8537	-0,291655	-0,417059	0,4674	0,707046	no
XLOC_001338	g1396	32,047	52,9039	0,723183	1,16869	0,03655	0,160304	no
XLOC_001339	g1398	1742,74	679,624	-1,35855	-1,56601	0,0146	0,0871033	no
XLOC_001340	g1401	40,2102	65,2095	0,697522	1,17795	0,04415	0,180927	no
XLOC_001341	g11851	24,2742	31,5202	0,376855	0,567914	0,29105	0,553778	no
XLOC_001342	g11852	61,2352	43,5729	-0,49093	-0,830922	0,14985	0,387142	no
XLOC_001343	g11855	14,7222	5,42611	-1,44	-2,02601	0,00165	0,0180837	yes
XLOC_001344	g11857	29,3572	16,0906	-0,867494	-1,32988	0,02015	0,109443	no
XLOC_001345	g11853	31,5344	22,9168	-0,460522	-0,768297	0,1636	0,405797	no
XLOC_001346	g11854	449,631	650,654	0,533147	0,654198	0,25255	0,511771	no
XLOC_001347	g11856	45,2741	14,9622	-1,59737	-2,42799	5,00E-05	0,00120049	yes
XLOC_001348	g11858	59,6895	51,5576	-0,211293	-0,322189	0,56285	0,772925	no
XLOC_001349	g11859	0	0	0	0	1	1	no
XLOC_001350	g11860	0	0,111173	inf	0	1	1	no
XLOC_001351	g1403	6,34197	3,059	-1,05187	-1,0513	0,06685	0,235715	no
XLOC_001352	g6117	50,9463	50,8921	-0,00153625	-0,00258363	0,99695	0,997966	no
XLOC_001353	g6116	111,574	144,601	0,374076	0,643193	0,2603	0,520918	no
XLOC_001354	g6118	105,218	573,944	2,44753	3,56003	5,00E-05	0,00120049	yes
XLOC_001355	g6119	60,7329	70,0677	0,206273	0,347995	0,54175	0,759889	no
XLOC_001356	g6120	82,2944	197,246	1,26113	2,14173	0,0002	0,00367103	yes
XLOC_001357	g11861	27,4728	40,4796	0,559188	0,585618	0,3215	0,581638	no
XLOC_001358	g11862	95,643	113,795	0,250703	0,417735	0,46965	0,707649	no
XLOC_001359	g6121	63,8684	75,6126	0,243524	0,38666	0,49355	0,724355	no
XLOC_001360	g6123	432,712	678,592	0,649138	0,960213	0,1016	0,3068	no
XLOC_001361	g6124	39,0784	46,6168	0,254477	0,431365	0,45015	0,694824	no
XLOC_001362	g6127	1,09917	0,981997	-0,162625	-0,127453	0,82045	0,918365	no
XLOC_001363	g6128	18,0149	5,73744	-1,65071	-2,25771	0,0002	0,00367103	yes
XLOC_001364	g6131	90,1026	65,3796	-0,462727	-0,779041	0,16755	0,410822	no
XLOC_001365	g6132	52,9769	60,5479	0,192716	0,299048	0,6031	0,798819	no
XLOC_001366	g6122	8,13027	10,2882	0,339616	0,518434	0,3445	0,604645	no
XLOC_001367	g6125	33,0604	41,0291	0,31154	0,517434	0,3433	0,60351	no
XLOC_001368	g6126	97,5298	48,3585	-1,01207	-1,64111	0,0053	0,0425908	yes
XLOC_001369	g6129	376,326	486,449	0,370308	0,569278	0,3164	0,577009	no
XLOC_001370	g6130	69,4094	44,6541	-0,636338	-0,995162	0,08545	0,276208	no
XLOC_001371	g11863	0	0,227316	inf	0	1	1	no
XLOC_001372	g6133	0	0	0	0	1	1	no
XLOC_001373	g6135	64,8892	52,2933	-0,311351	-0,529157	0,3455	0,605535	no
XLOC_001374	g6134	324,697	100,651	-1,68973	-2,47632	0,0001	0,00209829	yes
XLOC_001375	g6136	13,3395	8,6939	-0,617635	-0,842861	0,15055	0,387828	no
XLOC_001376	g6137	66,7874	29,4201	-1,18277	-1,64887	0,0052	0,0421668	yes
XLOC_001377	g6138	2,49285	1,76878	-0,495041	-0,475696	0,4462	0,692396	no
XLOC_001378	g11864	33,8868	27,9981	-0,275392	-0,39037	0,48685	0,720553	no
XLOC_001379	g6139	114,282	96,9357	-0,237499	-0,292931	0,61485	0,806441	no

XLOC_001380	g11865	0,0257126	0	#NAME?	0	1	1	no
XLOC_001381	g11866	80,0998	74,9092	-0,0966543	-0,165394	0,76915	0,89269	no
XLOC_001382	g11867	34,2048	52,3002	0,61262	0,814247	0,16615	0,409249	no
XLOC_001383	g158	2,29205	2,54971	0,153699	0,168846	0,7724	0,894131	no
XLOC_001384	g159	20,8706	13,6242	-0,615308	-0,907481	0,1061	0,315824	no
XLOC_001385	g11868	1269	1308,85	0,044604	0,0655246	0,9084	0,961052	no
XLOC_001386	g11869	129,583	157,477	0,281261	0,437433	0,44535	0,691546	no
XLOC_001387	g11870	116,585	181,362	0,637482	0,926971	0,10845	0,320198	no
XLOC_001388	g6140	0	0	0	0	1	1	no
XLOC_001389	g6141	113,085	159,368	0,494959	0,740264	0,1845	0,432718	no
XLOC_001390	g6143	55,972	47,2665	-0,243888	-0,409384	0,47285	0,710324	no
XLOC_001391	g6142	260,313	276,523	0,0871527	0,148787	0,79	0,90312	no
XLOC_001392	g6144	45,3916	44,6822	-0,0227245	-0,0368591	0,9476	0,976282	no
XLOC_001393	g6145	0,0250208	0,0223751	-0,161229	0	1	1	no
XLOC_001394	g11871	0	0	0	0	1	1	no
XLOC_001395	g11872	0	0	0	0	1	1	no
XLOC_001396	g6146	0	0	0	0	1	1	no
XLOC_001397	g6148	51,8207	29,6145	-0,807222	-1,1681	0,0364	0,159932	no
XLOC_001398	g6147	48,8364	61,7359	0,338153	0,481108	0,40015	0,654149	no
XLOC_001399	g11873	0	0	0	0	1	1	no
XLOC_001400	g11874	2,36995	4,47603	0,917364	0,959909	0,09705	0,298195	no
XLOC_001401	g11875	9,47098	10,2452	0,113356	0,120493	0,83035	0,922276	no
XLOC_001402	g11876	166,062	120,779	-0,459348	-0,718585	0,21175	0,467278	no
XLOC_001403	g11877	0	0	0	0	1	1	no
XLOC_001404	g11879	201,596	57,797	-1,8024	-1,98448	0,0007	0,00954722	yes
XLOC_001405	g11881	98,3792	112,327	0,191283	0,323597	0,5741	0,779975	no
XLOC_001406	g11878	44,5867	45,346	0,0243616	0,0411545	0,9426	0,974864	no
XLOC_001407	g11880	26,1263	23,0506	-0,1807	-0,278799	0,6233	0,811025	no
XLOC_001408	g11882	2,76646	1,64309	-0,751626	-0,965487	0,09385	0,292295	no
XLOC_001409	g11883	9,37873	9,94647	0,0847917	0,12399	0,83615	0,925299	no
XLOC_001410	g11884	263,685	219,331	-0,265704	-0,426004	0,4583	0,701013	no
XLOC_001411	g6149	63,8812	91,5708	0,519496	0,812656	0,14275	0,378049	no
XLOC_001412	g11885	85,1886	48,9296	-0,799955	-1,32114	0,02425	0,12409	no
XLOC_001413	g11887	57,3022	26,3794	-1,11918	-1,85815	0,00155	0,0171215	yes
XLOC_001414	g11888	31,1343	8,18763	-1,92699	-3,06316	5,00E-05	0,00120049	yes
XLOC_001415	g11890	14,9651	11,5419	-0,374716	-0,525559	0,3689	0,628841	no
XLOC_001416	g11886	26,7093	16,1565	-0,725228	-1,13783	0,04085	0,172008	no
XLOC_001417	g11889	3,30113	2,36486	-0,481206	-0,470302	0,4116	0,664133	no
XLOC_001418	g11891	24,4541	40,2518	0,718977	1,03609	0,06225	0,224989	no
XLOC_001419	g6150	0	0	0	0	1	1	no
XLOC_001420	g160	0,222555	0,275369	0,307206	0	1	1	no
XLOC_001421	g6151	199,253	108,241	-0,880352	-1,49585	0,01235	0,0774438	no
XLOC_001422	g6153	12,6142	9,25996	-0,445967	-0,59185	0,2884	0,551849	no
XLOC_001423	g6152	10,7645	4,34314	-1,30947	-1,33351	0,0325	0,148719	no
XLOC_001424	g11892	112,239	80,386	-0,481561	-0,8031	0,15435	0,392266	no
XLOC_001425	g6154	81,4049	49,958	-0,7044	-1,08331	0,06615	0,233919	no
XLOC_001426	g11893	6,33669	13,5179	1,09307	1,4952	0,0091	0,0622298	no
XLOC_001427	g11894	14,1	23,9344	0,763387	1,1257	0,056	0,211697	no
XLOC_001428	g11895	241,924	679,152	1,48918	2,1497	0,00045	0,00669545	yes
XLOC_001429	g6155	44,1717	64,6722	0,550024	0,767116	0,17615	0,422829	no
XLOC_001430	g6156	18,408	27,7256	0,590887	0,827745	0,1555	0,392876	no
XLOC_001431	g6157	649,151	682,405	0,0720747	0,118156	0,8341	0,924164	no
XLOC_001432	g11896	0,84458	4,04852	2,26109	1,60682	0,25775	0,517915	no
XLOC_001433	g1404	189,385	152,337	-0,314057	-0,394339	0,4758	0,712433	no
XLOC_001434	g1405	34,7822	34,1309	-0,0272683	-0,0467941	0,9343	0,971293	no
XLOC_001435	g1407	37,4885	40,0195	0,0942561	0,131897	0,81445	0,91572	no
XLOC_001436	g1406	37,6645	33,9953	-0,147869	-0,244622	0,6737	0,8415	no
XLOC_001437	g1408	20,9833	18,0209	-0,219569	-0,316251	0,57145	0,77837	no
XLOC_001438	g6158	0	0	0	0	1	1	no
XLOC_001439	g6159	0	0	0	0	1	1	no
XLOC_001440	g11897	67,5903	85,9089	0,345992	0,478024	0,40075	0,654912	no
XLOC_001441	g11900	147,337	259,152	0,814679	1,36465	0,01555	0,0907314	no
XLOC_001442	g11902	22,1026	30,6429	0,47134	0,718736	0,21505	0,470651	no
XLOC_001443	g11904	29,1772	47,8785	0,714536	1,02721	0,0713	0,245672	no
XLOC_001444	g11905	40,9726	45,179	0,140993	0,214215	0,71105	0,862891	no
XLOC_001445	g11908	59,3917	103,186	0,796921	1,34853	0,0208	0,112044	no
XLOC_001446	g11898	77,1161	61,2162	-0,333119	-0,571128	0,3564	0,617039	no
XLOC_001447	g11899	189,948	155,615	-0,287628	-0,468802	0,3976	0,651607	no
XLOC_001448	g11901	32,7379	33,8348	0,0475445	0,0794409	0,88345	0,947933	no
XLOC_001449	g11903	134,102	121,108	-0,147027	-0,25192	0,66935	0,839037	no
XLOC_001450	g11906	28,0838	25,9283	-0,115211	-0,17234	0,76325	0,888537	no
XLOC_001451	g11907	45,901	49,385	0,105545	0,169033	0,7604	0,886938	no
XLOC_001452	g11909	44,3278	77,9003	0,813418	1,1658	0,04015	0,17046	no
XLOC_001453	g11910	84,0709	169,403	1,01079	1,6227	0,00755	0,05496	no
XLOC_001454	g11914	49,6261	108,161	1,12401	1,81669	0,00235	0,0236687	yes
XLOC_001455	g11916	11,1528	41,8752	1,90869	2,59655	5,00E-05	0,00120049	yes
XLOC_001456	g11911	16,6987	13,1665	-0,342869	-0,529682	0,3505	0,611135	no
XLOC_001457	g11912	17,5364	38,8071	1,14597	1,68516	0,0033	0,0304282	yes
XLOC_001458	g11913	38,484	55,15	0,5191	0,814022	0,16435	0,406426	no
XLOC_001459	g11915	12,6816	9,07024	-0,483524	-0,664948	0,2534	0,512647	no
XLOC_001460	g11917	391,418	231,102	-0,760177	-1,2627	0,0243	0,12409	no
XLOC_001461	g6160	103,15	156,852	0,604663	1,04133	0,0666	0,235003	no
XLOC_001462	g6161	1,42616	0	#NAME?	0	1	1	no
XLOC_001463	g11918	12,5919	15,5216	0,301773	0,489527	0,3794	0,636522	no
XLOC_001464	g11919	34,001	66,9132	0,976714	1,67518	0,0031	0,0290477	yes
XLOC_001465	g11920	93,4716	143,956	0,62303	1,07048	0,05815	0,215973	no
XLOC_001466	g11921	47,0551	43,3607	-0,117962	-0,187451	0,7398	0,876968	no
XLOC_001467	g6162	3,85321	1,67867	-1,19874	-1,37257	0,02095	0,112666	no
XLOC_001468	g6163	20,9188	23,7877	0,185413	0,275574	0,63285	0,816463	no
XLOC_001469	g11924	45,2551	33,6468	-0,427609	-0,73674	0,1954	0,446863	no
XLOC_001470	g11922	281,788	335,41	0,251316	0,263689	0,64265	0,823115	no
XLOC_001471	g11923	383,671	1194,09	1,63797	2,10622	0,0008	0,0106019	yes

XLOC_001472	g11925	116,438	152,625	0,390432	0,606066	0,2928	0,555278	no
XLOC_001473	g6164	12,7569	18,0264	0,498829	0,58454	0,3253	0,585922	no
XLOC_001474	g6165	0,83225	1,2377	0,572567	0,535634	0,3423	0,602399	no
XLOC_001475	g6166	13,9454	22,6489	0,699654	0,810279	0,1582	0,398135	no
XLOC_001476	g6167	40,0515	44,5168	0,152493	0,195651	0,7318	0,873287	no
XLOC_001477	g6169	0	0,265544	inf	0	1	1	no
XLOC_001478	g6168	0	0	0	0	1	1	no
XLOC_001479	g6170	0	0	0	0	1	1	no
XLOC_001480	g6171	4,89046	6,7951	0,474525	0,517073	0,3735	0,631938	no
XLOC_001481	g6172	5,82754	11,7373	1,01014	1,10861	0,06495	0,231007	no
XLOC_001482	g6173	0	0	0	0	1	1	no
XLOC_001483	g6175	0	0	0	0	1	1	no
XLOC_001484	g6174	0	1,08463	inf	0	1	1	no
XLOC_001485	g11926	300,651	244,33	-0,299257	-0,438564	0,4492	0,694559	no
XLOC_001486	g11927	121,279	75,8705	-0,676722	-1,12375	0,05605	0,211697	no
XLOC_001487	g11928	27,0457	12,0665	-1,1644	-1,63757	0,0029	0,0276485	yes
XLOC_001488	g11930	16,823	22,4192	0,414301	0,639507	0,25105	0,509889	no
XLOC_001489	g11929	22,1725	31,932	0,526234	0,855917	0,12865	0,354275	no
XLOC_001490	g11931	5,57773	11,7769	1,07821	1,19725	0,0443	0,181185	no
XLOC_001491	g11932	16,6203	33,5264	1,01235	1,51538	0,0085	0,0594939	no
XLOC_001492	g6176	8,65347	9,44306	0,125976	0,166072	0,7711	0,893264	no
XLOC_001493	g6177	3,26588	5,45159	0,739206	0,806142	0,16415	0,406341	no
XLOC_001494	g6178	3,28724	2,76573	-0,249216	-0,241473	0,67635	0,843308	no
XLOC_001495	g6179	0	0	0	0	1	1	no
XLOC_001496	g6180	0	0,17115	inf	0	1	1	no
XLOC_001497	g6181	63,1695	68,5381	0,117678	0,197808	0,725	0,869807	no
XLOC_001498	g6182	1,29228	1,22305	-0,0794352	-0,0509519	0,8795	0,945715	no
XLOC_001499	g161	37,2617	51,0495	0,454204	0,773838	0,1672	0,410619	no
XLOC_001500	g11933	73,7881	52,4345	-0,492872	-0,742128	0,18535	0,433572	no
XLOC_001501	g11934	7,94785	24,7057	1,63621	1,72114	0,0077	0,0555169	no
XLOC_001502	g1409	6,68228	2,86334	-1,22264	-1,60935	0,0067	0,0503783	no
XLOC_001503	g1410	0	0	0	0	1	1	no
XLOC_001504	g1411	0	0	0	0	1	1	no
XLOC_001505	g1412	0	0,191897	inf	0	1	1	no
XLOC_001506	g11935	73,1309	74,756	0,031709	0,0496713	0,92785	0,968021	no
XLOC_001507	g6183	6,8847	5,97134	-0,205341	-0,278836	0,61935	0,808526	no
XLOC_001508	g11936	166,271	103,076	-0,689826	-1,16722	0,0447	0,182342	no
XLOC_001509	g6184	18,338	24,1422	0,396724	0,612782	0,27305	0,53424	no
XLOC_001510	g1414	398,192	261,773	-0,605145	-0,974544	0,0854	0,276208	no
XLOC_001511	g1413	28,1617	24,4301	-0,205077	-0,30401	0,58315	0,784861	no
XLOC_001512	g1415	6,95073	6,65873	-0,0619182	-0,0826411	0,8859	0,949006	no
XLOC_001513	g6185	67,7764	306,259	2,1759	3,46931	5,00E-05	0,00120049	yes
XLOC_001514	g6186	56,0047	88,4416	0,659176	1,11603	0,05255	0,203246	no
XLOC_001515	g6187	120,635	275,098	1,1893	1,88212	0,00105	0,0130684	yes
XLOC_001516	g11938	8,22613	10,4793	0,349255	0,482337	0,39725	0,651251	no
XLOC_001517	g11937	11,818	7,23659	-0,707608	-1,11199	0,04605	0,185789	no
XLOC_001518	g1417	0	0	0	0	1	1	no
XLOC_001519	g1418	0	0	0	0	1	1	no
XLOC_001520	g1416	0	0,122418	inf	0	1	1	no
XLOC_001521	g6188	0	0	0	0	1	1	no
XLOC_001522	g11939	25,0745	15,3752	-0,705613	-0,938657	0,24925	0,508092	no
XLOC_001523	g6189	57,0869	40,0863	-0,51005	-0,737425	0,1943	0,445592	no
XLOC_001524	g6190	0	0	0	0	1	1	no
XLOC_001525	g6191	12,9492	9,50719	-0,445768	-0,630389	0,2466	0,505345	no
XLOC_001526	g11941	40,7345	62,0299	0,606713	0,971184	0,0825	0,270501	no
XLOC_001527	g11940	85,9492	73,934	-0,217246	-0,364791	0,5188	0,743522	no
XLOC_001528	g11942	0,255115	0,247233	-0,0452802	0	1	1	no
XLOC_001529	g11943	35,5188	40,5308	0,190437	0,319293	0,57265	0,779083	no
XLOC_001530	g11944	184,2	329,729	0,840007	1,24582	0,0349	0,156065	no
XLOC_001531	g6192	10,9296	6,29025	-0,797048	-1,0093	0,07715	0,257779	no
XLOC_001532	g6193	1,60371	1,18743	-0,433577	-0,460906	0,435	0,681944	no
XLOC_001533	g11945	28,2099	25,8205	-0,127685	-0,194411	0,7349	0,87486	no
XLOC_001534	g1419	194,352	338,04	0,79852	0,939928	0,1221	0,344943	no
XLOC_001535	g1420	56,7099	75,1868	0,406879	0,700836	0,2203	0,477349	no
XLOC_001536	g1423	8,79759	15,3582	0,803824	1,30201	0,0255	0,128087	no
XLOC_001537	g1424	44,2871	48,5593	0,132862	0,224719	0,69625	0,855717	no
XLOC_001538	g1421	11,2775	19,5318	0,792374	1,14624	0,0545	0,207922	no
XLOC_001539	g1422	36,9229	58,2527	0,65781	1,09537	0,06135	0,223298	no
XLOC_001540	g1425	11,1516	13,3978	0,26475	0,436897	0,44385	0,690199	no
XLOC_001541	g11947	9,58944	20,5957	1,10282	1,46948	0,0093	0,0632452	no
XLOC_001542	g11949	64,144	59,2088	-0,115504	-0,200076	0,72465	0,869722	no
XLOC_001543	g11951	80,1517	85,9379	0,100563	0,147805	0,79315	0,904567	no
XLOC_001544	g11946	9,58067	16,091	0,748059	1,20749	0,03025	0,142404	no
XLOC_001545	g11948	100,496	121,174	0,269938	0,466049	0,41385	0,666012	no
XLOC_001546	g11950	245,027	233,297	-0,0707707	-0,120124	0,8309	0,922283	no
XLOC_001547	g11952	33,7122	36,51	0,115021	0,1868	0,7379	0,876306	no
XLOC_001548	g6194	164,898	169,893	0,0430526	0,0719127	0,90315	0,957458	no
XLOC_001549	g6195	0	0	0	0	1	1	no
XLOC_001550	g11953	7,79271	19,8242	1,34706	2,15395	0,00025	0,00430702	yes
XLOC_001551	g11954	46,4115	73,3825	0,660953	1,04957	0,0563	0,212477	no
XLOC_001552	g6197	106,654	113,775	0,0932445	0,144291	0,79985	0,907885	no
XLOC_001553	g6196	39,7639	83,6795	1,07342	1,64591	0,0066	0,0498938	yes
XLOC_001554	g11955	75,4371	87,6604	0,216651	0,360493	0,5218	0,74532	no
XLOC_001555	g6198	56,8737	332,129	2,54591	3,34965	5,00E-05	0,00120049	yes
XLOC_001556	g6200	0	2,15248	inf	0	1	1	no
XLOC_001557	g6199	5,40372	8,37588	0,632288	0,831865	0,1429	0,378099	no
XLOC_001558	g6201	10,5206	22,325	1,08545	1,27889	0,03065	0,143257	no
XLOC_001559	g6202	17,7857	14,7092	-0,274	-0,361729	0,53395	0,753431	no
XLOC_001560	g11956	0,321129	1,11771	1,79932	0	1	1	no
XLOC_001561	g11957	37,915	59,1694	0,642084	1,01004	0,0721	0,24756	no
XLOC_001562	g6203	20,0313	24,6605	0,299948	0,396875	0,4809	0,715629	no
XLOC_001563	g6208	52,8323	81,5851	0,626885	1,05692	0,07155	0,246447	no

XLOC_001564	g6210	28,4632	7,52278	-1,91976	-2,81748	5,00E-05	0,00120049	yes
XLOC_001565	g6204	44,7576	29,6928	-0,592018	-0,730305	0,2126	0,468106	no
XLOC_001566	g6205	29,5461	18,9064	-0,644097	-1,02335	0,07355	0,250691	no
XLOC_001567	g6206	17,3349	16,1986	-0,0978054	-0,140595	0,80015	0,908063	no
XLOC_001568	g6207	15,9636	20,0559	0,329238	0,50789	0,3782	0,636208	no
XLOC_001569	g6209	1563,89	2177,31	0,477406	0,647152	0,268	0,528997	no
XLOC_001570	g6211	1001,6	753,329	-0,410954	-0,669826	0,2431	0,50173	no
XLOC_001571	g6212	19,5879	7,588	-1,36817	-2,0571	0,00025	0,00430702	yes
XLOC_001572	g6213	0	0	0	0	1	1	no
XLOC_001573	g6214	46,8223	19,2789	-1,28017	-1,17507	0,05505	0,208964	no
XLOC_001574	g6215	9,81029	19,2807	0,974789	1,24159	0,03405	0,153311	no
XLOC_001575	g11958	232,565	208,458	-0,157881	-0,254046	0,6591	0,832458	no
XLOC_001576	g6216	0	0	0	0	1	1	no
XLOC_001577	g6217	0	0	0	0	1	1	no
XLOC_001578	g6218	0	0	0	0	1	1	no
XLOC_001579	g11959	6,03723	3,20817	-0,912135	-1,13359	0,0582	0,215995	no
XLOC_001580	g6219	0	0,0148763	inf	0	1	1	no
XLOC_001581	g6220	30,5698	28,8902	-0,0815272	-0,119826	0,84015	0,926581	no
XLOC_001582	g6221	124,942	87,0343	-0,521602	-0,691702	0,2242	0,481866	no
XLOC_001583	g6222	14,8457	5,79759	-1,35652	-1,93653	0,0015	0,0168343	yes
XLOC_001584	g6224	120,209	94,2412	-0,351121	-0,5087	0,37215	0,630523	no
XLOC_001585	g6223	210,303	214,934	0,0314185	0,0541002	0,9178	0,964054	no
XLOC_001586	g6225	290,963	452,339	0,636569	0,964003	0,0955	0,295373	no
XLOC_001587	g6226	1,67766	0	#NAME?	0	1	1	no
XLOC_001588	g11960	4,68609	7,34751	0,64887	0,593892	0,31575	0,576546	no
XLOC_001589	g6227	0	0	0	0	1	1	no
XLOC_001590	g6228	64,5501	39,04	-0,725466	-1,20241	0,033	0,150446	no
XLOC_001591	g6229	25,0786	39,1484	0,642493	1,00179	0,07295	0,249343	no
XLOC_001592	g6230	229,495	315,799	0,46054	0,700054	0,21905	0,476217	no
XLOC_001593	g6231	509,831	262,961	-0,955168	-1,3288	0,028	0,135891	no
XLOC_001594	g6232	23,829	17,1385	-0,475479	-0,628392	0,2608	0,521008	no
XLOC_001595	g6233	0	0	0	0	1	1	no
XLOC_001596	g6234	0	0	0	0	1	1	no
XLOC_001597	g11961	39,479	36,405	-0,116951	-0,197971	0,7358	0,874871	no
XLOC_001598	g6235	19,6498	12,7124	-0,628274	-0,796577	0,1584	0,398434	no
XLOC_001599	g1426	0	0	0	0	1	1	no
XLOC_001600	g6236	81,6697	56,095	-0,541929	-0,658797	0,24255	0,500911	no
XLOC_001601	g6237	0	0	0	0	1	1	no
XLOC_001602	g6238	0	0	0	0	1	1	no
XLOC_001603	g6239	0	0	0	0	1	1	no
XLOC_001604	g6240	0	1,19344	inf	0	1	1	no
XLOC_001605	g6241	1788,09	2785,19	0,639358	1,02864	0,07445	0,252007	no
XLOC_001606	g6242	3,60871	6,62412	0,876245	0,972898	0,099	0,301919	no
XLOC_001607	g6243	44,7468	77,2587	0,787912	1,30262	0,02365	0,122556	no
XLOC_001608	g1427	0	0,0927663	inf	0	1	1	no
XLOC_001609	g6244	0	0	0	0	1	1	no
XLOC_001610	g6246	979,7	912,224	-0,102952	-0,173006	0,76525	0,890163	no
XLOC_001611	g6245	216,211	142,916	-0,597272	-1,01102	0,07985	0,264372	no
XLOC_001612	g6247	11,312	8,76001	-0,368846	-0,560054	0,33765	0,597535	no
XLOC_001613	g11962	37,1925	43,1899	0,215682	0,323255	0,5798	0,782953	no
XLOC_001614	g6248	39,9214	36,6355	-0,12392	-0,184336	0,7418	0,877648	no
XLOC_001615	g6249	18,7821	18,2823	-0,0389153	-0,0546946	0,9205	0,96497	no
XLOC_001616	g6250	24,6421	25,7099	0,0612004	0,102723	0,85735	0,935212	no
XLOC_001617	g6253	116,244	88,4217	-0,39468	-0,680856	0,2398	0,497851	no
XLOC_001618	g6251	300,347	36,0318	-3,05929	-4,58446	5,00E-05	0,00120049	yes
XLOC_001619	g6252	160,336	218,429	0,446061	0,748582	0,18	0,426853	no
XLOC_001620	g6254	91,301	98,9832	0,116553	0,193681	0,74315	0,878716	no
XLOC_001621	g11963	60,3377	77,9064	0,368682	0,55539	0,32075	0,580709	no
XLOC_001622	g11964	26,0187	37,2649	0,518269	0,705282	0,21005	0,464989	no
XLOC_001623	g11965	0	0	0	0	1	1	no
XLOC_001624	g6255	5,87801	7,87302	0,42159	0,655415	0,25785	0,517915	no
XLOC_001625	g1428	0	0	0	0	1	1	no
XLOC_001626	g1429	10,9715	13,4975	0,298922	0,418512	0,4601	0,701766	no
XLOC_001627	g11967	78,6017	41,6206	-0,917265	-1,5054	0,00805	0,0571591	no
XLOC_001628	g11968	77,1766	64,7783	-0,252653	-0,426608	0,45785	0,701013	no
XLOC_001629	g11970	32,3652	91,258	1,49551	2,38469	5,00E-05	0,00120049	yes
XLOC_001630	g11971	40,9002	77,9261	0,929997	1,54355	0,00525	0,0423624	yes
XLOC_001631	g11966	48,1837	43,3833	-0,151406	-0,248579	0,67335	0,841366	no
XLOC_001632	g11969	50,3275	75,0236	0,575999	0,974921	0,0928	0,290499	no
XLOC_001633	g1430	81,5082	79,2574	-0,0403989	-0,060112	0,9166	0,963809	no
XLOC_001634	g1431	109,921	73,8116	-0,574548	-0,972969	0,0829	0,271381	no
XLOC_001635	g1432	77,7609	43,7964	-0,828233	-1,17443	0,0366	0,16038	no
XLOC_001636	g6256	402,863	201,096	-1,00241	-1,48489	0,0106	0,0692562	no
XLOC_001637	g6257	34,0368	30,2537	-0,169983	-0,27231	0,63305	0,816463	no
XLOC_001638	g11972	29,6981	36,5754	0,300505	0,492255	0,38795	0,643448	no
XLOC_001639	g11973	17,6961	13,0372	-0,440803	-0,391548	0,4897	0,722376	no
XLOC_001640	g1433	0	0	0	0	1	1	no
XLOC_001641	g6258	0	0	0	0	1	1	no
XLOC_001642	g11974	34,2375	22,5866	-0,600109	-0,73594	0,2027	0,455287	no
XLOC_001643	g11975	24,9668	20,7549	-0,266558	-0,389917	0,49565	0,725992	no
XLOC_001644	g11976	27,5732	42,4362	0,622028	0,857923	0,1368	0,368958	no
XLOC_001645	g1434	0,179347	0,331308	0,885421	0	1	1	no
XLOC_001646	g11977	4,77072	9,06934	0,92679	0,760017	0,19855	0,451438	no
XLOC_001647	g11978	0	0	0	0	1	1	no
XLOC_001648	g1435	117,79	127,474	0,113982	0,176865	0,7562	0,884666	no
XLOC_001649	g11979	43,608	51,2054	0,231703	0,331386	0,54495	0,761892	no
XLOC_001650	g11980	0,465255	0,620108	0,414499	0	1	1	no
XLOC_001651	g11981	50,5501	28,62	-0,820691	-0,91568	0,1193	0,339474	no
XLOC_001652	g6259	0	2,0217	inf	0	1	1	no
XLOC_001653	g6260	0	0	0	0	1	1	no
XLOC_001654	g1436	98,183	112,19	0,192401	0,303131	0,58855	0,789071	no
XLOC_001655	g1437	46,4764	74,0181	0,67138	1,15559	0,044	0,18056	no

XLOC_001656	g6261	0	0	0	0	1	1	no
XLOC_001657	g6262	0	0	0	0	1	1	no
XLOC_001658	g6263	10,8427	7,10869	-0,609071	-0,744831	0,2013	0,453491	no
XLOC_001659	g6264	1,34115	3,79953	1,50235	1,25061	0,0417	0,174624	no
XLOC_001660	g6266	269,144	389,093	0,531733	0,788375	0,1643	0,406405	no
XLOC_001661	g6267	4,7035	3,32633	-0,499804	-0,555194	0,34535	0,605488	no
XLOC_001662	g6265	13,2396	10,4106	-0,346804	-0,420078	0,46325	0,704415	no
XLOC_001663	g6268	27,6411	90,4953	1,71103	2,42364	5,00E-05	0,00120049	yes
XLOC_001664	g1439	0,204828	0,413479	1,0134	0	1	1	no
XLOC_001665	g1441	12,6445	15,2067	0,266193	0,448319	0,44635	0,692442	no
XLOC_001666	g1442	252,548	345,476	0,452029	0,74069	0,18945	0,439291	no
XLOC_001667	g1438	8,13516	47,5003	2,54569	2,07476	0,0045	0,038062	yes
XLOC_001668	g1440	15,8715	7,31899	-1,11672	-1,48959	0,00945	0,0639552	no
XLOC_001669	g1443	228,692	195,156	-0,228779	-0,393285	0,48565	0,719067	no
XLOC_001670	g6269	184,646	200,427	0,118321	0,197809	0,73535	0,874871	no
XLOC_001671	g6270	10,095	27,9743	1,47046	1,92757	0,0007	0,00954722	yes
XLOC_001672	g1444	0	0	0	0	1	1	no
XLOC_001673	g6271	55,5936	30,5336	-0,864521	-1,17821	0,0419	0,175163	no
XLOC_001674	g6272	5,61407	5,84369	0,0578326	0,0624181	0,91245	0,962641	no
XLOC_001675	g6273	17,1829	9,39528	-0,870968	-0,841003	0,15135	0,388869	no
XLOC_001676	g6274	0	0	0	0	1	1	no
XLOC_001677	g6275	7,22696	20,2771	1,48839	2,03069	0,00085	0,011085	yes
XLOC_001678	g6277	98,2725	120,899	0,298944	0,495689	0,3918	0,646417	no
XLOC_001679	g6276	10,8652	29,4843	1,44023	2,14262	0,00035	0,00552572	yes
XLOC_001680	g6278	206,679	332,868	0,687557	1,07415	0,05335	0,205128	no
XLOC_001681	g1445	4,72261	4,27848	-0,142486	-0,167216	0,77615	0,895628	no
XLOC_001682	g6280	0	0	0	0	1	1	no
XLOC_001683	g6279	0	0	0	0	1	1	no
XLOC_001684	g6281	69,4099	93,9213	0,436312	0,675354	0,23495	0,492678	no
XLOC_001685	g6282	0,0883894	0,0910952	0,0435026	0	1	1	no
XLOC_001686	g1446	51,5696	62,477	0,276802	0,429834	0,43755	0,68432	no
XLOC_001687	g1448	81,7781	81,92	0,00250138	0,00426826	0,99395	0,996589	no
XLOC_001688	g1450	48,4272	57,8664	0,256908	0,438071	0,43665	0,683876	no
XLOC_001689	g1447	38,7566	48,4617	0,322406	0,467955	0,40505	0,658433	no
XLOC_001690	g1449	34,3471	39,7916	0,212278	0,338601	0,5304	0,750941	no
XLOC_001691	g1451	5,76864	11,838	1,03712	1,45887	0,01405	0,0848009	no
XLOC_001692	g6283	81,2909	214,42	1,39927	2,36801	5,00E-05	0,00120049	yes
XLOC_001693	g1452	0	0	0	0	1	1	no
XLOC_001694	g1453	0	0	0	0	1	1	no
XLOC_001695	g6284	0	0	0	0	1	1	no
XLOC_001696	g6285	10,9045	11,9426	0,131195	0,150734	0,7896	0,902873	no
XLOC_001697	g6286	0	0	0	0	1	1	no
XLOC_001698	g1454	146,876	103,498	-0,505002	-0,822605	0,14565	0,381918	no
XLOC_001699	g1458	7,07305	5,44142	-0,378349	-0,550098	0,34305	0,603374	no
XLOC_001700	g1459	633,618	867,229	0,4528	0,660629	0,22515	0,483378	no
XLOC_001701	g1455	98,3947	96,6242	-0,0261975	-0,0448944	0,9363	0,972153	no
XLOC_001702	g1456	44,1758	43,9268	-0,008155	-0,0128134	0,9816	0,990985	no
XLOC_001703	g1457	41,1542	22,1446	-0,894084	-1,55155	0,00835	0,0587371	no
XLOC_001704	g1460	24,8041	11,2253	-1,14383	-1,71277	0,00555	0,0439524	yes
XLOC_001705	g1461	138,567	176,93	0,352598	0,514508	0,36175	0,621784	no
XLOC_001706	g6287	134,266	93,9833	-0,514613	-0,860447	0,13865	0,371397	no
XLOC_001707	g6288	13,4418	7,12817	-0,915128	-1,27603	0,03055	0,143091	no
XLOC_001708	g6289	116,228	165,98	0,514051	0,855934	0,12855	0,354275	no
XLOC_001709	g6290	19,7207	6,01087	-1,71407	-2,21493	0,0004	0,0061761	yes
XLOC_001710	g6291	0	0	0	0	1	1	no
XLOC_001711	g6292	0	0	0	0	1	1	no
XLOC_001712	g6293	14,4716	9,14445	-0,662254	-0,911489	0,1133	0,329709	no
XLOC_001713	g6294	287,553	288,127	0,00287745	0,00492867	0,99275	0,995792	no
XLOC_001714	g1462	61,3321	50,8441	-0,27056	-0,438557	0,43105	0,678624	no
XLOC_001715	g1463	4,62373	12,2582	1,40661	2,03415	0,0006	0,00845337	yes
XLOC_001716	g1466	60,3804	75,8573	0,329209	0,542355	0,33675	0,597018	no
XLOC_001717	g1464	7,66184	2,84071	-1,43144	-1,82624	0,00275	0,0265797	yes
XLOC_001718	g1465	16,2127	45,1336	1,47708	2,3287	5,00E-05	0,00120049	yes
XLOC_001719	g6296	113,647	94,272	-0,269652	-0,40921	0,45495	0,699375	no
XLOC_001720	g6295	28,2602	31,9883	0,17877	0,269026	0,61805	0,808291	no
XLOC_001721	g6297	11,9068	10,6304	-0,163599	-0,134627	0,8126	0,914478	no
XLOC_001722	g6298	118,19	432,532	1,8717	2,76612	5,00E-05	0,00120049	yes
XLOC_001723	g6300	47,5882	40,6518	-0,227284	-0,391314	0,4884	0,721433	no
XLOC_001724	g6301	33,3774	29,4208	-0,182035	-0,269623	0,632	0,816117	no
XLOC_001725	g6299	31,6479	28,629	-0,14463	-0,236601	0,6722	0,840955	no
XLOC_001726	g6302	22,6247	25,702	0,183979	0,290829	0,6142	0,805977	no
XLOC_001727	g6303	453,598	1484,18	1,71019	2,73331	5,00E-05	0,00120049	yes
XLOC_001728	g6304	0	0	0	0	1	1	no
XLOC_001729	g6305	54,3436	59,065	0,120195	0,205523	0,7169	0,865633	no
XLOC_001730	g6306	14,2468	38,6657	1,44042	1,54519	0,01165	0,0741432	no
XLOC_001731	g1468	82,6117	75,3036	-0,133626	-0,224152	0,6838	0,84795	no
XLOC_001732	g1467	53,7351	62,5077	0,218169	0,360045	0,5209	0,744468	no
XLOC_001733	g1469	101,262	193,686	0,935635	1,42754	0,01465	0,0872426	no
XLOC_001734	g1471	43,7599	81,0361	0,888956	1,45277	0,0111	0,0715706	no
XLOC_001735	g1474	41,5169	74,8974	0,851218	1,0704	0,07665	0,256537	no
XLOC_001736	g1477	154,73	214,774	0,473072	0,81658	0,1561	0,393961	no
XLOC_001737	g1470	128,992	20,4797	-2,65502	-4,28008	5,00E-05	0,00120049	yes
XLOC_001738	g1472	27,5151	52,2811	0,926066	1,38318	0,01555	0,0907314	no
XLOC_001739	g1473	43,4415	11,0438	-1,97584	-2,51624	0,0001	0,00209829	yes
XLOC_001740	g1475	25,8259	50,268	0,960821	1,43605	0,0107	0,0697703	no
XLOC_001741	g1476	12,4408	23,8748	0,940405	1,4824	0,00895	0,0617198	no
XLOC_001742	g1478	18,9255	28,919	0,611688	0,972091	0,09635	0,296973	no
XLOC_001743	g6307	0	0	0	0	1	1	no
XLOC_001744	g6308	0	0	0	0	1	1	no
XLOC_001745	g6309	0	0	0	0	1	1	no
XLOC_001746	g1480	107,954	128,352	0,249698	0,395398	0,47655	0,712937	no
XLOC_001747	g1483	76,2531	180,412	1,24242	2,07332	0,0005	0,00733931	yes

XLOC_001748	g1485	125,333	249,647		0,994129	1,66477	0,00395	0,0345713	yes
XLOC_001749	g1487	97,5633	157,151		0,687742	1,14429	0,0426	0,177109	no
XLOC_001750	g1479	42,0725	31,1548		-0,433425	-0,63553	0,27015	0,531531	no
XLOC_001751	g1481	88,0593	87,5853		-0,00778789	-0,0112894	0,9857	0,992622	no
XLOC_001752	g1482	95,8581	95,4793		-0,00571182	-0,00824724	0,9884	0,994069	no
XLOC_001753	g1484	36,642	55,8503		0,608069	0,959022	0,0884	0,281458	no
XLOC_001754	g1486	156,816	108,76		-0,527924	-0,89153	0,12275	0,346082	no
XLOC_001755	g1488	53,7964	43,9118		-0,292901	-0,444488	0,44325	0,689375	no
XLOC_001756	g1489	699,095	637,786		-0,132417	-0,215791	0,71035	0,862876	no
XLOC_001757	g6310	9,3538	2,18236		-2,09966	-2,28369	0,00085	0,011085	yes
XLOC_001758	g6311	11,8172	14,2864		0,273752	0,374797	0,51225	0,738446	no
XLOC_001759	g6312	7,59427	5,55909		-0,450062	-0,521459	0,37645	0,634524	no
XLOC_001760	g6313	17,0072	12,2091		-0,478187	-0,57047	0,3249	0,585506	no
XLOC_001761	g6314	16,434	12,4859		-0,39638	-0,59635	0,29545	0,557303	no
XLOC_001762	g6315	464,331	358,653		-0,372564	-0,615908	0,28465	0,546579	no
XLOC_001763	g1491	4,8622	8,61598		0,825406	1,09473	0,06005	0,220445	no
XLOC_001764	g1494	17,3393	19,2123		0,147987	0,197415	0,7383	0,876358	no
XLOC_001765	g1490	32,5369	29,1869		-0,156753	-0,254556	0,6496	0,827591	no
XLOC_001766	g1492	5,83127	4,56898		-0,351937	-0,481296	0,3874	0,643156	no
XLOC_001767	g1493	38,396	21,4403		-0,84063	-1,28218	0,02065	0,111542	no
XLOC_001768	g1495	33,7298	53,3778		0,662216	0,872111	0,14025	0,373746	no
XLOC_001769	g1496	21,0715	25,7324		0,288291	0,449397	0,4336	0,68051	no
XLOC_001770	g6316	0,197895	0,174549		-0,181101	0	1	1	no
XLOC_001771	g6319	52,1792	57,8098		0,147839	0,240577	0,6732	0,841348	no
XLOC_001772	g6325	24,4189	20,7705		-0,233462	-0,362643	0,51255	0,73877	no
XLOC_001773	g6327	31,6055	37,3556		0,241146	0,394038	0,48995	0,722384	no
XLOC_001774	g6317	29,4414	25,7372		-0,193989	-0,314509	0,5712	0,778297	no
XLOC_001775	g6318	19,6278	10,7465		-0,869027	-1,22061	0,0387	0,166386	no
XLOC_001776	g6320	185,639	103,055		-0,84909	-1,2851	0,0258	0,129197	no
XLOC_001777	g6321	6,88068	5,51667		-0,318753	-0,363055	0,5163	0,741989	no
XLOC_001778	g6322	22,4807	32,2451		0,520395	0,845301	0,12955	0,356154	no
XLOC_001779	g6323	21,4806	15,7291		-0,449594	-0,754094	0,191	0,441334	no
XLOC_001780	g6324	46,5	37,9999		-0,291237	-0,496412	0,3709	0,6296	no
XLOC_001781	g6326	23,2185	29,2716		0,334227	0,54442	0,33585	0,595745	no
XLOC_001782	g6328	291,81	190,438		-0,615708	-1,04584	0,07	0,242726	no
XLOC_001783	g6329	48,4458	48,2266		-0,00654374	-0,0101946	0,98525	0,992425	no
XLOC_001784	g1497	410,565	223,257		-0,878903	-0,951676	0,10785	0,319291	no
XLOC_001785	g6330	0	0		0	0	1	1	no
XLOC_001786	g6331	0	0		0	0	1	1	no
XLOC_001787	g6332	0	0		0	0	1	1	no
XLOC_001788	g1498	116,092	84,2998		-0,461664	-0,767445	0,16995	0,413711	no
XLOC_001789	g6333	180,038	99,5972		-0,854121	-1,18191	0,03715	0,162067	no
XLOC_001790	g6334	0,117199	0,704392		2,58742	0	1	1	no
XLOC_001791	g6335	0	0,635168	inf		0	1	1	no
XLOC_001792	g6336	193,048	329,625		0,771866	1,15915	0,05105	0,19917	no
XLOC_001793	g6338	19,3439	9,32486		-1,05273	-1,59582	0,00905	0,0621476	no
XLOC_001794	g6337	16727,2	5442,62		-1,61982	-1,00271	0,10425	0,31221	no
XLOC_001795	g1499	26,6616	45,8552		0,782321	1,27346	0,0275	0,13442	no
XLOC_001796	g6339	0	0		0	0	1	1	no
XLOC_001797	g6340	0	0		0	0	1	1	no
XLOC_001798	g6341	45,2896	55,7287		0,299242	0,512865	0,367	0,626554	no
XLOC_001799	g6342	50,394	62,6233		0,313448	0,46943	0,4202	0,671172	no
XLOC_001800	g6343	0	0		0	0	1	1	no
XLOC_001801	g6344	35,7395	36,9099		0,046488	0,0778614	0,894	0,952901	no
XLOC_001802	g6345	56,5634	47,769		-0,243795	-0,403035	0,47235	0,709899	no
XLOC_001803	g6346	63,5396	39,4156		-0,688888	-1,14837	0,0502	0,197423	no
XLOC_001804	g6347	7,51789	5,77047		-0,38164	-0,407893	0,4826	0,716713	no
XLOC_001805	g6348	0,0597193	0		#NAME?	0	1	1	no
XLOC_001806	g6349	89,2057	26,5712		-1,74727	-2,43291	0,00015	0,00294012	yes
XLOC_001807	g6350	0	0		0	0	1	1	no
XLOC_001808	g1500	11,7613	9,02606		-0,381878	-0,552063	0,33975	0,600023	no
XLOC_001809	g1501	43,0486	101,567		1,2384	2,00417	0,0003	0,00496796	yes
XLOC_001810	g6352	48,7029	68,2413		0,486639	0,802503	0,1685	0,411405	no
XLOC_001811	g6351	47,9371	72,8687		0,604156	0,784452	0,1872	0,435617	no
XLOC_001812	g1502	7,42328	7,22466		-0,039126	-0,0473313	0,93005	0,969131	no
XLOC_001813	g6353	20,5871	23,6758		0,201669	0,265011	0,64565	0,825343	no
XLOC_001814	g6354	0	0,0876394	inf		0	1	1	no
XLOC_001815	g6355	60,8224	116,485		0,937466	1,49985	0,0113	0,0724321	no
XLOC_001816	g6357	19,4306	22,7539		0,227781	0,390042	0,49305	0,724054	no
XLOC_001817	g6356	106,714	97,0879		-0,136388	-0,229818	0,68355	0,847854	no
XLOC_001818	g6358	133,391	133,74		0,00377008	0,00646573	0,99145	0,995293	no
XLOC_001819	g6359	0,941303	0		#NAME?	0	1	1	no
XLOC_001820	g6361	90,8127	132,953		0,549956	0,730774	0,1886	0,437837	no
XLOC_001821	g6360	474,144	107,926		-2,13528	-2,5472	5,00E-05	0,00120049	yes
XLOC_001822	g6362	11,6298	11,7863		0,0192806	0,0222235	0,97	0,986679	no
XLOC_001823	g6363	34,8456	41,5682		0,254505	0,334895	0,5667	0,775285	no
XLOC_001824	g6364	0	0		0	0	1	1	no
XLOC_001825	g6366	1,88497	1,06975		-0,817262	-0,884058	0,11825	0,337661	no
XLOC_001826	g6365	30,984	29,7521		-0,0585316	-0,0912338	0,8754	0,943833	no
XLOC_001827	g1503	0,268675	0,494079		0,878883	0	1	1	no
XLOC_001828	g1504	47,4042	79,1336		0,739276	1,26331	0,02755	0,134531	no
XLOC_001829	g1505	28,7659	14,3055		-1,0078	-1,32855	0,01845	0,102593	no
XLOC_001830	g6367	80,435	118,052		0,553533	0,81085	0,13905	0,372164	no
XLOC_001831	g6368	0	0		0	0	1	1	no
XLOC_001832	g1506	1,54396	2,72538		0,819824	0	1	1	no
XLOC_001833	g6370	30,0588	15,2502		-0,978961	-1,64482	0,0042	0,0360524	yes
XLOC_001834	g6369	13,9095	7,95343		-0,806419	-1,02089	0,08825	0,281186	no
XLOC_001835	g6371	75,2072	56,1923		-0,420499	-0,705685	0,2138	0,469375	no
XLOC_001836	g1508	29,2214	11,0855		-1,39835	-2,20659	0,00025	0,00430702	yes
XLOC_001837	g1509	87,0151	79,1839		-0,136059	-0,215656	0,71305	0,864143	no
XLOC_001838	g1510	10,1288	4,17481		-1,27868	-1,9801	0,00065	0,00901554	yes
XLOC_001839	g1511	728,147	14051,8		4,27038	4,96482	5,00E-05	0,00120049	yes

XLOC_001840	g1513	24,2993	26,7425	0,138219	0,233709	0,67485	0,842169	no
XLOC_001841	g1507	44,1989	35,9179	-0,299308	-0,513723	0,36765	0,627446	no
XLOC_001842	g1512	1,36419	8,91907	2,70885	2,50049	0,2275	0,485874	no
XLOC_001843	g6372	3,18859	9,16373	1,52301	1,23941	0,07835	0,260901	no
XLOC_001844	g162	1982,23	4456,34	1,16873	1,79919	0,0019	0,0201056	yes
XLOC_001845	g6373	2,64402	0,902708	-1,5504	-1,90145	0,00205	0,0212577	yes
XLOC_001846	g6374	49,0704	63,0974	0,362728	0,48479	0,385	0,640945	no
XLOC_001847	g6375	8,38615	11,1477	0,410672	0,587431	0,3023	0,564048	no
XLOC_001848	g6376	11,2402	26,3527	1,22929	1,85978	0,00145	0,0164423	yes
XLOC_001849	g6377	21,7644	21,0279	-0,0496653	-0,0821317	0,88925	0,950678	no
XLOC_001850	g6378	563,056	265,952	-1,08211	-1,62687	0,00415	0,0357482	yes
XLOC_001851	g1514	0	0	0	0	1	1	no
XLOC_001852	g6379	0	0	0	0	1	1	no
XLOC_001853	g6380	72,1643	50,8857	-0,504024	-0,742455	0,1831	0,43036	no
XLOC_001854	g6381	13,1944	23,3444	0,823151	1,15119	0,0498	0,196242	no
XLOC_001855	g6382	86,479	22,2354	-1,95949	-2,59071	0,0442	0,180927	no
XLOC_001856	g6383	7,7012	7,91099	0,0387741	0,0544726	0,92635	0,967431	no
XLOC_001857	g6384	11,7035	8,4082	-0,477067	-0,654238	0,25255	0,511771	no
XLOC_001858	g6385	31,4557	27,35	-0,201783	-0,347389	0,5371	0,75585	no
XLOC_001859	g6386	0	0	0	0	1	1	no
XLOC_001860	g6388	11,2145	38,9724	1,79709	2,44986	5,00E-05	0,00120049	yes
XLOC_001861	g6387	59,8366	63,4915	0,0855361	0,122349	0,82645	0,920883	no
XLOC_001862	g1516	43,2678	59,9219	0,469791	0,718939	0,21195	0,467509	no
XLOC_001863	g1515	74,7317	157,629	1,07674	1,80323	0,00265	0,0257909	yes
XLOC_001864	g6389	125,544	134,343	0,0977253	0,142904	0,80115	0,908537	no
XLOC_001865	g6390	4,23032	3,44164	-0,297672	-0,382205	0,49475	0,725323	no
XLOC_001866	g6391	3,56346	2,31448	-0,622588	-0,605417	0,28335	0,546123	no
XLOC_001867	g6392	0	0,0616887	inf	0	1	1	no
XLOC_001868	g6393	261,652	830,821	1,66689	1,56895	0,0031	0,0290477	yes
XLOC_001869	g6394	9,91991	24,114	1,28147	1,54438	0,01085	0,070421	no
XLOC_001870	g6395	6,33916	11,3148	0,835843	1,31729	0,0244	0,124472	no
XLOC_001871	g6396	94,7886	78,9043	-0,264611	-0,380829	0,49245	0,723714	no
XLOC_001872	g6397	52,8255	49,8387	-0,0839685	-0,129028	0,8282	0,921598	no
XLOC_001873	g6398	23,7871	24,147	0,0216676	0,0369596	0,9445	0,975496	no
XLOC_001874	g6399	19,7586	15,3103	-0,367983	-0,501998	0,36675	0,626431	no
XLOC_001875	g1517	32,8625	24,4584	-0,426112	-0,710227	0,2034	0,456337	no
XLOC_001876	g163	77,4603	88,7958	0,197034	0,335849	0,54415	0,761477	no
XLOC_001877	g164	65,6005	61,0867	-0,10285	-0,162801	0,77135	0,893401	no
XLOC_001878	g6400	30,9076	22,4207	-0,46313	-0,55533	0,3248	0,585451	no
XLOC_001879	g6402	72,1354	147,666	1,03356	1,7501	0,00165	0,0180837	yes
XLOC_001880	g6401	124,761	133,58	0,0985387	0,168952	0,7643	0,889268	no
XLOC_001881	g6403	36,0066	26,6321	-0,435092	-0,710589	0,20795	0,46253	no
XLOC_001882	g6404	47,8778	108,561	1,18108	1,65651	0,00585	0,0457017	yes
XLOC_001883	g6405	73,8641	83,358	0,174446	0,242831	0,6721	0,840955	no
XLOC_001884	g1522	43,3901	42,2568	-0,0381821	-0,0642539	0,91125	0,962529	no
XLOC_001885	g1518	5,53269	2,51737	-1,13606	-1,39327	0,0149	0,0881965	no
XLOC_001886	g1519	106,058	76,4494	-0,472276	-0,642904	0,2339	0,491315	no
XLOC_001887	g1520	5,27058	9,89466	0,90869	1,23581	0,03785	0,164245	no
XLOC_001888	g1521	6,6221	2,05366	-1,68909	-2,06092	0,0007	0,00954722	yes
XLOC_001889	g6406	86,9312	4,46806	-4,28215	-4,27504	5,00E-05	0,00120049	yes
XLOC_001890	g6407	105,048	207,699	0,983445	1,24668	0,0306	0,143091	no
XLOC_001891	g6408	44,6569	26,3706	-0,759955	-1,27661	0,02705	0,133148	no
XLOC_001892	g6409	86,3159	154,72	0,84196	1,39471	0,0144	0,086277	no
XLOC_001893	g6410	41,2375	58,8914	0,514102	0,765343	0,17385	0,419771	no
XLOC_001894	g6411	24,8293	23,7724	-0,0627525	-0,105251	0,8541	0,934519	no
XLOC_001895	g6412	586,484	847,047	0,530349	0,728612	0,1933	0,444129	no
XLOC_001896	g6414	60,7414	37,9488	-0,678626	-1,13109	0,04665	0,18744	no
XLOC_001897	g6413	67,473	94,4879	0,485818	0,82971	0,13795	0,370331	no
XLOC_001898	g6419	102,01	121,423	0,251326	0,438662	0,4405	0,686808	no
XLOC_001899	g6415	74,7603	115,247	0,624382	1,02538	0,06995	0,242638	no
XLOC_001900	g6416	104,433	156,57	0,584226	0,956826	0,0902	0,284995	no
XLOC_001901	g6417	4,76876	5,73955	0,267323	0,300403	0,6193	0,808526	no
XLOC_001902	g6418	54,2778	51,9281	-0,0638447	-0,106297	0,8453	0,929972	no
XLOC_001903	g6421	58,588	81,6778	0,479338	0,757111	0,18625	0,434333	no
XLOC_001904	g6420	10,1199	6,08366	-0,734185	-1,09982	0,0552	0,20921	no
XLOC_001905	g6422	1,80958	7,73365	2,0955	2,45545	0,00015	0,00294012	yes
XLOC_001906	g6423	20,7805	28,1771	0,439292	0,753225	0,19225	0,442349	no
XLOC_001907	g6424	65,3655	18,3606	-1,83191	-2,7151	5,00E-05	0,00120049	yes
XLOC_001908	g6425	55,1577	89,783	0,702879	1,19074	0,03225	0,148058	no
XLOC_001909	g6427	2330,43	1401,97	-0,733144	-0,792126	0,1728	0,418264	no
XLOC_001910	g6426	14,4586	11,8686	-0,284783	-0,365877	0,50845	0,73491	no
XLOC_001911	g6428	92,4581	75,3115	-0,295929	-0,47957	0,4103	0,662907	no
XLOC_001912	g6429	149,414	206,478	0,466678	0,762307	0,1823	0,42989	no
XLOC_001913	g1525	257,518	52,7959	-2,28618	-3,19833	5,00E-05	0,00120049	yes
XLOC_001914	g1523	60,1923	71,8258	0,254922	0,352987	0,52785	0,74938	no
XLOC_001915	g1524	104,831	136,13	0,376915	0,64457	0,2578	0,517915	no
XLOC_001916	g1526	11,5559	11,4278	-0,0160849	-0,0234981	0,96955	0,986579	no
XLOC_001917	g1527	55,364	34,3052	-0,690524	-0,980483	0,0814	0,268148	no
XLOC_001918	g6430	8,24341	4,8144	-0,775885	-1,10209	0,05505	0,208964	no
XLOC_001919	g6431	0	0	0	0	1	1	no
XLOC_001920	g1528	13,5249	72,2842	2,41806	3,71282	5,00E-05	0,00120049	yes
XLOC_001921	g1529	63,8616	50,2101	-0,34697	-0,561077	0,31975	0,580289	no
XLOC_001922	g6433	105,595	101,326	-0,0595369	-0,093755	0,87315	0,942649	no
XLOC_001923	g6432	571,648	370,325	-0,626333	-0,711618	0,21185	0,467393	no
XLOC_001924	g6434	206,925	288,901	0,481465	0,693084	0,22375	0,481109	no
XLOC_001925	g6435	11,8157	9,55652	-0,306154	-0,493639	0,39005	0,645047	no
XLOC_001926	g1530	2,38197	4,50954	0,920825	0,691721	0,24745	0,506238	no
XLOC_001927	g6436	71,2087	58,6397	-0,280176	-0,471093	0,4009	0,654912	no
XLOC_001928	g6437	0	0	0	0	1	1	no
XLOC_001929	g6438	14,2906	17,3118	0,276692	0,454186	0,4208	0,671416	no
XLOC_001930	g6439	0	0	0	0	1	1	no
XLOC_001931	g6440	0	0	0	0	1	1	no

XLOC_001932	g6441	16,9017	8,87585	-0,929213	-1,05409	0,0653	0,231832	no
XLOC_001933	g6442	69,2582	59,049	-0,230073	-0,3896	0,4841	0,717963	no
XLOC_001934	g6444	12,2898	10,7689	-0,190597	-0,308436	0,57805	0,78137	no
XLOC_001935	g6443	29,3324	13,8475	-1,08287	-1,59873	0,0065	0,0492896	yes
XLOC_001936	g6445	24,7395	29,662	0,261798	0,369316	0,522	0,745347	no
XLOC_001937	g6446	0	0	0	0	1	1	no
XLOC_001938	g6447	0,0216866	0,133497	2,62193	0	1	1	no
XLOC_001939	g6448	13,94	11,7128	-0,251149	-0,334671	0,5677	0,776436	no
XLOC_001940	g6450	52,2135	59,5507	0,189698	0,32285	0,5627	0,772925	no
XLOC_001941	g6449	68,4628	85,225	0,315957	0,495157	0,38525	0,640945	no
XLOC_001942	g6451	44,5212	55,8204	0,3263	0,526296	0,33985	0,600023	no
XLOC_001943	g6452	112,377	98,2592	-0,193682	-0,33094	0,5529	0,766659	no
XLOC_001944	g6453	38,6964	24,3685	-0,667179	-1,00011	0,0683	0,238941	no
XLOC_001945	g6454	0	0	0	0	1	1	no
XLOC_001946	g6455	0	0	0	0	1	1	no
XLOC_001947	g6456	0	0	0	0	1	1	no
XLOC_001948	g6457	0	0	0	0	1	1	no
XLOC_001949	g6458	0,178366	0,160906	-0,148618	0	1	1	no
XLOC_001950	g6459	0	0	0	0	1	1	no
XLOC_001951	g6460	0	0	0	0	1	1	no
XLOC_001952	g6461	0	0	0	0	1	1	no
XLOC_001953	g6462	0	0,259047	inf	0	1	1	no
XLOC_001954	g6464	125,746	187,469	0,576138	0,731217	0,17985	0,426853	no
XLOC_001955	g6466	42,2855	51,2636	0,277772	0,452763	0,41935	0,670796	no
XLOC_001956	g6463	59,4236	55,0401	-0,110552	-0,16985	0,7631	0,888504	no
XLOC_001957	g6465	98,5456	104,279	0,0815841	0,136685	0,8133	0,914846	no
XLOC_001958	g6467	4,44522	3,90664	-0,186328	-0,235182	0,67325	0,841348	no
XLOC_001959	g1531	37,1803	37,9723	0,0304073	0,0456119	0,93515	0,971715	no
XLOC_001960	g1533	46,1143	48,8572	0,0833581	0,124824	0,8225	0,919263	no
XLOC_001961	g1532	13,3145	78,8617	2,56632	2,17309	0,00335	0,0307449	yes
XLOC_001962	g1534	20,833	21,2897	0,0312836	0,0460158	0,9369	0,972153	no
XLOC_001963	g1535	721,873	773,987	0,100564	0,142838	0,7981	0,907108	no
XLOC_001964	g1536	24,1228	17,9083	-0,429769	-0,674577	0,2386	0,496725	no
XLOC_001965	g1537	0	0	0	0	1	1	no
XLOC_001966	g1538	15,5625	15,7681	0,0189357	0,0170859	0,96065	0,982256	no
XLOC_001967	g1542	45,4436	59,8224	0,396609	0,602236	0,28135	0,543655	no
XLOC_001968	g1539	14,3712	13,728	-0,0660541	-0,105433	0,8503	0,932746	no
XLOC_001969	g1540	25,3444	36,16	0,512731	0,734616	0,1962	0,447442	no
XLOC_001970	g1541	188,995	166,572	-0,182199	-0,307234	0,59145	0,790276	no
XLOC_001971	g1543	58,0561	45,0861	-0,364766	-0,610655	0,2935	0,555829	no
XLOC_001972	g6468	20,2108	20,6024	0,027683	0,0431652	0,93865	0,97252	no
XLOC_001973	g165	1590,21	2486,9	0,645134	0,88812	0,1213	0,343573	no
XLOC_001974	g6469	0	0	0	0	1	1	no
XLOC_001975	g6470	0	0	0	0	1	1	no
XLOC_001976	g6471	0	0	0	0	1	1	no
XLOC_001977	g6472	0	0,123847	inf	0	1	1	no
XLOC_001978	g6474	7,14683	9,18231	0,361555	0,492982	0,3815	0,637999	no
XLOC_001979	g6475	4,28257	6,43855	0,588259	0,753429	0,1959	0,447173	no
XLOC_001980	g6473	31,4791	21,9838	-0,517955	-0,803543	0,17285	0,418282	no
XLOC_001981	g6476	0	0	0	0	1	1	no
XLOC_001982	g6477	0	0	0	0	1	1	no
XLOC_001983	g6478	75,8259	87,2189	0,20195	0,320652	0,57575	0,780604	no
XLOC_001984	g6480	439,862	1063,97	1,27433	1,89571	0,00255	0,0250912	yes
XLOC_001985	g6479	138,874	529,228	1,93011	2,43514	5,00E-05	0,00120049	yes
XLOC_001986	g6481	67,8259	66,0046	-0,0392698	-0,0602751	0,9153	0,963526	no
XLOC_001987	g6482	0	0	0	0	1	1	no
XLOC_001988	g1544	122,636	95,0185	-0,368098	-0,620258	0,27035	0,531712	no
XLOC_001989	g1545	5,37482	1,64611	-1,70716	-1,88316	0,0031	0,0290477	yes
XLOC_001990	g6483	48,6099	41,2886	-0,235507	-0,364377	0,50975	0,736031	no
XLOC_001991	g6484	13,9117	46,5856	1,74358	2,74628	5,00E-05	0,00120049	yes
XLOC_001992	g6485	0	0	0	0	1	1	no
XLOC_001993	g6486	0	0	0	0	1	1	no
XLOC_001994	g1548	0,15444	1,17416	2,92651	1,40903	0,0958	0,295649	no
XLOC_001995	g1549	37,0658	43,6752	0,236726	0,334868	0,5521	0,766299	no
XLOC_001996	g1546	38,1825	26,7552	-0,513089	-0,742428	0,18	0,426853	no
XLOC_001997	g1547	39,6961	42,8883	0,111586	0,190382	0,73275	0,873889	no
XLOC_001998	g1550	27,8955	27,754	-0,00733827	-0,0100134	0,9847	0,992379	no
XLOC_001999	g1551	8,98274	5,48858	-0,710722	-0,87134	0,12365	0,347009	no
XLOC_002000	g1552	87,0943	99,996	0,199292	0,317667	0,57515	0,780604	no
XLOC_002001	g6487	22,3626	7,30563	-1,61401	-2,24407	0,00045	0,00669545	yes
XLOC_002002	g6488	62,9177	72,1679	0,197892	0,315062	0,58605	0,786849	no
XLOC_002003	g1553	19,4524	12,4662	-0,641924	-0,943771	0,08995	0,28448	no
XLOC_002004	g1554	17,22	23,2088	0,430586	0,520488	0,37025	0,629539	no
XLOC_002005	g1555	11,0189	8,68709	-0,343033	-0,507577	0,3811	0,637764	no
XLOC_002006	g1556	138,366	90,2324	-0,616772	-0,91908	0,10505	0,313649	no
XLOC_002007	g1557	571,52	143,675	-1,992	-2,84456	5,00E-05	0,00120049	yes
XLOC_002008	g6489	109,349	105,142	-0,0566028	-0,0921762	0,8725	0,942536	no
XLOC_002009	g6491	41,5026	39,727	-0,0630826	-0,0993505	0,8624	0,936559	no
XLOC_002010	g6492	76,8894	83,7677	0,12361	0,201033	0,7212	0,868141	no
XLOC_002011	g6490	2,22812	4,30282	0,949455	1,02756	0,075	0,253006	no
XLOC_002012	g6493	6,17696	8,25613	0,418569	0,632493	0,2627	0,523149	no
XLOC_002013	g6494	20,6691	49,7613	1,26755	2,0915	0,0004	0,0061761	yes
XLOC_002014	g6495	15,3508	13,5586	-0,179109	-0,183527	0,7439	0,879179	no
XLOC_002015	g6496	5,36469	5,44812	0,0222638	0,0257958	0,96895	0,98653	no
XLOC_002016	g6497	373,976	217,485	-0,782033	-1,20964	0,0362	0,159196	no
XLOC_002017	g6499	73,9234	88,5779	0,260915	0,45717	0,42885	0,677386	no
XLOC_002018	g6501	44,5344	32,7317	-0,44423	-0,702419	0,2095	0,4644	no
XLOC_002019	g6498	11,6518	11,7842	0,0162974	0,0247032	0,9646	0,984142	no
XLOC_002020	g6500	91,0933	101,89	0,161597	0,286455	0,62685	0,812951	no
XLOC_002021	g6502	85,2504	69,6905	-0,290744	-0,480276	0,4058	0,659433	no
XLOC_002022	g1559	240,012	298,507	0,314661	0,422598	0,47625	0,712814	no
XLOC_002023	g1560	575,42	565,429	-0,0252676	-0,033193	0,95405	0,979587	no

XLOC_002024	g1561	1,68728	2,07175	0,296153	0,243447	0,66775	0,83795	no
XLOC_002025	g1562	71,1369	53,5034	-0,410968	-0,703419	0,2198	0,477108	no
XLOC_002026	g1564	30,0331	39,9155	0,410394	0,66933	0,24115	0,499282	no
XLOC_002027	g1566	29,0363	42,2759	0,541978	0,893322	0,12445	0,34778	no
XLOC_002028	g1568	88,5274	139,844	0,659628	1,03159	0,06365	0,228702	no
XLOC_002029	g1570	104,147	155,561	0,578863	0,959764	0,0978	0,299843	no
XLOC_002030	g1571	56,0735	18,4028	-1,60739	-2,56909	5,00E-05	0,00120049	yes
XLOC_002031	g1573	6,89117	6,28161	-0,133616	-0,193328	0,7279	0,870385	no
XLOC_002032	g1575	74,1391	90,0882	0,281104	0,456655	0,4225	0,672657	no
XLOC_002033	g1578	39,5697	38,7712	-0,0294113	-0,0486145	0,93425	0,971293	no
XLOC_002034	g1580	35,1405	34,6069	-0,0220751	-0,0368717	0,9514	0,978606	no
XLOC_002035	g1582	98,3569	116,195	0,24045	0,408029	0,48175	0,716101	no
XLOC_002036	g1584	1,67649	1,80443	0,106106	0,100875	0,8596	0,935634	no
XLOC_002037	g1586	77,8798	131,844	0,759505	1,30213	0,02285	0,119609	no
XLOC_002038	g1588	120,682	135,972	0,172104	0,300981	0,59805	0,794918	no
XLOC_002039	g1589	67,4385	72,5886	0,106171	0,169765	0,76645	0,890609	no
XLOC_002040	g1592	36,5655	46,541	0,34802	0,58119	0,28935	0,55259	no
XLOC_002041	g1594	94,21	40,069	-1,23339	-2,02295	0,00075	0,0100752	yes
XLOC_002042	g1596	42,1899	35,8674	-0,234226	-0,391319	0,4914	0,723327	no
XLOC_002043	g1599	75,2896	69,3272	-0,119028	-0,201971	0,71705	0,865633	no
XLOC_002044	g1558	51,169	39,43	-0,375979	-0,605015	0,2917	0,554382	no
XLOC_002045	g1563	2,69721	7,67807	1,50928	2,06814	0,0006	0,00845337	yes
XLOC_002046	g1565	31,5088	37,0916	0,23534	0,375623	0,5059	0,733528	no
XLOC_002047	g1567	42,8938	33,6279	-0,351109	-0,573349	0,30795	0,568862	no
XLOC_002048	g1569	13,954	17,3417	0,313561	0,499373	0,3939	0,648574	no
XLOC_002049	g1572	6,7739	5,38602	-0,330767	-0,420374	0,46155	0,702668	no
XLOC_002050	g1574	32,8575	110,892	1,75486	2,73749	0,0001	0,00209829	yes
XLOC_002051	g1576	57,6276	84,584	0,553625	0,91415	0,1112	0,325383	no
XLOC_002052	g1577	57,1514	59,155	0,0497106	0,0850848	0,88225	0,947324	no
XLOC_002053	g1579	56,2331	82,1291	0,546475	0,807197	0,1625	0,404499	no
XLOC_002054	g1581	55,902	58,4339	0,0639074	0,0980508	0,85855	0,935373	no
XLOC_002055	g1583	62,7273	73,5213	0,229069	0,298357	0,60205	0,798289	no
XLOC_002056	g1585	68,3788	59,6797	-0,196309	-0,316166	0,57815	0,78137	no
XLOC_002057	g1587	119,327	120,45	0,0135063	0,020471	0,97165	0,986977	no
XLOC_002058	g1590	131,667	140,816	0,0969172	0,168297	0,76565	0,890392	no
XLOC_002059	g1591	54,3926	53,2948	-0,0294168	-0,048007	0,93525	0,971715	no
XLOC_002060	g1593	21,692	13,5923	-0,674376	-0,979199	0,08455	0,274382	no
XLOC_002061	g1595	8,85613	4,65263	-0,928629	-1,15418	0,047	0,188307	no
XLOC_002062	g1597	44,7999	35,6913	-0,327924	-0,55468	0,32865	0,5899	no
XLOC_002063	g1598	71,1043	105,854	0,574068	0,945068	0,09835	0,300778	no
XLOC_002064	g6503	10,8537	2,43369	-2,15697	-1,96253	0,006	0,0465771	yes
XLOC_002065	g6504	0,590791	0,549505	-0,104514	0	1	1	no
XLOC_002066	g6505	0	0	0	0	1	1	no
XLOC_002067	g6506	0	0	0	0	1	1	no
XLOC_002068	g6507	21,0999	14,4715	-0,544025	-0,919002	0,1136	0,33024	no
XLOC_002069	g1600	0	0	0	0	1	1	no
XLOC_002070	g6508	15,5951	49,7883	1,67472	1,75302	0,00335	0,0307449	yes
XLOC_002071	g1601	13,6094	13,3833	-0,0241646	-0,0340587	0,9552	0,98046	no
XLOC_002072	g1603	10,7463	0,460522	-4,54442	-3,49958	0,026	0,129802	no
XLOC_002073	g1604	12,2883	11,8975	-0,0466257	-0,0708718	0,90275	0,957458	no
XLOC_002074	g1602	87,9109	35,31	-1,31597	-1,78602	0,00335	0,0307449	yes
XLOC_002075	g1605	43,5589	40,275	-0,11308	-0,18977	0,7371	0,87578	no
XLOC_002076	g6509	42,5868	45,0117	0,0798933	0,111423	0,8392	0,92642	no
XLOC_002077	g6511	47,9079	44,8791	-0,09422	-0,164068	0,7734	0,894251	no
XLOC_002078	g6512	25,6024	40,6895	0,668379	1,08797	0,05565	0,210591	no
XLOC_002079	g6514	128,309	210,117	0,711567	1,13374	0,049	0,194024	no
XLOC_002080	g6516	152,225	191,228	0,329088	0,567633	0,31265	0,573414	no
XLOC_002081	g6517	88,3102	51,6853	-0,772825	-1,31798	0,0201	0,109292	no
XLOC_002082	g6519	34,2318	52,8964	0,627831	0,906019	0,10855	0,320397	no
XLOC_002083	g6521	63,2732	66,3298	0,0680629	0,100224	0,8593	0,935617	no
XLOC_002084	g6522	958,73	1333,43	0,475947	0,788869	0,1644	0,406447	no
XLOC_002085	g6524	32,6997	42,4045	0,37494	0,554948	0,3291	0,590276	no
XLOC_002086	g6510	685,972	1431,98	1,06179	1,43613	0,0152	0,0892194	no
XLOC_002087	g6513	50,4911	69,2701	0,456204	0,725101	0,2042	0,457401	no
XLOC_002088	g6515	5,77451	11,1505	0,949337	1,30126	0,02715	0,133373	no
XLOC_002089	g6518	11,3009	14,8288	0,39197	0,469764	0,4309	0,678624	no
XLOC_002090	g6520	32,8439	39,7575	0,275601	0,471685	0,39945	0,653222	no
XLOC_002091	g6523	208,293	276,83	0,410386	0,572203	0,30735	0,568181	no
XLOC_002092	g6525	9,45164	8,01435	-0,237979	-0,361675	0,53295	0,752918	no
XLOC_002093	g6526	0	0	0	0	1	1	no
XLOC_002094	g6527	13,793	13,7772	-0,00165068	-0,00232038	0,9968	0,997966	no
XLOC_002095	g1607	203,23	260,007	0,355438	0,527737	0,3303	0,591007	no
XLOC_002096	g1606	1526,34	2578,72	0,756584	1,14033	0,0396	0,168928	no
XLOC_002097	g6528	164,424	219,816	0,418871	0,688331	0,21145	0,46693	no
XLOC_002098	g6529	31,692	34,0634	0,104102	0,145008	0,8022	0,908624	no
XLOC_002099	g6531	15,253	33,9477	1,15423	1,83664	0,00215	0,022131	yes
XLOC_002100	g6530	70,0431	119,879	0,775259	1,31879	0,0248	0,125793	no
XLOC_002101	g6532	76,5257	103,03	0,429042	0,73079	0,2007	0,452554	no
XLOC_002102	g6533	30,5269	31,2275	0,0327381	0,0362662	0,94765	0,976282	no
XLOC_002103	g6534	0	0	0	0	1	1	no
XLOC_002104	g1608	40,1615	44,1449	0,136434	0,180287	0,7555	0,884596	no
XLOC_002105	g6535	0	0	0	0	1	1	no
XLOC_002106	g6536	1,15671	1,24609	0,107385	0,0910458	0,86935	0,940822	no
XLOC_002107	g6537	0	0	0	0	1	1	no
XLOC_002108	g6538	178,529	187,31	0,0692665	0,113724	0,83685	0,925387	no
XLOC_002109	g6539	11,8177	27,3594	1,21108	1,70724	0,0037	0,032971	yes
XLOC_002110	g6540	29,1502	47,2896	0,698017	1,08861	0,06025	0,220932	no
XLOC_002111	g6541	165,009	237,203	0,523573	0,743217	0,1907	0,440837	no
XLOC_002112	g1610	68,1458	106,898	0,649536	1,09633	0,0576	0,214906	no
XLOC_002113	g1613	27,3132	28,0798	0,0399347	0,0693664	0,90305	0,957458	no
XLOC_002114	g1615	579,394	524,604	-0,143316	-0,223146	0,7006	0,857628	no
XLOC_002115	g1617	50,7266	40,1128	-0,338681	-0,571946	0,30375	0,565143	no

XLOC_002116	g1618	83,5653	81,2072	-0,0412953	-0,0676074	0,90695	0,959725	no
XLOC_002117	g1620	9,0582	6,8526	-0,402573	-0,533517	0,35425	0,615379	no
XLOC_002118	g1622	44,2176	45,5166	0,0417705	0,069513	0,90505	0,958438	no
XLOC_002119	g1609	24,4864	23,9362	-0,0327858	-0,053745	0,9258	0,967166	no
XLOC_002120	g1611	53,9712	52,4094	-0,0423663	-0,061815	0,91325	0,963071	no
XLOC_002121	g1612	11,0815	17,3617	0,647756	0,684966	0,24195	0,500303	no
XLOC_002122	g1614	22,0036	25,1127	0,190676	0,323332	0,5804	0,783024	no
XLOC_002123	g1616	22,1132	27,4451	0,311645	0,501461	0,3885	0,643542	no
XLOC_002124	g1619	123,703	140,503	0,183729	0,29126	0,6044	0,79937	no
XLOC_002125	g1621	105,364	81,5809	-0,369081	-0,640903	0,27105	0,532449	no
XLOC_002126	g1623	89,0182	81,9234	-0,119825	-0,190067	0,7384	0,87637	no
XLOC_002127	g6542	0	0	0	0	1	1	no
XLOC_002128	g6543	46,2092	86,9179	0,911474	1,54314	0,007	0,0518011	no
XLOC_002129	g6544	9,08787	8,03355	-0,177905	-0,231191	0,69	0,851552	no
XLOC_002130	g6545	103,506	63,1818	-0,712133	-1,1913	0,0317	0,146422	no
XLOC_002131	g1625	59,548	54,2668	-0,133984	-0,218199	0,692	0,853055	no
XLOC_002132	g1626	4,01095	3,59474	-0,158058	-0,18461	0,7492	0,881846	no
XLOC_002133	g1628	8,72183	8,86731	0,023866	0,0374046	0,9471	0,97628	no
XLOC_002134	g1629	40,3159	45,8728	0,186292	0,319736	0,57205	0,778806	no
XLOC_002135	g1631	11,4468	28,734	1,32782	2,16325	0,0001	0,00209829	yes
XLOC_002136	g1632	65,1291	186,926	1,52109	2,23354	0,00035	0,00552572	yes
XLOC_002137	g1635	249,879	192,282	-0,378003	-0,605883	0,293	0,555336	no
XLOC_002138	g1624	61,6604	79,1342	0,359958	0,58825	0,31035	0,571193	no
XLOC_002139	g1627	50,786	67,5587	0,411711	0,625949	0,2613	0,521008	no
XLOC_002140	g1630	7,18484	11,5804	0,688652	0,918101	0,11485	0,332398	no
XLOC_002141	g1633	36,8193	42,6207	0,211095	0,366595	0,52635	0,748119	no
XLOC_002142	g1634	16,5462	19,6794	0,250184	0,330735	0,5512	0,766024	no
XLOC_002143	g1636	53,7096	88,3554	0,718138	1,09399	0,0484	0,192269	no
XLOC_002144	g6546	23,444	32,3421	0,464191	0,636911	0,2671	0,528062	no
XLOC_002145	g6547	26,9276	28,0882	0,0608756	0,105697	0,85735	0,935212	no
XLOC_002146	g6548	0,802983	1,64173	1,03178	0,830035	0,1694	0,412679	no
XLOC_002147	g6550	1,15745	0,4593	-1,33344	0	1	1	no
XLOC_002148	g6551	582,942	763,48	0,389237	0,541037	0,34545	0,605535	no
XLOC_002149	g6553	15,5731	21,0645	0,435759	0,625677	0,273	0,53424	no
XLOC_002150	g6549	0,330113	1,03483	1,64836	0	1	1	no
XLOC_002151	g6552	11,7743	7,70054	-0,612609	-0,777449	0,1757	0,421955	no
XLOC_002152	g6554	0	0,139837	inf	0	1	1	no
XLOC_002153	g1638	82,2732	63,5733	-0,372	-0,392998	0,48565	0,719067	no
XLOC_002154	g1637	1,07313	0,868658	-0,304961	0	1	1	no
XLOC_002155	g1639	2,25475	6,85478	1,60414	1,23574	0,24675	0,505547	no
XLOC_002156	g1640	1,65498	1,05729	-0,646449	-0,613163	0,3223	0,582656	no
XLOC_002157	g6555	382,531	295,296	-0,373415	-0,608358	0,2806	0,542847	no
XLOC_002158	g6557	91,788	133,693	0,542547	0,916865	0,1173	0,335631	no
XLOC_002159	g6558	19,0216	24,4937	0,364776	0,608971	0,282	0,544376	no
XLOC_002160	g6556	8,24718	9,22716	0,161986	0,186363	0,74915	0,881846	no
XLOC_002161	g6559	88,7327	14,4184	-2,62156	-3,67849	5,00E-05	0,00120049	yes
XLOC_002162	g6562	81,3468	43,8118	-0,892767	-1,34664	0,0145	0,0866119	no
XLOC_002163	g6560	40,9018	93,7119	1,19607	1,31013	0,02755	0,134531	no
XLOC_002164	g6561	58,4492	13,6033	-2,10323	-2,63894	5,00E-05	0,00120049	yes
XLOC_002165	g6563	18,1405	7,39605	-1,29439	-1,68661	0,00355	0,031924	yes
XLOC_002166	g1641	3,25751	4,52315	0,473556	0,458653	0,43245	0,679357	no
XLOC_002167	g1643	0,725408	0,474099	-0,613604	0	1	1	no
XLOC_002168	g1644	38,9984	14,8799	-1,39005	-1,96004	0,0049	0,0404013	yes
XLOC_002169	g1646	97,6322	94,0005	-0,054689	-0,089253	0,8762	0,944231	no
XLOC_002170	g1648	1,9132	2,3527	0,298327	0,311712	0,5838	0,784861	no
XLOC_002171	g1642	36,2494	54,6592	0,592507	0,914713	0,1099	0,323169	no
XLOC_002172	g1645	2,43527	1,69885	-0,519522	-0,604412	0,30165	0,563478	no
XLOC_002173	g1647	18,2616	4,18748	-2,12466	-2,98388	5,00E-05	0,00120049	yes
XLOC_002174	g6564	15,8312	8,7289	-0,858895	-1,02484	0,0794	0,263237	no
XLOC_002175	g6568	33,2213	17,4525	-0,92867	-1,42824	0,01345	0,082241	no
XLOC_002176	g6570	6,59148	2,50958	-1,39316	-1,0638	0,1827	0,429978	no
XLOC_002177	g6565	53,4483	48,1248	-0,151365	-0,235492	0,67955	0,845777	no
XLOC_002178	g6566	199,102	354,181	0,830983	1,09252	0,05435	0,20743	no
XLOC_002179	g6567	13,1516	7,98497	-0,719877	-0,977996	0,08765	0,279888	no
XLOC_002180	g6569	136,861	119,28	-0,198358	-0,34253	0,54155	0,759787	no
XLOC_002181	g6572	0,0553801	0,0315424	-0,812074	0	1	1	no
XLOC_002182	g6571	0	0	0	0	1	1	no
XLOC_002183	g6573	100,984	199,983	0,985753	1,66673	0,0052	0,0421668	yes
XLOC_002184	g6575	3,99156	9,66314	1,27554	1,68325	0,0045	0,038062	yes
XLOC_002185	g6574	6,56061	63,9594	3,28526	4,47119	5,00E-05	0,00120049	yes
XLOC_002186	g6576	0,0516969	0,895391	4,11437	0	1	1	no
XLOC_002187	g1651	5,37105	1,76001	-1,60962	-1,53186	0,01455	0,0868578	no
XLOC_002188	g1649	46,1815	45,405	-0,0244643	-0,0407188	0,94585	0,975656	no
XLOC_002189	g1650	220,525	70,0944	-1,65357	-2,54552	5,00E-05	0,00120049	yes
XLOC_002190	g6577	178,181	265,627	0,576059	0,814084	0,14985	0,387142	no
XLOC_002191	g6579	58,1834	76,4758	0,394396	0,672451	0,23115	0,488674	no
XLOC_002192	g6581	1125,89	2084,17	0,888405	0,878404	0,1268	0,351348	no
XLOC_002193	g6578	104,627	200,207	0,936237	1,57332	0,00585	0,0457017	yes
XLOC_002194	g6580	115,457	112,087	-0,042735	-0,073012	0,8964	0,953906	no
XLOC_002195	g6582	2,23327	2,18753	-0,0298546	-0,0231884	0,93715	0,972153	no
XLOC_002196	g6583	616,486	674,763	0,130313	0,212656	0,70385	0,859387	no
XLOC_002197	g6584	196,114	206,816	0,0766519	0,128408	0,82965	0,922056	no
XLOC_002198	g6585	24,7537	43,9875	0,82945	1,23309	0,0306	0,143091	no
XLOC_002199	g166	127,552	83,394	-0,613072	-1,05121	0,06475	0,230629	no
XLOC_002200	g167	22,7194	57,7264	1,34531	2,03154	0,00125	0,0146655	yes
XLOC_002201	g6586	9,88884	14,0468	0,506366	0,621955	0,2694	0,5308	no
XLOC_002202	g6587	0	0	0	0	1	1	no
XLOC_002203	g6588	0	0	0	0	1	1	no
XLOC_002204	g6589	0	0	0	0	1	1	no
XLOC_002205	g6590	0,217206	1,59779	2,87894	1,60629	0,2367	0,494447	no
XLOC_002206	g6591	29,294	48,5196	0,727963	1,17955	0,04425	0,181056	no
XLOC_002207	g1652	0	0	0	0	1	1	no

XLOC_002208	g6592	8,56253	6,20829		-0,463842	-0,519155	0,4646	0,705296	no
XLOC_002209	g6594	13,0704	30,1896		1,20775	1,88006	0,00125	0,0146655	yes
XLOC_002210	g6593	126,731	100,764		-0,330795	-0,495604	0,37845	0,636208	no
XLOC_002211	g6595	0	2,23196	inf		0	1	1	no
XLOC_002212	g6596	3,08041	2,65565		-0,214061	-0,217747	0,7026	0,859005	no
XLOC_002213	g6597	207,975	140,804		-0,562725	-0,717586	0,20765	0,462294	no
XLOC_002214	g6598	88,7741	89,4824		0,0114651	0,0195442	0,97345	0,98758	no
XLOC_002215	g6599	3,88861	7,07922		0,864335	2,22358	0,52395	0,746323	no
XLOC_002216	g6600	17,4356	16,9924		-0,0371485	-0,0634293	0,90905	0,961172	no
XLOC_002217	g6601	4,9232	6,89381		0,485705	0,634432	0,274	0,535565	no
XLOC_002218	g6602	28,9595	11,1708		-1,3743	-1,82213	0,00185	0,01964	yes
XLOC_002219	g6603	0	0		0	0	1	1	no
XLOC_002220	g1655	28,179	42,988		0,609313	0,985587	0,08455	0,274382	no
XLOC_002221	g1658	58,286	60,2863		0,0486822	0,0843288	0,885	0,94862	no
XLOC_002222	g1660	6,44353	10,2744		0,673128	0,946068	0,09835	0,300778	no
XLOC_002223	g1664	47,4599	67,2442		0,5027	0,851209	0,14025	0,373746	no
XLOC_002224	g1653	104,838	71,106		-0,560123	-0,831153	0,13925	0,372294	no
XLOC_002225	g1654	36,9987	46,4072		0,326873	0,52898	0,3591	0,619938	no
XLOC_002226	g1656	48,2621	49,1991		0,0277423	0,0460141	0,93575	0,972028	no
XLOC_002227	g1657	80,0325	70,8995		-0,174811	-0,301044	0,59275	0,7913	no
XLOC_002228	g1659	27,0854	23,3382		-0,214826	-0,363473	0,5108	0,736969	no
XLOC_002229	g1661	89,5146	21,3468		-2,0681	-3,25958	5,00E-05	0,00120049	yes
XLOC_002230	g1662	39,3951	39,3969		6,76E-05	0,000115183	0,99885	0,999053	no
XLOC_002231	g1663	113,283	150,479		0,40963	0,708263	0,22085	0,477698	no
XLOC_002232	g6605	42,2591	64,2807		0,605125	1,04689	0,073	0,249343	no
XLOC_002233	g6604	32,9925	38,0929		0,207387	0,324134	0,5662	0,775141	no
XLOC_002234	g1665	195,807	113,853		-0,782266	-1,03972	0,07435	0,252007	no
XLOC_002235	g6606	36,8655	68,5086		0,894013	1,47494	0,00945	0,0639552	no
XLOC_002236	g6607	2,90177	1,54376		-0,910487	-0,907652	0,1111	0,325284	no
XLOC_002237	g6608	394,079	0		#NAME? 0	#NAME? 0	0,0087	0,0603347	no
XLOC_002238	g6609	0	0		0	0	1	1	no
XLOC_002239	g6610	0	0		0	0	1	1	no
XLOC_002240	g6612	4,19655	5,30007		0,336808	0,38783	0,4966	0,726443	no
XLOC_002241	g6611	38,0709	55,9416		0,555233	0,809731	0,16355	0,405776	no
XLOC_002242	g6613	31,9046	27,0662		-0,237269	-0,389619	0,4911	0,723137	no
XLOC_002243	g6615	95,7687	140,635		0,554333	0,958611	0,0981	0,300387	no
XLOC_002244	g6618	54,8055	61,4285		0,164589	0,269945	0,63215	0,816117	no
XLOC_002245	g6614	45,1718	62,9506		0,478797	0,767884	0,1753	0,421303	no
XLOC_002246	g6616	31,7416	28,2328		-0,169004	-0,278214	0,6328	0,816463	no
XLOC_002247	g6617	22,2368	23,2736		0,0657472	0,106085	0,8463	0,929972	no
XLOC_002248	g1666	7,64372	6,65442		-0,199962	-0,272737	0,63765	0,819812	no
XLOC_002249	g6620	20,7749	22,6014		0,121573	0,190618	0,7385	0,876383	no
XLOC_002250	g6622	117,011	139,217		0,250698	0,438442	0,4486	0,694314	no
XLOC_002251	g6619	30,9903	24,3631		-0,34712	-0,420002	0,4711	0,708963	no
XLOC_002252	g6621	69,8613	56,3238		-0,31075	-0,519306	0,3616	0,621784	no
XLOC_002253	g6623	339,827	694,527		1,03123	1,62632	0,0049	0,0404013	yes
XLOC_002254	g6624	0	0,122577	inf		0	1	1	no
XLOC_002255	g6625	0,439891	0,560024		0,34834	0	1	1	no
XLOC_002256	g6626	12,3988	14,1434		0,189933	0,246795	0,67245	0,841098	no
XLOC_002257	g6627	102,437	201,792		0,978128	0,708737	0,23585	0,493721	no
XLOC_002258	g6628	10,3291	11,952		0,210537	0,265754	0,6485	0,827004	no
XLOC_002259	g6629	8,90932	26,6905		1,58294	2,24679	0,0002	0,00367103	yes
XLOC_002260	g6630	0,0891809	2,62777		4,88096	1,19716	0,1168	0,335079	no
XLOC_002261	g6631	0	0		0	0	1	1	no
XLOC_002262	g6632	31,5492	34,8747		0,144576	0,209987	0,70895	0,86194	no
XLOC_002263	g6633	0,964809	19,441		4,33271	3,36935	0,1237	0,347009	no
XLOC_002264	g6634	2,94044	6,78802		1,20696	1,23359	0,02875	0,137787	no
XLOC_002265	g6635	16,374	17,8213		0,1222	0,171871	0,75465	0,884327	no
XLOC_002266	g6636	1870,92	620,289		-1,59273	-1,77296	0,0029	0,0276485	yes
XLOC_002267	g6639	131,04	159,977		0,287851	0,472953	0,39475	0,648995	no
XLOC_002268	g6637	2,86658	3,46512		0,273574	0,237833	0,67775	0,844286	no
XLOC_002269	g6638	164,539	153,602		-0,099231	-0,145325	0,796	0,906216	no
XLOC_002270	g6640	0	0		0	0	1	1	no
XLOC_002271	g1667	44,5821	67,6004		0,600569	0,86594	0,12705	0,351842	no
XLOC_002272	g1668	1,61572	1,43934		-0,166761	-0,127601	0,8288	0,921619	no
XLOC_002273	g1669	691,113	915,147		0,405081	0,492358	0,388	0,643448	no
XLOC_002274	g6641	0	0		0	0	1	1	no
XLOC_002275	g6643	8,78444	7,57846		-0,213047	-0,238062	0,6737	0,8415	no
XLOC_002276	g6642	23,1256	14,0464		-0,719289	-1,05378	0,06925	0,241147	no
XLOC_002277	g1671	68,8045	121,434		0,819593	1,27807	0,02345	0,121841	no
XLOC_002278	g1672	116,663	101,497		-0,20091	-0,336957	0,5595	0,77102	no
XLOC_002279	g1673	5,95999	9,69698		0,702226	0,832407	0,17895	0,42632	no
XLOC_002280	g1674	231,512	245,54		0,0848705	0,135728	0,8149	0,915968	no
XLOC_002281	g1670	46,3221	51,4317		0,150957	0,245121	0,6621	0,834807	no
XLOC_002282	g1675	50,5027	59,1763		0,228657	0,367634	0,505	0,732944	no
XLOC_002283	g6645	5,05875	2,06066		-1,29567	-1,39915	0,0173	0,097748	no
XLOC_002284	g6644	93,0462	35,8903		-1,37435	-2,26199	5,00E-05	0,00120049	yes
XLOC_002285	g6646	0	0		0	0	1	1	no
XLOC_002286	g6647	0	0		0	0	1	1	no
XLOC_002287	g6649	39,3861	28,5761		-0,46288	-0,601196	0,27865	0,540139	no
XLOC_002288	g6648	448,139	60,1746		-2,89672	-4,04804	5,00E-05	0,00120049	yes
XLOC_002289	g6652	47,1983	50,8653		0,107947	0,178197	0,7584	0,885551	no
XLOC_002290	g6654	129,038	68,6064		-0,911385	-1,49862	0,0102	0,0675415	no
XLOC_002291	g6650	29,9066	155,277		2,37631	3,56105	5,00E-05	0,00120049	yes
XLOC_002292	g6651	6,14406	4,02591		-0,609878	-0,673182	0,24425	0,503031	no
XLOC_002293	g6653	1471,92	2034,75		0,46715	0,681094	0,2433	0,501934	no
XLOC_002294	g1676	21,1154	24,8757		0,236446	0,377268	0,50265	0,730938	no
XLOC_002295	g1677	359,03	868,204		1,27393	1,98169	0,00065	0,00901554	yes
XLOC_002296	g1678	15,3075	16,0963		0,0724863	0,109477	0,8425	0,927922	no
XLOC_002297	g1679	14,0907	7,65931		-0,879454	-1,26902	0,0279	0,135767	no
XLOC_002298	g1680	5,08913	2,03461		-1,32267	-1,26439	0,0386	0,166386	no
XLOC_002299	g1681	0	0		0	0	1	1	no

XLOC_002300	g1684	3,90022	1,2259	-1,66971	-1,40934	0,06275	0,226296	no
XLOC_002301	g1682	25,8357	8,93925	-1,53114	-2,0671	0,0006	0,00845337	yes
XLOC_002302	g1683	1155,55	408,134	-1,50147	-2,28562	0,00025	0,00430702	yes
XLOC_002303	g6655	50,9265	58,2654	0,194223	0,309564	0,5812	0,783336	no
XLOC_002304	g6656	14,7383	25,0401	0,764675	1,0389	0,0741	0,251525	no
XLOC_002305	g6657	19,1904	17,4691	-0,135585	-0,158629	0,77485	0,894759	no
XLOC_002306	g1685	77,9071	61,2018	-0,348182	-0,577384	0,31875	0,579009	no
XLOC_002307	g1686	0,49365	2,42681	2,2975	1,46334	0,0353	0,15728	no
XLOC_002308	g6658	4,51099	2,02748	-1,15375	-1,03425	0,08755	0,279773	no
XLOC_002309	g6659	82,2322	94,5539	0,201432	0,298181	0,59385	0,792015	no
XLOC_002310	g1687	38,3954	32,6614	-0,233349	-0,384425	0,5075	0,734618	no
XLOC_002311	g6660	0	0	0	0	1	1	no
XLOC_002312	g1688	115,47	110,784	-0,0597723	-0,0977811	0,86085	0,935644	no
XLOC_002313	g1689	191,382	200,871	0,0698133	0,116092	0,83505	0,92459	no
XLOC_002314	g6661	0,029175	0	#NAME?	0	1	1	no
XLOC_002315	g6662	0	0,058638	inf	0	1	1	no
XLOC_002316	g6663	3,2239	2,62779	-0,294953	-0,200618	0,8171	0,916915	no
XLOC_002317	g6664	5,30764	5,97768	0,171514	0,144615	0,79675	0,906252	no
XLOC_002318	g6665	0	0	0	0	1	1	no
XLOC_002319	g1691	81,5511	79,476	-0,0371848	-0,0635673	0,9166	0,963809	no
XLOC_002320	g1694	38,0587	40,5091	0,0900196	0,143191	0,80195	0,908624	no
XLOC_002321	g1695	65,8625	49,116	-0,423263	-0,693707	0,2175	0,474223	no
XLOC_002322	g1690	26,8756	22,5885	-0,250712	-0,421863	0,46135	0,702668	no
XLOC_002323	g1692	61,8609	46,8806	-0,400037	-0,679654	0,2366	0,494447	no
XLOC_002324	g1693	77,1298	51,1094	-0,5937	-1,00559	0,0745	0,252007	no
XLOC_002325	g6666	0	0	0	0	1	1	no
XLOC_002326	g6667	0	0	0	0	1	1	no
XLOC_002327	g1697	1762,37	1289,1	-0,451157	-0,454742	0,4242	0,673399	no
XLOC_002328	g1696	1,37867	3,09103	1,16481	1,1376	0,0535	0,205383	no
XLOC_002329	g168	143,716	81,8389	-0,812357	-1,26674	0,03055	0,143091	no
XLOC_002330	g169	2,15144	7,74492	1,84795	1,63811	0,0698	0,242546	no
XLOC_002331	g6668	0	0	0	0	1	1	no
XLOC_002332	g6671	0	2,54865	inf	#NAME?	5,00E-05	0,00120049	yes
XLOC_002333	g6669,g6670	0,0206474	0,562657	4,76823	0	1	1	no
XLOC_002334	g6672	0	0,502989	inf	0	1	1	no
XLOC_002335	g1698	12,0163	253,734	4,40025	6,42474	5,00E-05	0,00120049	yes
XLOC_002336	g1699	99,6244	93,6588	-0,0890858	-0,154115	0,7858	0,901303	no
XLOC_002337	g1700	12,3745	31,2263	1,3354	1,73389	0,00355	0,031924	yes
XLOC_002338	g1701	0	0	0	0	1	1	no
XLOC_002339	g6673	0,336922	0,421154	0,321935	0	1	1	no
XLOC_002340	g6674	25,4916	18,162	-0,489097	-0,767108	0,1807	0,427997	no
XLOC_002341	g6675	0	0	0	0	1	1	no
XLOC_002342	g6676	0	0	0	0	1	1	no
XLOC_002343	g6677	117,389	156,113	0,411292	0,551527	0,323	0,583565	no
XLOC_002344	g6679	218,225	222,374	0,0271713	0,0437401	0,9358	0,972028	no
XLOC_002345	g6678	55,4852	36,0734	-0,621169	-1,05138	0,06915	0,24097	no
XLOC_002346	g6680	856,928	1084,76	0,340125	0,569097	0,3165	0,577009	no
XLOC_002347	g1702	0	0,162726	inf	0	1	1	no
XLOC_002348	g6681	67,3877	94,5789	0,489032	0,83901	0,1372	0,369327	no
XLOC_002349	g1704	66,6634	59,3176	-0,168435	-0,273701	0,6195	0,808526	no
XLOC_002350	g1705	83,9927	72,2013	-0,218239	-0,361307	0,5353	0,754615	no
XLOC_002351	g1703	226,154	203,775	-0,150334	-0,253507	0,6536	0,829883	no
XLOC_002352	g6682	26,1007	23,265	-0,16593	-0,238469	0,67005	0,839486	no
XLOC_002353	g6683	157,3	221,069	0,490975	0,687056	0,20955	0,464406	no
XLOC_002354	g6684	24,9054	23,4221	-0,08859	-0,150775	0,7863	0,901303	no
XLOC_002355	g6685	60,4358	46,8606	-0,367029	-0,621397	0,27625	0,537929	no
XLOC_002356	g6686	0	0	0	0	1	1	no
XLOC_002357	g1706	3,37812	3,28616	-0,0398191	-0,053172	0,9235	0,966357	no
XLOC_002358	g6687	93,0671	104,392	0,165668	0,267546	0,63175	0,816117	no
XLOC_002359	g6688	98,6031	48,5773	-1,02135	-1,62938	0,0077	0,0555169	no
XLOC_002360	g6690	210,715	417,026	0,984847	1,47566	0,01075	0,0699569	no
XLOC_002361	g6692	40,9809	55,7644	0,444394	0,766231	0,1813	0,429004	no
XLOC_002362	g6694	103,013	101,332	-0,0237295	-0,0403961	0,9433	0,975074	no
XLOC_002363	g6696	114,595	171,039	0,577781	0,942628	0,09015	0,284995	no
XLOC_002364	g6700	109,591	88,7662	-0,304046	-0,523134	0,36475	0,624341	no
XLOC_002365	g6689	34,3502	33,1779	-0,0500943	-0,0774064	0,8912	0,951363	no
XLOC_002366	g6691	52,7794	71,573	0,439441	0,741912	0,1816	0,4293	no
XLOC_002367	g6693	46,9997	62,3642	0,408065	0,693375	0,21435	0,469638	no
XLOC_002368	g6695	73,807	56,7915	-0,378084	-0,637814	0,24995	0,508496	no
XLOC_002369	g6697	52,4351	42,0878	-0,31713	-0,501543	0,3687	0,628691	no
XLOC_002370	g6698	31,3189	36,8608	0,235054	0,401857	0,4806	0,715629	no
XLOC_002371	g6699	52,1695	69,9458	0,423031	0,702208	0,2226	0,480003	no
XLOC_002372	g6702	18,8171	16,6606	-0,175598	-0,254891	0,65595	0,831474	no
XLOC_002373	g6701	2,5329	3,97095	0,648696	0,865202	0,1185	0,337981	no
XLOC_002374	g6703	131,488	193,385	0,556541	0,890979	0,1159	0,333955	no
XLOC_002375	g6704	6,28676	6,70931	0,0938478	0,103449	0,85825	0,935373	no
XLOC_002376	g6705	155,006	25,016	-2,6314	-2,68122	0,00015	0,00294012	yes
XLOC_002377	g6706	9,27049	12,8238	0,468103	0,450397	0,4314	0,678624	no
XLOC_002378	g6707	111,427	171,531	0,622376	0,982665	0,0891	0,282793	no
XLOC_002379	g6708	0,196045	0,0894668	-1,13176	0	1	1	no
XLOC_002380	g6711	936,618	317,896	-1,55891	-2,38231	5,00E-05	0,00120049	yes
XLOC_002381	g6712	87,6425	141,393	0,690011	1,1781	0,0392	0,167659	no
XLOC_002382	g6715	120,073	178,608	0,572881	0,84802	0,15115	0,38866	no
XLOC_002383	g6716	14,7498	17,98	0,2857	0,486008	0,38225	0,638793	no
XLOC_002384	g6718	171,181	186,768	0,125723	0,178343	0,74055	0,876968	no
XLOC_002385	g6709	19,8592	15,3099	-0,375343	-0,638525	0,2615	0,521301	no
XLOC_002386	g6710	19,6618	28,4065	0,530826	0,632755	0,27195	0,53315	no
XLOC_002387	g6713	0,605915	1,0366	0,774669	0	1	1	no
XLOC_002388	g6714	5,20976	4,01593	-0,375484	-0,320736	0,58315	0,784861	no
XLOC_002389	g6717	49,022	41,2576	-0,248769	-0,404653	0,48115	0,715676	no
XLOC_002390	g6719	22,4157	28,3556	0,339124	0,551578	0,32605	0,586932	no
XLOC_002391	g6720	23,8422	38,3109	0,684235	0,969931	0,08305	0,271381	no

XLOC_002392	g6721	212,628	167,945		-0,340343	-0,549397	0,3356	0,595606	no
XLOC_002393	g6722	5,9697	5,61141		-0,0892945	-0,0990994	0,86235	0,936559	no
XLOC_002394	g6723	4,1702	1,71511		-1,28182	-1,48801	0,01385	0,0839549	no
XLOC_002395	g6724	0	0,0493518	inf		0	1	1	no
XLOC_002396	g6725	43,2713	43,2711		-6,56E-06	-7,58E-06	0,98375	0,992238	no
XLOC_002397	g6726	83,04	136,808		0,720276	1,15747	0,0432	0,178545	no
XLOC_002398	g6727	19,781	4,93299		-2,00358	-2,83527	5,00E-05	0,00120049	yes
XLOC_002399	g6728	1,07357	2,08571		0,958128	0,853299	0,15825	0,398159	no
XLOC_002400	g6729	508,149	878,564		0,789896	1,24295	0,0269	0,13301	no
XLOC_002401	g6730	14,1387	15,6481		0,146336	0,211477	0,70525	0,860058	no
XLOC_002402	g6731	5,23499	9,41639		0,846988	1,25359	0,02895	0,138206	no
XLOC_002403	g6732	30,4256	69,6252		1,19432	1,95436	0,0008	0,0106019	yes
XLOC_002404	g6733	0	0,0290243	inf		0	1	1	no
XLOC_002405	g6734	399,843	197,569		-1,01708	-1,52459	0,0073	0,053577	no
XLOC_002406	g6735	1,62126	2,55643		0,657013	0,603253	0,29935	0,561625	no
XLOC_002407	g6736	0	0		0	0	1	1	no
XLOC_002408	g1707	100,796	127,801		0,342458	0,583605	0,30555	0,566867	no
XLOC_002409	g1710	102,344	103,549		0,0168836	0,0283094	0,95935	0,981846	no
XLOC_002410	g1711	55,6216	58,2986		0,0678168	0,106095	0,8555	0,934827	no
XLOC_002411	g1713	23,7567	18,5176		-0,359436	-0,592094	0,28675	0,549334	no
XLOC_002412	g1714	72,8685	74,462		0,0312098	0,0541453	0,92365	0,966359	no
XLOC_002413	g1708	20,0986	26,0135		0,372164	0,576527	0,3187	0,579009	no
XLOC_002414	g1709	482,604	281,688		-0,776738	-1,31623	0,0243	0,12409	no
XLOC_002415	g1712	51,0926	52,7661		0,0464956	0,0774638	0,8976	0,954356	no
XLOC_002416	g1715	32,729	54,486		0,735316	1,13641	0,0486	0,192907	no
XLOC_002417	g1716	39,5221	59,5268		0,590878	0,872759	0,11485	0,332398	no
XLOC_002418	g1717	2,45576	2,56198		0,0610918	0,0768595	0,8886	0,950447	no
XLOC_002419	g1718	43,8791	62,0008		0,498755	0,801169	0,16535	0,407974	no
XLOC_002420	g1721	65,0351	81,0446		0,317497	0,531009	0,3526	0,613272	no
XLOC_002421	g1723	28,5701	25,7684		-0,148903	-0,246287	0,6592	0,832477	no
XLOC_002422	g1719	28,6551	70,3557		1,29588	2,02805	0,00045	0,00669545	yes
XLOC_002423	g1720	1296,42	1351,89		0,0604449	0,103316	0,85575	0,934827	no
XLOC_002424	g1722	25,5147	36,5013		0,516621	0,852215	0,14115	0,375025	no
XLOC_002425	g1724	42,65	93,8925		1,13846	1,81042	0,00225	0,0229201	yes
XLOC_002426	g170	39,8432	94,4272		1,24487	2,01624	0,00065	0,00901554	yes
XLOC_002427	g172	41,0308	34,9031		-0,233353	-0,401555	0,4803	0,71557	no
XLOC_002428	g171	61,4723	51,5074		-0,255157	-0,41745	0,46505	0,705296	no
XLOC_002429	g173	55,208	38,6036		-0,516141	-0,777381	0,1764	0,423119	no
XLOC_002430	g6737	0	0		0	0	1	1	no
XLOC_002431	g6738	0	0		0	0	1	1	no
XLOC_002432	g1725	0	0		0	0	1	1	no
XLOC_002433	g6739	0	0		0	0	1	1	no
XLOC_002434	g6740	25,443	28,2115		0,149017	0,194726	0,72395	0,86956	no
XLOC_002435	g6741	39,1082	26,9213		-0,538725	-0,899809	0,1143	0,331588	no
XLOC_002436	g6742	0	0		0	0	1	1	no
XLOC_002437	g6743	0	0		0	0	1	1	no
XLOC_002438	g1727	6,05676	6,419		0,0838016	0,110385	0,8468	0,930362	no
XLOC_002439	g1732	36,5641	44,486		0,282923	0,414824	0,47275	0,710282	no
XLOC_002440	g1733	38,517	53,1239		0,463865	0,785527	0,1593	0,399777	no
XLOC_002441	g1736	7,40532	6,63423		-0,158633	-0,237846	0,6669	0,837544	no
XLOC_002442	g1738	80,2544	85,3267		0,0884165	0,137254	0,8025	0,908624	no
XLOC_002443	g1726	56,3394	69,6611		0,306208	0,516299	0,3706	0,629539	no
XLOC_002444	g1728	69,0979	51,4908		-0,424326	-0,61808	0,2733	0,534623	no
XLOC_002445	g1729,g1730	109,607	107,738		-0,0248126	-0,00935076	0,9709	0,986977	no
XLOC_002446	g1731	256,386	352,612		0,459765	0,688758	0,2426	0,500911	no
XLOC_002447	g1734	76,4999	81,4294		0,0900919	0,136018	0,8063	0,910937	no
XLOC_002448	g1735	41,9696	70,6692		0,751738	1,20619	0,03715	0,162067	no
XLOC_002449	g1737	155,745	177,559		0,189108	0,302256	0,5943	0,792155	no
XLOC_002450	g1739	44,9089	60,088		0,420077	0,677359	0,22585	0,483824	no
XLOC_002451	g6744	335,796	338,689		0,0123752	0,0163568	0,97435	0,987624	no
XLOC_002452	g6745	456,023	894,181		0,971461	1,38608	0,01625	0,0933187	no
XLOC_002453	g6747	32,3316	36,0378		0,156566	0,249364	0,66355	0,835468	no
XLOC_002454	g6749	36,4323	39,6989		0,123881	0,20288	0,7176	0,865764	no
XLOC_002455	g6751	3,14977	6,33547		1,0082	1,28026	0,03175	0,14654	no
XLOC_002456	g6753	35,8463	52,189		0,541924	0,893192	0,1161	0,334145	no
XLOC_002457	g6755	17,3753	19,6578		0,178065	0,289567	0,59715	0,794259	no
XLOC_002458	g6746	95,1605	72,773		-0,38696	-0,635382	0,26715	0,528062	no
XLOC_002459	g6748	47,5379	62,8089		0,401891	0,606642	0,29015	0,552935	no
XLOC_002460	g6750	56,4773	52,7172		-0,0993969	-0,167136	0,76365	0,888723	no
XLOC_002461	g6752	94,3976	91,5701		-0,0438738	-0,0723014	0,89995	0,955716	no
XLOC_002462	g6754	63,322	59,5662		-0,0882141	-0,150364	0,7847	0,900961	no
XLOC_002463	g6756	0	0		0	0	1	1	no
XLOC_002464	g6757	0	0		0	0	1	1	no
XLOC_002465	g6761	67,2364	72,3993		0,106733	0,169824	0,75475	0,884339	no
XLOC_002466	g6763	26,9568	23,0627		-0,225088	-0,320907	0,5706	0,777831	no
XLOC_002467	g6758	324,806	327,025		0,00982166	0,016541	0,97645	0,98853	no
XLOC_002468	g6759	230,347	241,861		0,0703649	0,116041	0,84005	0,926574	no
XLOC_002469	g6760	47,6056	45,9423		-0,0513081	-0,0881506	0,87645	0,944343	no
XLOC_002470	g6762	12,5734	13,2159		0,0719026	0,119108	0,83655	0,925387	no
XLOC_002471	g6764	25,2126	34,1508		0,437776	0,567971	0,3217	0,581892	no
XLOC_002472	g175	105,196	70,7468		-0,572342	-0,840481	0,15285	0,39078	no
XLOC_002473	g174	47,0278	134,65		1,51763	2,44018	5,00E-05	0,00120049	yes
XLOC_002474	g176	185,737	183,692		-0,0159772	-0,0223906	0,9674	0,985566	no
XLOC_002475	g6765	15,7321	17,0636		0,117212	0,192989	0,73515	0,874871	no
XLOC_002476	g6767	24,3542	28,6179		0,232749	0,402786	0,48175	0,716101	no
XLOC_002477	g6766	29,0198	49,3478		0,765949	1,19187	0,0369	0,161479	no
XLOC_002478	g6768	149,316	115,192		-0,374327	-0,593042	0,2949	0,556907	no
XLOC_002479	g6769	26,0481	22,0932		-0,237575	-0,405043	0,46875	0,707556	no
XLOC_002480	g6770	83,8695	33,2528		-1,33467	-1,79157	0,00295	0,0280164	yes
XLOC_002481	g6771	0	0		0	0	1	1	no
XLOC_002482	g6772	53,157	25,4307		-1,06369	-1,65826	0,00385	0,0340297	yes
XLOC_002483	g6773	175,385	29,6642		-2,56373	-3,36148	5,00E-05	0,00120049	yes

XLOC_002484	g6774	85,7056	52,808	-0,698632	-1,11059	0,05765	0,214952	no
XLOC_002485	g6775	10,6093	7,63397	-0,474824	-0,698769	0,2117	0,467272	no
XLOC_002486	g6776	37,4459	54,3681	0,537953	0,888606	0,1246	0,348001	no
XLOC_002487	g6777	95,9166	51,9608	-0,884357	-1,4135	0,01305	0,0807505	no
XLOC_002488	g178	56,9464	55,9817	-0,0246493	-0,0412495	0,9382	0,972361	no
XLOC_002489	g179	38,0626	34,5222	-0,140851	-0,241682	0,67205	0,840955	no
XLOC_002490	g177	102,547	90,3525	-0,182646	-0,30501	0,58255	0,784832	no
XLOC_002491	g180	12,8669	16,9047	0,393761	0,567096	0,3063	0,567308	no
XLOC_002492	g181	69,8961	79,8669	0,192386	0,328615	0,5602	0,771227	no
XLOC_002493	g182	123,719	122,404	-0,0154176	-0,0252191	0,9628	0,983635	no
XLOC_002494	g6778	0	0	0	0	1	1	no
XLOC_002495	g6779	39,3483	33,937	-0,213442	-0,355253	0,5374	0,756164	no
XLOC_002496	g6780	0	0	0	0	1	1	no
XLOC_002497	g6781	56,5371	86,1016	0,606843	1,00859	0,0723	0,24816	no
XLOC_002498	g6782	51,5317	47,5588	-0,115748	-0,19318	0,73395	0,874259	no
XLOC_002499	g6783	25,5038	41,1727	0,690977	1,1577	0,042	0,175432	no
XLOC_002500	g6784	0	0	0	0	1	1	no
XLOC_002501	g6785	0	0	0	0	1	1	no
XLOC_002502	g6786	26,443	24,6585	-0,100802	-0,150147	0,7925	0,904567	no
XLOC_002503	g6787	5,00536	2,28651	-1,13033	-1,36021	0,02295	0,119813	no
XLOC_002504	g6788	177,973	382,468	1,10368	1,53176	0,0138	0,0837035	no
XLOC_002505	g6789	12,979	14,0107	0,110348	0,147597	0,8027	0,908624	no
XLOC_002506	g1740	6,57925	5,30634	-0,310206	-0,402672	0,47585	0,712433	no
XLOC_002507	g1741	37,4443	54,5604	0,543108	0,891743	0,12155	0,343983	no
XLOC_002508	g1742	48,5572	100,829	1,05415	1,78424	0,0024	0,0240735	yes
XLOC_002509	g6790	64,4123	38,1235	-0,756654	-1,08795	0,0572	0,214227	no
XLOC_002510	g1743	201,609	348,946	0,791445	1,26913	0,0249	0,126105	no
XLOC_002511	g1745	61,6371	64,646	0,0687618	0,113159	0,83435	0,924287	no
XLOC_002512	g1746	100,363	188,136	0,90655	0,765155	0,15485	0,392851	no
XLOC_002513	g1747	74,1396	64,4424	-0,202233	-0,339833	0,55335	0,767027	no
XLOC_002514	g1744	96,0665	129,655	0,432575	0,756813	0,1918	0,442199	no
XLOC_002515	g1748	191,529	222,229	0,214485	0,300326	0,5869	0,787626	no
XLOC_002516	g6791	70,364	26,9093	-1,38673	-2,13081	0,0003	0,00496796	yes
XLOC_002517	g6793	101,673	37,9016	-1,42361	-2,04473	0,001	0,0126221	yes
XLOC_002518	g6794	147,886	152,059	0,0401498	0,0699486	0,90685	0,959723	no
XLOC_002519	g6795	11,7649	7,43393	-0,662297	-0,925049	0,1077	0,319291	no
XLOC_002520	g6792	20,4774	17,9589	-0,189339	-0,264443	0,6392	0,820902	no
XLOC_002521	g6796	37,561	28,5464	-0,395927	-0,541165	0,32855	0,5899	no
XLOC_002522	g6797	4,27671	8,92631	1,06156	1,20538	0,0399	0,169765	no
XLOC_002523	g6798	305,133	526,368	0,786634	1,20838	0,03825	0,165396	no
XLOC_002524	g6799	190,788	202,479	0,085802	0,137692	0,80275	0,908624	no
XLOC_002525	g1749	0	0	0	0	1	1	no
XLOC_002526	g6800	27856,2	3790,2	-2,87765	-6413,38	0,13475	0,364932	no
XLOC_002527	g6801	29,9315	41,2684	0,463372	0,755111	0,1972	0,448888	no
XLOC_002528	g6802	0,576851	0,290242	-0,990941	0	1	1	no
XLOC_002529	g6803	32,5558	32,4199	-0,00603736	-0,00706891	0,99145	0,995293	no
XLOC_002530	g6804	55,5611	58,7326	0,0800865	0,137292	0,8092	0,912324	no
XLOC_002531	g6805	11,6211	14,8952	0,358103	0,524733	0,3653	0,624956	no
XLOC_002532	g6806	156,86	102,28	-0,616949	-0,931117	0,09785	0,299902	no
XLOC_002533	g6807	11,3736	12,213	0,102735	0,100806	0,8554	0,934827	no
XLOC_002534	g6808	2305,34	802,87	-1,52174	-1,59703	0,01245	0,077872	no
XLOC_002535	g6810	130,946	116,922	-0,163432	-0,267589	0,6462	0,825616	no
XLOC_002536	g6809	26,6752	40,7328	0,610691	0,945122	0,08935	0,283129	no
XLOC_002537	g6811	28,099	62,2481	1,14751	1,78482	0,0024	0,0240735	yes
XLOC_002538	g6814	52,3241	30,2469	-0,790691	-1,30385	0,0219	0,116248	no
XLOC_002539	g6812	302,943	68,5729	-2,14333	-3,05951	5,00E-05	0,00120049	yes
XLOC_002540	g6813	168,313	105,054	-0,680014	-1,03691	0,0641	0,229647	no
XLOC_002541	g6815	2,22163	5,70407	1,36038	1,53215	0,01285	0,0799158	no
XLOC_002542	g6816	10,221	12,2561	0,261963	0,382766	0,5019	0,730642	no
XLOC_002543	g6817	9,28776	6,34437	-0,549854	-0,508653	0,38785	0,643448	no
XLOC_002544	g6818	4,83304	2,68516	-0,84792	-1,09436	0,05645	0,212798	no
XLOC_002545	g1751	103,099	97,0149	-0,0877503	-0,145498	0,7963	0,906216	no
XLOC_002546	g1750	207,463	192,051	-0,111365	-0,153537	0,7862	0,901303	no
XLOC_002547	g1752	199,096	154,148	-0,369146	-0,593039	0,30595	0,567194	no
XLOC_002548	g6819	17,6037	16,9551	-0,0541596	-0,0782175	0,89025	0,951176	no
XLOC_002549	g6820	19,5661	21,3098	0,123162	0,195564	0,73175	0,873287	no
XLOC_002550	g6821	49,7739	37,8763	-0,394093	-0,637083	0,267	0,527978	no
XLOC_002551	g6822	23,9276	25,8495	0,111462	0,188765	0,74015	0,876968	no
XLOC_002552	g6824	88,554	75,088	-0,237976	-0,381245	0,5091	0,735417	no
XLOC_002553	g6823	224,185	451,892	1,01129	1,64376	0,00415	0,0357482	yes
XLOC_002554	g6825	24,4341	13,1633	-0,892378	-1,3221	0,01605	0,0927124	no
XLOC_002555	g6826	0	0	0	0	1	1	no
XLOC_002556	g6829	67,6086	91,1963	0,431768	0,703228	0,20905	0,464031	no
XLOC_002557	g6827	50,3172	44,7342	-0,169673	-0,277595	0,63745	0,819769	no
XLOC_002558	g6828	9,72638	16,5518	0,76701	1,06724	0,0568	0,213462	no
XLOC_002559	g6830	101,768	128,224	0,333384	0,51937	0,3628	0,622826	no
XLOC_002560	g6831	0	0	0	0	1	1	no
XLOC_002561	g6832	382,588	365,085	-0,0675596	-0,103633	0,85775	0,935277	no
XLOC_002562	g6834	20,9941	46,853	1,15816	1,93527	0,0007	0,00954722	yes
XLOC_002563	g6835	24,9289	28,0315	0,169232	0,213142	0,71095	0,862876	no
XLOC_002564	g6833	167,811	261,474	0,639833	0,908589	0,11045	0,32396	no
XLOC_002565	g6836	0	0	0	0	1	1	no
XLOC_002566	g6837	27,5077	21,3793	-0,363621	-0,481721	0,38775	0,643411	no
XLOC_002567	g1753	1186,06	427,542	-1,47204	-1,82132	0,00625	0,0479867	yes
XLOC_002568	g1754	104,269	138,233	0,406797	0,694565	0,2228	0,480118	no
XLOC_002569	g6838	4,64203	5,99103	0,368046	0,505022	0,36405	0,623795	no
XLOC_002570	g6839	10,3819	7,03864	-0,560707	-0,644055	0,26435	0,524958	no
XLOC_002571	g6840	71,334	41,8146	-0,770583	-1,28119	0,02415	0,123967	no
XLOC_002572	g6841	4,51631	3,3178	-0,444917	-0,465492	0,41745	0,669473	no
XLOC_002573	g6842	4,46731	5,98181	0,421173	0,419508	0,44695	0,693154	no
XLOC_002574	g6843	0	0	0	0	1	1	no
XLOC_002575	g6844	0	0	0	0	1	1	no

XLOC_002576	g6845	0	0	0	0	1	1	no
XLOC_002577	g1755	22,686	24,3066	0,0995484	0,133212	0,816	0,916518	no
XLOC_002578	g1756	452,228	704,845	0,640256	1,0948	0,0508	0,198827	no
XLOC_002579	g6846	276,873	117,498	-1,23659	-1,22359	0,038	0,164605	no
XLOC_002580	g6847	8,45303	4,29571	-0,976571	-1,06477	0,06535	0,231841	no
XLOC_002581	g6848	189,999	173,444	-0,13152	-0,207446	0,70355	0,859311	no
XLOC_002582	g6851	5,02218	5,77683	0,201967	0,282544	0,61065	0,804155	no
XLOC_002583	g6853	32,023	14,0751	-1,18596	-1,61376	0,0063	0,0482574	yes
XLOC_002584	g6849	142,79	127,687	-0,16128	-0,273438	0,6351	0,817604	no
XLOC_002585	g6850	16,7159	65,6288	1,97311	2,75723	5,00E-05	0,00120049	yes
XLOC_002586	g6852	33,9592	34,7437	0,0329478	0,0530893	0,9231	0,96635	no
XLOC_002587	g6854	0	0	0	0	1	1	no
XLOC_002588	g6855	0	0	0	0	1	1	no
XLOC_002589	g1758	50,9325	46,0906	-0,144114	-0,248011	0,66395	0,835468	no
XLOC_002590	g1760	63,0857	66,6741	0,0798139	0,127914	0,82795	0,921598	no
XLOC_002591	g1761	78,8591	118,833	0,591584	0,952212	0,08965	0,283805	no
XLOC_002592	g1757	119,852	68,9234	-0,798194	-1,21051	0,02885	0,137997	no
XLOC_002593	g1759	643,121	369,352	-0,800095	-1,14125	0,0527	0,203665	no
XLOC_002594	g1762	8,17379	7,41824	-0,139929	-0,183062	0,75315	0,883683	no
XLOC_002595	g1763	0	0	0	0	1	1	no
XLOC_002596	g6856	0	0	0	0	1	1	no
XLOC_002597	g6857	28,9656	15,6846	-0,884994	-1,3866	0,0131	0,0810088	no
XLOC_002598	g6859	7,87647	13,5864	0,786542	1,06286	0,0721	0,24756	no
XLOC_002599	g6858	171,205	513,129	1,5836	2,38889	5,00E-05	0,00120049	yes
XLOC_002600	g6860	2,8753	6,82134	1,24634	1,27101	0,03775	0,163956	no
XLOC_002601	g6862	46,0278	29,7735	-0,628479	-1,0527	0,069	0,240789	no
XLOC_002602	g6861	27,5792	34,1541	0,308478	0,519691	0,3513	0,611769	no
XLOC_002603	g6863	47,4101	54,5794	0,203162	0,341333	0,54605	0,762544	no
XLOC_002604	g6864	1,67464	14,7897	3,14267	2,21494	0,00755	0,05496	no
XLOC_002605	g6865	426,271	187,5	-1,18488	-1,90609	0,0011	0,0134856	yes
XLOC_002606	g6866	23,0036	17,3122	-0,410069	-0,60889	0,27675	0,538359	no
XLOC_002607	g6867	72,856	30,8006	-1,24209	-2,00942	0,00035	0,00552572	yes
XLOC_002608	g6868	56,3985	68,3036	0,276304	0,474943	0,3981	0,652209	no
XLOC_002609	g6869	63,7656	53,9355	-0,241543	-0,407484	0,47715	0,713292	no
XLOC_002610	g6870	42,0162	45,6168	0,11862	0,202473	0,71625	0,865564	no
XLOC_002611	g6872	45,1429	77,7859	0,785009	1,30764	0,02015	0,109443	no
XLOC_002612	g6874	49,3734	50,5842	0,0349525	0,0584084	0,91495	0,963526	no
XLOC_002613	g6875	165,332	149,741	-0,142896	-0,213339	0,698	0,85623	no
XLOC_002614	g6877	4052,01	4152,25	0,0352566	0,0338702	0,9555	0,980512	no
XLOC_002615	g6879	46,5848	64,6454	0,472689	0,8094	0,1475	0,384102	no
XLOC_002616	g6881	192,122	197,002	0,0361924	0,0600383	0,9193	0,964479	no
XLOC_002617	g6883	92,8719	85,4043	-0,120934	-0,208305	0,71995	0,867473	no
XLOC_002618	g6885	59,6342	60,6134	0,0234986	0,0368041	0,9491	0,97711	no
XLOC_002619	g6871	69,1707	43,8954	-0,656093	-0,898147	0,1165	0,334707	no
XLOC_002620	g6873	209,066	211,742	0,0183523	0,0309815	0,95555	0,980512	no
XLOC_002621	g6876	35,2969	57,8155	0,711914	1,09904	0,04865	0,193027	no
XLOC_002622	g6878	72,8617	143,202	0,97482	1,64149	0,00405	0,0351645	yes
XLOC_002623	g6880	39,8013	60,5973	0,606439	1,04083	0,068	0,238827	no
XLOC_002624	g6882	66,753	54,7923	-0,28486	-0,458638	0,43115	0,678624	no
XLOC_002625	g6884	65,3301	181,199	1,47176	2,32714	5,00E-05	0,00120049	yes
XLOC_002626	g6886	0	0	0	0	1	1	no
XLOC_002627	g6887	13,3646	8,23463	-0,69864	-0,884925	0,1396	0,372621	no
XLOC_002628	g6888	0,484961	3,12354	2,68724	1,67285	0,25135	0,510076	no
XLOC_002629	g6889	0,0225645	0,140686	2,64035	0	1	1	no
XLOC_002630	g6890	0	0	0	0	1	1	no
XLOC_002631	g1764	0	0	0	0	1	1	no
XLOC_002632	g6891	0	0,0249535	inf	0	1	1	no
XLOC_002633	g1765	27,947	158,902	2,50737	3,80855	5,00E-05	0,00120049	yes
XLOC_002634	g6892	25,22	6,42926	-1,97184	-2,24782	0,0007	0,00954722	yes
XLOC_002635	g6893	6,93622	9,55985	0,462837	0,574112	0,31805	0,578273	no
XLOC_002636	g6894	42,2607	43,296	0,0349141	0,0603133	0,9186	0,964054	no
XLOC_002637	g1767	0	0,0205154	inf	0	1	1	no
XLOC_002638	g1768	2,86422	1,61877	-0,823241	-0,819019	0,1581	0,398088	no
XLOC_002639	g1766	0	0	0	0	1	1	no
XLOC_002640	g1769	72,387	102,104	0,496242	0,791901	0,1641	0,406319	no
XLOC_002641	g183	26,546	24,0845	-0,140394	-0,212062	0,69815	0,85623	no
XLOC_002642	g185	69,0967	46,6174	-0,567749	-0,910351	0,1109	0,324892	no
XLOC_002643	g184	4,77858	11,1057	1,21664	1,1854	0,05345	0,205352	no
XLOC_002644	g6895	62,7909	24,0274	-1,38587	-1,97123	0,0011	0,0134856	yes
XLOC_002645	g1770	0	0	0	0	1	1	no
XLOC_002646	g1771	0	0	0	0	1	1	no
XLOC_002647	g1773	10,8316	10,1549	-0,0930727	-0,138721	0,80295	0,908624	no
XLOC_002648	g1775	97,0538	80,4101	-0,271408	-0,416524	0,46595	0,706334	no
XLOC_002649	g1777	51,731	43,573	-0,247595	-0,402695	0,4644	0,705296	no
XLOC_002650	g1772	113,555	189,977	0,742435	1,06625	0,0543	0,2074	no
XLOC_002651	g1774	1,93524	0,945171	-1,03387	-0,764813	0,2555	0,514986	no
XLOC_002652	g1776	52,8858	97,6564	0,884835	1,50426	0,0113	0,0724321	no
XLOC_002653	g6896	5,0059	3,04609	-0,716669	-0,705149	0,23455	0,492049	no
XLOC_002654	g6897	15,2827	6,8261	-1,16277	-1,39688	0,0186	0,103252	no
XLOC_002655	g6898	2,81068	2,1254	-0,403188	-0,502945	0,3874	0,643156	no
XLOC_002656	g6900	0	0	0	0	1	1	no
XLOC_002657	g6899	0	0	0	0	1	1	no
XLOC_002658	g1778	40,493	40,9559	0,0164006	0,0263547	0,96365	0,983889	no
XLOC_002659	g1780	5,61018	5,45483	-0,0405117	-0,0558225	0,92065	0,96497	no
XLOC_002660	g1779	147,631	100,496	-0,554851	-0,929784	0,0991	0,301943	no
XLOC_002661	g6901	12714,2	29370,4	1,20793	1,44385	0,00985	0,0657113	no
XLOC_002662	g6902	0,622369	1,93159	1,63395	1,266	0,05185	0,201172	no
XLOC_002663	g6903	37,3121	24,3011	-0,618619	-0,74738	0,1866	0,434839	no
XLOC_002664	g6904	1,02733	0	#NAME?	0	1	1	no
XLOC_002665	g6905	1,92545	5,92211	1,62091	1,8071	0,0036	0,0323144	yes
XLOC_002666	g6906	0,75777	2,60173	1,77964	1,63433	0,0143	0,08594	no
XLOC_002667	g1781	43,69	16,2817	-1,42406	-1,73295	0,0053	0,0425908	yes

XLOC_002668	g6907	0	0	0	0	1	1	no
XLOC_002669	g186	895,362	402,959	-1,15184	-1,82628	0,00185	0,01964	yes
XLOC_002670	g187	171,25	196,836	0,200892	0,341154	0,55315	0,766897	no
XLOC_002671	g6908	0	0	0	0	1	1	no
XLOC_002672	g6910	87,1276	82,8468	-0,0726837	-0,0914164	0,87515	0,943667	no
XLOC_002673	g6909	4,57552	6,26962	0,454443	0,428214	0,4419	0,688366	no
XLOC_002674	g1782	69,6307	41,5467	-0,74499	-1,22511	0,0345	0,154557	no
XLOC_002675	g1785	17,942	16,6091	-0,111367	-0,172456	0,77	0,893264	no
XLOC_002676	g1786	5,52365	2,80176	-0,979288	-1,05141	0,12345	0,347009	no
XLOC_002677	g1788	359,698	275,836	-0,382978	-0,666273	0,24415	0,503031	no
XLOC_002678	g1789	18,4172	10,1312	-0,862252	-1,37617	0,0151	0,0888981	no
XLOC_002679	g1790	18,5799	12,4926	-0,572671	-0,756674	0,1781	0,424913	no
XLOC_002680	g1791	2,24882	3,71241	0,723191	0,828531	0,1609	0,40225	no
XLOC_002681	g1792	84,4125	142,947	0,759954	1,21142	0,0357	0,158066	no
XLOC_002682	g1783	107,079	83,9805	-0,350548	-0,556395	0,31565	0,576471	no
XLOC_002683	g1784	6,43061	4,84288	-0,409089	-0,470444	0,41065	0,663116	no
XLOC_002684	g1787	31,5684	36,4585	0,207778	0,349473	0,53305	0,752918	no
XLOC_002685	g1793	39,7449	79,1821	0,994405	1,5896	0,0057	0,0448151	yes
XLOC_002686	g6911	43,2504	36,408	-0,248458	-0,404299	0,4701	0,708034	no
XLOC_002687	g6912	236,001	198,478	-0,249812	-0,419364	0,45175	0,696605	no
XLOC_002688	g6913	8,1459	6,79416	-0,26178	-0,352104	0,528	0,74938	no
XLOC_002689	g6914	50,0613	40,0945	-0,320292	-0,520221	0,36585	0,625461	no
XLOC_002690	g6915	12,1425	12,9146	0,0889378	0,118476	0,8382	0,926315	no
XLOC_002691	g1794	1,98125	2,94983	0,574224	0,820195	0,13955	0,372621	no
XLOC_002692	g1798	2,81101	2,25497	-0,317979	-0,307987	0,58875	0,789071	no
XLOC_002693	g1800	7,71437	3,25226	-1,24611	-1,31452	0,03575	0,158066	no
XLOC_002694	g1803	2,31024	1,18415	-0,964188	-0,823102	0,1455	0,381831	no
XLOC_002695	g1795	16,5601	26,1614	0,659731	1,0517	0,06495	0,231007	no
XLOC_002696	g1796	13,75	16,9695	0,303513	0,415264	0,45995	0,701766	no
XLOC_002697	g1797	1,83431	10,6016	2,53097	2,28174	0,0012	0,0143358	yes
XLOC_002698	g1799	29,6182	29,2859	-0,0162802	-0,0266781	0,96455	0,984142	no
XLOC_002699	g1801	30,7814	13,4589	-1,1935	-1,79521	0,00155	0,0171215	yes
XLOC_002700	g1802	210,273	145,665	-0,529615	-0,906017	0,11305	0,329226	no
XLOC_002701	g1804	136,995	36,3427	-1,91439	-2,70888	5,00E-05	0,00120049	yes
XLOC_002702	g1805	260,839	92,9664	-1,48838	-2,0613	0,00045	0,00669545	yes
XLOC_002703	g1806	126,716	163	0,363271	0,619782	0,2663	0,527231	no
XLOC_002704	g1807	93,8475	66,4574	-0,497888	-0,829639	0,1498	0,387142	no
XLOC_002705	g1808	132,393	487,967	1,88195	2,79222	5,00E-05	0,00120049	yes
XLOC_002706	g1809	24,8552	39,0305	0,651051	1,00367	0,079	0,262088	no
XLOC_002707	g6916	76,9222	79,8181	0,0533168	0,0865493	0,87945	0,945715	no
XLOC_002708	g1810	66,4967	75,9292	0,191372	0,329795	0,57045	0,777831	no
XLOC_002709	g1811	51,8239	56,5959	0,127081	0,205633	0,71295	0,864128	no
XLOC_002710	g1812	30,196	19,6994	-0,616206	-1,02237	0,06775	0,238119	no
XLOC_002711	g1814	23,5053	16,8565	-0,479675	-0,746646	0,19205	0,442199	no
XLOC_002712	g1819	46,7538	35,5797	-0,394027	-0,669306	0,24515	0,503845	no
XLOC_002713	g1822	82,425	264,726	1,68335	2,45434	0,0001	0,00209829	yes
XLOC_002714	g1813	16,9012	12,5641	-0,427819	-0,682643	0,2278	0,485987	no
XLOC_002715	g1815	134,845	152,055	0,173291	0,29553	0,6048	0,79937	no
XLOC_002716	g1816	50,0636	34,7067	-0,528548	-0,860893	0,11715	0,335495	no
XLOC_002717	g1817	51,2406	35,2469	-0,539794	-0,871835	0,1234	0,347009	no
XLOC_002718	g1818	120,941	111,742	-0,114128	-0,188591	0,7391	0,876777	no
XLOC_002719	g1820	77,9232	86,9782	0,1586	0,244305	0,66215	0,834807	no
XLOC_002720	g1821	0,0913497	0,0866673	-0,0759122	0	1	1	no
XLOC_002721	g1823	210,967	267,85	0,344407	0,537728	0,3554	0,616046	no
XLOC_002722	g6918	59,5956	85,8178	0,526071	0,860629	0,13	0,357191	no
XLOC_002723	g6917	74,0936	22,3092	-1,73171	-2,85702	5,00E-05	0,00120049	yes
XLOC_002724	g6919	34,0346	20,5369	-0,72878	-1,17286	0,04075	0,171966	no
XLOC_002725	g6921	99,9286	137,093	0,456189	0,769456	0,17425	0,420013	no
XLOC_002726	g6920	39,4928	32,2321	-0,293093	-0,493954	0,37265	0,631044	no
XLOC_002727	g6922	97,2144	64,6665	-0,588152	-1,00126	0,076	0,254891	no
XLOC_002728	g6923	72,3202	106,993	0,565046	0,971414	0,08865	0,281729	no
XLOC_002729	g6924	296,009	311,349	0,072893	0,103321	0,85235	0,933639	no
XLOC_002730	g6925	530,376	73,8276	-2,84478	-4,28213	5,00E-05	0,00120049	yes
XLOC_002731	g6927	150,585	111,206	-0,437352	-0,658176	0,25535	0,514895	no
XLOC_002732	g6926	50,0333	26,6587	-0,90828	-1,48911	0,00985	0,0657113	no
XLOC_002733	g6928	1374,83	720,776	-0,931633	-1,53885	0,00735	0,0538233	no
XLOC_002734	g6932	195,171	450,021	1,20525	1,99992	0,00025	0,00430702	yes
XLOC_002735	g6933	91,3821	91,0802	-0,00477419	-0,00822755	0,9892	0,99472	no
XLOC_002736	g6934	0,94392	1,82832	0,953783	0	1	1	no
XLOC_002737	g6936	4,68229	3,84202	-0,285349	-0,39783	0,4995	0,728948	no
XLOC_002738	g6929	28,2521	15,0389	-0,909661	-1,43332	0,00945	0,0639552	no
XLOC_002739	g6930	7,85892	8,9162	0,182098	0,285402	0,62235	0,810433	no
XLOC_002740	g6931	30,8747	17,6033	-0,81058	-1,37596	0,018	0,100603	no
XLOC_002741	g6935	165,815	162,643	-0,0278684	-0,0463544	0,9344	0,971293	no
XLOC_002742	g6937	25,7149	37,314	0,537109	0,777726	0,1854	0,433586	no
XLOC_002743	g6938	0	0,0183873	inf	0	1	1	no
XLOC_002744	g6939	55,657	61,8426	0,152037	0,25194	0,6557	0,831391	no
XLOC_002745	g6940	238,416	360,788	0,597667	0,977828	0,09685	0,29786	no
XLOC_002746	g6941	46,5056	31,4615	-0,563817	-0,936049	0,0985	0,300862	no
XLOC_002747	g6943	44,6228	41,9163	-0,0902681	-0,130096	0,8142	0,915544	no
XLOC_002748	g6942	16,4511	13,4159	-0,294249	-0,493823	0,38825	0,643479	no
XLOC_002749	g6945	11,9261	19,9705	0,743748	1,08927	0,0575	0,214859	no
XLOC_002750	g6944	3,3943	2,72874	-0,314879	-0,390783	0,48165	0,716101	no
XLOC_002751	g6946	162,548	153,377	-0,0837818	-0,136995	0,813	0,914613	no
XLOC_002752	g6947	0	0	0	0	1	1	no
XLOC_002753	g6948	53,7655	62,2232	0,210772	0,314019	0,5761	0,780604	no
XLOC_002754	g6949	0	0	0	0	1	1	no
XLOC_002755	g6950	2,50356	2,13321	-0,230959	-0,232648	0,68305	0,847555	no
XLOC_002756	g6951	0	0	0	0	1	1	no
XLOC_002757	g6952	32,2973	12,8098	-1,33416	-2,01778	0,0015	0,0168343	yes
XLOC_002758	g6953	93,4815	104,852	0,165602	0,276082	0,6246	0,811943	no
XLOC_002759	g6955	77,1483	83,5059	0,114245	0,175975	0,7523	0,88326	no

XLOC_002760	g6956	41,3152	48,0089	0,216629	0,367038	0,52215	0,745347	no
XLOC_002761	g6954	223,967	270,92	0,274577	0,455787	0,41795	0,669829	no
XLOC_002762	g6957	54,1061	71,2595	0,39729	0,637753	0,24115	0,499282	no
XLOC_002763	g1825	34,1484	14,7811	-1,20807	-1,48086	0,01575	0,0915719	no
XLOC_002764	g1824	15,2241	12,4756	-0,287244	-0,484118	0,3987	0,65273	no
XLOC_002765	g6958	0	0	0	0	1	1	no
XLOC_002766	g1826	30,5577	24,0118	-0,347791	-0,536041	0,34445	0,604645	no
XLOC_002767	g6959	27,7345	36,2847	0,387682	0,593571	0,30505	0,566474	no
XLOC_002768	g6960	4,40445	2,65007	-0,732932	-0,82587	0,1588	0,399236	no
XLOC_002769	g6961	113,522	64,7943	-0,809029	-1,3687	0,01865	0,103412	no
XLOC_002770	g6962	90,0711	78,9744	-0,18968	-0,317225	0,5747	0,780319	no
XLOC_002771	g6963	55,2206	129,89	1,23402	1,95266	0,0004	0,0061761	yes
XLOC_002772	g6964	5,38203	3,76171	-0,516763	-0,590833	0,2903	0,552994	no
XLOC_002773	g6965	0	0	0	0	1	1	no
XLOC_002774	g6966	0	0	0	0	1	1	no
XLOC_002775	g6968	132,616	99,3867	-0,416134	-0,699576	0,21765	0,474223	no
XLOC_002776	g6967	58,0011	59,1844	0,0291361	0,049399	0,9292	0,968451	no
XLOC_002777	g6969	31,3805	40,8208	0,379433	0,631327	0,27575	0,537479	no
XLOC_002778	g189	4,42674	4,03125	-0,135016	-0,17459	0,75165	0,883025	no
XLOC_002779	g188	7,96895	8,89115	0,157979	0,217769	0,6997	0,857274	no
XLOC_002780	g190	17,9805	48,19	1,4223	2,16356	0,00045	0,00669545	yes
XLOC_002781	g6970	120,633	86,1227	-0,486159	-0,815835	0,14725	0,384102	no
XLOC_002782	g6971	509,395	588,305	0,207778	0,338702	0,54365	0,76125	no
XLOC_002783	g6974	23,4006	41,5501	0,828307	1,33558	0,0231	0,120468	no
XLOC_002784	g6976	1112,36	598,86	-0,893329	-1,42737	0,011	0,0711126	no
XLOC_002785	g6972	26,6813	54,5422	1,03154	1,07288	0,05845	0,216433	no
XLOC_002786	g6973	21,1982	15,5607	-0,446035	-0,733508	0,2002	0,45251	no
XLOC_002787	g6975	13,9694	16,2092	0,214547	0,340679	0,54755	0,76377	no
XLOC_002788	g6977	10,6141	10,6649	0,00688713	0,0094933	0,98425	0,992379	no
XLOC_002789	g6978	0	0	0	0	1	1	no
XLOC_002790	g6979	0	0	0	0	1	1	no
XLOC_002791	g1827	88,1633	40,0954	-1,13674	-1,73383	0,0023	0,0233326	yes
XLOC_002792	g1828	1,31609	0,413884	-1,66896	-1,12711	0,07555	0,253989	no
XLOC_002793	g6980	0	0	0	0	1	1	no
XLOC_002794	g6982	557,432	518,377	-0,104792	-0,122301	0,831	0,92229	no
XLOC_002795	g6981	65,926	69,858	0,0835771	0,137222	0,81585	0,916455	no
XLOC_002796	g1829	0,0629669	0,10832	0,782635	0	1	1	no
XLOC_002797	g1830	83,5583	74,0126	-0,175012	-0,293301	0,60915	0,802826	no
XLOC_002798	g1831	125,049	111,229	-0,168954	-0,281686	0,61795	0,808291	no
XLOC_002799	g6983	11,5029	3,89422	-1,56259	-1,38385	0,25415	0,513318	no
XLOC_002800	g6984	18,2235	42,2328	1,21256	1,98207	0,0006	0,00845337	yes
XLOC_002801	g1834	27,6369	38,8137	0,489969	0,80743	0,15815	0,398112	no
XLOC_002802	g1835	32,1648	40,2332	0,322906	0,521524	0,3656	0,62536	no
XLOC_002803	g1836	73,653	74,1329	0,0093704	0,0146	0,98005	0,990233	no
XLOC_002804	g1838	7,86527	21,2396	1,43319	1,45472	0,01585	0,0918813	no
XLOC_002805	g1832	73,3007	74,8387	0,0299586	0,0505722	0,92935	0,968504	no
XLOC_002806	g1833	15,085	22,4584	0,574142	0,805889	0,137	0,369295	no
XLOC_002807	g1837	21,144	25,0885	0,246776	0,417029	0,46735	0,707046	no
XLOC_002808	g6985	14,3219	15,3733	0,102213	0,135055	0,8112	0,913216	no
XLOC_002809	g1839	887,928	2068,42	1,22002	1,21384	0,04035	0,170865	no
XLOC_002810	g1840	38,4651	24,0355	-0,678386	-1,15021	0,03885	0,166524	no
XLOC_002811	g1841	365,085	690,04	0,918448	1,51889	0,00865	0,0602433	no
XLOC_002812	g6987	3,85377	8,92351	1,21134	1,20363	0,0424	0,176802	no
XLOC_002813	g6986	45,0625	53,3857	0,244526	0,384744	0,5008	0,730196	no
XLOC_002814	g1843	88,347	120,48	0,447547	0,76557	0,17145	0,41633	no
XLOC_002815	g1844	1,40296	1,01753	-0,463404	-0,314299	0,67585	0,843051	no
XLOC_002816	g1846	15,7314	4,72764	-1,73445	-2,4029	0,00015	0,00294012	yes
XLOC_002817	g1842	40,4591	50,4599	0,318673	0,535368	0,35125	0,611769	no
XLOC_002818	g1845	65,6719	75,1713	0,194905	0,326265	0,56375	0,773404	no
XLOC_002819	g6988	172,776	404,311	1,22656	2,08495	0,00035	0,00552572	yes
XLOC_002820	g6989	9,10561	16,1643	0,827984	1,20011	0,03885	0,166524	no
XLOC_002821	g6994	237,743	413,866	0,79976	1,31144	0,0254	0,127912	no
XLOC_002822	g6990	29,7142	34,1242	0,199643	0,314107	0,5877	0,78831	no
XLOC_002823	g6991	313,755	266,712	-0,234355	-0,376362	0,4948	0,725323	no
XLOC_002824	g6992	1235,13	918,757	-0,426912	-0,692993	0,2245	0,482299	no
XLOC_002825	g6993	11268,3	16016,8	0,507311	0,443894	0,42705	0,67661	no
XLOC_002826	g6995	651,576	1274,73	0,968188	1,49232	0,0119	0,0753436	no
XLOC_002827	g6996	35,2553	42,6421	0,27444	0,460048	0,42265	0,672787	no
XLOC_002828	g6997	0	0	0	0	1	1	no
XLOC_002829	g6998	0	0	0	0	1	1	no
XLOC_002830	g6999	8,83039	6,239	-0,501162	-0,449338	0,41505	0,667287	no
XLOC_002831	g7000	36,1178	38,14	0,0785967	0,122448	0,82555	0,920778	no
XLOC_002832	g7001	2,07247	11,886	2,51984	3,22344	5,00E-05	0,00120049	yes
XLOC_002833	g1848	677,359	705,17	0,0580492	0,0819776	0,88065	0,946479	no
XLOC_002834	g1850	75,1312	59,7902	-0,329503	-0,558485	0,3138	0,574481	no
XLOC_002835	g1852	38,7054	29,3292	-0,400196	-0,682031	0,2216	0,478582	no
XLOC_002836	g1847	0,22503	0,255175	0,181372	0	1	1	no
XLOC_002837	g1849	22,477	17,0065	-0,40236	-0,583914	0,30725	0,568181	no
XLOC_002838	g1851	14,8176	23,9086	0,690217	0,919621	0,1101	0,32337	no
XLOC_002839	g1853	20,8494	34,2234	0,714977	1,04311	0,0687	0,240083	no
XLOC_002840	g7003	5,22423	8,8142	0,75461	0,74436	0,2094	0,464283	no
XLOC_002841	g7002	6,261	5,92505	-0,079566	-0,128763	0,89145	0,951423	no
XLOC_002842	g7004	19,593	7,71122	-1,34531	-2,01864	0,0006	0,00845337	yes
XLOC_002843	g7005	12,1687	9,59856	-0,342286	-0,495165	0,3823	0,638793	no
XLOC_002844	g7006	6,52934	5,66176	-0,205687	-0,247925	0,66175	0,834624	no
XLOC_002845	g7007	10,2298	7,50437	-0,446972	-0,504276	0,38695	0,643156	no
XLOC_002846	g7008	0	0	0	0	1	1	no
XLOC_002847	g7009	0,161041	0	#NAME?	0	1	1	no
XLOC_002848	g7010	70,0957	83,7333	0,256475	0,416399	0,46925	0,707556	no
XLOC_002849	g7012	365,039	342,077	-0,0937309	-0,0980683	0,8431	0,928374	no
XLOC_002850	g7011	34,7943	99,2952	1,51287	2,44206	5,00E-05	0,00120049	yes
XLOC_002851	g7013	0	0,933748	inf	0	1	1	no

XLOC_002852	g7014	0,0176074	0	#NAME?	0	1	1	no
XLOC_002853	g7015	0	0	0	0	1	1	no
XLOC_002854	g1854	0	0	0	0	1	1	no
XLOC_002855	g1855	7,93388	1,10209	-2,84778	-2,33784	0,0388	0,166524	no
XLOC_002856	g1856	59,1489	48,0738	-0,299102	-0,501154	0,37855	0,636208	no
XLOC_002857	g7017	35,0201	23,9195	-0,549995	-0,802463	0,1676	0,410842	no
XLOC_002858	g7019	65,8272	28,8419	-1,19052	-1,8754	0,00155	0,0171215	yes
XLOC_002859	g7020	22,0031	20,3584	-0,112088	-0,175207	0,75785	0,8854	no
XLOC_002860	g7016	96,625	45,1548	-1,09752	-1,77172	0,00165	0,0180837	yes
XLOC_002861	g7018	43,4851	140,136	1,68823	2,77533	5,00E-05	0,00120049	yes
XLOC_002862	g7021	23,7759	8,6547	-1,45795	-1,31951	0,02725	0,133597	no
XLOC_002863	g1857	3,52373	4,38774	0,316372	0,468558	0,41375	0,666012	no
XLOC_002864	g7022	0	0,0525268	inf	0	1	1	no
XLOC_002865	g1858	73,6891	28,2964	-1,38083	-2,08163	0,00045	0,00669545	yes
XLOC_002866	g1861	23,6508	25,0577	0,0833632	0,124759	0,8268	0,921064	no
XLOC_002867	g1864	22,0448	21,2021	-0,0562282	-0,0798156	0,88435	0,948388	no
XLOC_002868	g1859	0,147641	0,132676	-0,15419	0	1	1	no
XLOC_002869	g1860	1,26229	1,47434	0,224027	0,267927	0,6347	0,817196	no
XLOC_002870	g1862	14,9435	37,25	1,31772	1,68926	0,00445	0,0377692	yes
XLOC_002871	g1863	57,8455	37,4377	-0,627713	-0,923821	0,11015	0,32337	no
XLOC_002872	g1865	8,49905	9,50622	0,16157	0,225037	0,6972	0,856211	no
XLOC_002873	g7023	31,4077	56,1094	0,837121	1,22409	0,03335	0,151479	no
XLOC_002874	g7024	0	0	0	0	1	1	no
XLOC_002875	g7025	0	0	0	0	1	1	no
XLOC_002876	g7026	0	0	0	0	1	1	no
XLOC_002877	g1866	42,9126	39,9422	-0,103485	-0,176513	0,7533	0,883694	no
XLOC_002878	g7027	0,0808281	3,74871	5,53539	1,42974	0,12375	0,347009	no
XLOC_002879	g1869	53,0375	70,9065	0,418906	0,6588	0,2392	0,497337	no
XLOC_002880	g1867	50,0726	31,7465	-0,657424	-1,03442	0,06525	0,231738	no
XLOC_002881	g1868	118,174	34,5543	-1,77398	-2,46607	5,00E-05	0,00120049	yes
XLOC_002882	g1870	9,80235	14,1713	0,53177	0,846062	0,1412	0,375057	no
XLOC_002883	g1871	21,3511	18,5828	-0,200346	-0,324482	0,55875	0,770744	no
XLOC_002884	g7028	1,58445	1,74698	0,140873	0,112474	0,84745	0,930764	no
XLOC_002885	g7029	7,46905	8,59444	0,202479	0,249516	0,6578	0,8321	no
XLOC_002886	g7030	9,92388	10,8045	0,122652	0,148466	0,7909	0,903527	no
XLOC_002887	g7031	4,53802	7,73312	0,768986	0,585174	0,49405	0,724872	no
XLOC_002888	g7032	1,78386	0,565051	-1,65855	0	1	1	no
XLOC_002889	g7033	19,5943	32,3118	0,721625	1,07424	0,06035	0,221051	no
XLOC_002890	g7034	19,0021	25,3381	0,415154	0,660756	0,24075	0,49898	no
XLOC_002891	g7035	29,2222	18,528	-0,657357	-1,04158	0,0657	0,232663	no
XLOC_002892	g1873	61,8772	36,7281	-0,752525	-1,22204	0,0338	0,152886	no
XLOC_002893	g1874	4,5864	6,80829	0,569932	0,815916	0,14585	0,38234	no
XLOC_002894	g1876	92,4436	63,0258	-0,55263	-0,853435	0,13015	0,357303	no
XLOC_002895	g1877	98,0725	66,5842	-0,558668	-0,94871	0,09405	0,29264	no
XLOC_002896	g1878	244,145	349,069	0,515773	0,787694	0,1672	0,410619	no
XLOC_002897	g1872	89,3273	50,9788	-0,809204	-1,31058	0,0159	0,0920625	no
XLOC_002898	g1875	132,119	38,2875	-1,78689	-2,39954	0,0001	0,00209829	yes
XLOC_002899	g7036	20,0236	29,8429	0,57569	0,685191	0,22015	0,477349	no
XLOC_002900	g7037	94,2539	68,496	-0,460533	-0,744158	0,18255	0,42989	no
XLOC_002901	g7038	18,7194	19,0533	0,0255069	0,0334612	0,95675	0,980821	no
XLOC_002902	g7039	23,7676	25,7032	0,112952	0,177747	0,7577	0,8854	no
XLOC_002903	g7040	74,68	3806,31	5,67153	7,18739	5,00E-05	0,00120049	yes
XLOC_002904	g7041	0	0	0	0	1	1	no
XLOC_002905	g7042	0	0	0	0	1	1	no
XLOC_002906	g7043	12,0074	10,6478	-0,17337	-0,233574	0,6639	0,835468	no
XLOC_002907	g7044	0	0	0	0	1	1	no
XLOC_002908	g7045	1989,26	1234,13	-0,68874	-1,03839	0,07565	0,254064	no
XLOC_002909	g7046	90,2162	98,0208	0,119702	0,185135	0,74545	0,880058	no
XLOC_002910	g7047	0	0	0	0	1	1	no
XLOC_002911	g7048	47,6206	21,7884	-1,12802	-1,89063	0,00105	0,0130684	yes
XLOC_002912	g7049	0	0,197963	inf	0	1	1	no
XLOC_002913	g7050	10,3282	9,06375	-0,188414	-0,275197	0,6339	0,816873	no
XLOC_002914	g7051	199,2	151,941	-0,390704	-0,485545	0,42915	0,677533	no
XLOC_002915	g7052	113,315	191,996	0,760735	1,20313	0,03395	0,153141	no
XLOC_002916	g7053	11,4819	7,52488	-0,609622	-0,634494	0,2765	0,538183	no
XLOC_002917	g7054	10,8508	12,0465	0,150807	0,178716	0,7576	0,8854	no
XLOC_002918	g7055	35,6066	30,6331	-0,217057	-0,362625	0,5185	0,743201	no
XLOC_002919	g7056	293,553	110,631	-1,40787	-2,32909	5,00E-05	0,00120049	yes
XLOC_002920	g7057	7,76391	8,60125	0,147762	0,213842	0,70905	0,861955	no
XLOC_002921	g7058	185,44	120,94	-0,616658	-0,837202	0,1442	0,380146	no
XLOC_002922	g7059	0	0	0	0	1	1	no
XLOC_002923	g7061	145,555	203,738	0,485146	0,77545	0,17575	0,421972	no
XLOC_002924	g7062	36,3219	30,2866	-0,262161	-0,43939	0,431	0,678624	no
XLOC_002925	g7063	1,86638	5,31626	1,51017	1,19501	0,054	0,206576	no
XLOC_002926	g7060	223,696	135,678	-0,721357	-1,09652	0,04475	0,182342	no
XLOC_002927	g7064	19,9402	9,42779	-1,08069	-1,44984	0,0119	0,0753436	no
XLOC_002928	g7066	163,476	283,41	0,793809	1,26039	0,0277	0,134995	no
XLOC_002929	g7067	22,9118	11,1514	-1,03887	-1,5909	0,0059	0,0459825	yes
XLOC_002930	g7065	88,2893	91,5081	0,0516611	0,0849741	0,8796	0,945715	no
XLOC_002931	g1879	2,69476	6,13362	1,18658	1,48634	0,01255	0,0783976	no
XLOC_002932	g1880	11,8799	15,3993	0,374353	0,486021	0,38985	0,644825	no
XLOC_002933	g1881	0,888771	5,89581	2,7298	2,71569	0,00035	0,00552572	yes
XLOC_002934	g1882	13,7355	29,305	1,09324	0,901933	0,13015	0,357303	no
XLOC_002935	g7068	0	0	0	0	1	1	no
XLOC_002936	g7069	2,44928	1,93123	-0,342836	-0,342417	0,55925	0,770892	no
XLOC_002937	g7070	0,333535	0,40863	0,292957	0	1	1	no
XLOC_002938	g7071	0	0	0	0	1	1	no
XLOC_002939	g7072	0	0	0	0	1	1	no
XLOC_002940	g1883	22,9114	30,4995	0,412721	0,659838	0,24745	0,506238	no
XLOC_002941	g7073	0	0	0	0	1	1	no
XLOC_002942	g7074	0	0,0749608	inf	0	1	1	no
XLOC_002943	g7075	0	0	0	0	1	1	no

XLOC_002944	g7076	0	0	0	0	1	1	no
XLOC_002945	g7077	22,8414	9,88614	-1,20817	-1,67146	0,00485	0,0400901	yes
XLOC_002946	g7079	113,795	86,6097	-0,393839	-0,670848	0,2497	0,508092	no
XLOC_002947	g7078	50,8269	42,7978	-0,248057	-0,411295	0,4679	0,707324	no
XLOC_002948	g1884	0	0	0	0	1	1	no
XLOC_002949	g7080	35,0775	34,0805	-0,0415965	-0,0625068	0,909	0,961172	no
XLOC_002950	g7081	28,2889	23,0879	-0,293104	-0,497446	0,37585	0,634261	no
XLOC_002951	g7082	4,49975	1,1023	-2,02933	-1,70901	0,01235	0,0774438	no
XLOC_002952	g7083	483,858	896,514	0,889743	0,920004	0,1233	0,347009	no
XLOC_002953	g7084	42,1321	23,3168	-0,85355	-1,2663	0,0275	0,13442	no
XLOC_002954	g7085	0	0	0	0	1	1	no
XLOC_002955	g7086	127,39	319,852	1,32816	2,11959	0,0002	0,00367103	yes
XLOC_002956	g7087	49,7544	87,2787	0,810806	0,855886	0,14495	0,380896	no
XLOC_002957	g7089	29,4239	32,9777	0,164505	0,275427	0,62895	0,814277	no
XLOC_002958	g7088	35,0012	28,4678	-0,298074	-0,491137	0,3841	0,640475	no
XLOC_002959	g7090	888,96	477,707	-0,895993	-1,22132	0,0375	0,163304	no
XLOC_002960	g7091	14,692	8,82589	-0,73522	-1,08466	0,0648	0,230724	no
XLOC_002961	g1886	96,4704	160,481	0,734247	1,06774	0,0607	0,221589	no
XLOC_002962	g1888	37,4386	35,1488	-0,0910536	-0,155654	0,78245	0,899515	no
XLOC_002963	g1889	79,0338	72,9223	-0,116109	-0,196588	0,72055	0,867664	no
XLOC_002964	g1890	360,126	304,451	-0,24229	-0,373466	0,5004	0,729936	no
XLOC_002965	g1891	4,95595	10,2933	1,05447	1,31867	0,025	0,126481	no
XLOC_002966	g1893	45,4034	45,1965	-0,00658969	-0,0106173	0,9844	0,992379	no
XLOC_002967	g1895	696,046	989,921	0,508131	0,723529	0,19885	0,451802	no
XLOC_002968	g1897	188,318	375,294	0,994847	1,25714	0,02815	0,136174	no
XLOC_002969	g1899	1792,49	3074,17	0,77823	1,044	0,0753	0,253495	no
XLOC_002970	g1901	38,6106	58,2485	0,593226	0,916705	0,11505	0,332781	no
XLOC_002971	g1904	131,852	100,079	-0,397785	-0,67295	0,2249	0,482947	no
XLOC_002972	g1907	32,0365	30,068	-0,0914856	-0,140971	0,80595	0,910751	no
XLOC_002973	g1885	24,0589	19,3747	-0,312393	-0,520674	0,374	0,632348	no
XLOC_002974	g1887	99,5277	104,127	0,065171	0,1075	0,8499	0,932412	no
XLOC_002975	g1892	82,036	67,8598	-0,2737	-0,439784	0,43415	0,681156	no
XLOC_002976	g1894	44,5334	39,1345	-0,186446	-0,2985	0,595	0,792473	no
XLOC_002977	g1896	40,6026	46,4846	0,195179	0,33068	0,5735	0,779376	no
XLOC_002978	g1898	107,296	99,2377	-0,112634	-0,181018	0,7443	0,879499	no
XLOC_002979	g1900	28,374	25,4543	-0,156658	-0,261172	0,65055	0,828157	no
XLOC_002980	g1902	38,5638	30,5827	-0,334531	-0,547695	0,3374	0,597377	no
XLOC_002981	g1903	86,3781	67,4827	-0,356149	-0,536141	0,3493	0,610104	no
XLOC_002982	g1905	588,439	1091,08	0,890785	1,4687	0,00835	0,0587371	no
XLOC_002983	g1906	142,753	223,529	0,646944	0,979729	0,0767	0,256537	no
XLOC_002984	g7092	52,1032	22,3281	-1,22251	-1,9319	0,00075	0,0100752	yes
XLOC_002985	g7093	51,2797	23,6327	-1,1176	-1,7114	0,0031	0,0290477	yes
XLOC_002986	g7094	68,6534	58,4943	-0,231033	-0,396215	0,48695	0,720592	no
XLOC_002987	g7095	0	0	0	0	1	1	no
XLOC_002988	g1912	57,9494	118,937	1,03733	1,76851	0,0018	0,0192549	yes
XLOC_002989	g1908	80,8696	112,217	0,472624	0,820443	0,15545	0,392876	no
XLOC_002990	g1909	39,6244	38,6736	-0,0350394	-0,0587626	0,916	0,963749	no
XLOC_002991	g1910	20,5324	165,308	3,00918	3,14906	5,00E-05	0,00120049	yes
XLOC_002992	g1911	4,27733	1,94087	-1,14	-1,23385	0,051	0,199133	no
XLOC_002993	g7098	6,26986	6,42675	0,0356548	0,0430118	0,9376	0,972153	no
XLOC_002994	g7096	1,0939	4,08404	1,90052	1,76568	0,0048	0,0398445	yes
XLOC_002995	g7097	78,4268	112,096	0,515314	0,816828	0,1491	0,386628	no
XLOC_002996	g7099	27,2622	22,5642	-0,272867	-0,292883	0,60475	0,79937	no
XLOC_002997	g7100	6,26919	11,442	0,867992	1,0815	0,0624	0,225365	no
XLOC_002998	g7101	17,6719	7,39303	-1,25722	-1,2683	0,03575	0,158066	no
XLOC_002999	g7104	1732,83	2130,41	0,298	0,397515	0,4714	0,709122	no
XLOC_003000	g7102	0,352338	0,31171	-0,176753	0	1	1	no
XLOC_003001	g7103	43,8583	123,559	1,49428	2,10426	0,00055	0,00788467	yes
XLOC_003002	g1913	0	0	0	0	1	1	no
XLOC_003003	g7106	82,2526	26,0474	-1,65892	-2,42024	5,00E-05	0,00120049	yes
XLOC_003004	g7105	11,2524	11,306	0,00685526	0,00608966	0,96355	0,983889	no
XLOC_003005	g7107	0,671032	0,366364	-0,873103	0	1	1	no
XLOC_003006	g1915	31,5644	22,6585	-0,478247	-0,798937	0,1552	0,392851	no
XLOC_003007	g1914	23,0549	18,081	-0,350601	-0,556579	0,30545	0,566867	no
XLOC_003008	g1916	42,2624	52,9704	0,32581	0,563778	0,3235	0,584072	no
XLOC_003009	g7108	0	0	0	0	1	1	no
XLOC_003010	g7109	3,66462	1,78128	-1,04075	-1,05844	0,0861	0,276941	no
XLOC_003011	g1917	14,3869	9,2803	-0,63251	-0,771642	0,3308	0,591578	no
XLOC_003012	g1920	75,3402	213,373	1,50188	2,39229	5,00E-05	0,00120049	yes
XLOC_003013	g1918	214,896	477,218	1,15101	1,52866	0,00875	0,0605959	no
XLOC_003014	g1919	15,0308	18,9645	0,335384	0,479106	0,3945	0,648995	no
XLOC_003015	g1921	32,605	42,2909	0,375253	0,638182	0,2556	0,515081	no
XLOC_003016	g7110	20,4471	17,4547	-0,228278	-0,340174	0,5416	0,759787	no
XLOC_003017	g7111	69,982	44,7818	-0,644071	-0,959517	0,0957	0,295526	no
XLOC_003018	g7112	769,002	3558,59	2,21024	2,60921	0,0001	0,00209829	yes
XLOC_003019	g7113	5,15552	8,71357	0,757146	0,963291	0,1066	0,31712	no
XLOC_003020	g7114	14,7749	13,5586	-0,12395	-0,171754	0,7655	0,890348	no
XLOC_003021	g7115	11,9847	6,15818	-0,960614	-1,36472	0,0166	0,09494	no
XLOC_003022	g1922	94,7811	123,929	0,38684	0,619359	0,28275	0,545288	no
XLOC_003023	g1923	24,9671	14,9028	-0,744449	-0,712201	0,40505	0,658433	no
XLOC_003024	g7117	60,5409	68,8273	0,185071	0,320409	0,5795	0,782656	no
XLOC_003025	g7119	27,8717	45,4875	0,706669	1,14405	0,0455	0,18448	no
XLOC_003026	g7116	0	0	0	0	1	1	no
XLOC_003027	g7118	66,4186	63,5078	-0,0646533	-0,108671	0,8462	0,929972	no
XLOC_003028	g1926	27,3648	83,7784	1,61426	2,55207	5,00E-05	0,00120049	yes
XLOC_003029	g1928	51,6561	121,928	1,23901	1,98968	0,00055	0,00788467	yes
XLOC_003030	g1930	26,8656	29,5833	0,139022	0,232856	0,68445	0,84822	no
XLOC_003031	g1932	3,21778	4,51925	0,490015	0,630623	0,26475	0,525434	no
XLOC_003032	g1924	120,036	107,678	-0,15675	-0,243968	0,67535	0,842579	no
XLOC_003033	g1925	43,8452	50,1267	0,193161	0,307982	0,58335	0,784861	no
XLOC_003034	g1927	70,4507	103,842	0,559702	0,918275	0,1069	0,31782	no
XLOC_003035	g1929	36,5641	39,16	0,0989543	0,168392	0,76235	0,887899	no

XLOC_003036	g1931	2,14705	25,757	3,58454	3,73808	5,00E-05	0,00120049	yes
XLOC_003037	g7120	11,6428	11,4242	-0,0273456	-0,0377681	0,94295	0,97502	no
XLOC_003038	g1935	10,9567	6,48915	-0,755711	-0,963628	0,11145	0,326017	no
XLOC_003039	g1938	63,251	47,1506	-0,423812	-0,696079	0,21855	0,475656	no
XLOC_003040	g1933	9,74282	2,74588	-1,82707	-2,38695	5,00E-05	0,00120049	yes
XLOC_003041	g1934	118,159	73,5867	-0,683207	-1,00611	0,06815	0,238841	no
XLOC_003042	g1936	7,83332	4,29221	-0,867904	-1,2319	0,0314	0,145447	no
XLOC_003043	g1937	2,96853	0,573832	-2,37105	-1,48552	0,2059	0,460054	no
XLOC_003044	g7121	79,3077	79,1033	-0,00372176	-0,0061676	0,99175	0,995293	no
XLOC_003045	g1943	64,859	83,0201	0,356153	0,55634	0,33565	0,595606	no
XLOC_003046	g1939	18,5297	14,1987	-0,384087	-0,520702	0,36245	0,62266	no
XLOC_003047	g1940	114,847	58,9665	-0,961751	-1,58385	0,0078	0,0560322	no
XLOC_003048	g1941	134,287	94,4161	-0,508216	-0,635997	0,26545	0,52639	no
XLOC_003049	g1942	109,009	77,1976	-0,497818	-0,797703	0,16375	0,405862	no
XLOC_003050	g7122	38,5894	16,8111	-1,19879	-1,76064	0,00265	0,0257909	yes
XLOC_003051	g7123	4,91265	3,30176	-0,573265	-0,589567	0,30885	0,569881	no
XLOC_003052	g1944	0	0	0	0	1	1	no
XLOC_003053	g1945	0	0,0817213	inf	0	1	1	no
XLOC_003054	g1946	1,24731	0,969262	-0,363865	-0,367189	0,51755	0,742416	no
XLOC_003055	g1947	109,825	67,7407	-0,697106	-1,05829	0,07025	0,243335	no
XLOC_003056	g7124	0	0	0	0	1	1	no
XLOC_003057	g7125	4,94806	3,37015	-0,554053	-0,566182	0,3344	0,594729	no
XLOC_003058	g7126	20,7876	49,6751	1,2568	1,69801	0,00385	0,0340297	yes
XLOC_003059	g7127	52,2202	81,5499	0,643074	1,03385	0,0799	0,264448	no
XLOC_003060	g7128	0	0	0	0	1	1	no
XLOC_003061	g1949	81,3509	157,139	0,949812	1,52505	0,0065	0,0492896	yes
XLOC_003062	g1950	73,2474	52,6048	-0,477584	-0,801032	0,1613	0,402965	no
XLOC_003063	g1952	10,51	16,2855	0,631829	0,920613	0,1003	0,303714	no
XLOC_003064	g1954	12,7505	11,9812	-0,089784	-0,138162	0,8073	0,911019	no
XLOC_003065	g1948	62,3491	85,9334	0,46285	0,723492	0,2107	0,465798	no
XLOC_003066	g1951	70,1557	79,7442	0,184818	0,311778	0,58325	0,784861	no
XLOC_003067	g1953	1,47035	1,87928	0,35402	0,444126	0,44795	0,694047	no
XLOC_003068	g1955	52,592	72,6011	0,465147	0,817592	0,16135	0,402965	no
XLOC_003069	g1956	43,3639	32,3184	-0,42414	-0,680383	0,23665	0,494447	no
XLOC_003070	g7129	0	0	0	0	1	1	no
XLOC_003071	g7130	23,7136	26,1603	0,141665	0,185943	0,73955	0,876968	no
XLOC_003072	g7131	20,6553	23,247	0,170535	0,270962	0,63955	0,821072	no
XLOC_003073	g7132	11,2203	13,2696	0,242015	0,338374	0,5399	0,758378	no
XLOC_003074	g1957	0,474851	6,43287	3,75991	2,89326	0,0042	0,0360524	yes
XLOC_003075	g1958	6,28592	6,20827	-0,0179324	-0,0248191	0,96735	0,985566	no
XLOC_003076	g1959	11,2839	10,2698	-0,135864	-0,190001	0,75785	0,8854	no
XLOC_003077	g7133	0	0	0	0	1	1	no
XLOC_003078	g7134	0	0	0	0	1	1	no
XLOC_003079	g7135	0	0	0	0	1	1	no
XLOC_003080	g1961	3,50924	2,74721	-0,353192	-0,435468	0,4425	0,688832	no
XLOC_003081	g1962	6,66074	2,36765	-1,49223	-1,67761	0,0045	0,038062	yes
XLOC_003082	g1965	352,952	851,186	1,27	1,95718	0,0015	0,0168343	yes
XLOC_003083	g1960	143,694	286,898	0,997539	1,57282	0,0056	0,0440994	yes
XLOC_003084	g1963	8,30777	7,52898	-0,142008	-0,195263	0,73565	0,874871	no
XLOC_003085	g1964	99,782	58,6055	-0,767742	-1,23308	0,0302	0,142373	no
XLOC_003086	g1966	55,1204	57,1791	0,0529002	0,0789609	0,88895	0,950511	no
XLOC_003087	g7136	53,685	33,3615	-0,686334	-1,16004	0,04565	0,184859	no
XLOC_003088	g7137	4,97659	3,77589	-0,398343	-0,236706	0,6916	0,852776	no
XLOC_003089	g7138	4,6753	3,19196	-0,550616	-0,644654	0,264	0,524581	no
XLOC_003090	g7139	0	0	0	0	1	1	no
XLOC_003091	g7140	0	0,0241604	inf	0	1	1	no
XLOC_003092	g7141	0	0	0	0	1	1	no
XLOC_003093	g192	27,2007	28,8514	0,0849988	0,12417	0,83065	0,922276	no
XLOC_003094	g194	27,1447	25,1395	-0,110712	-0,182751	0,75	0,882036	no
XLOC_003095	g196	20,1894	19,317	-0,0637288	-0,103824	0,8576	0,935217	no
XLOC_003096	g198	25,0429	38,4163	0,617317	0,984291	0,08385	0,273013	no
XLOC_003097	g200	610,59	708,543	0,214653	0,358045	0,5203	0,744115	no
XLOC_003098	g201	14,8759	19,6129	0,398828	0,553315	0,3354	0,595593	no
XLOC_003099	g203	32,761	36,161	0,142454	0,21724	0,69535	0,855038	no
XLOC_003100	g205	207,746	161,827	-0,360362	-0,591053	0,2919	0,554536	no
XLOC_003101	g207	53,6035	88,1803	0,718129	1,14016	0,04415	0,180927	no
XLOC_003102	g191	31,8549	32,2018	0,0156277	0,026316	0,96025	0,982256	no
XLOC_003103	g193	39,8116	34,487	-0,207136	-0,317872	0,56585	0,774878	no
XLOC_003104	g195	74,6772	57,6797	-0,372606	-0,629079	0,2764	0,538114	no
XLOC_003105	g197	226,368	123,841	-0,87018	-1,42759	0,01265	0,078972	no
XLOC_003106	g199	8,83608	8,59227	-0,0403672	-0,0591734	0,91695	0,963869	no
XLOC_003107	g202	86,3225	145,049	0,748737	1,28517	0,0267	0,132288	no
XLOC_003108	g204	1186,03	760,675	-0,640787	-1,05276	0,06835	0,23903	no
XLOC_003109	g206	44,84	35,5728	-0,334013	-0,575321	0,3179	0,578273	no
XLOC_003110	g208	269,053	140,645	-0,935832	-1,50803	0,0076	0,0552012	no
XLOC_003111	g7142	0,105462	0,182906	0,794379	0	1	1	no
XLOC_003112	g7143	2499,47	154,433	-4,01657	-4,48752	5,00E-05	0,00120049	yes
XLOC_003113	g7144	123,663	0	#NAME?	0	1	1	no
XLOC_003114	g7145	0	0	0	0	1	1	no
XLOC_003115	g7146	7,08701	5,24803	-0,433402	-0,528127	0,363	0,622866	no
XLOC_003116	g7147	0	0	0	0	1	1	no
XLOC_003117	g1968	80,6565	51,188	-0,655984	-1,01221	0,0725	0,248673	no
XLOC_003118	g1967	281,244	1085,22	1,94809	3,17148	5,00E-05	0,00120049	yes
XLOC_003119	g1969	132,416	68,3882	-0,953255	-1,52286	0,0102	0,0675415	no
XLOC_003120	g1970	43,6055	26,4061	-0,72364	-1,06401	0,07015	0,24316	no
XLOC_003121	g1971	6,41218	8,64112	0,430403	0,553711	0,324	0,584313	no
XLOC_003122	g1972	19,8355	15,5788	-0,348499	-0,473139	0,40825	0,660681	no
XLOC_003123	g7148	0	0	0	0	1	1	no
XLOC_003124	g210	136,18	58,0422	-1,23034	-1,76766	0,00285	0,0272512	yes
XLOC_003125	g212	146,089	144,961	-0,0111805	-0,017699	0,97565	0,988025	no
XLOC_003126	g214	17,9925	30,4069	0,757004	1,22077	0,03585	0,158223	no
XLOC_003127	g216	69,5623	241,595	1,79621	2,91929	5,00E-05	0,00120049	yes

XLOC_003128	g218	33,5753	46,6353	0,474022	0,782485	0,1686	0,411547	no
XLOC_003129	g220	11,5165	26,1509	1,18315	1,82581	0,00225	0,0229201	yes
XLOC_003130	g222	276,061	299,971	0,119836	0,204361	0,7159	0,865461	no
XLOC_003131	g223	2272,1	2762,94	0,282179	0,303397	0,60595	0,800219	no
XLOC_003132	g225	146,579	222,764	0,603838	0,88631	0,12535	0,348905	no
XLOC_003133	g226	62,366	67,814	0,120824	0,177446	0,7604	0,886938	no
XLOC_003134	g228	14,9196	14,0736	-0,0842227	-0,13124	0,8201	0,918078	no
XLOC_003135	g230	434,464	769,988	0,825601	1,10966	0,05705	0,214074	no
XLOC_003136	g232	219,507	79,2967	-1,46894	-2,39164	5,00E-05	0,00120049	yes
XLOC_003137	g234	14,9229	13,5032	-0,144227	-0,215354	0,7081	0,861576	no
XLOC_003138	g236	2,54452	6,94796	1,4492	1,78139	0,0034	0,0310297	yes
XLOC_003139	g209	1,21593	4,31939	1,82877	1,57864	0,0113	0,0724321	no
XLOC_003140	g211	5,41075	4,15487	-0,381025	-0,419184	0,4768	0,712986	no
XLOC_003141	g213	79,4198	306,47	1,94818	3,03138	5,00E-05	0,00120049	yes
XLOC_003142	g215	62,7206	105,502	0,750258	1,17931	0,0442	0,180927	no
XLOC_003143	g217	17,5044	18,101	0,0483552	0,0722603	0,8994	0,955495	no
XLOC_003144	g219	3,20017	6,61392	1,04736	1,28389	0,02375	0,122944	no
XLOC_003145	g221	38,7941	42,6987	0,138358	0,238768	0,6773	0,844002	no
XLOC_003146	g224	16,4826	40,8304	1,3087	1,86672	0,00155	0,0171215	yes
XLOC_003147	g227	1,81467	1,54598	-0,231185	-0,183119	0,769	0,89268	no
XLOC_003148	g229	63,3703	66,7021	0,0739264	0,115038	0,83975	0,926574	no
XLOC_003149	g231	122,222	165,168	0,434429	0,706933	0,21265	0,468106	no
XLOC_003150	g233	70,1448	93,1468	0,409171	0,705212	0,21055	0,465571	no
XLOC_003151	g235	30,1518	23,6187	-0,352316	-0,547142	0,33985	0,600023	no
XLOC_003152	g237	95,7929	87,0228	-0,138525	-0,237212	0,67	0,839486	no
XLOC_003153	g1973	102,182	150,815	0,561643	0,891697	0,116	0,333955	no
XLOC_003154	g7149	54,3726	38,9891	-0,479809	-0,809356	0,1503	0,387489	no
XLOC_003155	g7150	2213,5	1952,75	-0,180819	-0,220912	0,7046	0,859524	no
XLOC_003156	g1974	0,0545473	0,2622	2,26509	0	1	1	no
XLOC_003157	g1976	18,8228	12,5259	-0,587569	-1,00348	0,07685	0,256864	no
XLOC_003158	g1979	76,2763	41,2755	-0,885949	-1,40854	0,01105	0,0713888	no
XLOC_003159	g1980	8,17786	5,68789	-0,52383	-0,746993	0,1893	0,439151	no
XLOC_003160	g1982	53,0266	159,223	1,58626	2,37743	0,0001	0,00209829	yes
XLOC_003161	g1984	98,9698	128,408	0,375675	0,620455	0,2593	0,519235	no
XLOC_003162	g1985	1,97653	2,9678	0,586422	0,43254	0,43235	0,679357	no
XLOC_003163	g1987	108,65	103,307	-0,0727415	-0,117025	0,8373	0,92562	no
XLOC_003164	g1988	45,3782	37,0485	-0,292582	-0,424614	0,45345	0,698162	no
XLOC_003165	g1990	96,473	141,536	0,552977	0,880884	0,12405	0,347197	no
XLOC_003166	g1992	88,9115	184,929	1,05653	1,57098	0,0062	0,0477522	yes
XLOC_003167	g1994	9,60739	11,5158	0,261399	0,395064	0,46885	0,707556	no
XLOC_003168	g1975	3,50242	5,32962	0,605679	0,588049	0,29735	0,559383	no
XLOC_003169	g1977	32,1658	48,6806	0,597821	0,830195	0,1533	0,391319	no
XLOC_003170	g1978	35,0809	60,7445	0,792069	1,33437	0,0181	0,10099	no
XLOC_003171	g1981	12,7545	3,85305	-1,72693	-1,50136	0,2216	0,478582	no
XLOC_003172	g1983	130,539	154,886	0,246719	0,427964	0,45905	0,701036	no
XLOC_003173	g1986	128,806	148,321	0,203522	0,333545	0,55025	0,765215	no
XLOC_003174	g1989	67,472	55,7841	-0,274433	-0,470079	0,40285	0,656594	no
XLOC_003175	g1991	8,37471	21,3459	1,34985	1,94463	0,0007	0,00954722	yes
XLOC_003176	g1993	28,6574	300,924	3,39242	4,19811	5,00E-05	0,00120049	yes
XLOC_003177	g7151	7,59249	11,5441	0,604508	0,759642	0,1814	0,429034	no
XLOC_003178	g7153	502,592	49,474	-3,34464	-4,37383	5,00E-05	0,00120049	yes
XLOC_003179	g7152	54,1735	30,7708	-0,816024	-1,21121	0,0313	0,145189	no
XLOC_003180	g238	0,225828	3,93265	4,12221	1,48094	0,1169	0,33517	no
XLOC_003181	g7154	91,355	109,529	0,261756	0,444621	0,43765	0,68432	no
XLOC_003182	g7157	27,5541	37,2282	0,434129	0,583749	0,30035	0,562226	no
XLOC_003183	g7155	73,4307	71,7126	-0,034157	-0,0558145	0,91805	0,964054	no
XLOC_003184	g7156	127,419	71,4516	-0,83454	-1,37651	0,01575	0,0915719	no
XLOC_003185	g7158	22,1702	16,4587	-0,429773	-0,63177	0,2758	0,537479	no
XLOC_003186	g7159	0,158817	0	#NAME?	0	1	1	no
XLOC_003187	g1995	0	0	0	0	1	1	no
XLOC_003188	g7160	0	0	0	0	1	1	no
XLOC_003189	g7161	0	0,0202583	inf	0	1	1	no
XLOC_003190	g7162	1,39097	0,865239	-0,684924	0	1	1	no
XLOC_003191	g7163	8,21396	61,4957	2,90433	4,33453	5,00E-05	0,00120049	yes
XLOC_003192	g1996	0	0	0	0	1	1	no
XLOC_003193	g1998	0,789326	16,4998	4,38569	4,47565	5,00E-05	0,00120049	yes
XLOC_003194	g1997	0,0219036	1,31765	5,91066	1,1912	0,12375	0,347009	no
XLOC_003195	g7165	138,302	105,418	-0,391701	-0,638163	0,2749	0,53647	no
XLOC_003196	g7164	132,681	205,469	0,630959	1,04958	0,07025	0,243335	no
XLOC_003197	g7166	1,50164	0,680311	-1,14227	-1,01471	0,089	0,282567	no
XLOC_003198	g1999	16,6655	28,0823	0,752794	1,01363	0,0734	0,250274	no
XLOC_003199	g7167	7432,17	14479,5	0,962154	0,946232	0,0991	0,301943	no
XLOC_003200	g7168	32,0779	23,7964	-0,430839	-0,716189	0,2016	0,45375	no
XLOC_003201	g7169	9,26362	7,51456	-0,301887	-0,466488	0,4066	0,659969	no
XLOC_003202	g7170	342,362	281,929	-0,280186	-0,442886	0,42325	0,67289	no
XLOC_003203	g7171	408,653	361,009	-0,178842	-0,262186	0,6407	0,821582	no
XLOC_003204	g7174	38,7662	39,5708	0,0296368	0,049909	0,93275	0,970606	no
XLOC_003205	g7176	58,0655	123,578	1,08967	1,69325	0,00525	0,0423624	yes
XLOC_003206	g7172	8,48054	22,1653	1,38608	1,02489	0,0997	0,302738	no
XLOC_003207	g7173	13,3855	18,8703	0,495446	0,540864	0,3538	0,614923	no
XLOC_003208	g7175	60,8088	103,44	0,766438	1,17733	0,0348	0,155688	no
XLOC_003209	g7177	56,6247	62,4603	0,141509	0,228932	0,6919	0,853039	no
XLOC_003210	g7178	1183,15	868,116	-0,446674	-0,748015	0,2	0,452439	no
XLOC_003211	g7179	0	0,0347618	inf	0	1	1	no
XLOC_003212	g7180	83,4093	79,0166	-0,0780514	-0,122321	0,83075	0,922276	no
XLOC_003213	g7181	0	0,811283	inf	0	1	1	no
XLOC_003214	g7182	0	0	0	0	1	1	no
XLOC_003215	g7184	65,1608	118,353	0,861023	1,38388	0,01725	0,097634	no
XLOC_003216	g7187	244,336	139,713	-0,806399	-1,28586	0,0301	0,14197	no
XLOC_003217	g7192	43,2955	50,4507	0,220656	0,38218	0,50795	0,734836	no
XLOC_003218	g7183	105,932	134,894	0,348689	0,584833	0,30225	0,564048	no
XLOC_003219	g7185	69,7037	57,6778	-0,273217	-0,458867	0,41335	0,665862	no

XLOC_003220	g7186	65,2934	65,086	-0,00458845	-0,0076391	0,9902	0,99501	no
XLOC_003221	g7188	53,7557	61,5921	0,196329	0,33362	0,55225	0,766299	no
XLOC_003222	g7189	52,3563	40,7525	-0,361474	-0,596108	0,2816	0,543914	no
XLOC_003223	g7190	473,606	5951,39	3,65147	4,69593	5,00E-05	0,00120049	yes
XLOC_003224	g7191	247,284	344,822	0,479685	0,751222	0,2034	0,456337	no
XLOC_003225	g7193	7,18602	4,51122	-0,671673	-0,678931	0,23875	0,496827	no
XLOC_003226	g7194	79,4318	48,577	-0,709443	-1,09013	0,0588	0,217237	no
XLOC_003227	g7195	0,0198246	0	#NAME?	0	1	1	no
XLOC_003228	g7196	0	0	0	0	1	1	no
XLOC_003229	g7198	27,7589	22,0177	-0,334286	-0,485062	0,38525	0,640945	no
XLOC_003230	g7197	0,463938	1,07329	1,21003	0,908965	0,1549	0,392851	no
XLOC_003231	g7199	14,1328	23,4833	0,732595	1,15623	0,04605	0,185789	no
XLOC_003232	g7200	0	0	0	0	1	1	no
XLOC_003233	g7201	0	0	0	0	1	1	no
XLOC_003234	g7202	16,0417	8,30869	-0,949132	-1,22416	0,04055	0,171343	no
XLOC_003235	g7203	222,546	159,023	-0,484869	-0,749206	0,19565	0,447122	no
XLOC_003236	g7204	57,3904	41,9503	-0,452129	-0,620135	0,27195	0,53315	no
XLOC_003237	g7205	3,44418	9,50128	1,46396	2,08552	0,00055	0,00788467	yes
XLOC_003238	g7206	0	0	0	0	1	1	no
XLOC_003239	g7207	81,9908	163,817	0,998553	1,47364	0,00765	0,0553596	no
XLOC_003240	g7208	14,2982	23,1308	0,69398	1,07747	0,05985	0,219875	no
XLOC_003241	g7209	0	0	0	0	1	1	no
XLOC_003242	g7210	68,025	98,4122	0,532773	0,722119	0,2269	0,485437	no
XLOC_003243	g7211	0,0709684	0	#NAME?	0	1	1	no
XLOC_003244	g7212	1,29878	1,99447	0,618845	0,750211	0,19845	0,451315	no
XLOC_003245	g7213	142,385	102,493	-0,474264	-0,79944	0,1647	0,406882	no
XLOC_003246	g7214	44,6958	31,3885	-0,509902	-0,854098	0,13365	0,363462	no
XLOC_003247	g7216	204,803	203,716	-0,00767658	-0,0128391	0,9825	0,991485	no
XLOC_003248	g7217	11,3215	12,0025	0,0842727	0,125003	0,8282	0,921598	no
XLOC_003249	g7215	26,8696	24,0471	-0,16011	-0,267967	0,63625	0,818612	no
XLOC_003250	g7218	0	0	0	0	1	1	no
XLOC_003251	g7219	5,76719	10,0219	0,797222	0,850644	0,14695	0,383892	no
XLOC_003252	g7220	0	0	0	0	1	1	no
XLOC_003253	g7221	0	0	0	0	1	1	no
XLOC_003254	g7222	36,8904	27,7389	-0,411336	-0,455683	0,4315	0,678624	no
XLOC_003255	g7223	17,6231	25,1685	0,514154	0,787154	0,15145	0,388922	no
XLOC_003256	g7224	62,8906	469,935	2,90155	4,00385	5,00E-05	0,00120049	yes
XLOC_003257	g7225	102,737	107,337	0,0631865	0,10922	0,8539	0,934503	no
XLOC_003258	g7226	0,0655673	0,404104	2,62368	0	1	1	no
XLOC_003259	g239	1252,57	2816,55	1,16903	1,1715	0,03765	0,163739	no
XLOC_003260	g7227	18,1342	9,609	-0,916253	-1,39658	0,01875	0,103908	no
XLOC_003261	g7228	249,172	101,414	-1,29689	-1,76473	0,00155	0,0171215	yes
XLOC_003262	g7229	0	0,0860962	inf	0	1	1	no
XLOC_003263	g7230	0	0	0	0	1	1	no
XLOC_003264	g7231	6,21458	7,97807	0,360382	0,433836	0,45425	0,698846	no
XLOC_003265	g7232	29,0901	104,419	1,84379	2,50531	5,00E-05	0,00120049	yes
XLOC_003266	g7233	109,744	19,7409	-2,47488	-3,57527	5,00E-05	0,00120049	yes
XLOC_003267	g7234	56,8955	11,7159	-2,27985	-3,60411	5,00E-05	0,00120049	yes
XLOC_003268	g7235	126,459	90,5161	-0,482428	-0,762526	0,17725	0,424122	no
XLOC_003269	g7236	22,5087	32,4895	0,529493	0,908191	0,1147	0,332356	no
XLOC_003270	g7237	46,1621	47,4704	0,0403211	0,066393	0,90495	0,958435	no
XLOC_003271	g7238	22,4557	26,0339	0,213306	0,234364	0,6825	0,847104	no
XLOC_003272	g7239	20,6747	31,9156	0,626395	0,774639	0,1768	0,423768	no
XLOC_003273	g7240	59,6126	67,2622	0,174179	0,251079	0,6577	0,832081	no
XLOC_003274	g7242	207,503	119,628	-0,79458	-1,23331	0,03235	0,148309	no
XLOC_003275	g7241	272,8	290,464	0,0905134	0,15119	0,79565	0,906216	no
XLOC_003276	g7243	25,6716	20,1187	-0,351636	-0,514067	0,3748	0,633023	no
XLOC_003277	g2000	22,2446	52,7878	1,24675	1,87501	0,0007	0,00954722	yes
XLOC_003278	g2001	178,176	107,941	-0,723066	-1,08604	0,0587	0,217031	no
XLOC_003279	g7244	0	0,0453098	inf	0	1	1	no
XLOC_003280	g2002	32,8028	34,8785	0,0885229	0,145205	0,7947	0,905607	no
XLOC_003281	g2003	2,04034	1,92079	-0,0871133	-0,100028	0,86365	0,936821	no
XLOC_003282	g2006	46,0627	38,9904	-0,24048	-0,234906	0,68165	0,846629	no
XLOC_003283	g2004	23,7145	24,5895	0,0522762	0,0840174	0,8824	0,947323	no
XLOC_003284	g2005	260,687	502,268	0,946136	1,55355	0,0091	0,0622298	no
XLOC_003285	g7245	9,01935	2,71501	-1,73207	-2,32925	0,00035	0,00552572	yes
XLOC_003286	g2008	11,2944	10,3068	-0,13201	-0,185252	0,749	0,881846	no
XLOC_003287	g2007	9,02735	9,00111	-0,00419866	-0,00594198	0,9929	0,995841	no
XLOC_003288	g2009	23,0824	21,214	-0,121779	-0,133214	0,81845	0,917235	no
XLOC_003289	g7246	184,307	227,675	0,304865	0,50727	0,3712	0,629674	no
XLOC_003290	g2010	3,7641	1,62797	-1,20923	-1,3904	0,0182	0,101432	no
XLOC_003291	g7247	11,1327	7,34967	-0,599053	-0,770352	0,1748	0,420617	no
XLOC_003292	g7248	2,45515	33,8078	3,78348	3,10772	0,00015	0,00294012	yes
XLOC_003293	g7249	1,59828	5,76048	1,84967	2,11122	0,0012	0,0143358	yes
XLOC_003294	g7250	539,418	181,111	-1,57453	-2,02139	0,00125	0,0146655	yes
XLOC_003295	g7251	2449,05	1059,08	-1,20942	-1,5841	0,00805	0,0571591	no
XLOC_003296	g7252	861,984	635,498	-0,439774	-0,588192	0,29305	0,555336	no
XLOC_003297	g2011	0	0	0	0	1	1	no
XLOC_003298	g7253	36,3419	17,4072	-1,06195	-1,71089	0,00405	0,0351645	yes
XLOC_003299	g7255	24,4233	29,307	0,262987	0,386938	0,5035	0,731632	no
XLOC_003300	g7254	21,8654	25,1215	0,200278	0,256971	0,6497	0,827611	no
XLOC_003301	g7256	23,6183	35,3216	0,580647	0,905548	0,1207	0,342367	no
XLOC_003302	g7257	62,2529	89,6381	0,525972	0,763195	0,17035	0,414171	no
XLOC_003303	g7258	20,7926	9,02903	-1,20343	-1,85265	0,00105	0,0130684	yes
XLOC_003304	g7259	37,8689	80,9466	1,09596	0,817028	0,43755	0,68432	no
XLOC_003305	g7262	95,848	103,242	0,107207	0,177487	0,7507	0,882542	no
XLOC_003306	g7260	20,9619	28,187	0,42726	0,683145	0,2302	0,487612	no
XLOC_003307	g7261	1039,28	1445,27	0,475757	0,736586	0,19505	0,446686	no
XLOC_003308	g7263	9,57576	7,76688	-0,302051	-0,340693	0,55595	0,768934	no
XLOC_003309	g2013	6,54749	9,4005	0,521796	0,700419	0,21875	0,475881	no
XLOC_003310	g2014	11,4012	9,68444	-0,235449	-0,347243	0,53605	0,755347	no
XLOC_003311	g2016	21,7617	18,5042	-0,23394	-0,393075	0,48995	0,722384	no

XLOC_003312	g2018	59,1421	67,9936	0,201213	0,34864	0,5458	0,762304	no
XLOC_003313	g2012	40,6028	40,9112	0,0109164	0,0187259	0,9742	0,987624	no
XLOC_003314	g2015	3,95745	4,40033	0,153041	0,201795	0,72605	0,869807	no
XLOC_003315	g2017	42,3556	57,6401	0,444521	0,763548	0,1799	0,426853	no
XLOC_003316	g2019	1226,73	2199,25	0,842193	1,34353	0,01945	0,106823	no
XLOC_003317	g7265	179,73	188,782	0,07089	0,116816	0,8374	0,925627	no
XLOC_003318	g7264	40,6172	28,4496	-0,513686	-0,773388	0,17755	0,424426	no
XLOC_003319	g7266	20,7976	15,2616	-0,446514	-0,713463	0,1931	0,443877	no
XLOC_003320	g7267	27,6578	34,5355	0,320393	0,496625	0,38535	0,640945	no
XLOC_003321	g7269	62,3068	101,504	0,704075	1,11218	0,05465	0,20817	no
XLOC_003322	g7270	35,3457	39,1908	0,148978	0,215356	0,7023	0,858746	no
XLOC_003323	g7268	456,599	279,382	-0,708689	-1,07764	0,0495	0,195374	no
XLOC_003324	g7271	16,7557	5,46388	-1,61665	-2,2665	0,0001	0,00209829	yes
XLOC_003325	g7272	28,2248	53,6887	0,927655	1,05516	0,0677	0,238029	no
XLOC_003326	g7273	91,3372	112,559	0,301406	0,50565	0,3783	0,636208	no
XLOC_003327	g7274	0	0	0	0	1	1	no
XLOC_003328	g2020	12,4015	8,70071	-0,511307	-0,67172	0,2272	0,48555	no
XLOC_003329	g2021	63,103	56,5644	-0,157814	-0,275566	0,63345	0,816722	no
XLOC_003330	g2022	2272,44	3501,61	0,623779	0,759513	0,1793	0,426373	no
XLOC_003331	g7275	0,031875	0,0638199	1,00158	0	1	1	no
XLOC_003332	g7276	19,2119	13,7092	-0,486859	-0,755912	0,16685	0,410129	no
XLOC_003333	g7277	13,4116	9,87987	-0,440916	-0,641323	0,26115	0,521008	no
XLOC_003334	g7278	11,9252	8,1633	-0,54679	-0,698315	0,233	0,490789	no
XLOC_003335	g7279	250,207	138,628	-0,851898	-1,14578	0,0504	0,197813	no
XLOC_003336	g7280	250,288	112,044	-1,15952	-1,8707	0,00175	0,0188639	yes
XLOC_003337	g7281	0	0	0	0	1	1	no
XLOC_003338	g2023	0	0	0	0	1	1	no
XLOC_003339	g7282	0	0	0	0	1	1	no
XLOC_003340	g7283	0	0	0	0	1	1	no
XLOC_003341	g7284	1,18303	0,897929	-0,397818	-0,36984	0,534	0,753431	no
XLOC_003342	g7285	0	0	0	0	1	1	no
XLOC_003343	g2026	14,4613	21,6607	0,582888	0,816489	0,14945	0,387025	no
XLOC_003344	g2027	218,753	5286,25	4,59487	6,17815	5,00E-05	0,00120049	yes
XLOC_003345	g2029	27,0319	28,4071	0,0715876	0,123396	0,8283	0,921598	no
XLOC_003346	g2031	62,2594	30,773	-1,01663	-1,72612	0,0031	0,0290477	yes
XLOC_003347	g2024	55,8694	50,9832	-0,132036	-0,22738	0,68645	0,849413	no
XLOC_003348	g2025	35,517	34,5962	-0,0378974	-0,0605595	0,91455	0,963407	no
XLOC_003349	g2028	232,47	98,2997	-1,24179	-1,6443	0,00505	0,0412571	yes
XLOC_003350	g2030	151,213	176,662	0,224406	0,259357	0,6174	0,808291	no
XLOC_003351	g7286	0	0	0	0	1	1	no
XLOC_003352	g7287	14,5753	12,647	-0,204728	-0,253312	0,6594	0,832623	no
XLOC_003353	g7288	117,656	70,0518	-0,748079	-1,06607	0,06205	0,224514	no
XLOC_003354	g7289	38,2295	30,1124	-0,344328	-0,472641	0,39295	0,647853	no
XLOC_003355	g7291	61,2768	36,3201	-0,754574	-1,22101	0,03125	0,145026	no
XLOC_003356	g7293	54,5408	49,191	-0,14894	-0,246077	0,6679	0,837968	no
XLOC_003357	g7290	188,952	146,313	-0,368961	-0,596169	0,29405	0,556265	no
XLOC_003358	g7292	93,5024	128,564	0,459408	0,733058	0,196	0,447297	no
XLOC_003359	g7294	48,1624	36,6855	-0,392698	-0,680856	0,23335	0,49079	no
XLOC_003360	g7295	0	0	0	0	1	1	no
XLOC_003361	g2032	150,085	266,722	0,829563	1,34061	0,01975	0,108047	no
XLOC_003362	g7296	3,97159	7,5662	0,929851	0,533234	0,37345	0,631938	no
XLOC_003363	g7297	30,7046	30,2561	-0,0212297	-0,0307799	0,95605	0,980564	no
XLOC_003364	g7298	153,953	150,989	-0,02805	-0,0488067	0,93305	0,970764	no
XLOC_003365	g7299	0	0	0	0	1	1	no
XLOC_003366	g7300	0,88722	0,765654	-0,212599	0	1	1	no
XLOC_003367	g2033	25,8956	40,2656	0,636842	1,04718	0,0741	0,251525	no
XLOC_003368	g2035	21,5026	14,2038	-0,598236	-0,977771	0,08535	0,276157	no
XLOC_003369	g2037	195,572	197,62	0,0150342	0,0224627	0,9657	0,984651	no
XLOC_003370	g2039	49,6549	58,7012	0,241455	0,398572	0,48755	0,720794	no
XLOC_003371	g2041	103,074	91,755	-0,167818	-0,269637	0,6389	0,820731	no
XLOC_003372	g2043	70,1753	71,0605	0,0180852	0,0291341	0,95795	0,981385	no
XLOC_003373	g2044	164,816	314,784	0,933511	1,49995	0,0076	0,0552012	no
XLOC_003374	g2047	61,4736	78,4465	0,351742	0,566878	0,30465	0,566374	no
XLOC_003375	g2034	52,3251	58,9085	0,170972	0,264839	0,63775	0,819833	no
XLOC_003376	g2036	494,569	495,184	0,00179094	0,00220602	0,99745	0,998008	no
XLOC_003377	g2038	25,7582	32,8084	0,349033	0,535037	0,3303	0,591007	no
XLOC_003378	g2040	19,0294	26,5909	0,482704	0,80977	0,15495	0,392851	no
XLOC_003379	g2042	1476,73	2345,99	0,66779	0,93628	0,08865	0,281729	no
XLOC_003380	g2045	38,1324	44,4681	0,221753	0,37399	0,5237	0,746189	no
XLOC_003381	g2046	44,6662	39,9316	-0,161654	-0,270068	0,62445	0,811876	no
XLOC_003382	g2048	99,4287	84,8874	-0,228111	-0,350891	0,55165	0,766224	no
XLOC_003383	g2049	44,7013	45,706	0,0320672	0,0489779	0,9275	0,967861	no
XLOC_003384	g7301	0	0	0	0	1	1	no
XLOC_003385	g7302	0	0	0	0	1	1	no
XLOC_003386	g7303	24,5962	21,5357	-0,191705	-0,273028	0,63365	0,816808	no
XLOC_003387	g2050	23,3278	19,7805	-0,237973	-0,354443	0,52295	0,745661	no
XLOC_003388	g2051	33,4448	31,0685	-0,10633	-0,180593	0,7466	0,880922	no
XLOC_003389	g16	29,6232	24,05	-0,300689	-0,456029	0,43145	0,678624	no
XLOC_003390	g17	9,44665	13,6634	0,532437	0,778329	0,17735	0,424257	no
XLOC_003391	g18	148,305	94,5444	-0,649499	-1,10148	0,0568	0,213462	no
XLOC_003392	g22	25,8495	44,7019	0,790203	1,30033	0,02615	0,13022	no
XLOC_003393	g19	31,0357	22,2316	-0,481316	-0,786535	0,1619	0,40362	no
XLOC_003394	g20	3,11355	2,11348	-0,558939	-0,672576	0,25515	0,514703	no
XLOC_003395	g21	8,61798	15,2392	0,822364	1,17233	0,04055	0,171343	no
XLOC_003396	g23	147,256	178,065	0,274082	0,476615	0,40475	0,658381	no
XLOC_003397	g1	58,9094	86,6685	0,557011	0,814381	0,14685	0,383835	no
XLOC_003398	g2052	0	0,0172732	inf	0	1	1	no
XLOC_003399	g2053	2,79365	1,48698	-0,909764	-1,14488	0,05545	0,210077	no
XLOC_003400	g2055	119,624	109,055	-0,13346	-0,226589	0,68805	0,850292	no
XLOC_003401	g2056	3,08404	4,31235	0,483652	0,484481	0,38095	0,637706	no
XLOC_003402	g2058	33,0433	36,6478	0,149368	0,234664	0,68575	0,849081	no
XLOC_003403	g2060	8,32094	7,15266	-0,218265	-0,257586	0,6649	0,835806	no

XLOC_003404	g2054	39,9166	70,4759	0,820142	1,22651	0,03615	0,15919	no
XLOC_003405	g2057	3,32391	4,62762	0,477389	0,611002	0,2857	0,548274	no
XLOC_003406	g2059	35,161	23,1844	-0,600819	-0,973857	0,0917	0,28825	no
XLOC_003407	g2061	22,7513	18,1084	-0,329289	-0,48628	0,4096	0,662373	no
XLOC_003408	g7304	109,796	150,985	0,45957	0,768239	0,17795	0,424658	no
XLOC_003409	g7305	71,8508	68,8139	-0,0623043	-0,104977	0,85095	0,933043	no
XLOC_003410	g7306	66,3288	93,125	0,489533	0,797262	0,15965	0,400553	no
XLOC_003411	g7307	241,517	120,613	-1,00174	-1,29021	0,02705	0,133148	no
XLOC_003412	g7308	11,1045	7,32641	-0,599959	-0,680616	0,2338	0,491315	no
XLOC_003413	g7309	0,277845	0,484059	0,800902	0	1	1	no
XLOC_003414	g7310	0	0	0	0	1	1	no
XLOC_003415	g7311	0	0	0	0	1	1	no
XLOC_003416	g2062	0	0	0	0	1	1	no
XLOC_003417	g7312	0	0	0	0	1	1	no
XLOC_003418	g7314	72,9869	23,9623	-1,60687	-2,62308	5,00E-05	0,00120049	yes
XLOC_003419	g7317	185,786	303,962	0,710253	1,18367	0,03555	0,15775	no
XLOC_003420	g7313	34,1553	53,8643	0,657217	0,752632	0,19535	0,446852	no
XLOC_003421	g7315	34,0295	48,0858	0,498825	0,799701	0,16185	0,403598	no
XLOC_003422	g7316	99,8742	127,008	0,346739	0,59593	0,29825	0,560431	no
XLOC_003423	g7318	41,554	58,3017	0,48855	0,818769	0,16065	0,401932	no
XLOC_003424	g7319	0	0	0	0	1	1	no
XLOC_003425	g7320	3,27852	4,66023	0,507356	0,573813	0,3153	0,575939	no
XLOC_003426	g2063	29,1685	72,1635	1,30686	2,13729	0,0002	0,00367103	yes
XLOC_003427	g2064	47,4812	52,5448	0,146193	0,249093	0,6576	0,832081	no
XLOC_003428	g2065	53,1451	42,6389	-0,317768	-0,544722	0,33085	0,591578	no
XLOC_003429	g7321	0	0	0	0	1	1	no
XLOC_003430	g7322	21,8214	33,1869	0,604871	0,884911	0,12775	0,352984	no
XLOC_003431	g7323	5,44648	2,90334	-0,90761	-1,2128	0,03	0,141635	no
XLOC_003432	g7325	9,26914	4,5051	-1,04088	-1,4548	0,0141	0,0850504	no
XLOC_003433	g7324	15,4676	13,4693	-0,199572	-0,291371	0,61645	0,807569	no
XLOC_003434	g7326	42,4057	25,5067	-0,733381	-1,19813	0,04025	0,170663	no
XLOC_003435	g7328	47,5165	54,3026	0,192593	0,28966	0,6045	0,79937	no
XLOC_003436	g7327	137,157	168,374	0,295847	0,500875	0,3816	0,638058	no
XLOC_003437	g7330	88,1182	56,2822	-0,64676	-0,74256	0,18505	0,43349	no
XLOC_003438	g7329	31,1265	23,8979	-0,381261	-0,518858	0,3748	0,633023	no
XLOC_003439	g7332	15,0271	9,71726	-0,628941	-0,943808	0,0929	0,290512	no
XLOC_003440	g7331	114,809	108,832	-0,0771334	-0,126552	0,82195	0,918997	no
XLOC_003441	g7333	3,94896	3,34937	-0,237582	-0,255502	0,65545	0,831391	no
XLOC_003442	g7334	0,78768	1,38756	0,816864	0,639704	0,28455	0,546579	no
XLOC_003443	g7335	15,1541	12,4073	-0,288523	-0,420697	0,4671	0,707046	no
XLOC_003444	g2066	23,5173	14,1769	-0,730178	-1,16062	0,0409	0,172008	no
XLOC_003445	g2067	91,9531	150,225	0,708159	0,977681	0,06955	0,241934	no
XLOC_003446	g7336	0	0	0	0	1	1	no
XLOC_003447	g7337	21,6825	16,9638	-0,354073	-0,513418	0,367	0,626554	no
XLOC_003448	g2068	31,9361	36,8626	0,206972	0,327286	0,57345	0,779376	no
XLOC_003449	g7338	18,2382	42,624	1,2247	1,78291	0,0012	0,0143358	yes
XLOC_003450	g7339	36,1726	30,7648	-0,233616	-0,338796	0,55715	0,770051	no
XLOC_003451	g7340	0,0322907	0,0289908	-0,155525	0	1	1	no
XLOC_003452	g7341	0	0	0	0	1	1	no
XLOC_003453	g2069	1,17223	2,20059	0,908628	0	1	1	no
XLOC_003454	g7342	71,6421	93,885	0,390088	0,650162	0,2453	0,50394	no
XLOC_003455	g7343	0	0	0	0	1	1	no
XLOC_003456	g7344	0	0	0	0	1	1	no
XLOC_003457	g7346	29,9883	42,4673	0,501949	0,814829	0,14875	0,386027	no
XLOC_003458	g7345	56,9004	58,8436	0,0484479	0,0789354	0,88775	0,950055	no
XLOC_003459	g7347	35,0588	54,4551	0,635293	0,986645	0,0841	0,273555	no
XLOC_003460	g7348	9,18577	5,61034	-0,711312	-0,973691	0,087	0,278559	no
XLOC_003461	g7349	7,92677	4,42375	-0,841462	-1,102	0,0502	0,197423	no
XLOC_003462	g7350	0	0	0	0	1	1	no
XLOC_003463	g7351	0	0	0	0	1	1	no
XLOC_003464	g7352	0	0	0	0	1	1	no
XLOC_003465	g241	13,9726	15,4574	0,145691	0,23419	0,6826	0,847104	no
XLOC_003466	g242	144,923	174,352	0,266719	0,448917	0,4377	0,68432	no
XLOC_003467	g244	22,4081	33,608	0,58478	0,887527	0,12	0,340972	no
XLOC_003468	g245	266,313	231,097	-0,204621	-0,338117	0,5616	0,77218	no
XLOC_003469	g248	186,344	202,387	0,119143	0,200682	0,72335	0,86956	no
XLOC_003470	g252	21,3565	22,6401	0,0842016	0,139541	0,81645	0,916563	no
XLOC_003471	g253	268,926	193,975	-0,471335	-0,811361	0,16035	0,401591	no
XLOC_003472	g254	54,5075	101,981	0,903769	1,51446	0,00885	0,061159	no
XLOC_003473	g256	66,9787	59,2086	-0,177897	-0,249684	0,66375	0,835468	no
XLOC_003474	g257	1588,24	1847,16	0,217881	0,249234	0,6615	0,834416	no
XLOC_003475	g258	37,2775	41,969	0,171022	0,28429	0,6184	0,808291	no
XLOC_003476	g260	6,92874	18,6745	1,43041	2,17417	0,0006	0,00845337	yes
XLOC_003477	g240	46,2846	248,455	2,42438	3,59204	5,00E-05	0,00120049	yes
XLOC_003478	g243	53,4499	59,8802	0,163892	0,280561	0,6141	0,805977	no
XLOC_003479	g246	24,1255	19,0681	-0,339393	-0,550627	0,3261	0,586932	no
XLOC_003480	g247	62,2869	93,7086	0,589253	0,936599	0,10575	0,31526	no
XLOC_003481	g249	90,7786	65,2592	-0,47617	-0,802722	0,16655	0,409802	no
XLOC_003482	g250	75,486	69,0266	-0,129057	-0,210464	0,71655	0,865607	no
XLOC_003483	g251	40,9158	38,2839	-0,0959222	-0,161636	0,7775	0,895827	no
XLOC_003484	g255	931,361	1389,06	0,576696	0,916811	0,10995	0,323169	no
XLOC_003485	g259	32,0049	22,5812	-0,503173	-0,829401	0,14	0,373384	no
XLOC_003486	g261	27,4395	29,6037	0,109524	0,180885	0,74975	0,881953	no
XLOC_003487	g7353	0	0	0	0	1	1	no
XLOC_003488	g7355	3763,54	3195,44	-0,236077	-0,29274	0,6074	0,801057	no
XLOC_003489	g7356	19,0941	19,2159	0,0091721	0,0132182	0,98015	0,990233	no
XLOC_003490	g7354	0,580565	0,57218	-0,0209885	0	1	1	no
XLOC_003491	g2070	39,6638	53,5892	0,434121	0,692953	0,2164	0,472443	no
XLOC_003492	g7357	11,0323	11,6335	0,0765585	0,100751	0,86175	0,936105	no
XLOC_003493	g7358	6,40646	6,4269	0,00459613	0,00399603	0,9157	0,963585	no
XLOC_003494	g7359	102,629	88,8313	-0,208296	-0,389111	0,5526	0,766459	no
XLOC_003495	g7360	0,221064	0	#NAME?	0	1	1	no

XLOC_003496	g2071	17,2663	50,7218	1,55465	2,04814	0,00025	0,00430702	yes
XLOC_003497	g2073	23,0589	21,2139	-0,12031	-0,205716	0,71945	0,867399	no
XLOC_003498	g2072	25,0224	25,6804	0,0374524	0,0577561	0,917	0,963869	no
XLOC_003499	g2074	112,299	91,2674	-0,299179	-0,484648	0,37455	0,632951	no
XLOC_003500	g2075	462,086	161,64	-1,51538	-2,16509	0,0004	0,0061761	yes
XLOC_003501	g2076	40,3047	44,9051	0,155933	0,243526	0,66705	0,837544	no
XLOC_003502	g7361	66,4048	92,717	0,481547	0,80085	0,1655	0,408139	no
XLOC_003503	g7362	11,2727	36,7903	1,7065	2,53501	5,00E-05	0,00120049	yes
XLOC_003504	g2078	9,75872	19,32	0,985331	1,52882	0,0082	0,0580981	no
XLOC_003505	g2077	33,3054	26,4175	-0,334261	-0,545429	0,33165	0,592577	no
XLOC_003506	g2079	26,925	11,3959	-1,24043	-1,64748	0,00515	0,0418998	yes
XLOC_003507	g7363	0	0	0	0	1	1	no
XLOC_003508	g7364	0	0	0	0	1	1	no
XLOC_003509	g7367	28,8193	39,8206	0,466477	0,711707	0,1968	0,448186	no
XLOC_003510	g7365	49,2832	53,639	0,122187	0,190708	0,72875	0,871129	no
XLOC_003511	g7366	72,6894	86,6641	0,25369	0,434074	0,4391	0,685309	no
XLOC_003512	g7368	41,3036	34,1092	-0,276106	-0,424925	0,4366	0,683876	no
XLOC_003513	g7369	0,261975	0,708048	1,43442	0	1	1	no
XLOC_003514	g7370	0	0	0	0	1	1	no
XLOC_003515	g7371	15,7639	11,1686	-0,497185	-0,763712	0,1825	0,42989	no
XLOC_003516	g7375	162,786	173,475	0,0917445	0,132405	0,82055	0,918373	no
XLOC_003517	g7376	56,0418	48,627	-0,204745	-0,301367	0,5944	0,792155	no
XLOC_003518	g7372	18,1861	6,51093	-1,4819	-2,03655	0,0007	0,00954722	yes
XLOC_003519	g7373	37,4423	52,1482	0,477948	0,808147	0,14745	0,384102	no
XLOC_003520	g7374	23,8997	73,3058	1,61693	2,44015	5,00E-05	0,00120049	yes
XLOC_003521	g7377	0	0	0	0	1	1	no
XLOC_003522	g7378	139,128	71,7046	-0,956275	-1,44872	0,0127	0,0791835	no
XLOC_003523	g7381	30,5783	36,8812	0,27038	0,351552	0,53755	0,756267	no
XLOC_003524	g7379	59,4849	75,7848	0,349385	0,589525	0,30625	0,567308	no
XLOC_003525	g7380	39,6707	42,1653	0,0879849	0,144076	0,8023	0,908624	no
XLOC_003526	g7382	32,1714	18,8103	-0,774257	-1,20717	0,0287	0,137614	no
XLOC_003527	g7383	30,0312	43,9466	0,549289	0,746815	0,19205	0,442199	no
XLOC_003528	g7384	2,17177	7,25983	1,74107	1,55202	0,01335	0,0818844	no
XLOC_003529	g7387	267,552	232,91	-0,200042	-0,321993	0,56245	0,772769	no
XLOC_003530	g7385	1008,8	2488,29	1,30251	1,46405	0,01045	0,068504	no
XLOC_003531	g7386	437,316	266,449	-0,714821	-1,13972	0,0477	0,189795	no
XLOC_003532	g2081	9,93232	14,3974	0,535602	0,848369	0,13135	0,359392	no
XLOC_003533	g2080	15,9618	15,6068	-0,0324455	-0,043613	0,94305	0,975021	no
XLOC_003534	g7388	2,65348	3,10288	0,225725	0,291224	0,60565	0,800146	no
XLOC_003535	g7389	26,5678	39,076	0,556607	0,807206	0,15325	0,391294	no
XLOC_003536	g262	55,2231	30,6192	-0,850832	-1,41328	0,0163	0,0934965	no
XLOC_003537	g263	14,8114	3,65221	-2,01987	-2,55947	5,00E-05	0,00120049	yes
XLOC_003538	g265	80,6791	84,5683	0,0679229	0,110322	0,84575	0,929972	no
XLOC_003539	g264	19,7117	12,9712	-0,603736	-0,889307	0,10555	0,314759	no
XLOC_003540	g7390	58,537	80,7468	0,464056	0,665354	0,2342	0,491629	no
XLOC_003541	g7392	1,92863	1,48671	-0,375452	-0,403577	0,49055	0,722869	no
XLOC_003542	g7391	188,962	312,338	0,725012	0,857025	0,1328	0,362148	no
XLOC_003543	g2083	0	0	0	0	1	1	no
XLOC_003544	g2082	0	0	0	0	1	1	no
XLOC_003545	g7393	0	0	0	0	1	1	no
XLOC_003546	g7394	8,41549	5,36064	-0,650641	-0,870132	0,136	0,367406	no
XLOC_003547	g7395	3,26462	1,88537	-0,792073	-0,628315	0,27155	0,532898	no
XLOC_003548	g7396	0	0	0	0	1	1	no
XLOC_003549	g7397	0	0	0	0	1	1	no
XLOC_003550	g2084	0	0	0	0	1	1	no
XLOC_003551	g268	15,5515	4,9042	-1,66496	-2,50849	5,00E-05	0,00120049	yes
XLOC_003552	g269	99,1294	168,134	0,762227	1,25551	0,0298	0,14137	no
XLOC_003553	g270	23,8951	28,7467	0,266681	0,419952	0,4739	0,711248	no
XLOC_003554	g266	18,2978	15,939	-0,199113	-0,313445	0,5834	0,784861	no
XLOC_003555	g267	15,4645	17,4874	0,177361	0,288281	0,59905	0,795708	no
XLOC_003556	g7399	7,68297	11,371	0,565623	0,799791	0,16675	0,409986	no
XLOC_003557	g7398	27,3752	20,3709	-0,42636	-0,579657	0,33395	0,594729	no
XLOC_003558	g7400	117,577	111,45	-0,0772134	-0,119737	0,8295	0,922056	no
XLOC_003559	g7401	7,71263	3,29699	-1,22607	-1,07083	0,06815	0,238841	no
XLOC_003560	g7402	0	0,777141	inf	0	1	1	no
XLOC_003561	g7403	8,02689	3,56254	-1,17194	-1,49518	0,0079	0,0564204	no
XLOC_003562	g7404	77,1921	62,1176	-0,313452	-0,511231	0,3572	0,617771	no
XLOC_003563	g2086	13,7772	5,51479	-1,3209	-1,94344	0,00085	0,011085	yes
XLOC_003564	g2089	86,1665	113,417	0,396443	0,670642	0,242	0,500303	no
XLOC_003565	g2090	62,6958	81,22	0,373466	0,614331	0,28915	0,55259	no
XLOC_003566	g2091	40,0734	37,8767	-0,0813353	-0,137887	0,8073	0,911019	no
XLOC_003567	g2095	174,097	66,0834	-1,39753	-1,97106	0,0007	0,00954722	yes
XLOC_003568	g2098	12,1663	9,0922	-0,420193	-0,561467	0,3502	0,610916	no
XLOC_003569	g2085	64,446	82,9093	0,363443	0,598413	0,26775	0,528716	no
XLOC_003570	g2087	28,2618	23,0776	-0,292357	-0,473219	0,4125	0,66501	no
XLOC_003571	g2088	69,2762	100,208	0,532564	0,903312	0,1166	0,334897	no
XLOC_003572	g2092	138,089	50,5101	-1,45095	-2,34491	0,00015	0,00294012	yes
XLOC_003573	g2093	139,762	125,513	-0,155131	-0,252216	0,66855	0,83857	no
XLOC_003574	g2094	20,6602	14,1166	-0,549463	-0,699627	0,2328	0,490579	no
XLOC_003575	g2096	34,807	8,36576	-2,05681	-2,82425	5,00E-05	0,00120049	yes
XLOC_003576	g2097	54,5162	49,4403	-0,140998	-0,212438	0,69955	0,857274	no
XLOC_003577	g2099	52,7882	208,104	1,97902	2,67928	5,00E-05	0,00120049	yes
XLOC_003578	g7405	0	0	0	0	1	1	no
XLOC_003579	g2100	18,51	38,8836	1,07085	1,67431	0,00345	0,0313115	yes
XLOC_003580	g7406	33,1409	23,6515	-0,486683	-0,783094	0,1744	0,420169	no
XLOC_003581	g7408	16,0596	28,782	0,841731	1,2823	0,02465	0,125227	no
XLOC_003582	g7407	73,5825	61,68	-0,254559	-0,421784	0,4574	0,701013	no
XLOC_003583	g7409	0,114207	0	#NAME?	0	1	1	no
XLOC_003584	g2101	35,4754	20,6898	-0,777898	-1,21899	0,02885	0,137997	no
XLOC_003585	g271	99,0287	61,7592	-0,681191	-1,16401	0,04075	0,171966	no
XLOC_003586	g273	51,6011	28,8163	-0,840518	-1,28469	0,0242	0,123967	no
XLOC_003587	g275	28,5128	38,8812	0,447462	0,634292	0,2681	0,529088	no

XLOC_003588	g272	109,179	108,475	-0,00933755	-0,0155276	0,9794	0,990233	no
XLOC_003589	g274	87,1458	156,147	0,841405	1,25134	0,02335	0,12145	no
XLOC_003590	g7410	0	0	0	0	1	1	no
XLOC_003591	g7411	31,4081	125,874	2,00278	2,20751	0,0003	0,00496796	yes
XLOC_003592	g7412	5,67553	5,65754	-0,00458175	-0,00467919	0,95925	0,981846	no
XLOC_003593	g7413	76,4172	45,9475	-0,733911	-1,25981	0,03105	0,144576	no
XLOC_003594	g7414	57,088	150,685	1,40028	2,01796	0,00035	0,00552572	yes
XLOC_003595	g7416	31,1168	42,8413	0,461306	0,745809	0,1992	0,451802	no
XLOC_003596	g7417	48,5411	75,0004	0,627692	0,968997	0,09805	0,300328	no
XLOC_003597	g7415	2,3553	2,65637	0,173548	0,141307	0,8253	0,920751	no
XLOC_003598	g2103	55,8106	73,9308	0,405636	0,699072	0,218	0,474775	no
XLOC_003599	g2105	124,354	113,407	-0,132945	-0,231843	0,6882	0,850292	no
XLOC_003600	g2102	29,6561	47,3056	0,673682	1,01187	0,07185	0,247134	no
XLOC_003601	g2104	97,8677	143,457	0,551713	0,926365	0,09975	0,302796	no
XLOC_003602	g2107	2,59731	3,41831	0,396267	0,570417	0,3294	0,590473	no
XLOC_003603	g2110	0,623546	2,44114	1,96899	1,98785	0,0031	0,0290477	yes
XLOC_003604	g2106	29,4604	35,4074	0,265272	0,402935	0,4777	0,713645	no
XLOC_003605	g2108	13,3842	102,278	2,9339	4,17536	5,00E-05	0,00120049	yes
XLOC_003606	g2109	19,9412	407,243	4,35207	6,24309	5,00E-05	0,00120049	yes
XLOC_003607	g2111	58,2423	64,166	0,13974	0,223068	0,698	0,85623	no
XLOC_003608	g7419	54,971	42,0905	-0,385177	-0,66609	0,2448	0,503231	no
XLOC_003609	g7418	489,924	365,408	-0,423051	-0,69775	0,21505	0,470651	no
XLOC_003610	g7420	121,671	103,811	-0,229016	-0,32184	0,5716	0,77837	no
XLOC_003611	g7421	15,9086	7,39707	-1,10478	-1,53324	0,0095	0,0641609	no
XLOC_003612	g7423	21,2455	14,9722	-0,504875	-0,771977	0,1895	0,439304	no
XLOC_003613	g7422	41,9604	44,6751	0,0904438	0,143476	0,7999	0,907885	no
XLOC_003614	g7424	127,749	208,262	0,705086	1,05195	0,07365	0,250691	no
XLOC_003615	g7425	48,3	67,4222	0,4812	0,799012	0,15325	0,391294	no
XLOC_003616	g276	41,5861	61,1181	0,555498	0,724451	0,216	0,471989	no
XLOC_003617	g24	99,4104	125,506	0,336286	0,583278	0,3062	0,567308	no
XLOC_003618	g7426	26,9564	37,7309	0,485119	0,834156	0,147	0,38392	no
XLOC_003619	g2113	114,898	80,342	-0,516125	-0,882715	0,1165	0,334707	no
XLOC_003620	g2116	6,92368	11,378	0,716639	0,967014	0,07515	0,253425	no
XLOC_003621	g2118	15,7356	14,3127	-0,13673	-0,218182	0,71225	0,863888	no
XLOC_003622	g2119	12,3535	9,34673	-0,402388	-0,48382	0,40305	0,656594	no
XLOC_003623	g2121	56,3661	61,8036	0,132864	0,224987	0,6809	0,846464	no
XLOC_003624	g2122	19,3288	33,0084	0,772079	1,31102	0,02555	0,128272	no
XLOC_003625	g2124	152,663	158,659	0,0555816	0,0923787	0,86755	0,939446	no
XLOC_003626	g2129	53,2239	84,9304	0,674206	0,97786	0,0863	0,277473	no
XLOC_003627	g2130	36,4005	43,4355	0,254916	0,435813	0,44295	0,689127	no
XLOC_003628	g2131	40,0212	51,2178	0,355883	0,609208	0,27815	0,539702	no
XLOC_003629	g2134	1,85795	1,73556	-0,0983127	-0,111277	0,842	0,927787	no
XLOC_003630	g2135	28,7298	28,3033	-0,0215796	-0,0345276	0,95215	0,978762	no
XLOC_003631	g2137	62,4519	52,6923	-0,245154	-0,408654	0,4583	0,701013	no
XLOC_003632	g2139	57,1895	52,1858	-0,132095	-0,216505	0,6936	0,85417	no
XLOC_003633	g2141	161,965	220,137	0,44272	0,741723	0,18415	0,43231	no
XLOC_003634	g2143	12,3478	26,0987	1,07973	1,2516	0,0374	0,163013	no
XLOC_003635	g2112	47,1347	27,149	-0,795889	-1,3057	0,0218	0,115905	no
XLOC_003636	g2114	48,2706	80,7522	0,742358	1,26878	0,0238	0,122944	no
XLOC_003637	g2115	38,6636	53,8479	0,477913	0,748063	0,17365	0,419391	no
XLOC_003638	g2117	12,4968	9,66505	-0,370711	-0,573643	0,31325	0,573795	no
XLOC_003639	g2120	81,0813	163,824	1,0147	1,62266	0,004	0,0348845	yes
XLOC_003640	g2123	104,768	140,912	0,42759	0,727091	0,2093	0,464271	no
XLOC_003641	g2125	61,8451	50,4291	-0,294401	-0,498007	0,38725	0,643156	no
XLOC_003642	g2126	30,3939	33,4788	0,139467	0,224817	0,6822	0,847104	no
XLOC_003643	g2127	39,8952	31,8118	-0,326651	-0,548021	0,34	0,600141	no
XLOC_003644	g2128	58,046	88,5006	0,608492	1,03804	0,0625	0,22556	no
XLOC_003645	g2132	24,6107	49,5381	1,00925	1,48943	0,0129	0,0801252	no
XLOC_003646	g2133	86,1189	199,167	1,20957	1,94119	0,0009	0,0115984	yes
XLOC_003647	g2136	61,5819	61,7879	0,00481896	0,00826749	0,98765	0,993619	no
XLOC_003648	g2138	308,657	246,599	-0,32384	-0,553132	0,34295	0,603327	no
XLOC_003649	g2140	59,6663	83,2776	0,481013	0,827796	0,1462	0,382951	no
XLOC_003650	g2142	353,596	657,467	0,894817	1,4559	0,01035	0,068167	no
XLOC_003651	g7427	15,9995	19,1007	0,255591	0,366007	0,5359	0,755244	no
XLOC_003652	g7428	0	0	0	0	1	1	no
XLOC_003653	g2145	71,8957	266,249	1,8888	2,24385	0,0001	0,00209829	yes
XLOC_003654	g2144	55,1099	71,121	0,367964	0,616717	0,2824	0,544934	no
XLOC_003655	g2146	9,45819	6,78067	-0,480136	-0,733185	0,18495	0,433359	no
XLOC_003656	g2147	43,1065	75,8576	0,815387	1,3342	0,0223	0,117671	no
XLOC_003657	g7429	59,0289	50,4365	-0,226953	-0,356506	0,52855	0,749511	no
XLOC_003658	g7430	14,5839	13,0801	-0,157004	-0,203383	0,718	0,86608	no
XLOC_003659	g7431	46,7725	33,0264	-0,502043	-0,759668	0,19155	0,442073	no
XLOC_003660	g7433	24,4886	50,9267	1,05631	1,47871	0,0123	0,0774269	no
XLOC_003661	g7432	46,2949	49,8228	0,105953	0,144243	0,7873	0,901713	no
XLOC_003662	g7434	29,2345	19,4787	-0,585777	-0,941063	0,08955	0,28358	no
XLOC_003663	g7435	208,658	148,267	-0,492948	-0,701869	0,1914	0,441831	no
XLOC_003664	g7436	208,314	126,129	-0,723857	-0,990714	0,0783	0,260823	no
XLOC_003665	g7438	105,58	65,9484	-0,678922	-1,16334	0,0407	0,171903	no
XLOC_003666	g7437	28,3404	31,883	0,169926	0,26179	0,6338	0,816873	no
XLOC_003667	g7439	10,7191	11,8965	0,150358	0,158256	0,7815	0,898633	no
XLOC_003668	g7440	39,1847	26,7935	-0,548407	-0,80571	0,15395	0,39191	no
XLOC_003669	g7441	4,09078	2,93281	-0,480095	-0,348251	0,54995	0,765215	no
XLOC_003670	g7442	4,33987	2,02418	-1,10031	-0,884871	0,1324	0,361761	no
XLOC_003671	g277	36,2341	16,1812	-1,16303	-1,83708	0,0017	0,0185283	yes
XLOC_003672	g279	158,436	121,28	-0,385562	-0,639746	0,27075	0,532269	no
XLOC_003673	g280	246,534	76,3829	-1,69046	-2,50976	0,00015	0,00294012	yes
XLOC_003674	g281	324,104	208,278	-0,637947	-1,0312	0,06695	0,235983	no
XLOC_003675	g278	27,3364	16,3776	-0,7391	-1,20387	0,038	0,164605	no
XLOC_003676	g7444	283,771	255,622	-0,150712	-0,258341	0,65405	0,83003	no
XLOC_003677	g7443	71,8431	45,9482	-0,64484	-0,810399	0,1475	0,384102	no
XLOC_003678	g7446	74,1631	37,3337	-0,990222	-1,66964	0,0041	0,0354419	yes
XLOC_003679	g7448	82,62	87,0379	0,0751533	0,123344	0,82985	0,922056	no

XLOC_003680	g7449	117,778	132,424	0,169088	0,287332	0,61025	0,803802	no
XLOC_003681	g7450	14,6244	16,4713	0,171584	0,256135	0,648	0,826625	no
XLOC_003682	g7445	87,5351	58,2163	-0,588439	-0,997327	0,0861	0,276941	no
XLOC_003683	g7447	71,2546	38,9868	-0,869997	-1,01942	0,07455	0,252007	no
XLOC_003684	g7451	48,7741	54,1194	0,15003	0,240264	0,66395	0,835468	no
XLOC_003685	g7452	0	0	0	0	1	1	no
XLOC_003686	g7453	0	0	0	0	1	1	no
XLOC_003687	g7454	3,75781	6,98251	0,893854	1,18721	0,04025	0,170663	no
XLOC_003688	g7455	0,660343	1,03683	0,650894	0	1	1	no
XLOC_003689	g7456	3,37826	4,17294	0,304783	0,351385	0,5425	0,76057	no
XLOC_003690	g7457	80,2412	164,162	1,0327	1,42583	0,0124	0,0776582	no
XLOC_003691	g7458	216,673	137,919	-0,651696	-0,976842	0,0827	0,270976	no
XLOC_003692	g7459	63,8414	79,7399	0,320809	0,508226	0,3773	0,635496	no
XLOC_003693	g7460	56,6456	61,5079	0,118809	0,188205	0,7515	0,883013	no
XLOC_003694	g7461	1,50337	3,68176	1,2922	1,48536	0,01755	0,0987062	no
XLOC_003695	g7462	15,3761	7,22884	-1,08885	-1,08158	0,0619	0,224219	no
XLOC_003696	g7463	0	0,234827	inf	0	1	1	no
XLOC_003697	g7464	0	0	0	0	1	1	no
XLOC_003698	g7465	0	0,0405098	inf	0	1	1	no
XLOC_003699	g7467	2349,84	2395,34	0,027673	0,0354924	0,9485	0,976646	no
XLOC_003700	g7468	70,3458	43,1482	-0,705165	-1,09617	0,0475	0,18946	no
XLOC_003701	g7466	104,4	126,443	0,27636	0,435633	0,44555	0,691747	no
XLOC_003702	g7469	0	0	0	0	1	1	no
XLOC_003703	g2148	0	0	0	0	1	1	no
XLOC_003704	g7470	12,7079	6,20463	-1,03431	-1,41671	0,0148	0,0877633	no
XLOC_003705	g7471	55,4873	52,5056	-0,0796874	-0,124178	0,8222	0,919172	no
XLOC_003706	g7474	45,5393	47,2861	0,0543029	0,0895927	0,87455	0,943538	no
XLOC_003707	g7472	68,4359	63,1429	-0,116133	-0,131656	0,8215	0,918912	no
XLOC_003708	g7473	46,4781	66,2273	0,510877	0,87277	0,12005	0,341016	no
XLOC_003709	g7475	0	0	0	0	1	1	no
XLOC_003710	g7476	7,34939	16,9625	1,20665	0,870571	0,22805	0,486203	no
XLOC_003711	g2149	12,0599	12,6321	0,0668858	0,0954708	0,86	0,935634	no
XLOC_003712	g282	0	0	0	0	1	1	no
XLOC_003713	g7478	28,8533	11,6821	-1,30444	-2,01108	0,00035	0,00552572	yes
XLOC_003714	g7477	15,1579	9,24468	-0,713376	-1,07278	0,06285	0,226491	no
XLOC_003715	g7479	19,465	29,9295	0,620683	0,95383	0,0859	0,276731	no
XLOC_003716	g7480	0,782582	3,02706	1,9516	2,03639	0,00205	0,0212577	yes
XLOC_003717	g7481	24,403	12,7622	-0,935187	-1,11416	0,057	0,213968	no
XLOC_003718	g7483	213,732	182,574	-0,227323	-0,353595	0,537	0,75585	no
XLOC_003719	g7482	63,8434	60,0057	-0,0894365	-0,137919	0,80245	0,908624	no
XLOC_003720	g7484	1,93634	3,49287	0,851085	0,907679	0,11015	0,32337	no
XLOC_003721	g7485	5,1263	9,71391	0,922135	1,03529	0,07525	0,253495	no
XLOC_003722	g7486	18,0337	22,135	0,29563	0,410032	0,46925	0,707556	no
XLOC_003723	g7487	13,5304	8,33051	-0,699731	-0,965497	0,09535	0,295281	no
XLOC_003724	g7489	10,4626	12,4984	0,256509	0,394197	0,4808	0,715629	no
XLOC_003725	g7491	79,037	104,46	0,402348	0,578816	0,28925	0,55259	no
XLOC_003726	g7488	76,077	77,3194	0,0233693	0,0327957	0,9561	0,980564	no
XLOC_003727	g7490	24,2323	31,2729	0,367983	0,55738	0,33155	0,592577	no
XLOC_003728	g7492	31,536	30,4214	-0,0519128	-0,0842621	0,88195	0,947255	no
XLOC_003729	g7493	0	0	0	0	1	1	no
XLOC_003730	g7494	51,6921	224,916	2,12137	2,50139	5,00E-05	0,00120049	yes
XLOC_003731	g7496	40,3744	38,2099	-0,079494	-0,134384	0,81095	0,913039	no
XLOC_003732	g7495	27,794	27,7586	-0,00183952	-0,00317038	0,99545	0,997682	no
XLOC_003733	g7497	206,997	152,478	-0,441004	-0,682922	0,2323	0,490156	no
XLOC_003734	g7499	19,6729	21,6624	0,138987	0,197932	0,724	0,86956	no
XLOC_003735	g7498	0	0	0	0	1	1	no
XLOC_003736	g285	6,75599	4,85758	-0,475929	-0,628057	0,284	0,546579	no
XLOC_003737	g287	193,924	112,037	-0,791514	-1,33532	0,0189	0,104327	no
XLOC_003738	g288	35,068	12,2744	-1,5145	-2,18834	0,00025	0,00430702	yes
XLOC_003739	g289	89,3113	83,757	-0,0926335	-0,159378	0,7767	0,895794	no
XLOC_003740	g283	21,3828	53,2771	1,31706	2,19523	0,0001	0,00209829	yes
XLOC_003741	g284	24,815	16,6491	-0,575763	-0,841891	0,12395	0,347197	no
XLOC_003742	g286	384,029	434,509	0,178171	0,273247	0,622	0,810192	no
XLOC_003743	g290	16,5714	8,03841	-1,04371	-1,329	0,01685	0,0959229	no
XLOC_003744	g7500	5,10723	5,62142	0,138393	0,160547	0,78555	0,901285	no
XLOC_003745	g7501	0	0	0	0	1	1	no
XLOC_003746	g7502	210,981	238,809	0,178742	0,270569	0,633	0,816463	no
XLOC_003747	g7503	25,6028	19,0639	-0,425459	-0,651317	0,2392	0,497337	no
XLOC_003748	g7504	0	2,10592	inf	0	1	1	no
XLOC_003749	g7505	0	0	0	0	1	1	no
XLOC_003750	g7506	220,82	232,415	0,0738331	0,117515	0,84	0,926574	no
XLOC_003751	g7507	0	0	0	0	1	1	no
XLOC_003752	g7508	0	0,418019	inf	0	1	1	no
XLOC_003753	g7510	166,355	142,732	-0,220948	-0,325857	0,5655	0,77483	no
XLOC_003754	g7512	58,0745	36,6352	-0,664676	-1,10162	0,04895	0,193904	no
XLOC_003755	g7513	49,4649	40,2548	-0,297246	-0,471711	0,40095	0,654912	no
XLOC_003756	g7515	80,0289	30,8308	-1,37615	-2,34951	0,0001	0,00209829	yes
XLOC_003757	g7517	73,9058	122,525	0,729321	1,24318	0,028	0,135891	no
XLOC_003758	g7519	52,744	94,0595	0,834566	1,33088	0,02055	0,111185	no
XLOC_003759	g7509	28,7768	21,6604	-0,409848	-0,623806	0,27075	0,532269	no
XLOC_003760	g7511	13,5435	11,4931	-0,236842	-0,39624	0,48435	0,718043	no
XLOC_003761	g7514	33,0675	14,2425	-1,21522	-1,91683	0,00145	0,0164423	yes
XLOC_003762	g7516	193,039	117,071	-0,721506	-1,16115	0,03875	0,166386	no
XLOC_003763	g7518	37,9788	95,1511	1,32503	2,13686	5,00E-05	0,00120049	yes
XLOC_003764	g7520	112,501	214,732	0,9326	1,4943	0,01045	0,068504	no
XLOC_003765	g7521	3,08277	2,8153	-0,13094	-0,111787	0,84635	0,929972	no
XLOC_003766	g7522	112,581	88,894	-0,3408	-0,566009	0,33195	0,592663	no
XLOC_003767	g7523	7,08863	5,02625	-0,496025	-0,489727	0,39595	0,649988	no
XLOC_003768	g7524	8,1084	8,15716	0,00864839	0,0128348	0,9826	0,991485	no
XLOC_003769	g7525	15,5577	24,1471	0,634227	1,00101	0,0755	0,253989	no
XLOC_003770	g7526	1,45034	2,88836	0,993859	0,648465	0,2911	0,553778	no
XLOC_003771	g7527	52,4171	63,4127	0,274735	0,450232	0,42145	0,671748	no

XLOC_003772	g7528	27,1683	23,1551	-0,230597	-0,369802	0,50265	0,730938	no
XLOC_003773	g7529	19,669	23,1193	0,233172	0,326409	0,56835	0,776676	no
XLOC_003774	g7531	58,0135	93,7758	0,692826	1,1727	0,0463	0,186491	no
XLOC_003775	g7533	103	100,544	-0,0348193	-0,0578026	0,9141	0,963407	no
XLOC_003776	g7530	42,7117	92,3961	1,1132	1,7283	0,0026	0,0255065	yes
XLOC_003777	g7532	20,6064	6,68628	-1,62382	-2,26043	0,00065	0,00901554	yes
XLOC_003778	g7534	41,61	36,8271	-0,176163	-0,298757	0,5879	0,78847	no
XLOC_003779	g7535	28,6576	8,22341	-1,80111	-2,43051	5,00E-05	0,00120049	yes
XLOC_003780	g2151	51,4968	83,2532	0,693022	1,1639	0,0427	0,17745	no
XLOC_003781	g2153	80,6109	71,4467	-0,174108	-0,303577	0,5992	0,7958	no
XLOC_003782	g2155	1,56177	0,425143	-1,87717	-1,60884	0,011	0,0711126	no
XLOC_003783	g2157	70,5045	52,4306	-0,427308	-0,743014	0,2077	0,462294	no
XLOC_003784	g2159	20,2441	32,4453	0,680507	1,12084	0,0546	0,20817	no
XLOC_003785	g2160	38,5639	39,5921	0,0379589	0,0539282	0,924	0,966622	no
XLOC_003786	g2161	43,93	53,1814	0,275716	0,440009	0,44955	0,694772	no
XLOC_003787	g2163	213,028	226,226	0,0867203	0,138051	0,8083	0,911624	no
XLOC_003788	g2164	249,67	224,139	-0,15563	-0,260623	0,6468	0,825909	no
XLOC_003789	g2165	72,4084	144,248	0,994324	1,39993	0,0107	0,0697703	no
XLOC_003790	g2150	13,8078	13,8984	0,00943468	0,0143728	0,97985	0,990233	no
XLOC_003791	g2152	29,8048	31,0851	0,0606811	0,10341	0,8586	0,935373	no
XLOC_003792	g2154	23,3059	25,3115	0,1191	0,198449	0,7322	0,873552	no
XLOC_003793	g2156	14,0354	6,40228	-1,13241	-1,42641	0,0171	0,0969527	no
XLOC_003794	g2158	31,0288	94,3369	1,60421	2,58714	5,00E-05	0,00120049	yes
XLOC_003795	g2162	46,8395	44,2078	-0,0834243	-0,141824	0,81225	0,914189	no
XLOC_003796	g2166	28,9511	21,7598	-0,411955	-0,568043	0,31185	0,573032	no
XLOC_003797	g7536	0	0,336319	inf	0	1	1	no
XLOC_003798	g7537	52,5036	42,3501	-0,31005	-0,491647	0,3793	0,636522	no
XLOC_003799	g7538	2648,77	995,786	-1,41141	-1,42798	0,00895	0,0617198	no
XLOC_003800	g7539	7,08889	6,17459	-0,199216	-0,19836	0,73465	0,874834	no
XLOC_003801	g7541	12,4246	4,8679	-1,35182	-1,87621	0,0014	0,016042	yes
XLOC_003802	g7540	11,7551	9,62879	-0,287866	-0,343231	0,52085	0,744468	no
XLOC_003803	g7542	42,2239	38,5124	-0,132737	-0,218761	0,70025	0,85752	no
XLOC_003804	g7543	5870,21	3814,58	-0,621887	-0,818712	0,14715	0,384006	no
XLOC_003805	g7544	0,391285	0,53064	0,439513	0	1	1	no
XLOC_003806	g7545	109,862	104,174	-0,0766969	-0,132312	0,82135	0,918849	no
XLOC_003807	g7546	18,0564	36,7441	1,025	1,72393	0,00245	0,0243512	yes
XLOC_003808	g7547	15,1831	21,0578	0,471883	0,756066	0,1842	0,432324	no
XLOC_003809	g7548	35,3549	29,7505	-0,248995	-0,357604	0,53435	0,753817	no
XLOC_003810	g7550	101,224	293,742	1,537	2,46551	0,0001	0,00209829	yes
XLOC_003811	g7549	69,1769	56,5384	-0,29106	-0,498183	0,3788	0,636385	no
XLOC_003812	g7551	5,74797	8,12245	0,498863	0,579745	0,3176	0,578204	no
XLOC_003813	g7552	26,1403	14,7122	-0,829259	-1,3322	0,022	0,116526	no
XLOC_003814	g7555	274,535	287,354	0,0658396	0,112838	0,84185	0,927787	no
XLOC_003815	g7557	20,7349	15,6407	-0,406756	-0,652085	0,2609	0,521008	no
XLOC_003816	g7553	20,9875	31,1119	0,567935	0,907853	0,10755	0,319224	no
XLOC_003817	g7554	24,6819	23,3334	-0,0810596	-0,135792	0,8083	0,911624	no
XLOC_003818	g7556	29,2062	26,7284	-0,127902	-0,216845	0,6977	0,856213	no
XLOC_003819	g7558	20,6459	17,3977	-0,246962	-0,377352	0,52215	0,745347	no
XLOC_003820	g7559	26,339	137,509	2,38425	2,56855	5,00E-05	0,00120049	yes
XLOC_003821	g7560	8,28983	9,20237	0,150662	0,190713	0,7239	0,86956	no
XLOC_003822	g7561	18,7109	12,1413	-0,623955	-0,919556	0,0999	0,30297	no
XLOC_003823	g7562	0	0	0	0	1	1	no
XLOC_003824	g7563	0	0	0	0	1	1	no
XLOC_003825	g7564	4,66056	4,76574	0,0321967	0,0390784	0,9507	0,978193	no
XLOC_003826	g7566	526,038	751,372	0,514362	0,69699	0,20025	0,45251	no
XLOC_003827	g7565	46,5435	55,2401	0,247137	0,417274	0,4648	0,705296	no
XLOC_003828	g7567	0,356692	0,31373	-0,185156	0	1	1	no
XLOC_003829	g7568	4,08954	0,504307	-3,01957	-2,0191	0,20975	0,464639	no
XLOC_003830	g7570	0	0	0	0	1	1	no
XLOC_003831	g7569	0	0	0	0	1	1	no
XLOC_003832	g7571	0	0	0	0	1	1	no
XLOC_003833	g7572	0,406398	0,729309	0,843637	0	1	1	no
XLOC_003834	g7573	108,113	61,8322	-0,806105	-1,37045	0,01525	0,089406	no
XLOC_003835	g7574	0,0179703	0	#NAME?	0	1	1	no
XLOC_003836	g7575	0	0	0	0	1	1	no
XLOC_003837	g2167	191,973	154,615	-0,312223	-0,532909	0,34095	0,601208	no
XLOC_003838	g2169	41,6293	57,3644	0,462555	0,788284	0,1576	0,397032	no
XLOC_003839	g2172	66,7696	62,1717	-0,102934	-0,161561	0,76795	0,891825	no
XLOC_003840	g2168	247,749	348,181	0,490957	0,821869	0,14675	0,383778	no
XLOC_003841	g2170	40,5971	40,1803	-0,0148878	-0,0254957	0,96515	0,984192	no
XLOC_003842	g2171	25,113	36,6358	0,544822	0,358802	0,5431	0,760875	no
XLOC_003843	g2173	160,535	196,628	0,292582	0,495243	0,3625	0,62266	no
XLOC_003844	g7576	64,141	189,227	1,5608	2,23134	0,0001	0,00209829	yes
XLOC_003845	g7577	0	0	0	0	1	1	no
XLOC_003846	g7578	13,5436	3,78931	-1,83761	-1,99357	0,0507	0,198594	no
XLOC_003847	g7579	0	0	0	0	1	1	no
XLOC_003848	g7580	195,406	114,853	-0,766689	-0,942112	0,1021	0,307647	no
XLOC_003849	g7581	384,976	678,979	0,8186	1,20204	0,02935	0,139843	no
XLOC_003850	g7582	891,925	390,818	-1,19043	-2,0108	0,00085	0,011085	yes
XLOC_003851	g7585	54,2289	61,1129	0,172415	0,289988	0,6031	0,798819	no
XLOC_003852	g7588	31,3071	35,5255	0,182368	0,295025	0,59125	0,790276	no
XLOC_003853	g7589	98,2518	164,824	0,746366	1,26732	0,02545	0,127967	no
XLOC_003854	g7583	226,302	427,699	0,918345	1,38918	0,01415	0,0852472	no
XLOC_003855	g7584	19,9408	27,0566	0,440255	0,595047	0,2984	0,560566	no
XLOC_003856	g7586	8,51051	3,67273	-1,21239	-1,51499	0,01185	0,075124	no
XLOC_003857	g7587	82,2026	105,187	0,355699	0,586547	0,30915	0,570327	no
XLOC_003858	g7590	77,6103	73,6387	-0,0757847	-0,121579	0,82295	0,919278	no
XLOC_003859	g7591	80,4237	118,988	0,565121	0,893545	0,12475	0,348123	no
XLOC_003860	g7592	16,7269	48,7232	1,54244	2,39672	5,00E-05	0,00120049	yes
XLOC_003861	g7593	0	0	0	0	1	1	no
XLOC_003862	g7595	111,566	132,763	0,25096	0,427795	0,45715	0,701012	no
XLOC_003863	g7594	1597,52	3611,64	1,17682	1,35926	0,0162	0,0931405	no

XLOC_003864	g7596	5,12135	6,90915	0,431986	0,516913	0,37035	0,629539	no
XLOC_003865	g7597	4,31617	3,84526	-0,166672	-0,211747	0,7151	0,865069	no
XLOC_003866	g7598	1,48876	2,4764	0,734138	0,766699	0,17425	0,420014	no
XLOC_003867	g7599	0	0	0	0	1	1	no
XLOC_003868	g291	38,2085	39,606	0,0518272	0,0850406	0,88165	0,947197	no
XLOC_003869	g7600	16,2519	23,508	0,532542	0,727229	0,20455	0,457976	no
XLOC_003870	g7601	0	0	0	0	1	1	no
XLOC_003871	g7602	12,644	10,8808	-0,216665	-0,318335	0,5803	0,783024	no
XLOC_003872	g7603	0	0	0	0	1	1	no
XLOC_003873	g2174	48,8337	52,0291	0,091444	0,143429	0,80095	0,908537	no
XLOC_003874	g2175	6,63619	3,43724	-0,949107	-1,38014	0,0152	0,0892194	no
XLOC_003875	g2177	174,113	294,296	0,757246	0,980045	0,09105	0,286666	no
XLOC_003876	g2176	93,1306	84,7191	-0,136568	-0,217084	0,706	0,860698	no
XLOC_003877	g7604	50,5006	16,8217	-1,58598	-1,98703	0,00155	0,0171215	yes
XLOC_003878	g7606	13,421	20,1615	0,587108	0,986417	0,08255	0,270574	no
XLOC_003879	g7605	40,6312	43,0916	0,0848204	0,123114	0,8318	0,922663	no
XLOC_003880	g7607	116,532	159,2	0,45012	0,743014	0,20755	0,462268	no
XLOC_003881	g292	17,6332	22,4038	0,345447	0,590347	0,30125	0,563145	no
XLOC_003882	g295	77,0895	87,0367	0,17509	0,247075	0,6631	0,835468	no
XLOC_003883	g297	167,835	63,2949	-1,40689	-1,97452	0,0009	0,0115984	yes
XLOC_003884	g293	7,99306	5,74026	-0,477631	-0,541411	0,3546	0,615463	no
XLOC_003885	g294	1,76314	3,15338	0,838754	0,754642	0,1744	0,420169	no
XLOC_003886	g296	113,578	102,151	-0,152985	-0,24729	0,65895	0,832458	no
XLOC_003887	g298	48,0893	64,6046	0,425921	0,674935	0,24315	0,50173	no
XLOC_003888	g7608	0	0	0	0	1	1	no
XLOC_003889	g2178	2,47543	2,97871	0,267011	0,349163	0,5498	0,765215	no
XLOC_003890	g2179	3,17742	0,679057	-2,22625	-1,40906	0,2445	0,503031	no
XLOC_003891	g7609	0,609616	1,10473	0,857717	0,956283	0,10555	0,314759	no
XLOC_003892	g7610	22,5988	17,8633	-0,339244	-0,523103	0,3506	0,611179	no
XLOC_003893	g7611	13,0056	12,9229	-0,00919747	-0,0145834	0,9801	0,990233	no
XLOC_003894	g7612	51,9978	26,6615	-0,963694	-1,54589	0,0091	0,0622298	no
XLOC_003895	g2180	33,1754	38,8014	0,225994	0,372215	0,5181	0,742916	no
XLOC_003896	g2181	26,2376	24,3604	-0,107098	-0,170926	0,7652	0,890163	no
XLOC_003897	g7613	82,1503	100,693	0,293628	0,473846	0,39455	0,648995	no
XLOC_003898	g7614	0	0	0	0	1	1	no
XLOC_003899	g7615	89,3781	70,8999	-0,334138	-0,400516	0,489	0,721885	no
XLOC_003900	g7616	10,9368	13,1852	0,26973	0,303638	0,607	0,800918	no
XLOC_003901	g7617	0	0	0	0	1	1	no
XLOC_003902	g7618	9,83132	14,6496	0,575407	0,609122	0,3064	0,567386	no
XLOC_003903	g7619	12,7183	6,31335	-1,01042	-1,54288	0,0095	0,0641609	no
XLOC_003904	g2182	49,0336	44,316	-0,145945	-0,186792	0,73705	0,87578	no
XLOC_003905	g7620	104,326	83,9597	-0,313331	-0,51825	0,3636	0,623459	no
XLOC_003906	g7621	0	0	0	0	1	1	no
XLOC_003907	g7622	0	0	0	0	1	1	no
XLOC_003908	g2185	120,341	161,044	0,420328	0,558543	0,32305	0,583565	no
XLOC_003909	g2183	1,98147	1,62524	-0,285915	-0,305363	0,5904	0,790204	no
XLOC_003910	g2184	113,688	82,6783	-0,459494	-0,771677	0,17855	0,425676	no
XLOC_003911	g2186	82,4209	61,4834	-0,422813	-0,686133	0,2272	0,48555	no
XLOC_003912	g7623	14,1278	15,3977	0,124179	0,175981	0,75355	0,883777	no
XLOC_003913	g7624	441,109	649,991	0,559286	0,79578	0,15505	0,392851	no
XLOC_003914	g7626	23,0171	37,5742	0,707038	1,16302	0,0426	0,177109	no
XLOC_003915	g7627	2,02505	2,51327	0,311609	0,317465	0,582	0,784306	no
XLOC_003916	g7629	61,2452	112,371	0,875595	1,22308	0,03515	0,156826	no
XLOC_003917	g7630	83,2334	60,2342	-0,466579	-0,75504	0,17205	0,417065	no
XLOC_003918	g7633	24,3883	34,1975	0,487704	0,820344	0,159	0,39933	no
XLOC_003919	g7636	70,8356	103,093	0,541405	0,877722	0,12155	0,343983	no
XLOC_003920	g7637	192,563	208,129	0,112145	0,175556	0,74885	0,881846	no
XLOC_003921	g7638	74,1773	108,237	0,545139	0,925897	0,10835	0,319999	no
XLOC_003922	g7625	28,6923	35,013	0,287228	0,471471	0,4089	0,661624	no
XLOC_003923	g7628	121,463	138,132	0,185521	0,317542	0,5764	0,780604	no
XLOC_003924	g7631	77,2735	103,017	0,414842	0,71869	0,20675	0,461219	no
XLOC_003925	g7632	47,9339	49,9041	0,0581107	0,0948733	0,8654	0,938305	no
XLOC_003926	g7634	65,5844	49,4563	-0,407198	-0,662501	0,2362	0,494137	no
XLOC_003927	g7635	61,7038	126,248	1,03283	1,77129	0,00145	0,0164423	yes
XLOC_003928	g7639	183,427	126,605	-0,534876	-0,851191	0,14415	0,380116	no
XLOC_003929	g7640	0	0,213843	inf	0	1	1	no
XLOC_003930	g7641	193,092	198,71	0,0413747	0,0583274	0,9197	0,96459	no
XLOC_003931	g7642	23,1794	17,4107	-0,412871	-0,539777	0,342	0,602087	no
XLOC_003932	g7643	6,55422	2,34319	-1,48395	-1,91963	0,00125	0,0146655	yes
XLOC_003933	g7644	72,7199	52,4333	-0,471867	-0,746882	0,19575	0,447153	no
XLOC_003934	g7645	42,9511	37,5622	-0,193415	-0,212823	0,71275	0,863993	no
XLOC_003935	g2187	1309,07	843,709	-0,63372	-0,735714	0,192	0,442199	no
XLOC_003936	g7646	1,81948	0	#NAME?	#NAME?	0,00445	0,0377692	yes
XLOC_003937	g2188	5,66377	6,75393	0,253964	0,372798	0,5226	0,745487	no
XLOC_003938	g2189	86,9598	52,0887	-0,739378	-1,13107	0,044	0,18056	no
XLOC_003939	g7647	16,9895	19,5029	0,199045	0,306501	0,5851	0,786218	no
XLOC_003940	g7648	6,90179	5,32076	-0,37534	-0,437355	0,42865	0,677386	no
XLOC_003941	g7649	30,8062	55,1297	0,839609	1,20254	0,02725	0,133597	no
XLOC_003942	g2191	71,0836	42,4873	-0,742484	-1,27116	0,0256	0,128392	no
XLOC_003943	g2190	27,7396	24,76	-0,163938	-0,2293	0,6838	0,84795	no
XLOC_003944	g2192	1,43882	8,17884	2,50701	2,56618	0,0001	0,00209829	yes
XLOC_003945	g2194	78,0745	66,177	-0,238521	-0,370932	0,4964	0,726367	no
XLOC_003946	g2197	76,615	30,4888	-1,32935	-2,08574	0,00055	0,00788467	yes
XLOC_003947	g2193	5020,42	6771,08	0,431579	0,536028	0,3611	0,621451	no
XLOC_003948	g2195	43,8123	43,6201	-0,00634543	-0,0107523	0,98315	0,991836	no
XLOC_003949	g2196	8,04389	9,60494	0,255883	0,3536	0,5295	0,750316	no
XLOC_003950	g7650	154,056	182,389	0,243565	0,411008	0,4715	0,709164	no
XLOC_003951	g7651	1,23955	2,95481	1,25325	1,15347	0,1993	0,451802	no
XLOC_003952	g7652	0,438025	2,1074	2,26638	1,03727	0,02225	0,117534	no
XLOC_003953	g7653	83,6161	37,5681	-1,15427	-1,90033	0,00115	0,0138564	yes
XLOC_003954	g7654	30,8382	28,3546	-0,121133	-0,190512	0,74525	0,880033	no
XLOC_003955	g7655	42,5746	72,3452	0,764905	1,14934	0,05005	0,19699	no

XLOC_003956	g7656	69,8297	53,6659	-0,379837	-0,634973	0,26665	0,527597	no
XLOC_003957	g7657	3,73788	2,07879	-0,846472	-1,17853	0,04185	0,175029	no
XLOC_003958	g7658	9,13361	7,87991	-0,213007	-0,322578	0,55445	0,767724	no
XLOC_003959	g7659	0	0	0	0	1	1	no
XLOC_003960	g7660	0	0	0	0	1	1	no
XLOC_003961	g7661	0	0	0	0	1	1	no
XLOC_003962	g7662	0	0	0	0	1	1	no
XLOC_003963	g7663	0,0992615	0	#NAME?	0	1	1	no
XLOC_003964	g7664	132,596	10,5768	-3,64805	-4,9077	5,00E-05	0,00120049	yes
XLOC_003965	g7665	10,693	9,40808	-0,184694	-0,147176	0,803	0,908624	no
XLOC_003966	g7666	7,75972	6,25369	-0,311297	-0,399021	0,49325	0,72417	no
XLOC_003967	g7667	3,30727	6,59082	0,994819	0,866742	0,15	0,387278	no
XLOC_003968	g7668	13,4685	13,7122	0,025868	0,029876	0,9525	0,978815	no
XLOC_003969	g7670	374,062	661,102	0,821596	1,22899	0,03325	0,151375	no
XLOC_003970	g7669	17,2793	9,65603	-0,839544	-1,20347	0,03445	0,154404	no
XLOC_003971	g7672	4,27697	25,4578	2,57345	3,31141	5,00E-05	0,00120049	yes
XLOC_003972	g7674	47,715	80,2949	0,750866	1,21166	0,03615	0,15919	no
XLOC_003973	g7671	39,9861	90,4031	1,17687	1,99676	0,00055	0,00788467	yes
XLOC_003974	g7673	263,888	342,661	0,376859	0,584443	0,30175	0,563557	no
XLOC_003975	g7675	14,3471	10,3856	-0,466173	-0,59048	0,30845	0,569464	no
XLOC_003976	g7676	0	0,0959487	inf	0	1	1	no
XLOC_003977	g2198	278,835	440,491	0,659701	0,929735	0,0957	0,295526	no
XLOC_003978	g7677	1,45076	1,31791	-0,138558	-0,179301	0,7526	0,883507	no
XLOC_003979	g7678	0	0	0	0	1	1	no
XLOC_003980	g2199	0,0709684	0	#NAME?	0	1	1	no
XLOC_003981	g7680	3,92563	4,51912	0,203118	0,226974	0,6985	0,856445	no
XLOC_003982	g7679	1,35809	1,49228	0,135943	0,129483	0,82825	0,921598	no
XLOC_003983	g7681	11,4732	9,48607	-0,274381	-0,379818	0,5134	0,739778	no
XLOC_003984	g7682	6,53765	7,47423	0,193152	0,24199	0,66435	0,83565	no
XLOC_003985	g7683	0	0	0	0	1	1	no
XLOC_003986	g7684	0	0	0	0	1	1	no
XLOC_003987	g7685	0	0	0	0	1	1	no
XLOC_003988	g7686	54,9147	35,3969	-0,633567	-1,04547	0,071	0,245069	no
XLOC_003989	g7687	198,644	49,982	-1,99071	-2,57339	0,00015	0,00294012	yes
XLOC_003990	g7688	0	0	0	0	1	1	no
XLOC_003991	g7689	10,3192	11,1535	0,112171	0,139787	0,79925	0,907785	no
XLOC_003992	g2201	6,07894	13,8099	1,18381	1,65692	0,00555	0,0439524	yes
XLOC_003993	g2203	13,306	11,7706	-0,176891	-0,225501	0,7023	0,858746	no
XLOC_003994	g2204	26,0044	29,1833	0,166386	0,258848	0,6493	0,827531	no
XLOC_003995	g2205	45,4937	51,376	0,175427	0,291076	0,61245	0,805014	no
XLOC_003996	g2200	278,693	222,057	-0,32775	-0,512316	0,352	0,612641	no
XLOC_003997	g2202	941,07	1081,63	0,200834	0,275119	0,6169	0,807943	no
XLOC_003998	g7690	0	0	0	0	1	1	no
XLOC_003999	g7691	2,15845	8,44007	1,96726	1,97178	0,00205	0,0212577	yes
XLOC_004000	g7692	0,0436066	0,30606	2,81119	0	1	1	no
XLOC_004001	g7693	59,2826	73,9173	0,318303	0,531275	0,34995	0,610718	no
XLOC_004002	g7694	0	0	0	0	1	1	no
XLOC_004003	g7695	0	0	0	0	1	1	no
XLOC_004004	g7696	0	0	0	0	1	1	no
XLOC_004005	g300	87,058	106,925	0,296545	0,489293	0,38905	0,643936	no
XLOC_004006	g301	53,7437	42,4312	-0,34097	-0,578953	0,30275	0,564459	no
XLOC_004007	g299	7,8005	12,5195	0,682539	1,01633	0,0905	0,285667	no
XLOC_004008	g7697	31,7659	20,9046	-0,603658	-0,676529	0,2557	0,515162	no
XLOC_004009	g2206	29,6963	33,3922	0,16923	0,228348	0,68015	0,846112	no
XLOC_004010	g2208	18,3607	23,2401	0,339992	0,530038	0,3592	0,619938	no
XLOC_004011	g2209	7,95784	5,76446	-0,465191	-0,582346	0,3166	0,577009	no
XLOC_004012	g2210	13,1682	15,6686	0,250824	0,392504	0,48805	0,721133	no
XLOC_004013	g2211	1,20309	2,5585	1,08855	0,933875	0,1121	0,327042	no
XLOC_004014	g2214	446,514	149,642	-1,57719	-1,98921	0,00115	0,0138564	yes
XLOC_004015	g2216	3,79781	5,1749	0,446363	0,411639	0,48885	0,721772	no
XLOC_004016	g2217	1083,23	558,032	-0,956918	-1,17503	0,0365	0,160228	no
XLOC_004017	g2219	27,9938	67,0303	1,25971	1,97848	0,00075	0,0100752	yes
XLOC_004018	g2221	1,26547	0,788002	-0,683397	-0,68228	0,22795	0,486095	no
XLOC_004019	g2207	97,5821	117,643	0,269733	0,342255	0,5596	0,77105	no
XLOC_004020	g2212	5,3213	14,5304	1,44922	1,83278	0,00245	0,0243512	yes
XLOC_004021	g2213	8,84571	11,5768	0,38819	0,587553	0,3205	0,580544	no
XLOC_004022	g2215	15,0265	8,73681	-0,782326	-1,21814	0,02805	0,135891	no
XLOC_004023	g2218	329,056	267,38	-0,299441	-0,415727	0,4678	0,707282	no
XLOC_004024	g2220	55,12	39,2075	-0,491448	-0,780878	0,1665	0,409782	no
XLOC_004025	g7698	21,9572	27,8009	0,340437	0,528839	0,36025	0,620859	no
XLOC_004026	g7700	2938,04	4224,67	0,523983	0,473397	0,4182	0,669829	no
XLOC_004027	g7699	6,31407	16,5178	1,38738	1,33844	0,03395	0,153141	no
XLOC_004028	g7701	108,952	127,552	0,227389	0,345082	0,54015	0,75862	no
XLOC_004029	g7702	0	0	0	0	1	1	no
XLOC_004030	g7703	18,5219	15,5506	-0,252262	-0,41389	0,4457	0,691871	no
XLOC_004031	g2222	0	95,5599	inf	#NAME?	0,0005	0,00733931	yes
XLOC_004032	g7704	45	26,4476	-0,766788	-1,09947	0,0573	0,214357	no
XLOC_004033	g7706	22,3524	21,5337	-0,0538318	-0,0839404	0,88525	0,94862	no
XLOC_004034	g7705	273,714	332,937	0,282579	0,485108	0,3911	0,646031	no
XLOC_004035	g2224	28,2481	21,1411	-0,418103	-0,644607	0,25065	0,509287	no
XLOC_004036	g2225	11,5901	9,91404	-0,225344	-0,324668	0,57715	0,781041	no
XLOC_004037	g2227	1080,23	1521,52	0,494171	0,720651	0,19455	0,445957	no
XLOC_004038	g2223	20,8963	22,5921	0,112575	0,191691	0,732	0,873419	no
XLOC_004039	g2226	130,479	261,304	1,00192	1,43819	0,01325	0,0814746	no
XLOC_004040	g2228	131,135	120,85	-0,117835	-0,201079	0,72155	0,86823	no
XLOC_004041	g2229	13,5305	13,5914	0,00647643	0,00740644	0,99205	0,995293	no
XLOC_004042	g2230	3,22551	4,14907	0,36326	0,373343	0,50125	0,730308	no
XLOC_004043	g2231	100,352	65,5877	-0,613575	-0,803709	0,167	0,410395	no
XLOC_004044	g7707	0	0	0	0	1	1	no
XLOC_004045	g7709	61,8604	30,7467	-1,00859	-1,67379	0,0028	0,0269305	yes
XLOC_004046	g7708	104,943	163,993	0,644023	0,98284	0,09395	0,292514	no
XLOC_004047	g7710	30,2486	14,58	-1,05288	-1,36157	0,018	0,100603	no

XLOC_004048	g7712	25,4462	41,3281	0,699675	1,08083	0,05495	0,208908	no
XLOC_004049	g7711	26,3669	32,7916	0,314598	0,462009	0,4181	0,669829	no
XLOC_004050	g7713	89,2616	81,4813	-0,131571	-0,22577	0,69725	0,856211	no
XLOC_004051	g7714	138,705	151,283	0,125227	0,173065	0,77585	0,895387	no
XLOC_004052	g7715	37,6229	44,9667	0,257245	0,41623	0,47975	0,715436	no
XLOC_004053	g7716	35,6282	15,8076	-1,1724	-1,88747	0,001	0,0126221	yes
XLOC_004054	g7717	8,71929	10,9159	0,324146	0,418063	0,477	0,713176	no
XLOC_004055	g302	0	0	0	0	1	1	no
XLOC_004056	g7718	0	0	0	0	1	1	no
XLOC_004057	g7719	0	0	0	0	1	1	no
XLOC_004058	g7720	9,50294	6,67253	-0,510139	-0,711927	0,2151	0,470651	no
XLOC_004059	g7721	0	0	0	0	1	1	no
XLOC_004060	g7722	0	0	0	0	1	1	no
XLOC_004061	g7724	22,5124	28,5306	0,341789	0,559492	0,3371	0,597208	no
XLOC_004062	g7725	1,83165	1,98248	0,114168	0,135905	0,8211	0,918849	no
XLOC_004063	g7728	37,5018	27,3362	-0,456144	-0,731563	0,2086	0,463556	no
XLOC_004064	g7723	32,2864	76,5477	1,24543	1,64985	0,00465	0,0389616	yes
XLOC_004065	g7726	27,771	34,0676	0,29482	0,475652	0,3913	0,646031	no
XLOC_004066	g7727	57,0865	22,1799	-1,3639	-1,93518	0,0012	0,0143358	yes
XLOC_004067	g7729	5,18485	17,7461	1,77513	1,73696	0,06165	0,223809	no
XLOC_004068	g7730	72,4958	55,0665	-0,396721	-0,631472	0,26105	0,521008	no
XLOC_004069	g7731	1,05603	0	#NAME?	0	1	1	no
XLOC_004070	g7732	101,963	88,0666	-0,211385	-0,257718	0,6548	0,830874	no
XLOC_004071	g2232	108,57	92,5041	-0,231033	-0,371736	0,52035	0,744115	no
XLOC_004072	g7733	0	0	0	0	1	1	no
XLOC_004073	g7734	12,108	17,0973	0,497805	0,534891	0,37015	0,629539	no
XLOC_004074	g7735	55,4867	62,8206	0,179097	0,306209	0,5896	0,789887	no
XLOC_004075	g7736	56,6994	73,6129	0,376624	0,559619	0,32365	0,584128	no
XLOC_004076	g7737	0	0	0	0	1	1	no
XLOC_004077	g7738	5,99691	4,16832	-0,524753	-0,581454	0,31915	0,579522	no
XLOC_004078	g2233	16,1254	38,4099	1,25215	1,39343	0,01995	0,108718	no
XLOC_004079	g2234	7,8759	14,3409	0,864618	1,09326	0,05985	0,219875	no
XLOC_004080	g7739	5,0851	16,0741	1,66039	2,13884	0,00055	0,00788467	yes
XLOC_004081	g7740	53,3258	42,4297	-0,329761	-0,55187	0,3324	0,593162	no
XLOC_004082	g7741	23,6136	24,4208	0,0484936	0,034054	0,91845	0,964054	no
XLOC_004083	g2235	0	0	0	0	1	1	no
XLOC_004084	g7742	0	0	0	0	1	1	no
XLOC_004085	g7743	86,5453	55,6637	-0,636719	-1,0483	0,0729	0,249343	no
XLOC_004086	g7745	26,2474	37,6062	0,518794	0,874975	0,14055	0,374038	no
XLOC_004087	g7744	23,8231	30,7686	0,369099	0,505107	0,3801	0,637068	no
XLOC_004088	g7746	84,3244	94,7964	0,168882	0,29201	0,61185	0,804764	no
XLOC_004089	g7747	53,725	45,0525	-0,253986	-0,427493	0,46075	0,702027	no
XLOC_004090	g7748	0	0	0	0	1	1	no
XLOC_004091	g2237	0,387721	0,444951	0,198625	0	1	1	no
XLOC_004092	g2238	15,9992	10,0005	-0,677926	-1,07329	0,0589	0,217443	no
XLOC_004093	g2236	108,713	180,581	0,732127	1,14468	0,04745	0,189337	no
XLOC_004094	g7749	35,7798	37,9635	0,0854683	0,0907819	0,87805	0,945394	no
XLOC_004095	g2239	41,1891	36,0296	-0,19308	-0,321598	0,56305	0,772944	no
XLOC_004096	g2240	9,52025	97,5549	3,35714	4,85916	5,00E-05	0,00120049	yes
XLOC_004097	g7750	0	0	0	0	1	1	no
XLOC_004098	g7751	0	0	0	0	1	1	no
XLOC_004099	g7752	1208,71	2687,63	1,15286	1,47027	0,0148	0,0877633	no
XLOC_004100	g7753	0	0,636411	inf	0	1	1	no
XLOC_004101	g303	52,8956	66,3808	0,32762	0,555089	0,3258	0,586607	no
XLOC_004102	g304	189,584	163,372	-0,214679	-0,355733	0,5284	0,749406	no
XLOC_004103	g306	80,2428	139,611	0,798966	1,31512	0,0213	0,114111	no
XLOC_004104	g308	65,0057	45,999	-0,498962	-0,855789	0,13465	0,364863	no
XLOC_004105	g305	113,96	55,0235	-1,05041	-1,72591	0,00245	0,0243512	yes
XLOC_004106	g307	4,88216	8,0287	0,717647	0,820553	0,1564	0,394515	no
XLOC_004107	g2241	149,58	152,943	0,0320733	0,0417926	0,94325	0,975074	no
XLOC_004108	g2242	17,6258	22,423	0,34729	0,500348	0,366	0,625499	no
XLOC_004109	g7754	0	0	0	0	1	1	no
XLOC_004110	g7755	25,2972	25,8406	0,030659	0,0461637	0,93655	0,972153	no
XLOC_004111	g2243	34,1687	21,3705	-0,677051	-0,827321	0,1897	0,43956	no
XLOC_004112	g2246	1199,9	1857,09	0,630128	0,830127	0,13715	0,369327	no
XLOC_004113	g2247	23,8521	28,8168	0,272797	0,42925	0,46185	0,70294	no
XLOC_004114	g2249	127,747	324,493	1,34489	1,95282	0,00095	0,0120686	yes
XLOC_004115	g2250	91,5949	92,8409	0,0194925	0,0335022	0,9551	0,98046	no
XLOC_004116	g2252	70,2497	107,188	0,609582	1,01734	0,07845	0,261145	no
XLOC_004117	g2253	5,63598	5,2126	-0,112663	-0,157784	0,7844	0,900913	no
XLOC_004118	g2255	25,6356	45,0187	0,812377	0,66022	0,2399	0,497953	no
XLOC_004119	g2257	121,472	97,4501	-0,317891	-0,461091	0,4074	0,660503	no
XLOC_004120	g2259	124,761	122,158	-0,0304176	-0,0504188	0,932	0,970236	no
XLOC_004121	g2261	110,49	161,142	0,544413	0,930906	0,09945	0,302353	no
XLOC_004122	g2263	34,1209	40,2752	0,239235	0,407022	0,4767	0,712986	no
XLOC_004123	g2265	125,668	157,367	0,324512	0,534138	0,35665	0,617124	no
XLOC_004124	g2266	10,3689	4,41858	-1,23061	-1,69588	0,00345	0,0313115	yes
XLOC_004125	g2268	201,998	211,678	0,0675312	0,107803	0,8479	0,930946	no
XLOC_004126	g2270	148,522	156,937	0,0795088	0,12864	0,82515	0,920688	no
XLOC_004127	g2272	55,2162	35,179	-0,650376	-1,11285	0,0489	0,193785	no
XLOC_004128	g2274	147,279	90,0599	-0,709591	-1,0682	0,06005	0,220445	no
XLOC_004129	g2276	38,2106	61,5574	0,687959	1,09001	0,0535	0,205383	no
XLOC_004130	g2279	22,6335	32,0486	0,501804	0,813463	0,1467	0,383749	no
XLOC_004131	g2282	33,9193	23,3048	-0,541479	-0,82104	0,15355	0,391754	no
XLOC_004132	g2284	104,264	140,412	0,42943	0,720325	0,20115	0,453361	no
XLOC_004133	g2286	305,739	221,342	-0,46602	-0,780656	0,16805	0,410959	no
XLOC_004134	g2287	54,3873	55,0649	0,0178621	0,0282853	0,95905	0,981744	no
XLOC_004135	g2289	172,576	354,239	1,03749	1,59123	0,00475	0,0395632	yes
XLOC_004136	g2291	25,7994	35,6874	0,468076	0,769885	0,17915	0,426373	no
XLOC_004137	g2293	55,7205	174,157	1,64411	2,39835	0,0001	0,00209829	yes
XLOC_004138	g2294	14,0477	5,55922	-1,33738	-1,79506	0,0021	0,0216618	yes
XLOC_004139	g2296	26,474	29,8338	0,172375	0,284505	0,6128	0,805258	no

XLOC_004140	g2298	389,981	610,551	0,646708	0,957691	0,09535	0,295281	no
XLOC_004141	g2300	141,052	176,499	0,323435	0,55336	0,33005	0,590901	no
XLOC_004142	g2302	68,8723	48,3538	-0,510296	-0,862882	0,131	0,358734	no
XLOC_004143	g2303	20,1618	30,1402	0,580068	0,872871	0,13035	0,357652	no
XLOC_004144	g2304	40,5038	77,6824	0,93953	1,52654	0,00915	0,0624413	no
XLOC_004145	g2306	27,2695	74,2043	1,44421	2,34896	0,0001	0,00209829	yes
XLOC_004146	g2308	75,0037	63,9616	-0,229754	-0,379366	0,5037	0,731814	no
XLOC_004147	g2310	134,4	146,51	0,124459	0,208525	0,7287	0,871129	no
XLOC_004148	g2313	336,575	507,262	0,591802	0,779367	0,1521	0,389904	no
XLOC_004149	g2314	225,123	343,474	0,609489	0,984277	0,0856	0,27649	no
XLOC_004150	g2315	8,78815	7,77205	-0,177265	-0,163006	0,77735	0,895827	no
XLOC_004151	g2317	643,979	2085,26	1,69514	2,71509	5,00E-05	0,00120049	yes
XLOC_004152	g2319	75,2157	71,5379	-0,0723274	-0,121992	0,8307	0,922276	no
XLOC_004153	g2321	52,787	56,4063	0,0956726	0,154225	0,7806	0,898182	no
XLOC_004154	g2323	63,8473	91,2198	0,514722	0,860253	0,12255	0,345717	no
XLOC_004155	g2326	368,274	446,293	0,277211	0,378732	0,4958	0,725995	no
XLOC_004156	g2327	10,4597	3,80965	-1,45712	-2,25349	0,00025	0,00430702	yes
XLOC_004157	g2328	67,2871	60,1724	-0,161228	-0,264972	0,632	0,816117	no
XLOC_004158	g2330	142,2	140,579	-0,0165422	-0,0275915	0,95955	0,981847	no
XLOC_004159	g2332	43,9237	38,2433	-0,199792	-0,334151	0,55705	0,770021	no
XLOC_004160	g2334	23,3067	24,8155	0,0904957	0,153187	0,78675	0,901522	no
XLOC_004161	g2336	44,5475	60,2051	0,434542	0,745223	0,19605	0,447307	no
XLOC_004162	g2337	30,5422	25,9473	-0,235223	-0,371925	0,5028	0,731048	no
XLOC_004163	g2339	28,3825	25,5799	-0,149989	-0,256283	0,64645	0,825677	no
XLOC_004164	g2341	64,6145	82,8802	0,35917	0,616187	0,2786	0,540139	no
XLOC_004165	g2343	35,7458	27,2152	-0,393359	-0,640184	0,25135	0,510076	no
XLOC_004166	g2345	76,784	114,4	0,575209	0,696236	0,2169	0,473324	no
XLOC_004167	g2347	92,8333	172,342	0,892563	1,2556	0,02695	0,133036	no
XLOC_004168	g2348	29,7522	31,9868	0,104476	0,15311	0,793	0,904567	no
XLOC_004169	g2350	127,468	157,252	0,302943	0,496992	0,38885	0,643822	no
XLOC_004170	g2353	19,9604	36,057	0,853137	1,09875	0,05715	0,214203	no
XLOC_004171	g2244	87,6269	126,136	0,525529	0,840764	0,1417	0,375978	no
XLOC_004172	g2245	966,399	2627,62	1,44307	1,77525	0,0041	0,0354419	yes
XLOC_004173	g2248	37,1026	35,3644	-0,0692235	-0,100938	0,86065	0,935634	no
XLOC_004174	g2251	95,646	170,325	0,832517	1,38846	0,01665	0,0951152	no
XLOC_004175	g2254	136,365	156,941	0,202752	0,353483	0,55095	0,765793	no
XLOC_004176	g2256	39,2273	50,7361	0,371153	0,609143	0,2896	0,552637	no
XLOC_004177	g2258	74,2867	53,7457	-0,466953	-0,788144	0,1689	0,411869	no
XLOC_004178	g2260	647,403	784,937	0,277912	0,414412	0,45545	0,700034	no
XLOC_004179	g2262	17,7434	26,2707	0,566171	0,8521	0,1283	0,353906	no
XLOC_004180	g2264	76,4776	149,023	0,96243	1,58943	0,005	0,0410192	yes
XLOC_004181	g2267	28,3018	28,2363	-0,00333986	-0,00570443	0,9919	0,995293	no
XLOC_004182	g2269	75,797	106,281	0,487675	0,757769	0,1752	0,421166	no
XLOC_004183	g2271	25,8423	49,94	0,950463	1,50372	0,00855	0,0597587	no
XLOC_004184	g2273	32,1223	38,8404	0,273985	0,453996	0,42995	0,678111	no
XLOC_004185	g2275	420,873	380,11	-0,146967	-0,20527	0,7213	0,868141	no
XLOC_004186	g2277	158,846	240,822	0,600343	0,909628	0,1147	0,332356	no
XLOC_004187	g2278	109,465	103,232	-0,0845691	-0,136781	0,80965	0,912517	no
XLOC_004188	g2280	58,3321	92,9432	0,672057	1,0209	0,0664	0,234381	no
XLOC_004189	g2281	79,8225	84,3281	0,0792181	0,124922	0,8319	0,922663	no
XLOC_004190	g2283	57,8476	70,4559	0,284464	0,484716	0,3863	0,642307	no
XLOC_004191	g2285	111,231	137,751	0,308509	0,461221	0,4171	0,669268	no
XLOC_004192	g2288	81,5016	95,9276	0,235117	0,368297	0,5105	0,736753	no
XLOC_004193	g2290	26,5927	26,9214	0,0177244	0,02975	0,957	0,980975	no
XLOC_004194	g2292	86,042	110,872	0,365778	0,626645	0,26835	0,529369	no
XLOC_004195	g2295	30,336	94,932	1,64586	1,82976	0,00265	0,0257909	yes
XLOC_004196	g2297	82,7342	132,167	0,675805	1,16343	0,0409	0,172008	no
XLOC_004197	g2299	247,872	360,063	0,538653	0,922001	0,1018	0,307309	no
XLOC_004198	g2301	610,028	396,98	-0,619812	-1,03314	0,0728	0,249343	no
XLOC_004199	g2305	251,959	240,161	-0,0691864	-0,110157	0,84605	0,929972	no
XLOC_004200	g2307	19,7709	16,1057	-0,295804	-0,391516	0,4843	0,718043	no
XLOC_004201	g2309	23,4581	32,6208	0,475706	0,740991	0,1953	0,446852	no
XLOC_004202	g2311	35,9713	31,7083	-0,181984	-0,291334	0,6066	0,800754	no
XLOC_004203	g2312	29,6124	24,2054	-0,29087	-0,466724	0,4072	0,660397	no
XLOC_004204	g2316	1077,53	207,905	-2,37374	-3,09764	5,00E-05	0,00120049	yes
XLOC_004205	g2318	38,0306	37,7908	-0,00912625	-0,0147696	0,9798	0,990233	no
XLOC_004206	g2320	85,183	196,473	1,20569	1,96985	0,00115	0,0138564	yes
XLOC_004207	g2322	105,45	104,913	-0,0073714	-0,0127364	0,982	0,991184	no
XLOC_004208	g2324	109,676	158,507	0,531299	0,865283	0,1261	0,350199	no
XLOC_004209	g2325	1109,2	1910,27	0,784265	1,06427	0,0736	0,250691	no
XLOC_004210	g2329	42,2431	49,3778	0,225148	0,371863	0,5242	0,746323	no
XLOC_004211	g2331	65,6868	115,518	0,814437	1,3536	0,0196	0,107406	no
XLOC_004212	g2333	20,9173	37,5413	0,843786	1,10092	0,05325	0,204904	no
XLOC_004213	g2335	91,8159	59,4826	-0,626276	-0,977075	0,0881	0,280799	no
XLOC_004214	g2338	30,495	34,3781	0,172919	0,231095	0,6747	0,842151	no
XLOC_004215	g2340	212,859	171,622	-0,310662	-0,507239	0,37485	0,633023	no
XLOC_004216	g2342	55,7605	67,246	0,270205	0,404495	0,4748	0,711946	no
XLOC_004217	g2344	8,8263	18,8771	1,09676	1,27094	0,02995	0,141467	no
XLOC_004218	g2346	202,427	121,527	-0,736119	-1,04846	0,0588	0,217237	no
XLOC_004219	g2349	223,662	300,046	0,423864	0,601945	0,2855	0,548008	no
XLOC_004220	g2351	303,566	342,331	0,173381	0,276127	0,63465	0,817196	no
XLOC_004221	g2352	47,5971	53,7098	0,17431	0,288157	0,616	0,807411	no
XLOC_004222	g7756	0	0	0	0	1	1	no
XLOC_004223	g7757	0	0	0	0	1	1	no
XLOC_004224	g7758	12,1886	9,92243	-0,296772	-0,414352	0,4589	0,701036	no
XLOC_004225	g2355	1031,06	1287,44	0,32038	0,492311	0,3809	0,637706	no
XLOC_004226	g2359	13,6007	21,1725	0,638517	0,878449	0,12875	0,354351	no
XLOC_004227	g2354	1533,59	68,2875	-4,48914	-4,82531	5,00E-05	0,00120049	yes
XLOC_004228	g2356	3,70883	2,88957	-0,360106	-0,46369	0,4074	0,660503	no
XLOC_004229	g2357	5,97536	8,92599	0,578987	0,73176	0,2064	0,460752	no
XLOC_004230	g2358	45,7932	99,2577	1,11605	1,76938	0,0024	0,0240735	yes
XLOC_004231	g2360	27,2029	34,4427	0,340441	0,541925	0,3431	0,603374	no

XLOC_004232	g2362	2,07169	1,85093	-0,162558	-0,164599	0,7804	0,898182	no	
XLOC_004233	g2361	12,8013	23,1154	0,85257	1,27001	0,02395	0,123459	no	
XLOC_004234	g2363	4,43294	1,64964	-1,42612	-1,71822	0,0044	0,0374419	yes	
XLOC_004235	g2364	30,7468	44,0657	0,51922	0,776389	0,1771	0,424073	no	
XLOC_004236	g2365	107,6	132,398	0,299204	0,489364	0,38005	0,637068	no	
XLOC_004237	g2366	26,7313	11,0671	-1,27225	-1,61963	0,00635	0,0485646	yes	
XLOC_004238	g7759	4,83319	2,47926	-0,963065	-1,22818	0,0335	0,151669	no	
XLOC_004239	g309	74,4682	68,9134	-0,111841	-0,18482	0,74045	0,876968	no	
XLOC_004240	g311	101,216	83,2395	-0,282089	-0,486296	0,39655	0,650756	no	
XLOC_004241	g312	728,044	995,797	0,451827	0,618673	0,26085	0,521008	no	
XLOC_004242	g314	66,33	60,9199	-0,122747	-0,183095	0,7404	0,876968	no	
XLOC_004243	g315	12,2104	7,32587	-0,73704	-1,0645	0,0629	0,226588	no	
XLOC_004244	g316	63,9536	88,2584	0,464708	0,802737	0,16255	0,404521	no	
XLOC_004245	g310	32,9305	35,8424	0,122241	0,196751	0,73645	0,875431	no	
XLOC_004246	g313	38,6976	31,455	-0,298955	-0,469529	0,3956	0,649604	no	
XLOC_004247	g317	1643,72	2616,32	0,670577	1,00927	0,08085	0,266873	no	
XLOC_004248	g318	17,0314	19,7809	0,215911	0,30419	0,58695	0,787626	no	
XLOC_004249	g319	291,795	664,238	1,18675	1,83897	0,00155	0,0171215	yes	
XLOC_004250	g7760	0	0,0521105	inf	0	1	1	no	
XLOC_004251	g2367	18,6406	12,1075	-0,622546	-0,79783	0,17625	0,422966	no	
XLOC_004252	g2368	49,4099	47,5162	-0,0563814	-0,0887387	0,87515	0,943667	no	
XLOC_004253	g2371	754,291	467,962	-0,688732	-1,12828	0,0512	0,199517	no	
XLOC_004254	g2372	2,40109	4,14979	0,789347	0,668278	0,31235	0,573414	no	
XLOC_004255	g2369	4,60173	5,63282	0,291681	0,396219	0,4991	0,728689	no	
XLOC_004256	g2370	15,5409	10,6511	-0,545072	-0,817114	0,1551	0,392851	no	
XLOC_004257	g7761	0	0	0	0	1	1	no	
XLOC_004258	g7762	0,118897	0,402471	1,75917	0	1	1	no	
XLOC_004259	g7763	22,8553	17,1075	-0,417897	-0,583478	0,30325	0,564534	no	
XLOC_004260	g7764	0	0	0	0	1	1	no	
XLOC_004261	g7765	0	0	0	0	1	1	no	
XLOC_004262	g7766	65,8672	86,0884	0,386257	0,57981	0,3111	0,572083	no	
XLOC_004263	g7767	0	0	0	0	1	1	no	
XLOC_004264	g7769	7,95112	3,2762	-1,27914	-1,46362	0,01745	0,0982563	no	
XLOC_004265	g7768	7,80778	3,12261	-1,32216	-1,46791	0,03655	0,160304	no	
XLOC_004266	g7771	47,8224	97,4154	1,02646	1,72245	0,0046	0,0386416	yes	
XLOC_004267	g7770	11,2869	27,8122	1,30107	1,8138	0,00225	0,0229201	yes	
XLOC_004268	g7772	24,8106	66,5974	1,42451	1,97268	0,00145	0,0164423	yes	
XLOC_004269	g7773	230,694	277,6	0,267024	0,357316	0,54515	0,761892	no	
XLOC_004270	g7774	187,496	209,872	0,16265	0,264369	0,64415	0,824348	no	
XLOC_004271	g7775	259,193	617,863	1,25326	1,68249	0,00675	0,0506379	no	
XLOC_004272	g7777	4,01149	1,52694	-1,39349	-1,38992	0,0229	0,119743	no	
XLOC_004273	g7776	1067,3	648,259	-0,719322	-0,830587	0,13055	0,357801	no	
XLOC_004274	g7778	214,963	138,843	-0,630637	-1,08457	0,05515	0,209102	no	
XLOC_004275	g7779	0	0	0	0	1	1	no	
XLOC_004276	g7780	19,2237	24,9867	0,378274	0,532828	0,35885	0,619938	no	
XLOC_004277	g7781	116,388	60,4845	-0,94431	-1,45611	0,00965	0,0647288	no	
XLOC_004278	g321	97,5526	209,969	1,10592	1,54673	0,00925	0,0629487	no	
XLOC_004279	g322	130,795	215,16	0,718103	1,18354	0,03885	0,166524	no	
XLOC_004280	g323	132,73	42,2737	-1,65067	-2,19077	0,00045	0,00669545	yes	
XLOC_004281	g325	110,114	140,998	0,356687	0,542227	0,3483	0,608812	no	
XLOC_004282	g327	20,6396	19,116	-0,110634	-0,150893	0,79605	0,906216	no	
XLOC_004283	g329	43,4859	32,1591	-0,435321	-0,745358	0,1851	0,433504	no	
XLOC_004284	g332	56,9594	56,8329	-0,00320577	-0,0055138	0,99205	0,995293	no	
XLOC_004285	g320	24,2968	31,5387	0,376358	0,371821	0,5168	0,741989	no	
XLOC_004286	g324	42,9929	250,562	2,54299	3,2147	5,00E-05	0,00120049	yes	
XLOC_004287	g326	61,21	61,0411	-0,00398487	-0,00673519	0,9901	0,99501	no	
XLOC_004288	g328	13,4762	14,6025	0,115803	0,175736	0,74685	0,880922	no	
XLOC_004289	g330	13,9935	22,5749	0,689955	1,06593	0,06405	0,229635	no	
XLOC_004290	g331	23,6145	30,973	0,391338	0,61527	0,29075	0,553529	no	
XLOC_004291	g333	882,327	291,639	-1,59713	-1,64671	0,0042	0,0360524	yes	
XLOC_004292	g7782	30,9127	39,9697	0,370708	0,491585	0,393	0,647853	no	
XLOC_004293	g7784	0	0	0	0	1	1	no	
XLOC_004294	g7783	0,0144477	0	#NAME?	0	1	1	no	
XLOC_004295	g7785	0	0,115734	inf	0	1	1	no	
XLOC_004296	g7786	147,669	99,1927	-0,574066	-0,942981	0,09075	0,286154	no	
XLOC_004297	g7787	2,85904	2,92906	0,0349071	0,0278506	0,9189	0,964163	no	
XLOC_004298	g7788	53,8214	40,8571	-0,397591	-0,666283	0,2359	0,493721	no	
XLOC_004299	g7789	0,64421	3,42129	2,40894	1,99871	0,0079	0,0564204	no	
XLOC_004300	g7790	14,7113	142,168	3,2726	3,87292	5,00E-05	0,00120049	yes	
XLOC_004301	g7792	35,9623	20,1046	-0,838962	-1,355	0,01885	0,10411	no	
XLOC_004302	g7793	98,4287	77,9834	-0,335911	-0,582698	0,315	0,57568	no	
XLOC_004303	g7794	86,4656	93,1953	0,108131	0,168916	0,7621	0,887899	no	
XLOC_004304	g7791	11,3707	14,4758	0,348323	0,509303	0,37115	0,629674	no	
XLOC_004305	g7795	28,2249	15,8827	-0,829514	-1,19494	0,035	0,156369	no	
XLOC_004306	g7796	0	0	0	0	1	1	no	
XLOC_004307	g7797	0	0	0	0	1	1	no	
XLOC_004308	g7798	0	0	0	0	1	1	no	
XLOC_004309	g2374	133,096	58,6747	-1,18166	-1,98749	0,0004	0,0061761	yes	
XLOC_004310	g2376	91,6368	30,4124	-1,59127	-2,438	0,0001	0,00209829	yes	
XLOC_004311	g2377	11,3414	40,6212	1,84064	2,46408	5,00E-05	0,00120049	yes	
XLOC_004312	g2379	1,93414	0,914161	-1,08117	0	1	1	no	
XLOC_004313	g2373	9,8955	9,61752	-0,0411073	-0,0508073	0,9268	0,967593	no	
XLOC_004314	g2375	2590,02	1281,79	-1,0148	-1,34327	0,01895	0,104544	no	
XLOC_004315	g2378	20,9919	12,7091	-0,723965	-1,03002	0,07395	0,251276	no	
XLOC_004316	g7799	7,07608	6,2512	-0,178818	-0,198922	0,73775	0,876234	no	
XLOC_004317	g2380	2,02442	0,674782	-1,58502	-1,20139	0,08045	0,26591	no	
XLOC_004318	g7800	0	0	0	0	1	1	no	
XLOC_004319	g7801	0	0	0	0	1	1	no	
XLOC_004320	g2381	0,111557	0,342407	1,61793	0	1	1	no	
XLOC_004321	g7802	88,5921	92,1786	0,0572526	0,0753891	0,89665	0,954069	no	
XLOC_004322	g7803	8,56163	1,63284	-2,3905	-1,37774	0,17195	0,416926	no	
XLOC_004323	g7804	9,27881	8,06835	-0,201666	-0,217076	0,7075	0,86135	no	

XLOC_004324	g7805	5,99208	7,9723	0,411938	0,575113	0,28985	0,552768	no
XLOC_004325	g2382	39,6106	45,7654	0,208373	0,332366	0,54495	0,761892	no
XLOC_004326	g2384	172,644	169,882	-0,0232639	-0,036836	0,94735	0,976281	no
XLOC_004327	g2386	105,994	477,76	2,1723	2,98979	5,00E-05	0,00120049	yes
XLOC_004328	g2387	18,0706	33,7779	0,90243	1,38503	0,02255	0,118481	no
XLOC_004329	g2388	91,7604	123,503	0,428602	0,683369	0,229	0,487276	no
XLOC_004330	g2390	29,9871	39,7593	0,406949	0,628326	0,26795	0,528997	no
XLOC_004331	g2392	19,6946	18,0307	-0,127342	-0,184378	0,746	0,880495	no
XLOC_004332	g2393	95,1171	44,8583	-1,08433	-1,64398	0,0042	0,0360524	yes
XLOC_004333	g2394	22,1319	8,47857	-1,38423	-1,95889	0,0009	0,0115984	yes
XLOC_004334	g2396	20,6884	21,2947	0,0416754	0,0605002	0,91765	0,964054	no
XLOC_004335	g2397	8,0498	9,9141	0,300528	0,444251	0,44515	0,6915	no
XLOC_004336	g2383	15,9374	12,6315	-0,335386	-0,354458	0,5427	0,76057	no
XLOC_004337	g2385	38,1075	39,1197	0,0378192	0,0612814	0,9115	0,962529	no
XLOC_004338	g2389	19,5826	12,0426	-0,701418	-0,9719	0,08925	0,283062	no
XLOC_004339	g2391	93,4028	96,3259	0,0444581	0,0750164	0,89515	0,953456	no
XLOC_004340	g2395	34,0338	30,8308	-0,142595	-0,245649	0,66425	0,83565	no
XLOC_004341	g7806	44,9891	63,1724	0,489718	0,725139	0,21595	0,471985	no
XLOC_004342	g7807	0	0	0	0	1	1	no
XLOC_004343	g7808	77,5467	6,56282	-3,56268	-2,72291	0,0038	0,0337091	yes
XLOC_004344	g7809	0,124923	0	#NAME?	0	1	1	no
XLOC_004345	g7810	3,27189	7,22892	1,14365	0,915228	0,1191	0,3392	no
XLOC_004346	g7811	12,6632	15,4375	0,285791	0,322643	0,56575	0,774849	no
XLOC_004347	g7812	11,4967	12,0115	0,0631947	0,0731506	0,8954	0,953462	no
XLOC_004348	g7813	0	0	0	0	1	1	no
XLOC_004349	g7814	8,11326	7,41071	-0,130669	-0,164078	0,7735	0,894251	no
XLOC_004350	g7815	2,7699	0,691592	-2,00184	-1,53317	0,0429	0,177679	no
XLOC_004351	g7816	61,3671	30,1798	-1,02388	-1,56744	0,0051	0,0415963	yes
XLOC_004352	g7817	0	0	0	0	1	1	no
XLOC_004353	g2399	61,4103	61,9156	0,0118216	0,0183991	0,9735	0,98758	no
XLOC_004354	g2402	37,6628	35,4571	-0,0870685	-0,148461	0,7926	0,904567	no
XLOC_004355	g2403	51,1116	63,8766	0,321636	0,52392	0,35275	0,613315	no
XLOC_004356	g2405	58,8389	105,897	0,847817	1,39835	0,0162	0,0931405	no
XLOC_004357	g2398	135,794	151,453	0,157458	0,212605	0,7147	0,865069	no
XLOC_004358	g2400	33,0588	50,0208	0,597492	0,922527	0,1	0,303086	no
XLOC_004359	g2401	10,6487	13,6131	0,354323	0,461641	0,41675	0,668925	no
XLOC_004360	g2404	33,8341	39,9405	0,239371	0,342446	0,5555	0,768636	no
XLOC_004361	g7818	0	0	0	0	1	1	no
XLOC_004362	g7819	0	0	0	0	1	1	no
XLOC_004363	g7820	0	0	0	0	1	1	no
XLOC_004364	g7821	35,2063	25,6541	-0,456643	-0,742532	0,187	0,435358	no
XLOC_004365	g2406	8,1093	15,6079	0,944624	1,33016	0,02515	0,127109	no
XLOC_004366	g2407	105,335	17,5567	-2,58489	-3,26123	5,00E-05	0,00120049	yes
XLOC_004367	g2410	268,24	206,841	-0,375004	-0,608821	0,2608	0,521008	no
XLOC_004368	g2411	48,4511	41,5048	-0,22325	-0,349696	0,5391	0,758039	no
XLOC_004369	g2414	56,6487	38,1582	-0,570049	-0,977374	0,08545	0,276208	no
XLOC_004370	g2416	13,0333	9,71271	-0,424258	-0,711473	0,21725	0,473982	no
XLOC_004371	g2408	352,221	260,48	-0,435308	-0,674294	0,2374	0,495382	no
XLOC_004372	g2409	29,4624	27,4309	-0,103074	-0,148385	0,79275	0,904567	no
XLOC_004373	g2412	30,2257	29,7555	-0,022623	-0,0388164	0,94635	0,975967	no
XLOC_004374	g2413	6,55966	9,15101	0,48031	0,520405	0,37945	0,636522	no
XLOC_004375	g2415	149,753	50,9475	-1,5555	-2,58655	5,00E-05	0,00120049	yes
XLOC_004376	g7822	3,12632	5,09484	0,704574	0,798746	0,18245	0,42989	no
XLOC_004377	g7823	36,7079	29,398	-0,320372	-0,481675	0,39975	0,653604	no
XLOC_004378	g7824	232,368	142,347	-0,707004	-0,976776	0,083	0,271381	no
XLOC_004379	g7825	0	0	0	0	1	1	no
XLOC_004380	g7826	34,508	50,0157	0,53545	0,821102	0,1399	0,37332	no
XLOC_004381	g7827	60,3684	33,5136	-0,849047	-1,32451	0,02235	0,117872	no
XLOC_004382	g7829	17,6208	24,5021	0,47562	0,742689	0,17695	0,42392	no
XLOC_004383	g7828	8,15713	13,1201	0,685644	0,986377	0,08	0,26469	no
XLOC_004384	g7830	36,4223	45,5672	0,323173	0,468204	0,40365	0,657326	no
XLOC_004385	g7833	76,4185	136,794	0,840009	1,22542	0,0335	0,151669	no
XLOC_004386	g7831	806,774	2719,99	1,75337	2,5452	0,0001	0,00209829	yes
XLOC_004387	g7832	18,1093	21,9444	0,277119	0,469558	0,403	0,656594	no
XLOC_004388	g7834	11,3187	14,6537	0,372546	0,627869	0,27275	0,53424	no
XLOC_004389	g7835	0	0	0	0	1	1	no
XLOC_004390	g7836	77,5376	95,8135	0,305333	0,477669	0,3947	0,648995	no
XLOC_004391	g2417	28,6219	30,6464	0,0985982	0,164829	0,77055	0,893264	no
XLOC_004392	g2418	12,3299	11,4646	-0,104973	-0,150152	0,78775	0,902018	no
XLOC_004393	g7837	601,135	1255,4	1,06239	1,42089	0,02295	0,119813	no
XLOC_004394	g7838	7,32856	3,29829	-1,15181	-1,5067	0,01185	0,075124	no
XLOC_004395	g7839	0	0	0	0	1	1	no
XLOC_004396	g7840	0	0	0	0	1	1	no
XLOC_004397	g7841	3,98492	5,2433	0,395925	0,461943	0,4385	0,684886	no
XLOC_004398	g7842	13,9425	20,2019	0,534995	0,747119	0,1917	0,442199	no
XLOC_004399	g7844	0,199677	1,60545	3,00723	1,71277	0,02995	0,141467	no
XLOC_004400	g7843	7,77099	6,73066	-0,20735	-0,280255	0,62235	0,810433	no
XLOC_004401	g7846	49,4537	42,2924	-0,225682	-0,380346	0,5142	0,740605	no
XLOC_004402	g7845	97,1124	75,4004	-0,365084	-0,510168	0,3598	0,620301	no
XLOC_004403	g7847	33,9289	25,6964	-0,400947	-0,579492	0,3105	0,571193	no
XLOC_004404	g7848	0,838423	0,83898	0,000958116	0	1	1	no
XLOC_004405	g7849	0	0	0	0	1	1	no
XLOC_004406	g7850	37,2292	55,1648	0,567312	0,85442	0,13255	0,361969	no
XLOC_004407	g2419	49,1729	95,9438	0,964326	1,34124	0,0216	0,115278	no
XLOC_004408	g2420	3,33786	3,17659	-0,0714438	-0,0720488	0,9049	0,958435	no
XLOC_004409	g7851	0,739159	0,255767	-1,53106	0	1	1	no
XLOC_004410	g7852	0	3,26568	inf	0	1	1	no
XLOC_004411	g7853	4,70268	4,92723	0,0672925	0,0812924	0,8881	0,950222	no
XLOC_004412	g7854	8,74974	14,2485	0,703492	0,977079	0,093	0,290512	no
XLOC_004413	g334	0	0	0	0	1	1	no
XLOC_004414	g7855	0	0,40735	inf	0	1	1	no
XLOC_004415	g7856	0	0	0	0	1	1	no

XLOC_004416	g7857	22,0325	24,8299	0,172444	0,26525	0,65035	0,828117	no
XLOC_004417	g7858	12,6469	22,3104	0,818932	1,20271	0,037	0,161772	no
XLOC_004418	g7860	28,4902	33,1608	0,219009	0,378029	0,5144	0,740676	no
XLOC_004419	g7859	11,175	23,013	1,04217	0,821629	0,1476	0,384261	no
XLOC_004420	g7862	0	0,0309072	inf	0	1	1	no
XLOC_004421	g7861	0	0	0	0	1	1	no
XLOC_004422	g7863	0	0	0	0	1	1	no
XLOC_004423	g7864	0,0746436	0	#NAME?	0	1	1	no
XLOC_004424	g7865	22,9485	32,363	0,495943	0,809095	0,15335	0,391345	no
XLOC_004425	g7866	25,5442	29,7576	0,220262	0,362445	0,5269	0,748684	no
XLOC_004426	g7867	119,999	81,3189	-0,561359	-0,745694	0,20285	0,45552	no
XLOC_004427	g7868	44,8115	31,4976	-0,508627	-0,842215	0,1481	0,384848	no
XLOC_004428	g2421	0,127434	0,201952	0,664263	0	1	1	no
XLOC_004429	g7869	13,1633	11,4248	-0,204346	-0,208527	0,72465	0,869722	no
XLOC_004430	g7870	0	0	0	0	1	1	no
XLOC_004431	g7871	31,6478	30,2081	-0,0671679	-0,106829	0,85115	0,933158	no
XLOC_004432	g7872	49,9001	50,0804	0,00520562	0,00901325	0,9867	0,993275	no
XLOC_004433	g2422	4,18359	6,85507	0,712432	1,05638	0,07335	0,250274	no
XLOC_004434	g7873	2,10376	2,80941	0,417295	0,399359	0,47775	0,713645	no
XLOC_004435	g7874	4,26547	3,73955	-0,189842	-0,162678	0,7777	0,895837	no
XLOC_004436	g7876	109,287	112,316	0,0394441	0,0627413	0,9131	0,963016	no
XLOC_004437	g7877	41,4753	48,9203	0,238183	0,380634	0,52145	0,745145	no
XLOC_004438	g7883	15,5456	15,0623	-0,0455558	-0,0701653	0,9016	0,956954	no
XLOC_004439	g7884	200,526	242,491	0,274141	0,46738	0,41555	0,667815	no
XLOC_004440	g7885	50,3403	31,2114	-0,689641	-1,15086	0,0464	0,186818	no
XLOC_004441	g7886	52,0596	78,1224	0,585571	0,794618	0,1586	0,398835	no
XLOC_004442	g7875	26,9858	38,4742	0,511694	0,69451	0,2328	0,490579	no
XLOC_004443	g7878	110,795	85,6963	-0,370583	-0,623118	0,27825	0,53979	no
XLOC_004444	g7879	55,2029	44,7426	-0,303092	-0,471092	0,4075	0,660523	no
XLOC_004445	g7880	215,508	246,149	0,191791	0,298525	0,6053	0,799791	no
XLOC_004446	g7881	313,032	456,193	0,543333	0,908466	0,09695	0,297981	no
XLOC_004447	g7882	198,369	228,653	0,20497	0,34914	0,5415	0,759787	no
XLOC_004448	g7887	80,7014	94,1979	0,223101	0,381338	0,5002	0,729752	no
XLOC_004449	g7889	65,6975	27,7368	-1,24404	-2,06362	0,00045	0,00669545	yes
XLOC_004450	g7892	59,3336	65,6811	0,146628	0,235879	0,66895	0,838813	no
XLOC_004451	g7888	91,0602	36,9685	-1,30052	-1,82105	0,00205	0,0212577	yes
XLOC_004452	g7890	16,0152	12,6311	-0,342461	-0,485841	0,3912	0,646031	no
XLOC_004453	g7891	37,7708	40,2096	0,0902667	0,133396	0,8184	0,917235	no
XLOC_004454	g7893	37,8608	31,1471	-0,281608	-0,416444	0,47055	0,708386	no
XLOC_004455	g7894	0,2762	0,227307	-0,281069	0	1	1	no
XLOC_004456	g2423	4,03676	8,76887	1,11919	1,40298	0,0196	0,107406	no
XLOC_004457	g2424	1,27603	0,141661	-3,17115	-2,21104	0,00865	0,0602433	no
XLOC_004458	g2425	0,0970444	1,11367	3,52053	0	1	1	no
XLOC_004459	g7895	0	0	0	0	1	1	no
XLOC_004460	g7896	40,993	35,583	-0,204188	-0,330428	0,55405	0,767387	no
XLOC_004461	g7898	111,173	125,636	0,176445	0,281024	0,62525	0,812208	no
XLOC_004462	g7901	53,665	89,5486	0,73869	1,22419	0,0287	0,137614	no
XLOC_004463	g7905	210,897	129,894	-0,699205	-0,951437	0,0893	0,283062	no
XLOC_004464	g7906	264,796	238,71	-0,149622	-0,252428	0,6526	0,829583	no
XLOC_004465	g7908	286,642	103,949	-1,46337	-2,29757	0,0001	0,00209829	yes
XLOC_004466	g7897	32,8383	20,0603	-0,711035	-1,08097	0,05795	0,215475	no
XLOC_004467	g7899	41,118	39,3572	-0,0631434	-0,107245	0,84835	0,931286	no
XLOC_004468	g7900	58,0813	62,5761	0,107539	0,181044	0,7454	0,880058	no
XLOC_004469	g7902	47,3461	86,9907	0,877616	1,49746	0,01125	0,0722531	no
XLOC_004470	g7903	133,537	159,452	0,255882	0,397033	0,49195	0,723707	no
XLOC_004471	g7904	2,58347	8,26125	1,67705	1,58095	0,02485	0,125917	no
XLOC_004472	g7907	21,4184	28,5562	0,414949	0,656615	0,2608	0,521008	no
XLOC_004473	g7909	72,257	66,7072	-0,115296	-0,188853	0,7339	0,874259	no
XLOC_004474	g7910	0	0	0	0	1	1	no
XLOC_004475	g7911	21,0756	11,6279	-0,85799	-1,0133	0,08165	0,268611	no
XLOC_004476	g7912	97,511	63,8487	-0,610908	-1,00575	0,08115	0,267593	no
XLOC_004477	g7913	61,2325	66,7455	0,124373	0,213759	0,7028	0,859051	no
XLOC_004478	g7914	2,18657	2,38681	0,126416	0,117325	0,8339	0,924151	no
XLOC_004479	g7915	62,5113	43,8558	-0,51135	-0,87593	0,1172	0,335541	no
XLOC_004480	g335	3,58308	0,985896	-1,86169	-1,73224	0,02105	0,113019	no
XLOC_004481	g336	52,4017	47,4333	-0,143711	-0,247821	0,67955	0,845777	no
XLOC_004482	g338	105,117	110,427	0,0710969	0,120017	0,8339	0,924151	no
XLOC_004483	g337	48,19	25,4846	-0,919105	-1,505	0,00915	0,0624413	no
XLOC_004484	g339	18,151	16,7483	-0,116034	-0,148426	0,79635	0,906216	no
XLOC_004485	g7916	91,8068	103,204	0,168832	0,284874	0,627	0,813038	no
XLOC_004486	g7917	465,323	344,358	-0,434324	-0,74197	0,188	0,436961	no
XLOC_004487	g7918	154,491	186,596	0,272395	0,442919	0,44055	0,686808	no
XLOC_004488	g2428	18,1365	20,6743	0,188945	0,300518	0,58985	0,790006	no
XLOC_004489	g2430	45,2369	46,5154	0,040208	0,0682583	0,9049	0,958435	no
XLOC_004490	g2432	23,8593	20,3708	-0,228052	-0,37461	0,50445	0,732471	no
XLOC_004491	g2426	40,4001	51,4769	0,349566	0,600996	0,30365	0,565064	no
XLOC_004492	g2427	38,4975	30,3373	-0,343671	-0,559712	0,32015	0,580479	no
XLOC_004493	g2429	55,6207	70,4479	0,340935	0,58686	0,3062	0,567308	no
XLOC_004494	g2431	116,651	103,642	-0,170583	-0,2876	0,61275	0,805258	no
XLOC_004495	g2433	0	0	0	0	1	1	no
XLOC_004496	g7919	98,4927	137,605	0,48245	0,739546	0,18415	0,43231	no
XLOC_004497	g7920	50,9732	31,7537	-0,682813	-1,05038	0,0641	0,229647	no
XLOC_004498	g7921	3,56719	0,79148	-2,17216	-1,63936	0,13325	0,362872	no
XLOC_004499	g7922	0	0	0	0	1	1	no
XLOC_004500	g7923	63,2025	72,3488	0,194987	0,304777	0,579	0,782263	no
XLOC_004501	g7924	0	0	0	0	1	1	no
XLOC_004502	g340	0,0976908	1,02051	3,38493	0	1	1	no
XLOC_004503	g7925	0	0	0	0	1	1	no
XLOC_004504	g7926	46,6632	29,9138	-0,641474	-1,00488	0,0818	0,268744	no
XLOC_004505	g7927	38,6331	37,3554	-0,0485224	-0,0716807	0,9055	0,958604	no
XLOC_004506	g2434	59,2887	31,0744	-0,932032	-1,50417	0,00935	0,0635412	no
XLOC_004507	g7928	0	0	0	0	1	1	no

XLOC_004508	g7929	0	0	0	0	1	1	no
XLOC_004509	g7930	271,604	393,14	0,533537	0,680228	0,2427	0,501012	no
XLOC_004510	g7932	1,04007	1,17262	0,173062	0	1	1	no
XLOC_004511	g7931	32,6978	22,3966	-0,545917	-0,891454	0,11595	0,333955	no
XLOC_004512	g7933	0	0	0	0	1	1	no
XLOC_004513	g7935	29,642	11,1681	-1,40826	-1,35225	0,0297	0,140964	no
XLOC_004514	g7936	4,97373	1,84417	-1,43135	-1,45248	0,02075	0,111959	no
XLOC_004515	g7938	64,3407	82,9852	0,367123	0,585482	0,3127	0,573414	no
XLOC_004516	g7934	59,1522	59,0573	-0,00231582	-0,00303105	0,99635	0,997874	no
XLOC_004517	g7937	46,0736	19,5488	-1,23686	-1,56777	0,0087	0,0603347	no
XLOC_004518	g7939	22,4528	18,7797	-0,257724	-0,372189	0,50875	0,735127	no
XLOC_004519	g7940	80,6588	56,5715	-0,511757	-0,834585	0,1506	0,387855	no
XLOC_004520	g7941	0	0	0	0	1	1	no
XLOC_004521	g7944	17,7188	10,0083	-0,824082	-1,14808	0,03875	0,166386	no
XLOC_004522	g7942	145,544	170,185	0,225643	0,307961	0,56055	0,771601	no
XLOC_004523	g7943	56,2759	35,3935	-0,669035	-1,10393	0,0583	0,216122	no
XLOC_004524	g7945	508,351	449,757	-0,176679	-0,256308	0,6568	0,8318	no
XLOC_004525	g7946	21,642	22,1432	0,0330307	0,0512336	0,92565	0,967112	no
XLOC_004526	g7947	0,0736974	0	#NAME?	0	1	1	no
XLOC_004527	g7948	1,34985	1,12727	-0,259969	-0,258406	0,6449	0,824921	no
XLOC_004528	g2435	7,23028	1,61032	-2,16671	-2,39587	0,00035	0,00552572	yes
XLOC_004529	g2436	2,51742	0,775538	-1,69868	-1,64028	0,01955	0,107252	no
XLOC_004530	g7949	34,1515	31,7178	-0,106657	-0,159834	0,7831	0,899946	no
XLOC_004531	g2437	0	0	0	0	1	1	no
XLOC_004532	g7950	18,7853	31,9124	0,764516	0,863079	0,15105	0,388505	no
XLOC_004533	g2438	0,62773	1,01732	0,69656	0	1	1	no
XLOC_004534	g7951	367,331	179,012	-1,03702	-1,66605	0,0036	0,0323144	yes
XLOC_004535	g7952	86,3213	220,935	1,35583	1,91507	0,00235	0,0236687	yes
XLOC_004536	g2440	21,2551	10,8716	-0,967244	-1,45789	0,01005	0,0667282	no
XLOC_004537	g2441	236,375	407,917	0,787197	1,21075	0,0319	0,146794	no
XLOC_004538	g2442	100,602	101,318	0,010222	0,0154831	0,97845	0,989941	no
XLOC_004539	g2443	23,8096	31,6456	0,41046	0,669838	0,2303	0,487612	no
XLOC_004540	g2445	150,136	22,0523	-2,76727	-2,94683	0,00015	0,00294012	yes
XLOC_004541	g2446	26,1017	18,0848	-0,529366	-0,859836	0,12495	0,348384	no
XLOC_004542	g2439	17,51	15,4713	-0,178583	-0,289828	0,60045	0,796813	no
XLOC_004543	g2444	47,8932	52,8112	0,141023	0,227647	0,6881	0,850292	no
XLOC_004544	g2447	29,4544	26,7998	-0,136265	-0,230092	0,68835	0,850371	no
XLOC_004545	g7953	64,2956	49,0418	-0,390707	-0,648006	0,24825	0,507023	no
XLOC_004546	g7954	133,783	88,3085	-0,599272	-0,920974	0,09815	0,300447	no
XLOC_004547	g7955	8,34917	12,5583	0,588941	0,853118	0,1325	0,361933	no
XLOC_004548	g7956	2,08886	8,21811	1,97609	1,56659	0,0211	0,113225	no
XLOC_004549	g7957	9,4247	91,2132	3,27473	4,06476	5,00E-05	0,00120049	yes
XLOC_004550	g2448	0,245034	0,428335	0,805758	0	1	1	no
XLOC_004551	g7958	0	0	0	0	1	1	no
XLOC_004552	g7959	19,8441	27,068	0,447876	0,672782	0,24115	0,499282	no
XLOC_004553	g7960	0	0	0	0	1	1	no
XLOC_004554	g2449	39,9888	28,4585	-0,490738	-0,71283	0,20225	0,454692	no
XLOC_004555	g7961	0	0	0	0	1	1	no
XLOC_004556	g7962	0	0	0	0	1	1	no
XLOC_004557	g7963	17,8678	36,9994	1,05014	1,35124	0,01915	0,10547	no
XLOC_004558	g7964	17,7424	23,1449	0,383493	0,346506	0,54975	0,765215	no
XLOC_004559	g7965	14,42	24,2886	0,75221	0,814685	0,16025	0,401443	no
XLOC_004560	g7966	0,192414	0,21603	0,167018	0	1	1	no
XLOC_004561	g7967	136,264	123,04	-0,147273	-0,220154	0,70425	0,859387	no
XLOC_004562	g2450	24,6878	38,4176	0,637971	0,494054	0,37475	0,633023	no
XLOC_004563	g2451	13,2818	14,1599	0,0923656	0,0996238	0,86735	0,939446	no
XLOC_004564	g2452	0	0	0	0	1	1	no
XLOC_004565	g2453	0	0	0	0	1	1	no
XLOC_004566	g7968	0	0	0	0	1	1	no
XLOC_004567	g7969	511,512	430,314	-0,249379	-0,337273	0,5492	0,765215	no
XLOC_004568	g2456	0	0	0	0	1	1	no
XLOC_004569	g2454	0	0	0	0	1	1	no
XLOC_004570	g2455	0	0	0	0	1	1	no
XLOC_004571	g2457	0	0	0	0	1	1	no
XLOC_004572	g7970	0	0	0	0	1	1	no
XLOC_004573	g7971	13,5628	25,1523	0,891031	1,42548	0,0115	0,0733788	no
XLOC_004574	g7972	1,53539	1,60744	0,0661602	0,0630713	0,91185	0,962529	no
XLOC_004575	g7973	86,9419	56,7454	-0,615549	-0,984113	0,08385	0,273013	no
XLOC_004576	g7974	45,793	48,2226	0,0745795	0,0996449	0,8542	0,934519	no
XLOC_004577	g7975	68,3737	84,2715	0,301603	0,347581	0,56155	0,77218	no
XLOC_004578	g7976	43,8667	169,229	1,94778	2,99109	5,00E-05	0,00120049	yes
XLOC_004579	g7977	82,6903	37,5789	-1,1378	-1,8138	0,002	0,0208936	yes
XLOC_004580	g7979	10,8304	4,673	-1,21266	-1,49882	0,0111	0,0715706	no
XLOC_004581	g7978	9,58299	8,3889	-0,191995	-0,216581	0,687	0,849665	no
XLOC_004582	g2459	42,1492	53,6238	0,347368	0,51067	0,34595	0,606215	no
XLOC_004583	g2461	12,2332	20,0767	0,714712	0,982575	0,07435	0,252007	no
XLOC_004584	g2462	13,9387	46,4743	1,73733	2,49391	5,00E-05	0,00120049	yes
XLOC_004585	g2465	44,4523	56,6858	0,350732	0,52697	0,36045	0,620877	no
XLOC_004586	g2458	27,7875	15,4854	-0,843526	-1,03588	0,0622	0,224891	no
XLOC_004587	g2460	35,987	52,5641	0,546604	0,90648	0,10775	0,319291	no
XLOC_004588	g2463	11,1003	32,5287	1,55111	2,14415	0,00045	0,00669545	yes
XLOC_004589	g2464	39,566	41,3991	0,0653377	0,100559	0,8632	0,936801	no
XLOC_004590	g2466	18,1956	10,895	-0,739918	-1,10362	0,05315	0,20476	no
XLOC_004591	g7980	0	0	0	0	1	1	no
XLOC_004592	g7981	0	0	0	0	1	1	no
XLOC_004593	g7984	38,5656	36,592	-0,0757847	-0,129628	0,8184	0,917235	no
XLOC_004594	g7986	16,6181	7,50446	-1,14693	-1,53914	0,00825	0,0582842	no
XLOC_004595	g7982	9,40976	7,54095	-0,319412	-0,332275	0,55795	0,77025	no
XLOC_004596	g7983	116,024	113,617	-0,0302417	-0,0524564	0,9272	0,967857	no
XLOC_004597	g7985	33,2741	79,8818	1,26347	2,07576	0,0004	0,0061761	yes
XLOC_004598	g7987	126,404	82,7857	-0,610584	-0,880728	0,12725	0,351999	no
XLOC_004599	g7988	0	0	0	0	1	1	no

XLOC_004600	g7989	16,7136	23,6403	0,500224	0,699063	0,2227	0,480113	no
XLOC_004601	g7991	28,6736	35,5235	0,309048	0,499674	0,3825	0,639018	no
XLOC_004602	g7990	157,261	109,55	-0,521575	-0,83308	0,1465	0,38343	no
XLOC_004603	g7992	42,9756	51,975	0,274299	0,469069	0,40325	0,656811	no
XLOC_004604	g2467	2,74222	0	#NAME?	0	1	1	no
XLOC_004605	g2468	1980,97	656,793	-1,59269	-1,47489	0,0112	0,0720262	no
XLOC_004606	g7993	168,457	118,118	-0,512154	-0,852842	0,14255	0,377723	no
XLOC_004607	g7994	212,514	149,79	-0,504616	-0,769479	0,1748	0,420617	no
XLOC_004608	g7995	50,5888	32,2484	-0,64959	-1,09721	0,0584	0,216329	no
XLOC_004609	g7996	21,9259	20,7622	-0,0786715	-0,134512	0,81695	0,916851	no
XLOC_004610	g7997	56,1266	30,813	-0,865146	-1,33333	0,0174	0,0980873	no
XLOC_004611	g7999	72,4945	48,42	-0,582269	-0,960007	0,09495	0,294414	no
XLOC_004612	g8001	0,547192	1,39945	1,35474	1,04218	0,07415	0,251608	no
XLOC_004613	g8002	4,0642	3,87794	-0,0676819	-0,101098	0,8586	0,935373	no
XLOC_004614	g7998	69,9813	97,8834	0,484096	0,826324	0,14375	0,379469	no
XLOC_004615	g8000	3,40582	39,7935	3,54646	4,36927	5,00E-05	0,00120049	yes
XLOC_004616	g8003	26,2547	22,5363	-0,220326	-0,330554	0,56245	0,772769	no
XLOC_004617	g8004	0	0	0	0	1	1	no
XLOC_004618	g2470	0	0	0	0	1	1	no
XLOC_004619	g2469	0	0	0	0	1	1	no
XLOC_004620	g8005	106,356	97,9591	-0,11865	-0,205513	0,72185	0,868484	no
XLOC_004621	g8006	77,6214	120,523	0,634784	1,06104	0,0583	0,216122	no
XLOC_004622	g8007	8,71503	8,93335	0,0356965	0,0492896	0,9342	0,971293	no
XLOC_004623	g8008	109,1	119,578	0,132301	0,221489	0,7064	0,860758	no
XLOC_004624	g8009	224,948	512,116	1,18688	1,8467	0,0013	0,0151795	yes
XLOC_004625	g2471	4,9148	6,38308	0,377119	0,48027	0,39805	0,652209	no
XLOC_004626	g2472	244,37	93,3203	-1,38881	-2,01643	0,0006	0,00845337	yes
XLOC_004627	g2473	2,89111	7,52646	1,38035	0,896476	0,1336	0,363462	no
XLOC_004628	g8010	25,2675	38,21	0,596666	0,94347	0,10715	0,31837	no
XLOC_004629	g8011	353,292	3108,29	3,13719	3,27854	5,00E-05	0,00120049	yes
XLOC_004630	g2476	6,69274	6,12647	-0,12754	-0,10983	0,84425	0,929224	no
XLOC_004631	g2474	13,3125	12,1113	-0,136425	-0,183139	0,7571	0,8854	no
XLOC_004632	g2475	188,196	187,361	-0,00641656	-0,00999493	0,9844	0,992379	no
XLOC_004633	g2477	395,211	186,079	-1,08671	-1,61241	0,0043	0,0368143	yes
XLOC_004634	g341	220,289	582,435	1,4027	2,07477	0,00035	0,00552572	yes
XLOC_004635	g342	33,7219	34,3828	0,0280018	0,0481902	0,9318	0,970131	no
XLOC_004636	g344	146,944	161,791	0,138863	0,225804	0,6857	0,849081	no
XLOC_004637	g345	96,2313	117,79	0,291643	0,388353	0,49305	0,724054	no
XLOC_004638	g347	168,012	246,55	0,553316	0,863525	0,1371	0,369327	no
XLOC_004639	g343	27,7559	17,3903	-0,674512	-1,00981	0,07785	0,259765	no
XLOC_004640	g346	134,491	180,422	0,423864	0,730075	0,19875	0,451788	no
XLOC_004641	g8012	45,9	33,0075	-0,475701	-0,799227	0,16565	0,408406	no
XLOC_004642	g8013	68,6613	68,2928	-0,00776497	-0,0128196	0,98155	0,990985	no
XLOC_004643	g8014	0	0	0	0	1	1	no
XLOC_004644	g8016	8,80122	6,80648	-0,370795	-0,474315	0,40785	0,660523	no
XLOC_004645	g8015	17,2087	13,4008	-0,360822	-0,560278	0,32965	0,590615	no
XLOC_004646	g2479	0	0	0	0	1	1	no
XLOC_004647	g2478	0,542481	0,354205	-0,614989	0	1	1	no
XLOC_004648	g2480	8,82635	17,7614	1,00886	1,47055	0,01145	0,0731073	no
XLOC_004649	g2481	27,4181	74,0674	1,43371	2,15858	0,0002	0,00367103	yes
XLOC_004650	g8017	37,9495	79,2943	1,06314	1,70046	0,00385	0,0340297	yes
XLOC_004651	g8018	227,276	388,29	0,772693	1,14518	0,03915	0,167518	no
XLOC_004652	g8019	4,07258	6,24448	0,616638	0,792827	0,1646	0,406737	no
XLOC_004653	g8020	4,164	4,99195	0,261634	0,314664	0,57935	0,782561	no
XLOC_004654	g2482	0	0	0	0	1	1	no
XLOC_004655	g2484	18,277	16,6237	-0,13678	-0,207903	0,72125	0,868141	no
XLOC_004656	g2483	72,1736	106,278	0,558301	0,960764	0,09265	0,290215	no
XLOC_004657	g8021	16,3208	14,5085	-0,16981	-0,276075	0,61425	0,805977	no
XLOC_004658	g8022	40,624	62,2658	0,616109	1,04387	0,0664	0,234381	no
XLOC_004659	g8023	33,8502	83,1644	1,2968	1,73932	0,003	0,0284638	yes
XLOC_004660	g348	27,6143	40,6579	0,55812	0,872856	0,12665	0,351229	no
XLOC_004661	g349	30,4027	31,0663	0,0311483	0,0518155	0,92905	0,968397	no
XLOC_004662	g350	38,0832	29,6797	-0,359677	-0,44683	0,42965	0,678074	no
XLOC_004663	g351	144,422	95,2615	-0,60033	-0,945082	0,0958	0,295649	no
XLOC_004664	g353	76,2433	57,5533	-0,405712	-0,67734	0,21915	0,476329	no
XLOC_004665	g354	21,3194	14,0668	-0,599872	-0,824125	0,1572	0,396126	no
XLOC_004666	g352	10,0678	8,7185	-0,207594	-0,26179	0,65175	0,829167	no
XLOC_004667	g8024	46,16	75,4543	0,70896	0,997535	0,0798	0,264372	no
XLOC_004668	g8025	4,71726	5,5923	0,245493	0,324968	0,5759	0,780604	no
XLOC_004669	g8026	29,1785	27,2774	-0,0972015	-0,159394	0,77625	0,895638	no
XLOC_004670	g8027	34,5077	21,3903	-0,689958	-1,11348	0,0514	0,200059	no
XLOC_004671	g8028	8,82068	6,6979	-0,39718	-0,41576	0,4954	0,725877	no
XLOC_004672	g8029	1,11388	2,65676	1,25408	1,14508	0,0577	0,214952	no
XLOC_004673	g8030	0	0	0	0	1	1	no
XLOC_004674	g8031	0,0792947	2,18727	4,78576	1,21029	0,1236	0,347009	no
XLOC_004675	g8032	29,7309	15,9376	-0,899531	-0,994451	0,0753	0,253495	no
XLOC_004676	g8033	0	0	0	0	1	1	no
XLOC_004677	g8035	50,4377	26,6545	-0,920121	-1,54323	0,0063	0,0482574	yes
XLOC_004678	g8034	41,8453	49,5099	0,242652	0,38128	0,51585	0,741893	no
XLOC_004679	g8036	113,346	87,8869	-0,36701	-0,624472	0,27625	0,537929	no
XLOC_004680	g8038	0	0	0	0	1	1	no
XLOC_004681	g8037	0	0	0	0	1	1	no
XLOC_004682	g2485	395,342	282,948	-0,482564	-0,722498	0,207	0,461672	no
XLOC_004683	g355	72,5359	72,4961	-0,000791325	-0,00134911	0,9981	0,998507	no
XLOC_004684	g356	48,2359	41,1909	-0,227782	-0,378401	0,52025	0,744115	no
XLOC_004685	g357	70,379	82,4231	0,227903	0,349002	0,53575	0,755141	no
XLOC_004686	g2487	26,2797	32,6966	0,315194	0,462351	0,4202	0,671172	no
XLOC_004687	g2486	81,2667	69,0885	-0,234218	-0,392779	0,49565	0,725992	no
XLOC_004688	g8039	49,5309	72,4884	0,549421	0,898043	0,11525	0,333163	no
XLOC_004689	g2488	162,827	153,799	-0,0822944	-0,142782	0,81075	0,912919	no
XLOC_004690	g2489	3,97468	2,88853	-0,460507	-0,434945	0,45825	0,701013	no
XLOC_004691	g8041	4,76398	2,50835	-0,925428	-1,30968	0,02695	0,133036	no

XLOC_004692	g8044	141,559	94,5053	-0,582939	-0,932293	0,1027	0,308792	no
XLOC_004693	g8040	11,6351	9,98806	-0,220204	-0,297228	0,60365	0,799224	no
XLOC_004694	g8042	42,8412	40,332	-0,0870735	-0,13858	0,80495	0,91004	no
XLOC_004695	g8043	39,0989	24,554	-0,671172	-1,10445	0,05925	0,218489	no
XLOC_004696	g8045	41,0543	49,3189	0,264607	0,458464	0,42335	0,67289	no
XLOC_004697	g8046	10,3497	10,8628	0,0698141	0,0783832	0,8936	0,952578	no
XLOC_004698	g8047	4,54046	1,57041	-1,5317	-1,82055	0,0032	0,0297576	yes
XLOC_004699	g8048	42,1609	24,7219	-0,770114	-1,14316	0,03865	0,166386	no
XLOC_004700	g8049	56,8087	22,013	-1,36775	-2,24127	0,0002	0,00367103	yes
XLOC_004701	g2490	72,6097	64,8006	-0,164154	-0,198088	0,7264	0,869908	no
XLOC_004702	g2491	25,4924	14,8731	-0,777368	-1,22492	0,0306	0,143091	no
XLOC_004703	g8051	103,921	116,487	0,164674	0,279334	0,62265	0,810609	no
XLOC_004704	g8050	36,8281	69,2577	0,91117	1,49061	0,01165	0,0741432	no
XLOC_004705	g8052	259,69	147,365	-0,817399	-1,32034	0,02115	0,113431	no
XLOC_004706	g2492	41,4142	139,09	1,74782	2,59238	5,00E-05	0,00120049	yes
XLOC_004707	g2494	0,93147	0,325698	-1,51598	0	1	1	no
XLOC_004708	g2496	6,26134	15,4025	1,29862	1,76644	0,00195	0,0205021	yes
XLOC_004709	g2493	3,59578	2,17671	-0,724155	-0,829641	0,1435	0,379217	no
XLOC_004710	g2495	42,9707	45,8508	0,0935944	0,144608	0,7997	0,907867	no
XLOC_004711	g2497	14,4685	16,7184	0,208519	0,302773	0,5958	0,793092	no
XLOC_004712	g8053	80,5098	32,1452	-1,32456	-1,62496	0,0056	0,0440994	yes
XLOC_004713	g2499	36,3569	47,1433	0,374823	0,564197	0,31665	0,577009	no
XLOC_004714	g2498	50,8313	87,6622	0,786238	1,30304	0,01845	0,102593	no
XLOC_004715	g8054	9,64304	7,73332	-0,3184	-0,460106	0,4175	0,669473	no
XLOC_004716	g8055	8,19895	6,10248	-0,426042	-0,616458	0,2847	0,546579	no
XLOC_004717	g8056	17,9992	16,1769	-0,154	-0,225423	0,6981	0,85623	no
XLOC_004718	g8057	0	0	0	0	1	1	no
XLOC_004719	g2501	349,93	710,911	1,0226	1,46026	0,01375	0,0835551	no
XLOC_004720	g2502	38,9104	67,4336	0,793312	1,32183	0,01885	0,10411	no
XLOC_004721	g2503	36,2827	76,0495	1,06766	1,84897	0,0014	0,016042	yes
XLOC_004722	g2504	1,9623	1,38295	-0,5048	-0,533558	0,3692	0,628841	no
XLOC_004723	g2507	149,645	103,822	-0,527431	-0,867196	0,1373	0,369495	no
XLOC_004724	g2500	8,22032	21,2635	1,37111	1,96873	0,0009	0,0115984	yes
XLOC_004725	g2505	19,6806	7,98181	-1,30198	-1,78019	0,0017	0,0185283	yes
XLOC_004726	g2506	123,383	124,642	0,0146506	0,0249571	0,96475	0,984142	no
XLOC_004727	g2508	43,2815	90,3457	1,0617	1,804	0,00195	0,0205021	yes
XLOC_004728	g358	58,1027	40,5925	-0,517393	-0,875704	0,128	0,353376	no
XLOC_004729	g360	13,8442	14,2343	0,0400908	0,0609071	0,91135	0,962529	no
XLOC_004730	g361	358,946	249,294	-0,52592	-0,830998	0,13265	0,362041	no
XLOC_004731	g363	346,659	309,648	-0,162885	-0,233829	0,6794	0,845777	no
XLOC_004732	g364	59,3955	62,9387	0,0835926	0,143889	0,8066	0,910938	no
XLOC_004733	g365	44,6268	66,992	0,586078	0,953729	0,0915	0,287898	no
XLOC_004734	g366	68,8269	69,9171	0,0226725	0,0366736	0,9472	0,97628	no
XLOC_004735	g359	14,7355	21,0163	0,512212	0,848061	0,1281	0,353454	no
XLOC_004736	g362	39,0969	23,3842	-0,74152	-1,12209	0,0509	0,199059	no
XLOC_004737	g8058	204,038	164,466	-0,311046	-0,514628	0,3665	0,626221	no
XLOC_004738	g8059	181,38	182,27	0,00705809	0,0116513	0,98495	0,992425	no
XLOC_004739	g8060	87,6181	66,9758	-0,38759	-0,580329	0,31645	0,577009	no
XLOC_004740	g8061	2,51482	0	#NAME?	0	1	1	no
XLOC_004741	g8062	25,1125	34,0624	0,439774	0,595061	0,2936	0,555829	no
XLOC_004742	g8063	6,99741	4,18969	-0,739979	-1,02342	0,07675	0,256617	no
XLOC_004743	g8064	0,174975	0,0808822	-1,11326	0	1	1	no
XLOC_004744	g8066	28,8195	27,2226	-0,0822441	-0,139324	0,79815	0,907108	no
XLOC_004745	g8065	0	0	0	0	1	1	no
XLOC_004746	g8067	100,133	73,2792	-0,450436	-0,597927	0,28895	0,552363	no
XLOC_004747	g8068	10,5349	14,4822	0,459106	0,666451	0,24785	0,506634	no
XLOC_004748	g8069	0	0	0	0	1	1	no
XLOC_004749	g2509	0	0	0	0	1	1	no
XLOC_004750	g8070	31,8331	17,4954	-0,863553	-1,20315	0,03855	0,166386	no
XLOC_004751	g8071	63,6587	121,836	0,936509	1,41146	0,0145	0,0866119	no
XLOC_004752	g8072	34,0483	30,4653	-0,160416	-0,254554	0,6522	0,829397	no
XLOC_004753	g8074	95,7762	89,0927	-0,104361	-0,164777	0,7677	0,89164	no
XLOC_004754	g8073	101,508	102,344	0,0118381	0,0198787	0,9711	0,986977	no
XLOC_004755	g8075	0	0	0	0	1	1	no
XLOC_004756	g8076	0	0,129275	inf	0	1	1	no
XLOC_004757	g8077	8,5493	9,08703	0,0880032	0,0976567	0,8678	0,939455	no
XLOC_004758	g2510	13,4018	10,6467	-0,332017	-0,483923	0,4065	0,659969	no
XLOC_004759	g8078	0	0	0	0	1	1	no
XLOC_004760	g2512	62,8733	49,5497	-0,343571	-0,581012	0,31265	0,573414	no
XLOC_004761	g2513	7,38103	10,6898	0,534343	0,653169	0,2531	0,512145	no
XLOC_004762	g2511	55,5275	77,7331	0,485326	0,808124	0,1422	0,376999	no
XLOC_004763	g8079	8,65253	9,62902	0,154266	0,220405	0,69405	0,854464	no
XLOC_004764	g8081	34,2649	36,5485	0,0930817	0,146536	0,7961	0,906216	no
XLOC_004765	g8080	15,7742	11,3648	-0,472992	-0,693708	0,2316	0,48931	no
XLOC_004766	g8082	223,797	86,4662	-1,37198	-2,28299	0,00015	0,00294012	yes
XLOC_004767	g8083	47,7789	73,9128	0,629452	0,85415	0,14245	0,37756	no
XLOC_004768	g8084	145,093	173,588	0,258689	0,373506	0,51675	0,741989	no
XLOC_004769	g2514	244,287	159,405	-0,615875	-0,991016	0,08055	0,265972	no
XLOC_004770	g2516	31,6969	32,9017	0,0538196	0,0818934	0,883	0,94776	no
XLOC_004771	g2518	5,9956	5,05729	-0,245538	-0,273924	0,6254	0,812208	no
XLOC_004772	g2519	1981,39	1674,15	-0,24308	-0,246256	0,6597	0,832895	no
XLOC_004773	g2515	281,427	121,447	-1,21243	-1,99149	0,00055	0,00788467	yes
XLOC_004774	g2517	1600,02	2061,49	0,365599	0,550906	0,3268	0,587654	no
XLOC_004775	g8085	0	0	0	0	1	1	no
XLOC_004776	g2521	219,248	288,283	0,394923	0,616156	0,27715	0,538827	no
XLOC_004777	g2523	1233,37	1806,29	0,550427	0,846729	0,1306	0,357838	no
XLOC_004778	g2524	24,121	25,959	0,105947	0,17448	0,75985	0,886717	no
XLOC_004779	g2525	261,701	153,758	-0,767255	-1,12875	0,0525	0,203212	no
XLOC_004780	g2520	38,4363	30,5709	-0,330309	-0,566806	0,3201	0,580479	no
XLOC_004781	g2522	13876,8	8854,63	-0,648168	-1,05339	0,06435	0,229955	no
XLOC_004782	g2526	9,26688	7,54467	-0,296626	-0,397844	0,46915	0,707556	no
XLOC_004783	g8086	14,0453	24,8378	0,822447	1,05139	0,07635	0,255715	no

XLOC_004784	g8087	14,6864	13,7119	-0,0990497	-0,141658	0,7987	0,907467	no
XLOC_004785	g8088	0	0	0	0	1	1	no
XLOC_004786	g2527	13,4563	6,13091	-1,13411	-1,57483	0,00805	0,0571591	no
XLOC_004787	g2528	31,6132	76,6309	1,2774	1,88681	0,0018	0,0192549	yes
XLOC_004788	g2529	67,6764	100,115	0,564941	0,847642	0,13655	0,368385	no
XLOC_004789	g8089	66,7481	126,618	0,923687	1,3657	0,01615	0,093071	no
XLOC_004790	g8090	0	0	0	0	1	1	no
XLOC_004791	g2530	2,52427	2,5587	0,0195457	0,0217145	0,9769	0,98873	no
XLOC_004792	g2531	30,667	25,0224	-0,293471	-0,462224	0,4079	0,660523	no
XLOC_004793	g8091	6,63419	9,61245	0,534984	0,65948	0,2675	0,528541	no
XLOC_004794	g8092	19,8555	19,8278	-0,00201815	-0,00279558	0,9963	0,997874	no
XLOC_004795	g8093	24,8745	24,2656	-0,0357542	-0,057597	0,915	0,963526	no
XLOC_004796	g8094	0	0	0	0	1	1	no
XLOC_004797	g8095	0	0	0	0	1	1	no
XLOC_004798	g8096	12,6239	7,51129	-0,749023	-0,924945	0,1201	0,341059	no
XLOC_004799	g8097	1,15023	0,114884	-3,32367	-1,81582	0,0476	0,189628	no
XLOC_004800	g8098	62,2326	57,4436	-0,115523	-0,193182	0,7257	0,869807	no
XLOC_004801	g8099	12,0679	9,5212	-0,341964	-0,363118	0,5366	0,75558	no
XLOC_004802	g8100	96,5323	48,7571	-0,985398	-1,65688	0,00485	0,0400901	yes
XLOC_004803	g8102	50,8439	20,438	-1,31482	-1,83176	0,00205	0,0212577	yes
XLOC_004804	g8101	151,6	233	0,620057	0,9315	0,10265	0,308736	no
XLOC_004805	g2533	0,681475	0,239587	-1,50811	0	1	1	no
XLOC_004806	g2534	6,65903	5,95279	-0,161744	-0,228155	0,68875	0,850713	no
XLOC_004807	g2535	19,5638	12,2267	-0,678156	-0,717233	0,4154	0,667741	no
XLOC_004808	g2537	9,2341	26,5955	1,52614	2,09051	0,00045	0,00669545	yes
XLOC_004809	g2532	33,1691	50,8976	0,617757	0,594705	0,31045	0,571193	no
XLOC_004810	g2536	19,9693	21,4556	0,103573	0,128442	0,8213	0,918849	no
XLOC_004811	g8103	8,16593	13,8144	0,758485	0,936356	0,0923	0,289488	no
XLOC_004812	g8104	58,9679	56,6195	-0,0586315	-0,0949889	0,8632	0,936801	no
XLOC_004813	g8105	14,508	25,1499	0,793698	0,838959	0,1456	0,381889	no
XLOC_004814	g2539	195,023	191,281	-0,0279566	-0,0398709	0,9451	0,975499	no
XLOC_004815	g2538	114,127	146,196	0,357266	0,551755	0,32655	0,587312	no
XLOC_004816	g8106	0,0997076	0	#NAME?	0	1	1	no
XLOC_004817	g8108	10,7879	7,4235	-0,539245	-0,542006	0,3361	0,596081	no
XLOC_004818	g8107	153,235	125,834	-0,284224	-0,41276	0,44735	0,693445	no
XLOC_004819	g8109	20,7339	18,8301	-0,138952	-0,190479	0,7265	0,869922	no
XLOC_004820	g8110	57,6802	54,347	-0,085877	-0,136689	0,8041	0,90945	no
XLOC_004821	g8111	2,25347	0	#NAME?	#NAME?	0,0148	0,0877633	no
XLOC_004822	g2540	5,00031	8,89297	0,830648	1,20115	0,02765	0,134818	no
XLOC_004823	g8112	0	0,0453368	inf	0	1	1	no
XLOC_004824	g8113	0	0,219991	inf	0	1	1	no
XLOC_004825	g2541	659,881	296,518	-1,15409	-1,55072	0,0104	0,0684046	no
XLOC_004826	g2543	2,56704	4,91674	0,937595	0,684911	0,31805	0,578273	no
XLOC_004827	g2544	20,6802	6,22459	-1,7322	-2,19274	0,0052	0,0421668	yes
XLOC_004828	g2542	5,48461	1,55317	-1,82017	-1,85088	0,00495	0,0407111	yes
XLOC_004829	g2545	0	0	0	0	1	1	no
XLOC_004830	g2548	53,0291	65,0001	0,29366	0,441414	0,42795	0,677386	no
XLOC_004831	g2550	15,9112	8,7803	-0,857698	-1,25964	0,03375	0,15273	no
XLOC_004832	g2552	9,968	19,6851	0,981725	1,27656	0,0271	0,133261	no
XLOC_004833	g2554	60,4687	33,5238	-0,851003	-1,25283	0,0297	0,140964	no
XLOC_004834	g2556	55,4004	55,0632	-0,00880606	-0,0137929	0,98105	0,990634	no
XLOC_004835	g2557	1,36737	5,55059	2,02124	1,18912	0,27605	0,53785	no
XLOC_004836	g2559	97,6629	396,634	2,02193	2,60478	0,0002	0,00367103	yes
XLOC_004837	g2560	113,087	108,776	-0,0560699	-0,0968052	0,86445	0,937482	no
XLOC_004838	g2561	160,198	119,907	-0,417937	-0,689482	0,21015	0,465105	no
XLOC_004839	g2563	64,0142	84,5857	0,40202	0,682526	0,2331	0,49079	no
XLOC_004840	g2565	15,4252	16,7373	0,117783	0,175698	0,7539	0,884081	no
XLOC_004841	g2567	36,9859	40,4899	0,130589	0,212556	0,70095	0,85795	no
XLOC_004842	g2570	78,0843	67,0915	-0,218902	-0,281969	0,6192	0,808526	no
XLOC_004843	g2546	11,4718	27,9731	1,28595	1,93718	0,0007	0,00954722	yes
XLOC_004844	g2547	23,7135	27,6918	0,223745	0,359955	0,5288	0,74972	no
XLOC_004845	g2549	341,691	439,695	0,363809	0,513277	0,35685	0,617274	no
XLOC_004846	g2551	46,413	25,7606	-0,849363	-1,12761	0,05345	0,205352	no
XLOC_004847	g2553	75,2199	99,0532	0,397088	0,634473	0,24965	0,508092	no
XLOC_004848	g2555	55,9025	30,7881	-0,860539	-1,39477	0,01555	0,0907314	no
XLOC_004849	g2558	21,9667	34,8125	0,664284	1,10783	0,0456	0,184733	no
XLOC_004850	g2562	37,3779	45,4351	0,28162	0,454191	0,40955	0,662373	no
XLOC_004851	g2564	192,242	135,745	-0,502025	-0,745082	0,19705	0,448651	no
XLOC_004852	g2566	21,8049	34,6686	0,668978	1,08919	0,0494	0,195136	no
XLOC_004853	g2568	98,7548	105,69	0,0979128	0,169341	0,76905	0,89268	no
XLOC_004854	g2569	30,2951	32,0184	0,0798194	0,133396	0,81795	0,917235	no
XLOC_004855	g2571	32,9401	18,4928	-0,832881	-1,10643	0,0505	0,198047	no
XLOC_004856	g2572	0	0	0	0	1	1	no
XLOC_004857	g8114	10,6221	29,5549	1,47633	2,25336	0,0001	0,00209829	yes
XLOC_004858	g8115	0	0	0	0	1	1	no
XLOC_004859	g2574	30,9926	75,7817	1,28993	1,67852	0,0038	0,0337091	yes
XLOC_004860	g2573	324,226	143,435	-1,1766	-1,63096	0,00645	0,0491	yes
XLOC_004861	g2576	17,8468	28,1688	0,658429	0,911269	0,13085	0,358423	no
XLOC_004862	g2575	39,0918	46,3404	0,245403	0,356108	0,53035	0,750941	no
XLOC_004863	g2577	6,2488	3,35851	-0,89576	-1,15383	0,04685	0,187936	no
XLOC_004864	g8116	0	0	0	0	1	1	no
XLOC_004865	g8117	0	0	0	0	1	1	no
XLOC_004866	g8118	0	0	0	0	1	1	no
XLOC_004867	g8119	246,525	105,045	-1,23073	-1,9446	0,00065	0,00901554	yes
XLOC_004868	g8121	32,9378	16,2663	-1,01786	-1,51379	0,00705	0,0520143	no
XLOC_004869	g8120	9,24402	17,0179	0,880457	1,26578	0,02665	0,132107	no
XLOC_004870	g8122	0	0	0	0	1	1	no
XLOC_004871	g2579	62,4592	114,861	0,878897	1,48962	0,01495	0,0883859	no
XLOC_004872	g2578	120,615	119,931	-0,0081975	-0,0142067	0,9821	0,991184	no
XLOC_004873	g2580	427,142	289,532	-0,560991	-0,818738	0,1512	0,388687	no
XLOC_004874	g8124	20,5361	23,7856	0,211929	0,332601	0,5652	0,774704	no
XLOC_004875	g8123	10,8508	16,0765	0,567145	0,756059	0,1991	0,451802	no

XLOC_004876	g8125	23,5287	12,4575	-0,917405	-1,31757	0,0209	0,112459	no
XLOC_004877	g8126	12,4987	20,5763	0,719204	0,982887	0,08665	0,278073	no
XLOC_004878	g8128	127,904	273,713	1,0976	1,52274	0,01005	0,0667282	no
XLOC_004879	g8127	167,422	237,308	0,503274	0,660898	0,25615	0,515661	no
XLOC_004880	g8129	0	0,0464289	inf	0	1	1	no
XLOC_004881	g2581	17,3878	18,8554	0,116905	0,158136	0,77425	0,894738	no
XLOC_004882	g2582	3,32972	0,308386	-3,43259	-1,42393	0,11485	0,332398	no
XLOC_004883	g2584	140,628	92,9356	-0,597581	-0,906244	0,1196	0,340032	no
XLOC_004884	g2583	8,05534	7,50352	-0,102379	-0,145322	0,7992	0,907785	no
XLOC_004885	g2585	108,975	239,314	1,13491	1,90816	0,00115	0,0138564	yes
XLOC_004886	g8130	0	0	0	0	1	1	no
XLOC_004887	g2586	106,463	110,123	0,0487607	0,0830604	0,8873	0,949988	no
XLOC_004888	g2587	77,8612	80,1848	0,0424242	0,0707925	0,89735	0,954356	no
XLOC_004889	g8131	0	0	0	0	1	1	no
XLOC_004890	g8132	28,6942	27,728	-0,0494158	-0,072172	0,90165	0,956954	no
XLOC_004891	g8133	51,9911	161,244	1,63291	2,55928	5,00E-05	0,00120049	yes
XLOC_004892	g8134	3,62186	8,98094	1,31014	1,39329	0,0233	0,121254	no
XLOC_004893	g8135	3,42347	9,32203	1,44519	1,63688	0,00785	0,0561859	no
XLOC_004894	g8136	5,33285	19,8417	1,89555	2,32062	0,0002	0,00367103	yes
XLOC_004895	g8137	39,1658	50,5137	0,367081	0,62804	0,2718	0,53315	no
XLOC_004896	g8138	18,4176	15,5015	-0,248676	-0,369899	0,5055	0,733345	no
XLOC_004897	g8139	28,5035	128,814	2,17608	3,21347	5,00E-05	0,00120049	yes
XLOC_004898	g8140	52,6794	62,2743	0,241397	0,372584	0,5189	0,743557	no
XLOC_004899	g8141	31,1253	21,7414	-0,517645	-0,673895	0,2462	0,505152	no
XLOC_004900	g8142	188,083	89,8951	-1,06506	-1,66214	0,0056	0,0440994	yes
XLOC_004901	g8143	430,023	284,733	-0,594804	-0,926895	0,10305	0,309371	no
XLOC_004902	g8145	2,44954	3,32808	0,44218	0,444799	0,5009	0,730196	no
XLOC_004903	g8144	11,9419	20,5664	0,784255	1,07268	0,06025	0,220932	no
XLOC_004904	g8146	197,059	278,992	0,501598	0,826759	0,1536	0,39178	no
XLOC_004905	g8148	159,195	294,257	0,886284	1,43243	0,01515	0,0890856	no
XLOC_004906	g8147	56,5345	84,1884	0,57449	0,955624	0,09835	0,300778	no
XLOC_004907	g8149	21,0592	34,0104	0,691523	1,07175	0,0656	0,232476	no
XLOC_004908	g8150	0	0	0	0	1	1	no
XLOC_004909	g8151	0	0,202702	inf	0	1	1	no
XLOC_004910	g2588	45,4493	38,1826	-0,251344	-0,434129	0,45725	0,701012	no
XLOC_004911	g2589	38,0394	57,6178	0,599021	0,98995	0,07765	0,259185	no
XLOC_004912	g2591	37,0536	33,2377	-0,156795	-0,257451	0,65675	0,8318	no
XLOC_004913	g2590	46,7546	33,8014	-0,468025	-0,805196	0,1599	0,401077	no
XLOC_004914	g2592	3,70216	66,7384	4,17208	6,00742	5,00E-05	0,00120049	yes
XLOC_004915	g8152	94,5303	156,789	0,729975	1,10349	0,0547	0,208199	no
XLOC_004916	g8153	6,43545	3,17625	-1,01872	-1,20689	0,0328	0,149882	no
XLOC_004917	g2593	28,1365	12,9758	-1,11662	-1,1791	0,0318	0,14654	no
XLOC_004918	g2595	4,53353	3,54931	-0,353095	-0,464802	0,423	0,672849	no
XLOC_004919	g2596	58,9848	20,3227	-1,53725	-2,41613	0,00015	0,00294012	yes
XLOC_004920	g2597	22,8477	19,0748	-0,260384	-0,417071	0,48	0,715483	no
XLOC_004921	g2598	85,8714	40,598	-1,08077	-1,70486	0,0035	0,0317359	yes
XLOC_004922	g2600	6,74725	3,16417	-1,09247	-1,45534	0,01285	0,0799158	no
XLOC_004923	g2594	17,7551	14,083	-0,334276	-0,521727	0,3697	0,629256	no
XLOC_004924	g2599	12,398	5,67922	-1,12635	-1,61695	0,006	0,0465771	yes
XLOC_004925	g2601	76,9318	78,4942	0,0290068	0,0466088	0,9333	0,970869	no
XLOC_004926	g2602	151,489	144,76	-0,0655514	-0,112426	0,84215	0,927848	no
XLOC_004927	g8154	200,439	170,656	-0,232073	-0,368017	0,5234	0,746189	no
XLOC_004928	g8155	115,211	141,162	0,293082	0,481829	0,403	0,656594	no
XLOC_004929	g8156	6,44789	5,85368	-0,139483	-0,202009	0,71225	0,863888	no
XLOC_004930	g8157	0	0	0	0	1	1	no
XLOC_004931	g8158	41,1099	50,1732	0,28743	0,475192	0,4009	0,654912	no
XLOC_004932	g8159	129,841	98,0869	-0,404619	-0,649018	0,2491	0,508092	no
XLOC_004933	g8160	71,1413	121,152	0,768065	1,16852	0,04365	0,1798	no
XLOC_004934	g8161	0	0	0	0	1	1	no
XLOC_004935	g8162	0	0	0	0	1	1	no
XLOC_004936	g8163	22,7136	32,4338	0,513945	0,591277	0,29955	0,561799	no
XLOC_004937	g8165	3,14513	8,61357	1,45349	1,57888	0,0069	0,051293	no
XLOC_004938	g8164	11,5501	11,8832	0,0410149	0,0512606	0,92755	0,967861	no
XLOC_004939	g8166	3,13787	6,97921	1,15328	1,28491	0,0314	0,145447	no
XLOC_004940	g8167	17,081	36,312	1,08805	1,46252	0,0096	0,0644815	no
XLOC_004941	g2603	45,1411	43,0433	-0,0686516	-0,115206	0,83635	0,925299	no
XLOC_004942	g8168	5,19377	6,48218	0,319697	0,29524	0,62145	0,809691	no
XLOC_004943	g8169	0	0	0	0	1	1	no
XLOC_004944	g8170	167,132	146,321	-0,191848	-0,327349	0,5663	0,77517	no
XLOC_004945	g8171	0	0	0	0	1	1	no
XLOC_004946	g8172	85,4405	94,3658	0,143345	0,216828	0,70915	0,86197	no
XLOC_004947	g8173	0	0	0	0	1	1	no
XLOC_004948	g8174	17,1484	31,9449	0,897506	1,0045	0,1002	0,303505	no
XLOC_004949	g8175	15,3355	40,8487	1,41341	1,9761	0,00065	0,00901554	yes
XLOC_004950	g8176	0	0	0	0	1	1	no
XLOC_004951	g8177	0	0	0	0	1	1	no
XLOC_004952	g8178	353,348	227,71	-0,633896	-1,00343	0,09055	0,285733	no
XLOC_004953	g8179	2,10163	3,83873	0,869116	0,821215	0,1548	0,392851	no
XLOC_004954	g8180	13,6692	12,4005	-0,140535	-0,195943	0,7299	0,871761	no
XLOC_004955	g8181	39,4221	17,2691	-1,19081	-1,28014	0,03035	0,142533	no
XLOC_004956	g368	40,1919	37,6759	-0,0932646	-0,160412	0,7805	0,898182	no
XLOC_004957	g370	251,132	181,554	-0,468051	-0,730285	0,20895	0,464018	no
XLOC_004958	g367	18,6874	15,8989	-0,233147	-0,353621	0,52435	0,746357	no
XLOC_004959	g369	54,158	137,042	1,33938	2,25197	0,0001	0,00209829	yes
XLOC_004960	g371	6,34391	4,307	-0,558687	-0,536923	0,35375	0,614923	no
XLOC_004961	g8182	871,281	611,769	-0,51015	-0,579927	0,29255	0,555233	no
XLOC_004962	g8183	65,3383	72,1057	0,142184	0,222952	0,6997	0,857274	no
XLOC_004963	g8185	35,1781	30,5926	-0,201496	-0,33746	0,55245	0,76636	no
XLOC_004964	g8186	123,324	59,6622	-1,04757	-1,70121	0,00345	0,0313115	yes
XLOC_004965	g8189	17,2565	14,1719	-0,284105	-0,381858	0,5193	0,743805	no
XLOC_004966	g8184	17,7186	8,42522	-1,07248	-1,44423	0,0208	0,112044	no
XLOC_004967	g8187	46,6757	37,7783	-0,305116	-0,42897	0,4512	0,696117	no

XLOC_004968	g8188	21,9889	17,8847	-0,298051	-0,462932	0,41605	0,6681	no
XLOC_004969	g2604	25,5824	17,6371	-0,536538	-0,881005	0,12455	0,34796	no
XLOC_004970	g2605	28,7042	28,1493	-0,0281626	-0,0437192	0,9391	0,972781	no
XLOC_004971	g2607	0,749241	1,98152	1,40311	1,42204	0,0188	0,10411	no
XLOC_004972	g2606	0,654561	1,88923	1,5292	0,9885	0,15505	0,392851	no
XLOC_004973	g8190	20,3793	10,9034	-0,90233	-0,895991	0,12915	0,355253	no
XLOC_004974	g2610	2,87079	1,75675	-0,708539	-0,815853	0,1662	0,409249	no
XLOC_004975	g2608	329,649	279,465	-0,23826	-0,37187	0,5202	0,744115	no
XLOC_004976	g2609	5,07135	4,66785	-0,119611	-0,152814	0,79065	0,903527	no
XLOC_004977	g372	40,231	103,419	1,36213	2,28056	5,00E-05	0,00120049	yes
XLOC_004978	g375	110,679	17,4772	-2,66283	-3,93717	5,00E-05	0,00120049	yes
XLOC_004979	g373	20,6402	3,67758	-2,48863	-3,38575	5,00E-05	0,00120049	yes
XLOC_004980	g374	16,2126	17,8964	0,142551	0,201871	0,7204	0,86765	no
XLOC_004981	g376	3,06748	6,38867	1,05846	1,20046	0,04495	0,18293	no
XLOC_004982	g8191	0	0	0	0	1	1	no
XLOC_004983	g2612	0	0	0	0	1	1	no
XLOC_004984	g2611	0	0	0	0	1	1	no
XLOC_004985	g8192	440,704	696,581	0,660479	1,07656	0,05385	0,206404	no
XLOC_004986	g2613	20,3075	20,4595	0,0107594	0,0174535	0,9755	0,987975	no
XLOC_004987	g8193	217,675	90,7581	-1,26208	-2,01827	0,00035	0,00552572	yes
XLOC_004988	g8194	0	0	0	0	1	1	no
XLOC_004989	g2614	0	0	0	0	1	1	no
XLOC_004990	g2617	34,931	72,2905	1,0493	1,57224	0,00785	0,0561859	no
XLOC_004991	g2615	2,54335	5,44649	1,0986	1,24748	0,0299	0,141434	no
XLOC_004992	g2616	68,4522	113,255	0,726407	1,21987	0,0355	0,157671	no
XLOC_004993	g2618	66,6817	122,81	0,881068	1,48521	0,01005	0,0667282	no
XLOC_004994	g2619	0	0	0	0	1	1	no
XLOC_004995	g2620	20,9359	23,1315	0,14388	0,233352	0,67285	0,841277	no
XLOC_004996	g2621	0	0,393106	inf	0	1	1	no
XLOC_004997	g8195	71,298	44,2399	-0,688514	-1,15033	0,0416	0,174578	no
XLOC_004998	g8197	44,3956	35,093	-0,339235	-0,531156	0,34715	0,607451	no
XLOC_004999	g8198	37,0094	19,2555	-0,942622	-1,25524	0,03095	0,144179	no
XLOC_005000	g8200	8,14929	8,63557	0,083616	0,130392	0,8185	0,917235	no
XLOC_005001	g8196	67,0724	91,7603	0,452152	0,760984	0,1815	0,429167	no
XLOC_005002	g8199	16,696	15,0959	-0,145349	-0,238245	0,6815	0,846595	no
XLOC_005003	g8201	124,968	82,1413	-0,605376	-1,01178	0,0763	0,255715	no
XLOC_005004	g377	3,20517	7,85907	1,29396	0,807553	0,2503	0,508786	no
XLOC_005005	g378	4,18106	3,47307	-0,267655	-0,202969	0,71935	0,867399	no
XLOC_005006	g8202	171,786	94,3814	-0,864041	-1,45992	0,01415	0,0852472	no
XLOC_005007	g8203	86,8412	64,0291	-0,439652	-0,738642	0,1923	0,442349	no
XLOC_005008	g8204	31,545	28,9844	-0,122133	-0,20109	0,7241	0,86956	no
XLOC_005009	g8205	1,04783	2,27263	1,11696	0,969188	0,0964	0,297002	no
XLOC_005010	g8206	2,12477	1,03813	-1,03332	-1,06509	0,07665	0,256537	no
XLOC_005011	g8207	0	0	0	0	1	1	no
XLOC_005012	g2622	33,4516	31,6888	-0,0781057	-0,121086	0,8308	0,922276	no
XLOC_005013	g2623	230,236	226,875	-0,0212164	-0,0362926	0,94725	0,97628	no
XLOC_005014	g8208	19,3331	10,1403	-0,930977	-1,26447	0,02405	0,123779	no
XLOC_005015	g8209	0	0	0	0	1	1	no
XLOC_005016	g380	33,4252	31,9766	-0,063917	-0,110193	0,846	0,929972	no
XLOC_005017	g382	49,0632	35,9238	-0,4497	-0,694612	0,22835	0,486526	no
XLOC_005018	g384	40,1481	36,9731	-0,118855	-0,202462	0,7197	0,867399	no
XLOC_005019	g379	597,305	1289,53	1,11031	1,17757	0,05495	0,208908	no
XLOC_005020	g381	0,834024	0,981598	0,235043	0	1	1	no
XLOC_005021	g383	16,1288	14,8511	-0,119075	-0,189463	0,7338	0,874259	no
XLOC_005022	g8210	165,908	108,48	-0,612951	-0,964633	0,08955	0,28358	no
XLOC_005023	g8211	0	0,390554	inf	0	1	1	no
XLOC_005024	g8212	0	0	0	0	1	1	no
XLOC_005025	g8213	0	0	0	0	1	1	no
XLOC_005026	g2625	31,0596	29,1402	-0,0920273	-0,124583	0,8302	0,922236	no
XLOC_005027	g2624	3,06797	1,97558	-0,635012	-0,790596	0,1735	0,419132	no
XLOC_005028	g2626	10,1704	8,63049	-0,236857	-0,33654	0,5474	0,76377	no
XLOC_005029	g8214	0	0	0	0	1	1	no
XLOC_005030	g8215	0	0	0	0	1	1	no
XLOC_005031	g8216	0,437742	0,239097	-0,872489	0	1	1	no
XLOC_005032	g8217	18,494	39,8452	1,10734	1,69593	0,00365	0,0326439	yes
XLOC_005033	g8218	80,279	89,6718	0,159632	0,276184	0,62765	0,813452	no
XLOC_005034	g2627	42,7884	104,842	1,29293	1,71248	0,00535	0,0426783	yes
XLOC_005035	g2628	78,0289	71,9844	-0,116325	-0,193553	0,72785	0,870385	no
XLOC_005036	g2629	52,6818	38,2992	-0,45999	-0,755301	0,19135	0,441819	no
XLOC_005037	g2630	671,39	598,456	-0,165906	-0,182289	0,74165	0,877635	no
XLOC_005038	g8219	0	1,00742	inf	0	1	1	no
XLOC_005039	g8220	5,11757	2,17369	-1,23531	-1,22859	0,0892	0,283019	no
XLOC_005040	g8221	0	0	0	0	1	1	no
XLOC_005041	g8222	0	0	0	0	1	1	no
XLOC_005042	g8223	0	0	0	0	1	1	no
XLOC_005043	g8224	1,03496	0,161796	-2,67733	0	1	1	no
XLOC_005044	g8225	0	0	0	0	1	1	no
XLOC_005045	g8226	3,85024	6,10246	0,664443	0,553547	0,3417	0,601774	no
XLOC_005046	g2632	258,558	129,298	-0,999791	-1,66156	0,00275	0,0265797	yes
XLOC_005047	g2631	8,90935	9,82669	0,141385	0,210298	0,70515	0,860058	no
XLOC_005048	g8227	17,5904	22,905	0,380874	0,27424	0,61225	0,805014	no
XLOC_005049	g8228	6,563	9,9608	0,601905	0,788415	0,1778	0,42461	no
XLOC_005050	g2633	39,0266	67,7992	0,79681	1,36249	0,01725	0,097634	no
XLOC_005051	g2634	68,6446	122,036	0,830093	1,36186	0,018	0,100603	no
XLOC_005052	g2635	78,2036	31,4041	-1,31628	-1,64637	0,0066	0,0498938	yes
XLOC_005053	g2637	17,3511	18,2804	0,0752663	0,100584	0,8673	0,939446	no
XLOC_005054	g2638	31,8195	38,1042	0,260037	0,377101	0,50675	0,734399	no
XLOC_005055	g2640	74,8644	28,5693	-1,38981	-1,95352	0,00115	0,0138564	yes
XLOC_005056	g2642	203,761	337,511	0,728055	1,24966	0,0307	0,143354	no
XLOC_005057	g2644	136,154	135,228	-0,00984601	-0,0166182	0,97645	0,98853	no
XLOC_005058	g2645	179,001	88,3438	-1,01877	-1,68686	0,00345	0,0313115	yes
XLOC_005059	g2646	114,292	393,023	1,78188	2,80551	5,00E-05	0,00120049	yes

XLOC_005060	g2647	0	0	0	0	1	1	no
XLOC_005061	g2636	176,558	191,623	0,118125	0,156406	0,77725	0,895827	no
XLOC_005062	g2639	22,425	22,5293	0,00669696	0,0112734	0,98335	0,991936	no
XLOC_005063	g2641	46,0308	40,384	-0,188816	-0,319957	0,57665	0,780632	no
XLOC_005064	g2643	65,4545	66,4946	0,0227456	0,036722	0,9494	0,977163	no
XLOC_005065	g8229	0,0313546	0	#NAME?	0	1	1	no
XLOC_005066	g8230	0	0	0	0	1	1	no
XLOC_005067	g2648	0	0	0	0	1	1	no
XLOC_005068	g8231	3,60304	3,0367	-0,246708	-0,312437	0,581	0,783281	no
XLOC_005069	g8232	135,78	126,859	-0,0980375	-0,16448	0,7853	0,901209	no
XLOC_005070	g8233	55,0282	57,2231	0,0564269	0,0918557	0,86925	0,940817	no
XLOC_005071	g8234	18,167	63,5772	1,80719	2,09084	0,00045	0,00669545	yes
XLOC_005072	g8235	71,0376	65,129	-0,125284	-0,214267	0,7153	0,865069	no
XLOC_005073	g8236	0	0	0	0	1	1	no
XLOC_005074	g8237	27,7259	38,022	0,455596	0,760126	0,17775	0,424594	no
XLOC_005075	g8238	3,15725	4,99231	0,661038	0,60084	0,31045	0,571193	no
XLOC_005076	g8240	109,389	76,9025	-0,50837	-0,811395	0,13685	0,368991	no
XLOC_005077	g8239	46,0438	43,0723	-0,0962442	-0,158161	0,77195	0,89378	no
XLOC_005078	g8242	40,1311	63,4642	0,661221	1,0632	0,06435	0,229955	no
XLOC_005079	g8243	137,412	95,581	-0,523712	-0,824386	0,1428	0,378079	no
XLOC_005080	g8241	45,6719	160,797	1,81587	2,85777	5,00E-05	0,00120049	yes
XLOC_005081	g8244	18,4814	14,1355	-0,386748	-0,501688	0,36435	0,623983	no
XLOC_005082	g8245	0	0	0	0	1	1	no
XLOC_005083	g8246	38,2204	37,5262	-0,0264475	-0,0447899	0,93655	0,972153	no
XLOC_005084	g8247	0	0	0	0	1	1	no
XLOC_005085	g8248	83,8094	85,1118	0,0222465	0,0337053	0,9526	0,978815	no
XLOC_005086	g8249	32,4687	18,5528	-0,807413	-1,28628	0,0284	0,137046	no
XLOC_005087	g8251	2,22347	0,544522	-2,02975	-1,35915	0,13925	0,372294	no
XLOC_005088	g8250	17,5064	23,2538	0,409584	0,649214	0,2495	0,508092	no
XLOC_005089	g8252	0	0	0	0	1	1	no
XLOC_005090	g8253	0	0	0	0	1	1	no
XLOC_005091	g8254	0	0	0	0	1	1	no
XLOC_005092	g8255	0	0	0	0	1	1	no
XLOC_005093	g8256	10,2318	22,9853	1,16765	1,40994	0,0154	0,0901777	no
XLOC_005094	g8257	0	0	0	0	1	1	no
XLOC_005095	g8258	0	0	0	0	1	1	no
XLOC_005096	g8259	0	0	0	0	1	1	no
XLOC_005097	g2649	36,8722	51,4199	0,479794	0,774948	0,16175	0,403553	no
XLOC_005098	g2651	115,687	84,3923	-0,455049	-0,790955	0,1741	0,420013	no
XLOC_005099	g2655	52,5908	40,4614	-0,378266	-0,610155	0,2887	0,551993	no
XLOC_005100	g2650	87,9786	55,2967	-0,669959	-1,12931	0,04355	0,179539	no
XLOC_005101	g2652	20,7087	13,076	-0,663311	-0,955878	0,09565	0,295526	no
XLOC_005102	g2653	170,276	270,08	0,665512	0,999502	0,06435	0,229955	no
XLOC_005103	g2654	117,209	25,9474	-2,17542	-3,42658	5,00E-05	0,00120049	yes
XLOC_005104	g2656	248,37	409,341	0,720811	0,827836	0,13995	0,373352	no
XLOC_005105	g2658	1703,34	2786,56	0,71012	1,11363	0,05465	0,20817	no
XLOC_005106	g2657	25,8405	53,8846	1,06024	1,54889	0,0086	0,0599801	no
XLOC_005107	g8260	47,3418	26,822	-0,819695	-1,15735	0,0473	0,189046	no
XLOC_005108	g2659	45,7845	42,8444	-0,095751	-0,158674	0,7776	0,895827	no
XLOC_005109	g2661	249,921	333,746	0,417277	0,604082	0,2864	0,549199	no
XLOC_005110	g2664	36,2781	56,7716	0,64607	1,10299	0,0523	0,202678	no
XLOC_005111	g2667	752,652	1073,42	0,512162	0,728816	0,1953	0,446852	no
XLOC_005112	g2669	45,0452	55,7581	0,307808	0,519793	0,36385	0,62367	no
XLOC_005113	g2670	50,5136	244,088	2,27266	3,49437	5,00E-05	0,00120049	yes
XLOC_005114	g2671	194,577	220,999	0,183703	0,247854	0,6668	0,837544	no
XLOC_005115	g2673	6,39612	13,0993	1,03422	1,09354	0,067	0,23599	no
XLOC_005116	g2674	101,299	74,5891	-0,441579	-0,719855	0,2177	0,474227	no
XLOC_005117	g2660	28,8403	30,1969	0,0663148	0,102772	0,8537	0,934493	no
XLOC_005118	g2662	44,6366	51,4329	0,204464	0,345031	0,5453	0,761892	no
XLOC_005119	g2663	2574,98	2879,11	0,161061	0,221816	0,69065	0,851926	no
XLOC_005120	g2665	69,7055	80,634	0,210115	0,355785	0,5422	0,760303	no
XLOC_005121	g2666	403,134	347,182	-0,215568	-0,365369	0,5092	0,735453	no
XLOC_005122	g2668	16,4296	15,5576	-0,078677	-0,118451	0,8391	0,92642	no
XLOC_005123	g2672	49,2155	32,4908	-0,59908	-0,950125	0,10235	0,308211	no
XLOC_005124	g2675	103,143	119,626	0,213877	0,338148	0,5491	0,765215	no
XLOC_005125	g2676	61,3467	81,3179	0,406588	0,677687	0,23495	0,492678	no
XLOC_005126	g8261	0	0	0	0	1	1	no
XLOC_005127	g8262	40,6693	66,1992	0,702875	1,16839	0,0442	0,180927	no
XLOC_005128	g8263	32,3296	29,131	-0,1503	-0,252285	0,65375	0,829883	no
XLOC_005129	g2678	240,76	289,76	0,267263	0,415998	0,45715	0,701012	no
XLOC_005130	g2682	438,811	1155,46	1,39679	2,15931	0,0004	0,0061761	yes
XLOC_005131	g2684	91,0342	106,888	0,231618	0,400581	0,4887	0,721659	no
XLOC_005132	g2677	29,4815	35,9786	0,287327	0,486613	0,4018	0,655646	no
XLOC_005133	g2679	71,2992	59,7774	-0,254286	-0,404813	0,47115	0,708963	no
XLOC_005134	g2680	148,333	101,529	-0,546941	-0,883506	0,1141	0,331302	no
XLOC_005135	g2681	313,203	469,803	0,584956	0,871997	0,124	0,347197	no
XLOC_005136	g2683	31,6902	48,1402	0,603206	0,958317	0,08635	0,277473	no
XLOC_005137	g8264	38,3533	34,5594	-0,150272	-0,249885	0,6698	0,839387	no
XLOC_005138	g8265	80,3249	30,1486	-1,41376	-2,27963	5,00E-05	0,00120049	yes
XLOC_005139	g2685	0	0	0	0	1	1	no
XLOC_005140	g8266	0	0	0	0	1	1	no
XLOC_005141	g2686	0	0	0	0	1	1	no
XLOC_005142	g8267	8,07839	4,4593	-0,85725	-1,18905	0,04285	0,177622	no
XLOC_005143	g8268	18,4019	12,7605	-0,528178	-0,77455	0,16115	0,402772	no
XLOC_005144	g8269	46,6599	89,1919	0,934729	0,674432	0,26425	0,524865	no
XLOC_005145	g8270	33,6661	30,2268	-0,155469	-0,2349	0,68075	0,846464	no
XLOC_005146	g2687	222,407	151,685	-0,552123	-0,867246	0,13775	0,370198	no
XLOC_005147	g8271	6,4306	1,67009	-1,94503	-2,25515	0,0003	0,00496796	yes
XLOC_005148	g8272	4,77501	4,29927	-0,151412	-0,14394	0,80355	0,908981	no
XLOC_005149	g8273	20,4518	11,6816	-0,807989	-1,25732	0,0384	0,165826	no
XLOC_005150	g8274	64,7979	35,3973	-0,872309	-1,20381	0,0387	0,166386	no
XLOC_005151	g8275	65,4481	68,5406	0,0666077	0,11021	0,8478	0,93094	no

XLOC_005152	g8276	0	0	0	0	1	1	no
XLOC_005153	g2690	59,4599	66,5757	0,163079	0,266363	0,6423	0,822774	no
XLOC_005154	g2691	116,179	112,573	-0,0454958	-0,0758731	0,896	0,953687	no
XLOC_005155	g2693	77,6707	92,3318	0,249458	0,418809	0,4661	0,706452	no
XLOC_005156	g2695	32,6217	29,9986	-0,120937	-0,206543	0,7142	0,865069	no
XLOC_005157	g2697	24,1682	24,579	0,0243168	0,0383227	0,94915	0,97711	no
XLOC_005158	g2699	7,20849	4,26332	-0,757719	-1,08805	0,05925	0,218489	no
XLOC_005159	g2688	41,6471	42,7529	0,0378062	0,0617715	0,91445	0,963407	no
XLOC_005160	g2689	151,256	286,983	0,923977	1,53813	0,0068	0,0508963	no
XLOC_005161	g2692	13,9306	10,8929	-0,354867	-0,530336	0,34375	0,603977	no
XLOC_005162	g2694	128,105	153,92	0,264851	0,45507	0,4217	0,671928	no
XLOC_005163	g2696	84,043	79,569	-0,0789211	-0,136164	0,81165	0,913618	no
XLOC_005164	g2698	54,6534	43,0459	-0,344433	-0,582361	0,30925	0,570405	no
XLOC_005165	g2700	26,6172	46,7883	0,813788	1,33695	0,0201	0,109292	no
XLOC_005166	g8277	0	0,145652	inf	0	1	1	no
XLOC_005167	g8278	146,149	236,785	0,696135	1,14694	0,0473	0,189046	no
XLOC_005168	g8279	88,1352	44,3166	-0,991871	-1,49362	0,0094	0,0637927	no
XLOC_005169	g8280	0	0	0	0	1	1	no
XLOC_005170	g8281	361,49	164,367	-1,13704	-1,8324	0,00175	0,0188639	yes
XLOC_005171	g8282	183,823	265,74	0,531693	0,821266	0,1441	0,380116	no
XLOC_005172	g8283	0	0	0	0	1	1	no
XLOC_005173	g8284	19,2817	13,7819	-0,484457	-0,613221	0,27305	0,53424	no
XLOC_005174	g8285	72,4969	34,4495	-1,07344	-1,48071	0,0114	0,0729303	no
XLOC_005175	g8287	11,6636	6,55724	-0,830854	-1,19692	0,036	0,158743	no
XLOC_005176	g8288	147,599	106,171	-0,475295	-0,733775	0,21425	0,469524	no
XLOC_005177	g8289	225,61	270,718	0,262958	0,396553	0,47995	0,715483	no
XLOC_005178	g8286	32,9503	19,3587	-0,767313	-1,17191	0,0449	0,182878	no
XLOC_005179	g2701	25,5253	31,7341	0,314106	0,506471	0,35755	0,618267	no
XLOC_005180	g8290	15,9071	32,0392	1,01017	1,50647	0,0074	0,0541087	no
XLOC_005181	g8291	0	0	0	0	1	1	no
XLOC_005182	g2702	104,942	78,1752	-0,424813	-0,739365	0,20005	0,452439	no
XLOC_005183	g2703	20,2489	28,4994	0,493089	0,809165	0,1591	0,399479	no
XLOC_005184	g8293	11,1011	13,7172	0,305282	0,409409	0,48635	0,719921	no
XLOC_005185	g8292	10,9858	16,5478	0,591003	0,675186	0,25385	0,513134	no
XLOC_005186	g8294	38,9956	38,1291	-0,0324182	-0,052417	0,92725	0,967857	no
XLOC_005187	g2704	10,7202	3,48666	-1,62041	-1,59318	0,01035	0,068167	no
XLOC_005188	g8295	0	0	0	0	1	1	no
XLOC_005189	g8296	63,1716	54,2081	-0,220768	-0,378779	0,5058	0,733528	no
XLOC_005190	g2705	0	0	0	0	1	1	no
XLOC_005191	g8297	346,302	183,343	-0,917483	-1,3816	0,0158	0,0916998	no
XLOC_005192	g8298	42,1172	40,5019	-0,0564192	-0,0873304	0,8784	0,94551	no
XLOC_005193	g8299	51,7665	50,7618	-0,0282744	-0,0446251	0,93965	0,973145	no
XLOC_005194	g2707	2,41764	1,41283	-0,775007	-0,781775	0,1941	0,445446	no
XLOC_005195	g2709	216,551	91,8076	-1,23802	-1,67345	0,00345	0,0313115	yes
XLOC_005196	g2712	39,1993	46,6128	0,249897	0,439155	0,45885	0,701036	no
XLOC_005197	g2713	38,9912	79,82	1,0336	1,74385	0,0037	0,032971	yes
XLOC_005198	g2715	16,0848	20,1145	0,32254	0,538145	0,3353	0,595523	no
XLOC_005199	g2716	8,72669	3,18058	-1,45614	-1,48632	0,01525	0,089406	no
XLOC_005200	g2720	23,8614	23,8715	0,00060972	0,000933123	0,99715	0,998008	no
XLOC_005201	g2721	15,8482	28,8675	0,865132	1,2339	0,0285	0,137326	no
XLOC_005202	g2722	28,4641	87,8176	1,62537	2,43639	0,0001	0,00209829	yes
XLOC_005203	g2706	79,6787	77,9748	-0,0311874	-0,0472667	0,9331	0,970764	no
XLOC_005204	g2708	137,267	126,711	-0,11544	-0,179252	0,7564	0,884794	no
XLOC_005205	g2710	13,0977	13,1459	0,00529021	0,00768007	0,9898	0,99501	no
XLOC_005206	g2711	6,21024	8,99293	0,534142	0,837286	0,15065	0,387882	no
XLOC_005207	g2714	41,066	50,9457	0,311016	0,409639	0,4684	0,707556	no
XLOC_005208	g2717	57,4013	54,0506	-0,0867717	-0,126796	0,82	0,918071	no
XLOC_005209	g2718	148,377	20,5496	-2,85209	-3,99457	5,00E-05	0,00120049	yes
XLOC_005210	g2719	133,231	101,4	-0,393879	-0,673323	0,2295	0,487604	no
XLOC_005211	g2723	56,9862	36,0881	-0,659089	-1,08149	0,06155	0,223694	no
XLOC_005212	g8300	0	0	0	0	1	1	no
XLOC_005213	g8301	10,618	9,43936	-0,169758	-0,219806	0,71135	0,863041	no
XLOC_005214	g8302	155,189	275,91	0,830175	1,24998	0,02865	0,137614	no
XLOC_005215	g8303	180,923	462,686	1,35466	1,93084	0,001	0,0126221	yes
XLOC_005216	g8304	0	0	0	0	1	1	no
XLOC_005217	g8306	7,02309	8,65553	0,301516	0,441686	0,44825	0,694183	no
XLOC_005218	g8305	45,2843	70,8208	0,645163	1,09535	0,0635	0,228247	no
XLOC_005219	g8307	214,944	73,4931	-1,54828	-1,94169	0,00095	0,0120686	yes
XLOC_005220	g8308	0	0	0	0	1	1	no
XLOC_005221	g8309	20,4277	21,4172	0,0682489	0,0982264	0,86325	0,936801	no
XLOC_005222	g385	25,3621	30,6831	0,274772	0,453116	0,4248	0,674133	no
XLOC_005223	g387	21,7136	15,2955	-0,505496	-0,596126	0,29365	0,555829	no
XLOC_005224	g386	4,0019	13,6788	1,77318	1,61664	0,0304	0,142632	no
XLOC_005225	g388	420,569	599,788	0,51211	0,804343	0,1429	0,378099	no
XLOC_005226	g8311	104,531	71,1782	-0,554423	-0,914392	0,10115	0,305723	no
XLOC_005227	g8313	72,6684	63,4006	-0,196832	-0,308168	0,5765	0,780604	no
XLOC_005228	g8310	12,5827	22,9986	0,870107	1,34432	0,0171	0,0969527	no
XLOC_005229	g8312	118,352	24,8495	-2,25179	-3,41923	5,00E-05	0,00120049	yes
XLOC_005230	g8314	1,11709	3,67017	1,7161	1,34812	0,34405	0,604287	no
XLOC_005231	g8315	14,37	16,0352	0,158176	0,202051	0,72015	0,867502	no
XLOC_005232	g8316	0	0	0	0	1	1	no
XLOC_005233	g8317	11,4751	9,12885	-0,330005	-0,480047	0,40505	0,658433	no
XLOC_005234	g2724	20,8333	11,2827	-0,884782	-1,21533	0,0386	0,166386	no
XLOC_005235	g2726	2,48081	1,70096	-0,544461	-0,395096	0,57765	0,781124	no
XLOC_005236	g2725	8,1413	2,86114	-1,50867	-1,23835	0,22735	0,48566	no
XLOC_005237	g8318	0	0	0	0	1	1	no
XLOC_005238	g8319	9,19147	5,63155	-0,706765	-0,857959	0,14075	0,374368	no
XLOC_005239	g2727	5,13506	3,93508	-0,383988	-0,434146	0,43855	0,684886	no
XLOC_005240	g8320	36,2886	18,8093	-0,948073	-1,48692	0,00885	0,061159	no
XLOC_005241	g8321	162,353	35,2431	-2,20372	-3,5182	5,00E-05	0,00120049	yes
XLOC_005242	g8323	123,845	127,028	0,0366172	0,0559293	0,92495	0,966895	no
XLOC_005243	g8325	148,969	136,365	-0,127537	-0,203353	0,7133	0,864233	no

XLOC_005244	g8326	3,22505	3,17022	-0,0247389	-0,0236289	0,9695	0,986579	no
XLOC_005245	g8328	56,5024	12,3118	-2,19827	-2,15856	0,00045	0,00669545	yes
XLOC_005246	g8329	484,021	1148,07	1,24606	1,98977	0,00085	0,011085	yes
XLOC_005247	g8332	125,305	144,717	0,207792	0,358349	0,52425	0,746323	no
XLOC_005248	g8333	71,2324	85,4983	0,263363	0,445894	0,4501	0,694824	no
XLOC_005249	g8322	21,2078	36,8153	0,795709	1,11484	0,05985	0,219875	no
XLOC_005250	g8324	51,5839	76,3572	0,565843	0,975875	0,0868	0,278372	no
XLOC_005251	g8327	5,36329	3,41223	-0,652403	-0,903437	0,1111	0,325284	no
XLOC_005252	g8330	17,0613	11,6903	-0,545419	-0,782154	0,17305	0,418456	no
XLOC_005253	g8331	46,7245	29,8286	-0,647483	-1,07378	0,05465	0,20817	no
XLOC_005254	g8334	12,4925	9,76518	-0,355344	-0,570619	0,31735	0,578178	no
XLOC_005255	g8335	0,950395	1,67658	0,81892	0,615707	0,29015	0,552935	no
XLOC_005256	g8336	0,644863	0,990364	0,618966	0	1	1	no
XLOC_005257	g8337	0	0	0	0	1	1	no
XLOC_005258	g8338	65,1175	33,2432	-0,969988	-1,45945	0,0105	0,06874	no
XLOC_005259	g8339	24,1035	18,7119	-0,365289	-0,502048	0,3785	0,636208	no
XLOC_005260	g8340	6,11459	8,13541	0,411958	0,785598	0,7916	0,904002	no
XLOC_005261	g8341	135,557	152,401	0,168977	0,275886	0,6341	0,816959	no
XLOC_005262	g8342	32,4605	14,7421	-1,13875	-1,79695	0,00215	0,022131	yes
XLOC_005263	g8344	74,3225	20,5282	-1,85619	-2,53542	5,00E-05	0,00120049	yes
XLOC_005264	g8343	65,3011	15,6131	-2,06435	-2,41389	0,00015	0,00294012	yes
XLOC_005265	g8345	68,7595	93,0578	0,436568	0,699088	0,21	0,464983	no
XLOC_005266	g8346	52,4892	60,2163	0,198133	0,338484	0,5559	0,768934	no
XLOC_005267	g8347	65,3393	81,4357	0,317711	0,524578	0,351	0,611572	no
XLOC_005268	g8348	148,928	128,536	-0,212445	-0,32642	0,56915	0,777229	no
XLOC_005269	g2728	110,683	102,533	-0,110347	-0,187633	0,7406	0,876968	no
XLOC_005270	g2729	21,6978	28,8231	0,409674	0,657262	0,25415	0,513318	no
XLOC_005271	g8349	114,169	55,6316	-1,03719	-1,57228	0,00745	0,0543933	no
XLOC_005272	g2732	128,148	127,08	-0,0120673	-0,0192982	0,97135	0,986977	no
XLOC_005273	g2730	46,1562	63,9422	0,470242	0,766713	0,1678	0,410922	no
XLOC_005274	g2731	58,2141	125,753	1,11115	1,76476	0,0022	0,0225747	yes
XLOC_005275	g2733	78,4023	169,229	1,11001	1,73555	0,00315	0,0293482	yes
XLOC_005276	g2734	22,4155	25,347	0,177317	0,271264	0,6383	0,820111	no
XLOC_005277	g8350	1,31006	2,98578	1,18848	1,33645	0,02105	0,113019	no
XLOC_005278	g8351	102,195	108,543	0,0869428	0,14238	0,803	0,908624	no
XLOC_005279	g8352	82,6115	56,2635	-0,554143	-0,929401	0,09965	0,302738	no
XLOC_005280	g390	41,2945	47,5062	0,202166	0,345045	0,5387	0,757667	no
XLOC_005281	g389	72,9437	58,055	-0,329364	-0,576517	0,3186	0,578951	no
XLOC_005282	g391	23,8468	24,6035	0,0450717	0,0765968	0,89065	0,951189	no
XLOC_005283	g8353	13,4615	8,82688	-0,608859	-0,755274	0,20415	0,457393	no
XLOC_005284	g8354	161,73	87,3399	-0,888878	-1,28761	0,0242	0,123967	no
XLOC_005285	g8355	0	0	0	0	1	1	no
XLOC_005286	g2735	19,6176	113,791	2,53617	3,41357	5,00E-05	0,00120049	yes
XLOC_005287	g2736	109,809	645,997	2,55652	3,65786	5,00E-05	0,00120049	yes
XLOC_005288	g8356	0,593794	0,336912	-0,817592	0	1	1	no
XLOC_005289	g8357	0	0,00366899	inf	0	1	1	no
XLOC_005290	g8358	0	0	0	0	1	1	no
XLOC_005291	g8359	22,7071	46,1936	1,02455	1,30035	0,0257	0,128828	no
XLOC_005292	g2737	104,081	127,758	0,295712	0,508898	0,3767	0,634837	no
XLOC_005293	g2739	120,122	100,818	-0,252752	-0,381595	0,4966	0,726443	no
XLOC_005294	g2740	22,3891	14,091	-0,668025	-0,958045	0,09475	0,294072	no
XLOC_005295	g2741	54,7654	36,5525	-0,583294	-0,982038	0,07425	0,25186	no
XLOC_005296	g2738	29,1792	35,2464	0,272536	0,464927	0,4071	0,660344	no
XLOC_005297	g2742	29,2436	11,9436	-1,29188	-2,10274	0,00045	0,00669545	yes
XLOC_005298	g2743	23,7157	22,6451	-0,0666425	-0,100812	0,85945	0,935634	no
XLOC_005299	g2745	45,3065	48,9096	0,110399	0,188289	0,74035	0,876968	no
XLOC_005300	g2746	55,1106	70,9925	0,365337	0,602645	0,29145	0,554336	no
XLOC_005301	g2747	2,73972	4,05285	0,564907	0,809117	0,16775	0,410902	no
XLOC_005302	g2744	23,0842	27,7764	0,266951	0,381878	0,49065	0,722873	no
XLOC_005303	g2748	7,58906	7,46707	-0,0233805	-0,0378045	0,9448	0,975496	no
XLOC_005304	g8360	0	0	0	0	1	1	no
XLOC_005305	g8361	9,80624	11,7555	0,26157	0,318253	0,56905	0,7772	no
XLOC_005306	g8362	0	0	0	0	1	1	no
XLOC_005307	g8363	0	0	0	0	1	1	no
XLOC_005308	g8364	0	0	0	0	1	1	no
XLOC_005309	g2749	307,165	274,904	-0,160088	-0,255402	0,6461	0,825616	no
XLOC_005310	g2750	0	0	0	0	1	1	no
XLOC_005311	g2751	25,1248	39,1431	0,639646	0,939449	0,09225	0,289423	no
XLOC_005312	g8365	0	0,455067	inf	0	1	1	no
XLOC_005313	g8366	0	0	0	0	1	1	no
XLOC_005314	g8367	0	0	0	0	1	1	no
XLOC_005315	g8370	1259,16	1921,63	0,609873	0,81359	0,1417	0,375978	no
XLOC_005316	g8368	9,00361	5,05635	-0,832408	-1,16796	0,0417	0,174624	no
XLOC_005317	g8369	12,2037	13,7144	0,168376	0,245672	0,6625	0,835141	no
XLOC_005318	g8371	0	0	0	0	1	1	no
XLOC_005319	g8372	0,1481	0,524869	1,82539	0	1	1	no
XLOC_005320	g8373	0	0,773368	inf	0	1	1	no
XLOC_005321	g2752	0	0	0	0	1	1	no
XLOC_005322	g8374	0	0	0	0	1	1	no
XLOC_005323	g8375	35,5502	26,4385	-0,427218	-0,565337	0,3343	0,594729	no
XLOC_005324	g8377	57,1243	41,9185	-0,446518	-0,660725	0,23735	0,495382	no
XLOC_005325	g8376	11,0791	4,5683	-1,27811	-1,46763	0,0144	0,086277	no
XLOC_005326	g8378	79,104	31,0184	-1,35063	-1,76229	0,00155	0,0171215	yes
XLOC_005327	g8379	46,9018	30,0473	-0,64241	-0,991187	0,0866	0,278073	no
XLOC_005328	g8380	1938,18	2665,78	0,459855	0,766944	0,16305	0,40515	no
XLOC_005329	g2753	10,0857	34,3348	1,76736	2,84635	5,00E-05	0,00120049	yes
XLOC_005330	g2754	7,41497	14,308	0,948314	1,35343	0,0161	0,0928919	no
XLOC_005331	g8381	225,189	249,068	0,145401	0,204617	0,7209	0,867979	no
XLOC_005332	g8383	14,2127	20,6123	0,536323	0,839256	0,14555	0,38186	no
XLOC_005333	g8382	29,1434	37,9705	0,381712	0,613719	0,2811	0,543386	no
XLOC_005334	g8384	4,97623	1,56361	-1,67018	-1,6481	0,0099	0,066	no
XLOC_005335	g8385	0	0	0	0	1	1	no

XLOC_005336	g8386	82,2467	53,2008	-0,62851	-1,05946	0,0767	0,256537	no
XLOC_005337	g8387	60,2422	85,426	0,5039	0,862304	0,1261	0,350199	no
XLOC_005338	g8388	153,412	126,154	-0,28222	-0,439093	0,436	0,683076	no
XLOC_005339	g8389	0,300955	1,27381	2,08153	1,34051	0,0585	0,216536	no
XLOC_005340	g8390	39,8622	56,896	0,513305	0,846015	0,12805	0,353415	no
XLOC_005341	g2755	4,38788	4,65137	0,084133	0,108507	0,8515	0,933294	no
XLOC_005342	g8391	24,5581	19,306	-0,347145	-0,414318	0,47505	0,712212	no
XLOC_005343	g8392	292,602	232,35	-0,33264	-0,470339	0,41855	0,670062	no
XLOC_005344	g2757	3,10892	5,79106	0,897411	0,508749	0,34935	0,610104	no
XLOC_005345	g2756	3,04642	2,77676	-0,133709	-0,139286	0,8072	0,911019	no
XLOC_005346	g8393	23,0034	46,2368	1,0072	1,30446	0,0276	0,134708	no
XLOC_005347	g8394	3,79611	2,52575	-0,58781	-0,715159	0,22095	0,477809	no
XLOC_005348	g8395	3,12235	0,974758	-1,67952	-1,49905	0,0387	0,166386	no
XLOC_005349	g8396	351,786	164,537	-1,09629	-1,75535	0,0025	0,0247231	yes
XLOC_005350	g8397	0,0998299	0,340848	1,77158	0	1	1	no
XLOC_005351	g392	0	0	0	0	1	1	no
XLOC_005352	g2758	165,767	111,633	-0,570398	-0,949407	0,0931	0,290512	no
XLOC_005353	g2760	12,0489	12,086	0,00442819	0,00657184	0,9904	0,99501	no
XLOC_005354	g2761	442,854	902,874	1,02769	1,5459	0,00605	0,0468911	yes
XLOC_005355	g2763	3,12495	2,82207	-0,147081	-0,191926	0,7329	0,873916	no
XLOC_005356	g2765	427,21	718,17	0,749379	0,88011	0,11765	0,336437	no
XLOC_005357	g2766	73,7672	46,2338	-0,67403	-1,16061	0,0454	0,184379	no
XLOC_005358	g2767	6,42768	3,86796	-0,732725	-0,78737	0,2662	0,527139	no
XLOC_005359	g2759	51,1518	62,6187	0,291809	0,488739	0,37945	0,636522	no
XLOC_005360	g2762	9,24817	12,5111	0,435975	0,515544	0,3717	0,630087	no
XLOC_005361	g2764	70,405	32,279	-1,12508	-1,55855	0,00955	0,0643217	no
XLOC_005362	g2768	17,2338	10,202	-0,756393	-1,14907	0,03415	0,15362	no
XLOC_005363	g2769	199,236	311,009	0,642477	1,03161	0,0639	0,229349	no
XLOC_005364	g2770	3,15029	2,00698	-0,650458	-0,733955	0,19375	0,44485	no
XLOC_005365	g8398	16,9941	26,9773	0,666711	0,7194	0,20505	0,458886	no
XLOC_005366	g8399	22,6588	12,1342	-0,90099	-1,33476	0,0226	0,11868	no
XLOC_005367	g8401	461,425	513,673	0,154754	0,237476	0,67175	0,840955	no
XLOC_005368	g8400	27,4863	33,2175	0,273233	0,455695	0,41425	0,666219	no
XLOC_005369	g8402	129,107	104,752	-0,30159	-0,453617	0,42125	0,671618	no
XLOC_005370	g8403	6,74629	5,29961	-0,348209	-0,43889	0,4381	0,684618	no
XLOC_005371	g8404	10,4591	4,65376	-1,16828	-1,06292	0,0729	0,249343	no
XLOC_005372	g8405	96,3802	64,1458	-0,587383	-0,961061	0,09095	0,286535	no
XLOC_005373	g8406	16,6325	25,3264	0,606634	0,796702	0,16785	0,410942	no
XLOC_005374	g8407	12,2085	10,4632	-0,222566	-0,313873	0,58365	0,784861	no
XLOC_005375	g8408	70,9382	79,3626	0,161895	0,280385	0,6261	0,812515	no
XLOC_005376	g8409	0	0	0	0	1	1	no
XLOC_005377	g8410	0,0819015	1,15989	3,82396	0	1	1	no
XLOC_005378	g8411	62,9249	28,3934	-1,14807	-1,8052	0,0017	0,0185283	yes
XLOC_005379	g8412	4,31747	4,57824	0,0846078	0,0964236	0,8674	0,939446	no
XLOC_005380	g8413	0	0	0	0	1	1	no
XLOC_005381	g8414	0	0	0	0	1	1	no
XLOC_005382	g2771	7,48825	6,00015	-0,319631	-0,469115	0,4161	0,6681	no
XLOC_005383	g2772	32,4695	36,9501	0,186496	0,28731	0,6116	0,804651	no
XLOC_005384	g8416	247,302	499,971	1,01557	1,66236	0,00335	0,0307449	yes
XLOC_005385	g8415	11,5417	23,4782	1,02447	1,2738	0,03825	0,165396	no
XLOC_005386	g2773	6,64561	8,86038	0,414968	0,556823	0,34635	0,606591	no
XLOC_005387	g8417	110,987	127,62	0,20147	0,332903	0,5613	0,77218	no
XLOC_005388	g8418	9,05148	16,5317	0,86901	1,19047	0,0416	0,174578	no
XLOC_005389	g8419	155,709	86,198	-0,853125	-1,06028	0,06405	0,229635	no
XLOC_005390	g8421	160,365	154,737	-0,0515444	-0,0837223	0,88275	0,947596	no
XLOC_005391	g8420	240,176	204,05	-0,235175	-0,397973	0,48245	0,716707	no
XLOC_005392	g8422	37,1397	46,8231	0,334259	0,531005	0,36515	0,624894	no
XLOC_005393	g2774	0	15,8335	inf	#NAME?	5,00E-05	0,00120049	yes
XLOC_005394	g8424	132,227	180,913	0,452278	0,775985	0,1727	0,418125	no
XLOC_005395	g8425	15,8541	1,8016	-3,13751	-3,62524	5,00E-05	0,00120049	yes
XLOC_005396	g8423	234,284	277,026	0,241762	0,383028	0,4971	0,726923	no
XLOC_005397	g8426	40,3395	48,6386	0,269908	0,422441	0,46215	0,703287	no
XLOC_005398	g8427	0	0	0	0	1	1	no
XLOC_005399	g8428	0	0	0	0	1	1	no
XLOC_005400	g8429	0	0	0	0	1	1	no
XLOC_005401	g8430	46,8885	22,3662	-1,06791	-1,30836	0,02715	0,133373	no
XLOC_005402	g394	10,0074	11,4489	0,194152	0,248568	0,65275	0,829667	no
XLOC_005403	g396	62,3127	70,693	0,182041	0,313748	0,58115	0,783336	no
XLOC_005404	g393	18,8334	22,437	0,252584	0,319226	0,57995	0,783024	no
XLOC_005405	g395	190,358	326,723	0,779352	1,30427	0,0242	0,123967	no
XLOC_005406	g397	17,6603	21,728	0,299049	0,433495	0,4381	0,684618	no
XLOC_005407	g8431	0,25696	0,809319	1,65516	0	1	1	no
XLOC_005408	g8432	0,0171522	0,233525	3,76711	0	1	1	no
XLOC_005409	g2775	19,4596	28,9451	0,572838	0,907317	0,1123	0,327528	no
XLOC_005410	g8433	0	0	0	0	1	1	no
XLOC_005411	g8434	22,294	15,6034	-0,514793	-0,828941	0,15005	0,387278	no
XLOC_005412	g2776	0	0	0	0	1	1	no
XLOC_005413	g8435	56,903	66,7501	0,230266	0,327531	0,57545	0,780604	no
XLOC_005414	g8436	0	0	0	0	1	1	no
XLOC_005415	g2777	3,91818	7,00045	0,837264	0,429179	0,2496	0,508092	no
XLOC_005416	g2778	5,12348	8,83084	0,785426	1,02984	0,06435	0,229955	no
XLOC_005417	g2779	18,9984	48,6315	1,35601	1,97563	0,00035	0,00552572	yes
XLOC_005418	g8437	390,954	393,23	0,00837503	0,0142805	0,97945	0,990233	no
XLOC_005419	g8438	72,3027	121,045	0,743418	1,19207	0,03935	0,168154	no
XLOC_005420	g8439	0	0	0	0	1	1	no
XLOC_005421	g8440	4,45714	4,12199	-0,112777	-0,142667	0,80675	0,910938	no
XLOC_005422	g8441	0	0	0	0	1	1	no
XLOC_005423	g8442	13,8192	10,8745	-0,345726	-0,505004	0,3828	0,639193	no
XLOC_005424	g8444	35,3884	44,5013	0,330571	0,483178	0,39835	0,65251	no
XLOC_005425	g8443	7,77683	11,5415	0,569576	0,722454	0,19915	0,451802	no
XLOC_005426	g8445	61,71	164,965	1,41858	2,09581	0,00025	0,00430702	yes
XLOC_005427	g8446	10,5725	7,67231	-0,462583	-0,635813	0,2651	0,526022	no

XLOC_005428	g8447	13,5702	9,64008	-0,493322	-0,607799	0,30245	0,564114	no
XLOC_005429	g8448	3,0937	4,89332	0,661481	0,824799	0,14375	0,379469	no
XLOC_005430	g8449	0	0	0	0	1	1	no
XLOC_005431	g8450	14,9677	11,4673	-0,384329	-0,582736	0,3087	0,569804	no
XLOC_005432	g8451	11,8765	18,1866	0,614767	0,752551	0,1679	0,410959	no
XLOC_005433	g8452	171,56	307,333	0,841092	1,22726	0,033	0,150446	no
XLOC_005434	g8456	30,2665	43,2373	0,514553	0,863564	0,1286	0,354275	no
XLOC_005435	g8453	14,9938	21,6839	0,532256	0,757177	0,19345	0,444265	no
XLOC_005436	g8454	34,1626	33,9531	-0,0088747	-0,0140195	0,98025	0,990233	no
XLOC_005437	g8455	98,0876	57,7917	-0,763209	-1,188	0,0414	0,173887	no
XLOC_005438	g8457	5,43623	9,13806	0,749281	1,07174	0,0651	0,231372	no
XLOC_005439	g8458	4,4925	3,82618	-0,231614	-0,405598	0,85875	0,935433	no
XLOC_005440	g8459	6,76612	5,38329	-0,32984	-0,318678	0,59315	0,791511	no
XLOC_005441	g8460	107,488	174,942	0,702702	1,1593	0,0429	0,177679	no
XLOC_005442	g8461	3,31778	3,93264	0,24528	0,313334	0,57475	0,780319	no
XLOC_005443	g8462	18,1225	17,4078	-0,0580523	-0,0861928	0,8877	0,950055	no
XLOC_005444	g8463	34,6819	55,1679	0,669644	0,871738	0,13	0,357191	no
XLOC_005445	g8464	31,3703	28,1643	-0,155534	-0,262341	0,63725	0,81962	no
XLOC_005446	g8465	19,4163	18,4278	-0,0753908	-0,0876266	0,88105	0,946806	no
XLOC_005447	g8466	7,96212	5,21248	-0,611183	-0,833574	0,143	0,378099	no
XLOC_005448	g8467	0	0	0	0	1	1	no
XLOC_005449	g8468	40,6333	27,1313	-0,582702	-0,818298	0,1553	0,392851	no
XLOC_005450	g8469	41,6775	21,706	-0,941177	-1,10476	0,2285	0,48674	no
XLOC_005451	g8470	51,2137	50,0769	-0,0323849	-0,0534233	0,9263	0,967431	no
XLOC_005452	g2780	180,449	26,4412	-2,77073	-3,91697	5,00E-05	0,00120049	yes
XLOC_005453	g8471	0	0	0	0	1	1	no
XLOC_005454	g8472	0	0	0	0	1	1	no
XLOC_005455	g8473	9,14577	7,11884	-0,361462	-0,47265	0,41295	0,665546	no
XLOC_005456	g8474	0	0	0	0	1	1	no
XLOC_005457	g8475	30,1649	28,6684	-0,0734069	-0,120177	0,83235	0,923058	no
XLOC_005458	g8476	0	0	0	0	1	1	no
XLOC_005459	g8477	0	0	0	0	1	1	no
XLOC_005460	g2782	29,3267	48,448	0,724221	1,1617	0,0446	0,182109	no
XLOC_005461	g2783	186,249	132,041	-0,496251	-0,717258	0,18975	0,439572	no
XLOC_005462	g2784	457,633	423,34	-0,112372	-0,192068	0,7395	0,876968	no
XLOC_005463	g2788	67,6635	106,894	0,659735	1,11503	0,0532	0,204792	no
XLOC_005464	g2781	32,5992	40,0125	0,295615	0,443404	0,43595	0,683076	no
XLOC_005465	g2785	200,322	55,474	-1,85244	-3,14811	5,00E-05	0,00120049	yes
XLOC_005466	g2786	40,0807	54,8134	0,451622	0,764106	0,19265	0,44305	no
XLOC_005467	g2787	40,6866	36,5104	-0,156244	-0,259396	0,6537	0,829883	no
XLOC_005468	g2790	19,4056	15,5496	-0,319589	-0,440939	0,43935	0,685481	no
XLOC_005469	g2789	70,138	61,3959	-0,192054	-0,306443	0,59085	0,790268	no
XLOC_005470	g2791	31,476	59,8563	0,927251	1,57684	0,007	0,0518011	no
XLOC_005471	g2792	19,5893	13,6274	-0,523556	-0,753478	0,1858	0,433695	no
XLOC_005472	g8478	24,6759	53,0683	1,10475	1,61094	0,00995	0,0662434	no
XLOC_005473	g8479	0	0	0	0	1	1	no
XLOC_005474	g8480	54,1062	59,6577	0,140914	0,205357	0,7262	0,869881	no
XLOC_005475	g8481	7,12375	6,44952	-0,143445	-0,192432	0,73985	0,876968	no
XLOC_005476	g8482	780,011	662,577	-0,235406	-0,38848	0,48375	0,717735	no
XLOC_005477	g8483	130,316	82,9085	-0,65242	-1,03561	0,06855	0,239644	no
XLOC_005478	g8484	0,391106	0,240898	-0,699139	0	1	1	no
XLOC_005479	g8485	38,6067	22,2216	-0,796888	-1,17749	0,03645	0,16008	no
XLOC_005480	g8486	0	0,0675604	inf	0	1	1	no
XLOC_005481	g2794	4,01964	1,53552	-1,38834	-1,8814	0,001	0,0126221	yes
XLOC_005482	g2793	0	0	0	0	1	1	no
XLOC_005483	g8487	71,706	84,4678	0,236307	0,264282	0,6335	0,816722	no
XLOC_005484	g2795	0	0	0	0	1	1	no
XLOC_005485	g8488	34,7723	35,9125	0,0465451	0,0786673	0,89235	0,951866	no
XLOC_005486	g8490	64,5061	103,234	0,678403	1,10651	0,05	0,196872	no
XLOC_005487	g8489	33,8686	51,1473	0,594707	0,979145	0,08165	0,268611	no
XLOC_005488	g8491	72,3824	59,4109	-0,284911	-0,467252	0,40125	0,655075	no
XLOC_005489	g2796	9,1282	11,4334	0,324849	0,430918	0,4605	0,701864	no
XLOC_005490	g2798	23,7551	53,069	1,15963	1,69784	0,00315	0,0293482	yes
XLOC_005491	g2797	8,31908	15,1282	0,862746	1,24163	0,03025	0,142404	no
XLOC_005492	g8492	0	0	0	0	1	1	no
XLOC_005493	g8493	13,6851	18,6551	0,446959	0,661853	0,2612	0,521008	no
XLOC_005494	g8494	7,10616	8,10453	0,189659	0,276937	0,6261	0,812515	no
XLOC_005495	g8495	18,0643	28,818	0,673828	1,01219	0,08165	0,268611	no
XLOC_005496	g8496	0	0	0	0	1	1	no
XLOC_005497	g8497	3,60334	3,0111	-0,259041	-0,276461	0,63895	0,820731	no
XLOC_005498	g398	0	0	0	0	1	1	no
XLOC_005499	g8498	2,18123	1,1929	-0,870669	-0,968777	0,10595	0,315568	no
XLOC_005500	g8499	14,4245	8,73546	-0,723568	-1,05797	0,07105	0,245155	no
XLOC_005501	g8500	0	0	0	0	1	1	no
XLOC_005502	g8501	39,393	57,2889	0,540318	0,695796	0,23585	0,493721	no
XLOC_005503	g8502	1499,63	2416,01	0,688018	0,883002	0,1044	0,312563	no
XLOC_005504	g8503	162,057	574,431	1,82563	2,71485	5,00E-05	0,00120049	yes
XLOC_005505	g2799	15,9823	17,4168	0,124006	0,193025	0,7344	0,874689	no
XLOC_005506	g2800	0,810984	0,435394	-0,897352	0	1	1	no
XLOC_005507	g2802	1892,4	5487,57	1,53595	2,03507	0,0008	0,0106019	yes
XLOC_005508	g2801	64,2107	74,9434	0,222987	0,361611	0,52355	0,746189	no
XLOC_005509	g2803	12,1948	45,975	1,91458	2,8567	5,00E-05	0,00120049	yes
XLOC_005510	g2804	44,1172	33,5835	-0,39359	-0,655497	0,2572	0,517138	no
XLOC_005511	g8504	2,20805	2,65081	0,263657	0,255092	0,65105	0,828686	no
XLOC_005512	g8505	110,259	119,941	0,121422	0,155863	0,791	0,903527	no
XLOC_005513	g8506	2,0256	2,1459	0,083234	0,063111	0,90455	0,958425	no
XLOC_005514	g8507	73,066	112,514	0,622833	1,01967	0,07045	0,24377	no
XLOC_005515	g8508	177,387	134,391	-0,400463	-0,570775	0,32015	0,580479	no
XLOC_005516	g8509	0	1,46688	inf	0	1	1	no
XLOC_005517	g8510	38,1499	35,3108	-0,111569	-0,162666	0,771	0,893264	no
XLOC_005518	g2805	68,3116	274,752	2,00793	1,84378	0,01115	0,0717987	no
XLOC_005519	g2807	306,832	536,226	0,805394	1,23943	0,0268	0,132582	no

XLOC_005520	g2808	49,4046	50,4279	0,0295775	0,0476311	0,93035	0,969135	no
XLOC_005521	g2809	52,8568	33,3687	-0,663596	-1,0723	0,0542	0,207099	no
XLOC_005522	g2806	42,8388	50,5985	0,240176	0,409401	0,4623	0,703407	no
XLOC_005523	g2810	40,9969	36,5736	-0,164711	-0,269145	0,6312	0,816117	no
XLOC_005524	g8511	235,792	211,409	-0,157474	-0,239224	0,6648	0,835806	no
XLOC_005525	g8512	14,2778	7,89232	-0,855252	-0,649659	0,25395	0,513231	no
XLOC_005526	g8513	85,4868	208,639	1,28723	2,10238	0,0002	0,00367103	yes
XLOC_005527	g8514	5,14812	4,33183	-0,24907	-0,272458	0,6394	0,820987	no
XLOC_005528	g8515	54,3014	52,6806	-0,0437168	-0,0725214	0,8996	0,955551	no
XLOC_005529	g2812	97,8756	113,36	0,211885	0,358896	0,5219	0,745347	no
XLOC_005530	g2814	81,9093	81,115	-0,0140588	-0,0218173	0,97125	0,986977	no
XLOC_005531	g2811	154,427	346,049	1,16405	1,84096	0,00175	0,0188639	yes
XLOC_005532	g2813	304,796	357,088	0,228436	0,361686	0,52615	0,747943	no
XLOC_005533	g8516	26,5582	14,2031	-0,902946	-1,46198	0,01285	0,0799158	no
XLOC_005534	g2815	24,7353	22,4743	-0,138297	-0,168387	0,77465	0,894738	no
XLOC_005535	g8517	0	0	0	0	1	1	no
XLOC_005536	g8518	0	0	0	0	1	1	no
XLOC_005537	g8519	0	0	0	0	1	1	no
XLOC_005538	g8520	25,3849	26,1108	0,0406735	0,0658013	0,91145	0,962529	no
XLOC_005539	g8521	41,5967	39,2289	-0,08455	-0,134127	0,8101	0,912557	no
XLOC_005540	g8522	0	0	0	0	1	1	no
XLOC_005541	g8523	0	0	0	0	1	1	no
XLOC_005542	g8524	2,80315	2,37043	-0,241899	-0,225179	0,68515	0,848738	no
XLOC_005543	g8525	0	0	0	0	1	1	no
XLOC_005544	g399	80,757	66,9308	-0,270916	-0,453249	0,429	0,677514	no
XLOC_005545	g8526	52,4913	42,9841	-0,288278	-0,412332	0,4809	0,715629	no
XLOC_005546	g8527	0	0	0	0	1	1	no
XLOC_005547	g8528	18,6599	19,1	0,0336328	0,0455958	0,93765	0,972153	no
XLOC_005548	g8529	366,647	83,4751	-2,13497	-2,68147	0,0002	0,00367103	yes
XLOC_005549	g8530	0	0	0	0	1	1	no
XLOC_005550	g8531	0	0	0	0	1	1	no
XLOC_005551	g8532	0	0	0	0	1	1	no
XLOC_005552	g8533	0	0	0	0	1	1	no
XLOC_005553	g8534	15,9319	19,5526	0,295446	0,389707	0,5034	0,731595	no
XLOC_005554	g8535	21,8606	15,7063	-0,47699	-0,604916	0,28785	0,551119	no
XLOC_005555	g8536	62,6367	48,0245	-0,383238	-0,631785	0,2758	0,537479	no
XLOC_005556	g8537	9,81116	12,7004	0,372381	0,573993	0,31045	0,571193	no
XLOC_005557	g8539	30,2784	36,017	0,250389	0,407624	0,47675	0,712986	no
XLOC_005558	g8540	75,2799	56,5795	-0,411985	-0,682522	0,23015	0,487612	no
XLOC_005559	g8538	6,81201	21,3616	1,64887	2,18797	0,00035	0,00552572	yes
XLOC_005560	g8541	38,777	32,35	-0,261434	-0,364506	0,50595	0,733528	no
XLOC_005561	g2818	177,211	248,084	0,485357	0,765953	0,17775	0,424594	no
XLOC_005562	g2820	94,901	72,8933	-0,380639	-0,608529	0,2846	0,546579	no
XLOC_005563	g2822	66,6343	68,2054	0,0336196	0,057537	0,9153	0,963526	no
XLOC_005564	g2824	130,873	169,381	0,372108	0,620036	0,28355	0,546283	no
XLOC_005565	g2816	62,3522	58,8056	-0,0844886	-0,134171	0,81895	0,917418	no
XLOC_005566	g2817	245,094	1034,24	2,07717	2,94829	5,00E-05	0,00120049	yes
XLOC_005567	g2819	21,767	59,4583	1,44973	2,21985	5,00E-05	0,00120049	yes
XLOC_005568	g2821	139,07	207,888	0,579995	0,983899	0,0878	0,280025	no
XLOC_005569	g2823	75,5475	68,5397	-0,140443	-0,232633	0,6853	0,848738	no
XLOC_005570	g8542	29,5957	28,0888	-0,0753893	-0,120227	0,8302	0,922236	no
XLOC_005571	g8543	251,016	111,207	-1,17453	-1,8327	0,00125	0,0146655	yes
XLOC_005572	g8544	0	0	0	0	1	1	no
XLOC_005573	g8545	0,18242	0,314121	0,784057	0	1	1	no
XLOC_005574	g8546	164,953	285,789	0,792895	1,0665	0,07305	0,249427	no
XLOC_005575	g8547	5,69587	6,10818	0,100826	0,113228	0,8403	0,926642	no
XLOC_005576	g8548	0	0,064272	inf	0	1	1	no
XLOC_005577	g8549	26,1502	5,31015	-2,3	-2,06115	0,23795	0,496002	no
XLOC_005578	g8551	4,22272	2,84261	-0,570953	-0,588053	0,31755	0,578204	no
XLOC_005579	g8550	83,4193	72,2135	-0,208113	-0,29183	0,5964	0,793476	no
XLOC_005580	g2825	51,1109	38,5719	-0,406082	-0,657832	0,2405	0,498883	no
XLOC_005581	g2826	20,7639	8,75659	-1,24564	-1,66443	0,00505	0,0412571	yes
XLOC_005582	g2831	19,33	27,2005	0,492795	0,722242	0,2067	0,461212	no
XLOC_005583	g2827	7,50024	7,35068	-0,0290583	-0,0361661	0,94955	0,977215	no
XLOC_005584	g2828	8,93303	11,6263	0,380166	0,555209	0,3286	0,5899	no
XLOC_005585	g2829	7,67938	16,7957	1,12903	1,55973	0,00695	0,0515476	no
XLOC_005586	g2830	263,977	473,146	0,841873	1,40684	0,013	0,0805426	no
XLOC_005587	g2832	0	0	0	0	1	1	no
XLOC_005588	g8552	33,1956	35,0744	0,0794268	0,0638574	0,9223	0,965872	no
XLOC_005589	g2833	0,618711	11,9718	4,27424	3,04015	0,2446	0,503031	no
XLOC_005590	g8553	17,9276	10,5711	-0,762049	-0,902048	0,1185	0,337981	no
XLOC_005591	g8554	20,678	20,1679	-0,0360302	-0,0435937	0,9423	0,974708	no
XLOC_005592	g8555	0	0	0	0	1	1	no
XLOC_005593	g8556	15,2341	10,6008	-0,523132	-0,579663	0,32985	0,590866	no
XLOC_005594	g2834	27,0481	14,4934	-0,900135	-1,36407	0,01885	0,10411	no
XLOC_005595	g2835	9,71251	15,0458	0,631449	0,928089	0,0955	0,295373	no
XLOC_005596	g2836	33,8112	27,992	-0,272491	-0,445666	0,42615	0,675809	no
XLOC_005597	g8557	12,8888	4,03949	-1,67388	-2,41321	5,00E-05	0,00120049	yes
XLOC_005598	g8558	24,2472	19,0267	-0,349792	-0,488076	0,39635	0,650536	no
XLOC_005599	g8559	43,8054	34,6669	-0,33755	-0,541432	0,339	0,599061	no
XLOC_005600	g8561	53,6057	49,7752	-0,106959	-0,186371	0,7451	0,880033	no
XLOC_005601	g8560	61,7293	52,5752	-0,231572	-0,397157	0,49215	0,723707	no
XLOC_005602	g8562	25,4694	8,28778	-1,61971	-2,32019	0,00015	0,00294012	yes
XLOC_005603	g8563	38,4789	59,8041	0,636177	0,933925	0,108	0,319542	no
XLOC_005604	g8564	211,833	244,797	0,208657	0,349851	0,54105	0,759667	no
XLOC_005605	g2837	0	0	0	0	1	1	no
XLOC_005606	g8565	22,7166	23,8376	0,0694895	0,102488	0,8604	0,935634	no
XLOC_005607	g8566	121,788	99,8512	-0,286514	-0,455976	0,41735	0,669451	no
XLOC_005608	g8567	59,4118	75,8374	0,35216	0,544126	0,33295	0,593819	no
XLOC_005609	g8568	0	0	0	0	1	1	no
XLOC_005610	g8569	0	0	0	0	1	1	no
XLOC_005611	g8570	59,6789	64,2955	0,107497	0,185623	0,74905	0,881846	no

XLOC_005612	g2838	0	0	0	0	1	1	no
XLOC_005613	g8571	0	0	0	0	1	1	no
XLOC_005614	g8573	0	0	0	0	1	1	no
XLOC_005615	g8572	0	0	0	0	1	1	no
XLOC_005616	g8574	0	0	0	0	1	1	no
XLOC_005617	g8575	0	0	0	0	1	1	no
XLOC_005618	g8576	0	0	0	0	1	1	no
XLOC_005619	g8577	0	0	0	0	1	1	no
XLOC_005620	g8578	3,0286	3,50633	0,211309	0,25617	0,64985	0,827695	no
XLOC_005621	g8579	4,1411	3,54611	-0,223775	-0,308474	0,59135	0,790276	no
XLOC_005622	g8580	28,8613	40,7189	0,496565	0,696915	0,2206	0,477472	no
XLOC_005623	g8581	0	0	0	0	1	1	no
XLOC_005624	g8582	38,1011	56,074	0,5575	0,774911	0,1741	0,420013	no
XLOC_005625	g8583	0	0	0	0	1	1	no
XLOC_005626	g8584	52,5639	56,9905	0,116649	0,184323	0,74815	0,881846	no
XLOC_005627	g8585	31,9596	32,5239	0,0252532	0,041585	0,9449	0,975496	no
XLOC_005628	g8586	0	0	0	0	1	1	no
XLOC_005629	g8587	35,766	50,8309	0,507116	0,688662	0,2339	0,491315	no
XLOC_005630	g8588	0,570324	0,59173	0,0531555	0	1	1	no
XLOC_005631	g8589	184,092	104,887	-0,811597	-1,24408	0,03255	0,148878	no
XLOC_005632	g8590	0	0	0	0	1	1	no
XLOC_005633	g8591	1,06338	1,50332	0,499495	0,590013	0,29925	0,561558	no
XLOC_005634	g2839	18,8668	32,5722	0,787789	1,26825	0,02965	0,140863	no
XLOC_005635	g2840	692,186	1351,07	0,964869	1,08177	0,0643	0,229955	no
XLOC_005636	g8592	112,452	122,012	0,117717	0,202343	0,7221	0,868678	no
XLOC_005637	g8594	31,2144	34,046	0,125276	0,216477	0,7069	0,860848	no
XLOC_005638	g8596	735,154	1328,18	0,853333	1,34535	0,02195	0,11645	no
XLOC_005639	g8593	53,5014	57,4387	0,102447	0,17722	0,7544	0,884245	no
XLOC_005640	g8595	85,645	71,5459	-0,259501	-0,441076	0,42515	0,67458	no
XLOC_005641	g8597	6,40771	12,3851	0,950728	1,05866	0,0734	0,250274	no
XLOC_005642	g8600	20,5515	22,2621	0,115347	0,171549	0,77525	0,894905	no
XLOC_005643	g8598	28,0805	26,6086	-0,0776777	-0,127308	0,8229	0,919278	no
XLOC_005644	g8599	157,847	131,05	-0,268417	-0,37269	0,51085	0,736969	no
XLOC_005645	g2841	111,758	630,262	2,49558	4,05433	5,00E-05	0,00120049	yes
XLOC_005646	g2842	31,4397	30,4281	-0,0471835	-0,0777074	0,89275	0,951982	no
XLOC_005647	g2843	36,2674	33,2205	-0,126603	-0,219354	0,70425	0,859387	no
XLOC_005648	g8601	26,743	73,4621	1,45784	2,22056	0,00015	0,00294012	yes
XLOC_005649	g8602	6,10953	8,19143	0,423054	0,479444	0,4131	0,665649	no
XLOC_005650	g2844	46,6	30,39	-0,616736	-0,795799	0,16925	0,41262	no
XLOC_005651	g2845	27,3144	21,9623	-0,314627	-0,504164	0,3744	0,632807	no
XLOC_005652	g8603	20,2449	81,439	2,00816	2,58913	0,00025	0,00430702	yes
XLOC_005653	g8604	7,02593	7,34509	0,0640916	0,0942473	0,87125	0,942359	no
XLOC_005654	g8605	27,801	29,6152	0,0912056	0,114744	0,84065	0,926771	no
XLOC_005655	g8606	0,0224421	0,0407468	0,860479	0	1	1	no
XLOC_005656	g8607	53,2906	63,2642	0,247508	0,406341	0,4714	0,709122	no
XLOC_005657	g8608	102,653	87,8613	-0,224478	-0,378828	0,5083	0,734874	no
XLOC_005658	g2846	0	0	0	0	1	1	no
XLOC_005659	g2847	0	0	0	0	1	1	no
XLOC_005660	g2848	8,9774	13,0332	0,537826	0,698151	0,21765	0,474223	no
XLOC_005661	g8609	0,289962	0,591187	1,02775	0	1	1	no
XLOC_005662	g2852	225,145	123,516	-0,866157	-1,22582	0,02985	0,141402	no
XLOC_005663	g2849	50,1206	28,8275	-0,797958	-1,23333	0,0266	0,131992	no
XLOC_005664	g2850	119,212	60,3197	-0,982823	-1,44058	0,0134	0,0820886	no
XLOC_005665	g2851	74,8211	73,5327	-0,0250575	-0,0421125	0,94155	0,974188	no
XLOC_005666	g8610	2,17131	2,70535	0,31725	0,338401	0,56595	0,774906	no
XLOC_005667	g8611	9,06666	9,17363	0,0169214	0,0212313	0,9717	0,986977	no
XLOC_005668	g8613	8,31304	7,22514	-0,20235	-0,265149	0,64015	0,821262	no
XLOC_005669	g8614	257,291	351,09	0,44844	0,733026	0,19175	0,442199	no
XLOC_005670	g8612	2,20973	4,46325	1,01423	1,16583	0,0473	0,189046	no
XLOC_005671	g8615	49,1286	25,655	-0,937321	-1,27745	0,027	0,133036	no
XLOC_005672	g8616	29,9741	26,5024	-0,177591	-0,271164	0,64055	0,821582	no
XLOC_005673	g2853	17,1412	32,8394	0,937959	1,60732	0,0054	0,0429724	yes
XLOC_005674	g2855	27,2265	46,1584	0,761583	1,15341	0,0466	0,187392	no
XLOC_005675	g2854	12,5875	14,4047	0,194545	0,286777	0,6139	0,805977	no
XLOC_005676	g2856	1,23982	3,47917	1,48862	1,02729	0,0658	0,232933	no
XLOC_005677	g8617	38,8972	40,3888	0,0542906	0,0902304	0,8716	0,942376	no
XLOC_005678	g8618	11,7705	8,8526	-0,411005	-0,3901	0,50725	0,734473	no
XLOC_005679	g8619	236,702	85,8023	-1,46398	-2,46706	5,00E-05	0,00120049	yes
XLOC_005680	g8620	17,2109	0,96458	-4,15728	-3,53953	0,00045	0,00669545	yes
XLOC_005681	g2857	138,381	78,715	-0,813939	-1,25152	0,02455	0,124848	no
XLOC_005682	g8621	0	0	0	0	1	1	no
XLOC_005683	g8622	2,56232	3,8095	0,572152	0,574945	0,31455	0,575318	no
XLOC_005684	g8623	13,529	6,80589	-0,991201	-1,19886	0,05305	0,204615	no
XLOC_005685	g8624	0	0,259047	inf	0	1	1	no
XLOC_005686	g8625	0	0	0	0	1	1	no
XLOC_005687	g2858	44,434	27,6887	-0,682363	-1,116	0,04655	0,187268	no
XLOC_005688	g2859	0,54651	0,395309	-0,467267	0	1	1	no
XLOC_005689	g2860	33,8525	32,2212	-0,0712528	-0,122744	0,8288	0,921619	no
XLOC_005690	g8626	4,64922	7,40158	0,670844	0,744761	0,20625	0,460627	no
XLOC_005691	g2861	50,0961	59,581	0,250155	0,424398	0,44905	0,694436	no
XLOC_005692	g8627	0	0	0	0	1	1	no
XLOC_005693	g8628	0	0	0	0	1	1	no
XLOC_005694	g8629	18,1028	15,0115	-0,270144	-0,362199	0,5312	0,75164	no
XLOC_005695	g8630	0	0	0	0	1	1	no
XLOC_005696	g8631	9,01638	19,8397	1,13777	1,6632	0,0033	0,0304282	yes
XLOC_005697	g8632	21,6523	24,6064	0,184515	0,286936	0,6129	0,805282	no
XLOC_005698	g8633	0	0	0	0	1	1	no
XLOC_005699	g8634	0	0	0	0	1	1	no
XLOC_005700	g8635	25,1833	1,24627	-4,33677	-6,20522	5,00E-05	0,00120049	yes
XLOC_005701	g8637	117,802	130,397	0,146549	0,231623	0,68615	0,849255	no
XLOC_005702	g8639	20,6936	23,5958	0,189349	0,316611	0,5744	0,780167	no
XLOC_005703	g8641	46,9642	37,0253	-0,343051	-0,578898	0,30685	0,567991	no

XLOC_005704	g8642	99,4885	118,733	0,255121	0,441539	0,4461	0,692382	no
XLOC_005705	g8644,g8645	122,232	107,207	-0,189218	-0,285701	0,62845	0,813951	no
XLOC_005706	g8647	72,1729	39,2081	-0,880305	-1,42623	0,013	0,0805426	no
XLOC_005707	g8649	62,5062	38,3218	-0,705833	-1,1527	0,0434	0,179071	no
XLOC_005708	g8650	454,522	476,645	0,0685641	0,0804574	0,88325	0,947925	no
XLOC_005709	g8653	242,236	327,275	0,43409	0,705534	0,21515	0,470656	no
XLOC_005710	g8655	163,64	151,186	-0,114205	-0,173632	0,75745	0,8854	no
XLOC_005711	g8636	4,48864	7,02002	0,645195	0,783155	0,16135	0,402965	no
XLOC_005712	g8638	64,7877	58,9841	-0,135393	-0,227459	0,69445	0,854574	no
XLOC_005713	g8640	473,199	370,837	-0,351664	-0,540582	0,3276	0,588769	no
XLOC_005714	g8643	138,554	177,409	0,356638	0,588154	0,30875	0,569804	no
XLOC_005715	g8646	92,1325	112,176	0,283982	0,423676	0,46745	0,707046	no
XLOC_005716	g8648	106,442	63,7814	-0,738861	-1,23994	0,0335	0,151669	no
XLOC_005717	g8651	6,60866	1,52761	-2,11308	-2,6266	5,00E-05	0,00120049	yes
XLOC_005718	g8652	878,087	1207,13	0,459145	0,663883	0,2545	0,513708	no
XLOC_005719	g8654	18,7717	12,8499	-0,546808	-0,712715	0,2236	0,480997	no
XLOC_005720	g8656	41,1283	28,1664	-0,546157	-0,91525	0,11445	0,331925	no
XLOC_005721	g8657	258,269	430,852	0,738318	0,714898	0,22875	0,486955	no
XLOC_005722	g8658	23,0847	36,9539	0,67879	1,04776	0,06745	0,237235	no
XLOC_005723	g8659	6,45977	2,07849	-1,63595	-1,12987	0,3343	0,594729	no
XLOC_005724	g2862	0,0860148	0,156137	0,860151	0	1	1	no
XLOC_005725	g8660	25,4221	1280,88	5,65491	6,53091	5,00E-05	0,00120049	yes
XLOC_005726	g8661	9,60047	28,0737	1,54804	1,68486	0,00475	0,0395632	yes
XLOC_005727	g8662	1,82524	2,1914	0,263766	0,18454	0,73645	0,875431	no
XLOC_005728	g8663	0	0	0	0	1	1	no
XLOC_005729	g8664	17,8996	19,9259	0,154712	0,197392	0,73485	0,87486	no
XLOC_005730	g8665	380,805	603,196	0,663577	0,868835	0,14805	0,38482	no
XLOC_005731	g8667	17,5839	24,1154	0,455694	0,252655	0,63995	0,82122	no
XLOC_005732	g8666	414,304	946,345	1,19168	1,61562	0,006	0,0465771	yes
XLOC_005733	g8668	0	0	0	0	1	1	no
XLOC_005734	g2863	13,1256	15,3494	0,225799	0,325245	0,563	0,772944	no
XLOC_005735	g2864	19,05	27,6791	0,539004	0,878953	0,1353	0,365817	no
XLOC_005736	g8669	0	0	0	0	1	1	no
XLOC_005737	g8670	0	0	0	0	1	1	no
XLOC_005738	g2866	15,3178	11,9226	-0,361514	-0,613958	0,2695	0,530883	no
XLOC_005739	g2868	25,8452	37,5601	0,539303	0,697019	0,238	0,496002	no
XLOC_005740	g2865	95,8349	78,6701	-0,284736	-0,49265	0,3934	0,64816	no
XLOC_005741	g2867	93,5466	85,0851	-0,136779	-0,174605	0,756	0,884596	no
XLOC_005742	g2869	24,255	42,4229	0,806562	1,35677	0,01845	0,102593	no
XLOC_005743	g8671	77,5096	91,3211	0,236574	0,408659	0,47525	0,712403	no
XLOC_005744	g2871	51,4162	165,22	1,68409	2,49605	5,00E-05	0,00120049	yes
XLOC_005745	g2870	242,051	447,962	0,888061	1,19339	0,03895	0,16688	no
XLOC_005746	g8672	0	0	0	0	1	1	no
XLOC_005747	g8673	0	0	0	0	1	1	no
XLOC_005748	g8674	0	0	0	0	1	1	no
XLOC_005749	g8675	10,3781	10,0489	-0,0464971	-0,0645092	0,90885	0,961172	no
XLOC_005750	g8678	12,1633	15,9734	0,393139	0,584216	0,3026	0,564286	no
XLOC_005751	g8679	23,059	18,6867	-0,303318	-0,515375	0,36165	0,621784	no
XLOC_005752	g8676	33,5279	43,3858	0,371859	0,62543	0,27305	0,53424	no
XLOC_005753	g8677	17,8885	18,5554	0,0528054	0,0867536	0,8778	0,945394	no
XLOC_005754	g8680	36,0552	22,5523	-0,67693	-1,02806	0,0787	0,261524	no
XLOC_005755	g8681	16,9505	14,0785	-0,267839	-0,353598	0,53935	0,758039	no
XLOC_005756	g8682	0	0	0	0	1	1	no
XLOC_005757	g8683	8,52854	15,5597	0,867441	0,785396	0,26445	0,52505	no
XLOC_005758	g8684	4,47795	2,29963	-0,961435	-1,20943	0,0377	0,163884	no
XLOC_005759	g2872	54,3284	33,1159	-0,714181	-1,04446	0,0746	0,252089	no
XLOC_005760	g2873	9,61407	9,91727	0,0447953	0,053953	0,92755	0,967861	no
XLOC_005761	g8685	0	0	0	0	1	1	no
XLOC_005762	g8686	0	0	0	0	1	1	no
XLOC_005763	g8687	0	0	0	0	1	1	no
XLOC_005764	g8688	0	0	0	0	1	1	no
XLOC_005765	g8689	2,49319	4,55488	0,86942	0,628576	0,2876	0,550747	no
XLOC_005766	g8690	0	0	0	0	1	1	no
XLOC_005767	g2875	148,266	46,3628	-1,67715	-1,94432	0,00495	0,0407111	yes
XLOC_005768	g2877	83,2408	91,8951	0,142697	0,244633	0,66705	0,837544	no
XLOC_005769	g2880	129,813	202,171	0,639144	1,05384	0,0579	0,215452	no
XLOC_005770	g2874	11,1989	25,358	1,17908	1,71726	0,0039	0,0342252	yes
XLOC_005771	g2876	92,5039	103,159	0,157278	0,27223	0,6359	0,818312	no
XLOC_005772	g2878	100,071	213,916	1,09602	1,61671	0,00535	0,0426783	yes
XLOC_005773	g2879	284,206	308,589	0,11875	0,186685	0,74245	0,878159	no
XLOC_005774	g8691	58,0697	75,1152	0,371318	0,489176	0,40475	0,658381	no
XLOC_005775	g2881	24,4925	62,3701	1,34851	1,88599	0,00195	0,0205021	yes
XLOC_005776	g8692	13,3783	11,0475	-0,276175	-0,400027	0,488	0,721133	no
XLOC_005777	g2883	0,339759	0,368284	0,11631	0	1	1	no
XLOC_005778	g2882	1,91456	2,00059	0,0634165	0,0457836	0,9353	0,971715	no
XLOC_005779	g2884	27,9862	19,7277	-0,50449	-0,84723	0,1382	0,370596	no
XLOC_005780	g2885	15,4366	14,6074	-0,0796561	-0,113258	0,84545	0,929972	no
XLOC_005781	g2886	110,56	155,063	0,488022	0,784534	0,1525	0,390394	no
XLOC_005782	g8693	66,7826	36,2756	-0,880473	-1,30973	0,02175	0,11589	no
XLOC_005783	g8694	203,931	140,082	-0,541811	-0,935569	0,1034	0,310232	no
XLOC_005784	g8695	0	0	0	0	1	1	no
XLOC_005785	g8696	0	0	0	0	1	1	no
XLOC_005786	g8697	0	0	0	0	1	1	no
XLOC_005787	g8698	0,257131	1,07269	2,06066	1,22296	0,0458	0,185237	no
XLOC_005788	g8699	0	0	0	0	1	1	no
XLOC_005789	g8700	0	0,0503422	inf	0	1	1	no
XLOC_005790	g8702	79,8994	52,5061	-0,605699	-0,921933	0,0956	0,295496	no
XLOC_005791	g8701	61,4549	60,7446	-0,0167698	-0,0264424	0,96265	0,983635	no
XLOC_005792	g2887	0	0	0	0	1	1	no
XLOC_005793	g8703	12,7659	9,41576	-0,43915	-0,595027	0,2873	0,55028	no
XLOC_005794	g2889	25,8228	24,8621	-0,0546992	-0,0642337	0,9099	0,96181	no
XLOC_005795	g2890	96,9137	468,101	2,27205	3,61288	5,00E-05	0,00120049	yes

XLOC_005796	g2888	5663,49	1039,31	-2,44607	-2,15772	0,00025	0,00430702	yes
XLOC_005797	g2891	12,7676	76,9251	2,59097	3,43059	5,00E-05	0,00120049	yes
XLOC_005798	g8704	0	0	0	0	1	1	no
XLOC_005799	g2894	1,61784	4,63118	1,51731	1,78653	0,0046	0,0386416	yes
XLOC_005800	g2892	117,058	159,433	0,445732	0,755472	0,1798	0,426853	no
XLOC_005801	g2893	1,36279	2,83834	1,05848	1,10618	0,06075	0,221689	no
XLOC_005802	g2895	1,95612	4,41003	1,17279	1,4228	0,01685	0,0959229	no
XLOC_005803	g2896	3,88093	1,91624	-1,01813	-1,4229	0,01485	0,0880066	no
XLOC_005804	g2897	66,056	63,3379	-0,0606212	-0,104068	0,8517	0,933294	no
XLOC_005805	g2899	8,22797	33,1761	2,01154	3,11432	5,00E-05	0,00120049	yes
XLOC_005806	g2901	68,0773	88,194	0,373505	0,614846	0,27125	0,532735	no
XLOC_005807	g2898	16,4211	20,7163	0,335217	0,505751	0,37185	0,630232	no
XLOC_005808	g2900	39,8856	57,6584	0,531663	0,912547	0,1104	0,32396	no
XLOC_005809	g2902	24,8232	23,5744	-0,0744697	-0,128885	0,82315	0,919396	no
XLOC_005810	g2903	93,7346	77,7923	-0,268953	-0,439427	0,4277	0,677203	no
XLOC_005811	g8705	36,8874	49,9747	0,438072	0,533709	0,35045	0,611135	no
XLOC_005812	g8706	5,55424	7,82293	0,49412	0,637237	0,2761	0,53785	no
XLOC_005813	g400	86,8776	113,4	0,384368	0,639189	0,2646	0,525242	no
XLOC_005814	g403	165,637	171,905	0,0535794	0,0884925	0,8793	0,945715	no
XLOC_005815	g401	46,8482	45,7503	-0,0342143	-0,0539883	0,92555	0,967112	no
XLOC_005816	g402	16,5159	12,2019	-0,436755	-0,717048	0,20635	0,460745	no
XLOC_005817	g8707	0	0	0	0	1	1	no
XLOC_005818	g8708	16,7502	13,0744	-0,357434	-0,572734	0,31255	0,573414	no
XLOC_005819	g8709	147,114	108,156	-0,443817	-0,643377	0,26125	0,521008	no
XLOC_005820	g8710	0	0	0	0	1	1	no
XLOC_005821	g2906	18,6798	15,6384	-0,256384	-0,301603	0,59765	0,794601	no
XLOC_005822	g2904	252,274	254,544	0,0129224	0,0214124	0,96905	0,98653	no
XLOC_005823	g2905	94,1075	93,9695	-0,00211733	-0,00347633	0,99435	0,996888	no
XLOC_005824	g8711	6,98443	6,37565	-0,131571	-0,136939	0,81495	0,915968	no
XLOC_005825	g8712	13,8011	23,6922	0,779632	1,05642	0,0601	0,220546	no
XLOC_005826	g8713	9,55786	17,5577	0,877347	0,64155	0,25015	0,508587	no
XLOC_005827	g8714	0	0	0	0	1	1	no
XLOC_005828	g8715	0	0	0	0	1	1	no
XLOC_005829	g8716	0	0	0	0	1	1	no
XLOC_005830	g8717	289,565	11772	5,34533	6,34646	5,00E-05	0,00120049	yes
XLOC_005831	g8719	19,6993	25,9635	0,398338	0,528488	0,36655	0,626221	no
XLOC_005832	g8718	39,8039	58,0426	0,544203	0,825319	0,15235	0,390111	no
XLOC_005833	g8721	14,644	32,5831	1,15381	1,75733	0,00315	0,0293482	yes
XLOC_005834	g8720	23,7597	31,5904	0,410972	0,693707	0,2296	0,487604	no
XLOC_005835	g8722	2,65112	4,63828	0,806987	0,973578	0,093	0,290512	no
XLOC_005836	g2907	0	0	0	0	1	1	no
XLOC_005837	g8723	0	0	0	0	1	1	no
XLOC_005838	g8724	0	0	0	0	1	1	no
XLOC_005839	g2908	5,60294	6,69812	0,257572	0,272359	0,63585	0,818312	no
XLOC_005840	g2909	34,7469	25,7015	-0,435032	-0,742361	0,18555	0,433654	no
XLOC_005841	g8725	40,2394	35,1186	-0,196373	-0,298095	0,5907	0,790268	no
XLOC_005842	g8726	12,9358	14,2691	0,141516	0,199239	0,7252	0,869807	no
XLOC_005843	g8727	7,53519	20,438	1,43954	1,7918	0,0021	0,0216618	yes
XLOC_005844	g8728	0	0	0	0	1	1	no
XLOC_005845	g8729	0	0	0	0	1	1	no
XLOC_005846	g8730	0	0	0	0	1	1	no
XLOC_005847	g8731	0,680099	0,272962	-1,31704	0	1	1	no
XLOC_005848	g2910	214,709	730,734	1,76696	2,09265	0,0011	0,0134856	yes
XLOC_005849	g2911	80,1421	48,6224	-0,720941	-1,22633	0,03145	0,145541	no
XLOC_005850	g8732	0	0	0	0	1	1	no
XLOC_005851	g2913	6,99121	5,52304	-0,340079	-0,496989	0,3783	0,636208	no
XLOC_005852	g2912	49,4813	44,1361	-0,164923	-0,277912	0,6283	0,813864	no
XLOC_005853	g8734	98,8583	79,7823	-0,309294	-0,5588	0,3475	0,607846	no
XLOC_005854	g8733	245,338	329,088	0,423699	0,684435	0,22965	0,487604	no
XLOC_005855	g2914	0	0	0	0	1	1	no
XLOC_005856	g8735	0	0,151151	inf	0	1	1	no
XLOC_005857	g8736	0	0	0	0	1	1	no
XLOC_005858	g8737	1,35624	1,39184	0,0373781	0,0366853	0,9607	0,982256	no
XLOC_005859	g2915	0	0	0	0	1	1	no
XLOC_005860	g8738	14,4013	10,8213	-0,412323	-0,533468	0,33	0,590901	no
XLOC_005861	g2916	0,0308174	0,0279258	-0,14215	0	1	1	no
XLOC_005862	g8739	13,0608	5,08283	-1,36154	-2,98125	0,41385	0,666012	no
XLOC_005863	g8740	96,316	88,8536	-0,116345	-0,191912	0,7266	0,869936	no
XLOC_005864	g8741	0,0368137	0	#NAME?	0	1	1	no
XLOC_005865	g8742	0	0	0	0	1	1	no
XLOC_005866	g8743	30,5983	9,93815	-1,6224	-2,41032	5,00E-05	0,00120049	yes
XLOC_005867	g8744	12,7189	8,65932	-0,554653	-0,734957	0,1955	0,446987	no
XLOC_005868	g8745	0	0,0528895	inf	0	1	1	no
XLOC_005869	g8746	0	0	0	0	1	1	no
XLOC_005870	g25	0	1,68028	inf	0	1	1	no
XLOC_005871	g8747	15,7173	14,083	-0,1584	-0,234511	0,6888	0,850713	no
XLOC_005872	g8748	4,79325	3,3251	-0,527604	-0,478118	0,3974	0,651388	no
XLOC_005873	g2917	55,8763	72,9952	0,385564	0,663419	0,24835	0,507023	no
XLOC_005874	g8749	60,3444	39,4714	-0,612413	-1,03897	0,07445	0,252007	no
XLOC_005875	g8750	33,1208	11,652	-1,50716	-1,93245	0,0012	0,0143358	yes
XLOC_005876	g2918	0	0	0	0	1	1	no
XLOC_005877	g8751	30,9901	33,3397	0,105437	0,144271	0,79655	0,906252	no
XLOC_005878	g8753	64,2788	50,1697	-0,357526	-0,599952	0,2908	0,553529	no
XLOC_005879	g8752	24,0261	13,837	-0,79607	-1,23763	0,02615	0,13022	no
XLOC_005880	g2919	30,6809	31,5828	0,0417955	0,0717865	0,8991	0,95533	no
XLOC_005881	g8754	88,7442	64,0402	-0,470676	-0,785546	0,15895	0,39933	no
XLOC_005882	g8755	46,2115	47,9185	0,0523303	0,0915114	0,87625	0,944231	no
XLOC_005883	g8756	14,8863	11,0887	-0,424896	-0,61005	0,2897	0,552721	no
XLOC_005884	g8757	61,9787	60,6232	-0,0319015	-0,0545053	0,9284	0,968234	no
XLOC_005885	g8758	38,1394	38,4339	0,0110984	0,0187221	0,97215	0,987026	no
XLOC_005886	g8759	125,77	95,6161	-0,395465	-0,650903	0,2631	0,523428	no
XLOC_005887	g2920	29,0979	26,7851	-0,119487	-0,182313	0,75045	0,882354	no

XLOC_005888	g8760	65,5079	84,123	0,360832	0,597901	0,29925	0,561558	no
XLOC_005889	g8761	45,2401	56,4248	0,31873	0,52854	0,337	0,597208	no
XLOC_005890	g8762	240,035	151,134	-0,667419	-1,09081	0,06065	0,221571	no
XLOC_005891	g8763	11,7631	10,5431	-0,157974	-0,207705	0,70865	0,861789	no
XLOC_005892	g8764	141,36	149,938	0,0849969	0,140374	0,80945	0,912397	no
XLOC_005893	g8765	116,647	7,06535	-4,04525	-5,51039	5,00E-05	0,00120049	yes
XLOC_005894	g8766	86,2254	174,409	1,01629	0,945861	0,0971	0,298255	no
XLOC_005895	g8767	0	0	0	0	1	1	no
XLOC_005896	g8768	27,4241	13,6638	-1,00508	-0,914597	0,11555	0,333246	no
XLOC_005897	g2921	21,1481	23,8286	0,172167	0,241269	0,6649	0,835806	no
XLOC_005898	g2922	1,60658	2,21392	0,46261	0,528072	0,36925	0,628841	no
XLOC_005899	g2923	1,44162	11,8672	3,04122	2,5171	0,0044	0,0374419	yes
XLOC_005900	g2924	15,8946	13,4458	-0,241388	-0,28059	0,6204	0,808752	no
XLOC_005901	g8769	25,4717	25,682	0,0118576	0,0161361	0,97415	0,987624	no
XLOC_005902	g2926	3,61357	2,17483	-0,732521	-0,934296	0,11815	0,337571	no
XLOC_005903	g2925	7,83936	16,2958	1,05569	1,65803	0,0046	0,0386416	yes
XLOC_005904	g8770	209,839	144,54	-0,537811	-0,832038	0,14415	0,380116	no
XLOC_005905	g8771	0	0	0	0	1	1	no
XLOC_005906	g8772	177,775	186,804	0,0714759	0,121721	0,82645	0,920883	no
XLOC_005907	g8773	9,8907	6,94232	-0,510655	-0,721473	0,2151	0,470651	no
XLOC_005908	g8774	21,9659	13,9702	-0,652918	-0,870067	0,1378	0,370231	no
XLOC_005909	g8775	124,136	149,575	0,268941	0,445838	0,42335	0,67289	no
XLOC_005910	g8776	0	0	0	0	1	1	no
XLOC_005911	g8777	0	0	0	0	1	1	no
XLOC_005912	g404	18,699	43,0674	1,20363	1,75689	0,0022	0,0225747	yes
XLOC_005913	g8778	0	0	0	0	1	1	no
XLOC_005914	g8779	63,9054	46,3647	-0,46291	-0,755921	0,1827	0,429978	no
XLOC_005915	g8780	0	5,35012	inf	#NAME?	5,00E-05	0,00120049	yes
XLOC_005916	g8781	0	0	0	0	1	1	no
XLOC_005917	g8782	0	0	0	0	1	1	no
XLOC_005918	g8783	14,668	19,6896	0,424754	0,595361	0,28045	0,542664	no
XLOC_005919	g8784	0	0	0	0	1	1	no
XLOC_005920	g8785	0	0	0	0	1	1	no
XLOC_005921	g8786	0	0	0	0	1	1	no
XLOC_005922	g405	0	0	0	0	1	1	no
XLOC_005923	g406	0	0	0	0	1	1	no
XLOC_005924	g8787	36,4788	46,0242	0,335334	0,46425	0,4231	0,672849	no
XLOC_005925	g8788	4,11129	4,8554	0,239999	0,288898	0,61535	0,806882	no
XLOC_005926	g8789	0,315282	0,452787	0,522189	0	1	1	no
XLOC_005927	g8790	0	0	0	0	1	1	no
XLOC_005928	g8791	33,4795	39,0341	0,221458	0,326417	0,5759	0,780604	no
XLOC_005929	g8792	25,9386	32,6239	0,33083	0,518656	0,3567	0,617124	no
XLOC_005930	g8793	33,8652	56,6298	0,741756	1,25378	0,0306	0,143091	no
XLOC_005931	g8794	0	0,564609	inf	0	1	1	no
XLOC_005932	g407	20,1416	23,9075	0,247284	0,361885	0,51185	0,737978	no
XLOC_005933	g8795	130,14	1747,9	3,74748	4,93857	5,00E-05	0,00120049	yes
XLOC_005934	g8796	0	0	0	0	1	1	no
XLOC_005935	g8797	1,64931	3,46658	1,07165	0,901219	0,2014	0,453612	no
XLOC_005936	g2927	20,2202	23,9601	0,244834	0,409647	0,4719	0,70959	no
XLOC_005937	g2928	10,858	23,2963	1,10134	1,49655	0,01235	0,0774438	no
XLOC_005938	g8798	0	0	0	0	1	1	no
XLOC_005939	g8799	0	0	0	0	1	1	no
XLOC_005940	g8800	62,8877	172,45	1,45533	2,34104	0,00015	0,00294012	yes
XLOC_005941	g8801	40,8547	30,3372	-0,429417	-0,609583	0,2886	0,551993	no
XLOC_005942	g2929	47,5915	42,6529	-0,158061	-0,26355	0,63755	0,819791	no
XLOC_005943	g2931	31,8309	55,6356	0,805582	1,13624	0,0463	0,186491	no
XLOC_005944	g2934	36,7594	35,2858	-0,0590253	-0,101253	0,85455	0,934694	no
XLOC_005945	g2935	7,29433	4,06717	-0,842751	-1,08369	0,0681	0,238841	no
XLOC_005946	g2930	21,1027	19,6204	-0,105075	-0,168332	0,75465	0,884327	no
XLOC_005947	g2932	114,865	73,5217	-0,643701	-1,09397	0,0507	0,198594	no
XLOC_005948	g2933	67,5571	95,4298	0,498332	0,777059	0,16325	0,405236	no
XLOC_005949	g8802	0	0	0	0	1	1	no
XLOC_005950	g8803	73,2461	26,9689	-1,44146	-1,70503	0,00285	0,0272512	yes
XLOC_005951	g8804	582,421	744,271	0,353765	0,572388	0,3056	0,566867	no
XLOC_005952	g8805	98,842	100,4	0,0225669	0,0385584	0,9453	0,975499	no
XLOC_005953	g410	103,381	99,0789	-0,0613263	-0,0806274	0,8785	0,945514	no
XLOC_005954	g411	61,3638	56,1499	-0,128104	-0,217472	0,7033	0,859113	no
XLOC_005955	g413	65,8182	86,3367	0,391487	0,678581	0,2407	0,49898	no
XLOC_005956	g414	66,0509	51,9744	-0,345776	-0,399846	0,48085	0,715629	no
XLOC_005957	g415	54,1148	103,873	0,940726	1,04235	0,08215	0,269713	no
XLOC_005958	g408	102,717	61,9763	-0,728884	-0,890296	0,1184	0,337981	no
XLOC_005959	g409	0,164243	0	#NAME?	0	1	1	no
XLOC_005960	g412	32,4941	25,4501	-0,352506	-0,58946	0,3047	0,566374	no
XLOC_005961	g2936	25,7112	21,4702	-0,26006	-0,401319	0,48035	0,71557	no
XLOC_005962	g8806	58,7223	170,837	1,54064	2,60211	5,00E-05	0,00120049	yes
XLOC_005963	g8807	27,4093	18,8437	-0,540582	-0,819882	0,14745	0,384102	no
XLOC_005964	g8808	0	0	0	0	1	1	no
XLOC_005965	g2938	39,1214	26,201	-0,578336	-0,844807	0,13525	0,365817	no
XLOC_005966	g2939	366,665	362,136	-0,0179313	-0,0277444	0,96005	0,982153	no
XLOC_005967	g2937	796,623	1545,23	0,955851	1,21761	0,02995	0,141467	no
XLOC_005968	g2940	551,331	52,4688	-3,39339	-4,68419	5,00E-05	0,00120049	yes
XLOC_005969	g8809	129,496	98,0976	-0,400618	-0,610549	0,2655	0,52639	no
XLOC_005970	g8810	0	0	0	0	1	1	no
XLOC_005971	g8811	0	0	0	0	1	1	no
XLOC_005972	g8812	56,8301	57,6104	0,0196735	0,0338726	0,95215	0,978762	no
XLOC_005973	g8813	30,0491	41,793	0,475938	0,698572	0,20825	0,462987	no
XLOC_005974	g417	93,8909	71,0392	-0,402369	-0,648908	0,25465	0,513905	no
XLOC_005975	g420	87,8657	80,6591	-0,123462	-0,205023	0,72425	0,86956	no
XLOC_005976	g424	37,6681	56,3337	0,580655	0,994372	0,07975	0,264308	no
XLOC_005977	g426	92,2719	80,7807	-0,191879	-0,27175	0,623	0,81085	no
XLOC_005978	g427	194,873	228,693	0,23088	0,334751	0,5539	0,767387	no
XLOC_005979	g429	22,2152	11,2499	-0,981641	-1,44832	0,01035	0,068167	no

XLOC_005980	g430	1899,2	675,12	-1,49218	-2,29127	5,00E-05	0,00120049	yes
XLOC_005981	g431	13,2682	10,6503	-0,317083	-0,460755	0,41565	0,667815	no
XLOC_005982	g432	77,6674	67,1203	-0,210559	-0,319244	0,58385	0,784861	no
XLOC_005983	g433	86,6362	52,9689	-0,709826	-1,21509	0,0334	0,151566	no
XLOC_005984	g435	222,887	152,232	-0,550042	-0,926771	0,09285	0,290512	no
XLOC_005985	g437	7,42668	10,2303	0,46206	0,721087	0,2094	0,464283	no
XLOC_005986	g416	17,4641	16,7107	-0,0636172	-0,081045	0,888	0,950219	no
XLOC_005987	g418	2,62147	3,69057	0,493468	0,66899	0,2479	0,506634	no
XLOC_005988	g419	25,3935	69,3898	1,45026	2,34686	0,00025	0,00430702	yes
XLOC_005989	g421	69,9608	67,4049	-0,0536942	-0,0871599	0,8781	0,945394	no
XLOC_005990	g422	34,513	40,0437	0,214435	0,312766	0,5838	0,784861	no
XLOC_005991	g423	61,9653	109,852	0,826027	1,37722	0,01685	0,0959229	no
XLOC_005992	g425	52,2474	57,6858	0,142858	0,245667	0,66545	0,836176	no
XLOC_005993	g428	210,833	143,603	-0,554015	-0,807657	0,14955	0,387107	no
XLOC_005994	g434	34,0519	36,3543	0,0943921	0,159049	0,78105	0,898537	no
XLOC_005995	g436	285,378	115,679	-1,30275	-1,92847	0,00105	0,0130684	yes
XLOC_005996	g8814	0	0	0	0	1	1	no
XLOC_005997	g8815	0	0	0	0	1	1	no
XLOC_005998	g8816	0	0	0	0	1	1	no
XLOC_005999	g8817	0	0	0	0	1	1	no
XLOC_006000	g8818	112,068	94,1408	-0,251486	-0,416906	0,47775	0,713645	no
XLOC_006001	g8819	9,34636	5,74787	-0,701378	-0,818834	0,1503	0,387489	no
XLOC_006002	g8820	0	0	0	0	1	1	no
XLOC_006003	g8821	1,04169	1,66973	0,680688	0,428547	0,43865	0,684902	no
XLOC_006004	g8822	0	0,011893	inf	0	1	1	no
XLOC_006005	g2942	216,248	865,508	2,00086	2,88205	5,00E-05	0,00120049	yes
XLOC_006006	g2941	170,605	170,383	-0,00187858	-0,00300821	0,99635	0,997874	no
XLOC_006007	g8823	300,764	277,897	-0,114081	-0,179899	0,7577	0,8854	no
XLOC_006008	g2943	23,8839	22,1231	-0,110482	-0,163814	0,7709	0,893264	no
XLOC_006009	g2944	0,699498	1,91217	1,45082	1,44022	0,0173	0,097748	no
XLOC_006010	g26	0	0	0	0	1	1	no
XLOC_006011	g2945	53,0727	49,4489	-0,102032	-0,168598	0,767	0,891038	no
XLOC_006012	g2946	24,2222	47,0812	0,958822	1,32163	0,0219	0,116248	no
XLOC_006013	g2948	168,816	87,1459	-0,953947	-1,1241	0,0445	0,181852	no
XLOC_006014	g2947	34,3287	18,3165	-0,906271	-1,18618	0,0353	0,15728	no
XLOC_006015	g8824	0	0	0	0	1	1	no
XLOC_006016	g8825	24,8854	32,441	0,382518	0,64247	0,2587	0,518762	no
XLOC_006017	g8826	105,881	66,162	-0,678372	-1,13272	0,0537	0,20607	no
XLOC_006018	g8827	0	0	0	0	1	1	no
XLOC_006019	g8828	36,3171	52,979	0,544769	0,891455	0,1169	0,33517	no
XLOC_006020	g8829	25,2877	30,7544	0,282355	0,465942	0,4102	0,662854	no
XLOC_006021	g8830	22,8662	32,9863	0,52865	0,83186	0,1688	0,41183	no
XLOC_006022	g8831	57,0199	68,129	0,256802	0,443487	0,43245	0,679357	no
XLOC_006023	g8832	359,865	507,956	0,497245	0,784922	0,2205	0,477466	no
XLOC_006024	g2950	4,06756	2,10329	-0,951514	-1,09336	0,0579	0,215452	no
XLOC_006025	g2949	4,53807	4,54899	0,00346781	0,00295494	0,94295	0,97502	no
XLOC_006026	g2951	556,163	1263,67	1,18404	1,54373	0,00695	0,0515476	no
XLOC_006027	g8833	200,902	137,42	-0,547902	-0,916109	0,1141	0,331302	no
XLOC_006028	g8834	0	0	0	0	1	1	no
XLOC_006029	g8835	5,9584	9,35279	0,650472	0,803908	0,15225	0,390059	no
XLOC_006030	g8836	29,8381	36,8469	0,304387	0,435797	0,43705	0,68432	no
XLOC_006031	g8837	51,1308	34,6515	-0,561275	-0,905687	0,12055	0,34204	no
XLOC_006032	g8838	116,018	233,509	1,00914	1,40014	0,0169	0,0961518	no
XLOC_006033	g2952	17,0821	27,3542	0,679276	0,769627	0,1906	0,440813	no
XLOC_006034	g2953	5,01633	16,5822	1,72493	2,22954	0,00025	0,00430702	yes
XLOC_006035	g8839	0,469855	0,298937	-0,652372	0	1	1	no
XLOC_006036	g8840	23,6844	13,6359	-0,796521	-1,13331	0,03625	0,159344	no
XLOC_006037	g8841	71,9997	68,8669	-0,0641808	-0,108432	0,8459	0,929972	no
XLOC_006038	g438	26,7832	32,8761	0,295707	0,437892	0,4545	0,699121	no
XLOC_006039	g440	90,2346	72,2419	-0,320844	-0,55449	0,3352	0,595523	no
XLOC_006040	g441	34,5817	45,4524	0,394349	0,651561	0,2397	0,497749	no
XLOC_006041	g442	13,8732	8,7425	-0,666185	-0,933634	0,0998	0,302854	no
XLOC_006042	g444	98,609	80,9097	-0,285407	-0,443364	0,4387	0,684902	no
XLOC_006043	g446	818,893	956,651	0,224318	0,367961	0,52365	0,746189	no
XLOC_006044	g448	80,4658	47,3871	-0,763882	-1,32038	0,0218	0,115905	no
XLOC_006045	g439	18,3977	18,9506	0,0427149	0,0710719	0,9017	0,956954	no
XLOC_006046	g443	38,7613	72,0297	0,893976	1,32926	0,018	0,100603	no
XLOC_006047	g445	183,806	127,802	-0,524274	-0,864839	0,12015	0,341102	no
XLOC_006048	g447	25,4537	37,7709	0,569399	0,905355	0,11205	0,326993	no
XLOC_006049	g8842	48,1454	48,1461	2,07E-05	3,56E-05	0,9996	0,9996	no
XLOC_006050	g8843	34,6626	28,3512	-0,289973	-0,399533	0,49715	0,726923	no
XLOC_006051	g8844	0	0	0	0	1	1	no
XLOC_006052	g8845	0	0	0	0	1	1	no
XLOC_006053	g8846	0	0	0	0	1	1	no
XLOC_006054	g8847	34,4576	15,3029	-1,17102	-1,52283	0,00975	0,0653104	no
XLOC_006055	g8848	33,1219	17,9028	-0,887601	-1,32414	0,0265	0,131629	no
XLOC_006056	g8849	0	0	0	0	1	1	no
XLOC_006057	g2954	120,052	134,446	0,163369	0,249354	0,65635	0,831659	no
XLOC_006058	g2955	78,3693	71,7532	-0,127247	-0,176903	0,758	0,8854	no
XLOC_006059	g2957	43,098	49,6406	0,203901	0,345742	0,537	0,75585	no
XLOC_006060	g2960	168,948	133,622	-0,338423	-0,548012	0,33235	0,593162	no
XLOC_006061	g2962	86,9358	95,3478	0,13325	0,216922	0,69085	0,852066	no
XLOC_006062	g2963	75,8041	224,105	1,56383	2,55088	5,00E-05	0,00120049	yes
XLOC_006063	g2964	29,2546	25,6888	-0,187525	-0,280868	0,6179	0,808291	no
XLOC_006064	g2956	258,374	340,764	0,399313	0,626249	0,2528	0,511956	no
XLOC_006065	g2958	2,41823	5,67252	1,23004	1,45056	0,0142	0,0854436	no
XLOC_006066	g2959	7,35175	6,38417	-0,20359	-0,207723	0,71655	0,865607	no
XLOC_006067	g2961	39,4045	29,5639	-0,41452	-0,712541	0,2129	0,468347	no
XLOC_006068	g8850	16,303	11,2469	-0,535606	-0,681269	0,22595	0,483932	no
XLOC_006069	g8851	60,4657	59,1491	-0,0317612	-0,0535191	0,9219	0,96566	no
XLOC_006070	g8852	0	0	0	0	1	1	no
XLOC_006071	g2965	0,0876051	4,46183	5,67048	1,32668	0,1168	0,335079	no

XLOC_006072	g2966	0	0	0	0	1	1	no
XLOC_006073	g2967	0	0	0	0	1	1	no
XLOC_006074	g8853	200,394	155,712	-0,36396	-0,600588	0,3053	0,566738	no
XLOC_006075	g8854	41,1293	36,6649	-0,165768	-0,281979	0,61425	0,805977	no
XLOC_006076	g2968	1,31893	0,994929	-0,406703	-0,371256	0,5229	0,745661	no
XLOC_006077	g2969	16,6418	45,4911	1,45077	2,19974	5,00E-05	0,00120049	yes
XLOC_006078	g8855	16,997	27,7391	0,706643	1,08075	0,06595	0,23338	no
XLOC_006079	g8856	270,656	108,529	-1,31837	-1,93428	0,0005	0,00733931	yes
XLOC_006080	g8857	0	0	0	0	1	1	no
XLOC_006081	g8858	30,4495	29,0517	-0,0677927	-0,0982404	0,86145	0,935986	no
XLOC_006082	g449	44,5655	44,2111	-0,0115197	-0,0183301	0,97335	0,98758	no
XLOC_006083	g450	46,7508	47,7536	0,0306193	0,0475473	0,93125	0,969867	no
XLOC_006084	g8859	15,9857	29,943	0,905436	1,0226	0,0786	0,261524	no
XLOC_006085	g8860	56,0594	38,5365	-0,54073	-0,65538	0,50235	0,730935	no
XLOC_006086	g8861	46,1551	30,9236	-0,577779	-0,809606	0,1615	0,403237	no
XLOC_006087	g2970	100,376	133,937	0,416142	0,604219	0,29555	0,557384	no
XLOC_006088	g8862	163,673	60,2353	-1,44213	-2,30199	0,0001	0,00209829	yes
XLOC_006089	g8863	0,128148	0,0584334	-1,13295	0	1	1	no
XLOC_006090	g2971	62,477	37,662	-0,730215	-1,23787	0,0273	0,133642	no
XLOC_006091	g2973	37,9249	30,6395	-0,307752	-0,521248	0,35015	0,610916	no
XLOC_006092	g2974	61,0981	87,0381	0,51052	0,871458	0,12405	0,347197	no
XLOC_006093	g2975	115,526	58,9813	-0,969889	-1,5922	0,00475	0,0395632	yes
XLOC_006094	g2972	41,1612	47,1516	0,196022	0,325406	0,56995	0,777701	no
XLOC_006095	g2976	10,79	9,132	-0,240697	-0,190035	0,7336	0,874259	no
XLOC_006096	g2977	20,3821	37,5822	0,882743	1,42163	0,0137	0,0833544	no
XLOC_006097	g2978	132,592	208,888	0,655741	0,944907	0,093	0,290512	no
XLOC_006098	g2979	72,0247	152,276	1,08013	1,78818	0,0026	0,0255065	yes
XLOC_006099	g8864	130,537	144,433	0,145933	0,241914	0,65905	0,832458	no
XLOC_006100	g8865	9,80021	11,5052	0,231403	0,304279	0,5961	0,793185	no
XLOC_006101	g8866	52,312	67,9689	0,377732	0,636794	0,27105	0,532449	no
XLOC_006102	g8868	42,2058	32,2585	-0,387762	-0,644809	0,2584	0,51859	no
XLOC_006103	g8867	238,614	111,877	-1,09276	-1,5905	0,0079	0,0564204	no
XLOC_006104	g8869	13,6333	7,4026	-0,881037	-0,810253	0,1725	0,417847	no
XLOC_006105	g2981	74,4236	50,8995	-0,548108	-0,757522	0,18705	0,435371	no
XLOC_006106	g2980	64,2206	35,3629	-0,8608	-1,37888	0,0151	0,0888981	no
XLOC_006107	g8870	0	0	0	0	1	1	no
XLOC_006108	g2982	107,638	70,3891	-0,612762	-0,935642	0,0908	0,286154	no
XLOC_006109	g2983	25,3566	30,6428	0,273184	0,44151	0,43725	0,68432	no
XLOC_006110	g2985	9,99055	8,12726	-0,297796	-0,460333	0,41415	0,666219	no
XLOC_006111	g2987	1290,8	887,851	-0,539871	-0,819518	0,1492	0,386786	no
XLOC_006112	g2989	71,281	72,3314	0,0211028	0,0352958	0,95135	0,978606	no
XLOC_006113	g2990	3,989	1,36798	-1,54398	-1,78551	0,00305	0,028799	yes
XLOC_006114	g2991	135,256	99,8793	-0,437433	-0,69049	0,21605	0,471994	no
XLOC_006115	g2993	81,9169	72,3898	-0,178375	-0,285274	0,61925	0,808526	no
XLOC_006116	g2995	219,793	307,371	0,483839	0,807128	0,15095	0,388451	no
XLOC_006117	g2996	290,119	464,962	0,680469	1,07903	0,06395	0,229444	no
XLOC_006118	g2984	37,5277	35,4161	-0,0835526	-0,140946	0,7993	0,907785	no
XLOC_006119	g2986	185,321	213,795	0,2062	0,355568	0,533	0,752918	no
XLOC_006120	g2988	99,2013	125,479	0,33901	0,56653	0,33355	0,594565	no
XLOC_006121	g2992	297,398	347,159	0,223199	0,308526	0,5804	0,783024	no
XLOC_006122	g2994	4,35068	2,75486	-0,659261	-0,7574	0,2011	0,453352	no
XLOC_006123	g2997	24,128	18,6703	-0,369965	-0,578231	0,36235	0,622621	no
XLOC_006124	g8871	25,9076	22,4948	-0,203782	-0,249587	0,6538	0,829883	no
XLOC_006125	g8872	37,1554	17,7846	-1,06294	-1,49261	0,01025	0,0677355	no
XLOC_006126	g8873	0,337923	1,30885	1,95353	1,22465	0,10455	0,312822	no
XLOC_006127	g2998	5,09038	14,2183	1,48191	1,60768	0,008	0,0570102	no
XLOC_006128	g2999	0	0,273871	inf	0	1	1	no
XLOC_006129	g3000	118,29	103,07	-0,198704	-0,325621	0,5697	0,777616	no
XLOC_006130	g3002	1968,77	2948,72	0,582797	0,710657	0,1707	0,414817	no
XLOC_006131	g3001	16,0667	24,4106	0,603435	0,823669	0,15255	0,39042	no
XLOC_006132	g8874	41,2099	30,8602	-0,417243	-0,592405	0,2864	0,549199	no
XLOC_006133	g8875	0	0	0	0	1	1	no
XLOC_006134	g3003	34,7129	40,3016	0,215366	0,373291	0,51765	0,742416	no
XLOC_006135	g8876	107,271	46,7042	-1,19964	-1,9931	0,00055	0,00788467	yes
XLOC_006136	g8879	28,5206	15,0534	-0,921919	-1,34425	0,0205	0,110976	no
XLOC_006137	g8877	14,3662	18,3768	0,355211	0,512466	0,36905	0,628841	no
XLOC_006138	g8878	9,56635	6,79823	-0,492809	-0,610827	0,29755	0,559437	no
XLOC_006139	g3004	0	0	0	0	1	1	no
XLOC_006140	g8880	0	0,174549	inf	0	1	1	no
XLOC_006141	g8882	57,6759	54,7196	-0,0759103	-0,129789	0,8151	0,915983	no
XLOC_006142	g8881	141,847	165,805	0,22515	0,364746	0,5198	0,743978	no
XLOC_006143	g8883	5,00823	2,38777	-1,06864	-1,69237	0,4043	0,657976	no
XLOC_006144	g8884	0	0	0	0	1	1	no
XLOC_006145	g8885	7,82805	7,62203	-0,0384769	-0,0541377	0,9221	0,965766	no
XLOC_006146	g8886	1,38861	0,629084	-1,14231	-0,951074	0,1093	0,321837	no
XLOC_006147	g8887	1024,77	4596,13	2,16512	1228,21	0,2371	0,495072	no
XLOC_006148	g8889	84,1305	114,427	0,44372	0,760252	0,1822	0,42989	no
XLOC_006149	g8891	23,1083	30,2541	0,38872	0,54456	0,33275	0,59366	no
XLOC_006150	g8888	23,046	26,463	0,199459	0,297922	0,58605	0,786849	no
XLOC_006151	g8890	13,413	9,47534	-0,50138	-0,668866	0,2484	0,507023	no
XLOC_006152	g8892	96,0193	125,51	0,386409	0,555954	0,3127	0,573414	no
XLOC_006153	g8893	0	0	0	0	1	1	no
XLOC_006154	g8894	307,297	215,709	-0,510543	-0,832959	0,14895	0,386342	no
XLOC_006155	g8895	531,153	430,444	-0,303299	-0,479851	0,39565	0,649604	no
XLOC_006156	g8896	28,9707	26,9331	-0,105214	-0,158957	0,7791	0,896924	no
XLOC_006157	g8899	77,9151	62,5482	-0,316935	-0,53073	0,35065	0,611179	no
XLOC_006158	g8897	59,5323	99,8415	0,745967	1,22938	0,03085	0,143918	no
XLOC_006159	g8898	965,358	816,099	-0,242319	-0,372777	0,5172	0,742096	no
XLOC_006160	g8900	0	1,19189	inf	0	1	1	no
XLOC_006161	g3005	0	0,0454763	inf	0	1	1	no
XLOC_006162	g8901	77,3521	275,455	1,8323	3,0136	5,00E-05	0,00120049	yes
XLOC_006163	g8902	247,456	94,1257	-1,39451	-2,20993	0,00025	0,00430702	yes

XLOC_006164	g8903	29,5629	2,25357	-3,7135	-4,74774	5,00E-05	0,00120049	yes
XLOC_006165	g8905	61,4921	21,0496	-1,54661	-1,45671	0,01735	0,0979178	no
XLOC_006166	g8904	81,1629	56,9609	-0,510848	-0,675005	0,2378	0,496002	no
XLOC_006167	g8906	300,131	758,168	1,33693	2,17007	0,0002	0,00367103	yes
XLOC_006168	g8907	0	0	0	0	1	1	no
XLOC_006169	g8908	21,3355	18,5949	-0,198348	-0,252176	0,6567	0,8318	no
XLOC_006170	g8909	12,4206	14,3405	0,207356	0,30667	0,59905	0,795708	no
XLOC_006171	g8910	21,2541	22,8784	0,106247	0,149217	0,792	0,904248	no
XLOC_006172	g8912	118,713	204,819	0,786867	1,143	0,0469	0,187983	no
XLOC_006173	g8914	27,4634	35,4971	0,370191	0,612894	0,28135	0,543655	no
XLOC_006174	g8918	111,712	253,967	1,18485	1,96722	0,00065	0,00901554	yes
XLOC_006175	g8920	22,3924	21,0731	-0,0876117	-0,145149	0,80135	0,908537	no
XLOC_006176	g8923	43,994	36,629	-0,264322	-0,446426	0,43175	0,678909	no
XLOC_006177	g8911	168,845	539,409	1,67568	2,81506	5,00E-05	0,00120049	yes
XLOC_006178	g8913	49,0313	40,6547	-0,27028	-0,408953	0,4638	0,704924	no
XLOC_006179	g8915	116,265	149,142	0,359271	0,580053	0,3055	0,566867	no
XLOC_006180	g8916	250,216	191,36	-0,386886	-0,649897	0,24575	0,504445	no
XLOC_006181	g8917	72,9827	83,8802	0,200776	0,342922	0,54515	0,761892	no
XLOC_006182	g8919	42,308	46,1516	0,12545	0,194754	0,72975	0,871761	no
XLOC_006183	g8921	25,6688	18,9627	-0,436855	-0,636553	0,25895	0,518851	no
XLOC_006184	g8922	150,668	138,386	-0,122674	-0,201817	0,72435	0,869574	no
XLOC_006185	g8924	92,4518	177,385	0,940111	1,37955	0,01565	0,0912065	no
XLOC_006186	g451	156,103	156,335	0,00214524	0,00306921	0,9959	0,997829	no
XLOC_006187	g452	31,19	10,4352	-1,57963	-2,10882	0,0006	0,00845337	yes
XLOC_006188	g8925	0	0	0	0	1	1	no
XLOC_006189	g3006	21,4232	13,2992	-0,687836	-0,917447	0,11625	0,334478	no
XLOC_006190	g8926	3,43043	3,97466	0,21244	0,223931	0,70035	0,857536	no
XLOC_006191	g8927	12,5243	18,6608	0,575276	0,789759	0,16765	0,410862	no
XLOC_006192	g3007	3,52762	5,62056	0,672016	0,951547	0,09065	0,285957	no
XLOC_006193	g3008	0,777053	4,15931	2,42026	1,98766	0,00705	0,0520143	no
XLOC_006194	g3009	36,9208	154,506	2,06516	3,39785	5,00E-05	0,00120049	yes
XLOC_006195	g3010	0	0	0	0	1	1	no
XLOC_006196	g8928	101,121	69,1493	-0,5483	-0,935272	0,0929	0,290512	no
XLOC_006197	g8929	94,1382	109,202	0,214154	0,36008	0,5278	0,74938	no
XLOC_006198	g8930	0	0	0	0	1	1	no
XLOC_006199	g3011	35,7226	22,3493	-0,676611	-1,1271	0,0437	0,17993	no
XLOC_006200	g3012	7,69473	14,7361	0,937417	1,41499	0,01405	0,0848009	no
XLOC_006201	g3013	15,2484	16,0015	0,0695477	0,113389	0,83965	0,926574	no
XLOC_006202	g3014	80,6442	60,3414	-0,418422	-0,671594	0,22795	0,486095	no
XLOC_006203	g3015	2596,66	2606,01	0,00518064	0,00660714	0,9907	0,995057	no
XLOC_006204	g8931	77,4811	50,5774	-0,61535	-1,02739	0,06945	0,241672	no
XLOC_006205	g8932	34,2971	37,5556	0,130943	0,168374	0,7708	0,893264	no
XLOC_006206	g8933	6,71482	6,29578	-0,0929651	-0,127872	0,8227	0,919263	no
XLOC_006207	g8934	102,409	6581,82	6,00608	5,63184	5,00E-05	0,00120049	yes
XLOC_006208	g453	0	0	0	0	1	1	no
XLOC_006209	g8935	0	0	0	0	1	1	no
XLOC_006210	g3016	19,8365	28,0624	0,500484	0,647642	0,2666	0,527597	no
XLOC_006211	g8936	42,068	41,1977	-0,0301596	-0,050188	0,93075	0,969449	no
XLOC_006212	g8937	0,257574	0,362457	0,492826	0	1	1	no
XLOC_006213	g3017	68,3746	17,9352	-1,93067	-2,83428	5,00E-05	0,00120049	yes
XLOC_006214	g3018	34,6507	20,8727	-0,731268	-1,24362	0,0273	0,133642	no
XLOC_006215	g3019	0	0	0	0	1	1	no
XLOC_006216	g8938	54,6502	74,9607	0,455908	0,754169	0,18675	0,434879	no
XLOC_006217	g8940	19,7681	13,295	-0,572293	-0,871454	0,1304	0,357689	no
XLOC_006218	g8939	1,33143	1,1262	-0,241519	-0,15171	0,771	0,893264	no
XLOC_006219	g8941	0	0	0	0	1	1	no
XLOC_006220	g3020	9,55366	22,1497	1,21316	1,7459	0,00335	0,0307449	yes
XLOC_006221	g3021	8,44257	6,99376	-0,271614	-0,362183	0,52245	0,745381	no
XLOC_006222	g3023	67,1903	56,0589	-0,261311	-0,436442	0,4314	0,678624	no
XLOC_006223	g3022	88,058	65,9906	-0,416194	-0,627847	0,27135	0,532816	no
XLOC_006224	g3024	29,7551	25,4139	-0,22752	-0,249112	0,6602	0,833204	no
XLOC_006225	g8942	1,66223	0,336895	-2,30275	-1,54949	0,10895	0,321288	no
XLOC_006226	g3026	48,2371	15,3054	-1,6561	-2,71482	5,00E-05	0,00120049	yes
XLOC_006227	g3027	11,9171	3,90431	-1,6099	-1,77714	0,00485	0,0400901	yes
XLOC_006228	g3028	96,7596	65,8427	-0,555382	-0,930859	0,10375	0,311187	no
XLOC_006229	g3025	46,5593	50,6322	0,120987	0,159374	0,77555	0,895146	no
XLOC_006230	g3029	85,5919	82,373	-0,0553015	-0,0939307	0,8673	0,939446	no
XLOC_006231	g8943	0	0	0	0	1	1	no
XLOC_006232	g8944	0	0	0	0	1	1	no
XLOC_006233	g8945	54,9498	54,4632	-0,0128322	-0,0217106	0,96665	0,985414	no
XLOC_006234	g8946	272,7	1611,82	2,56331	3,31038	5,00E-05	0,00120049	yes
XLOC_006235	g8947	192,109	159,593	-0,267529	-0,431065	0,4379	0,684524	no
XLOC_006236	g8948	0	0	0	0	1	1	no
XLOC_006237	g8949	0	0	0	0	1	1	no
XLOC_006238	g3031	54,2023	42,3734	-0,355195	-0,606204	0,2844	0,546579	no
XLOC_006239	g3030	5,45107	2,19134	-1,31473	-1,75246	0,00275	0,0265797	yes
XLOC_006240	g3032	21,5138	22,2791	0,0504312	0,0850186	0,8834	0,947933	no
XLOC_006241	g8950	44,3741	58,9142	0,408898	0,652507	0,2479	0,506634	no
XLOC_006242	g8951	44,9942	37,9816	-0,244438	-0,420549	0,46015	0,701766	no
XLOC_006243	g8952	5,70629	16,1233	1,49852	2,13188	0,00025	0,00430702	yes
XLOC_006244	g3033	48,7165	39,8633	-0,289349	-0,498459	0,38435	0,640475	no
XLOC_006245	g3034	2,31976	3,64934	0,653658	0,854907	0,1496	0,387107	no
XLOC_006246	g8953	76,9739	133,033	0,789337	1,19362	0,04285	0,177622	no
XLOC_006247	g8954	77,5507	104,945	0,436423	0,6938	0,2193	0,476549	no
XLOC_006248	g8955	37,2334	37,815	0,0223643	0,0342467	0,9553	0,98046	no
XLOC_006249	g8956	0	0	0	0	1	1	no
XLOC_006250	g8957	20,9236	13,379	-0,645164	-0,912625	0,10415	0,3121	no
XLOC_006251	g8958	5,33933	7,10775	0,412734	0,554882	0,3319	0,592663	no
XLOC_006252	g454	22,9709	35,8812	0,643422	1,03849	0,06135	0,223298	no
XLOC_006253	g455	42,9352	39,1407	-0,133491	-0,205408	0,71965	0,867399	no
XLOC_006254	g8959	16,0683	13,7066	-0,229349	-0,308468	0,59455	0,792155	no
XLOC_006255	g8960	190,468	224,615	0,237904	0,372799	0,50835	0,734874	no

XLOC_006256	g8961	0	0	0	0	1	1	no
XLOC_006257	g8962	23,2516	59,4083	1,35333	1,81189	0,00115	0,0138564	yes
XLOC_006258	g3035	0	0	0	0	1	1	no
XLOC_006259	g8963	0	0	0	0	1	1	no
XLOC_006260	g8965	0	0	0	0	1	1	no
XLOC_006261	g8966	0	0	0	0	1	1	no
XLOC_006262	g8964	0	0	0	0	1	1	no
XLOC_006263	g3036	0,532643	0,413128	-0,366578	0	1	1	no
XLOC_006264	g3037	149,797	107,111	-0,48391	-0,658984	0,2235	0,480993	no
XLOC_006265	g8967	1,33236	1,56701	0,23403	0,269665	0,6462	0,825616	no
XLOC_006266	g3038	6,25987	3,30654	-0,920811	-1,07308	0,0661	0,233826	no
XLOC_006267	g3039	18,1285	18,2071	0,0062411	0,0054639	0,95275	0,978815	no
XLOC_006268	g3040	7,01268	5,3915	-0,379279	-0,465753	0,4159	0,667998	no
XLOC_006269	g3042	5,4016	1,74076	-1,63367	-1,09862	0,3206	0,580544	no
XLOC_006270	g3044	0,692469	0,375602	-0,882543	0	1	1	no
XLOC_006271	g3045	0,421403	0,233246	-0,853348	0	1	1	no
XLOC_006272	g3047	4,28821	3,74239	-0,196416	-0,222593	0,707	0,860848	no
XLOC_006273	g3041	1,73132	0,575856	-1,58809	-0,864868	0,3291	0,590276	no
XLOC_006274	g3043	13,0957	7,07414	-0,888466	-1,20053	0,0348	0,155688	no
XLOC_006275	g3046	20,9643	32,1456	0,616684	0,992908	0,0908	0,286154	no
XLOC_006276	g3048	26,6186	54,5926	1,03627	1,23097	0,03495	0,156217	no
XLOC_006277	g3049	0	0	0	0	1	1	no
XLOC_006278	g3050	0	0	0	0	1	1	no
XLOC_006279	g3051	0	0	0	0	1	1	no
XLOC_006280	g3052	0	0	0	0	1	1	no
XLOC_006281	g8968	45,0992	51,4557	0,19023	0,293203	0,5918	0,790569	no
XLOC_006282	g3053	16,3051	10,964	-0,572552	-0,621283	0,281	0,5433	no
XLOC_006283	g3054	15,7778	11,5625	-0,448447	-0,719498	0,2066	0,461094	no
XLOC_006284	g3055	22,5034	12,6258	-0,833766	-1,25146	0,0289	0,138101	no
XLOC_006285	g8969	0	0,793525	inf	0	1	1	no
XLOC_006286	g3057	13463	1751,51	-2,94233	-3,20895	5,00E-05	0,00120049	yes
XLOC_006287	g3056	77,2602	63,0766	-0,292619	-0,481482	0,3908	0,646031	no
XLOC_006288	g3058	7,7927	7,01441	-0,151801	-0,22062	0,70155	0,858424	no
XLOC_006289	g3059	10,5674	8,36767	-0,336717	-0,486575	0,3906	0,645848	no
XLOC_006290	g8970	4,67243	4,47698	-0,061645	-0,0846488	0,88175	0,947197	no
XLOC_006291	g8972	42,8396	128,759	1,58766	2,63748	5,00E-05	0,00120049	yes
XLOC_006292	g8971	602,706	181,502	-1,73147	-2,34038	5,00E-05	0,00120049	yes
XLOC_006293	g8973	107,157	45,0284	-1,25081	-1,85381	0,00175	0,0188639	yes
XLOC_006294	g8974	0	0	0	0	1	1	no
XLOC_006295	g3060	84,5251	97,8775	0,211597	0,355047	0,52775	0,74938	no
XLOC_006296	g8975	0	0	0	0	1	1	no
XLOC_006297	g8976	10,739	7,98227	-0,427986	-0,272409	0,60245	0,798521	no
XLOC_006298	g8978	45,5211	31,4474	-0,533596	-0,877542	0,11875	0,338586	no
XLOC_006299	g8977	133,241	69,0579	-0,948162	-1,59726	0,0062	0,0477522	yes
XLOC_006300	g8979	64,6714	68,9617	0,092666	0,147908	0,79485	0,905607	no
XLOC_006301	g3062	17,1238	15,225	-0,16956	-0,258903	0,6428	0,8232	no
XLOC_006302	g3065	21,462	16,241	-0,402144	-0,625659	0,27765	0,539372	no
XLOC_006303	g3066	256,35	435,651	0,765058	1,09376	0,06535	0,231841	no
XLOC_006304	g3061	0,561112	1,08881	0,956396	0,599308	0,3051	0,566474	no
XLOC_006305	g3063	0,934384	2,49873	1,41911	1,43292	0,0193	0,106177	no
XLOC_006306	g3064	12,9186	22,2336	0,783293	1,20932	0,0355	0,157671	no
XLOC_006307	g8980	62,8136	70,4335	0,165185	0,230927	0,68395	0,848029	no
XLOC_006308	g8981	3,59179	10,5236	1,55085	0	1	1	no
XLOC_006309	g8982	0	0	0	0	1	1	no
XLOC_006310	g3067	55,5551	49,418	-0,168882	-0,276289	0,62935	0,814495	no
XLOC_006311	g8983	189,672	77,4626	-1,29193	-1,32799	0,029	0,138377	no
XLOC_006312	g8984	0	0	0	0	1	1	no
XLOC_006313	g8985	6,86535	8,16101	0,249414	0,337568	0,55785	0,77025	no
XLOC_006314	g3069	30,2829	18,0411	-0,74722	-1,07108	0,06185	0,224203	no
XLOC_006315	g3070	31,4403	21,9451	-0,518714	-0,77267	0,175	0,420892	no
XLOC_006316	g3068	2,93167	3,28843	0,165676	0,215978	0,7044	0,859387	no
XLOC_006317	g3071	32,085	31,1812	-0,0412188	-0,0505828	0,93645	0,972153	no
XLOC_006318	g3073	384,733	478,793	0,315547	0,486263	0,384	0,640475	no
XLOC_006319	g3072	55,1287	54,7618	-0,00963421	-0,0145664	0,9798	0,990233	no
XLOC_006320	g3074	26,3419	27,4773	0,060883	0,101611	0,8544	0,934634	no
XLOC_006321	g8986	60,1644	62,8527	0,0630647	0,0731645	0,9026	0,957458	no
XLOC_006322	g8987	121,186	141,722	0,225838	0,366903	0,5193	0,743805	no
XLOC_006323	g8989	196,694	84,7334	-1,21495	-1,71215	0,00475	0,0395632	yes
XLOC_006324	g8988	123,671	98,109	-0,334049	-0,561186	0,3256	0,586355	no
XLOC_006325	g8990	1,88504	1,33698	-0,495619	-0,559309	0,3113	0,572236	no
XLOC_006326	g8991	24,7973	10,2508	-1,27444	-1,71588	0,0033	0,0304282	yes
XLOC_006327	g8992	1,31016	1,30706	-0,00341381	0	1	1	no
XLOC_006328	g3075	123,139	100,189	-0,297562	-0,398224	0,47465	0,711939	no
XLOC_006329	g457	186,579	112,719	-0,727053	-0,931728	0,0975	0,299297	no
XLOC_006330	g458	14,3222	11,6928	-0,292623	-0,447295	0,423	0,672849	no
XLOC_006331	g456	0,831222	1,97248	1,2467	1,36291	0,0254	0,127912	no
XLOC_006332	g459	92,3943	67,8469	-0,445521	-0,687161	0,2306	0,487722	no
XLOC_006333	g8993	5,49428	7,75356	0,49693	0,652439	0,25405	0,513318	no
XLOC_006334	g8994	65,4361	122,085	0,899722	1,4829	0,01215	0,0766793	no
XLOC_006335	g8995	0	0	0	0	1	1	no
XLOC_006336	g3076	8,05937	12,5186	0,635334	0,755169	0,1767	0,423735	no
XLOC_006337	g3077	32,4189	58,3819	0,848687	1,27111	0,02365	0,122556	no
XLOC_006338	g3078	14,4093	16,9311	0,232678	0,322825	0,57065	0,777831	no
XLOC_006339	g8996	0	0	0	0	1	1	no
XLOC_006340	g8997	0	0	0	0	1	1	no
XLOC_006341	g3080	20,2247	55,727	1,46226	2,10126	0,0003	0,00496796	yes
XLOC_006342	g3081	3,13241	1,01686	-1,62315	-1,60614	0,01255	0,0783976	no
XLOC_006343	g3079	10,7909	44,3991	2,04072	2,88088	5,00E-05	0,00120049	yes
XLOC_006344	g3082	230,173	230,359	0,00116508	0,00188031	0,99695	0,997966	no
XLOC_006345	g8998	0	0	0	0	1	1	no
XLOC_006346	g8999	0,832173	1,53082	0,879353	0,594386	0,36345	0,623397	no
XLOC_006347	g3083	64,7046	53,8691	-0,264412	-0,437607	0,4377	0,68432	no

XLOC_006348	g3085	101,742	90,0027	-0,176868	-0,27616	0,62805	0,813648	no
XLOC_006349	g3087	66,226	128,99	0,961793	1,56743	0,0053	0,0425908	yes
XLOC_006350	g3089	36,6987	37,3535	0,0255128	0,0399336	0,94455	0,975496	no
XLOC_006351	g3084	75,185	52,3727	-0,521631	-0,885117	0,1249	0,348384	no
XLOC_006352	g3086	46,6295	96,956	1,05609	1,6903	0,0029	0,0276485	yes
XLOC_006353	g3088	8,46604	10,5915	0,323144	0,497115	0,3915	0,646031	no
XLOC_006354	g9000	43,7332	35,1874	-0,313669	-0,402432	0,4874	0,720794	no
XLOC_006355	g9001	56,6881	52,7511	-0,103845	-0,176738	0,75295	0,883683	no
XLOC_006356	g3091	5,2045	6,98027	0,423524	0,562771	0,3319	0,592663	no
XLOC_006357	g3090	131,105	71,3606	-0,877521	-1,47492	0,009	0,0618908	no
XLOC_006358	g9002	1,50013	0	#NAME?	#NAME?	0,00775	0,0557955	no
XLOC_006359	g9003	253,4	229,261	-0,144431	-0,24836	0,6682	0,838238	no
XLOC_006360	g9004	9,95558	12,4837	0,326468	0,404562	0,47385	0,711248	no
XLOC_006361	g9005	0	0	0	0	1	1	no
XLOC_006362	g9007	40,271	2,84737	-3,82204	-3,91948	0,00015	0,00294012	yes
XLOC_006363	g9006	43,5291	153,328	1,81657	2,40328	5,00E-05	0,00120049	yes
XLOC_006364	g9009	4,01943	1,67745	-1,26072	-1,21284	0,11845	0,337981	no
XLOC_006365	g9011	5,98094	15,2211	1,34763	1,87607	0,00155	0,0171215	yes
XLOC_006366	g9008	0,518163	0,159677	-1,69825	0	1	1	no
XLOC_006367	g9010	19,768	11,4428	-0,788723	-1,08307	0,0624	0,225365	no
XLOC_006368	g9012	0	0	0	0	1	1	no
XLOC_006369	g3092	0	0	0	0	1	1	no
XLOC_006370	g3094	83,8308	123,048	0,553674	0,73818	0,2013	0,453491	no
XLOC_006371	g3093	24,5889	22,013	-0,159647	-0,244313	0,66555	0,836195	no
XLOC_006372	g3095	245,342	290,614	0,244312	0,420726	0,46505	0,705296	no
XLOC_006373	g3097	51,7496	44,5652	-0,215631	-0,336717	0,56355	0,773346	no
XLOC_006374	g3098	39,8191	30,0911	-0,404124	-0,651751	0,24015	0,498367	no
XLOC_006375	g3100	93,8533	74,0255	-0,342386	-0,556333	0,32015	0,580479	no
XLOC_006376	g3101	56,3219	53,5761	-0,0721056	-0,12254	0,8335	0,924151	no
XLOC_006377	g3103	62,318	95,038	0,608857	0,954247	0,10045	0,303888	no
XLOC_006378	g3105	652,061	1177,07	0,852124	1,05763	0,06735	0,236968	no
XLOC_006379	g3109	74,5482	60,7164	-0,296087	-0,492771	0,37255	0,631044	no
XLOC_006380	g3096	364,688	207,777	-0,811627	-1,34284	0,0193	0,106177	no
XLOC_006381	g3099	63,1809	57,927	-0,125251	-0,21422	0,70045	0,857551	no
XLOC_006382	g3102	144,609	197,069	0,446545	0,751539	0,18195	0,42961	no
XLOC_006383	g3104	55,8139	51,4903	-0,116323	-0,1692	0,76235	0,887899	no
XLOC_006384	g3106	94,8653	103,195	0,121417	0,212538	0,7171	0,865633	no
XLOC_006385	g3107	66,0625	54,6798	-0,272822	-0,459941	0,4216	0,671878	no
XLOC_006386	g3108	58,2607	42,6113	-0,451286	-0,77437	0,17515	0,421149	no
XLOC_006387	g3110	79,7894	39,6595	-1,00853	-1,66408	0,00405	0,0351645	yes
XLOC_006388	g9013	0,137918	0,454003	1,71889	0	1	1	no
XLOC_006389	g9014	9,41952	5,62066	-0,744913	-0,959547	0,09645	0,297002	no
XLOC_006390	g9015	0	5,48357	inf	#NAME?	5,00E-05	0,00120049	yes
XLOC_006391	g9016	9,79909	15,0828	0,622183	0,853434	0,1392	0,372294	no
XLOC_006392	g9017	0	0	0	0	1	1	no
XLOC_006393	g9018	0	0	0	0	1	1	no
XLOC_006394	g9019	0	0	0	0	1	1	no
XLOC_006395	g9020	125,852	161,038	0,35567	0,516989	0,369	0,628841	no
XLOC_006396	g9022	243,861	438,462	0,84639	1,46332	0,01315	0,0811137	no
XLOC_006397	g9021	8,52853	4,07696	-1,0648	-1,04345	0,0855	0,276278	no
XLOC_006398	g9023	135,532	120,188	-0,173335	-0,291685	0,61405	0,805977	no
XLOC_006399	g9024	57,3061	40,1118	-0,514663	-0,798147	0,1537	0,391831	no
XLOC_006400	g9025	17,0288	8,12291	-1,06791	-1,29567	0,027	0,133036	no
XLOC_006401	g9026	0	0	0	0	1	1	no
XLOC_006402	g9027	0	0	0	0	1	1	no
XLOC_006403	g9028	1,7387	2,47	0,506502	0,661581	0,24025	0,498469	no
XLOC_006404	g9029	0,639007	4,92918	2,94744	1,36113	0,01975	0,108047	no
XLOC_006405	g9030	3,01348	5,14261	0,771067	0,914727	0,12835	0,353945	no
XLOC_006406	g9031	0	2,64251	inf	#NAME?	5,00E-05	0,00120049	yes
XLOC_006407	g9032	0	0	0	0	1	1	no
XLOC_006408	g9033	123,946	159,079	0,360029	0,504583	0,36045	0,620877	no
XLOC_006409	g9034	110,119	103,296	-0,0922706	-0,161422	0,77875	0,896837	no
XLOC_006410	g9035	7,04207	12,6071	0,84017	0,852836	0,15105	0,388505	no
XLOC_006411	g9036	0	0	0	0	1	1	no
XLOC_006412	g3111	15,7021	30,6861	0,96663	1,33654	0,02105	0,113019	no
XLOC_006413	g9037	0	0	0	0	1	1	no
XLOC_006414	g9038	0	0	0	0	1	1	no
XLOC_006415	g9039	0,0995755	0	#NAME?	0	1	1	no
XLOC_006416	g9041	51,8777	45,643	-0,184723	-0,299751	0,5877	0,78831	no
XLOC_006417	g9040	96,3459	140,441	0,543666	0,911334	0,1075	0,319217	no
XLOC_006418	g9042	131,689	97,3774	-0,435471	-0,712117	0,20145	0,45362	no
XLOC_006419	g9043	103,802	170,811	0,718562	1,23445	0,0342	0,153704	no
XLOC_006420	g9045	21,1075	20,7247	-0,0264045	-0,0450888	0,93895	0,972728	no
XLOC_006421	g9044	86,2174	73,0735	-0,238631	-0,404875	0,48385	0,717735	no
XLOC_006422	g9046	100,764	90,0064	-0,162885	-0,275659	0,6175	0,808291	no
XLOC_006423	g9050	3,19432	2,03342	-0,651602	-0,62735	0,26575	0,526567	no
XLOC_006424	g9047	91,3501	122,41	0,422246	0,491023	0,3942	0,648851	no
XLOC_006425	g9048	1,17099	0	#NAME?	0	1	1	no
XLOC_006426	g9049	3,5954	9,89731	1,46089	1,11954	0,06735	0,236968	no
XLOC_006427	g9051	1,7464	6,77345	1,95551	1,0324	0,09665	0,297431	no
XLOC_006428	g9052	0	0	0	0	1	1	no
XLOC_006429	g9053	46,1777	31,1925	-0,565997	-0,943119	0,0969	0,29792	no
XLOC_006430	g9054	633,106	272,07	-1,21847	-1,92471	0,0011	0,0134856	yes
XLOC_006431	g9055	0	0	0	0	1	1	no
XLOC_006432	g3112	50,3513	39,5725	-0,347531	-0,579223	0,3049	0,566474	no
XLOC_006433	g9056	0	0	0	0	1	1	no
XLOC_006434	g9057	0	0	0	0	1	1	no
XLOC_006435	g9059	295,453	170,033	-0,797116	-0,945915	0,09105	0,286666	no
XLOC_006436	g9060	22,6877	38,6852	0,769871	1,28721	0,02255	0,118481	no
XLOC_006437	g9062	45,4159	94,0791	1,05068	1,68668	0,0047	0,03928	yes
XLOC_006438	g9058	30,1905	44,7606	0,568137	0,896177	0,14145	0,375517	no
XLOC_006439	g9061	23,4148	21,1817	-0,144605	-0,247737	0,6678	0,83795	no

XLOC_006440	g9063	28,0532	34,3958	0,29407	0,459327	0,42085	0,671416	no
XLOC_006441	g9064	41,4697	38,9771	-0,089432	-0,15426	0,7892	0,90274	no
XLOC_006442	g3113	26,4049	34,5719	0,388792	0,64883	0,2478	0,506634	no
XLOC_006443	g3114	13,0878	11,6716	-0,16522	-0,236715	0,6759	0,843051	no
XLOC_006444	g9065	4,43714	1,6383	-1,43743	-1,09357	0,1849	0,433346	no
XLOC_006445	g9066	0,811665	4,02818	2,31117	2,37678	0,0002	0,00367103	yes
XLOC_006446	g9067	0	0	0	0	1	1	no
XLOC_006447	g9070	50,5499	59,632	0,23838	0,410618	0,4691	0,707556	no
XLOC_006448	g9072	27,2315	33,5097	0,299303	0,488074	0,3953	0,649464	no
XLOC_006449	g9073	54,7961	33,898	-0,692872	-1,04029	0,06425	0,229955	no
XLOC_006450	g9068	20,957	30,6863	0,550164	0,904888	0,1178	0,336767	no
XLOC_006451	g9069	33,7949	46,3224	0,454904	0,76706	0,1726	0,417986	no
XLOC_006452	g9071	57,5912	69,4726	0,270596	0,44452	0,43035	0,678415	no
XLOC_006453	g3116	12,4498	13,1605	0,0800927	0,113626	0,84475	0,929566	no
XLOC_006454	g3117	42,3051	40,8444	-0,0506964	-0,0855918	0,87965	0,945715	no
XLOC_006455	g3118	99,7762	86,9912	-0,197826	-0,317367	0,57215	0,778835	no
XLOC_006456	g3119	31,9009	36,0278	0,175515	0,290534	0,6103	0,803802	no
XLOC_006457	g3115	41,98	37,5596	-0,160519	-0,229983	0,68935	0,850964	no
XLOC_006458	g3120	9,98304	18,4747	0,888001	1,29087	0,02765	0,134818	no
XLOC_006459	g3121	70,8099	106,121	0,583689	0,88038	0,11915	0,339244	no
XLOC_006460	g3124	398,286	1545,65	1,95634	2,80476	5,00E-05	0,00120049	yes
XLOC_006461	g3122	37,4051	47,5112	0,345031	0,581839	0,30745	0,568259	no
XLOC_006462	g3123	46,0006	68,9101	0,583061	0,975046	0,07875	0,261524	no
XLOC_006463	g3125	38,6507	42,5392	0,138299	0,228083	0,6892	0,850886	no
XLOC_006464	g3126	2,26585	0,871098	-1,37914	-0,963045	0,2005	0,452519	no
XLOC_006465	g3127	0	0	0	0	1	1	no
XLOC_006466	g3128	2,04727	2,4101	0,235385	0,259498	0,65635	0,831659	no
XLOC_006467	g9074	0	0	0	0	1	1	no
XLOC_006468	g9075	0	0	0	0	1	1	no
XLOC_006469	g9076	0	0	0	0	1	1	no
XLOC_006470	g3129	0,0691121	0,60286	3,12481	0	1	1	no
XLOC_006471	g9077	106,011	87,1149	-0,283218	-0,345736	0,5338	0,753431	no
XLOC_006472	g460	39,1414	16,3494	-1,25946	-1,69637	0,0023	0,0233326	yes
XLOC_006473	g461	66,974	65,2783	-0,036997	-0,061781	0,91085	0,962529	no
XLOC_006474	g462	387,221	174,021	-1,1539	-1,7884	0,002	0,0208936	yes
XLOC_006475	g466	90,0659	58,9115	-0,612431	-0,913623	0,1078	0,319291	no
XLOC_006476	g463	115,265	108,15	-0,0919191	-0,150821	0,793	0,904567	no
XLOC_006477	g464	299,783	266,32	-0,170761	-0,200351	0,7108	0,862876	no
XLOC_006478	g465	155,631	167,016	0,10186	0,178189	0,76165	0,887763	no
XLOC_006479	g9078	0	0,211781 inf		0	1	1	no
XLOC_006480	g9079	0	0	0	0	1	1	no
XLOC_006481	g3132	15,0492	8,76571	-0,779746	-0,949218	0,2221	0,479345	no
XLOC_006482	g3130	13,4942	16,8175	0,317629	0,482379	0,3915	0,646031	no
XLOC_006483	g3131	45,3722	40,7981	-0,153305	-0,245885	0,6602	0,833204	no
XLOC_006484	g3133	20,7696	27,8139	0,421335	0,706359	0,21585	0,471872	no
XLOC_006485	g3134	1066,58	663,732	-0,684324	-0,907058	0,10865	0,320596	no
XLOC_006486	g9080	0,121985	0,688742	2,49726	0	1	1	no
XLOC_006487	g9081	8,14738	9,96369	0,290344	0,434259	0,44275	0,689034	no
XLOC_006488	g9083	45,9433	49,0882	0,0955208	0,1671	0,7746	0,894738	no
XLOC_006489	g9082	58,0348	26,9709	-1,10551	-1,06642	0,07525	0,253495	no
XLOC_006490	g3135	0	0	0	0	1	1	no
XLOC_006491	g9084	122,049	130,105	0,09222	0,151806	0,78625	0,901303	no
XLOC_006492	g3136	4,24033	2,01283	-1,07495	-1,21087	0,04505	0,183261	no
XLOC_006493	g3137	19,6643	876,789	5,47858	6,58894	5,00E-05	0,00120049	yes
XLOC_006494	g3138	8,27803	10,7987	0,383492	0,535171	0,35125	0,611769	no
XLOC_006495	g9085	82,6041	167,265	1,01785	1,69773	0,00235	0,0236687	yes
XLOC_006496	g9086	6,01047	11,7385	0,965692	1,23889	0,0318	0,14654	no
XLOC_006497	g9087	0	0	0	0	1	1	no
XLOC_006498	g9089	58,035	55,5808	-0,0623349	-0,100525	0,8521	0,93347	no
XLOC_006499	g9088	67,7728	100,286	0,565345	0,915549	0,11635	0,33457	no
XLOC_006500	g9090	65,8837	72,9042	0,146082	0,157119	0,78655	0,901485	no
XLOC_006501	g3140	0	0	0	0	1	1	no
XLOC_006502	g3139	0	0	0	0	1	1	no
XLOC_006503	g9091	0	0	0	0	1	1	no
XLOC_006504	g9092	0	0	0	0	1	1	no
XLOC_006505	g9093	0,237659	0	#NAME?	0	1	1	no
XLOC_006506	g3141	15,897	44,7912	1,49446	2,17205	0,0001	0,00209829	yes
XLOC_006507	g3142	15,1198	16,4628	0,122764	0,157044	0,7764	0,895706	no
XLOC_006508	g3143	0	0	0	0	1	1	no
XLOC_006509	g9094	0	0	0	0	1	1	no
XLOC_006510	g9095	45,6413	351,936	2,9469	3,83779	5,00E-05	0,00120049	yes
XLOC_006511	g9096	105,318	70,4872	-0,579315	-0,894243	0,1265	0,351012	no
XLOC_006512	g467	0	0	0	0	1	1	no
XLOC_006513	g3144	0	0	0	0	1	1	no
XLOC_006514	g9097	0	0	0	0	1	1	no
XLOC_006515	g3145	3,6504	1,6602	-1,13669	-1,04266	0,12795	0,353338	no
XLOC_006516	g3146	585,106	512,681	-0,190636	-0,219974	0,68715	0,849743	no
XLOC_006517	g3147	88,9419	87,1198	-0,0298629	-0,0512138	0,929	0,968397	no
XLOC_006518	g3148	37,8779	19,0372	-0,992531	-1,56732	0,00765	0,0553596	no
XLOC_006519	g3149	103,944	70,1282	-0,567736	-0,934578	0,10825	0,319992	no
XLOC_006520	g3150	59,3951	52,9595	-0,165454	-0,275263	0,6314	0,816117	no
XLOC_006521	g3152	60,4333	88,4173	0,548985	0,948988	0,0994	0,302294	no
XLOC_006522	g3153	61,4717	103,707	0,754524	1,26485	0,0328	0,149882	no
XLOC_006523	g3155	101,754	121,917	0,260809	0,421784	0,4442	0,690525	no
XLOC_006524	g3157	34,6131	31,0294	-0,157682	-0,264937	0,6442	0,824348	no
XLOC_006525	g3159	183,168	135,698	-0,43277	-0,722475	0,19905	0,451802	no
XLOC_006526	g3161	11,1884	29,7071	1,40881	1,7558	0,00285	0,0272512	yes
XLOC_006527	g3164	52,0083	71,953	0,468312	0,784775	0,17295	0,418317	no
XLOC_006528	g3166	50,1208	32,6098	-0,620103	-0,913632	0,11245	0,327868	no
XLOC_006529	g3167	70,6629	87,3611	0,306039	0,479932	0,38735	0,643156	no
XLOC_006530	g3169	14,3273	123,587	3,10869	3,62337	5,00E-05	0,00120049	yes
XLOC_006531	g3171	44,4912	65,7562	0,563608	0,961305	0,08745	0,279635	no

XLOC_006532	g3173	121,483	313,378	1,36714	2,22499	0,0001	0,00209829	yes
XLOC_006533	g3175	39,9781	32,6455	-0,292326	-0,490799	0,37985	0,636866	no
XLOC_006534	g3177	37,9488	92,3434	1,28296	2,06086	0,00065	0,00901554	yes
XLOC_006535	g3151	118,489	177,533	0,583329	0,863742	0,1259	0,350039	no
XLOC_006536	g3154	38,0316	45,9002	0,271302	0,465979	0,4122	0,664882	no
XLOC_006537	g3156	74,5328	91,9633	0,303182	0,523401	0,35895	0,619938	no
XLOC_006538	g3158	236,073	136,901	-0,7861	-1,14991	0,043	0,177944	no
XLOC_006539	g3160	205,472	92,0303	-1,15876	-1,81464	0,0016	0,0176341	yes
XLOC_006540	g3162	7,83584	29,6288	1,91884	2,51065	5,00E-05	0,00120049	yes
XLOC_006541	g3163	35,5031	14,8263	-1,25979	-1,78076	0,0027	0,0261737	yes
XLOC_006542	g3165	44,3317	29,2483	-0,599985	-0,971628	0,08635	0,277473	no
XLOC_006543	g3168	26,6217	52,3656	0,976019	1,54546	0,0056	0,0440994	yes
XLOC_006544	g3170	80,2392	45,9959	-0,802802	-1,22438	0,03425	0,153718	no
XLOC_006545	g3172	276,965	185,402	-0,579044	-0,774754	0,17335	0,418975	no
XLOC_006546	g3174	81,9964	93,5613	0,190351	0,309527	0,59235	0,791197	no
XLOC_006547	g3176	30,9795	37,8977	0,290797	0,475069	0,4201	0,671172	no
XLOC_006548	g3178	84,0896	97,5965	0,214901	0,330666	0,55765	0,77025	no
XLOC_006549	g3179	3,75769	1,5705	-1,25862	-1,45393	0,0147	0,0874343	no
XLOC_006550	g3180	2,87162	1,35691	-1,08154	-0,734814	0,1864	0,434579	no
XLOC_006551	g3181	50,9642	55,0541	0,111366	0,16062	0,7813	0,898614	no
XLOC_006552	g3182	16,9279	12,0692	-0,488076	-0,759342	0,1838	0,431901	no
XLOC_006553	g9098	10,8755	13,1342	0,272245	0,363054	0,53245	0,752862	no
XLOC_006554	g3183	350,261	1082,81	1,62828	1,99996	0,00265	0,0257909	yes
XLOC_006555	g3185	9,12993	9,3369	0,032341	0,0459031	0,9377	0,972153	no
XLOC_006556	g3186	16,6733	15,583	-0,0975742	-0,154593	0,78375	0,900444	no
XLOC_006557	g3187	0,965365	0,949579	-0,023786	0	1	1	no
XLOC_006558	g3188	14,931	73,2574	2,29467	3,29905	5,00E-05	0,00120049	yes
XLOC_006559	g3184	2,36221	24,2423	3,35932	4,24446	5,00E-05	0,00120049	yes
XLOC_006560	g9099	12,9841	17,2056	0,406131	0,560704	0,30995	0,571052	no
XLOC_006561	g9100	0	0	0	0	1	1	no
XLOC_006562	g9101	0	0	0	0	1	1	no
XLOC_006563	g9102	12,2616	13,6128	0,150812	0,253266	0,65185	0,829167	no
XLOC_006564	g9106	363,472	280,733	-0,372643	-0,635104	0,2588	0,518762	no
XLOC_006565	g9107	36,5125	30,2503	-0,271444	-0,351094	0,555	0,768269	no
XLOC_006566	g9103	271,181	338,796	0,321159	0,533995	0,3617	0,621784	no
XLOC_006567	g9104	402,462	294,656	-0,44982	-0,732087	0,21425	0,469524	no
XLOC_006568	g9105	3,92668	0,532557	-2,8823	-1,90758	0,21265	0,468106	no
XLOC_006569	g9108	32,4564	36,8217	0,182056	0,285731	0,62585	0,812405	no
XLOC_006570	g9109	0	0	0	0	1	1	no
XLOC_006571	g9110	0	0	0	0	1	1	no
XLOC_006572	g3190	170,675	107,681	-0,664495	-0,98686	0,0815	0,268387	no
XLOC_006573	g3191	54,1875	46,4603	-0,22196	-0,345358	0,5334	0,753234	no
XLOC_006574	g3192	45,5865	76,6929	0,750485	1,26225	0,02905	0,138548	no
XLOC_006575	g3196	44,6726	49,1104	0,136639	0,228589	0,6994	0,857228	no
XLOC_006576	g3189	43,3393	47,1276	0,120897	0,185523	0,7388	0,876527	no
XLOC_006577	g3193	27,4004	46,1492	0,752109	1,27753	0,0278	0,135415	no
XLOC_006578	g3194	107,562	146,129	0,442073	0,737414	0,1921	0,442199	no
XLOC_006579	g3195	14,3595	8,00445	-0,843132	-1,23523	0,0264	0,131198	no
XLOC_006580	g3197	19,1923	25,5541	0,413023	0,609168	0,27515	0,536851	no
XLOC_006581	g3198	229,663	212,199	-0,114104	-0,170551	0,75515	0,884596	no
XLOC_006582	g9111	56,2464	51,6842	-0,122036	-0,202096	0,72145	0,868216	no
XLOC_006583	g9112	324,101	281,902	-0,201253	-0,314729	0,5707	0,777831	no
XLOC_006584	g9113	0	0	0	0	1	1	no
XLOC_006585	g3199	1,08652	1,63099	0,586025	0,501371	0,3546	0,615463	no
XLOC_006586	g3200	0	0	0	0	1	1	no
XLOC_006587	g9114	0	0	0	0	1	1	no
XLOC_006588	g9115	57,738	64,7347	0,165018	0,281395	0,6162	0,807415	no
XLOC_006589	g9116	22,5143	23,6072	0,0683848	0,102086	0,8561	0,934827	no
XLOC_006590	g3201	3,60492	5,58189	0,630789	0,827769	0,15075	0,388038	no
XLOC_006591	g3202	0	0	0	0	1	1	no
XLOC_006592	g9117	0	0	0	0	1	1	no
XLOC_006593	g9118	0	0	0	0	1	1	no
XLOC_006594	g3203	8,03652	8,93119	0,152282	0,178909	0,7358	0,874871	no
XLOC_006595	g3204	17,8063	60,3608	1,76122	2,70025	5,00E-05	0,00120049	yes
XLOC_006596	g3205	23,225	30,4943	0,392864	0,662153	0,22695	0,485439	no
XLOC_006597	g3206	87,1941	146,743	0,750986	1,25032	0,02545	0,127967	no
XLOC_006598	g9119	52,8053	33,9374	-0,637805	-1,00527	0,0755	0,253989	no
XLOC_006599	g3207	22,718	24,049	0,0821435	0,137415	0,8078	0,911374	no
XLOC_006600	g3208	0	0	0	0	1	1	no
XLOC_006601	g9120	42,1925	31,8742	-0,404598	-0,683803	0,2189	0,476101	no
XLOC_006602	g9121	30,0593	24,2803	-0,308022	-0,48304	0,37735	0,635496	no
XLOC_006603	g9124	272,731	267,981	-0,0253479	-0,0415247	0,944	0,975337	no
XLOC_006604	g9125	3,00756	2,36114	-0,349112	-0,390373	0,50535	0,733344	no
XLOC_006605	g9126	3,84892	2,52783	-0,606556	-0,709027	0,21425	0,469524	no
XLOC_006606	g9128	63,162	66,124	0,0661173	0,102392	0,85595	0,934827	no
XLOC_006607	g9122	15,5349	11,8101	-0,395491	-0,559892	0,31825	0,578529	no
XLOC_006608	g9123	11,6822	10,3956	-0,168328	-0,22495	0,69665	0,855994	no
XLOC_006609	g9127	20,6659	22,8327	0,143854	0,209451	0,71445	0,865069	no
XLOC_006610	g9129	14,9504	32,5517	1,12255	0,973304	0,0914	0,287676	no
XLOC_006611	g9130	42,0718	78,9526	0,908133	1,36159	0,01985	0,108293	no
XLOC_006612	g9131	35,316	12,8657	-1,45679	-2,13693	0,0004	0,0061761	yes
XLOC_006613	g9132	17,7089	32,715	0,885478	1,41671	0,01445	0,0864711	no
XLOC_006614	g9134	28,5556	64,5785	1,17728	1,52402	0,0078	0,0560322	no
XLOC_006615	g9133	164,556	197,799	0,265456	0,406898	0,47955	0,715246	no
XLOC_006616	g9135	10,788	16,0097	0,569513	0,596127	0,30195	0,563717	no
XLOC_006617	g9136	6,26671	11,8758	0,922246	1,05464	0,0724	0,248416	no
XLOC_006618	g9137	0	0	0	0	1	1	no
XLOC_006619	g9138	25,7666	16,7797	-0,61878	-0,830908	0,15665	0,394842	no
XLOC_006620	g9139	60,7915	45,536	-0,416861	-0,637342	0,27195	0,53315	no
XLOC_006621	g9140	169,024	127,433	-0,407495	-0,47486	0,3764	0,634524	no
XLOC_006622	g9141	3,06481	2,19435	-0,482004	-0,55512	0,3331	0,593979	no
XLOC_006623	g9142	34,6789	33,3541	-0,0561922	-0,0911784	0,87215	0,942536	no

XLOC_006624	g9143	132,631	153,891	0,214496	0,365014	0,5165	0,741989	no
XLOC_006625	g9144	5,60388	4,89846	-0,194097	-0,222564	0,6976	0,856213	no
XLOC_006626	g3209	102,668	77,2775	-0,409868	-0,68074	0,2257	0,483713	no
XLOC_006627	g3210	13,8279	18,6986	0,435341	0,668248	0,22935	0,487604	no
XLOC_006628	g3211	7,17957	8,44722	0,234579	0,293945	0,6029	0,798819	no
XLOC_006629	g3214	35,3006	47,5456	0,42962	0,72307	0,2004	0,45251	no
XLOC_006630	g3212	1630,55	1144,49	-0,51065	-0,757654	0,1719	0,416907	no
XLOC_006631	g3213	79,7773	117,933	0,563921	0,960115	0,0979	0,299962	no
XLOC_006632	g9145	479,624	103,698	-2,20951	-3,11323	5,00E-05	0,00120049	yes
XLOC_006633	g9146	0	0	0	0	1	1	no
XLOC_006634	g9147	71,5063	52,2655	-0,452211	-0,71885	0,2128	0,468332	no
XLOC_006635	g9148	138,512	224,907	0,699319	1,19796	0,034	0,153226	no
XLOC_006636	g9149	84,9273	51,8318	-0,712391	-1,07917	0,0594	0,218877	no
XLOC_006637	g3215	63,4158	56,8184	-0,158484	-0,270443	0,6316	0,816117	no
XLOC_006638	g3218	77,0054	74,0156	-0,0571304	-0,0960541	0,8695	0,94088	no
XLOC_006639	g3216	36,2521	29,5921	-0,292853	-0,478733	0,38835	0,643536	no
XLOC_006640	g3217	285,357	327,1	0,196964	0,282433	0,61955	0,808526	no
XLOC_006641	g9150	0	0	0	0	1	1	no
XLOC_006642	g9151	0	0	0	0	1	1	no
XLOC_006643	g3220	25,5649	21,3997	-0,256575	-0,314908	0,57635	0,780604	no
XLOC_006644	g3223	78,3718	44,5108	-0,81618	-1,08464	0,06335	0,227874	no
XLOC_006645	g3225	9,18448	11,7216	0,351903	0,491496	0,3881	0,643448	no
XLOC_006646	g3226	86,6595	97,3967	0,168515	0,291412	0,6202	0,808752	no
XLOC_006647	g3228	45,7352	54,921	0,264053	0,455311	0,4229	0,672849	no
XLOC_006648	g3219	2351,08	607,384	-1,95265	-3,21938	5,00E-05	0,00120049	yes
XLOC_006649	g3221	9,89399	4,73715	-1,06253	-1,26135	0,03575	0,158066	no
XLOC_006650	g3222	20,1337	17,3635	-0,21356	-0,298049	0,60955	0,803245	no
XLOC_006651	g3224	57,2001	66,7772	0,223337	0,319827	0,57735	0,781041	no
XLOC_006652	g3227	65,0538	130,724	1,00681	1,7311	0,00235	0,0236687	yes
XLOC_006653	g3229	189,716	204,215	0,10625	0,164351	0,7726	0,894131	no
XLOC_006654	g9152	4,06003	1,34929	-1,58929	-1,41395	0,0547	0,208199	no
XLOC_006655	g9153	9,38624	6,24486	-0,587877	-0,553934	0,33445	0,594729	no
XLOC_006656	g9154	0	0	0	0	1	1	no
XLOC_006657	g9155	11,1508	17,218	0,626771	0,914606	0,1097	0,322918	no
XLOC_006658	g9156	0	0	0	0	1	1	no
XLOC_006659	g9159	52,0529	115,992	1,15598	1,52567	0,0083	0,0585532	no
XLOC_006660	g9157	204,771	226,608	0,146187	0,220106	0,6975	0,856213	no
XLOC_006661	g9158	61,8714	63,8356	0,04509	0,078174	0,89485	0,95329	no
XLOC_006662	g9160	13,375	29,0886	1,12091	1,76919	0,00145	0,0164423	yes
XLOC_006663	g9161	9,15802	14,2029	0,633078	0,838017	0,1475	0,384102	no
XLOC_006664	g9162	0	0	0	0	1	1	no
XLOC_006665	g9164	24,1215	12,977	-0,894357	-1,37187	0,0175	0,0984814	no
XLOC_006666	g9163	70,0363	69,397	-0,0132285	-0,0188902	0,9735	0,98758	no
XLOC_006667	g9165	22,9634	28,7015	0,321788	0,486738	0,3873	0,643156	no
XLOC_006668	g9166	68,8496	62,7666	-0,133451	-0,200403	0,72365	0,86956	no
XLOC_006669	g9167	0	0	0	0	1	1	no
XLOC_006670	g9169	62,1831	51,4681	-0,272844	-0,421811	0,4599	0,701766	no
XLOC_006671	g9168	117,938	56,7704	-1,05482	-1,73732	0,00225	0,0229201	yes
XLOC_006672	g9170	130,404	189,792	0,541434	0,809746	0,1562	0,394112	no
XLOC_006673	g9171	26,4573	29,7904	0,171185	0,228692	0,6914	0,852637	no
XLOC_006674	g9172	166,535	123,831	-0,42746	-0,685741	0,2396	0,497749	no
XLOC_006675	g9173	72,4797	83,6808	0,207319	0,269818	0,62945	0,814495	no
XLOC_006676	g9174	8,30954	11,5629	0,476663	0,621843	0,27655	0,538183	no
XLOC_006677	g3230	21,573	18,1076	-0,252635	-0,417175	0,46425	0,705281	no
XLOC_006678	g3231	18,1701	6,65815	-1,44837	-2,07941	0,00045	0,00669545	yes
XLOC_006679	g9175	82,8495	91,6925	0,146311	0,240693	0,65605	0,831493	no
XLOC_006680	g9176	0	0	0	0	1	1	no
XLOC_006681	g9181	66,8005	134,924	1,01421	1,59577	0,00485	0,0400901	yes
XLOC_006682	g9183	2,3173	2,50323	0,111349	0,128566	0,82785	0,921598	no
XLOC_006683	g9185	52,9426	44,0655	-0,264779	-0,437468	0,44285	0,68908	no
XLOC_006684	g9186	66,1488	77,1486	0,221925	0,350385	0,5294	0,750283	no
XLOC_006685	g9188	35,6823	28,0881	-0,34525	-0,586331	0,3151	0,57568	no
XLOC_006686	g9177	161,523	188,673	0,22415	0,384179	0,4964	0,726367	no
XLOC_006687	g9178	132,901	161,425	0,280507	0,461921	0,407	0,660291	no
XLOC_006688	g9179	101,92	60,0422	-0,763387	-1,03548	0,0572	0,214227	no
XLOC_006689	g9180	39,3315	48,6094	0,305552	0,496523	0,3843	0,640475	no
XLOC_006690	g9182	2,0764	1,5963	-0,379357	-0,343367	0,54965	0,765215	no
XLOC_006691	g9184	28,9486	20,295	-0,512368	-0,876811	0,12525	0,348824	no
XLOC_006692	g9187	150,251	226,402	0,591511	0,932663	0,09645	0,297002	no
XLOC_006693	g9189	5,05715	12,1423	1,26365	1,70345	0,00285	0,0272512	yes
XLOC_006694	g9190	38,319	26,6472	-0,524078	-0,867192	0,11925	0,33943	no
XLOC_006695	g9191	178,521	184,977	0,0512475	0,0864025	0,8862	0,949224	no
XLOC_006696	g468	8,21621	7,84931	-0,0659083	-0,0897837	0,87295	0,942536	no
XLOC_006697	g469	0,331521	0,33831	0,0292463	0	1	1	no
XLOC_006698	g28	7,53286	8,19846	0,122155	0,169817	0,77005	0,893264	no
XLOC_006699	g30	38,3492	21,3429	-0,845436	-1,11468	0,05025	0,19754	no
XLOC_006700	g27	15,3427	17,788	0,213352	0,224134	0,69395	0,854464	no
XLOC_006701	g29	47,3869	67,4325	0,508954	0,808265	0,14945	0,387025	no
XLOC_006702	g9192	1,47967	0,575186	-1,36317	-0,834556	0,16665	0,409843	no
XLOC_006703	g3232	21,6028	24,7239	0,194688	0,333367	0,56805	0,776676	no
XLOC_006704	g3234	12,7116	22,5048	0,824094	1,34271	0,01745	0,0982563	no
XLOC_006705	g3233	19,9742	27,5576	0,464309	0,68332	0,23645	0,49424	no
XLOC_006706	g3235	113,382	60,931	-0,895938	-1,25832	0,02485	0,125917	no
XLOC_006707	g3237	55,695	72,312	0,376688	0,647862	0,2576	0,517837	no
XLOC_006708	g3236	162,981	98,7236	-0,723239	-1,21626	0,03695	0,161625	no
XLOC_006709	g3238	93,6352	113,779	0,281106	0,403128	0,47815	0,713917	no
XLOC_006710	g3239	127,78	115,047	-0,151443	-0,258872	0,64765	0,826393	no
XLOC_006711	g9193	38,3512	31,4642	-0,285562	-0,459164	0,4195	0,670927	no
XLOC_006712	g471	109,965	124,127	0,174775	0,291041	0,61945	0,808526	no
XLOC_006713	g470	73,1844	60,3958	-0,277087	-0,466091	0,42445	0,673687	no
XLOC_006714	g472	66,1383	53,7931	-0,298066	-0,483965	0,3896	0,644629	no
XLOC_006715	g9194	52,5732	25,392	-1,04995	-1,56294	0,0059	0,0459825	yes

XLOC_006716	g9195	50,1768	73,436	0,549469	0,735117	0,20925	0,464271	no
XLOC_006717	g9196	20,5732	22,6716	0,140119	0,218108	0,702	0,858715	no
XLOC_006718	g9197	103,08	117,657	0,190832	0,317778	0,57465	0,780319	no
XLOC_006719	g9198	120,176	141,07	0,231258	0,3647	0,5114	0,737546	no
XLOC_006720	g9199	8,11231	7,07701	-0,196972	-0,171138	0,76335	0,888537	no
XLOC_006721	g9200	0	2,16637	inf	0	1	1	no
XLOC_006722	g9201	0	0	0	0	1	1	no
XLOC_006723	g3240	0	0	0	0	1	1	no
XLOC_006724	g3241	13,8281	14,0014	0,0179676	0,0254448	0,9623	0,983533	no
XLOC_006725	g3242	33,9908	31,517	-0,109016	-0,174932	0,75155	0,883013	no
XLOC_006726	g9202	11,1138	4,75265	-1,22555	-1,06626	0,0689	0,240525	no
XLOC_006727	g3243	70,8993	58,3186	-0,281814	-0,410363	0,4572	0,701012	no
XLOC_006728	g3244	20,2949	12,7115	-0,674989	-1,01743	0,0704	0,243769	no
XLOC_006729	g3245	14,861	25,3277	0,769186	1,19168	0,0384	0,165826	no
XLOC_006730	g3246	7,85736	5,49557	-0,515777	-0,731872	0,2112	0,466588	no
XLOC_006731	g3249	24,6975	30,816	0,319312	0,443823	0,44175	0,688351	no
XLOC_006732	g3252	41,3494	26,1292	-0,662205	-1,11264	0,0582	0,215995	no
XLOC_006733	g3247	2,59008	0,946118	-1,4529	-1,08756	0,1488	0,386054	no
XLOC_006734	g3248	3,03162	2,9015	-0,0632907	-0,080561	0,8925	0,951872	no
XLOC_006735	g3250	16,8005	9,95254	-0,755368	-1,19147	0,03965	0,169068	no
XLOC_006736	g3251	18,4106	21,6062	0,230911	0,385032	0,5056	0,733381	no
XLOC_006737	g3256	54,9032	72,2147	0,395403	0,684645	0,2343	0,491629	no
XLOC_006738	g3253	196,888	402,653	1,03216	1,49837	0,01	0,0665312	no
XLOC_006739	g3254	24,0187	10,5744	-1,18358	-1,78685	0,00255	0,0250912	yes
XLOC_006740	g3255	13,4008	7,02836	-0,931058	-1,27758	0,02795	0,135891	no
XLOC_006741	g3257	110,019	115,692	0,0725352	0,122238	0,8319	0,922663	no
XLOC_006742	g9203	5,63817	2,94864	-0,935176	-0,983693	0,08845	0,281458	no
XLOC_006743	g9204	6,56309	4,60025	-0,512663	-0,667873	0,24105	0,499282	no
XLOC_006744	g3258	0	0	0	0	1	1	no
XLOC_006745	g3259	12,4055	7,57158	-0,712311	-1,01245	0,0831	0,271381	no
XLOC_006746	g9205	95,5151	106,901	0,162477	0,276445	0,63815	0,820025	no
XLOC_006747	g9206	20,5793	9,71578	-1,08279	-1,32754	0,01765	0,0992118	no
XLOC_006748	g9207	0	0	0	0	1	1	no
XLOC_006749	g9208	394,939	537,191	0,443808	0,729452	0,1964	0,447606	no
XLOC_006750	g3260	8,49571	10,0712	0,245429	0,263194	0,6516	0,829167	no
XLOC_006751	g9209	68,8157	69,8536	0,0215957	0,0349717	0,9482	0,976439	no
XLOC_006752	g9211	0	0	0	0	1	1	no
XLOC_006753	g9210	13,62	4,21551	-1,69195	-1,83486	0,0039	0,0342252	yes
XLOC_006754	g9212	132,924	59,8594	-1,15095	-1,46414	0,0095	0,0641609	no
XLOC_006755	g9213	77,0275	60,1598	-0,356572	-0,592916	0,3001	0,562226	no
XLOC_006756	g9214	354,64	314,535	-0,173135	-0,296433	0,59705	0,794233	no
XLOC_006757	g9216	60,8614	64,5346	0,0845471	0,134056	0,8261	0,920883	no
XLOC_006758	g9215	63,2958	29,4323	-1,10471	-1,78267	0,00355	0,031924	yes
XLOC_006759	g9217	255,557	200,399	-0,350773	-0,532518	0,3606	0,621026	no
XLOC_006760	g9218	44,6899	133,48	1,57861	1,35567	0,02985	0,141402	no
XLOC_006761	g9219	245,48	342,104	0,478829	0,753708	0,1791	0,426373	no
XLOC_006762	g9220	109,856	114,27	0,0568426	0,0905608	0,8726	0,942536	no
XLOC_006763	g3261	0	0	0	0	1	1	no
XLOC_006764	g3262	0	0	0	0	1	1	no
XLOC_006765	g9221	0	0	0	0	1	1	no
XLOC_006766	g3263	0	0	0	0	1	1	no
XLOC_006767	g3264	1,97632	3,16619	0,67993	0,645189	0,2591	0,51904	no
XLOC_006768	g3265	1892,13	2843,5	0,587654	0,507232	0,37945	0,636522	no
XLOC_006769	g3267	32,248	11,6784	-1,46537	-2,37332	5,00E-05	0,00120049	yes
XLOC_006770	g3268	0,182091	0,214206	0,234339	0	1	1	no
XLOC_006771	g3269	45,8213	41,3503	-0,148121	-0,250965	0,65215	0,829397	no
XLOC_006772	g3266	7,4274	3,25728	-1,18919	-1,49449	0,00955	0,0643217	no
XLOC_006773	g3270	0	0	0	0	1	1	no
XLOC_006774	g3272	34,4386	29,6596	-0,21553	-0,288209	0,6114	0,804495	no
XLOC_006775	g3273	132,907	114,586	-0,213986	-0,316814	0,5902	0,790152	no
XLOC_006776	g3274	36,0285	332,66	3,20684	4,10766	5,00E-05	0,00120049	yes
XLOC_006777	g3271	2,66131	3,37259	0,34172	0,388748	0,50125	0,730308	no
XLOC_006778	g3275	14,024	15,7796	0,170167	0,236316	0,6802	0,846112	no
XLOC_006779	g9222	0	0	0	0	1	1	no
XLOC_006780	g9223	0	0	0	0	1	1	no
XLOC_006781	g9224	7,50203	11,1136	0,566979	0,70675	0,21055	0,465571	no
XLOC_006782	g9225	15,6353	17,0255	0,122896	0,158902	0,78125	0,898614	no
XLOC_006783	g3276	22,7095	17,8809	-0,344874	-0,516984	0,35475	0,615463	no
XLOC_006784	g3277	3,37463	3,19015	-0,081103	-0,0894165	0,87925	0,945715	no
XLOC_006785	g3279	58,1908	44,5477	-0,385442	-0,661166	0,24625	0,505152	no
XLOC_006786	g3281	25,4807	29,7673	0,224322	0,387122	0,4971	0,726923	no
XLOC_006787	g3283	30,7801	27,1866	-0,179105	-0,285479	0,61025	0,803802	no
XLOC_006788	g3284	2,09806	5,00272	1,25366	1,52779	0,0066	0,0498938	yes
XLOC_006789	g3287	292,901	150,351	-0,962076	-1,02172	0,0842	0,273699	no
XLOC_006790	g3288	6,02394	4,11795	-0,54878	-0,693067	0,2297	0,487604	no
XLOC_006791	g3289	14,3501	15,7937	0,138282	0,174488	0,758	0,8854	no
XLOC_006792	g3278	18,2949	14,0154	-0,38443	-0,598605	0,2819	0,54429	no
XLOC_006793	g3280	44,0239	36,2868	-0,278843	-0,46229	0,4146	0,666673	no
XLOC_006794	g3282	36,2065	29,7673	-0,282519	-0,389756	0,49875	0,728503	no
XLOC_006795	g3285	4,78451	12,671	1,40509	1,94094	0,001	0,0126221	yes
XLOC_006796	g3286	58,6682	52,7537	-0,153307	-0,248352	0,6537	0,829883	no
XLOC_006797	g9226	0	0	0	0	1	1	no
XLOC_006798	g9227	209,383	164,41	-0,348845	-0,529335	0,344	0,604287	no
XLOC_006799	g9228	0	0	0	0	1	1	no
XLOC_006800	g3290	42,3744	71,9808	0,764417	1,28784	0,0255	0,128087	no
XLOC_006801	g3291	88,3383	109,793	0,313681	0,523032	0,34155	0,601706	no
XLOC_006802	g3293	51,3548	114,616	1,15824	1,96302	0,0005	0,00733931	yes
XLOC_006803	g3294	191,178	366,099	0,937315	1,49149	0,0087	0,0603347	no
XLOC_006804	g3292	9,35961	103,265	3,46376	4,27965	5,00E-05	0,00120049	yes
XLOC_006805	g9229	0	0	0	0	1	1	no
XLOC_006806	g3297	4,04607	8,68294	1,10166	1,27929	0,03385	0,153042	no
XLOC_006807	g3299	116,022	155,06	0,418433	0,672868	0,23625	0,494137	no

XLOC_006808	g3295	20,7841	21,046	0,0180629	0,0234388	0,9694	0,986579	no
XLOC_006809	g3296	0,436264	0,211781	-1,04263	0	1	1	no
XLOC_006810	g3298	81,5281	48,6697	-0,744274	-1,25852	0,0319	0,146794	no
XLOC_006811	g9230	3,41691	13,4112	1,97267	2,20703	0,00135	0,0156148	yes
XLOC_006812	g9231	0	0	0	0	1	1	no
XLOC_006813	g3301	9,41177	23,3869	1,31316	2,0722	0,0002	0,00367103	yes
XLOC_006814	g3300	46,6533	60,8856	0,384121	0,447917	0,4503	0,694947	no
XLOC_006815	g9232	4,17957	1,90056	-1,13693	-1,0144	0,20185	0,454105	no
XLOC_006816	g9233	148,471	121,065	-0,294394	-0,494261	0,3954	0,64952	no
XLOC_006817	g3302	0	0	0	0	1	1	no
XLOC_006818	g9234	0	0	0	0	1	1	no
XLOC_006819	g9235	55,1515	48,1285	-0,196509	-0,226048	0,69505	0,854884	no
XLOC_006820	g9236	0	0,148707	inf	0	1	1	no
XLOC_006821	g3303	0	0	0	0	1	1	no
XLOC_006822	g9237	7,35874	3,10978	-1,24265	-1,526	0,01055	0,0690213	no
XLOC_006823	g9238	36,8118	47,5789	0,370154	0,601839	0,3023	0,564048	no
XLOC_006824	g9239	83,7528	43,865	-0,933066	-1,21302	0,027	0,133036	no
XLOC_006825	g9240	0	0	0	0	1	1	no
XLOC_006826	g9241	6,30269	5,54162	-0,185658	-0,231801	0,6931	0,853876	no
XLOC_006827	g3304	26,4514	12,2444	-1,11122	-1,02812	0,0934	0,291263	no
XLOC_006828	g3305	73,8148	45,1207	-0,71012	-1,11442	0,04575	0,185188	no
XLOC_006829	g3306	45,0913	40,1857	-0,166166	-0,287198	0,60975	0,803401	no
XLOC_006830	g31	43,3555	45,684	0,0754766	0,129861	0,8233	0,919418	no
XLOC_006831	g33	186,518	161,813	-0,204992	-0,241726	0,66	0,833166	no
XLOC_006832	g34	174,413	159,295	-0,130813	-0,178881	0,73735	0,875865	no
XLOC_006833	g35	35,0041	30,8823	-0,180745	-0,248631	0,64875	0,827152	no
XLOC_006834	g32	30,0338	13,25	-1,1806	-1,81033	0,00225	0,0229201	yes
XLOC_006835	g36	103,08	96,8414	-0,0900663	-0,145392	0,7916	0,904002	no
XLOC_006836	g9242	0	0	0	0	1	1	no
XLOC_006837	g9243	50,655	35,9711	-0,493867	-0,837347	0,1396	0,372621	no
XLOC_006838	g9245	0,478211	0,0882145	-2,43856	0	1	1	no
XLOC_006839	g9244	14,2369	9,33064	-0,609586	-0,873712	0,12105	0,342963	no
XLOC_006840	g9246	15,4424	19,0028	0,299313	0,448238	0,43405	0,681108	no
XLOC_006841	g9247	0	0	0	0	1	1	no
XLOC_006842	g3308	8,03602	14,9702	0,897543	1,2593	0,0287	0,137614	no
XLOC_006843	g3307	3,26123	4,76041	0,54567	0,650226	0,27155	0,532898	no
XLOC_006844	g3309	2,60208	2,95427	0,183134	0,219142	0,7033	0,859113	no
XLOC_006845	g9248	5,01987	12,0313	1,26107	1,27718	0,11335	0,329709	no
XLOC_006846	g9249	11,5877	6,5022	-0,833598	-1,18105	0,0324	0,148469	no
XLOC_006847	g9250	119,823	66,289	-0,854062	-1,43294	0,0117	0,0743169	no
XLOC_006848	g9251	25,7022	30,5987	0,25158	0,392385	0,4907	0,722873	no
XLOC_006849	g9253	172,109	302,406	0,81316	1,36496	0,0178	0,0998264	no
XLOC_006850	g9255	84,9807	75,1315	-0,177716	-0,307245	0,593	0,791419	no
XLOC_006851	g9252	23,6055	33,0832	0,486971	0,836296	0,1381	0,370429	no
XLOC_006852	g9254	48,8772	112,9	1,20781	1,92304	0,0008	0,0106019	yes
XLOC_006853	g9256	15,8851	13,5145	-0,233167	-0,333263	0,5694	0,777354	no
XLOC_006854	g9257	61,6716	70,8482	0,200124	0,340868	0,54415	0,761477	no
XLOC_006855	g9258	18,5235	25,5885	0,466142	0,724328	0,2017	0,453871	no
XLOC_006856	g3310	0	0,0532262	inf	0	1	1	no
XLOC_006857	g9259	0	0	0	0	1	1	no
XLOC_006858	g9260	0	0	0	0	1	1	no
XLOC_006859	g9261	0	0	0	0	1	1	no
XLOC_006860	g9262	10,0137	25,2001	1,33146	1,84844	0,00185	0,01964	yes
XLOC_006861	g3311	0,175812	1,557	3,14666	1,71437	0,03095	0,144179	no
XLOC_006862	g3312	7,25136	7,46827	0,042523	0,0396616	0,9482	0,976439	no
XLOC_006863	g3313	29,6103	39,0955	0,400901	0,653846	0,24445	0,503031	no
XLOC_006864	g3314	6247,72	4388,11	-0,509729	-0,70524	0,2243	0,481975	no
XLOC_006865	g3315	24,2271	21,1283	-0,197445	-0,272301	0,6322	0,816117	no
XLOC_006866	g3316	89,8671	94,8478	0,0778207	0,128148	0,82195	0,918997	no
XLOC_006867	g9263	0	0	0	0	1	1	no
XLOC_006868	g473	0	0	0	0	1	1	no
XLOC_006869	g9264	0,364526	0	#NAME?	0	1	1	no
XLOC_006870	g9265	20,6534	10,469	-0,980253	-1,16978	0,04925	0,1947	no
XLOC_006871	g9266	32,0529	15,9891	-1,00336	-1,60197	0,0048	0,0398445	yes
XLOC_006872	g3317	69,0355	20,509	-1,75108	-2,53788	5,00E-05	0,00120049	yes
XLOC_006873	g3318	17,7109	22,0336	0,315071	0,535832	0,3375	0,597377	no
XLOC_006874	g3319	89,1615	59,173	-0,591481	-0,774443	0,17955	0,426507	no
XLOC_006875	g9267	218,663	159,536	-0,454823	-0,700481	0,21865	0,475768	no
XLOC_006876	g9268	0	0	0	0	1	1	no
XLOC_006877	g9269	543,303	327,89	-0,728544	-1,23316	0,0322	0,147897	no
XLOC_006878	g9270	70,9315	67,565	-0,0701515	-0,11697	0,8441	0,929163	no
XLOC_006879	g9271	20,4842	13,6105	-0,589798	-0,827416	0,152	0,389904	no
XLOC_006880	g3320	0	0	0	0	1	1	no
XLOC_006881	g3321	30,5286	45,2878	0,568961	0,736094	0,19845	0,451315	no
XLOC_006882	g9272	11,6291	4,59473	-1,33969	-1,13705	0,23165	0,48931	no
XLOC_006883	g3322	145,948	110,202	-0,40531	-0,488722	0,40625	0,659809	no
XLOC_006884	g3324	9,91208	2,50049	-1,98697	-1,83536	0,09965	0,302738	no
XLOC_006885	g3327	55,7331	74,2721	0,414286	0,690743	0,2135	0,469241	no
XLOC_006886	g3329	22,1189	30,025	0,440886	0,705402	0,2166	0,472774	no
XLOC_006887	g3330	7,21416	10,603	0,555566	0,767906	0,1822	0,42989	no
XLOC_006888	g3331	9,49688	8,32413	-0,190156	-0,249061	0,65825	0,832348	no
XLOC_006889	g3332	16,7208	12,1909	-0,455839	-0,590389	0,294	0,556265	no
XLOC_006890	g3323	250,786	275,179	0,133915	0,20464	0,7162	0,865564	no
XLOC_006891	g3325	3,47841	2,39249	-0,539914	-0,605947	0,29325	0,555608	no
XLOC_006892	g3326	42,0654	73,6068	0,807204	1,24488	0,02895	0,138206	no
XLOC_006893	g3328	125,34	210,372	0,747102	1,23589	0,0342	0,153704	no
XLOC_006894	g9273	13,2253	13,4054	0,019508	0,0258394	0,96505	0,984192	no
XLOC_006895	g9275	30,8228	13,5234	-1,18854	-1,30002	0,0261	0,130103	no
XLOC_006896	g9274	152,212	87,4606	-0,799378	-1,29786	0,0334	0,151566	no
XLOC_006897	g9276	14,7574	24,8962	0,754488	1,14339	0,05185	0,201172	no
XLOC_006898	g9277	52,6901	39,4339	-0,418094	-0,644476	0,24955	0,508092	no
XLOC_006899	g9278	0	0	0	0	1	1	no

XLOC_006900	g9279	4,49677	3,26212	-0,463077	-0,556463	0,34005	0,600141	no
XLOC_006901	g9280	7,09865	5,50205	-0,367576	-0,380346	0,4989	0,728578	no
XLOC_006902	g9281	0	0	0	0	1	1	no
XLOC_006903	g3333	2,54895	6,83305	1,42263	1,13475	0,072	0,247476	no
XLOC_006904	g9282	42,3659	64,8811	0,614895	1,00056	0,06925	0,241147	no
XLOC_006905	g9283	0	0	0	0	1	1	no
XLOC_006906	g9284	0,192224	1,35545	2,81791	0	1	1	no
XLOC_006907	g3334	27,7133	16,6958	-0,731091	-1,16288	0,03705	0,161847	no
XLOC_006908	g3336	21,5624	58,4649	1,43905	1,90407	0,00155	0,0171215	yes
XLOC_006909	g3337	2,73186	2,53373	-0,108618	-0,122352	0,8258	0,920785	no
XLOC_006910	g3335	60,1012	152,766	1,34586	2,18096	0,0003	0,00496796	yes
XLOC_006911	g9285	21,1579	15,984	-0,404563	-0,515239	0,3687	0,628691	no
XLOC_006912	g3338	0,739898	0,575546	-0,362395	0	1	1	no
XLOC_006913	g9286	33,7673	39,8657	0,23952	0,376644	0,5164	0,741989	no
XLOC_006914	g9287	21,3811	22,0094	0,0417839	0,0597	0,91145	0,962529	no
XLOC_006915	g9288	0	0	0	0	1	1	no
XLOC_006916	g9289	10,0448	18,1225	0,851328	0,9629	0,09745	0,299237	no
XLOC_006917	g9290	0	0	0	0	1	1	no
XLOC_006918	g9292	0	0	0	0	1	1	no
XLOC_006919	g9291	0	0	0	0	1	1	no
XLOC_006920	g3340	81,961	28,9356	-1,50209	-2,32961	0,0001	0,00209829	yes
XLOC_006921	g3342	3,8096	8,01449	1,07297	1,3272	0,0206	0,111394	no
XLOC_006922	g3343	24,7422	16,9099	-0,54911	-0,860967	0,1405	0,374038	no
XLOC_006923	g3339	81,4667	67,3308	-0,274944	-0,450745	0,43745	0,68432	no
XLOC_006924	g3341	194,681	198,248	0,0261923	0,0441053	0,94185	0,974396	no
XLOC_006925	g3344	31,6313	31,8795	0,0112757	0,0184947	0,97595	0,988227	no
XLOC_006926	g9293	22,3179	22,9446	0,0399529	0,0552827	0,9199	0,964696	no
XLOC_006927	g3345	1,71845	4,53225	1,39912	1,38684	0,01885	0,10411	no
XLOC_006928	g3346	22,5966	21,9965	-0,0388296	-0,0479601	0,9342	0,971293	no
XLOC_006929	g3347	36,0784	41,4485	0,200185	0,286037	0,6201	0,808752	no
XLOC_006930	g3348	30,9026	11,3575	-1,44408	-1,98101	0,00075	0,0100752	yes
XLOC_006931	g3349	90,0172	72,591	-0,31041	-0,511586	0,37265	0,631044	no
XLOC_006932	g9294	12,9449	19,7431	0,608956	0,887194	0,1302	0,35734	no
XLOC_006933	g9295	39,6979	20,6354	-0,943944	-1,36583	0,02035	0,110224	no
XLOC_006934	g9296	309,712	174,816	-0,825085	-1,34106	0,01605	0,0927124	no
XLOC_006935	g9297	26,212	10,4268	-1,32993	-2,07836	0,0003	0,00496796	yes
XLOC_006936	g3350	90,0154	88,5382	-0,0238722	-0,0377883	0,9456	0,975552	no
XLOC_006937	g9298	0	0	0	0	1	1	no
XLOC_006938	g9299	0	0	0	0	1	1	no
XLOC_006939	g3352	293,999	91,4695	-1,68445	-2,58294	5,00E-05	0,00120049	yes
XLOC_006940	g3353	87,8368	83,0091	-0,0815568	-0,119106	0,8369	0,925387	no
XLOC_006941	g3351	40,1584	31,4971	-0,350484	-0,523995	0,34775	0,608067	no
XLOC_006942	g9300	0	0	0	0	1	1	no
XLOC_006943	g3354	40,2096	50,4767	0,328077	0,537108	0,33785	0,597781	no
XLOC_006944	g3356	23,8963	24,5154	0,0369037	0,05653	0,9186	0,964054	no
XLOC_006945	g3358	58,6391	29,1737	-1,00719	-1,4446	0,0114	0,0729303	no
XLOC_006946	g3362	34,5355	64,3586	0,898056	1,31049	0,02275	0,11934	no
XLOC_006947	g3364	19,3846	39,4628	1,02558	1,46218	0,0101	0,0670149	no
XLOC_006948	g3366	194,78	314,015	0,688991	1,16624	0,04375	0,179985	no
XLOC_006949	g3371	569,932	505,897	-0,171944	-0,292713	0,5934	0,79163	no
XLOC_006950	g3372	232,841	90,9313	-1,35649	-1,81884	0,00265	0,0257909	yes
XLOC_006951	g3373	13,8131	9,00157	-0,617786	-0,925677	0,1028	0,308903	no
XLOC_006952	g3374	34,903	41,985	0,266521	0,443607	0,4291	0,677533	no
XLOC_006953	g3375	19,1098	18,4878	-0,04774	-0,0690937	0,90365	0,957834	no
XLOC_006954	g3378	30,6361	30,4894	-0,00692637	-0,0108624	0,98435	0,992379	no
XLOC_006955	g3379	50,866	25,112	-1,01832	-1,67746	0,00305	0,028799	yes
XLOC_006956	g3381	240,247	384,311	0,677754	0,831801	0,12655	0,351051	no
XLOC_006957	g3382	37,4943	173,167	2,20742	3,23332	5,00E-05	0,00120049	yes
XLOC_006958	g3385	56,707	96,8142	0,771692	1,14646	0,0411	0,172775	no
XLOC_006959	g3387	205,338	552,302	1,42745	2,26622	0,0001	0,00209829	yes
XLOC_006960	g3388	334,303	232,545	-0,523644	-0,805164	0,17175	0,416749	no
XLOC_006961	g3389	76,0098	45,5816	-0,737734	-1,19388	0,0366	0,16038	no
XLOC_006962	g3355	73,8986	156,722	1,08459	1,68151	0,0046	0,0386416	yes
XLOC_006963	g3357	21,3467	18,1096	-0,237258	-0,38473	0,50135	0,730345	no
XLOC_006964	g3359	28,5165	15,3957	-0,88927	-1,37837	0,01245	0,077872	no
XLOC_006965	g3360	1,01489	0,53254	-0,930359	0	1	1	no
XLOC_006966	g3361	23,4743	26,8827	0,195592	0,332999	0,54745	0,76377	no
XLOC_006967	g3363	24,7065	46,0579	0,898558	1,534	0,0074	0,0541087	no
XLOC_006968	g3365	28,6014	40,5267	0,502788	0,656423	0,25185	0,510774	no
XLOC_006969	g3367	1236,93	1807,54	0,547268	0,896826	0,12325	0,346994	no
XLOC_006970	g3368	10,4745	8,13021	-0,365518	-0,512254	0,37745	0,635531	no
XLOC_006971	g3369	27,8764	38,3127	0,458776	0,757187	0,18065	0,427981	no
XLOC_006972	g3370	55,0281	62,6194	0,186441	0,318108	0,56475	0,774343	no
XLOC_006973	g3376	52,8307	52,7882	-0,00116067	-0,00196748	0,99745	0,998008	no
XLOC_006974	g3377	68,6419	160,939	1,22936	1,99518	0,00095	0,0120686	yes
XLOC_006975	g3380	22,8031	27,2305	0,255992	0,444152	0,4414	0,687914	no
XLOC_006976	g3383	73,5434	139,317	0,921702	1,57954	0,00535	0,0426783	yes
XLOC_006977	g3384	37,8652	37,1438	-0,027754	-0,0388825	0,94485	0,975496	no
XLOC_006978	g3386	26,0735	24,3436	-0,099037	-0,151645	0,78545	0,901276	no
XLOC_006979	g3390	57,5797	51,2608	-0,167702	-0,286897	0,60885	0,802538	no
XLOC_006980	g3391	19,7439	25,1561	0,349501	0,466184	0,41885	0,670105	no
XLOC_006981	g9301	0	0	0	0	1	1	no
XLOC_006982	g9303	178,724	123,356	-0,534908	-0,905347	0,1171	0,335495	no
XLOC_006983	g9305	71,7168	85,872	0,259876	0,37816	0,50805	0,734873	no
XLOC_006984	g9302	24,9396	11,5124	-1,11525	-1,6949	0,0039	0,0342252	yes
XLOC_006985	g9304	24,2265	29,1939	0,269081	0,460814	0,42015	0,671172	no
XLOC_006986	g9306	7,19995	26,8681	1,89984	1,86772	0,00535	0,0426783	yes
XLOC_006987	g9307	9,8457	17,8639	0,859482	1,18008	0,0376	0,163594	no
XLOC_006988	g9308	0	0	0	0	1	1	no
XLOC_006989	g9309	2,60689	1,88749	-0,465857	-0,422887	0,4571	0,701012	no
XLOC_006990	g9310	0	0	0	0	1	1	no
XLOC_006991	g9311	38,95	26,918	-0,533054	-0,701361	0,22905	0,487277	no

XLOC_006992	g9312	47,0013	41,0451	-0,195492	-0,324552	0,56565	0,774849	no
XLOC_006993	g3392	0	0	0	0	1	1	no
XLOC_006994	g9313	0	0	0	0	1	1	no
XLOC_006995	g9314	19,0396	10,7887	-0,819487	-1,04861	0,06815	0,238841	no
XLOC_006996	g9315	28,3355	31,6793	0,160928	0,236434	0,68315	0,847572	no
XLOC_006997	g9316	0	0	0	0	1	1	no
XLOC_006998	g3394	6,33505	1,85818	-1,76947	-2,06769	0,00095	0,0120686	yes
XLOC_006999	g3397	391,709	332,156	-0,237924	-0,320684	0,5499	0,765215	no
XLOC_007000	g3393	32,3526	36,2885	0,16563	0,254294	0,6439	0,824286	no
XLOC_007001	g3395	225,632	274,323	0,281903	0,412424	0,4615	0,702668	no
XLOC_007002	g3396	194,766	83,8184	-1,2164	-1,4155	0,0129	0,0801252	no
XLOC_007003	g3398	4,45015	3,14175	-0,502287	-0,600219	0,2922	0,554783	no
XLOC_007004	g9317	36,7833	22,8067	-0,689592	-1,10922	0,0438	0,18004	no
XLOC_007005	g9318	23,4583	32,9394	0,489715	0,737967	0,1901	0,440071	no
XLOC_007006	g9319	92,0485	121,488	0,400344	0,664041	0,2444	0,503031	no
XLOC_007007	g9320	27,6714	32,761	0,243579	0,395363	0,4764	0,71293	no
XLOC_007008	g9321	16460,2	26556,7	0,690096	0,905552	0,10585	0,315366	no
XLOC_007009	g9322	39,5578	19,3183	-1,03399	-1,7058	0,00205	0,0212577	yes
XLOC_007010	g9323	21,1945	19,9739	-0,0855762	-0,115187	0,83865	0,926383	no
XLOC_007011	g3399	62,027	57,9705	-0,0975762	-0,164145	0,77285	0,894131	no
XLOC_007012	g3400	214,804	97,9881	-1,13234	-1,48467	0,0112	0,0720262	no
XLOC_007013	g9324	0	0	0	0	1	1	no
XLOC_007014	g9325	0	0	0	0	1	1	no
XLOC_007015	g9326	18,8996	14,9081	-0,342266	-0,392108	0,4911	0,723137	no
XLOC_007016	g9327	0	0	0	0	1	1	no
XLOC_007017	g9328	0	0	0	0	1	1	no
XLOC_007018	g9329	153,681	114,331	-0,426712	-0,669514	0,24155	0,499688	no
XLOC_007019	g3402	34,0354	43,206	0,344194	0,568198	0,32655	0,587312	no
XLOC_007020	g3403	75,6362	72,6846	-0,0574279	-0,0947597	0,8729	0,942536	no
XLOC_007021	g3407	3,17305	2,0933	-0,600091	-0,646979	0,26325	0,523621	no
XLOC_007022	g3408	5,16264	8,96831	0,796727	1,09122	0,0561	0,211804	no
XLOC_007023	g3410	42,1458	46,5876	0,144557	0,24092	0,6676	0,83795	no
XLOC_007024	g3412	53,3931	113,825	1,09209	1,75817	0,0028	0,0269305	yes
XLOC_007025	g3414	17,3538	34,0161	0,970969	1,39105	0,014	0,0846552	no
XLOC_007026	g3401	30,4224	19,2678	-0,658939	-0,998985	0,0841	0,273555	no
XLOC_007027	g3404	70,5897	99,9233	0,501363	0,752752	0,1805	0,427729	no
XLOC_007028	g3405	21,1799	17,4723	-0,277627	-0,464462	0,4097	0,662373	no
XLOC_007029	g3406	153,207	609,953	1,99322	3,20501	5,00E-05	0,00120049	yes
XLOC_007030	g3409	2,74146	2,93239	0,0971308	0,104571	0,89565	0,953625	no
XLOC_007031	g3411	55,9442	45,4105	-0,300962	-0,448665	0,42845	0,677386	no
XLOC_007032	g3413	296,545	249,552	-0,248907	-0,377091	0,50885	0,735164	no
XLOC_007033	g3415	73,4571	110,409	0,587878	0,994137	0,08715	0,278857	no
XLOC_007034	g3416	48,3953	37,0389	-0,385828	-0,555943	0,32785	0,589002	no
XLOC_007035	g9330	0	0	0	0	1	1	no
XLOC_007036	g3417	450,499	103,387	-2,12347	-2,83486	5,00E-05	0,00120049	yes
XLOC_007037	g9331	0	0	0	0	1	1	no
XLOC_007038	g9332	0	0	0	0	1	1	no
XLOC_007039	g9333	2,57094	2,81554	0,131112	0,130804	0,83535	0,924776	no
XLOC_007040	g9334	39,9483	40,055	0,00384712	0,0051073	0,96425	0,984142	no
XLOC_007041	g9335	28,6647	39,1206	0,448652	0,683715	0,2162	0,472111	no
XLOC_007042	g9336	42,6106	41,0087	-0,0552836	-0,0950534	0,86775	0,939455	no
XLOC_007043	g9338	54,2712	41,0365	-0,403277	-0,676125	0,2278	0,485987	no
XLOC_007044	g9340	92,8869	90,8737	-0,0316122	-0,0510071	0,92885	0,968394	no
XLOC_007045	g9343	291,617	493,857	0,760017	1,07267	0,05655	0,213012	no
XLOC_007046	g9337	144,419	139,251	-0,0525724	-0,0906803	0,8735	0,942923	no
XLOC_007047	g9339	30,2891	22,4419	-0,4326	-0,6561	0,2612	0,521008	no
XLOC_007048	g9341	27,8481	35,1776	0,337077	0,534831	0,33985	0,600023	no
XLOC_007049	g9342	500,149	429,153	-0,220867	-0,346626	0,53385	0,753431	no
XLOC_007050	g9344	60,2476	50,5106	-0,254317	-0,403144	0,4697	0,707649	no
XLOC_007051	g3419	27,8896	20,7718	-0,425103	-0,719875	0,1991	0,451802	no
XLOC_007052	g3423	29,0313	23,8098	-0,286055	-0,403841	0,4693	0,707556	no
XLOC_007053	g3418	26,9926	38,3878	0,508082	0,828695	0,1385	0,371198	no
XLOC_007054	g3420	74,9896	62,769	-0,256637	-0,430103	0,4507	0,695455	no
XLOC_007055	g3421	620,263	241,839	-1,35884	-1,5116	0,0066	0,0498938	yes
XLOC_007056	g3422	2,75576	0,91516	-1,59035	-1,36109	0,01945	0,106823	no
XLOC_007057	g9345	16,6078	28,233	0,765518	0,756715	0,1853	0,433572	no
XLOC_007058	g9346	81,5995	36,4334	-1,1633	-1,47684	0,00905	0,0621476	no
XLOC_007059	g9347	55,2215	40,4054	-0,450683	-0,64557	0,2785	0,540061	no
XLOC_007060	g3424	50,0237	40,788	-0,294469	-0,481183	0,40765	0,660523	no
XLOC_007061	g3426	183,233	112,933	-0,698219	-1,14505	0,0453	0,184125	no
XLOC_007062	g3425	264,423	1003,16	1,92364	1,70694	0,0034	0,0310297	yes
XLOC_007063	g9348	12,4488	23,3232	0,905753	1,11136	0,05865	0,216928	no
XLOC_007064	g9350	134,666	196,153	0,542599	0,80336	0,15475	0,392851	no
XLOC_007065	g9349	84,4844	69,389	-0,283979	-0,488884	0,38755	0,643188	no
XLOC_007066	g3427	6,64248	10,7776	0,698243	0,91953	0,104	0,311841	no
XLOC_007067	g3429	57,2342	40,9928	-0,481507	-0,717996	0,209	0,464024	no
XLOC_007068	g3430	843,27	391,394	-1,10737	-1,50118	0,0056	0,0440994	yes
XLOC_007069	g3432	3719,14	1742,28	-1,09399	-1,64505	0,00535	0,0426783	yes
XLOC_007070	g3428	317,777	1117,97	1,8148	2,82065	5,00E-05	0,00120049	yes
XLOC_007071	g3431	14,7112	295,234	4,32687	5,45656	5,00E-05	0,00120049	yes
XLOC_007072	g3433	2,50338	3,23831	0,371362	0,423664	0,45325	0,698162	no
XLOC_007073	g3435	5,07679	8,18271	0,688662	0,899866	0,11055	0,324157	no
XLOC_007074	g3438	5,89892	4,79239	-0,299705	-0,341186	0,5852	0,786245	no
XLOC_007075	g3434	949,029	610,491	-0,636482	-0,836995	0,13925	0,372294	no
XLOC_007076	g3436	16,4153	19,3205	0,235099	0,373492	0,5083	0,734874	no
XLOC_007077	g3437	120,048	107,135	-0,164186	-0,254215	0,6577	0,832081	no
XLOC_007078	g3439	134,614	141,822	0,0752469	0,10772	0,8455	0,929972	no
XLOC_007079	g3440	35,2527	14,1259	-1,31939	-2,22918	0,0001	0,00209829	yes
XLOC_007080	g3443	25,4282	35,6114	0,485911	0,757144	0,18175	0,429344	no
XLOC_007081	g3441	45,3864	60,261	0,408963	0,726444	0,23315	0,49079	no
XLOC_007082	g3442	42,0637	64,3825	0,614094	1,04911	0,0603	0,22095	no
XLOC_007083	g9351	0	0	0	0	1	1	no

XLOC_007084	g9352	241,834	190,133	-0,347004	-0,529884	0,33935	0,599571	no
XLOC_007085	g9353	0	0	0	0	1	1	no
XLOC_007086	g9354	0,459979	1,23447	1,42425	0,927494	0,3094	0,570452	no
XLOC_007087	g3445	31,804	242,616	2,93139	3,1059	0,0001	0,00209829	yes
XLOC_007088	g3444	2,23832	24,0134	3,42335	4,45841	5,00E-05	0,00120049	yes
XLOC_007089	g474	44,8265	44,4924	-0,0107951	-0,0165793	0,9749	0,987724	no
XLOC_007090	g476	5,84096	7,31346	0,324346	0,436228	0,45495	0,699375	no
XLOC_007091	g479	84,3749	114,481	0,440228	0,759403	0,18565	0,433654	no
XLOC_007092	g481	22,4428	42,2949	0,914232	1,55637	0,0073	0,053577	no
XLOC_007093	g484	159,51	52,6017	-1,60046	-2,53961	5,00E-05	0,00120049	yes
XLOC_007094	g475	73,5274	54,4727	-0,43275	-0,735322	0,18885	0,438314	no
XLOC_007095	g477	6107,89	3496,26	-0,80486	-1,01802	0,0883	0,281254	no
XLOC_007096	g478	90,2145	91,8103	0,0252953	0,0346776	0,9519	0,978762	no
XLOC_007097	g480	28,7398	35,5212	0,305631	0,50916	0,3611	0,621451	no
XLOC_007098	g482	126,678	168,853	0,414602	0,723638	0,213	0,468352	no
XLOC_007099	g483	32,4466	70,3193	1,11585	1,78265	0,00175	0,0188639	yes
XLOC_007100	g9355	0	0	0	0	1	1	no
XLOC_007101	g3446	10,3919	7,1057	-0,548416	-0,700858	0,2231	0,480342	no
XLOC_007102	g9356	165,754	152,656	-0,118765	-0,200747	0,72315	0,86956	no
XLOC_007103	g9358	34,8771	39,3215	0,173038	0,292558	0,5994	0,795859	no
XLOC_007104	g9357	168,801	248,496	0,557897	0,956634	0,0925	0,28993	no
XLOC_007105	g3448	4,4283	3,23622	-0,452445	-0,568731	0,3343	0,594729	no
XLOC_007106	g3447	180,945	94,3875	-0,938884	-1,2981	0,02155	0,115074	no
XLOC_007107	g3449	41,9101	41,918	0,000273439	0,000454908	0,99905	0,999152	no
XLOC_007108	g3450	28,7504	26,1465	-0,136966	-0,236024	0,67635	0,843308	no
XLOC_007109	g3451	14,0353	4,88264	-1,52332	-2,4195	5,00E-05	0,00120049	yes
XLOC_007110	g9359	0,298818	0,934128	1,64435	0	1	1	no
XLOC_007111	g9360	2835,4	1616,32	-0,81084	-0,968806	0,0858	0,276611	no
XLOC_007112	g9361	47,6392	27,3488	-0,80067	-1,02236	0,0848	0,27483	no
XLOC_007113	g3452	43,9627	89,4317	1,02451	1,53813	0,00725	0,0533296	no
XLOC_007114	g9362	0	0	0	0	1	1	no
XLOC_007115	g486	47,108	77,1057	0,710866	1,13509	0,04325	0,178677	no
XLOC_007116	g488	288,95	309,904	0,101	0,17449	0,7585	0,885563	no
XLOC_007117	g490	37,8357	41,1891	0,122516	0,195528	0,72585	0,869807	no
XLOC_007118	g492	335,815	444,93	0,40591	0,566075	0,2949	0,556907	no
XLOC_007119	g485	50,4827	56,5482	0,163692	0,261286	0,65185	0,829167	no
XLOC_007120	g487	126,181	273,236	1,11466	1,79386	0,0033	0,0304282	yes
XLOC_007121	g489	9,12093	20,9887	1,20236	1,89395	0,0011	0,0134856	yes
XLOC_007122	g491	57,4958	43,866	-0,390354	-0,641709	0,253	0,512145	no
XLOC_007123	g493	60,463	40,7481	-0,569322	-0,896277	0,11705	0,335495	no
XLOC_007124	g9363	41,9576	41,7104	-0,00852588	-0,0145834	0,97815	0,989843	no
XLOC_007125	g9364	2,72847	2,65932	-0,0370385	-0,0324026	0,95905	0,981744	no
XLOC_007126	g9365	0	0,32072	inf	0	1	1	no
XLOC_007127	g9366	10,784	6,96054	-0,631615	-0,710738	0,22005	0,477334	no
XLOC_007128	g9367	0	0	0	0	1	1	no
XLOC_007129	g9368	3,84984	1,62657	-1,24296	-1,06474	0,083	0,271381	no
XLOC_007130	g9369	0	0	0	0	1	1	no
XLOC_007131	g9370	0	0	0	0	1	1	no
XLOC_007132	g9371	0	0	0	0	1	1	no
XLOC_007133	g9372	1711,17	219,482	-2,96281	-4,09953	5,00E-05	0,00120049	yes
XLOC_007134	g9373	56,6576	55,312	-0,034675	-0,0569458	0,92105	0,965078	no
XLOC_007135	g9374	7,16891	9,28487	0,373127	0,578637	0,2979	0,559975	no
XLOC_007136	g9375	22,5366	20,2047	-0,157581	-0,204345	0,72605	0,869807	no
XLOC_007137	g9376	0	0	0	0	1	1	no
XLOC_007138	g9377	37,0458	75,5349	1,02783	1,6301	0,00645	0,0491	yes
XLOC_007139	g9379	2167,14	3955,99	0,86825	1,43168	0,014	0,0846552	no
XLOC_007140	g9378	595,38	1618,21	1,44251	2,24389	0,0001	0,00209829	yes
XLOC_007141	g9380	0	0	0	0	1	1	no
XLOC_007142	g9381	0	0	0	0	1	1	no
XLOC_007143	g9382	0	0	0	0	1	1	no
XLOC_007144	g3454	349,135	232,408	-0,587127	-0,849933	0,13395	0,363669	no
XLOC_007145	g3453	3,88307	3,99637	0,0414903	0,05242	0,92455	0,966785	no
XLOC_007146	g9383	1,53032	1,24707	-0,295293	-0,297933	0,6061	0,80031	no
XLOC_007147	g3455	0	0	0	0	1	1	no
XLOC_007148	g3457	7,88594	4,61414	-0,773219	-1,11493	0,04625	0,186443	no
XLOC_007149	g3459	63,1261	93,317	0,563903	0,928259	0,0943	0,292953	no
XLOC_007150	g3460	32,2968	51,964	0,686122	1,07975	0,05375	0,206101	no
XLOC_007151	g3461	145,551	231,803	0,671378	1,12065	0,0531	0,204648	no
XLOC_007152	g3462	24,3327	23,3835	-0,0574027	-0,0835491	0,8799	0,94588	no
XLOC_007153	g3464	33,0639	25,0714	-0,399217	-0,644266	0,2612	0,521008	no
XLOC_007154	g3465	0,910092	12,7161	3,8045	2,59381	0,0567	0,213413	no
XLOC_007155	g3467	72,0321	136,449	0,921652	1,52004	0,00945	0,0639552	no
XLOC_007156	g3469	56,9177	28,782	-0,983709	-1,59738	0,00805	0,0571591	no
XLOC_007157	g3471	155,671	143,822	-0,114222	-0,190831	0,74	0,876968	no
XLOC_007158	g3473	123,964	116,596	-0,0883957	-0,152445	0,78945	0,902806	no
XLOC_007159	g3476	140,451	132,337	-0,0858576	-0,139615	0,8074	0,911027	no
XLOC_007160	g3477	495,929	410,39	-0,273138	-0,45183	0,43	0,678111	no
XLOC_007161	g3479	77,6291	48,7674	-0,67068	-1,13035	0,04925	0,1947	no
XLOC_007162	g3481	452,226	554,631	0,294483	0,463726	0,4077	0,660523	no
XLOC_007163	g3482	94,9359	58,7081	-0,693395	-1,15792	0,042	0,175432	no
XLOC_007164	g3456	494,59	362,295	-0,449066	-0,721872	0,20985	0,464756	no
XLOC_007165	g3458	154,475	136,991	-0,173285	-0,296666	0,60695	0,800918	no
XLOC_007166	g3463	144,684	161,829	0,161571	0,278436	0,6252	0,812208	no
XLOC_007167	g3466	37,3126	89,9879	1,27007	2,02666	0,00045	0,00669545	yes
XLOC_007168	g3468	9,14715	11,0008	0,266213	0,304624	0,5996	0,795859	no
XLOC_007169	g3470	14,6178	21,4338	0,552162	0,854982	0,15235	0,390111	no
XLOC_007170	g3472	9,20825	6,51208	-0,499809	-0,505963	0,4482	0,694183	no
XLOC_007171	g3474	15,7861	27,6298	0,807568	1,25207	0,0285	0,137326	no
XLOC_007172	g3475	4,79169	18,6762	1,96259	2,31243	0,0002	0,00367103	yes
XLOC_007173	g3478	18,4145	24,2277	0,395816	0,674756	0,2333	0,49079	no
XLOC_007174	g3480	32,0612	34,6244	0,11096	0,18313	0,7493	0,881846	no
XLOC_007175	g3483	110,159	132,192	0,263052	0,438959	0,42855	0,677386	no

XLOC_007176	g3484	28,0175	34,8976	0,316798	0,438375	0,44255	0,688832	no
XLOC_007177	g3485	2,35974	6,09076	1,36799	1,54	0,1514	0,388896	no
XLOC_007178	g9384	0	1,40308	inf	0	1	1	no
XLOC_007179	g3486	49,6439	52,0533	0,0683739	0,109395	0,84195	0,927787	no
XLOC_007180	g9385	0,0186128	0,016665	-0,159472	0	1	1	no
XLOC_007181	g9386	17,1281	18,2018	0,0877128	0,101564	0,85805	0,935373	no
XLOC_007182	g3487	24,6544	12,231	-1,0113	-1,34019	0,0198	0,10814	no
XLOC_007183	g3488	66,4183	83,5749	0,33149	0,534207	0,35025	0,610916	no
XLOC_007184	g496	37,7674	40,6712	0,106867	0,183087	0,7485	0,881846	no
XLOC_007185	g498	102,072	130,837	0,358172	0,546118	0,34125	0,601413	no
XLOC_007186	g494	146,392	64,9103	-1,17332	-1,77646	0,0026	0,0255065	yes
XLOC_007187	g495	7,01634	8,22906	0,230009	0,323189	0,5595	0,77102	no
XLOC_007188	g497	7,24953	12,1145	0,740773	1,01999	0,08095	0,267113	no
XLOC_007189	g9387	10,3094	10,819	0,0696094	0,106622	0,8495	0,932181	no
XLOC_007190	g9388	41,8404	32,1189	-0,381476	-0,621732	0,29165	0,554382	no
XLOC_007191	g3489	16,1639	11,4358	-0,49922	-0,702573	0,232	0,489839	no
XLOC_007192	g3490	13,9779	19,6215	0,489286	0,619955	0,28355	0,546283	no
XLOC_007193	g3491	0,0717337	0	#NAME?	0	1	1	no
XLOC_007194	g9389	40,8828	76,2825	0,899859	1,48096	0,0077	0,0555169	no
XLOC_007195	g9390	2159,94	2638,86	0,288927	0,365497	0,52795	0,74938	no
XLOC_007196	g9391	53,1601	30,0211	-0,824367	-1,40892	0,01335	0,0818844	no
XLOC_007197	g9392	1276,48	349,623	-1,8683	-2,39924	0,00015	0,00294012	yes
XLOC_007198	g3492	7,03296	7,71516	0,133564	0,172377	0,76305	0,888504	no
XLOC_007199	g3493	7,29794	11,8765	0,702544	0,77791	0,19065	0,440825	no
XLOC_007200	g9393	0	0	0	0	1	1	no
XLOC_007201	g9394	21,8163	20,493	-0,0902722	-0,125754	0,8264	0,920883	no
XLOC_007202	g3494	6,12745	8,86036	0,53208	0,552795	0,34645	0,606638	no
XLOC_007203	g3495	89,0591	73,5179	-0,276668	-0,4148	0,4672	0,707046	no
XLOC_007204	g9395	30,9768	41,7546	0,430748	0,663146	0,251	0,509889	no
XLOC_007205	g9396	11,1336	8,30699	-0,422524	-0,616839	0,28035	0,542577	no
XLOC_007206	g9398	35,3276	33,5017	-0,0765609	-0,131125	0,81855	0,917235	no
XLOC_007207	g9397	79,4176	109,33	0,461163	0,789716	0,1628	0,404836	no
XLOC_007208	g9399	25,324	31,9434	0,335013	0,47756	0,4106	0,663116	no
XLOC_007209	g3496	0	0	0	0	1	1	no
XLOC_007210	g9400	2,71161	2,94221	0,11775	0,0969783	0,857	0,935212	no
XLOC_007211	g9401	6,29596	0	#NAME?	#NAME?	0,0201	0,109292	no
XLOC_007212	g9402	0	0	0	0	1	1	no
XLOC_007213	g3497	7,73833	13,3318	0,784772	1,10635	0,05775	0,215057	no
XLOC_007214	g9403	0	0	0	0	1	1	no
XLOC_007215	g9404	0	0	0	0	1	1	no
XLOC_007216	g9405	127,015	53,4813	-1,2479	-2,01063	0,00065	0,00901554	yes
XLOC_007217	g9406	0	0	0	0	1	1	no
XLOC_007218	g9407	0	1,38651	inf	0	1	1	no
XLOC_007219	g9408	14,105	21,2643	0,592233	0,905896	0,1032	0,309726	no
XLOC_007220	g9409	245,387	137,951	-0,830906	-0,906494	0,3382	0,598274	no
XLOC_007221	g3498	0	0	0	0	1	1	no
XLOC_007222	g3500	0	0	0	0	1	1	no
XLOC_007223	g3499	0	0	0	0	1	1	no
XLOC_007224	g9410	6,39441	9,64403	0,592824	0,804914	0,16985	0,413672	no
XLOC_007225	g9411	7,70284	15,0479	0,966098	0,916374	0,11185	0,3267	no
XLOC_007226	g9413	227,553	299,094	0,394396	0,655489	0,25845	0,51859	no
XLOC_007227	g9414	33,3878	35,4238	0,0853972	0,140236	0,80605	0,91076	no
XLOC_007228	g9412	31,8138	42,2717	0,410043	0,58797	0,2926	0,555233	no
XLOC_007229	g9415	35,7558	60,8516	0,767117	0,828016	0,14935	0,38697	no
XLOC_007230	g9419	20,2901	27,3628	0,431441	0,630512	0,2774	0,538992	no
XLOC_007231	g9416	94,5104	56,6237	-0,739066	-1,21282	0,0281	0,135999	no
XLOC_007232	g9417	83,9859	45,3752	-0,888244	-1,39593	0,01495	0,0883859	no
XLOC_007233	g9418	154,01	188,048	0,288074	0,474163	0,4063	0,659809	no
XLOC_007234	g3501	51,6194	15,0311	-1,77996	-2,58769	0,0001	0,00209829	yes
XLOC_007235	g9420	140,893	152,688	0,115985	0,184795	0,74035	0,876968	no
XLOC_007236	g9421	0	0	0	0	1	1	no
XLOC_007237	g9422	0	0	0	0	1	1	no
XLOC_007238	g9423	9,80413	17,3057	0,819787	0,905789	0,13395	0,363669	no
XLOC_007239	g9424	172,993	105,368	-0,715275	-1,20649	0,0358	0,158216	no
XLOC_007240	g9425	350,741	327,013	-0,10106	-0,161913	0,77455	0,894738	no
XLOC_007241	g9427	11,1575	12,6351	0,179423	0,17948	0,7519	0,883213	no
XLOC_007242	g9426	46,3216	56,2007	0,278904	0,450964	0,4275	0,676996	no
XLOC_007243	g9428	106,063	90,07	-0,235801	-0,286332	0,63165	0,816117	no
XLOC_007244	g9429	24,9705	32,919	0,398696	0,382057	0,4908	0,722912	no
XLOC_007245	g9430	3,62377	2,70097	-0,424015	-0,520132	0,3708	0,629539	no
XLOC_007246	g9431	35,2836	24,7488	-0,511637	-0,859064	0,1381	0,370429	no
XLOC_007247	g3502	1,68884	1,30186	-0,375454	-0,327694	0,62885	0,814255	no
XLOC_007248	g3503	44,8903	50,6002	0,172739	0,232496	0,69705	0,856211	no
XLOC_007249	g9432	0	0	0	0	1	1	no
XLOC_007250	g9433	0	0	0	0	1	1	no
XLOC_007251	g9434	39,5739	25,6545	-0,62534	-0,837042	0,1478	0,384374	no
XLOC_007252	g9435	0	0	0	0	1	1	no
XLOC_007253	g9436	0	0	0	0	1	1	no
XLOC_007254	g9437	0	0	0	0	1	1	no
XLOC_007255	g9438	8,58011	22,7708	1,40812	1,80392	0,00125	0,0146655	yes
XLOC_007256	g9441	26,4142	21,3316	-0,308319	-0,30531	0,59125	0,790276	no
XLOC_007257	g9439	12,496	7,87061	-0,666917	-0,97677	0,09205	0,289073	no
XLOC_007258	g9440	26,1391	26,3088	0,00934082	0,0146832	0,97885	0,990041	no
XLOC_007259	g3504	16,039	5,43219	-1,56198	-1,66012	0,1149	0,332445	no
XLOC_007260	g3506	6,22917	4,64807	-0,422408	-0,633577	0,27015	0,531531	no
XLOC_007261	g3505	72,0849	48,3545	-0,576048	-0,947282	0,09425	0,292953	no
XLOC_007262	g3507	43,2748	22,8522	-0,921195	-1,4796	0,0111	0,0715706	no
XLOC_007263	g9442	108,067	88,2165	-0,292807	-0,42458	0,4392	0,685356	no
XLOC_007264	g9443	84,1477	71,6229	-0,232503	-0,400352	0,48345	0,717575	no
XLOC_007265	g9444	153,365	160,038	0,0614392	0,100387	0,8573	0,935212	no
XLOC_007266	g3508	3,80389	2,7756	-0,454676	-0,629867	0,25545	0,514986	no
XLOC_007267	g3509	33,4091	40,1884	0,266539	0,449445	0,4285	0,677386	no

XLOC_007268	g3511	32,9284	38,8496	0,238568	0,393716	0,4838	0,717735	no
XLOC_007269	g3514	33,7074	41,2879	0,292653	0,428503	0,4445	0,690882	no
XLOC_007270	g3515	20,3937	66,563	1,7066	2,21106	0,0002	0,00367103	yes
XLOC_007271	g3516	106,999	166,377	0,636858	1,0446	0,0656	0,232476	no
XLOC_007272	g3510	455,435	350,866	-0,376324	-0,485818	0,38355	0,640119	no
XLOC_007273	g3512	60,9041	121,054	0,991041	1,63398	0,004	0,0348845	yes
XLOC_007274	g3513	35,1995	18,1017	-0,959433	-1,4869	0,0084	0,0588359	no
XLOC_007275	g9445	0	0	0	0	1	1	no
XLOC_007276	g3518	50,9272	37,6624	-0,435311	-0,682834	0,22945	0,487604	no
XLOC_007277	g3519	109,573	221,134	1,01302	1,63068	0,00585	0,0457017	yes
XLOC_007278	g3521	28,1184	26,2172	-0,101	-0,17361	0,75675	0,885098	no
XLOC_007279	g3517	74,0985	103,336	0,479833	0,782127	0,17675	0,423751	no
XLOC_007280	g3520	8,18471	12,6916	0,632871	0,805281	0,1662	0,409249	no
XLOC_007281	g3522	5,24586	9,42219	0,844883	0,991219	0,0858	0,276611	no
XLOC_007282	g9446	11,2915	10,7291	-0,0737051	-0,093857	0,86575	0,938581	no
XLOC_007283	g9447	0	0	0	0	1	1	no
XLOC_007284	g9448	134,85	136,19	0,0142711	0,0174321	0,97535	0,987925	no
XLOC_007285	g9449	11,7687	4,72401	-1,31687	-1,77387	0,00265	0,0257909	yes
XLOC_007286	g9450	141,395	72,3261	-0,967139	-1,6354	0,0046	0,0386416	yes
XLOC_007287	g9451	11,4093	18,1717	0,67148	0,696977	0,227	0,48544	no
XLOC_007288	g9452	2248,66	1096,2	-1,03656	-1,43889	0,0109	0,0706055	no
XLOC_007289	g9453	0	0	0	0	1	1	no
XLOC_007290	g9454	0	0,104917	inf	0	1	1	no
XLOC_007291	g9455	71,8327	75,5773	0,0733123	0,125636	0,82635	0,920883	no
XLOC_007292	g9456	0	0	0	0	1	1	no
XLOC_007293	g3524	32,7618	25,8127	-0,343933	-0,572648	0,3223	0,582656	no
XLOC_007294	g3526	805,481	1985,1	1,30129	2,18701	0,00035	0,00552572	yes
XLOC_007295	g3523	44,6263	36,173	-0,302981	-0,433065	0,44525	0,6915	no
XLOC_007296	g3525	18,1022	13,2427	-0,450966	-0,644375	0,26225	0,522372	no
XLOC_007297	g3527	36,4012	37,4254	0,0400316	0,0690495	0,9037	0,957834	no
XLOC_007298	g3528	1,31029	1,9116	0,544893	0,623382	0,2899	0,552768	no
XLOC_007299	g9457	113,818	79,4555	-0,518507	-0,6542	0,2609	0,521008	no
XLOC_007300	g3530	0	0	0	0	1	1	no
XLOC_007301	g3529	0	0	0	0	1	1	no
XLOC_007302	g9458	26,1418	11,6594	-1,16486	-1,40479	0,01315	0,0811137	no
XLOC_007303	g9459	25,8151	21,0404	-0,295055	-0,43211	0,45695	0,701012	no
XLOC_007304	g9460	0,773676	3,33298	2,10701	1,42794	0,03525	0,1572	no
XLOC_007305	g9461	0	0	0	0	1	1	no
XLOC_007306	g9462	22,2645	22,4266	0,0104615	0,0152659	0,97945	0,990233	no
XLOC_007307	g9463	26,8238	25,7748	-0,0575497	-0,0980466	0,8603	0,935634	no
XLOC_007308	g9465	58,8379	47,4337	-0,310832	-0,506387	0,37055	0,629539	no
XLOC_007309	g9467	5,75595	11,1613	0,955377	1,33516	0,02605	0,129919	no
XLOC_007310	g9464	97,1711	123,908	0,350673	0,556444	0,3286	0,5899	no
XLOC_007311	g9466	58,8283	64,103	0,12388	0,214633	0,7044	0,859387	no
XLOC_007312	g9468	27,1883	35,6125	0,389396	0,629185	0,2493	0,508092	no
XLOC_007313	g9469	26,1367	32,0457	0,29405	0,405222	0,47415	0,711515	no
XLOC_007314	g9471	11,3644	15,7219	0,468253	0,586345	0,3102	0,571193	no
XLOC_007315	g9473	2454,1	3898,59	0,667756	0,698998	0,18435	0,432469	no
XLOC_007316	g9475	2,44729	5,64567	1,20596	1,01018	0,09545	0,295373	no
XLOC_007317	g9470	1,43904	3,7151	1,36829	1,15744	0,06465	0,230608	no
XLOC_007318	g9472	51,7156	44,4108	-0,21969	-0,299012	0,60195	0,798265	no
XLOC_007319	g9474	64,9907	27,2183	-1,25566	-1,85209	0,00125	0,0146655	yes
XLOC_007320	g9476	13,9918	19,8107	0,501702	0,761507	0,19465	0,445978	no
XLOC_007321	g9477	45,413	29,7595	-0,609754	-1,02932	0,0749	0,252755	no
XLOC_007322	g9478	0	0	0	0	1	1	no
XLOC_007323	g9479	0	0	0	0	1	1	no
XLOC_007324	g9480	102,341	223,621	1,12768	1,47104	0,01215	0,0766793	no
XLOC_007325	g9481	69,6452	58,8745	-0,242379	-0,39975	0,47575	0,712433	no
XLOC_007326	g9482	0	0	0	0	1	1	no
XLOC_007327	g9483	10,1872	12,2461	0,265562	0,383148	0,49225	0,723714	no
XLOC_007328	g9484	17,1563	21,6123	0,333119	0,44766	0,44	0,686277	no
XLOC_007329	g9485	126,207	114,7	-0,137918	-0,227955	0,6906	0,851926	no
XLOC_007330	g3531	26,9363	29,0585	0,109411	0,152414	0,78485	0,900961	no
XLOC_007331	g9486	62,3579	82,4243	0,402497	0,591734	0,29065	0,553458	no
XLOC_007332	g3532	0	0	0	0	1	1	no
XLOC_007333	g9487	297,058	318,024	0,0983905	0,157172	0,77675	0,895794	no
XLOC_007334	g3533	0	0	0	0	1	1	no
XLOC_007335	g3534	0	0	0	0	1	1	no
XLOC_007336	g3535	60,6508	31,917	-0,926201	-1,47762	0,01125	0,0722531	no
XLOC_007337	g3537	54,8473	55,0127	0,00434213	0,00742374	0,99045	0,99501	no
XLOC_007338	g3538	278,269	419,869	0,593459	0,695854	0,20745	0,462268	no
XLOC_007339	g3540	55,5934	46,3866	-0,261205	-0,416761	0,4663	0,706537	no
XLOC_007340	g3536	3692,78	2803,95	-0,397242	-0,511716	0,3694	0,628902	no
XLOC_007341	g3539	82,4628	108,47	0,395486	0,657651	0,25585	0,515162	no
XLOC_007342	g3541	130,634	196,213	0,586886	1,00992	0,07555	0,253989	no
XLOC_007343	g3542	36,0723	78,9776	1,13055	1,86317	0,00075	0,0100752	yes
XLOC_007344	g3545	99,095	62,8288	-0,657386	-1,11762	0,04665	0,18744	no
XLOC_007345	g3543	21,8688	26,7491	0,290619	0,494339	0,3872	0,643156	no
XLOC_007346	g3544	366,838	348,112	-0,0755909	-0,126924	0,81985	0,918071	no
XLOC_007347	g3546	29,8192	45,9337	0,623314	1,04308	0,06305	0,226962	no
XLOC_007348	g9488	15,6413	22,226	0,506892	0,593877	0,2909	0,553612	no
XLOC_007349	g499	116,644	60,542	-0,946098	-1,35727	0,02065	0,111542	no
XLOC_007350	g9489	40,2539	55,9131	0,474057	0,813586	0,143	0,378099	no
XLOC_007351	g9490	28,0997	58,8337	1,06609	1,73676	0,0028	0,0269305	yes
XLOC_007352	g3548	11,4602	15,7247	0,456392	0,671261	0,2421	0,500405	no
XLOC_007353	g3549	205,489	207,533	0,0142803	0,022411	0,968	0,985921	no
XLOC_007354	g3547	0,486049	1,08279	1,15558	0	1	1	no
XLOC_007355	g9491	39,0279	40,0553	0,0374894	0,0623217	0,91205	0,962529	no
XLOC_007356	g9492	26,1704	15,1546	-0,788182	-0,998883	0,0787	0,261524	no
XLOC_007357	g9493	16,546	9,14268	-0,855794	-1,22582	0,0333	0,151392	no
XLOC_007358	g9494	225,832	1580,84	2,80737	2,86007	5,00E-05	0,00120049	yes
XLOC_007359	g9495	0	0	0	0	1	1	no

XLOC_007360	g9496	7,51075	9,67324	0,365041	0,346331	0,5396	0,758065	no
XLOC_007361	g9497	16,728	17,2452	0,0439267	0,0560192	0,92145	0,965291	no
XLOC_007362	g9498	0	0	0	0	1	1	no
XLOC_007363	g9499	0	0	0	0	1	1	no
XLOC_007364	g9500	13,0194	12,3699	-0,0738223	-0,107093	0,8508	0,933043	no
XLOC_007365	g9501	48,948	43,1303	-0,182549	-0,305083	0,59415	0,792155	no
XLOC_007366	g9503	48,8824	52,0254	0,0899016	0,143044	0,79935	0,907785	no
XLOC_007367	g9502	46,7759	87,4856	0,903281	1,43676	0,0103	0,0680202	no
XLOC_007368	g9504	0	0	0	0	1	1	no
XLOC_007369	g9505	38,451	27,1991	-0,49946	-0,753668	0,1953	0,446852	no
XLOC_007370	g9508	20,5585	11,3561	-0,856273	-1,30274	0,02445	0,124597	no
XLOC_007371	g9506	25,8602	20,399	-0,342235	-0,500306	0,39355	0,648216	no
XLOC_007372	g9507	74,2043	103,334	0,47774	0,832493	0,14835	0,385294	no
XLOC_007373	g9509	81,0979	112,351	0,470275	0,758379	0,1693	0,41264	no
XLOC_007374	g9510	0,096956	0,0466882	-1,05427	0	1	1	no
XLOC_007375	g9511	0	0	0	0	1	1	no
XLOC_007376	g3550	16,4117	16,3001	-0,00984254	-0,0120698	0,98775	0,993619	no
XLOC_007377	g3551	0	0	0	0	1	1	no
XLOC_007378	g3553	92,5449	73,6385	-0,329693	-0,532391	0,3328	0,59366	no
XLOC_007379	g3552	24,8633	23,3649	-0,0896754	-0,146868	0,79315	0,904567	no
XLOC_007380	g3554	98,0535	60,6402	-0,693296	-1,19554	0,0369	0,161479	no
XLOC_007381	g3555	22,2297	15,9558	-0,478411	-0,768212	0,1782	0,424945	no
XLOC_007382	g9512	22,9125	44,1384	0,945898	0,882157	0,13335	0,363043	no
XLOC_007383	g9513	16,2344	10,1351	-0,679694	-0,859728	0,13905	0,372164	no
XLOC_007384	g9514	0	2,08972	inf	0	1	1	no
XLOC_007385	g9515	0	0	0	0	1	1	no
XLOC_007386	g9516	7,10975	1,83135	-1,95689	-2,06832	0,002	0,0208936	yes
XLOC_007387	g9517	58,68	64,1686	0,128999	0,196694	0,7259	0,869807	no
XLOC_007388	g9520	41,729	79,8701	0,936605	1,57199	0,0061	0,0471669	yes
XLOC_007389	g9522	30,0913	28,9306	-0,0567459	-0,0974002	0,8668	0,939408	no
XLOC_007390	g9518	82,5508	104,906	0,34575	0,491816	0,39725	0,651251	no
XLOC_007391	g9519	121,932	169,887	0,478497	0,819537	0,14405	0,380116	no
XLOC_007392	g9521	187,462	223,974	0,256735	0,415513	0,4601	0,701766	no
XLOC_007393	g9523	20,5821	45,4083	1,14157	1,36197	0,02805	0,135891	no
XLOC_007394	g9524	0	0	0	0	1	1	no
XLOC_007395	g9525	181,765	178,023	-0,0300118	-0,0515751	0,9256	0,967112	no
XLOC_007396	g9526	19,8885	17,9194	-0,150414	-0,230718	0,68255	0,847104	no
XLOC_007397	g9527	0,251178	0,0577606	-2,12055	0	1	1	no
XLOC_007398	g3556	44,1132	34,4555	-0,356475	-0,603329	0,2836	0,546283	no
XLOC_007399	g9528	17,1423	11,6381	-0,558707	-0,800379	0,1616	0,403281	no
XLOC_007400	g9530	18,3116	15,778	-0,21484	-0,254946	0,6591	0,832458	no
XLOC_007401	g9529	198,898	99,3571	-1,00133	-1,59496	0,00545	0,0433002	yes
XLOC_007402	g9531	0	0	0	0	1	1	no
XLOC_007403	g9532	0	0	0	0	1	1	no
XLOC_007404	g9533	0	0	0	0	1	1	no
XLOC_007405	g3557	104,147	106,201	0,0281711	0,0474112	0,93265	0,970604	no
XLOC_007406	g3558	69,5191	70,1886	0,0138257	0,0230469	0,9678	0,985871	no
XLOC_007407	g9534	21,2593	23,558	0,148124	0,210237	0,71705	0,865633	no
XLOC_007408	g3559	56,3343	23,1033	-1,28592	-1,83844	0,00135	0,0156148	yes
XLOC_007409	g3561	36,7921	28,7229	-0,357196	-0,550999	0,337	0,597208	no
XLOC_007410	g3560	3706,81	0	#NAME?	0	1	1	no
XLOC_007411	g9535	0	0	0	0	1	1	no
XLOC_007412	g9536	1413,96	568,534	-1,31442	-1,7303	0,0021	0,0216618	yes
XLOC_007413	g9537	14,2497	12,2973	-0,212592	-0,292957	0,5953	0,792657	no
XLOC_007414	g9538	14,5467	46,5635	1,6785	2,567	5,00E-05	0,00120049	yes
XLOC_007415	g9539	8,91847	76,868	3,10752	3,72986	5,00E-05	0,00120049	yes
XLOC_007416	g3562	87,8143	74,4153	-0,238857	-0,386885	0,48735	0,720794	no
XLOC_007417	g3564	20,3508	32,9976	0,697277	1,16637	0,0425	0,177068	no
XLOC_007418	g3563	75,2129	79,5227	0,0803863	0,137038	0,80655	0,910938	no
XLOC_007419	g3565	12,9075	7,34482	-0,813413	-1,01336	0,0873	0,279246	no
XLOC_007420	g9540	52,6931	20,5553	-1,3581	-2,07139	0,00015	0,00294012	yes
XLOC_007421	g9541	0	0	0	0	1	1	no
XLOC_007422	g9542	5,47487	7,08074	0,371076	0,508957	0,3647	0,624341	no
XLOC_007423	g9543	0	0	0	0	1	1	no
XLOC_007424	g3567	40,061	57,7343	0,527229	0,851433	0,12715	0,35192	no
XLOC_007425	g3570	5,77675	14,9895	1,37562	1,90201	0,0013	0,0151795	yes
XLOC_007426	g3572	1299,35	2038,38	0,649638	0,996198	0,0924	0,289709	no
XLOC_007427	g3573	239,802	484,987	1,0161	0,908139	0,11265	0,328256	no
XLOC_007428	g3566	83,6115	64,5844	-0,372515	-0,604791	0,29565	0,557466	no
XLOC_007429	g3568	137,68	96,435	-0,513688	-0,711225	0,21445	0,469752	no
XLOC_007430	g3569	4,8482	11,2619	1,21593	1,65206	0,0064	0,048795	yes
XLOC_007431	g3571	821,502	1297,78	0,659712	0,959293	0,0954	0,295343	no
XLOC_007432	g3574	44,7888	40,6096	-0,141319	-0,234319	0,6741	0,841661	no
XLOC_007433	g3576	110,51	112,614	0,0272095	0,039244	0,94565	0,975552	no
XLOC_007434	g3575	53,1532	41,29	-0,364363	-0,618334	0,2893	0,55259	no
XLOC_007435	g9544	21,3879	26,0526	0,28463	0,337621	0,5347	0,754094	no
XLOC_007436	g3577	35,6495	27,1233	-0,394348	-0,586869	0,3002	0,562226	no
XLOC_007437	g9545	0	0	0	0	1	1	no
XLOC_007438	g9546	10,1227	25,6485	1,34128	2,0852	0,00015	0,00294012	yes
XLOC_007439	g9547	263,451	214,174	-0,298752	-0,448531	0,4262	0,675809	no
XLOC_007440	g9548	153,197	196,129	0,356414	0,577984	0,29545	0,557303	no
XLOC_007441	g9549	123,873	126,916	0,0350141	0,0594105	0,9175	0,964054	no
XLOC_007442	g3578	211,096	266,235	0,334802	0,575348	0,31055	0,571193	no
XLOC_007443	g3579	63,9845	90,7587	0,504314	0,844726	0,13785	0,370264	no
XLOC_007444	g3580	136,8	105,69	-0,372232	-0,642421	0,2629	0,523242	no
XLOC_007445	g9550	0	0,370737	inf	0	1	1	no
XLOC_007446	g3581	0	0	0	0	1	1	no
XLOC_007447	g9551	433,976	168,866	-1,36174	-2,08908	0,0004	0,0061761	yes
XLOC_007448	g9552	486,102	97,8312	-2,31289	-3,43136	5,00E-05	0,00120049	yes
XLOC_007449	g9553	0	0	0	0	1	1	no
XLOC_007450	g3582	43,1676	65,3463	0,598154	0,80543	0,1683	0,411121	no
XLOC_007451	g3583	23,107	31,5487	0,44925	0,561794	0,3341	0,594729	no

XLOC_007452	g9554	0	0	0	0	1	1	no
XLOC_007453	g9555	0	0	0	0	1	1	no
XLOC_007454	g3584	1,40537	1,33152	-0,0778714	-0,0792995	0,89135	0,951419	no
XLOC_007455	g3585	0	0	0	0	1	1	no
XLOC_007456	g9556	118,769	115,548	-0,0396642	-0,0592508	0,9145	0,963407	no
XLOC_007457	g501	2,37823	1,80846	-0,395128	-0,391742	0,4854	0,719022	no
XLOC_007458	g502	271,8	375,834	0,467551	0,682989	0,2236	0,480997	no
XLOC_007459	g503	477,567	551,328	0,207208	0,315328	0,58055	0,783024	no
XLOC_007460	g504	202,673	409,104	1,01331	1,11853	0,06145	0,223496	no
XLOC_007461	g500	124,346	218,705	0,814633	1,21711	0,03115	0,144699	no
XLOC_007462	g9557	0,719964	0,970735	0,431153	0	1	1	no
XLOC_007463	g9558	0	0	0	0	1	1	no
XLOC_007464	g3586	0	0	0	0	1	1	no
XLOC_007465	g3587	275,579	253,139	-0,122534	-0,196241	0,7295	0,871707	no
XLOC_007466	g3588	37,1705	27,4362	-0,438079	-0,731587	0,20045	0,45251	no
XLOC_007467	g9559	0	0	0	0	1	1	no
XLOC_007468	g505	233,093	1211,03	2,37726	3,00721	5,00E-05	0,00120049	yes
XLOC_007469	g9560	64,6702	130,478	1,01263	1,57516	0,00955	0,0643217	no
XLOC_007470	g9561	0	0	0	0	1	1	no
XLOC_007471	g3589	44,0103	32,0077	-0,459422	-0,738407	0,20485	0,458543	no
XLOC_007472	g9562	56,3376	73,023	0,374252	0,433372	0,45595	0,700583	no
XLOC_007473	g3590	16,451	18,6651	0,182166	0,295773	0,6078	0,801477	no
XLOC_007474	g3594	35,1353	51,2745	0,545321	0,78755	0,164	0,406174	no
XLOC_007475	g3591	361,24	389,967	0,110394	0,178309	0,74875	0,881846	no
XLOC_007476	g3592	24,9536	28,7606	0,204842	0,345804	0,534	0,753431	no
XLOC_007477	g3593	78,112	31,1101	-1,32816	-2,21853	0,0002	0,00367103	yes
XLOC_007478	g9563	0	0	0	0	1	1	no
XLOC_007479	g9564	23,4439	15,0516	-0,639296	-1,06491	0,0577	0,214952	no
XLOC_007480	g9565	0	0	0	0	1	1	no
XLOC_007481	g9566	0	0	0	0	1	1	no
XLOC_007482	g9567	0	0	0	0	1	1	no
XLOC_007483	g9568	0	1,42343	inf	0	1	1	no
XLOC_007484	g9569	0	0	0	0	1	1	no
XLOC_007485	g9570	0	0	0	0	1	1	no
XLOC_007486	g9571	0	0	0	0	1	1	no
XLOC_007487	g9572	0	0	0	0	1	1	no
XLOC_007488	g3595	0	0	0	0	1	1	no
XLOC_007489	g9573	0	0	0	0	1	1	no
XLOC_007490	g9574	39,4559	38,174	-0,0476491	-0,0712117	0,9021	0,957275	no
XLOC_007491	g3596	57,3126	72,3473	0,336086	0,532213	0,3552	0,615917	no
XLOC_007492	g3597	34,989	80,6611	1,20497	1,75695	0,00295	0,0280164	yes
XLOC_007493	g3598	92,4179	94,7	0,0351927	0,0585355	0,9194	0,964481	no
XLOC_007494	g3599	22,0316	13,4131	-0,715928	-1,04624	0,0644	0,23005	no
XLOC_007495	g3600	73,5639	54,539	-0,431709	-0,730726	0,2075	0,462268	no
XLOC_007496	g3602	127,491	141,166	0,146994	0,22929	0,69285	0,853782	no
XLOC_007497	g3604	31,4014	26,2086	-0,260791	-0,445839	0,4305	0,678464	no
XLOC_007498	g3605	80,7004	107,509	0,41381	0,640586	0,2699	0,531359	no
XLOC_007499	g3606	13,505	4,31563	-1,64585	-2,30022	5,00E-05	0,00120049	yes
XLOC_007500	g3608	215,516	408,158	0,921335	1,16832	0,0431	0,178282	no
XLOC_007501	g3610	191,426	303,282	0,663876	0,806161	0,1247	0,348082	no
XLOC_007502	g3612	56,8783	37,4795	-0,601776	-1,03235	0,06975	0,242544	no
XLOC_007503	g3613	7,7464	12,2754	0,664174	0,975491	0,0821	0,269639	no
XLOC_007504	g3601	3419,1	5785,29	0,758771	1,26262	0,02805	0,135891	no
XLOC_007505	g3603	300,291	239,178	-0,328278	-0,558485	0,3453	0,605488	no
XLOC_007506	g3607	852,388	1208,09	0,503148	0,592243	0,2845	0,546579	no
XLOC_007507	g3609	3,58274	2,28717	-0,647498	-0,74307	0,1994	0,451802	no
XLOC_007508	g3611	125,684	119,161	-0,0768927	-0,127877	0,8219	0,918997	no
XLOC_007509	g3614	147,645	120,773	-0,289838	-0,497671	0,37795	0,636045	no
XLOC_007510	g9575	36,6747	33,4985	-0,130691	-0,217997	0,7021	0,858715	no
XLOC_007511	g9577	31,8425	27,9984	-0,185607	-0,316273	0,57335	0,779376	no
XLOC_007512	g9579	74,2351	74,6041	0,00715353	0,0119536	0,98515	0,992425	no
XLOC_007513	g9581	38,1219	56,4489	0,566326	0,868843	0,12775	0,352984	no
XLOC_007514	g9576	112,529	79,1911	-0,506889	-0,852282	0,14055	0,374038	no
XLOC_007515	g9578	1195,48	768,806	-0,636905	-1,01348	0,08235	0,27028	no
XLOC_007516	g9580	6,28599	8,03495	0,35415	0,487428	0,39275	0,647658	no
XLOC_007517	g9582	0	0	0	0	1	1	no
XLOC_007518	g9583	1,28902	5,06823	1,9752	2,399	0,00015	0,00294012	yes
XLOC_007519	g9584	0	0	0	0	1	1	no
XLOC_007520	g9585	65,1929	39,1569	-0,735449	-1,14728	0,03955	0,168788	no
XLOC_007521	g9586	0,477822	0,708117	0,567513	0	1	1	no
XLOC_007522	g9587	0	0	0	0	1	1	no
XLOC_007523	g9588	73,5491	87,2473	0,246403	0,39116	0,4825	0,716707	no
XLOC_007524	g9589	87,4865	77,6597	-0,171895	-0,286956	0,61935	0,808526	no
XLOC_007525	g9590	0	5,31015	inf	0	1	1	no
XLOC_007526	g9591	0	0	0	0	1	1	no
XLOC_007527	g9592	0	0	0	0	1	1	no
XLOC_007528	g9593	0	0	0	0	1	1	no
XLOC_007529	g9594	189,542	168,42	-0,170448	-0,283586	0,62565	0,812361	no
XLOC_007530	g3615	2,87914	3,19432	0,149873	0,189666	0,73255	0,873757	no
XLOC_007531	g37	0	0	0	0	1	1	no
XLOC_007532	g9595	69,7362	200,087	1,52065	2,4691	5,00E-05	0,00120049	yes
XLOC_007533	g9596	9,30399	11,113	0,256323	0,259052	0,6558	0,831391	no
XLOC_007534	g3616	0	0,0161604	inf	0	1	1	no
XLOC_007535	g9597	358,329	643,977	0,845722	1,4301	0,0151	0,0888981	no
XLOC_007536	g9598	0	0	0	0	1	1	no
XLOC_007537	g9600	44,4436	84,275	0,92313	1,49276	0,0091	0,0622298	no
XLOC_007538	g9599	5,29343	8,03579	0,602238	0,744628	0,1824	0,42989	no
XLOC_007539	g3617	15,2231	18,5841	0,287807	0,389042	0,48015	0,715489	no
XLOC_007540	g3618	277,626	255,527	-0,119666	-0,159758	0,7744	0,894738	no
XLOC_007541	g3619	23,6031	15,6991	-0,588292	-0,699351	0,2354	0,49341	no
XLOC_007542	g9601	15,2029	13,6478	-0,155678	-0,204367	0,7167	0,865633	no
XLOC_007543	g9602	0	0	0	0	1	1	no

XLOC_007544	g9605	16,4585	8,99291	-0,871974	-1,16565	0,03875	0,166386	no
XLOC_007545	g9603	30,5694	26,189	-0,223125	-0,307827	0,60475	0,79937	no
XLOC_007546	g9604	2712,51	5804,61	1,09757	1,45807	0,00895	0,0617198	no
XLOC_007547	g3624	151,689	214,319	0,498643	0,745224	0,18095	0,428382	no
XLOC_007548	g3620	78,8367	67,9326	-0,214765	-0,362722	0,5203	0,744115	no
XLOC_007549	g3621	18,4566	36,4997	0,983747	1,59206	0,00625	0,0479867	yes
XLOC_007550	g3622	74,4395	93,4165	0,327609	0,54394	0,33525	0,595523	no
XLOC_007551	g3623	282,864	201,755	-0,487504	-0,78997	0,1654	0,407995	no
XLOC_007552	g9606	130,127	107,426	-0,276573	-0,419351	0,46945	0,707598	no
XLOC_007553	g3625	189,031	235,352	0,316198	0,401209	0,49385	0,724687	no
XLOC_007554	g9607	0	0	0	0	1	1	no
XLOC_007555	g9608	5,50382	3,99411	-0,46256	-0,356933	0,5167	0,741989	no
XLOC_007556	g9609	0	0	0	0	1	1	no
XLOC_007557	g9610	124,608	121,434	-0,037221	-0,0570577	0,921	0,965078	no
XLOC_007558	g507	19,2377	44,803	1,21966	1,84037	0,00115	0,0138564	yes
XLOC_007559	g506	44,7182	44,0812	-0,0206967	-0,0304554	0,95765	0,981334	no
XLOC_007560	g508	18,6866	17,3041	-0,110894	-0,150574	0,7897	0,902882	no
XLOC_007561	g509	50,7144	50,4067	-0,0087804	-0,014249	0,97855	0,989941	no
XLOC_007562	g9611	72,5664	69,5265	-0,0617397	-0,079846	0,88885	0,950507	no
XLOC_007563	g9612	0	0,380935	inf	0	1	1	no
XLOC_007564	g9613	102,272	117,13	0,195709	0,307027	0,59495	0,792473	no
XLOC_007565	g9614	0	0	0	0	1	1	no
XLOC_007566	g3626	2,30124	5,19971	1,17602	1,28138	0,0315	0,145704	no
XLOC_007567	g9615	0,757338	1,21665	0,683906	0,707149	0,22715	0,48555	no
XLOC_007568	g3629	54,9336	47,3558	-0,214147	-0,3465	0,5331	0,752918	no
XLOC_007569	g3630	92,619	61,8128	-0,583403	-0,974783	0,0889	0,282341	no
XLOC_007570	g3632	14,823	15,0001	0,0171407	0,0294083	0,9584	0,98159	no
XLOC_007571	g3627	48,9682	73,2481	0,580946	0,993757	0,0794	0,263237	no
XLOC_007572	g3628	31,4985	53,6404	0,768038	1,21101	0,0318	0,14654	no
XLOC_007573	g3631	86,3697	105,331	0,286331	0,384247	0,4921	0,723707	no
XLOC_007574	g9616	13,8915	9,36647	-0,568623	-0,702667	0,2043	0,45752	no
XLOC_007575	g3633	13,626	18,2569	0,422076	0,610158	0,28855	0,551993	no
XLOC_007576	g3634	0	0	0	0	1	1	no
XLOC_007577	g9617	0	0	0	0	1	1	no
XLOC_007578	g3635	0	0	0	0	1	1	no
XLOC_007579	g3636	0	0	0	0	1	1	no
XLOC_007580	g9618	0	0	0	0	1	1	no
XLOC_007581	g9619	1,7418	2,283	0,390351	0,312484	0,5934	0,79163	no
XLOC_007582	g9620	10,2773	9,82561	-0,0648395	-0,0617082	0,90635	0,959297	no
XLOC_007583	g9621	4,38257	8,33297	0,927053	1,41097	0,01395	0,0844568	no
XLOC_007584	g9622	0	0	0	0	1	1	no
XLOC_007585	g9623	193,687	173,126	-0,161904	-0,217511	0,7136	0,86449	no
XLOC_007586	g9625	183,81	144,51	-0,347047	-0,529351	0,3558	0,616435	no
XLOC_007587	g9624	92,2194	134,093	0,540089	0,822749	0,1473	0,384102	no
XLOC_007588	g9626	0	0	0	0	1	1	no
XLOC_007589	g9627	0	0	0	0	1	1	no
XLOC_007590	g3640	333,981	483,206	0,532874	0,742386	0,1939	0,445091	no
XLOC_007591	g3637	261,965	250,548	-0,0642853	-0,112278	0,84285	0,928203	no
XLOC_007592	g3638	19,3354	24,6974	0,353118	0,594492	0,2997	0,561973	no
XLOC_007593	g3639	43,2672	38,1241	-0,182568	-0,308061	0,58865	0,789071	no
XLOC_007594	g9628	43,4595	31,8866	-0,446722	-0,646061	0,27085	0,532269	no
XLOC_007595	g9629	0,0971563	0	#NAME?	0	1	1	no
XLOC_007596	g9630	0	0	0	0	1	1	no
XLOC_007597	g9631	18,7285	6,90501	-1,43952	-2,11022	0,00015	0,00294012	yes
XLOC_007598	g3641	1,34595	2,1771	0,693784	0,707109	0,2302	0,487612	no
XLOC_007599	g3642	14,9226	12,2044	-0,2901	-0,425413	0,46445	0,705296	no
XLOC_007600	g3643	40,9415	37,5405	-0,125117	-0,192998	0,72635	0,869908	no
XLOC_007601	g3644	197,036	189,68	-0,054896	-0,0894586	0,8766	0,944401	no
XLOC_007602	g9636	99,619	66,6712	-0,579358	-0,846579	0,1449	0,380867	no
XLOC_007603	g9639	53,0378	29,8018	-0,831622	-1,30577	0,0235	0,122036	no
XLOC_007604	g9632	119,862	101,99	-0,232942	-0,391614	0,49195	0,723707	no
XLOC_007605	g9633	20,9147	9,09413	-1,20151	-1,77643	0,0028	0,0269305	yes
XLOC_007606	g9634	51,6072	38,652	-0,41703	-0,636875	0,2565	0,516048	no
XLOC_007607	g9635	25,7271	32,3726	0,331486	0,467878	0,3993	0,653194	no
XLOC_007608	g9637	29,2731	29,5487	0,0135181	0,0222062	0,96735	0,985566	no
XLOC_007609	g9638	9,90769	6,27709	-0,658454	-0,918825	0,116	0,333955	no
XLOC_007610	g3645	1,22867	2,67762	1,12385	1,03003	0,0838	0,273013	no
XLOC_007611	g3646	0	0	0	0	1	1	no
XLOC_007612	g3647	0	0	0	0	1	1	no
XLOC_007613	g9640	0	0	0	0	1	1	no
XLOC_007614	g9641	0	0	0	0	1	1	no
XLOC_007615	g9642	0	0	0	0	1	1	no
XLOC_007616	g9643	35,6236	32,4065	-0,136548	-0,206632	0,72555	0,869807	no
XLOC_007617	g9644	0,0233794	0,0965101	2,04544	0	1	1	no
XLOC_007618	g512	24,2836	22,3654	-0,118716	-0,19324	0,7301	0,871788	no
XLOC_007619	g513	54,2968	57,856	0,0915987	0,156941	0,7869	0,901522	no
XLOC_007620	g514	34,6534	30,0817	-0,204111	-0,3006	0,5982	0,795009	no
XLOC_007621	g515	31,8037	24,2693	-0,39006	-0,583461	0,3228	0,583345	no
XLOC_007622	g510	208,363	205,435	-0,0204182	-0,0330372	0,9552	0,98046	no
XLOC_007623	g511	6,49757	4,51377	-0,525568	-0,611735	0,26425	0,524866	no
XLOC_007624	g516	118,119	156,397	0,404976	0,655878	0,25625	0,515756	no
XLOC_007625	g517	27,7188	19,6556	-0,495927	-0,807878	0,1501	0,387279	no
XLOC_007626	g518	141,854	482,455	1,76599	2,66355	5,00E-05	0,00120049	yes
XLOC_007627	g519	46,618	40,395	-0,206712	-0,344307	0,5413	0,759787	no
XLOC_007628	g3650	206,027	381,152	0,887537	1,33027	0,02395	0,123459	no
XLOC_007629	g3651	59,3257	47,6363	-0,316595	-0,541681	0,33115	0,592007	no
XLOC_007630	g3652	182,44	214,863	0,235993	0,284822	0,60235	0,798521	no
XLOC_007631	g3654	0,573366	3,96179	2,78862	2,10562	0,0087	0,0603347	no
XLOC_007632	g3657	5,28496	5,65756	0,0982881	0,115884	0,8399	0,926574	no
XLOC_007633	g3648	461,686	735,016	0,670865	0,822564	0,14535	0,381641	no
XLOC_007634	g3649	88,8604	96,8394	0,124053	0,208536	0,71505	0,865069	no
XLOC_007635	g3653	49,0237	53,9954	0,139357	0,240031	0,6732	0,841348	no

XLOC_007636	g3655	85,5296	134,383	0,651854	1,09575	0,0555	0,210104	no
XLOC_007637	g3656	23,6526	56,5168	1,25668	1,84988	0,0011	0,0134856	yes
XLOC_007638	g3658	30,5829	64,2999	1,07209	1,85025	0,00245	0,0243512	yes
XLOC_007639	g3659	0	0	0	0	1	1	no
XLOC_007640	g9645	46,2196	34,4936	-0,422175	-0,711561	0,1958	0,447153	no
XLOC_007641	g3660	31,8717	29,7094	-0,101357	-0,159342	0,7839	0,900444	no
XLOC_007642	g3661	46,412	17,7137	-1,38963	-1,84108	0,0017	0,0185283	yes
XLOC_007643	g3662	27,9144	7,1781	-1,95933	-2,74298	5,00E-05	0,00120049	yes
XLOC_007644	g9646	0,690737	1,01583	0,556452	0	1	1	no
XLOC_007645	g3663	81,5046	64,0475	-0,347739	-0,509922	0,37595	0,634261	no
XLOC_007646	g3665	49,449	56,2447	0,185773	0,32186	0,5765	0,780604	no
XLOC_007647	g3667	1081,39	1072,49	-0,0119199	-0,0160379	0,97675	0,98873	no
XLOC_007648	g3668	78,6398	75,5557	-0,0577187	-0,09926	0,8606	0,935634	no
XLOC_007649	g3664	83,8145	64,5061	-0,377765	-0,633236	0,2708	0,532269	no
XLOC_007650	g3666	161,422	340,399	1,07639	1,80999	0,00185	0,01964	yes
XLOC_007651	g3669	104,644	133,45	0,350803	0,577565	0,32695	0,587816	no
XLOC_007652	g3670	29,5526	30,5844	0,0495105	0,0823601	0,8898	0,950902	no
XLOC_007653	g3671	1,05098	2,72889	1,37658	1,31746	0,0223	0,117671	no
XLOC_007654	g3672	21,2149	31,893	0,588163	0,832238	0,1526	0,390446	no
XLOC_007655	g3673	87,4569	135,377	0,630343	1,08638	0,05665	0,213306	no
XLOC_007656	g3674	407,821	283,388	-0,525157	-0,796502	0,18655	0,434826	no
XLOC_007657	g9647	125,232	68,0037	-0,880916	-1,41111	0,0154	0,0901777	no
XLOC_007658	g9648	474,201	1272,37	1,42394	1,9178	0,00155	0,0171215	yes
XLOC_007659	g3675	0	0	0	0	1	1	no
XLOC_007660	g9649	0	0	0	0	1	1	no
XLOC_007661	g3676	0	0	0	0	1	1	no
XLOC_007662	g520	0	0	0	0	1	1	no
XLOC_007663	g9650	39,0099	27,7224	-0,492787	-0,772963	0,17945	0,426373	no
XLOC_007664	g9651	0	0	0	0	1	1	no
XLOC_007665	g3677	0,533222	1,62992	1,612	0,956056	0,09155	0,287963	no
XLOC_007666	g3678	8,34029	8,30241	-0,00656756	-0,0102018	0,98425	0,992379	no
XLOC_007667	g3679	0	0	0	0	1	1	no
XLOC_007668	g9652	0	0	0	0	1	1	no
XLOC_007669	g9653	28,058	30,6656	0,128207	0,156142	0,7839	0,900444	no
XLOC_007670	g3681	30,2438	31,5703	0,0619305	0,0964647	0,86015	0,935634	no
XLOC_007671	g3680	0	0	0	0	1	1	no
XLOC_007672	g3682	0	0	0	0	1	1	no
XLOC_007673	g3684	312,887	68,9367	-2,1823	-3,42801	5,00E-05	0,00120049	yes
XLOC_007674	g3683	18,9768	15,8668	-0,258219	-0,396094	0,4824	0,716707	no
XLOC_007675	g9654	12,2472	24,4068	0,994837	1,39577	0,017	0,0966088	no
XLOC_007676	g9655	0	0	0	0	1	1	no
XLOC_007677	g3685	0	0,178076	inf	0	1	1	no
XLOC_007678	g9656	27,7545	21,0482	-0,399024	-0,598438	0,2985	0,560566	no
XLOC_007679	g9657	8,65864	23,872	1,46311	1,66789	0,0047	0,03928	yes
XLOC_007680	g9658	9,58623	13,1043	0,451003	0,689325	0,22305	0,480342	no
XLOC_007681	g3686	1,95004	4,61329	1,24229	1,14594	0,05095	0,199133	no
XLOC_007682	g9659	4,35465	3,03322	-0,521706	-0,405552	0,47815	0,713917	no
XLOC_007683	g9660	1,76206	9,43992	2,42151	1,62514	0,02025	0,109865	no
XLOC_007684	g9661	0	0	0	0	1	1	no
XLOC_007685	g9662	32,6073	31,6603	-0,0425201	-0,0611203	0,91425	0,963407	no
XLOC_007686	g9663	37,8739	31,1101	-0,283821	-0,445334	0,4331	0,68016	no
XLOC_007687	g9664	0	0	0	0	1	1	no
XLOC_007688	g9665	16,9234	18,67	0,141707	0,212759	0,7097	0,862318	no
XLOC_007689	g9666	18,8103	16,6913	-0,172427	-0,24584	0,6637	0,835468	no
XLOC_007690	g9667	9,43325	13,2913	0,494654	0,682019	0,2229	0,480228	no
XLOC_007691	g9668	2,98404	3,35932	0,170903	0,225172	0,6953	0,855038	no
XLOC_007692	g9669	39,3458	32,0951	-0,293856	-0,503117	0,3753	0,633565	no
XLOC_007693	g9670	104,693	101,277	-0,0478485	-0,0808395	0,8869	0,949663	no
XLOC_007694	g9671	21,4921	15,4345	-0,477647	-0,787469	0,17025	0,41403	no
XLOC_007695	g9672	21,8144	39,1777	0,844755	1,41155	0,0121	0,076462	no
XLOC_007696	g3687	0	0	0	0	1	1	no
XLOC_007697	g9673	0	0	0	0	1	1	no
XLOC_007698	g3688	53,2631	44,4886	-0,259702	-0,42588	0,4534	0,698162	no
XLOC_007699	g9674	153,495	149,59	-0,0371749	-0,0562994	0,9207	0,96497	no
XLOC_007700	g9677	16,2779	16,1344	-0,0127739	-0,0212914	0,9696	0,986579	no
XLOC_007701	g9681	15,6243	22,0811	0,499021	0,808375	0,1478	0,384374	no
XLOC_007702	g9683	188,061	199,259	0,0834448	0,13594	0,8153	0,916046	no
XLOC_007703	g9675	39,7477	42,4245	0,0940261	0,1571	0,7772	0,895827	no
XLOC_007704	g9676	67,9122	68,0029	0,00192723	0,00309609	0,99555	0,997682	no
XLOC_007705	g9678	43,3075	18,6372	-1,21643	-1,78131	0,0021	0,0216618	yes
XLOC_007706	g9679	50,9492	58,9906	0,211426	0,357546	0,5306	0,751116	no
XLOC_007707	g9680	12,7048	18,5089	0,54285	0,808556	0,1553	0,392851	no
XLOC_007708	g9682	35,1979	34,9009	-0,0122259	-0,0200431	0,97235	0,987127	no
XLOC_007709	g9684	70,76	64,7034	-0,129093	-0,221886	0,69735	0,856211	no
XLOC_007710	g9685	70,9585	72,2287	0,0255971	0,0387108	0,94525	0,975499	no
XLOC_007711	g9686	39,022	51,7496	0,40726	0,669731	0,2332	0,49079	no
XLOC_007712	g9688	139,516	144,339	0,0490265	0,0832611	0,8818	0,947197	no
XLOC_007713	g9690	22,5907	31,9228	0,498857	0,831079	0,1422	0,376999	no
XLOC_007714	g9692	83,9742	72,5015	-0,211935	-0,35444	0,5371	0,75585	no
XLOC_007715	g9687	95,2361	102,223	0,102139	0,162594	0,77075	0,893264	no
XLOC_007716	g9689	68,7031	49,8955	-0,461463	-0,793839	0,1675	0,410802	no
XLOC_007717	g9691	7,40749	9,43567	0,34914	0,570104	0,31645	0,577009	no
XLOC_007718	g9693	53,7598	72,376	0,428983	0,725156	0,20755	0,462268	no
XLOC_007719	g3692	3,35497	2,94482	-0,188117	-0,207182	0,71535	0,865069	no
XLOC_007720	g3689	16,6718	22,3596	0,423485	0,626478	0,26645	0,527422	no
XLOC_007721	g3690	1240,1	490,493	-1,33816	-1,97326	0,00185	0,01964	yes
XLOC_007722	g3691	6,49589	10,4963	0,692281	1,00332	0,0893	0,283062	no
XLOC_007723	g9694	18,1395	66,2001	1,8677	0,999944	0,1026	0,308736	no
XLOC_007724	g3693	98,5611	77,4796	-0,347201	-0,550451	0,3175	0,578204	no
XLOC_007725	g3694	12,1255	33,6302	1,47171	2,10394	0,00025	0,00430702	yes
XLOC_007726	g3695	20,1268	18,6774	-0,10782	-0,157136	0,78305	0,899946	no
XLOC_007727	g3696	36,6539	27,4063	-0,419462	-0,720743	0,2071	0,46179	no

XLOC_007728	g3697	7,03711	11,1378		0,662407	0,917515	0,10665	0,317172	no
XLOC_007729	g9695	13,0594	11,1803		-0,224128	-0,238785	0,68415	0,848063	no
XLOC_007730	g9696	20,4805	11,8704		-0,786881	-1,11959	0,0468	0,187812	no
XLOC_007731	g3698	24,7239	27,9723		0,17809	0,279006	0,6267	0,812951	no
XLOC_007732	g3699	11,1454	12,9389		0,215261	0,331096	0,5614	0,77218	no
XLOC_007733	g9697	30,3765	8,05759		-1,91454	-2,02246	0,00285	0,0272512	yes
XLOC_007734	g9698	47,0837	59,0052		0,325614	0,464682	0,4135	0,665995	no
XLOC_007735	g9699	2979,96	2329,08		-0,355535	-0,519045	0,3827	0,639193	no
XLOC_007736	g9700	222,415	157,431		-0,498541	-0,853385	0,136	0,367406	no
XLOC_007737	g9701	267,769	674,75		1,33337	1,84121	0,0027	0,0261737	yes
XLOC_007738	g9702	0	0		0	0	1	1	no
XLOC_007739	g9703	0	0		0	0	1	1	no
XLOC_007740	g3700	15,5257	12,5581		-0,306039	-0,413194	0,47295	0,710365	no
XLOC_007741	g9704	6,75979	20,8315		1,62372	1,98112	0,002	0,0208936	yes
XLOC_007742	g9705	7,19369	8,41618		0,226434	0,303909	0,5943	0,792155	no
XLOC_007743	g9706	17,4967	11,2188		-0,641165	-0,968264	0,09925	0,302119	no
XLOC_007744	g9708	22,5743	16,8118		-0,425207	-0,630626	0,26395	0,524581	no
XLOC_007745	g9707	31,7762	26,5792		-0,257652	-0,400876	0,4854	0,719022	no
XLOC_007746	g9709	0	0		0	0	1	1	no
XLOC_007747	g9710	0	0		0	0	1	1	no
XLOC_007748	g9711	43,0302	57,425		0,416328	0,466217	0,4081	0,660548	no
XLOC_007749	g9712	89,301	26,9341		-1,72924	-2,69563	5,00E-05	0,00120049	yes
XLOC_007750	g9713	50,5051	96,601		0,935609	1,44904	0,0104	0,0684046	no
XLOC_007751	g9714	19,9227	19,7067		-0,0157258	-0,0262081	0,96375	0,983889	no
XLOC_007752	g9715	218,996	317,814		0,537274	0,797074	0,15375	0,391857	no
XLOC_007753	g3701	0	0,276863	inf		0	1	1	no
XLOC_007754	g3702	0	0		0	0	1	1	no
XLOC_007755	g3703	0	0		0	0	1	1	no
XLOC_007756	g9716	0	0		0	0	1	1	no
XLOC_007757	g9717	0	0		0	0	1	1	no
XLOC_007758	g9718	87,4226	120,745		0,465889	0,465572	0,418	0,669829	no
XLOC_007759	g9719	0,513771	0,60067		0,225447	0	1	1	no
XLOC_007760	g9720	23,3313	37,4874		0,684137	0,974831	0,08575	0,276611	no
XLOC_007761	g9721	0	0		0	0	1	1	no
XLOC_007762	g9722	2,76102	4,97163		0,84852	0	1	1	no
XLOC_007763	g9723	22,5074	22,281		-0,0145797	-0,0222422	0,96675	0,985414	no
XLOC_007764	g9724	0	0		0	0	1	1	no
XLOC_007765	g3704	120,859	149,553		0,307329	0,521802	0,36275	0,622826	no
XLOC_007766	g3707	47,3677	29,0533		-0,705203	-1,08454	0,0617	0,223825	no
XLOC_007767	g3708	34,6444	37,9587		0,131807	0,219451	0,69265	0,853642	no
XLOC_007768	g3709	2,09956	1,95305		-0,104362	-0,13131	0,81285	0,914613	no
XLOC_007769	g3705	73,4464	116,116		0,660799	1,06023	0,06205	0,224514	no
XLOC_007770	g3706	9,93455	13,0598		0,394603	0,580024	0,3223	0,582656	no
XLOC_007771	g9725	0	0,370936	inf		0	1	1	no
XLOC_007772	g9726	0	0		0	0	1	1	no
XLOC_007773	g3711	18,6019	13,9293		-0,417326	-0,688287	0,23585	0,493721	no
XLOC_007774	g3713	67,1596	47,7972		-0,490668	-0,80891	0,15185	0,389644	no
XLOC_007775	g3714	18,8194	17,2875		-0,122495	-0,186289	0,73685	0,87578	no
XLOC_007776	g3710	6,36069	3,149		-1,01429	-1,2018	0,0458	0,185237	no
XLOC_007777	g3712	32,5613	36,3457		0,158625	0,242525	0,6722	0,840955	no
XLOC_007778	g9727	0	0		0	0	1	1	no
XLOC_007779	g3715	38,4379	30,3168		-0,342414	-0,447399	0,41385	0,666012	no
XLOC_007780	g9728	0	0		0	0	1	1	no
XLOC_007781	g3716	44,1756	87,0924		0,979298	1,61935	0,00515	0,0418998	yes
XLOC_007782	g3717	29,3977	24,7054		-0,250878	-0,401257	0,4821	0,716438	no
XLOC_007783	g3718	90,4088	125,332		0,471219	0,734397	0,17785	0,424626	no
XLOC_007784	g9731	30,8971	42,7104		0,467116	0,762343	0,17895	0,42632	no
XLOC_007785	g9729	70,0325	44,5944		-0,651163	-1,11821	0,0503	0,197657	no
XLOC_007786	g9730	26,8268	20,674		-0,375852	-0,501721	0,54395	0,761452	no
XLOC_007787	g3719	81,4933	114,494		0,490517	0,841642	0,1445	0,380558	no
XLOC_007788	g3721	23,1254	24,5127		0,0840492	0,142277	0,8033	0,908803	no
XLOC_007789	g3722	11,8065	16,7845		0,507547	0,810643	0,15805	0,398064	no
XLOC_007790	g3720	9,67026	10,4209		0,107851	0,147182	0,79465	0,905607	no
XLOC_007791	g9734	22,6779	19,8261		-0,193886	-0,318952	0,5751	0,780604	no
XLOC_007792	g9732	37,0554	24,346		-0,605997	-1,00369	0,0753	0,253495	no
XLOC_007793	g9733	30,3175	20,9244		-0,534966	-0,855602	0,13585	0,367203	no
XLOC_007794	g9735	28,6778	22,6007		-0,343568	-0,528393	0,34625	0,606524	no
XLOC_007795	g3723	0	0		0	0	1	1	no
XLOC_007796	g9736	110,351	112,873		0,0326037	0,0563628	0,9185	0,964054	no
XLOC_007797	g38	0,200037	0,0716531		-1,48117	0	1	1	no
XLOC_007798	g39	0	0		0	0	1	1	no
XLOC_007799	g9737	3,67251	1,0155		-1,85458	-1,66981	0,0263	0,130834	no
XLOC_007800	g3724	53,0513	36,4045		-0,54327	-0,908646	0,11515	0,332972	no
XLOC_007801	g3725	7,37336	13,7693		0,901064	1,31837	0,0229	0,119743	no
XLOC_007802	g3727	9,96723	10,9277		0,132721	0,182978	0,74865	0,881846	no
XLOC_007803	g3726	16,9049	10,3982		-0,701102	-1,12886	0,0476	0,189628	no
XLOC_007804	g9738	0	0		0	0	1	1	no
XLOC_007805	g9740	3,64242	4,00173		0,135723	0,13926	0,7983	0,907117	no
XLOC_007806	g9739	5,10251	8,06443		0,660365	1,33046	0,61995	0,808702	no
XLOC_007807	g9741	0	0		0	0	1	1	no
XLOC_007808	g3728	74,1071	91,1464		0,298572	0,322903	0,54265	0,76057	no
XLOC_007809	g3729	14,0283	12,1822		-0,203556	-0,311874	0,59125	0,790276	no
XLOC_007810	g3730	140,833	81,7795		-0,784175	-1,20042	0,03115	0,144699	no
XLOC_007811	g9742	3,57018	5,57055		0,641826	0,764278	0,1897	0,43956	no
XLOC_007812	g3731	41,103	41,5699		0,0162969	0,0242128	0,96415	0,984142	no
XLOC_007813	g3732	38,8822	30,1598		-0,366481	-0,565161	0,3144	0,575151	no
XLOC_007814	g9743	13,8213	11,5359		-0,260769	-0,33266	0,55925	0,770892	no
XLOC_007815	g9744	8,43146	5,93754		-0,505916	-0,665803	0,25015	0,508587	no
XLOC_007816	g9745	0	0		0	0	1	1	no
XLOC_007817	g3733	8,22201	10,3647		0,334111	0,425909	0,4488	0,694314	no
XLOC_007818	g9746	111,718	106,743		-0,0657226	-0,100274	0,8565	0,93516	no
XLOC_007819	g9748	70,3363	176,507		1,32738	2,09478	0,0005	0,00733931	yes

XLOC_007820	g9750	27,3853	27,0149	-0,0196476	-0,0323769	0,95375	0,979484	no
XLOC_007821	g9752	120,565	73,4411	-0,715149	-1,19283	0,0325	0,148719	no
XLOC_007822	g9753	118,342	122,188	0,0461511	0,0714354	0,90115	0,95668	no
XLOC_007823	g9755	67,5146	117,45	0,798776	1,19108	0,0356	0,157901	no
XLOC_007824	g9758	126,75	147,927	0,222906	0,378876	0,49635	0,726367	no
XLOC_007825	g9760	38,8967	30,783	-0,337515	-0,547297	0,3291	0,590276	no
XLOC_007826	g9761	104,965	104,468	-0,00685376	-0,0109675	0,9847	0,992379	no
XLOC_007827	g9747	69,0285	59,3392	-0,218207	-0,358059	0,5147	0,740817	no
XLOC_007828	g9749	11,6886	30,8501	1,40017	1,78656	0,0033	0,0304282	yes
XLOC_007829	g9751	48,7528	60,0242	0,300061	0,491842	0,38525	0,640945	no
XLOC_007830	g9754	67,8902	70,3454	0,0512533	0,0878933	0,88035	0,946261	no
XLOC_007831	g9756	151,426	173,719	0,198142	0,344183	0,55015	0,765215	no
XLOC_007832	g9757	72,9107	74,8954	0,0387458	0,065532	0,91115	0,962529	no
XLOC_007833	g9759	42,2492	75,1338	0,830539	1,40621	0,0158	0,0916998	no
XLOC_007834	g9762	49,7465	53,4593	0,103846	0,171964	0,76645	0,890609	no
XLOC_007835	g9763	141,784	47,0349	-1,59189	-2,29662	0,0002	0,00367103	yes
XLOC_007836	g9764	51,7233	33,2063	-0,639358	-1,01668	0,0708	0,244636	no
XLOC_007837	g9765	47,8025	38,3869	-0,316472	-0,327479	0,56755	0,776339	no
XLOC_007838	g3734	24,8397	27,9811	0,171807	0,237596	0,67225	0,840955	no
XLOC_007839	g9766	25,8189	9,03881	-1,51422	-1,63318	0,16885	0,41185	no
XLOC_007840	g9767	108,82	363,918	1,74167	2,05863	0,0005	0,00733931	yes
XLOC_007841	g9768	0	0	0	0	1	1	no
XLOC_007842	g9769	0	0	0	0	1	1	no
XLOC_007843	g3735	1,00938	1,75627	0,799048	0,792394	0,166	0,409064	no
XLOC_007844	g3737	5,48788	7,77138	0,501922	0,455169	0,4323	0,679357	no
XLOC_007845	g3736	844,796	678,836	-0,31554	-0,406212	0,4811	0,715676	no
XLOC_007846	g9770	18,7237	16,7078	-0,164346	-0,220134	0,6931	0,853876	no
XLOC_007847	g9772	114,445	52,72	-1,11824	-1,88349	0,00155	0,0171215	yes
XLOC_007848	g9771	12,6182	9,76879	-0,369256	-0,552484	0,32625	0,587095	no
XLOC_007849	g521	23,5401	27,8342	0,24174	0,394859	0,481	0,71567	no
XLOC_007850	g523	53,6276	57,8381	0,109045	0,175103	0,76215	0,887899	no
XLOC_007851	g524	38,9642	51,9041	0,413699	0,691653	0,2473	0,506238	no
XLOC_007852	g522	106,693	28,7447	-1,89209	-3,11868	5,00E-05	0,00120049	yes
XLOC_007853	g525	35,8188	36,1794	0,0144521	0,0208387	0,97045	0,98683	no
XLOC_007854	g9773	0,0797267	0	#NAME?	0	1	1	no
XLOC_007855	g9774	0,0111908	0	#NAME?	0	1	1	no
XLOC_007856	g9775	0	0	0	0	1	1	no
XLOC_007857	g9776	118,534	154,501	0,382309	0,619249	0,27705	0,538739	no
XLOC_007858	g9777	80,4586	235,533	1,54961	2,20796	0,00035	0,00552572	yes
XLOC_007859	g3738	0	0	0	0	1	1	no
XLOC_007860	g9778	10,6447	5,85958	-0,86126	-0,825854	0,2497	0,508092	no
XLOC_007861	g526	19,9246	13,0875	-0,606356	-0,528622	0,36045	0,620877	no
XLOC_007862	g527	41,8933	13,4773	-1,63619	-2,34522	0,0001	0,00209829	yes
XLOC_007863	g529	78,2251	55,5701	-0,493322	-0,836431	0,14705	0,383949	no
XLOC_007864	g528	9,64236	19,9531	1,04915	1,49923	0,01075	0,0699569	no
XLOC_007865	g9779	0,10145	1,47201	3,85894	0,960569	0,1173	0,335631	no
XLOC_007866	g9780	0,552149	0	#NAME?	0	1	1	no
XLOC_007867	g9781	0	0	0	0	1	1	no
XLOC_007868	g9784	86,099	51,4193	-0,743685	-1,12985	0,0542	0,207099	no
XLOC_007869	g9782	3398,57	7316,8	1,10628	1,66916	0,00445	0,0377692	yes
XLOC_007870	g9783	14,6571	15,0886	0,0418599	0,0665183	0,90005	0,955719	no
XLOC_007871	g9785	23,6577	30,3642	0,360058	0,483962	0,3986	0,65273	no
XLOC_007872	g9786	0	0	0	0	1	1	no
XLOC_007873	g9787	41,916	53,7809	0,359593	0,523324	0,363	0,622866	no
XLOC_007874	g530	0,317329	0,343168	0,112936	0	1	1	no
XLOC_007875	g3739	0	0	0	0	1	1	no
XLOC_007876	g3740	121,495	96,1182	-0,338016	-0,525271	0,3564	0,61704	no
XLOC_007877	g3741	1,07021	0,951553	-0,169543	-0,128888	0,8271	0,921084	no
XLOC_007878	g3743	2,1681	0	#NAME?	0	1	1	no
XLOC_007879	g3742	13,9389	16,0807	0,206212	0,24986	0,657	0,831946	no
XLOC_007880	g9788	1081,75	956,045	-0,178221	-0,303407	0,58635	0,787144	no
XLOC_007881	g3744	6,4929	3,44452	-0,914562	-0,948472	0,11555	0,333246	no
XLOC_007882	g9789	106,611	106,935	0,00437665	0,00681852	0,99105	0,995205	no
XLOC_007883	g9790	26,4639	36,4644	0,462465	0,788923	0,1632	0,405236	no
XLOC_007884	g9791	0	0	0	0	1	1	no
XLOC_007885	g3745	56,9539	58,2132	0,0315527	0,0543124	0,92555	0,967112	no
XLOC_007886	g9792	0	0	0	0	1	1	no
XLOC_007887	g3746	12,175	44,0186	1,85419	2,9159	5,00E-05	0,00120049	yes
XLOC_007888	g9793	32,4211	41,8581	0,368572	0,606642	0,28585	0,548358	no
XLOC_007889	g3747	9,26555	7,97302	-0,21675	-0,279034	0,6343	0,817002	no
XLOC_007890	g9795	84,932	39,5591	-1,1023	-1,69665	0,00345	0,0313115	yes
XLOC_007891	g9794	113,119	80,0925	-0,498108	-0,756667	0,1711	0,415583	no
XLOC_007892	g9796	294,721	125,749	-1,2288	-1,81391	0,00235	0,0236687	yes
XLOC_007893	g9797	0	0	0	0	1	1	no
XLOC_007894	g531	25,2901	26,4349	0,0638704	0,0955142	0,85975	0,935634	no
XLOC_007895	g41	138,728	103,854	-0,417692	-0,707662	0,203	0,455752	no
XLOC_007896	g40	41,5362	290,612	2,80665	4,3062	5,00E-05	0,00120049	yes
XLOC_007897	g42	78,8848	101,688	0,366329	0,645294	0,27005	0,531531	no
XLOC_007898	g9798	0,758125	2,37288	1,64613	0,917473	0,1447	0,380837	no
XLOC_007899	g9799	0,0209725	0,0563599	1,42617	0	1	1	no
XLOC_007900	g9801	33,9053	27,7474	-0,289159	-0,470644	0,41855	0,670062	no
XLOC_007901	g9800	21,2889	24,4313	0,19863	0,264932	0,62745	0,8133	no
XLOC_007902	g9802	82,2315	84,9403	0,0467588	0,0778957	0,89	0,951012	no
XLOC_007903	g3748	29,9169	23,5518	-0,34512	-0,522082	0,36975	0,629256	no
XLOC_007904	g3749	28,1254	20,7216	-0,440734	-0,559973	0,33365	0,594636	no
XLOC_007905	g3750	31,2732	29,3332	-0,0923905	-0,141324	0,80305	0,908624	no
XLOC_007906	g3751	61,8651	74,6759	0,271517	0,398094	0,4817	0,716101	no
XLOC_007907	g9803	103,07	76,0339	-0,43891	-0,658983	0,23925	0,497337	no
XLOC_007908	g9804	13,1937	13,4235	0,0249098	0,0383316	0,94415	0,975337	no
XLOC_007909	g9805	9,62055	13,7647	0,516785	0,599988	0,3013	0,563145	no
XLOC_007910	g9806	1,94659	2,74048	0,49348	0,492212	0,39465	0,648995	no
XLOC_007911	g9807	0	0	0	0	1	1	no

XLOC_007912	g3752	0	0	0	0	1	1	no
XLOC_007913	g9808	0	0	0	0	1	1	no
XLOC_007914	g3753	3,40838	4,31808	0,341306	0,479056	0,40525	0,658649	no
XLOC_007915	g3754	14,7855	14,8936	0,0105111	0,0163899	0,97695	0,98873	no
XLOC_007916	g3755	10,8711	7,01414	-0,632156	-0,755961	0,1894	0,439279	no
XLOC_007917	g3756	0,408922	1,50806	1,88279	1,95265	0,0021	0,0216618	yes
XLOC_007918	g9809	171,269	110,301	-0,63481	-1,03961	0,07255	0,248757	no
XLOC_007919	g3758	19,1893	37,3835	0,9621	1,53907	0,00835	0,0587371	no
XLOC_007920	g3757	31,9641	41,0802	0,36199	0,610748	0,2738	0,535281	no
XLOC_007921	g9810	7,43336	6,97703	-0,0914012	-0,115982	0,84005	0,926574	no
XLOC_007922	g9811	19,1184	28,348	0,56829	0,843634	0,1375	0,369729	no
XLOC_007923	g9812	89,5508	72,4157	-0,306403	-0,477624	0,40205	0,655836	no
XLOC_007924	g9813	3,7771	1,18539	-1,67192	-1,21905	0,05675	0,213437	no
XLOC_007925	g9814	818,044	530,088	-0,625946	-0,954834	0,083	0,271381	no
XLOC_007926	g9815	0	0	0	0	1	1	no
XLOC_007927	g9816	278,761	258,983	-0,106169	-0,162661	0,773	0,8942	no
XLOC_007928	g9817	151,102	330,424	1,12879	1,69277	0,00355	0,031924	yes
XLOC_007929	g9818	0	0	0	0	1	1	no
XLOC_007930	g9819	20,05	29,1058	0,537701	0,624304	0,28275	0,545288	no
XLOC_007931	g9820	13,1595	22,7295	0,788458	1,13216	0,05285	0,204165	no
XLOC_007932	g9821	0,0141889	0,395068	4,79927	0	1	1	no
XLOC_007933	g9822	43,3895	48,4807	0,160065	0,252418	0,6645	0,835731	no
XLOC_007934	g9823	82,0108	156,325	0,930662	1,5239	0,0082	0,0580981	no
XLOC_007935	g3759	0,130415	0,305101	1,22617	0	1	1	no
XLOC_007936	g9824	5,32618	2,40897	-1,14468	-1,43926	0,0161	0,0928919	no
XLOC_007937	g9825	7,53423	5,30887	-0,505056	-0,497186	0,394	0,64863	no
XLOC_007938	g9826	0	0	0	0	1	1	no
XLOC_007939	g9827	0	0	0	0	1	1	no
XLOC_007940	g9828	11,7418	16,5229	0,492804	0,511139	0,3881	0,643448	no
XLOC_007941	g9829	0	0	0	0	1	1	no
XLOC_007942	g533	11,3493	6,13173	-0,888237	-1,01421	0,0756	0,254064	no
XLOC_007943	g536	26,0428	34,055	0,386983	0,577125	0,29485	0,556907	no
XLOC_007944	g537	16,2146	13,6436	-0,249067	-0,294294	0,59075	0,790268	no
XLOC_007945	g539	2210,4	1720,38	-0,361586	-0,552355	0,33745	0,597377	no
XLOC_007946	g542	119,227	209,43	0,812763	1,29538	0,0182	0,101432	no
XLOC_007947	g532	93,1112	80,7363	-0,205737	-0,337	0,5522	0,766299	no
XLOC_007948	g534	37,2061	31,4891	-0,240684	-0,323009	0,55835	0,7703	no
XLOC_007949	g535	263,289	236,723	-0,153452	-0,244815	0,6732	0,841348	no
XLOC_007950	g538	1762,05	2857,87	0,697687	1,06724	0,0581	0,215869	no
XLOC_007951	g540	53,2074	55,4199	0,0587772	0,0891529	0,87245	0,942536	no
XLOC_007952	g541	51,6139	65,4722	0,343121	0,579288	0,3125	0,573414	no
XLOC_007953	g9830	11,209	11,7728	0,0708017	0,0886671	0,87915	0,945715	no
XLOC_007954	g9831	47,4237	72,3879	0,61014	0,972175	0,0877	0,279888	no
XLOC_007955	g9832	138,366	231,943	0,745276	1,27247	0,0241	0,123907	no
XLOC_007956	g9833	6,32355	10,5404	0,737116	1,01672	0,0729	0,249343	no
XLOC_007957	g9835	6,7597	448,724	6,05273	7,32317	5,00E-05	0,00120049	yes
XLOC_007958	g9834	3,81738	6,07834	0,671095	0,61304	0,3013	0,563145	no
XLOC_007959	g9837	49,4602	24,8911	-0,990636	-1,68892	0,00285	0,0272512	yes
XLOC_007960	g9836	22,7295	25,7299	0,178877	0,27549	0,62875	0,814232	no
XLOC_007961	g9838	59,6279	67,2776	0,174139	0,300882	0,6045	0,79937	no
XLOC_007962	g3760	84,1305	70,6169	-0,252617	-0,429115	0,467	0,707046	no
XLOC_007963	g3762	4,49548	3,83273	-0,230102	-0,193985	0,7347	0,874834	no
XLOC_007964	g3765	133,586	101,442	-0,397114	-0,66686	0,23785	0,496002	no
XLOC_007965	g3761	36,6917	55,1171	0,587045	0,760789	0,19105	0,441334	no
XLOC_007966	g3763	0	0	0	0	1	1	no
XLOC_007967	g3764	72,246	84,0622	0,218539	0,362076	0,51375	0,740174	no
XLOC_007968	g3766	23,8856	14,5549	-0,714642	-0,713598	0,20895	0,464018	no
XLOC_007969	g9839	37,2695	28,2262	-0,400962	-0,616933	0,30165	0,563478	no
XLOC_007970	g9840	658,692	100,175	-2,71708	-3,84765	5,00E-05	0,00120049	yes
XLOC_007971	g9841	119,356	32,2069	-1,88983	-2,75602	5,00E-05	0,00120049	yes
XLOC_007972	g9843	39,7988	49,8474	0,324793	0,499495	0,383	0,639419	no
XLOC_007973	g9842	16,8027	17,9278	0,0935076	0,104889	0,8548	0,93471	no
XLOC_007974	g3767	7,6537	12,6747	0,72772	0,986426	0,07985	0,264372	no
XLOC_007975	g9844	18,7099	29,6507	0,664268	0,823063	0,142	0,376672	no
XLOC_007976	g9845	34,8507	29,4108	-0,244842	-0,399468	0,4921	0,723707	no
XLOC_007977	g9846	0	0	0	0	1	1	no
XLOC_007978	g9847	302,761	210,077	-0,527262	-0,648188	0,25575	0,515162	no
XLOC_007979	g9848	148,119	152,053	0,0378194	0,0551208	0,92355	0,966357	no
XLOC_007980	g9849	4,91673	5,30754	0,110346	0,138028	0,81515	0,915983	no
XLOC_007981	g9850	0	0	0	0	1	1	no
XLOC_007982	g9851	0,336014	6,02029	4,16324	2,80855	0,06125	0,223182	no
XLOC_007983	g9852	47,7307	29,6684	-0,685991	-1,10462	0,06135	0,223298	no
XLOC_007984	g9853	0	0	0	0	1	1	no
XLOC_007985	g3768	65,8603	49,1961	-0,420868	-0,664424	0,25875	0,518762	no
XLOC_007986	g3769	49,389	57,8587	0,228344	0,393306	0,48535	0,719022	no
XLOC_007987	g3770	6,17079	6,26945	0,0228848	0,0284326	0,95865	0,981744	no
XLOC_007988	g9854	19,8509	3,81969	-2,37768	-2,8374	5,00E-05	0,00120049	yes
XLOC_007989	g9855	0	0	0	0	1	1	no
XLOC_007990	g9856	0	0	0	0	1	1	no
XLOC_007991	g9857	0	0	0	0	1	1	no
XLOC_007992	g3771	99,0453	112,921	0,189148	0,314274	0,58285	0,784861	no
XLOC_007993	g3772	6,42704	13,5382	1,07481	1,50875	0,01045	0,068504	no
XLOC_007994	g3774	58,2485	44,913	-0,375088	-0,568546	0,3176	0,578204	no
XLOC_007995	g3776	243,359	185,373	-0,392655	-0,590613	0,30325	0,564534	no
XLOC_007996	g3777	32,3592	16,012	-1,01502	-1,53485	0,00675	0,0506379	no
XLOC_007997	g3773	70,7364	33,1154	-1,09495	-1,60235	0,0065	0,0492896	yes
XLOC_007998	g3775	5390,84	5584,98	0,0510424	0,0731316	0,8958	0,953681	no
XLOC_007999	g9858	0	0	0	0	1	1	no
XLOC_008000	g9859	84,2902	31,2996	-1,42922	-2,29954	5,00E-05	0,00120049	yes
XLOC_008001	g9860	10,6158	10,4765	-0,0190453	-0,0232255	0,9648	0,984142	no
XLOC_008002	g3779	39,561	30,882	-0,357314	-0,595549	0,28575	0,548274	no
XLOC_008003	g3781	32,6459	22,5482	-0,533891	-0,830426	0,1289	0,354665	no

XLOC_008004	g3778	358,618	794,745	1,14805	1,50288	0,0068	0,0508963	no
XLOC_008005	g3780	126,357	75,3189	-0,746417	-1,11673	0,0515	0,20021	no
XLOC_008006	g9861	59,6218	34,14	-0,804375	-1,10743	0,054	0,206576	no
XLOC_008007	g9862	132,934	78,1353	-0,766661	-0,929488	0,10505	0,313649	no
XLOC_008008	g9863	0	0	0	0	1	1	no
XLOC_008009	g9864	760,91	229,355	-1,73015	-2,8614	5,00E-05	0,00120049	yes
XLOC_008010	g9865	0,749067	0,922146	0,299901	0	1	1	no
XLOC_008011	g9866	75,0901	88,6315	0,239196	0,404863	0,4677	0,70724	no
XLOC_008012	g9867	0	0	0	0	1	1	no
XLOC_008013	g9868	23,6223	28,0708	0,248923	0,355339	0,52445	0,746391	no
XLOC_008014	g9869	404,467	325,475	-0,313472	-0,401259	0,48735	0,720794	no
XLOC_008015	g9870	0	0	0	0	1	1	no
XLOC_008016	g9871	40,2804	35,9357	-0,164661	-0,258622	0,63455	0,817196	no
XLOC_008017	g9872	111,591	89,0987	-0,324747	-0,450946	0,4304	0,678415	no
XLOC_008018	g9873	5,77339	11,4591	0,988999	1,39791	0,013	0,0805426	no
XLOC_008019	g9874	935,305	1645,49	0,81501	0,881127	0,12725	0,351999	no
XLOC_008020	g9875	234,772	309,93	0,400682	0,675181	0,2336	0,49121	no
XLOC_008021	g9876	23,4729	23,1563	-0,0195875	-0,0274047	0,9611	0,982511	no
XLOC_008022	g9877	37,3158	37,8879	0,0219496	0,0338965	0,9524	0,978815	no
XLOC_008023	g9878	0	0	0	0	1	1	no
XLOC_008024	g9879	50,7657	54,4477	0,101017	0,16348	0,7702	0,893264	no
XLOC_008025	g9880	12,7873	20,7241	0,696592	0,812613	0,16425	0,406405	no
XLOC_008026	g9881	45,5436	37,0232	-0,298817	-0,464731	0,4019	0,6557	no
XLOC_008027	g9882	7,08199	3,2966	-1,10318	-1,27827	0,0296	0,140761	no
XLOC_008028	g9883	20,4284	11,0981	-0,880266	-1,19259	0,0383	0,16554	no
XLOC_008029	g9884	0,237541	0	#NAME?	0	1	1	no
XLOC_008030	g9885	6,70488	7,64686	0,189657	0,238996	0,6727	0,841196	no
XLOC_008031	g9886	0	0	0	0	1	1	no
XLOC_008032	g9887	9,84094	9,8861	0,00660498	0,00959185	0,98475	0,992379	no
XLOC_008033	g9888	0,571712	0,369057	-0,631447	0	1	1	no
XLOC_008034	g3782	234,725	313,744	0,418615	0,713321	0,20715	0,461796	no
XLOC_008035	g9889	101,631	49,2073	-1,04639	-1,75678	0,0025	0,0247231	yes
XLOC_008036	g9890	321,306	479,5	0,577579	0,754373	0,17695	0,42392	no
XLOC_008037	g9891	71,4153	34,2078	-1,06191	-1,48865	0,0134	0,0820886	no
XLOC_008038	g9892	38,9662	34,6825	-0,168013	-0,276474	0,6138	0,805977	no
XLOC_008039	g9893	0	0	0	0	1	1	no
XLOC_008040	g9895	0	0	0	0	1	1	no
XLOC_008041	g9894	0	0	0	0	1	1	no
XLOC_008042	g9896	12,657	10,5123	-0,267857	-0,354403	0,5347	0,754094	no
XLOC_008043	g9897	0	0	0	0	1	1	no
XLOC_008044	g9898	0	0	0	0	1	1	no
XLOC_008045	g9899	8,11365	13,0327	0,683711	0,73046	0,20565	0,459914	no
XLOC_008046	g9900	24,0711	22,2502	-0,113484	-0,153276	0,7849	0,900961	no
XLOC_008047	g9901	4,76947	6,75417	0,501951	0,613379	0,2882	0,551681	no
XLOC_008048	g9902	13,3773	13,3759	-0,000148104	-0,000196639	0,99185	0,995293	no
XLOC_008049	g9903	110,76	122,219	0,142029	0,242239	0,66945	0,839055	no
XLOC_008050	g9904	101,455	502,079	2,30707	3,18727	5,00E-05	0,00120049	yes
XLOC_008051	g9905	0	0	0	0	1	1	no
XLOC_008052	g9906	29,5245	17,5097	-0,753755	-1,05358	0,0691	0,240967	no
XLOC_008053	g9907	31,9973	41,4485	0,37337	0,575699	0,30485	0,566474	no
XLOC_008054	g3783	16,1718	9,71491	-0,735212	-0,568753	0,33705	0,597208	no
XLOC_008055	g9908	11,8714	11,1974	-0,0843235	-0,107775	0,8491	0,931846	no
XLOC_008056	g3784	76,8198	72,8184	-0,077176	-0,131837	0,8163	0,916541	no
XLOC_008057	g3785	72,6147	84,759	0,223106	0,379884	0,50815	0,734874	no
XLOC_008058	g9909	17,9428	12,9989	-0,465008	-0,58676	0,2967	0,558695	no
XLOC_008059	g9910	29,8375	19,3814	-0,62245	-1,04732	0,06815	0,238841	no
XLOC_008060	g9911	5,2061	7,71039	0,566601	0,647519	0,2694	0,5308	no
XLOC_008061	g9912	0	0	0	0	1	1	no
XLOC_008062	g9913	39,5086	40,5256	0,0366675	0,0575942	0,92115	0,96508	no
XLOC_008063	g3787	278,017	377,134	0,439905	0,667288	0,2325	0,490367	no
XLOC_008064	g3786	506,52	369,748	-0,454076	-0,741619	0,19555	0,446997	no
XLOC_008065	g3788	49,1306	32,1918	-0,609932	-0,96482	0,0885	0,281526	no
XLOC_008066	g3789	10,167	10,6224	0,0632175	0,0813744	0,8849	0,94862	no
XLOC_008067	g9914	1,83083	1,9762	0,110232	0,0853574	0,87795	0,945394	no
XLOC_008068	g3790	0	0	0	0	1	1	no
XLOC_008069	g9915	24,7527	29,1491	0,235864	0,351352	0,5198	0,743978	no
XLOC_008070	g9916	14,6811	20,237	0,463038	0,572959	0,31625	0,577009	no
XLOC_008071	g9917	0	0	0	0	1	1	no
XLOC_008072	g9918	0	0	0	0	1	1	no
XLOC_008073	g3792	34,1413	18,6929	-0,869026	-1,36325	0,01425	0,085692	no
XLOC_008074	g3791	72,6636	70,0204	-0,0534592	-0,0863043	0,87165	0,942376	no
XLOC_008075	g3793	0	0,59228	inf	0	1	1	no
XLOC_008076	g3794	8,5481	7,41089	-0,205958	-0,29427	0,5915	0,790276	no
XLOC_008077	g3795	202,297	302,305	0,579534	0,931323	0,1076	0,319224	no
XLOC_008078	g9919	0	0	0	0	1	1	no
XLOC_008079	g3797	0	0	0	0	1	1	no
XLOC_008080	g3796	0,578724	0,372596	-0,635264	0	1	1	no
XLOC_008081	g3798	0,843285	1,88319	1,15909	1,21228	0,0387	0,166386	no
XLOC_008082	g3799	3,09555	1,97506	-0,648299	-0,764756	0,17945	0,426373	no
XLOC_008083	g9920	12,3164	10,8233	-0,186437	-0,283804	0,6231	0,810872	no
XLOC_008084	g9921	32,3492	28,5792	-0,178763	-0,284555	0,61825	0,808291	no
XLOC_008085	g9922	4,33759	6,07127	0,485104	0,477292	0,42395	0,673219	no
XLOC_008086	g9923	121,629	119,998	-0,0194708	-0,0323835	0,95575	0,980512	no
XLOC_008087	g9926	14,5703	12,6255	-0,206698	-0,318449	0,57005	0,777701	no
XLOC_008088	g9924	1571,08	1037,81	-0,598209	-0,882016	0,127	0,351803	no
XLOC_008089	g9925	28,7991	18,9776	-0,601723	-0,92243	0,1012	0,30578	no
XLOC_008090	g9927	64,656	66,9164	0,049575	0,0855208	0,8772	0,944943	no
XLOC_008091	g3800	14,2067	15,5913	0,134178	0,191456	0,7383	0,876358	no
XLOC_008092	g3801	48,9526	86,9439	0,828701	1,06834	0,0598	0,219875	no
XLOC_008093	g9928	54,9378	32,9049	-0,739495	-0,865287	0,1301	0,357303	no
XLOC_008094	g9929	174,042	132,307	-0,395554	-0,686471	0,24585	0,504545	no
XLOC_008095	g9930	0	0	0	0	1	1	no

XLOC_008096	g3802	48,2208	49,7695	0,0456077	0,0780124	0,89225	0,951863	no
XLOC_008097	g3805	362,855	514	0,502377	0,671397	0,2512	0,510076	no
XLOC_008098	g3807	60,8935	64,9201	0,0923765	0,142532	0,7997	0,907868	no
XLOC_008099	g3803	143,967	60,6315	-1,24759	-2,07427	0,0004	0,0061761	yes
XLOC_008100	g3804	177,656	222,421	0,324208	0,560325	0,3292	0,590348	no
XLOC_008101	g3806	7,24981	9,81382	0,436871	0,633829	0,2628	0,523149	no
XLOC_008102	g3808	0	0	0	0	1	1	no
XLOC_008103	g9931	222,645	228,673	0,0385435	0,0561286	0,92075	0,96497	no
XLOC_008104	g3809	103,59	119,089	0,201149	0,326881	0,5615	0,77218	no
XLOC_008105	g9932	0	0	0	0	1	1	no
XLOC_008106	g9934	220,255	160,35	-0,457951	-0,765685	0,1796	0,426523	no
XLOC_008107	g9935	250,26	148,794	-0,750106	-1,20943	0,0323	0,148218	no
XLOC_008108	g9936	29,2938	46,3263	0,661235	1,10969	0,0497	0,195927	no
XLOC_008109	g9938	675,443	563,646	-0,261043	-0,431281	0,45815	0,701013	no
XLOC_008110	g9940	354,549	540,06	0,607136	1,04231	0,0664	0,234381	no
XLOC_008111	g9943	105,338	151,075	0,52024	0,842639	0,1521	0,389904	no
XLOC_008112	g9933	224,632	173,676	-0,371161	-0,637114	0,25915	0,51904	no
XLOC_008113	g9937	63,9335	67,0583	0,0688426	0,116288	0,8383	0,926315	no
XLOC_008114	g9939	31,2867	35,1446	0,167753	0,28812	0,61115	0,804448	no
XLOC_008115	g9941	28,8527	31,0279	0,104856	0,176856	0,7514	0,883013	no
XLOC_008116	g9942	3448,68	5035,22	0,546011	0,751142	0,1848	0,433318	no
XLOC_008117	g9944	58,8617	81,8449	0,475563	0,763561	0,1852	0,433572	no
XLOC_008118	g9945	36,8524	30,112	-0,291417	-0,453096	0,4254	0,674758	no
XLOC_008119	g3812	8,25289	9,50259	0,203421	0,288728	0,61315	0,805503	no
XLOC_008120	g3810	15,8377	18,9768	0,260879	0,387635	0,4919	0,723707	no
XLOC_008121	g3811	17,5012	15,326	-0,191475	-0,285507	0,62475	0,811943	no
XLOC_008122	g9946	39,2242	42,3291	0,109903	0,157942	0,7776	0,895827	no
XLOC_008123	g9947	679,315	370,347	-0,875202	-1,08428	0,0708	0,244636	no
XLOC_008124	g9949	101,517	84,8918	-0,258024	-0,407357	0,4648	0,705296	no
XLOC_008125	g9948	130,686	205,493	0,652979	0,96544	0,08415	0,273627	no
XLOC_008126	g9950	54,6094	92,0214	0,752821	1,25119	0,02845	0,13722	no
XLOC_008127	g9951	111,202	96,1708	-0,209515	-0,361702	0,5326	0,752862	no
XLOC_008128	g9952	1688,13	689,14	-1,29256	-1,7855	0,0037	0,032971	yes
XLOC_008129	g9953	25,0176	50,1671	1,0038	1,60702	0,0048	0,0398445	yes
XLOC_008130	g9954	30,1034	28,5309	-0,0774025	-0,1096	0,8441	0,929163	no
XLOC_008131	g3813	0	0	0	0	1	1	no
XLOC_008132	g9955	6,60915	0	#NAME?	0	1	1	no
XLOC_008133	g3816	112,806	9,21041	-3,61443	-4,51743	5,00E-05	0,00120049	yes
XLOC_008134	g3814	34,3462	14,1255	-1,28185	-1,959	0,0008	0,0106019	yes
XLOC_008135	g3815	7,8388	3,61094	-1,11826	-0,962482	0,10465	0,312835	no
XLOC_008136	g9956	0	0	0	0	1	1	no
XLOC_008137	g9958	2,01111	16,8931	3,07037	2,71352	0,00025	0,00430702	yes
XLOC_008138	g9959	53,6747	80,9768	0,593268	0,981972	0,08685	0,278442	no
XLOC_008139	g9957	8,94556	7,06545	-0,34039	-0,437148	0,45585	0,700539	no
XLOC_008140	g9960	0	0	0	0	1	1	no
XLOC_008141	g9961	0	0	0	0	1	1	no
XLOC_008142	g9962	0	0	0	0	1	1	no
XLOC_008143	g3817	99,2656	52,2314	-0,926376	-1,55813	0,0075	0,0546771	no
XLOC_008144	g3818	18,837	26,4076	0,487383	0,685771	0,23425	0,491629	no
XLOC_008145	g3819	2,29602	1,26701	-0,857701	-0,636299	0,3416	0,601706	no
XLOC_008146	g3820	0	0	0	0	1	1	no
XLOC_008147	g9963	0	0	0	0	1	1	no
XLOC_008148	g3821	48,4839	37,1929	-0,382477	-0,505287	0,378	0,636045	no
XLOC_008149	g9964	4,33124	4,48316	0,0497358	0,0623311	0,91265	0,962645	no
XLOC_008150	g9965	2,83425	2,6619	-0,0905081	-0,095895	0,8719	0,942536	no
XLOC_008151	g9966	142,014	132,464	-0,100434	-0,165857	0,77495	0,894769	no
XLOC_008152	g9967	0	0	0	0	1	1	no
XLOC_008153	g9968	8,01266	10,6668	0,41278	0,581216	0,3098	0,570883	no
XLOC_008154	g9969	400,746	1100,73	1,4577	2,04314	0,0005	0,00733931	yes
XLOC_008155	g9970	39,0704	52,6429	0,430162	0,714742	0,2225	0,479892	no
XLOC_008156	g9971	30,329	34,8259	0,199461	0,322511	0,557	0,770021	no
XLOC_008157	g9972	0	0	0	0	1	1	no
XLOC_008158	g544	48,0467	49,7396	0,049957	0,0798804	0,89085	0,951299	no
XLOC_008159	g546	83,5174	106,026	0,344276	0,595073	0,29195	0,554536	no
XLOC_008160	g548	16,8009	33,3143	0,987605	1,58314	0,0061	0,0471669	yes
XLOC_008161	g549	105,546	107,292	0,0236703	0,037677	0,94935	0,977163	no
XLOC_008162	g543	159,599	105,44	-0,598038	-0,948619	0,09405	0,29264	no
XLOC_008163	g545	32,2536	20,3349	-0,665501	-1,01898	0,0699	0,242551	no
XLOC_008164	g547	70,5169	97,0914	0,461373	0,783152	0,17315	0,418595	no
XLOC_008165	g9973	143,694	113,909	-0,335111	-0,517835	0,35525	0,615917	no
XLOC_008166	g9974	7,26399	16,6983	1,20087	1,48837	0,00965	0,0647288	no
XLOC_008167	g9975	84,2895	73,9361	-0,189073	-0,298193	0,5946	0,792155	no
XLOC_008168	g3822	19,8273	11,3263	-0,807818	-1,30697	0,02315	0,120601	no
XLOC_008169	g3824	119,443	129,727	0,119156	0,204726	0,71575	0,865386	no
XLOC_008170	g3825	40,0716	18,528	-1,11287	-1,72549	0,00385	0,0340297	yes
XLOC_008171	g3823	138,803	96,5012	-0,52442	-0,868265	0,121	0,342921	no
XLOC_008172	g3826	16,3103	31,0155	0,927206	1,4935	0,01015	0,0673011	no
XLOC_008173	g9976	0	0	0	0	1	1	no
XLOC_008174	g3827	0,69081	0,738356	0,0960269	0	1	1	no
XLOC_008175	g3828	41,0815	19,1911	-1,09805	-1,60139	0,00705	0,0520143	no
XLOC_008176	g3829	71,1779	67,6254	-0,0738647	-0,119627	0,8257	0,920778	no
XLOC_008177	g3830	53,1006	71,8095	0,435446	0,718302	0,2142	0,469524	no
XLOC_008178	g9977	6,2216	9,59813	0,625468	0,734535	0,21035	0,465443	no
XLOC_008179	g3831	0,430315	0,37578	-0,195502	0	1	1	no
XLOC_008180	g3832	59,2067	75,7733	0,355929	0,598251	0,2998	0,562053	no
XLOC_008181	g9979	0,943112	2,11025	1,16191	0,846694	0,14985	0,387142	no
XLOC_008182	g9978	3,26484	3,17305	-0,0411449	-0,0508454	0,92835	0,968234	no
XLOC_008183	g3833	0	0	0	0	1	1	no
XLOC_008184	g9980	0,237176	0,868705	1,87291	0	1	1	no
XLOC_008185	g9981	0	0	0	0	1	1	no
XLOC_008186	g3834	305,273	364,62	0,256294	0,378929	0,50955	0,73585	no
XLOC_008187	g3836	3,47165	0,765208	-2,1817	-2,09042	0,00365	0,0326439	yes

XLOC_008188	g3837	2,59096	0,752892	-1,78297	-1,48567	0,0584	0,216329	no
XLOC_008189	g3835	3,5299	4,61857	0,387818	0,396973	0,4981	0,727878	no
XLOC_008190	g9982	0	0,598244	inf	0	1	1	no
XLOC_008191	g3838	0	0	0	0	1	1	no
XLOC_008192	g3839	25,9665	49,4261	0,928621	1,46474	0,0098	0,0655112	no
XLOC_008193	g9983	0	0	0	0	1	1	no
XLOC_008194	g3840	82,5928	98,8217	0,258813	0,409992	0,4636	0,704839	no
XLOC_008195	g3841	17,064	13,7319	-0,313422	-0,366389	0,519	0,743592	no
XLOC_008196	g3842	28,787	38,0646	0,403031	0,627303	0,2714	0,532816	no
XLOC_008197	g3843	0	0	0	0	1	1	no
XLOC_008198	g550	0	0	0	0	1	1	no
XLOC_008199	g3844	32,6847	19,022	-0,780947	-1,23901	0,03035	0,142533	no
XLOC_008200	g3845	5,79607	22,2626	1,94148	2,04691	0,0011	0,0134856	yes
XLOC_008201	g9984	36,7234	52,2331	0,508265	0,855531	0,13125	0,359318	no
XLOC_008202	g9985	24,0642	23,8889	-0,0105435	-0,0117463	0,98775	0,993619	no
XLOC_008203	g3846	0	0	0	0	1	1	no
XLOC_008204	g9986	0	0	0	0	1	1	no
XLOC_008205	g9987	0	0	0	0	1	1	no
XLOC_008206	g9988	3,48741	2,01433	-0,791855	-0,75823	0,27455	0,536213	no
XLOC_008207	g9989	14,5963	16,5797	0,183821	0,267986	0,63925	0,820902	no
XLOC_008208	g9990	0	0	0	0	1	1	no
XLOC_008209	g9991	4,26809	4,40261	0,0447667	0,0309778	0,93155	0,970076	no
XLOC_008210	g9992	0	0	0	0	1	1	no
XLOC_008211	g3848	76,4989	81,5623	0,0924636	0,14288	0,80555	0,910614	no
XLOC_008212	g3847	11,9836	15,7815	0,397173	0,57843	0,30715	0,568132	no
XLOC_008213	g3849	0	0	0	0	1	1	no
XLOC_008214	g3850	0	0	0	0	1	1	no
XLOC_008215	g3851	0	0	0	0	1	1	no
XLOC_008216	g9993	25,5561	36,9749	0,532877	0,785569	0,1793	0,426373	no
XLOC_008217	g3852	516,033	649,724	0,332364	0,431347	0,4591	0,701036	no
XLOC_008218	g3853	75,932	76,637	0,0133327	0,0225286	0,9689	0,98653	no
XLOC_008219	g3854	8,1133	8,88269	0,130708	0,152918	0,78625	0,901303	no
XLOC_008220	g9994	4,29838	7,79914	0,859521	1,09998	0,06455	0,230502	no
XLOC_008221	g9995	19,5401	27,3515	0,485179	0,705468	0,2196	0,477096	no
XLOC_008222	g9996	26,6952	39,3837	0,561018	0,882463	0,12505	0,348564	no
XLOC_008223	g9998	75,2998	506,015	2,74846	3,39051	5,00E-05	0,00120049	yes
XLOC_008224	g9999	69,7366	61,2553	-0,187081	-0,309724	0,5735	0,779376	no
XLOC_008225	g9997	36,2834	38,3052	0,0782305	0,122717	0,8285	0,921598	no
XLOC_008226	g10000	14,0264	25,8987	0,884736	1,36947	0,0174	0,0980873	no
XLOC_008227	g10001	0,125282	0,228988	0,870096	0	1	1	no
XLOC_008228	g3855	0	0	0	0	1	1	no
XLOC_008229	g10002	415,321	362,099	-0,197843	-0,322809	0,59265	0,791274	no
XLOC_008230	g3856	63,8745	59,8096	-0,094861	-0,155522	0,7851	0,901085	no
XLOC_008231	g3857	88,6549	95,4701	0,10685	0,161747	0,76095	0,887368	no
XLOC_008232	g10003	7,18772	8,20853	0,191591	0,194833	0,7435	0,879024	no
XLOC_008233	g10004	106,05	191,824	0,855037	1,24932	0,03395	0,153141	no
XLOC_008234	g3858	4,52955	9,04368	0,997542	0,857905	0,1379	0,370298	no
XLOC_008235	g10006	71,3422	50,5131	-0,4981	-0,846786	0,1326	0,362005	no
XLOC_008236	g10005	8,96059	11,7213	0,387468	0,561028	0,32335	0,583909	no
XLOC_008237	g3859	4,69335	0	#NAME?	0	1	1	no
XLOC_008238	g3860	14,886	6,62924	-1,16704	-1,83809	0,00095	0,0120686	yes
XLOC_008239	g3861	4,89388	12,9708	1,40622	1,10589	0,0604	0,221069	no
XLOC_008240	g3862	8,89753	7,3908	-0,267675	-0,315188	0,58015	0,783024	no
XLOC_008241	g3864	30,5376	9,72499	-1,65082	-2,3782	0,0001	0,00209829	yes
XLOC_008242	g3863	4,8917	10,2418	1,06606	1,35428	0,0186	0,103252	no
XLOC_008243	g10007	0	0	0	0	1	1	no
XLOC_008244	g10008	10,3987	11,148	0,100381	0,131583	0,81765	0,917113	no
XLOC_008245	g10009	0	0	0	0	1	1	no
XLOC_008246	g3865	14,0362	17,6656	0,331789	0,412551	0,45685	0,701012	no
XLOC_008247	g3867	79,1248	66,8859	-0,242427	-0,415285	0,4703	0,708118	no
XLOC_008248	g3869	3,07516	4,78915	0,639107	0,834423	0,14875	0,386027	no
XLOC_008249	g3871	6,76584	15,0704	1,15537	1,7232	0,00245	0,0243512	yes
XLOC_008250	g3866	176,516	303,603	0,782386	1,25449	0,0295	0,140422	no
XLOC_008251	g3868	26,9074	31,8854	0,24489	0,385189	0,48545	0,719022	no
XLOC_008252	g3870	0,343539	1,22577	1,83514	1,23453	0,04965	0,195808	no
XLOC_008253	g3872	406,058	183,589	-1,14521	-1,9242	0,0008	0,0106019	yes
XLOC_008254	g10010	8,04468	6,86775	-0,228198	-0,285349	0,6124	0,805014	no
XLOC_008255	g10011	0	0	0	0	1	1	no
XLOC_008256	g10012	857,285	200,489	-2,09625	-2,145	0,0002	0,00367103	yes
XLOC_008257	g10013	14,0525	13,9579	-0,00974403	-0,0150881	0,9791	0,990192	no
XLOC_008258	g10014	0	0	0	0	1	1	no
XLOC_008259	g3873	23,3801	41,3228	0,821661	1,17442	0,03435	0,154026	no
XLOC_008260	g3874	30,2607	50,6556	0,743279	1,19942	0,04085	0,172008	no
XLOC_008261	g3876	17,6031	16,005	-0,137304	-0,196688	0,72405	0,86956	no
XLOC_008262	g3875	214,715	101,849	-1,076	-1,80165	0,0017	0,0185283	yes
XLOC_008263	g3877	3,58678	6,06511	0,757845	0,812319	0,1621	0,403833	no
XLOC_008264	g10015	0	0	0	0	1	1	no
XLOC_008265	g3878	0	0	0	0	1	1	no
XLOC_008266	g3879	142,691	152,447	0,095418	0,16091	0,77705	0,895827	no
XLOC_008267	g3880	41,3567	44,2116	0,0963056	0,148705	0,78685	0,901522	no
XLOC_008268	g10016	205,15	162,452	-0,336662	-0,557998	0,3135	0,574146	no
XLOC_008269	g10017	70,6526	108,784	0,622658	1,0014	0,08765	0,279888	no
XLOC_008270	g10018	45,547	23,0175	-0,984627	-1,56934	0,00605	0,0468911	yes
XLOC_008271	g10019	5,13363	10,3206	1,00748	1,14491	0,05415	0,207069	no
XLOC_008272	g3881	69,8004	94,985	0,444465	0,752326	0,18775	0,436587	no
XLOC_008273	g10020	214,599	328,785	0,615504	0,983843	0,07585	0,254649	no
XLOC_008274	g10021	12,9884	5,29395	-1,2948	-1,80109	0,0031	0,0290477	yes
XLOC_008275	g3882	26,3956	28,2831	0,0996448	0,162002	0,77275	0,894131	no
XLOC_008276	g3884	12,4743	2,66806	-2,22509	-2,80204	5,00E-05	0,00120049	yes
XLOC_008277	g3887	52,0347	47,3696	-0,135514	-0,223265	0,68145	0,846595	no
XLOC_008278	g3888	7,39475	9,39157	0,344864	0,313271	0,5736	0,779404	no
XLOC_008279	g3883	2,80401	2,47049	-0,182694	-0,210114	0,7198	0,867399	no

XLOC_008280	g3885	2,8404	1,03775	-1,45263	-1,58422	0,00925	0,0629487	no
XLOC_008281	g3886	6,18518	10,8234	0,807263	0,867916	0,14095	0,374697	no
XLOC_008282	g10022	0	0	0	0	1	1	no
XLOC_008283	g10023	0	0	0	0	1	1	no
XLOC_008284	g10024	5,72882	9,3266	0,703114	0,751036	0,18165	0,429315	no
XLOC_008285	g10027	901,072	1503,71	0,738809	0,749184	0,18605	0,434072	no
XLOC_008286	g10029	4,34268	4,85425	0,160662	0,213426	0,71435	0,865069	no
XLOC_008287	g10025	76,5633	48,0284	-0,672765	-1,09338	0,05605	0,211697	no
XLOC_008288	g10026	40,7861	62,35	0,612312	0,931151	0,10065	0,304399	no
XLOC_008289	g10028	27,3866	27,2028	-0,00971308	-0,0157752	0,9787	0,989991	no
XLOC_008290	g10031	25,8259	20,9102	-0,304614	-0,474953	0,39475	0,648995	no
XLOC_008291	g10030	170,921	202,154	0,242125	0,338423	0,56475	0,774343	no
XLOC_008292	g3889	222,73	228,412	0,0363399	0,0609985	0,91095	0,962529	no
XLOC_008293	g3890	61,7451	127,044	1,04093	1,71052	0,00295	0,0280164	yes
XLOC_008294	g3892	54,8361	51,3834	-0,0938235	-0,163328	0,77345	0,894251	no
XLOC_008295	g3891	7,67634	13,1476	0,776311	1,20253	0,03395	0,153141	no
XLOC_008296	g3893	189,653	119,989	-0,660458	-1,08369	0,06095	0,222254	no
XLOC_008297	g3894	12,1176	19,0451	0,652319	0,546206	0,33435	0,594729	no
XLOC_008298	g10032	3,35367	6,11508	0,866631	0,77153	0,307	0,568054	no
XLOC_008299	g3895	57,4155	55,0727	-0,0601026	-0,0919792	0,86855	0,940163	no
XLOC_008300	g3896	18,2756	33,7727	0,885935	1,30362	0,02825	0,136523	no
XLOC_008301	g10033	4,25472	3,47365	-0,292612	-0,408689	0,4789	0,714385	no
XLOC_008302	g10034	55,2657	113,37	1,03658	1,38839	0,0137	0,0833544	no
XLOC_008303	g551	13,9914	9,94438	-0,49259	-0,653297	0,2667	0,527598	no
XLOC_008304	g552	7,44771	14,2229	0,933352	1,19961	0,03705	0,161847	no
XLOC_008305	g10035	7,35835	5,50416	-0,41886	-0,531331	0,35875	0,619938	no
XLOC_008306	g10036	1,37184	1,43396	0,0638874	0	1	1	no
XLOC_008307	g10037	35,9708	65,2653	0,859491	1,31922	0,0228	0,119539	no
XLOC_008308	g3897	0	0	0	0	1	1	no
XLOC_008309	g554	132,508	130,721	-0,0195822	-0,0326542	0,95665	0,980821	no
XLOC_008310	g556	44,3164	59,9236	0,435284	0,668448	0,242	0,500303	no
XLOC_008311	g558	266,85	168,041	-0,667214	-1,11597	0,0504	0,197813	no
XLOC_008312	g559	163,709	164,727	0,00894645	0,0139816	0,9801	0,990233	no
XLOC_008313	g561	95,1218	74,472	-0,353077	-0,546626	0,34125	0,601413	no
XLOC_008314	g562	11,7779	11,4806	-0,0368755	-0,0599354	0,91455	0,963407	no
XLOC_008315	g563	55,5098	35,4343	-0,647599	-1,07609	0,06475	0,230629	no
XLOC_008316	g564	3,72401	3,17858	-0,228474	-0,317012	0,55735	0,770071	no
XLOC_008317	g566	30,1256	21,7972	-0,466846	-0,693405	0,23535	0,49341	no
XLOC_008318	g569	46,928	80,8252	0,784357	1,33135	0,02155	0,115074	no
XLOC_008319	g570	388,695	384,33	-0,0162942	-0,0252162	0,9648	0,984142	no
XLOC_008320	g571	34,5263	33,5261	-0,0424118	-0,0652522	0,90545	0,958604	no
XLOC_008321	g572	27,0161	30,3149	0,166208	0,28004	0,62115	0,809622	no
XLOC_008322	g573	89,3703	66,0539	-0,436153	-0,740528	0,18525	0,433572	no
XLOC_008323	g575	40,2063	44,5058	0,146572	0,250785	0,6585	0,83245	no
XLOC_008324	g576	82,6869	56,2046	-0,556971	-0,959183	0,08865	0,281729	no
XLOC_008325	g578	11,8392	9,62579	-0,298589	-0,476643	0,39695	0,651194	no
XLOC_008326	g582	6,55325	13,4811	1,04066	1,35372	0,022	0,116526	no
XLOC_008327	g585	48,243	66,1035	0,454408	0,782228	0,1828	0,429978	no
XLOC_008328	g586	147,536	131,353	-0,167615	-0,289739	0,6152	0,806793	no
XLOC_008329	g588	110,969	94,2245	-0,235984	-0,409578	0,4789	0,714385	no
XLOC_008330	g553	155,088	103,087	-0,589229	-0,831536	0,15365	0,391806	no
XLOC_008331	g555	32,0991	37,3762	0,219588	0,367731	0,52415	0,746323	no
XLOC_008332	g557	61,0834	55,0404	-0,150288	-0,255322	0,6438	0,824265	no
XLOC_008333	g560	65,7006	62,5896	-0,0699832	-0,120654	0,8367	0,925387	no
XLOC_008334	g565	63,0377	78,3237	0,313233	0,516065	0,3548	0,615463	no
XLOC_008335	g567	36,1541	25,7563	-0,489235	-0,813127	0,16185	0,403598	no
XLOC_008336	g568	1556,3	2342,75	0,590083	0,779861	0,15215	0,389904	no
XLOC_008337	g574	12,8712	20,8659	0,696997	1,04095	0,0683	0,238941	no
XLOC_008338	g577	45,1145	42,6648	-0,0805435	-0,138581	0,8005	0,908356	no
XLOC_008339	g579	75,3451	54,184	-0,475647	-0,712901	0,21295	0,468347	no
XLOC_008340	g580	171,719	206,529	0,266289	0,459913	0,4185	0,670062	no
XLOC_008341	g581	33,0811	31,9641	-0,049553	-0,0820609	0,88445	0,948388	no
XLOC_008342	g583	67,4528	44,0205	-0,615705	-0,991446	0,0877	0,279888	no
XLOC_008343	g584	54,3524	53,8596	-0,0131391	-0,0209777	0,9715	0,986977	no
XLOC_008344	g587	1234,86	1755,85	0,507815	0,784004	0,16215	0,403833	no
XLOC_008345	g589	2003	2084,97	0,0578623	0,0792115	0,89065	0,951189	no
XLOC_008346	g3899	28,1846	14,3135	-0,977528	-1,47629	0,0117	0,0743169	no
XLOC_008347	g3901	49,75	54,275	0,12559	0,199656	0,72765	0,870385	no
XLOC_008348	g3898	1,20641	0,247919	-2,28278	-1,13273	0,2673	0,528252	no
XLOC_008349	g3900	8,78006	7,07986	-0,310511	-0,322808	0,58285	0,784861	no
XLOC_008350	g10038	0,172758	0,159677	-0,113593	0	1	1	no
XLOC_008351	g10039	10,5266	44,2363	2,0712	2,39543	0,00045	0,00669545	yes
XLOC_008352	g10040	8,43575	4,12399	-1,03248	-1,12208	0,15555	0,392876	no
XLOC_008353	g10041	0	0	0	0	1	1	no
XLOC_008354	g10042	26,6356	131,052	2,29871	3,68369	5,00E-05	0,00120049	yes
XLOC_008355	g10043	23,654	109,687	2,21324	3,44542	5,00E-05	0,00120049	yes
XLOC_008356	g10044	35,8026	57,2597	0,677456	1,06538	0,065	0,231101	no
XLOC_008357	g10045	14,5098	15,0187	0,0497345	0,0837834	0,8856	0,948788	no
XLOC_008358	g10046	5,88398	18,9619	1,68824	2,17859	0,0001	0,00209829	yes
XLOC_008359	g10047	1,72232	1,17462	-0,552163	-0,588058	0,313	0,573658	no
XLOC_008360	g10048	0	0	0	0	1	1	no
XLOC_008361	g10049	657,148	559,891	-0,231072	-0,385012	0,49515	0,725692	no
XLOC_008362	g3902	190,906	465,196	1,28498	1,53299	0,009	0,0618908	no
XLOC_008363	g3904	155,378	166,685	0,10135	0,17227	0,7659	0,890392	no
XLOC_008364	g3903	1166,89	2516,46	1,10873	1,68996	0,0027	0,0261737	yes
XLOC_008365	g3905	7,6848	13,1618	0,77628	0,924795	0,1072	0,318422	no
XLOC_008366	g3906	15,9267	5,81228	-1,45427	-2,18156	0,0002	0,00367103	yes
XLOC_008367	g10050	66,3531	32,2727	-1,03985	-1,35263	0,0203	0,110075	no
XLOC_008368	g10051	2,36648	2,14735	-0,140187	-0,10237	0,86005	0,935634	no
XLOC_008369	g3907	87,6669	69,639	-0,332138	-0,541459	0,33085	0,591578	no
XLOC_008370	g3908	5,71074	15,5975	1,44956	1,88053	0,002	0,0208936	yes
XLOC_008371	g10052	60,6401	20,6369	-1,55504	-2,41229	5,00E-05	0,00120049	yes

XLOC_008372	g591	35,5646	42,165	0,245604	0,323515	0,57655	0,780604	no
XLOC_008373	g590	2,18191	1,60934	-0,439125	-0,543141	0,3316	0,592577	no
XLOC_008374	g10053	30,1514	16,635	-0,858	-1,22573	0,03025	0,142404	no
XLOC_008375	g10054	119,883	99,7363	-0,265439	-0,444334	0,42215	0,672427	no
XLOC_008376	g10055	9,25771	6,62226	-0,483331	-0,476445	0,41635	0,668393	no
XLOC_008377	g10056	0	0	0	0	1	1	no
XLOC_008378	g10057	0	0	0	0	1	1	no
XLOC_008379	g10058	0	0	0	0	1	1	no
XLOC_008380	g10059	87,9984	178,586	1,02107	1,64787	0,00515	0,0418998	yes
XLOC_008381	g10061	26,4492	16,1097	-0,715292	-1,14627	0,04035	0,170865	no
XLOC_008382	g10060	149,489	87,7928	-0,767867	-1,24679	0,0307	0,143354	no
XLOC_008383	g3909	23,9801	22,3878	-0,0991276	-0,159434	0,7789	0,896904	no
XLOC_008384	g3910	221,701	141,726	-0,645509	-0,74595	0,211	0,466356	no
XLOC_008385	g10062	47,0556	168,321	1,83877	1,92911	0,0039	0,0342252	yes
XLOC_008386	g3912	26,644	14,9448	-0,834166	-1,22838	0,03205	0,147346	no
XLOC_008387	g3913	13,0734	10,101	-0,372141	-0,53964	0,34375	0,603977	no
XLOC_008388	g3914	172,363	146,067	-0,238824	-0,423147	0,4702	0,708076	no
XLOC_008389	g3916	58,7412	37,3529	-0,653154	-1,06498	0,0593	0,218591	no
XLOC_008390	g3917	340,47	890,379	1,38689	1,75747	0,00235	0,0236687	yes
XLOC_008391	g3911	62,2828	45,4056	-0,455963	-0,783703	0,1675	0,410802	no
XLOC_008392	g3915	71,4731	68,7534	-0,0559691	-0,0960396	0,8572	0,935212	no
XLOC_008393	g10063	18,8325	15,5122	-0,279822	-0,408442	0,472	0,70959	no
XLOC_008394	g3918	138,279	181,929	0,395789	0,656515	0,24365	0,502445	no
XLOC_008395	g3919	62,7488	100,082	0,673525	1,05071	0,0555	0,210104	no
XLOC_008396	g3920	18,2378	12,6063	-0,532785	-0,899384	0,1125	0,327916	no
XLOC_008397	g3921	97,2803	69,422	-0,486755	-0,801716	0,155	0,392851	no
XLOC_008398	g10064	3,83289	4,35613	0,184613	0,205538	0,72405	0,86956	no
XLOC_008399	g10065	4,72502	6,13114	0,375834	0,463599	0,4102	0,662854	no
XLOC_008400	g10066	46,9422	39,0711	-0,264782	-0,371956	0,52065	0,744327	no
XLOC_008401	g10067	94,2753	75,9392	-0,312035	-0,523485	0,3611	0,621451	no
XLOC_008402	g10068	39,7995	46,4795	0,223843	0,383193	0,49585	0,725995	no
XLOC_008403	g10069	0	0	0	0	1	1	no
XLOC_008404	g10070	77,0576	88,1155	0,193458	0,314963	0,5716	0,77837	no
XLOC_008405	g10071	45,9691	86,8914	0,918548	1,03177	0,08515	0,275601	no
XLOC_008406	g10072	713,34	849,043	0,251247	0,353391	0,5229	0,745661	no
XLOC_008407	g10073	144,052	198,347	0,461433	0,614054	0,2975	0,559437	no
XLOC_008408	g10074	0	0	0	0	1	1	no
XLOC_008409	g10075	2,67476	6,40337	1,25942	1,48296	0,01235	0,0774438	no
XLOC_008410	g10076	37,0412	29,9506	-0,306545	-0,409174	0,46895	0,707556	no
XLOC_008411	g10077	67,5916	49,665	-0,444616	-0,662621	0,247	0,505742	no
XLOC_008412	g3922	197,134	65,6944	-1,58534	-2,42329	0,00025	0,00430702	yes
XLOC_008413	g3923	265,899	160,754	-0,726025	-1,21802	0,0303	0,142503	no
XLOC_008414	g3924	1,60425	0,22834	-2,81264	-1,32525	0,2141	0,469524	no
XLOC_008415	g3925	71,0431	172,687	1,2814	1,97337	0,00135	0,0156148	yes
XLOC_008416	g10078	0,670441	0,951843	0,505613	0	1	1	no
XLOC_008417	g10079	0,246164	0	#NAME?	0	1	1	no
XLOC_008418	g3927	2173,63	3512,55	0,692413	1,14108	0,04445	0,181723	no
XLOC_008419	g3928	278,623	184,387	-0,595575	-0,992284	0,07635	0,255715	no
XLOC_008420	g3926	69,381	61,5226	-0,173423	-0,205837	0,7109	0,862876	no
XLOC_008421	g10080	20,7368	9,35705	-1,14807	-1,74555	0,00205	0,0212577	yes
XLOC_008422	g10081	0	0	0	0	1	1	no
XLOC_008423	g3929	4,95686	4,56974	-0,117312	-0,130822	0,8176	0,917113	no
XLOC_008424	g3930	7,81325	10,5973	0,439704	0,403027	0,46935	0,707556	no
XLOC_008425	g10082	3,68055	5,06007	0,459238	0,605907	0,28675	0,549334	no
XLOC_008426	g10083	0	0	0	0	1	1	no
XLOC_008427	g10084	0	0	0	0	1	1	no
XLOC_008428	g3934	45,2348	57,9028	0,356198	0,622119	0,2803	0,542577	no
XLOC_008429	g3935	233,72	218,752	-0,0954887	-0,164338	0,7737	0,894377	no
XLOC_008430	g3938	9,46224	9,93979	0,0710344	0,0956903	0,866	0,938748	no
XLOC_008431	g3942	17,1976	31,6293	0,879054	1,39854	0,0127	0,0791835	no
XLOC_008432	g3931	57,3917	5,79334	-3,30837	-4,8895	5,00E-05	0,00120049	yes
XLOC_008433	g3932	133,237	205,471	0,624935	0,996936	0,0847	0,274597	no
XLOC_008434	g3933	27,84	25,506	-0,126323	-0,208009	0,71755	0,865764	no
XLOC_008435	g3936	15,5034	11,1159	-0,479965	-0,706971	0,2296	0,487604	no
XLOC_008436	g3937	118,541	248,968	1,07058	1,7699	0,0028	0,0269305	yes
XLOC_008437	g3939	147,251	247,974	0,751916	1,21634	0,03035	0,142533	no
XLOC_008438	g3940	67,058	82,1691	0,293187	0,495971	0,3842	0,640475	no
XLOC_008439	g3941	65,9027	89,7375	0,445374	0,749631	0,1934	0,444255	no
XLOC_008440	g592	63,2039	27,9092	-1,17927	-1,77501	0,0018	0,0192549	yes
XLOC_008441	g3943	4,40221	0,895858	-2,29689	-2,15152	0,0048	0,0398445	yes
XLOC_008442	g10085	0,653433	1,50591	1,20452	0,892419	0,10825	0,319992	no
XLOC_008443	g10086	13,6247	10,2541	-0,410016	-0,554787	0,3282	0,589523	no
XLOC_008444	g3945	32,4131	24,1492	-0,424603	-0,683521	0,23635	0,49424	no
XLOC_008445	g3946	1532,06	1974,55	0,366056	0,510694	0,3499	0,610718	no
XLOC_008446	g3944	45,7215	47,9146	0,0675938	0,113752	0,84235	0,927861	no
XLOC_008447	g3947	50,8768	14,5574	-1,80525	-2,35879	0,0002	0,00367103	yes
XLOC_008448	g10087	69,2892	55,7385	-0,313957	-0,46448	0,4213	0,671618	no
XLOC_008449	g10088	282,044	285,475	0,0174446	0,0293127	0,9567	0,980821	no
XLOC_008450	g43	0	0	0	0	1	1	no
XLOC_008451	g10089	5,67879	8,66013	0,608805	0,706525	0,22185	0,478911	no
XLOC_008452	g10090	0	2,37684	inf	0	1	1	no
XLOC_008453	g10091	6,64369	3,67567	-0,853976	-0,852618	0,14455	0,380558	no
XLOC_008454	g10092	41,5587	44,553	0,100372	0,0931046	0,8676	0,939446	no
XLOC_008455	g10095	267,62	215,209	-0,314448	-0,521773	0,3589	0,619938	no
XLOC_008456	g10093	30,5503	23,306	-0,390487	-0,62202	0,2699	0,531359	no
XLOC_008457	g10094	574,004	486,084	-0,239854	-0,40573	0,48075	0,715629	no
XLOC_008458	g10096	11,7492	9,83766	-0,256174	-0,281262	0,62805	0,813648	no
XLOC_008459	g10097	21,6899	25,7912	0,249858	0,319364	0,5726	0,779083	no
XLOC_008460	g10098	472,21	674,599	0,514601	0,869212	0,13285	0,362184	no
XLOC_008461	g10099	114,599	108,949	-0,0729371	-0,116593	0,8386	0,926383	no
XLOC_008462	g3948	0,0462086	0,278994	2,594	0	1	1	no
XLOC_008463	g3949	55,1879	15,7343	-1,81044	-2,62004	5,00E-05	0,00120049	yes

XLOC_008464	g3950	2,82604	11,5615	2,03247	2,4341	0,00025	0,00430702	yes
XLOC_008465	g3951	5,3804	4,23896	-0,344003	-0,390763	0,50185	0,730642	no
XLOC_008466	g10100	69,653	55,6756	-0,323141	-0,42835	0,45865	0,701036	no
XLOC_008467	g10101	53,6073	12,7419	-2,07285	-2,43675	0,00035	0,00552572	yes
XLOC_008468	g10102	46,109	38,5549	-0,258135	-0,445644	0,4332	0,680209	no
XLOC_008469	g10103	2,98551	3,92787	0,395771	0,340399	0,5488	0,765215	no
XLOC_008470	g10104	0	0	0	0	1	1	no
XLOC_008471	g10105	0	0,040376	inf	0	1	1	no
XLOC_008472	g10106	0	0	0	0	1	1	no
XLOC_008473	g10107	32,5498	20,037	-0,699984	-0,849195	0,13195	0,360732	no
XLOC_008474	g10108	27,8834	16,2972	-0,774778	-1,23005	0,03975	0,169274	no
XLOC_008475	g10109	148,59	155,546	0,0660047	0,112946	0,835	0,92459	no
XLOC_008476	g3952	419,894	64,9436	-2,69277	-3,97614	5,00E-05	0,00120049	yes
XLOC_008477	g10110	34,9397	37,6572	0,108059	0,157174	0,7806	0,898182	no
XLOC_008478	g10111	0	0	0	0	1	1	no
XLOC_008479	g10113	9,64294	9,54359	-0,0149409	-0,0163073	0,9747	0,987675	no
XLOC_008480	g10112	1,00453	1,48733	0,566201	0	1	1	no
XLOC_008481	g3953	0	0	0	0	1	1	no
XLOC_008482	g3954	107,419	112,11	0,0616535	0,0973384	0,85895	0,935547	no
XLOC_008483	g3955	16,6164	20,214	0,28274	0,459909	0,4079	0,660523	no
XLOC_008484	g3956	462,264	376,395	-0,296469	-0,403685	0,48415	0,717963	no
XLOC_008485	g3957	69,3487	56,2059	-0,303147	-0,468246	0,4238	0,67309	no
XLOC_008486	g10115	74,526	55,4535	-0,426465	-0,700472	0,23025	0,487612	no
XLOC_008487	g10114	21,3583	16,0069	-0,416106	-0,694835	0,22165	0,478585	no
XLOC_008488	g3958	149,069	134,112	-0,152547	-0,234736	0,67255	0,841116	no
XLOC_008489	g3960	24,4288	28,0764	0,200774	0,316609	0,5651	0,774704	no
XLOC_008490	g3962	197,714	137,918	-0,519605	-0,787115	0,159	0,39933	no
XLOC_008491	g3964	77,5367	111,027	0,517959	0,887955	0,11425	0,331588	no
XLOC_008492	g3959	42,0605	36,9697	-0,186123	-0,294409	0,6038	0,799232	no
XLOC_008493	g3961	159,42	335,248	1,0724	1,81434	0,0019	0,0201056	yes
XLOC_008494	g3963	110,02	45,2406	-1,28208	-2,05027	0,00035	0,00552572	yes
XLOC_008495	g10116	12,9253	2,12497	-2,60469	-2,19485	0,2549	0,514304	no
XLOC_008496	g10118	129,492	187,691	0,535498	0,75069	0,17725	0,424122	no
XLOC_008497	g10117	97,7705	131,387	0,426356	0,619728	0,28665	0,549334	no
XLOC_008498	g3965	9,19808	10,634	0,209278	0,218292	0,6979	0,85623	no
XLOC_008499	g10119	0	0	0	0	1	1	no
XLOC_008500	g3966	0,22794	0,0739297	-1,62443	0	1	1	no
XLOC_008501	g3967	1,83781	1,68302	-0,126935	-0,0704916	0,85255	0,933661	no
XLOC_008502	g3968	2,37967	3,33123	0,485292	0,558084	0,33835	0,598323	no
XLOC_008503	g10120	0	0	0	0	1	1	no
XLOC_008504	g3969	0	0	0	0	1	1	no
XLOC_008505	g10122	10,103	11,1759	0,145614	0,149157	0,84225	0,927854	no
XLOC_008506	g10121	829,077	911,421	0,136612	0,211928	0,7063	0,860743	no
XLOC_008507	g3970	63,1622	53,182	-0,248123	-0,421577	0,46285	0,704134	no
XLOC_008508	g3971	137,205	240,771	0,811333	1,22925	0,02415	0,123967	no
XLOC_008509	g10123	8,22372	19,6918	1,25973	1,75748	0,00315	0,0293482	yes
XLOC_008510	g10124	0	0	0	0	1	1	no
XLOC_008511	g10125	382,747	300,744	-0,347856	-0,579238	0,31265	0,573414	no
XLOC_008512	g593	8,59998	20,5356	1,25572	1,75039	0,00435	0,0372099	yes
XLOC_008513	g10126	23,0187	37,4682	0,702862	0,955132	0,10465	0,312835	no
XLOC_008514	g10127	0	0	0	0	1	1	no
XLOC_008515	g10128	2,48183	0	#NAME?	#NAME?	0,00055	0,00788467	yes
XLOC_008516	g3972	14,1175	13,1488	-0,102548	-0,144079	0,7937	0,904927	no
XLOC_008517	g10129	39,0743	45,9189	0,232869	0,342687	0,5557	0,768804	no
XLOC_008518	g10130	16,0242	9,74063	-0,718167	-0,95201	0,10285	0,308959	no
XLOC_008519	g10131	22,9659	30,8959	0,427922	0,433178	0,61205	0,804919	no
XLOC_008520	g10132	0	0	0	0	1	1	no
XLOC_008521	g10133	18,4158	17,806	-0,0485838	-0,0415933	0,95765	0,981334	no
XLOC_008522	g10134	4,62505	5,20522	0,170491	0,182354	0,7468	0,880922	no
XLOC_008523	g10135	83,6165	72,9051	-0,197769	-0,314714	0,57905	0,782263	no
XLOC_008524	g10136	0	0	0	0	1	1	no
XLOC_008525	g10137	62,9403	82,6701	0,39338	0,655144	0,2464	0,505251	no
XLOC_008526	g3973	26,8205	31,1622	0,216465	0,339284	0,55885	0,770773	no
XLOC_008527	g3975	89,9978	153,254	0,767968	1,28546	0,0242	0,123967	no
XLOC_008528	g3977	101,937	94,8666	-0,10371	-0,157562	0,78295	0,899946	no
XLOC_008529	g3974	34,8161	36,5218	0,0690045	0,118403	0,8337	0,924151	no
XLOC_008530	g3976	546,637	724,119	0,405643	0,678382	0,2393	0,497337	no
XLOC_008531	g10138	0,100961	0,986806	3,28897	0	1	1	no
XLOC_008532	g3978	16,6536	30,1647	0,857026	1,07118	0,0638	0,229073	no
XLOC_008533	g3979	2,21506	1,4754	-0,586237	-0,487677	0,36855	0,628654	no
XLOC_008534	g3981	31,1587	32,999	0,0827852	0,122028	0,82245	0,919263	no
XLOC_008535	g3980	21,717	16,858	-0,365394	-0,514166	0,3635	0,623397	no
XLOC_008536	g10139	69,2817	65,7649	-0,0751562	-0,124098	0,82655	0,92089	no
XLOC_008537	g10140	0	0	0	0	1	1	no
XLOC_008538	g10141	72,654	97,6158	0,426072	0,681741	0,23405	0,491525	no
XLOC_008539	g10142	0	0	0	0	1	1	no
XLOC_008540	g10144	0	0	0	0	1	1	no
XLOC_008541	g10143	0,0422315	0	#NAME?	0	1	1	no
XLOC_008542	g10145	0	1,41938	inf	0	1	1	no
XLOC_008543	g3982	0	0	0	0	1	1	no
XLOC_008544	g10146	0	0	0	0	1	1	no
XLOC_008545	g10147	50,6518	47,4581	-0,0939609	-0,151856	0,78145	0,898633	no
XLOC_008546	g3983	0	0,0508169	inf	0	1	1	no
XLOC_008547	g10148	7,99145	11,1283	0,477699	0,616875	0,262	0,52198	no
XLOC_008548	g10149	4,21794	2,10381	-1,00353	-0,917284	0,10925	0,321786	no
XLOC_008549	g10150	193,563	177,01	-0,128975	-0,146317	0,80135	0,908537	no
XLOC_008550	g10151	307,781	107,466	-1,51802	-2,37422	5,00E-05	0,00120049	yes
XLOC_008551	g3985	54,6898	33,385	-0,71207	-1,20944	0,03535	0,157432	no
XLOC_008552	g3986	17,155	18,6273	0,118792	0,168472	0,7733	0,894251	no
XLOC_008553	g3984	8,66053	10,1812	0,233383	0,329994	0,57305	0,779376	no
XLOC_008554	g3987	0	0	0	0	1	1	no
XLOC_008555	g10152	38,5098	35,4771	-0,118336	-0,191195	0,74145	0,877635	no

XLOC_008556	g10153	0	0	0	0	1	1	no
XLOC_008557	g3988	260,631	963,788	1,88671	2,4592	0,00025	0,00430702	yes
XLOC_008558	g10154	62,9544	126,193	1,00326	1,66509	0,005	0,0410192	yes
XLOC_008559	g594	3,35699	0	#NAME?	0	1	1	no
XLOC_008560	g10155	12,8817	7,17866	-0,843533	-1,25837	0,0287	0,137614	no
XLOC_008561	g10156	9,71478	12,3679	0,348343	0,457053	0,4284	0,677386	no
XLOC_008562	g10157	1192,41	294,965	-2,01527	-2,58116	5,00E-05	0,00120049	yes
XLOC_008563	g10158	54,3825	19,2728	-1,49658	-2,16207	0,0004	0,0061761	yes
XLOC_008564	g10159	57,0297	53,1639	-0,101265	-0,148332	0,7939	0,904945	no
XLOC_008565	g10160	0	0	0	0	1	1	no
XLOC_008566	g3989	27,1553	16,7077	-0,700719	-1,02091	0,07565	0,254064	no
XLOC_008567	g10161	26,8101	18,8556	-0,507782	-0,790543	0,16805	0,410959	no
XLOC_008568	g595	0	0	0	0	1	1	no
XLOC_008569	g10162	0	0	0	0	1	1	no
XLOC_008570	g3991	15,2379	21,8829	0,522139	0,800034	0,1539	0,39191	no
XLOC_008571	g3990	9,89611	9,04746	-0,129348	-0,189072	0,7416	0,877635	no
XLOC_008572	g10164	0	0	0	0	1	1	no
XLOC_008573	g10163	0	0,0165223	inf	0	1	1	no
XLOC_008574	g10165	15,4326	14,753	-0,0649765	-0,102194	0,8526	0,933661	no
XLOC_008575	g10166	0	0,679057	inf	0	1	1	no
XLOC_008576	g44	16,9407	16,031	-0,0796351	-0,113571	0,8394	0,926482	no
XLOC_008577	g46	30,3534	25,9876	-0,224034	-0,36471	0,5176	0,742416	no
XLOC_008578	g47	182,676	154,361	-0,242979	-0,368938	0,5148	0,740817	no
XLOC_008579	g48	84,9682	55,0752	-0,625522	-1,06773	0,06055	0,221453	no
XLOC_008580	g50	62,3624	15,6966	-1,99023	-3,2821	5,00E-05	0,00120049	yes
XLOC_008581	g52	0,649479	0,820958	0,338024	0	1	1	no
XLOC_008582	g45	1,50387	4,80001	1,67435	1,0971	0,0699	0,242551	no
XLOC_008583	g49	183,97	126,654	-0,538586	-0,859632	0,1163	0,334524	no
XLOC_008584	g51	66,2418	68,1412	0,0407853	0,0637922	0,90885	0,961172	no
XLOC_008585	g53	95,8479	85,7211	-0,161095	-0,258235	0,647	0,82595	no
XLOC_008586	g10167	4,18617	5,92801	0,501918	0,601376	0,28925	0,55259	no
XLOC_008587	g10168	16,5306	86,6027	2,38927	3,51848	5,00E-05	0,00120049	yes
XLOC_008588	g10169	57,5776	64,5691	0,165335	0,259796	0,6414	0,822157	no
XLOC_008589	g3992	0,206457	0	#NAME?	0	1	1	no
XLOC_008590	g3993	28,3173	17,3882	-0,703572	-0,960778	0,103	0,309315	no
XLOC_008591	g10170	14,2366	12,607	-0,175385	-0,180856	0,75965	0,886717	no
XLOC_008592	g10171	4,16884	1,78665	-1,22239	-0,906936	0,133	0,362492	no
XLOC_008593	g10172	0	0	0	0	1	1	no
XLOC_008594	g10173	0	0	0	0	1	1	no
XLOC_008595	g3994	0	0	0	0	1	1	no
XLOC_008596	g3996	13,5313	12,0757	-0,164194	-0,207552	0,7082	0,861576	no
XLOC_008597	g3995	9,2907	10,3788	0,159782	0,253397	0,65385	0,829883	no
XLOC_008598	g10176	1,10367	3,59541	1,70384	0,996953	0,09845	0,300802	no
XLOC_008599	g10174	120,36	232,014	0,946863	1,54607	0,00785	0,0561859	no
XLOC_008600	g10175	59,6654	49,2756	-0,276024	-0,453897	0,4169	0,669057	no
XLOC_008601	g10177	390,043	304,378	-0,35777	-0,549255	0,3551	0,615875	no
XLOC_008602	g10178	327,763	411,051	0,326666	0,456938	0,4501	0,694824	no
XLOC_008603	g10179	0	0	0	0	1	1	no
XLOC_008604	g10180	0	0	0	0	1	1	no
XLOC_008605	g10181	0	0	0	0	1	1	no
XLOC_008606	g10182	101,087	92,1114	-0,134153	-0,206984	0,7174	0,865764	no
XLOC_008607	g10183	31,5417	29,4246	-0,100241	-0,171605	0,7612	0,887449	no
XLOC_008608	g10184	116,192	235,093	1,01671	1,64367	0,0034	0,0310297	yes
XLOC_008609	g10185	24,727	22,4838	-0,137202	-0,217845	0,7011	0,858026	no
XLOC_008610	g10186	209,621	95,0204	-1,14147	-1,87102	0,0009	0,0115984	yes
XLOC_008611	g10187	29,0715	41,0194	0,496699	0,658918	0,2339	0,491315	no
XLOC_008612	g10188	0	0	0	0	1	1	no
XLOC_008613	g10189	221,172	215,664	-0,036385	-0,0578814	0,91685	0,963869	no
XLOC_008614	g10190	1,5858	7,46852	2,23561	1,66773	0,09535	0,295281	no
XLOC_008615	g10191	0,121986	0,259826	1,09083	0	1	1	no
XLOC_008616	g10192	9,49279	3,87917	-1,29108	-1,28628	0,0362	0,159196	no
XLOC_008617	g596	0	0	0	0	1	1	no
XLOC_008618	g10193	30,2891	14,5243	-1,06033	-1,2988	0,0244	0,124472	no
XLOC_008619	g10194	18,8288	68,9155	1,87189	2,24445	0,00035	0,00552572	yes
XLOC_008620	g10195	253,62	189,706	-0,418903	-0,615914	0,2928	0,555278	no
XLOC_008621	g10196	772,011	1258,89	0,705458	1,14364	0,04005	0,17033	no
XLOC_008622	g3997	223,698	193,03	-0,212725	-0,341579	0,55725	0,770071	no
XLOC_008623	g10197	0	0	0	0	1	1	no
XLOC_008624	g10198	0	0	0	0	1	1	no
XLOC_008625	g10199	2,72162	2,34394	-0,215531	-0,199115	0,724	0,86956	no
XLOC_008626	g10200	0	0,0142915	inf	0	1	1	no
XLOC_008627	g3998	132,429	107,933	-0,295082	-0,469151	0,4023	0,656026	no
XLOC_008628	g3999	20,3047	33,0316	0,702037	1,10093	0,05605	0,211697	no
XLOC_008629	g10201	161,242	137,024	-0,234794	-0,346924	0,5574	0,770071	no
XLOC_008630	g597	142,629	135,652	-0,0723577	-0,0781732	0,89255	0,951872	no
XLOC_008631	g10202	10,6186	21,7729	1,03594	1,48085	0,0084	0,0588359	no
XLOC_008632	g10203	0	0	0	0	1	1	no
XLOC_008633	g10204	352,485	417,122	0,242908	0,355163	0,53935	0,758039	no
XLOC_008634	g10205	137,671	107,17	-0,361324	-0,582077	0,3077	0,568599	no
XLOC_008635	g10206	0	0	0	0	1	1	no
XLOC_008636	g4000	0	0	0	0	1	1	no
XLOC_008637	g55	63,9388	51,1556	-0,3218	-0,510323	0,37075	0,629539	no
XLOC_008638	g58	13,4957	11,9062	-0,180781	-0,280599	0,6258	0,812405	no
XLOC_008639	g62	72,2227	82,5332	0,192522	0,312886	0,58395	0,784888	no
XLOC_008640	g64	56,5861	23,5807	-1,26284	-2,01778	0,00095	0,0120686	yes
XLOC_008641	g65	14,3006	16,3734	0,195278	0,26045	0,6451	0,825069	no
XLOC_008642	g67	27,0118	28,9045	0,0977043	0,163357	0,77025	0,893264	no
XLOC_008643	g54	141,162	97,0884	-0,53998	-0,874689	0,12565	0,349641	no
XLOC_008644	g56	38,0736	36,7052	-0,0528067	-0,0878632	0,87435	0,943426	no
XLOC_008645	g57	3,8367	5,61197	0,548642	0,819207	0,14435	0,380439	no
XLOC_008646	g59	43,1755	37,4637	-0,20472	-0,346866	0,54315	0,760875	no
XLOC_008647	g60	19,5893	17,109	-0,195311	-0,324928	0,55775	0,77025	no

XLOC_008648	g61	3,73293	5,97927	0,679658	0,969492	0,0922	0,289359	no
XLOC_008649	g63	1,64906	0,800051	-1,04348	-0,90496	0,16815	0,410959	no
XLOC_008650	g66	75,2715	73,2095	-0,0400736	-0,0668462	0,90795	0,96068	no
XLOC_008651	g10207	12,9125	12,0999	-0,0937816	-0,0834598	0,88235	0,947324	no
XLOC_008652	g4001	52,1021	37,7987	-0,463004	-0,754708	0,1843	0,432455	no
XLOC_008653	g10208	32,6511	23,8252	-0,454646	-0,764891	0,184	0,432165	no
XLOC_008654	g4002	7,30603	22,0146	1,5913	1,79288	0,00195	0,0205021	yes
XLOC_008655	g4003	12,1072	27,0362	1,15903	1,15005	0,0488	0,193466	no
XLOC_008656	g4005	0	0,88241	inf	0	1	1	no
XLOC_008657	g4006	164,594	79,8511	-1,04353	-1,38735	0,01785	0,10005	no
XLOC_008658	g4004	0,899228	0,950831	0,0805029	0	1	1	no
XLOC_008659	g10209	3,58741	6,05748	0,755774	0,645032	0,3668	0,626431	no
XLOC_008660	g10210	0	0	0	0	1	1	no
XLOC_008661	g10211	32,7075	27,4734	-0,251587	-0,307302	0,59035	0,790204	no
XLOC_008662	g10213	29,3816	75,7629	1,36658	2,14015	0,0003	0,00496796	yes
XLOC_008663	g10212	15,6548	48,0183	1,61697	2,36211	0,00015	0,00294012	yes
XLOC_008664	g4007	192,579	235,362	0,289433	0,460476	0,42055	0,671265	no
XLOC_008665	g4010	302,224	360,47	0,254262	0,421174	0,4638	0,704924	no
XLOC_008666	g4012	17,8211	19,588	0,136388	0,182473	0,74945	0,881917	no
XLOC_008667	g4014	159,513	244,115	0,613887	0,976953	0,0917	0,28825	no
XLOC_008668	g4015	105,37	197,116	0,903579	1,53931	0,00625	0,0479867	yes
XLOC_008669	g4016	3,86371	2,777	-0,476458	-0,600389	0,28955	0,552637	no
XLOC_008670	g4008	19,4503	10,7038	-0,86167	-1,17656	0,03585	0,158223	no
XLOC_008671	g4009	163,859	276,28	0,753679	1,29813	0,0238	0,122944	no
XLOC_008672	g4011	113,862	114,919	0,0133364	0,0182515	0,9738	0,987624	no
XLOC_008673	g4013	3,42524	1,76729	-0,954665	-1,27865	0,0281	0,135999	no
XLOC_008674	g599	7,03256	6,16623	-0,189661	-0,207	0,71975	0,867399	no
XLOC_008675	g600	24,9093	16,9524	-0,555193	-0,900717	0,1138	0,330626	no
XLOC_008676	g601	2,75918	7,29067	1,40181	1,34183	0,02285	0,119609	no
XLOC_008677	g603	37,3184	102,967	1,46423	2,1142	0,0004	0,0061761	yes
XLOC_008678	g606	17,3865	46,1268	1,40763	2,24477	5,00E-05	0,00120049	yes
XLOC_008679	g607	47,2331	45,7874	-0,0448475	-0,0712014	0,89905	0,95533	no
XLOC_008680	g609	290,417	246,164	-0,238507	-0,377534	0,50195	0,730642	no
XLOC_008681	g610	8,55577	5,17475	-0,725408	-1,01659	0,08325	0,27169	no
XLOC_008682	g613	32,2765	40,568	0,329857	0,53783	0,3414	0,60157	no
XLOC_008683	g616	20,009	14,7553	-0,439417	-0,728569	0,1995	0,451924	no
XLOC_008684	g598	22,6602	45,0321	0,990791	1,30232	0,02805	0,135891	no
XLOC_008685	g602	19,6007	59,7682	1,60848	2,35259	0,0001	0,00209829	yes
XLOC_008686	g604	41,612	52,6651	0,339848	0,584258	0,3069	0,567991	no
XLOC_008687	g605	26,8582	37,2379	0,471407	0,79789	0,15405	0,39191	no
XLOC_008688	g608	41,6426	25,8487	-0,687965	-1,17474	0,0373	0,162649	no
XLOC_008689	g611	17,2719	29,4965	0,772117	1,07202	0,06305	0,226962	no
XLOC_008690	g612	7,07887	4,50628	-0,65158	-0,785964	0,1742	0,420013	no
XLOC_008691	g614	10,7344	11,1582	0,0558608	0,0776354	0,8893	0,950678	no
XLOC_008692	g615	5,80797	5,63711	-0,0430791	-0,0565533	0,923	0,96635	no
XLOC_008693	g10214	0	0	0	0	1	1	no
XLOC_008694	g618	8,75031	7,4443	-0,233197	-0,380898	0,5018	0,730642	no
XLOC_008695	g617	18,2947	12,7362	-0,522488	-0,780351	0,16315	0,405236	no
XLOC_008696	g619	31,5271	68,4463	1,11838	1,85286	0,0015	0,0168343	yes
XLOC_008697	g10215	0	0	0	0	1	1	no
XLOC_008698	g4017	0,0181131	0,0632215	1,80338	0	1	1	no
XLOC_008699	g10216	0	0	0	0	1	1	no
XLOC_008700	g10217	9,50928	10,7611	0,178413	0,232129	0,6876	0,850193	no
XLOC_008701	g10218	4,10523	7,27758	0,825995	0,688789	0,2288	0,486956	no
XLOC_008702	g10219	10,1127	0	#NAME?	#NAME?	0,12245	0,345717	no
XLOC_008703	g10220	173,718	117,146	-0,568436	-0,670189	0,23005	0,487612	no
XLOC_008704	g10221	30,4408	43,6601	0,52031	0,729586	0,20005	0,452439	no
XLOC_008705	g10222	96,8321	159,961	0,724161	1,24561	0,03425	0,153718	no
XLOC_008706	g10223	14,8391	18,8435	0,344666	0,460707	0,41235	0,66501	no
XLOC_008707	g621	293,552	263,752	-0,154432	-0,189231	0,7271	0,870385	no
XLOC_008708	g625	7,5743	11,9451	0,657233	0,632849	0,4314	0,678624	no
XLOC_008709	g626	2,80874	1,4768	-0,92745	-1,0737	0,05975	0,219875	no
XLOC_008710	g628	49,717	51,455	0,0495717	0,076131	0,8884	0,950336	no
XLOC_008711	g629	100,297	67,8261	-0,564363	-0,910837	0,10915	0,321685	no
XLOC_008712	g630	104,242	88,525	-0,235774	-0,404028	0,4755	0,712433	no
XLOC_008713	g631	30,4711	54,2792	0,832961	1,43012	0,01165	0,0741432	no
XLOC_008714	g634	72,043	65,0618	-0,147047	-0,232263	0,68605	0,849239	no
XLOC_008715	g636	35,7087	47,021	0,397032	0,562002	0,3068	0,567991	no
XLOC_008716	g638	50,7723	83,5549	0,718684	1,13499	0,04555	0,184606	no
XLOC_008717	g641	206,346	279,336	0,436939	0,575954	0,31035	0,571193	no
XLOC_008718	g644	16,2814	14,2529	-0,191977	-0,328558	0,57	0,777701	no
XLOC_008719	g620	49,1403	44,769	-0,134406	-0,213196	0,70305	0,859113	no
XLOC_008720	g622	109,727	112,963	0,0419377	0,0719349	0,8976	0,954356	no
XLOC_008721	g623	62,9093	88,3462	0,489894	0,777572	0,16835	0,411141	no
XLOC_008722	g624	30,1729	40,1202	0,411074	0,701321	0,2126	0,468106	no
XLOC_008723	g627	1,39479	3,40931	1,28943	1,54098	0,00775	0,0557955	no
XLOC_008724	g632	39,9461	35,0848	-0,18721	-0,316061	0,57755	0,781096	no
XLOC_008725	g633	43,469	52,6236	0,275724	0,470263	0,42985	0,678093	no
XLOC_008726	g635	43,4704	87,7582	1,0135	1,69713	0,00305	0,028799	yes
XLOC_008727	g637	259,705	311,538	0,262534	0,407117	0,47315	0,710415	no
XLOC_008728	g639	118,608	110,266	-0,105212	-0,166233	0,771	0,893264	no
XLOC_008729	g640	82,1305	58,5449	-0,488374	-0,798812	0,16595	0,409043	no
XLOC_008730	g642	64,1396	114,152	0,831675	1,42268	0,01605	0,0927124	no
XLOC_008731	g643	104,396	132,413	0,342978	0,579307	0,3058	0,567131	no
XLOC_008732	g10224	601,353	570,481	-0,0760335	-0,111947	0,8355	0,924776	no
XLOC_008733	g10226	182,412	157,122	-0,215321	-0,35513	0,52235	0,745347	no
XLOC_008734	g10225	79,6798	72,9496	-0,127314	-0,216817	0,70685	0,860848	no
XLOC_008735	g10227	28,4548	30,1007	0,0811278	0,132414	0,8098	0,912533	no
XLOC_008736	g4018	0	0	0	0	1	1	no
XLOC_008737	g4019	0	0,0570584	inf	0	1	1	no
XLOC_008738	g10228	0	0	0	0	1	1	no
XLOC_008739	g10229	7,1456	8,6658	0,278278	0,320852	0,58215	0,784401	no

XLOC_008740	g645	91,5766	111,659	0,286054	0,393235	0,48835	0,721433	no
XLOC_008741	g647	105,671	108,128	0,0331718	0,0556001	0,9201	0,964803	no
XLOC_008742	g646	33,2395	54,5696	0,715201	1,00229	0,07795	0,259922	no
XLOC_008743	g648	31,9048	34,1894	0,0997773	0,165109	0,77735	0,895827	no
XLOC_008744	g10230	0	0	0	0	1	1	no
XLOC_008745	g10231	63,7287	81,7836	0,359869	0,573986	0,31775	0,578273	no
XLOC_008746	g10232	22,064	13,4475	-0,714355	-0,928646	0,10975	0,322968	no
XLOC_008747	g4020	0	0	0	0	1	1	no
XLOC_008748	g10233	10,5943	10,3239	-0,03731	-0,0598022	0,91535	0,963526	no
XLOC_008749	g649	10,2534	6,95823	-0,559308	-0,862027	0,1251	0,348604	no
XLOC_008750	g10234	24,097	68,1002	1,49881	2,37885	5,00E-05	0,00120049	yes
XLOC_008751	g4021	8,94164	7,84949	-0,187941	-0,253982	0,64945	0,827591	no
XLOC_008752	g4022	62,4499	117,898	0,91677	1,52277	0,0084	0,0588359	no
XLOC_008753	g4023	613,701	499,305	-0,297613	-0,401113	0,4924	0,723714	no
XLOC_008754	g10235	0	0	0	0	1	1	no
XLOC_008755	g10236	0	0	0	0	1	1	no
XLOC_008756	g650	0	0	0	0	1	1	no
XLOC_008757	g10237	263,855	88,325	-1,57885	-2,20779	0,00025	0,00430702	yes
XLOC_008758	g10238	275,892	206,74	-0,416286	-0,478939	0,39885	0,652731	no
XLOC_008759	g4024	0	0	0	0	1	1	no
XLOC_008760	g10239	83,8826	81,4433	-0,0425754	-0,0628385	0,9136	0,963233	no
XLOC_008761	g10240	0	0	0	0	1	1	no
XLOC_008762	g10241	0	0	0	0	1	1	no
XLOC_008763	g651	0,771871	0,165727	-2,21955	0	1	1	no
XLOC_008764	g10242	0	0	0	0	1	1	no
XLOC_008765	g10243	0	0,0514949	inf	0	1	1	no
XLOC_008766	g10244	34,2453	17,585	-0,961565	-1,4892	0,0088	0,0608992	no
XLOC_008767	g4025	28,6666	55,2802	0,947391	1,58803	0,00525	0,0423624	yes
XLOC_008768	g4026	53,5667	18,8986	-1,50305	-2,23516	0,00045	0,00669545	yes
XLOC_008769	g4027	7,44134	18,6735	1,32736	1,77942	0,0024	0,0240735	yes
XLOC_008770	g4028	341,493	254,639	-0,423405	-0,62344	0,2531	0,512145	no
XLOC_008771	g4030	49,8478	59,8816	0,264584	0,455058	0,42365	0,67307	no
XLOC_008772	g4034	19,7208	17,1182	-0,204182	-0,30776	0,5908	0,790268	no
XLOC_008773	g4029	62,7943	64,2033	0,0320152	0,0537672	0,91805	0,964054	no
XLOC_008774	g4031	12,3901	48,8007	1,97771	2,90883	5,00E-05	0,00120049	yes
XLOC_008775	g4032	14,1986	29,0943	1,03499	1,52465	0,0092	0,0627389	no
XLOC_008776	g4033	38,7835	78,0889	1,00968	1,36299	0,02395	0,123459	no
XLOC_008777	g10245	0	0	0	0	1	1	no
XLOC_008778	g4035	26,7766	18,327	-0,547005	-0,771653	0,18255	0,42989	no
XLOC_008779	g4036	82,8243	104,492	0,335259	0,537452	0,3358	0,595746	no
XLOC_008780	g4037	34,4787	73,1558	1,08527	1,80035	0,00185	0,01964	yes
XLOC_008781	g4039	181,321	144,686	-0,325623	-0,478407	0,39895	0,652731	no
XLOC_008782	g4042	95,998	88,4356	-0,118377	-0,201886	0,7256	0,869807	no
XLOC_008783	g4043	40,8275	44,7875	0,133556	0,229725	0,68685	0,849586	no
XLOC_008784	g4045	50,5487	28,6106	-0,821125	-1,39014	0,0167	0,0953453	no
XLOC_008785	g4046	44,4624	24,4733	-0,861375	-1,33217	0,0222	0,117333	no
XLOC_008786	g4038	28,2286	29,7425	0,0753664	0,124165	0,82945	0,922056	no
XLOC_008787	g4040	10,8162	22,5606	1,06061	1,59015	0,00555	0,0439524	yes
XLOC_008788	g4041	87,9073	77,646	-0,179071	-0,302715	0,6039	0,799232	no
XLOC_008789	g4044	71,1675	39,8593	-0,8363	-1,25356	0,03315	0,150989	no
XLOC_008790	g4047	0	0	0	0	1	1	no
XLOC_008791	g4048	0	0	0	0	1	1	no
XLOC_008792	g652	30,7655	24,2889	-0,341015	-0,513891	0,3618	0,621784	no
XLOC_008793	g10246	0,0490868	0,220033	2,16431	0	1	1	no
XLOC_008794	g10247	0	0	0	0	1	1	no
XLOC_008795	g10248	49,3162	118,705	1,26724	2,11812	0,0002	0,00367103	yes
XLOC_008796	g10249	0,987929	0,606765	-0,703268	0	1	1	no
XLOC_008797	g10250	255,706	299,01	0,225706	0,269934	0,63985	0,82122	no
XLOC_008798	g10251	0	0	0	0	1	1	no
XLOC_008799	g4049	36,5042	58,5815	0,682383	1,09709	0,051	0,199133	no
XLOC_008800	g10252	0,845585	1,79424	1,08535	1,17233	0,04275	0,177582	no
XLOC_008801	g10253	0	0,420587	inf	0	1	1	no
XLOC_008802	g4050	0	0	0	0	1	1	no
XLOC_008803	g10254	8,65651	13,5736	0,648943	0,824592	0,1554	0,392876	no
XLOC_008804	g4051	11,5924	15,1387	0,385066	0,449086	0,43425	0,681173	no
XLOC_008805	g653	0	0	0	0	1	1	no
XLOC_008806	g10255	0	0	0	0	1	1	no
XLOC_008807	g10256	0,0811518	1,26939	3,96736	1,01428	0,12365	0,347009	no
XLOC_008808	g4052	9,27946	8,40068	-0,143536	-0,176429	0,7581	0,885412	no
XLOC_008809	g10257	3,59919	6,52915	0,859224	1,02999	0,0761	0,255139	no
XLOC_008810	g10258	8,06618	10,7598	0,415696	0,583255	0,30315	0,564534	no
XLOC_008811	g10259	0,101909	0,19095	0,905915	0	1	1	no
XLOC_008812	g10260	0	0	0	0	1	1	no
XLOC_008813	g10261	0	0	0	0	1	1	no
XLOC_008814	g4053	0	0	0	0	1	1	no
XLOC_008815	g10262	168,521	48,9712	-1,78292	-2,80768	5,00E-05	0,00120049	yes
XLOC_008816	g4054	25,1459	4,78118	-2,39488	-3,55011	5,00E-05	0,00120049	yes
XLOC_008817	g10263	17,9931	12,905	-0,479516	-0,590581	0,301	0,563121	no
XLOC_008818	g10264	0	0	0	0	1	1	no
XLOC_008819	g10265	0	0	0	0	1	1	no
XLOC_008820	g10266	128,449	169,546	0,400476	0,660746	0,2375	0,495485	no
XLOC_008821	g10267	5,42134	16,3701	1,59434	2,19884	0,0002	0,00367103	yes
XLOC_008822	g10268	0	0	0	0	1	1	no
XLOC_008823	g4055	20,8912	26,2783	0,330976	0,396514	0,49245	0,723714	no
XLOC_008824	g10269	108,759	99,9807	-0,12141	-0,185942	0,7407	0,87698	no
XLOC_008825	g4056	17,1609	20,4016	0,249552	0,412143	0,472	0,70959	no
XLOC_008826	g4057	15,9769	3,75818	-2,08788	-2,76422	5,00E-05	0,00120049	yes
XLOC_008827	g4058	66,1626	70,6759	0,0952023	0,155684	0,7886	0,902465	no
XLOC_008828	g4059	0,587032	1,25255	1,09336	0,95941	0,1053	0,314204	no
XLOC_008829	g4061	18,0072	14,3591	-0,326611	-0,530293	0,34515	0,605461	no
XLOC_008830	g4064	42,6161	40,358	-0,0785433	-0,135038	0,813	0,914613	no
XLOC_008831	g4065	4,37462	16,7759	1,93916	2,34997	0,00045	0,00669545	yes

XLOC_008832	g4067	65,326	57,3552	-0,187733	-0,298529	0,59885	0,795658	no
XLOC_008833	g4060	45,6236	40,2824	-0,17963	-0,267594	0,63245	0,816225	no
XLOC_008834	g4062	52,758	55,0067	0,0602183	0,0898062	0,87155	0,942376	no
XLOC_008835	g4063	64,1188	83,147	0,374916	0,611952	0,2838	0,546561	no
XLOC_008836	g4066	16,6049	27,9972	0,753671	1,28637	0,0251	0,126922	no
XLOC_008837	g10270	0	0	0	0	1	1	no
XLOC_008838	g10273	323,014	394,086	0,286916	0,480536	0,3913	0,646031	no
XLOC_008839	g10274	10,871	8,11625	-0,421598	-0,609797	0,28865	0,551993	no
XLOC_008840	g10275	174,263	119,77	-0,540997	-0,876951	0,1167	0,334988	no
XLOC_008841	g10277	634,772	578,862	-0,133019	-0,211338	0,7062	0,860728	no
XLOC_008842	g10271	9,98617	8,89542	-0,166869	-0,233825	0,681	0,846464	no
XLOC_008843	g10272	48,3916	56,2346	0,216704	0,311705	0,5838	0,784861	no
XLOC_008844	g10276	42,4067	25,9337	-0,709467	-0,837684	0,1461	0,382791	no
XLOC_008845	g4069	2,65931	1,8258	-0,542522	-0,567818	0,3137	0,574405	no
XLOC_008846	g4070	32,6469	29,5289	-0,144819	-0,238826	0,6777	0,844286	no
XLOC_008847	g4068	3,52895	3,18248	-0,149089	-0,124825	0,8298	0,922056	no
XLOC_008848	g4071	4,22245	6,57606	0,639144	0,927943	0,1167	0,334988	no
XLOC_008849	g4072	11,3662	20,1836	0,828427	1,0573	0,0721	0,24756	no
XLOC_008850	g10278	311,117	1358,45	2,12643	3,23467	5,00E-05	0,00120049	yes
XLOC_008851	g10279	39,4627	38,3988	-0,0394278	-0,0562035	0,92355	0,966357	no
XLOC_008852	g10280	45,4344	43,7765	-0,0536274	-0,0840587	0,8839	0,948208	no
XLOC_008853	g10281	242,34	232,794	-0,0579786	-0,0886686	0,87285	0,942536	no
XLOC_008854	g10282	11,6954	13,4411	0,200711	0,28025	0,6213	0,809691	no
XLOC_008855	g4073	8,12224	9,0672	0,15878	0,16823	0,77665	0,895794	no
XLOC_008856	g4074	212,708	171,023	-0,314678	-0,523005	0,34365	0,603977	no
XLOC_008857	g4075	67,9114	39,0546	-0,798161	-1,09777	0,04985	0,196361	no
XLOC_008858	g4076	66,3092	66,2187	-0,0019702	-0,00328707	0,99565	0,997682	no
XLOC_008859	g4077	52,2707	23,6318	-1,14527	-1,5646	0,0098	0,0655112	no
XLOC_008860	g10283	18,869	14,675	-0,362659	-0,524403	0,3593	0,619938	no
XLOC_008861	g10284	67,2144	70,2669	0,0640743	0,106893	0,85285	0,933666	no
XLOC_008862	g4079	295,53	296,855	0,0064542	0,0109189	0,98525	0,992425	no
XLOC_008863	g4078	382,463	454,765	0,249802	0,413035	0,4783	0,713924	no
XLOC_008864	g4080	0	0	0	0	1	1	no
XLOC_008865	g4081	9,57447	7,27608	-0,396031	-0,64798	0,25145	0,510173	no
XLOC_008866	g10285	0	0	0	0	1	1	no
XLOC_008867	g10286	43,0059	64,0934	0,575642	0,944711	0,10425	0,31221	no
XLOC_008868	g10287	47,4742	172,836	1,86419	2,95602	5,00E-05	0,00120049	yes
XLOC_008869	g10288	0	0	0	0	1	1	no
XLOC_008870	g10289	0	0	0	0	1	1	no
XLOC_008871	g654	12,4633	14,1381	0,18191	0,195516	0,7356	0,874871	no
XLOC_008872	g655	10,3101	13,0651	0,341656	0,444538	0,4497	0,694824	no
XLOC_008873	g10290	88,9077	83,7659	-0,0859446	-0,144383	0,79565	0,906216	no
XLOC_008874	g10291	58,6975	59,6115	0,0222912	0,0352515	0,94965	0,977215	no
XLOC_008875	g10292	417,048	563,727	0,434784	0,711964	0,1958	0,447153	no
XLOC_008876	g10293	0	0	0	0	1	1	no
XLOC_008877	g4082	4,50156	7,07342	0,651983	0,864161	0,13825	0,370629	no
XLOC_008878	g656	40,6204	81,4326	1,0034	1,37982	0,01665	0,0951152	no
XLOC_008879	g4084	45,8456	43,8709	-0,0635195	-0,105719	0,8499	0,932412	no
XLOC_008880	g4083	6,25322	8,53211	0,448303	0,637733	0,2773	0,538905	no
XLOC_008881	g10294	22,4768	37,1066	0,723242	1,16573	0,038	0,164605	no
XLOC_008882	g10295	0	0	0	0	1	1	no
XLOC_008883	g10296	1,0547	1,03801	-0,0230154	0	1	1	no
XLOC_008884	g657	0,871893	2,12165	1,28296	1,2361	0,03755	0,163449	no
XLOC_008885	g658	25,2378	86,7018	1,78047	2,55175	5,00E-05	0,00120049	yes
XLOC_008886	g4086	1047,13	1118,31	0,0948821	0,103857	0,8477	0,930934	no
XLOC_008887	g4085	689,404	388,76	-0,826471	-1,04784	0,05985	0,219875	no
XLOC_008888	g4087	43,0664	58,4035	0,43949	0,75357	0,17815	0,424929	no
XLOC_008889	g4088	30,8957	33,2113	0,104267	0,166857	0,7707	0,893264	no
XLOC_008890	g4089	65,6787	56,732	-0,211263	-0,346616	0,5293	0,750249	no
XLOC_008891	g4090	23,822	12,6095	-0,917782	-1,28156	0,0311	0,144699	no
XLOC_008892	g10297	0	0	0	0	1	1	no
XLOC_008893	g10298	0	0	0	0	1	1	no
XLOC_008894	g4091	0,24768	1,33465	2,42992	1,59389	0,02285	0,119609	no
XLOC_008895	g10299	18,3032	18,2837	-0,00154071	-0,00246042	0,9975	0,998008	no
XLOC_008896	g4092	47,3194	47,1937	-0,00383593	-0,00612259	0,99225	0,995392	no
XLOC_008897	g4093	55,0546	74,8782	0,443681	0,738938	0,18225	0,42989	no
XLOC_008898	g659	943,519	54,1473	-4,12309	-5,18284	5,00E-05	0,00120049	yes
XLOC_008899	g661	52,2204	61,2835	0,230883	0,384751	0,4905	0,722869	no
XLOC_008900	g663	30,1327	41,0836	0,447233	0,72848	0,1923	0,442349	no
XLOC_008901	g665	11,0739	1,10463	-3,32553	-3,43842	5,00E-05	0,00120049	yes
XLOC_008902	g667	52,1454	96,9616	0,894872	1,52485	0,00765	0,0553596	no
XLOC_008903	g660	23,8214	10,386	-1,19761	-1,65971	0,0054	0,0429724	yes
XLOC_008904	g662	17,1534	13,9536	-0,297857	-0,5094	0,3596	0,620174	no
XLOC_008905	g664	409,221	400,625	-0,0306289	-0,0436616	0,93735	0,972153	no
XLOC_008906	g666	4496,22	7078,59	0,654749	0,974586	0,0919	0,288787	no
XLOC_008907	g4094	264,481	193,473	-0,451032	-0,647207	0,24095	0,499282	no
XLOC_008908	g4095	678,933	1108,71	0,707544	0,690657	0,1828	0,429978	no
XLOC_008909	g4097	67,9381	50,7094	-0,421966	-0,604234	0,2779	0,539702	no
XLOC_008910	g4099	165,279	279,308	0,75696	1,24051	0,02825	0,136523	no
XLOC_008911	g4100	95,509	104,902	0,135328	0,21316	0,6983	0,856307	no
XLOC_008912	g4101	8,65957	8,44789	-0,0357035	-0,051662	0,9302	0,969134	no
XLOC_008913	g4096	17,9958	51,4115	1,51443	1,99914	0,00145	0,0164423	yes
XLOC_008914	g4098	54,1922	36,005	-0,589887	-1,0005	0,0744	0,252007	no
XLOC_008915	g10300	67,6792	49,342	-0,455896	-0,406491	0,6609	0,833937	no
XLOC_008916	g10301	0	0	0	0	1	1	no
XLOC_008917	g4102	8,79415	13,0126	0,565289	0,836046	0,1375	0,369729	no
XLOC_008918	g669	77,4071	69,9207	-0,146747	-0,207927	0,71445	0,865069	no
XLOC_008919	g670	99,7368	178,604	0,840566	1,42399	0,01435	0,0860825	no
XLOC_008920	g671	14,7813	10,7484	-0,459644	-0,638607	0,2613	0,521008	no
XLOC_008921	g674	214,072	171,73	-0,317954	-0,533717	0,3412	0,601413	no
XLOC_008922	g675	109,984	59,1307	-0,89531	-1,53519	0,0071	0,0523438	no
XLOC_008923	g677	353,093	264,462	-0,416985	-0,664669	0,2434	0,502035	no

XLOC_008924	g678	25,2619	29,1113	0,204616	0,256896	0,6454	0,825131	no
XLOC_008925	g668	41,8504	46,7317	0,159159	0,25969	0,64225	0,822774	no
XLOC_008926	g672	35,7682	58,0375	0,698308	1,05919	0,0618	0,224105	no
XLOC_008927	g673	159,239	163,116	0,0347044	0,0563041	0,92455	0,966785	no
XLOC_008928	g676	106,775	72,1417	-0,565668	-0,967651	0,0902	0,284995	no
XLOC_008929	g10302	8,30878	9,70152	0,223574	0,283216	0,6194	0,808526	no
XLOC_008930	g4103	0,177935	1,14182	2,68191	0	1	1	no
XLOC_008931	g10303	987,416	3704,64	1,9076	3,09905	5,00E-05	0,00120049	yes
XLOC_008932	g4104	10,5703	1,69427	-2,64127	-2,42298	0,00025	0,00430702	yes
XLOC_008933	g4106	5,39808	2,70032	-0,999314	-1,08205	0,06985	0,242551	no
XLOC_008934	g4105	309,515	348,193	0,169879	0,280006	0,60725	0,801057	no
XLOC_008935	g10304	0	0,0881303	inf	0	1	1	no
XLOC_008936	g10305	0	0	0	0	1	1	no
XLOC_008937	g10306	0	0	0	0	1	1	no
XLOC_008938	g679	176,233	171,101	-0,0426334	-0,0707961	0,90315	0,957458	no
XLOC_008939	g681	12,7132	10,1375	-0,32663	-0,401666	0,47425	0,711556	no
XLOC_008940	g680	24,238	24,4065	0,00999619	0,0168784	0,9752	0,987875	no
XLOC_008941	g4108	39,5678	66,4826	0,748651	1,25419	0,02985	0,141402	no
XLOC_008942	g4109	20,5769	8,9995	-1,19311	-1,71241	0,00415	0,0357482	yes
XLOC_008943	g4111	18,596	16,2701	-0,192767	-0,281595	0,61835	0,808291	no
XLOC_008944	g4107	2,12096	1,94179	-0,127333	-0,117147	0,83125	0,922463	no
XLOC_008945	g4110	8,49764	8,65114	0,0258278	0,0317115	0,956	0,980564	no
XLOC_008946	g4112	20,2683	20,6274	0,0253373	0,0326426	0,9529	0,978816	no
XLOC_008947	g10307	5,34313	5,60622	0,069343	0,0398897	0,89475	0,953287	no
XLOC_008948	g10308	0	0	0	0	1	1	no
XLOC_008949	g4115	237,998	187,148	-0,346776	-0,580529	0,318	0,578273	no
XLOC_008950	g4113	48,5415	49,4064	0,0254786	0,043474	0,9379	0,972153	no
XLOC_008951	g4114	32,6549	39,8329	0,286663	0,382975	0,49435	0,724879	no
XLOC_008952	g4116	5,53689	6,47368	0,225509	0,234616	0,6847	0,848423	no
XLOC_008953	g10309	14,3253	6,70801	-1,09461	-1,5961	0,00635	0,0485646	yes
XLOC_008954	g10310	10,3899	17,4289	0,746293	0,900173	0,13275	0,362112	no
XLOC_008955	g10311	32,1479	12,5849	-1,35303	-1,95764	0,00075	0,0100752	yes
XLOC_008956	g10312	811,734	530,667	-0,613201	-1,00671	0,0738	0,251114	no
XLOC_008957	g10313	0	0	0	0	1	1	no
XLOC_008958	g4117	0	4,30413	inf	0	1	1	no
XLOC_008959	g4118	7,4037	11,5282	0,638845	0,738177	0,20575	0,460033	no
XLOC_008960	g10314	53,9921	94,3888	0,805867	1,15872	0,0401	0,170395	no
XLOC_008961	g10315	87,9587	50,2479	-0,807763	-1,14663	0,051	0,199133	no
XLOC_008962	g10317	67,9927	51,9006	-0,389628	-0,583007	0,3205	0,580544	no
XLOC_008963	g10321	21,6856	22,4013	0,0468469	0,0652198	0,9123	0,962586	no
XLOC_008964	g10316	24,8421	22,6601	-0,132635	-0,214394	0,70435	0,859387	no
XLOC_008965	g10318	14,8762	20,5599	0,466834	0,686389	0,2214	0,478466	no
XLOC_008966	g10319	14,2601	9,11351	-0,645903	-0,91935	0,0999	0,30297	no
XLOC_008967	g10320	64,6502	58,2251	-0,151013	-0,24026	0,6619	0,834706	no
XLOC_008968	g682	0,114668	0	#NAME?	0	1	1	no
XLOC_008969	g683	89,2546	79,5908	-0,165326	-0,262977	0,64485	0,824921	no
XLOC_008970	g4119	8,04028	4,49609	-0,838575	-1,01719	0,0833	0,271763	no
XLOC_008971	g4121	9,10137	8,09664	-0,168761	-0,221817	0,6941	0,854464	no
XLOC_008972	g4120	11,7873	9,6789	-0,284313	-0,441941	0,43845	0,684886	no
XLOC_008973	g4122	51,2542	19,7276	-1,37745	-2,16903	0,0003	0,00496796	yes
XLOC_008974	g4123	28,7331	30,6468	0,0930185	0,145819	0,7971	0,906383	no
XLOC_008975	g10323	197,103	205,033	0,0569109	0,0975678	0,8625	0,936559	no
XLOC_008976	g10324	38,6772	35,1535	-0,137814	-0,227396	0,68665	0,849508	no
XLOC_008977	g10326	101,202	145,582	0,524591	0,844871	0,14655	0,383459	no
XLOC_008978	g10322	152,613	292,879	0,940425	1,45074	0,0145	0,0866119	no
XLOC_008979	g10325	68,4278	80,8867	0,241319	0,419163	0,4692	0,707556	no
XLOC_008980	g10327	0	0	0	0	1	1	no
XLOC_008981	g4124	16,4345	19,2218	0,226015	0,255763	0,6535	0,829883	no
XLOC_008982	g10328	16,9645	21,0972	0,314529	0,469455	0,3882	0,643479	no
XLOC_008983	g10331	340,915	504,429	0,565237	0,950393	0,1015	0,306592	no
XLOC_008984	g10332	67,9103	94,6481	0,478942	0,818765	0,1403	0,373778	no
XLOC_008985	g10334	21,859	19,7367	-0,147348	-0,243772	0,67445	0,841991	no
XLOC_008986	g10329	152,756	130,144	-0,231123	-0,391482	0,4981	0,727878	no
XLOC_008987	g10330	8,59082	16,6889	0,958022	1,16631	0,0493	0,194819	no
XLOC_008988	g10333	62,0827	25,1025	-1,30636	-1,99556	0,00075	0,0100752	yes
XLOC_008989	g10335	66,1719	64,4861	-0,03723	-0,0646461	0,9091	0,961172	no
XLOC_008990	g4125	1,10556	0,433635	-1,35023	0	1	1	no
XLOC_008991	g685	175,885	154,922	-0,183091	-0,267268	0,6379	0,819919	no
XLOC_008992	g687	40,1746	35,1047	-0,19462	-0,325849	0,5729	0,779315	no
XLOC_008993	g688	50,1926	48,7457	-0,0422004	-0,070836	0,89945	0,955495	no
XLOC_008994	g689	2,48621	2,10187	-0,242274	-0,172753	0,81	0,912557	no
XLOC_008995	g684	10393,4	12301,8	0,243193	0,320143	0,56845	0,776705	no
XLOC_008996	g686	109,83	92,5797	-0,246509	-0,389062	0,4929	0,724054	no
XLOC_008997	g10336	11,2552	6,49414	-0,793381	-0,678651	0,22005	0,477334	no
XLOC_008998	g10337	36,9477	49,4959	0,421827	0,713783	0,2019	0,454113	no
XLOC_008999	g10338	24,0374	26,0849	0,11793	0,171279	0,77155	0,893469	no
XLOC_009000	g10339	1,96578	2,28091	0,214508	0,262705	0,64225	0,822774	no
XLOC_009001	g10340	39,0573	14,5717	-1,42242	-2,04703	0,00095	0,0120686	yes
XLOC_009002	g10341	110,251	112,131	0,0243934	0,0418132	0,94235	0,974708	no
XLOC_009003	g690	88,1458	128,261	0,541119	0,896522	0,1238	0,34705	no
XLOC_009004	g691	671,476	517,006	-0,377156	-0,620814	0,2558	0,515162	no
XLOC_009005	g10342	26,2033	37,1007	0,501699	0,641366	0,2808	0,54302	no
XLOC_009006	g4126	89,8859	151,86	0,756577	1,14798	0,04545	0,18448	no
XLOC_009007	g4127	33,8284	40,8198	0,271037	0,45656	0,42305	0,672849	no
XLOC_009008	g10343	171,229	65,7497	-1,38087	-2,07009	0,0005	0,00733931	yes
XLOC_009009	g10344	125,148	33,8062	-1,88827	-3,13496	5,00E-05	0,00120049	yes
XLOC_009010	g10345	107,666	83,1593	-0,372617	-0,616799	0,28145	0,543742	no
XLOC_009011	g10346	0	0	0	0	1	1	no
XLOC_009012	g10347	0	0	0	0	1	1	no
XLOC_009013	g10349	393,768	174,463	-1,17443	-1,44224	0,0132	0,081371	no
XLOC_009014	g10350	43,6045	47,4885	0,123099	0,179691	0,7532	0,883683	no
XLOC_009015	g10348	32,4757	35,7	0,136562	0,21331	0,70775	0,861548	no

XLOC_009016	g10351	617,554	403,604	-0,613624	-1,0168	0,07455	0,252007	no
XLOC_009017	g4128	63,8033	75,6608	0,245915	0,394384	0,4774	0,713448	no
XLOC_009018	g10352	128,142	130,151	0,02245	0,0313477	0,9528	0,978815	no
XLOC_009019	g10353	44,1389	118,953	1,43027	2,29202	0,0002	0,00367103	yes
XLOC_009020	g10354	0	0	0	0	1	1	no
XLOC_009021	g10355	22,826	54,7562	1,26234	1,88319	0,0009	0,0115984	yes
XLOC_009022	g4129	36,3486	36,5136	0,00653391	0,00793432	0,98925	0,99472	no
XLOC_009023	g4130	8,67032	15,1213	0,802426	1,159	0,0455	0,18448	no
XLOC_009024	g10356	0	0	0	0	1	1	no
XLOC_009025	g10357	0	0	0	0	1	1	no
XLOC_009026	g10358	0	0	0	0	1	1	no
XLOC_009027	g10359	0	0	0	0	1	1	no
XLOC_009028	g10360	0	0	0	0	1	1	no
XLOC_009029	g10361	69,4001	82,4913	0,249303	0,406772	0,45	0,694824	no
XLOC_009030	g10362	110,765	64,2112	-0,786605	-1,19443	0,04335	0,179015	no
XLOC_009031	g10363	48,486	36,9813	-0,390772	-0,513192	0,36425	0,62392	no
XLOC_009032	g10364	5359	11555,7	1,10857	0,742223	0,18095	0,428382	no
XLOC_009033	g10365	17,9917	26,0601	0,534508	0,84315	0,14485	0,380837	no
XLOC_009034	g10366	0	0,0500218	inf	0	1	1	no
XLOC_009035	g4131	45,4376	56,4279	0,312521	0,538618	0,3401	0,600141	no
XLOC_009036	g4132	22,5008	16,4435	-0,452462	-0,762907	0,19165	0,442199	no
XLOC_009037	g10367	2,7368	4,94793	0,854336	0,95641	0,49775	0,727692	no
XLOC_009038	g10368	105,048	53,8081	-0,965148	-1,43888	0,0135	0,082444	no
XLOC_009039	g10369	0,177959	0,164708	-0,111633	0	1	1	no
XLOC_009040	g10370	281,785	241,197	-0,224385	-0,366907	0,52235	0,745347	no
XLOC_009041	g10371	248,444	221,748	-0,163996	-0,267837	0,649	0,827363	no
XLOC_009042	g10372	219,159	175,039	-0,324305	-0,4026	0,6647	0,835806	no
XLOC_009043	g10373	153,261	196,379	0,35765	0,523733	0,3627	0,622826	no
XLOC_009044	g10374	0,0493754	0,258927	2,39068	0	1	1	no
XLOC_009045	g10375	4,2143	6,29814	0,579631	0,731103	0,2154	0,471098	no
XLOC_009046	g10376	0	0	0	0	1	1	no
XLOC_009047	g4133	51,4692	75,7217	0,556997	0,847787	0,13435	0,364251	no
XLOC_009048	g4134	16,4729	12,1384	-0,440517	-0,612906	0,2792	0,540885	no
XLOC_009049	g4135	11,956	18,9243	0,662501	1,00152	0,08175	0,26867	no
XLOC_009050	g692	0,480809	1,01805	1,08228	0	1	1	no
XLOC_009051	g10377	957,894	1081,63	0,175273	0,240365	0,67375	0,8415	no
XLOC_009052	g10378	0	0	0	0	1	1	no
XLOC_009053	g4137	31,1073	53,4586	0,781167	1,27138	0,02575	0,129013	no
XLOC_009054	g4136	100,585	168,236	0,742072	1,20806	0,0361	0,159112	no
XLOC_009055	g10379	115,283	249,693	1,11498	1,78761	0,00175	0,0188639	yes
XLOC_009056	g10380	0	0,0355674	inf	0	1	1	no
XLOC_009057	g10381	0	0	0	0	1	1	no
XLOC_009058	g10382	0,277939	0,890234	1,67942	0	1	1	no
XLOC_009059	g4138	3,09974	2,12715	-0,543226	-0,575953	0,33385	0,594729	no
XLOC_009060	g693	0	0	0	0	1	1	no
XLOC_009061	g4139	8,94888	12,0996	0,435183	0,619647	0,2846	0,546579	no
XLOC_009062	g4140	187,927	15,0043	-3,64672	-4,4828	5,00E-05	0,00120049	yes
XLOC_009063	g4141	0	0	0	0	1	1	no
XLOC_009064	g10383	0,189427	1,03471	2,44951	0	1	1	no
XLOC_009065	g10384	33,7584	45,6639	0,435808	0,596609	0,2756	0,537409	no
XLOC_009066	g10385	0	0	0	0	1	1	no
XLOC_009067	g4142	0	0	0	0	1	1	no
XLOC_009068	g10386	0	0	0	0	1	1	no
XLOC_009069	g4143	0	0	0	0	1	1	no
XLOC_009070	g10388	0	0	0	0	1	1	no
XLOC_009071	g10387	0	0	0	0	1	1	no
XLOC_009072	g10389	5,64897	5,20999	-0,116709	-0,144219	0,80415	0,90945	no
XLOC_009073	g4144	0	0,0200116	inf	0	1	1	no
XLOC_009074	g10390	15,132	6,97569	-1,11719	-1,46701	0,0122	0,0769454	no
XLOC_009075	g10391	38,6639	53,8449	0,477822	0,791385	0,17755	0,424426	no
XLOC_009076	g10393	19,6028	21,688	0,145837	0,215175	0,70695	0,860848	no
XLOC_009077	g10392	18,9045	18,6871	-0,0166872	-0,0278781	0,9605	0,982256	no
XLOC_009078	g4145	21,0132	34,0288	0,695463	1,06156	0,05695	0,213944	no
XLOC_009079	g10394	5,28312	6,79808	0,363738	0,421715	0,4665	0,706622	no
XLOC_009080	g4146	34,6415	35,763	0,0459643	0,0783556	0,89155	0,951426	no
XLOC_009081	g10395	45,2781	49,4277	0,126507	0,190288	0,7357	0,874871	no
XLOC_009082	g10400	117,003	161,898	0,468545	0,687108	0,21365	0,469361	no
XLOC_009083	g10401	367,709	457,74	0,315963	0,407208	0,47805	0,713917	no
XLOC_009084	g10403	29,5194	22,6947	-0,379312	-0,594325	0,3013	0,563145	no
XLOC_009085	g10396	1,42431	0,429001	-1,73122	-1,47975	0,02025	0,109865	no
XLOC_009086	g10397	44,1397	38,3724	-0,202009	-0,347622	0,54145	0,759787	no
XLOC_009087	g10398	77,8414	84,7767	0,12313	0,193724	0,7302	0,871801	no
XLOC_009088	g10399	63,0425	74,493	0,24078	0,415054	0,46935	0,707556	no
XLOC_009089	g10402	29,743	27,9251	-0,0909885	-0,148607	0,79065	0,903527	no
XLOC_009090	g10404	22,5117	13,2287	-0,767004	-1,15763	0,0394	0,168294	no
XLOC_009091	g10405	0	0	0	0	1	1	no
XLOC_009092	g4147	18,6576	55,8691	1,58229	2,28499	0,0001	0,00209829	yes
XLOC_009093	g4148	265,135	197,669	-0,42364	-0,716936	0,2138	0,469375	no
XLOC_009094	g10406	573,504	317,171	-0,854545	-1,09112	0,05795	0,215475	no
XLOC_009095	g10407	0	0	0	0	1	1	no
XLOC_009096	g10409	73,4511	85,299	0,215745	0,344679	0,5538	0,767365	no
XLOC_009097	g10408	58,4185	71,2214	0,285884	0,495257	0,3934	0,64816	no
XLOC_009098	g10410	4,50616	2,50269	-0,848419	-0,866078	0,1305	0,357801	no
XLOC_009099	g10411	0	0	0	0	1	1	no
XLOC_009100	g4149	60,5519	44,6834	-0,438432	-0,618579	0,2583	0,51859	no
XLOC_009101	g4150	1,48014	1,83079	0,30674	0,229074	0,73105	0,872604	no
XLOC_009102	g10412	0	0	0	0	1	1	no
XLOC_009103	g10413	0	28,1466	inf	#NAME?	0,00035	0,00552572	yes
XLOC_009104	g10414	1,74487	0,651	-1,42239	-1,22084	0,05635	0,212584	no
XLOC_009105	g4151	159,322	246,698	0,630802	0,994395	0,08715	0,278857	no
XLOC_009106	g10415	14,8775	12,7116	-0,22698	-0,298087	0,60455	0,79937	no
XLOC_009107	g10416	0	0	0	0	1	1	no

XLOC_009108	g4152	1106,4	836,152	-0,404035	-0,626376	0,25355	0,512845	no
XLOC_009109	g4153	0	0,049907	inf	0	1	1	no
XLOC_009110	g4154	3,08708	8,78263	1,50841	1,69584	0,00395	0,0345713	yes
XLOC_009111	g10417	29,0229	16,908	-0,779492	-1,05207	0,0614	0,223397	no
XLOC_009112	g10418	0	0	0	0	1	1	no
XLOC_009113	g4155	0	0	0	0	1	1	no
XLOC_009114	g4156	0	0	0	0	1	1	no
XLOC_009115	g4157	0,852962	0,549712	-0,633805	0	1	1	no
XLOC_009116	g10419	9,47419	12,6274	0,414486	0,474552	0,43825	0,684744	no
XLOC_009117	g10420	77,0948	109,615	0,507745	0,792257	0,1699	0,413692	no
XLOC_009118	g10421	83,2541	71,1934	-0,225778	-0,369026	0,4993	0,728873	no
XLOC_009119	g10424	60,0078	62,4322	0,0571388	0,0989164	0,85685	0,935212	no
XLOC_009120	g10422	418,678	577,804	0,464739	0,680265	0,22535	0,483492	no
XLOC_009121	g10423	26,6205	48,0518	0,852051	1,41939	0,011	0,0711126	no
XLOC_009122	g10425	69,27	64,82	-0,0957903	-0,161043	0,7714	0,893401	no
XLOC_009123	g10427	861,573	928,877	0,108515	0,154719	0,7835	0,900301	no
XLOC_009124	g10426	1351,84	867,054	-0,640732	-0,894534	0,08755	0,279773	no
XLOC_009125	g10428	0	0	0	0	1	1	no
XLOC_009126	g10429	15,304	19,6075	0,357499	0,419509	0,45405	0,698757	no
XLOC_009127	g10430	13,4496	11,7005	-0,200995	-0,291149	0,60815	0,80183	no
XLOC_009128	g10431	14,8701	14,1363	-0,0730063	-0,10688	0,8558	0,934827	no
XLOC_009129	g4158	0,528208	0	#NAME?	0	1	1	no
XLOC_009130	g10432	0	0	0	0	1	1	no
XLOC_009131	g10433	20,4757	7,73721	-1,40403	-1,61917	0,01035	0,068167	no
XLOC_009132	g10434	1,78779	3,09631	0,792372	0,797528	0,1702	0,41403	no
XLOC_009133	g10435	3,87578	8,64943	1,15812	1,02833	0,079	0,262088	no
XLOC_009134	g4159	27,2137	22,5012	-0,274331	-0,4475	0,4211	0,671517	no
XLOC_009135	g4160	8,32156	4,05455	-1,03731	-1,08366	0,06915	0,24097	no
XLOC_009136	g4161	0	0	0	0	1	1	no
XLOC_009137	g10436	143,98	112,022	-0,362087	-0,445627	0,4321	0,679242	no
XLOC_009138	g10437	43,5637	55,9391	0,360732	0,562179	0,3296	0,590615	no
XLOC_009139	g4162	53,0255	58,8291	0,149843	0,259218	0,65245	0,8295	no
XLOC_009140	g696	151,147	98,1737	-0,622541	-0,891779	0,1048	0,313188	no
XLOC_009141	g697	48,8077	55,2284	0,178303	0,30654	0,5915	0,790276	no
XLOC_009142	g699	23,3539	53,5888	1,19827	2,00814	0,00055	0,00788467	yes
XLOC_009143	g700	72,475	64,4567	-0,169153	-0,269544	0,65045	0,828137	no
XLOC_009144	g702	24,3966	12,8094	-0,929477	-1,43096	0,01375	0,0835551	no
XLOC_009145	g703	24,2652	23,7398	-0,031579	-0,0528395	0,92855	0,968287	no
XLOC_009146	g704	24,9313	21,7905	-0,19426	-0,333263	0,5515	0,766124	no
XLOC_009147	g707	832,324	1205,42	0,534317	0,83978	0,13715	0,369327	no
XLOC_009148	g708	41,7613	37,0161	-0,174014	-0,298314	0,5995	0,795859	no
XLOC_009149	g710	447,886	451,57	0,0118205	0,0185273	0,9739	0,987624	no
XLOC_009150	g711	8,19762	7,29276	-0,16874	-0,251721	0,6584	0,832431	no
XLOC_009151	g694	90,5244	126,89	0,487205	0,780461	0,1541	0,391935	no
XLOC_009152	g695	516,621	156,525	-1,72272	-2,23354	0,00085	0,011085	yes
XLOC_009153	g698	18,4348	12,0275	-0,616096	-0,904188	0,10765	0,319276	no
XLOC_009154	g701	18,6955	28,0656	0,586108	0,89194	0,12375	0,347009	no
XLOC_009155	g705	100,853	121,753	0,27171	0,362669	0,52585	0,747625	no
XLOC_009156	g706	48,8077	48,3792	-0,0127237	-0,0214422	0,96945	0,986579	no
XLOC_009157	g709	61,1142	70,5158	0,20644	0,327097	0,56015	0,771227	no
XLOC_009158	g2	28,8601	34,7352	0,267325	0,449199	0,4272	0,67663	no
XLOC_009159	g3	96,2585	94,8907	-0,0206466	-0,0313453	0,95575	0,980512	no
XLOC_009160	g4	342,055	95,9024	-1,83459	-2,64515	5,00E-05	0,00120049	yes
XLOC_009161	g6	23,4636	26,4499	0,17284	0,247491	0,6627	0,835242	no
XLOC_009162	g7	24,8792	27,4476	0,141741	0,24287	0,67085	0,840274	no
XLOC_009163	g9	54,8292	45,9624	-0,25449	-0,402114	0,46715	0,707046	no
XLOC_009164	g11	51,1352	68,286	0,417271	0,656079	0,2482	0,507023	no
XLOC_009165	g14	33,8958	56,8233	0,745374	1,10799	0,05165	0,200634	no
XLOC_009166	g5	200,81	319,558	0,670248	1,13452	0,04365	0,1798	no
XLOC_009167	g8	87,3087	104,474	0,258949	0,445534	0,4263	0,675858	no
XLOC_009168	g10	97,5425	113,082	0,213265	0,330101	0,56975	0,777616	no
XLOC_009169	g12	35,4691	51,3229	0,533038	0,895181	0,11995	0,340929	no
XLOC_009170	g13	37,4247	91,2972	1,28658	2,13547	0,00025	0,00430702	yes
XLOC_009171	g4163	40,5084	45,5989	0,170779	0,290366	0,6058	0,800219	no
XLOC_009172	g4164	9,98	10,6742	0,0970215	0,136148	0,81025	0,912565	no
XLOC_009173	g4165	31,0467	24,4902	-0,342236	-0,555037	0,31975	0,580289	no
XLOC_009174	g4166	44,434	46,9309	0,0788727	0,134979	0,8136	0,915079	no
XLOC_009175	g4167	43,9044	20,9005	-1,07083	-1,68842	0,0038	0,0337091	yes
XLOC_009176	g4168	41,7424	46,7033	0,16201	0,267483	0,6445	0,824624	no
XLOC_009177	g10440	446,772	388,174	-0,202835	-0,268515	0,63345	0,816722	no
XLOC_009178	g10442	5,23613	3,14185	-0,736886	-0,677249	0,2527	0,511863	no
XLOC_009179	g10438	119,097	85,8174	-0,472792	-0,794594	0,1601	0,401272	no
XLOC_009180	g10439	121,055	211,265	0,803395	0,966062	0,0866	0,278073	no
XLOC_009181	g10441	1,36508	4,94964	1,85833	2,13222	0,00085	0,011085	yes
XLOC_009182	g4170	258,455	168,122	-0,620406	-1,02216	0,062	0,224499	no
XLOC_009183	g4171	392,261	181,89	-1,10875	-1,41514	0,01305	0,0807505	no
XLOC_009184	g4173	270,909	375,965	0,472789	0,731833	0,20055	0,452528	no
XLOC_009185	g4169	64,2365	35,5402	-0,85394	-1,3449	0,01865	0,103412	no
XLOC_009186	g4172	4,20547	6,1702	0,553052	0,726372	0,20255	0,455054	no
XLOC_009187	g4174	1,35456	1,75832	0,37638	0,324093	0,56525	0,774704	no
XLOC_009188	g10443	0	0	0	0	1	1	no
XLOC_009189	g10444	0,0635851	0,144611	1,18541	0	1	1	no
XLOC_009190	g4175	0	0	0	0	1	1	no
XLOC_009191	g4176	22,2922	6,18863	-1,84885	-2,0562	0,00085	0,011085	yes
XLOC_009192	g4177	125,103	184,828	0,563067	0,887471	0,1241	0,347197	no
XLOC_009193	g10445	5,70261	4,42588	-0,365657	-0,496321	0,38175	0,6382	no
XLOC_009194	g4179	16,1356	20,3511	0,334859	0,484819	0,3972	0,651251	no
XLOC_009195	g4181	23,4016	38,1245	0,704113	0,936665	0,11	0,32322	no
XLOC_009196	g4178	6,11988	4,25309	-0,524993	-0,566093	0,32395	0,584313	no
XLOC_009197	g4180	33,4693	14,6217	-1,19472	-1,66713	0,00455	0,0383857	yes
XLOC_009198	g4182	6,49003	12,7243	0,971287	1,57373	0,01045	0,068504	no
XLOC_009199	g10446	10,6673	11,4137	0,09758	0,137232	0,80085	0,908537	no

XLOC_009200	g10447	0	0	0	0	1	1	no
XLOC_009201	g10448	88,8601	66,3368	-0,421726	-0,588341	0,3101	0,571193	no
XLOC_009202	g4183	88,0892	57,0698	-0,626238	-0,889774	0,1099	0,323169	no
XLOC_009203	g4184	92,0103	78,8276	-0,223094	-0,339853	0,55225	0,766299	no
XLOC_009204	g4186	132,112	111,208	-0,248497	-0,424698	0,45805	0,701013	no
XLOC_009205	g4188	209,704	331,981	0,662748	1,03292	0,0604	0,221069	no
XLOC_009206	g4191	141,737	135,32	-0,0668409	-0,116071	0,8391	0,92642	no
XLOC_009207	g4192	72,1639	89,7785	0,315093	0,52003	0,3585	0,619692	no
XLOC_009208	g4185	55,9523	78,1866	0,482725	0,591768	0,29025	0,552994	no
XLOC_009209	g4187	44,5282	44,0473	-0,0156667	-0,0270313	0,9628	0,983635	no
XLOC_009210	g4189	405,557	631,67	0,639265	0,698754	0,21215	0,467635	no
XLOC_009211	g4190	110,095	121,323	0,140095	0,234201	0,68525	0,848738	no
XLOC_009212	g4193	143,748	112,282	-0,356414	-0,576684	0,30705	0,568054	no
XLOC_009213	g10450	16,3091	14,1956	-0,200234	-0,318221	0,56825	0,776676	no
XLOC_009214	g10449	19,6115	20,9419	0,0946944	0,12914	0,81615	0,916533	no
XLOC_009215	g10451	89,7529	70,0616	-0,357334	-0,59297	0,28465	0,546579	no
XLOC_009216	g10452	0	0	0	0	1	1	no
XLOC_009217	g4194	24,4686	55,968	1,19367	1,90661	0,00125	0,0146655	yes
XLOC_009218	g4196	155,728	128,535	-0,276862	-0,429183	0,45275	0,697522	no
XLOC_009219	g4198	131,247	128,639	-0,0289569	-0,045216	0,93435	0,971293	no
XLOC_009220	g4199	78,8374	64,5084	-0,289393	-0,499805	0,38425	0,640475	no
XLOC_009221	g4201	104,07	175,373	0,752867	1,21598	0,03285	0,149971	no
XLOC_009222	g4203	59,8734	61,8026	0,0457521	0,0791598	0,89295	0,951989	no
XLOC_009223	g4205	261,066	92,1675	-1,50208	-2,36059	5,00E-05	0,00120049	yes
XLOC_009224	g4195	96,7034	326,34	1,75474	2,8	5,00E-05	0,00120049	yes
XLOC_009225	g4197	181,785	241,111	0,407464	0,695581	0,2299	0,487612	no
XLOC_009226	g4200	83,5638	48,4424	-0,786607	-1,29597	0,02665	0,132107	no
XLOC_009227	g4202	46,2647	84,5815	0,87043	1,30929	0,02135	0,114317	no
XLOC_009228	g4204	71,1521	32,4139	-1,13429	-1,83106	0,0015	0,0168343	yes
XLOC_009229	g4206	86,9239	90,7662	0,0624013	0,0888127	0,8741	0,94326	no
XLOC_009230	g4207	50,8473	40,1799	-0,339697	-0,5724	0,31665	0,577009	no
XLOC_009231	g10453	36,0045	26,107	-0,463743	-0,731737	0,1902	0,440199	no
XLOC_009232	g10454	25,7054	28,4461	0,146157	0,240587	0,66775	0,83795	no
XLOC_009233	g10455	12,366	6,06388	-1,02806	-1,20013	0,03905	0,167235	no
XLOC_009234	g10457	74,4201	78,4943	0,0768953	0,132399	0,8162	0,916533	no
XLOC_009235	g10456	34,9508	72,4713	1,05208	1,76861	0,0025	0,0247231	yes
XLOC_009236	g10458	45,7775	46,9258	0,0357443	0,0570205	0,92045	0,96497	no
XLOC_009237	g10459	5,14308	7,47144	0,538752	0,543692	0,4172	0,669319	no
XLOC_009238	g10460	16,5352	16,4709	-0,00562749	-0,00831259	0,9877	0,993619	no
XLOC_009239	g10461	164,936	68,2344	-1,27333	-2,09043	0,0003	0,00496796	yes
XLOC_009240	g10462	258,236	359,016	0,475355	0,785567	0,1715	0,416349	no
XLOC_009241	g4208	17,6487	8,95778	-0,978349	-1,36414	0,02215	0,117131	no
XLOC_009242	g10463	8,89682	12,8643	0,532014	0,792334	0,1643	0,406405	no
XLOC_009243	g10464	20,2499	33,5892	0,730086	0,626734	0,29275	0,555278	no
XLOC_009244	g10465	13,656	10,7077	-0,350883	-0,45626	0,41865	0,670084	no
XLOC_009245	g10467	148,819	122,098	-0,28552	-0,480314	0,39445	0,648995	no
XLOC_009246	g10466	170,548	119,546	-0,512615	-0,759055	0,18385	0,431916	no
XLOC_009247	g10468	9,87392	19,7168	0,997728	1,11416	0,06215	0,224793	no
XLOC_009248	g4209	8,72793	25,6276	1,55399	1,97758	0,00055	0,00788467	yes
XLOC_009249	g10469	27,1492	24,855	-0,127375	-0,182308	0,73695	0,87578	no
XLOC_009250	g10470	258,141	120,336	-1,10109	-1,56765	0,0067	0,0503783	no
XLOC_009251	g10471	29,7743	28,414	-0,0674657	-0,109419	0,84635	0,929972	no
XLOC_009252	g4210	179,836	62,4619	-1,52564	-2,0852	0,0011	0,0134856	yes
XLOC_009253	g10472	30,5265	21,2843	-0,52027	-0,685346	0,2383	0,496311	no
XLOC_009254	g10473	0	0	0	0	1	1	no
XLOC_009255	g4212	40,9567	14,6476	-1,48343	-2,38411	5,00E-05	0,00120049	yes
XLOC_009256	g4211	81,6003	71,813	-0,184329	-0,312883	0,5775	0,781096	no
XLOC_009257	g4213	57,6454	59,0943	0,0358126	0,0613764	0,9136	0,963233	no
XLOC_009258	g4216	194,323	149,432	-0,378971	-0,605079	0,29795	0,559975	no
XLOC_009259	g4218	1327,87	966,189	-0,458737	-0,518996	0,3712	0,629674	no
XLOC_009260	g4214	4,27527	5,09518	0,253118	0,242104	0,67645	0,843308	no
XLOC_009261	g4215	38,3504	44,3138	0,208515	0,357924	0,52785	0,74938	no
XLOC_009262	g4217	275,463	228,832	-0,267565	-0,423288	0,45705	0,701012	no
XLOC_009263	g10474	15,9673	10,2437	-0,640386	-0,753121	0,18835	0,43736	no
XLOC_009264	g10475	335,114	303,978	-0,140684	-0,190758	0,7398	0,876968	no
XLOC_009265	g10476	0	0	0	0	1	1	no
XLOC_009266	g10477	0	0	0	0	1	1	no
XLOC_009267	g10478	0	0	0	0	1	1	no
XLOC_009268	g10479	0	0	0	0	1	1	no
XLOC_009269	g10480	8,29106	10,5519	0,347877	0,383796	0,504	0,732142	no
XLOC_009270	g4219	501,39	209,309	-1,2603	-1,96402	0,0008	0,0106019	yes
XLOC_009271	g4220	46,5758	40,5487	-0,199924	-0,328064	0,55245	0,76636	no
XLOC_009272	g10481	68,1586	0	#NAME?	#NAME?	0,01025	0,0677355	no
XLOC_009273	g10482	0	0	0	0	1	1	no
XLOC_009274	g10483	0	0	0	0	1	1	no
XLOC_009275	g10484	0	0	0	0	1	1	no
XLOC_009276	g4221	0,0618198	0	#NAME?	0	1	1	no
XLOC_009277	g10485	0	0	0	0	1	1	no
XLOC_009278	g10486	58,6529	49,6043	-0,241736	-0,375312	0,50545	0,733345	no
XLOC_009279	g712	1,51949	2,64039	0,79717	0,854487	0,13155	0,359839	no
XLOC_009280	g713	16,5113	22,4228	0,441514	0,712852	0,2254	0,483492	no
XLOC_009281	g4222	119,74	192,757	0,686881	1,14705	0,04135	0,173751	no
XLOC_009282	g10487	0	0	0	0	1	1	no
XLOC_009283	g10488	0	0	0	0	1	1	no
XLOC_009284	g4223	593,06	582,306	-0,0264001	-0,044066	0,93925	0,972834	no
XLOC_009285	g10489	0	0	0	0	1	1	no
XLOC_009286	g10490	10,0832	17,858	0,824619	1,2318	0,0289	0,138101	no
XLOC_009287	g10491	0,108775	0,427399	1,97424	0	1	1	no
XLOC_009288	g10492	0	0	0	0	1	1	no
XLOC_009289	g4224	2,93097	2,14265	-0,451984	-0,411924	0,4765	0,712937	no
XLOC_009290	g4225	0	0	0	0	1	1	no
XLOC_009291	g10493	0	0	0	0	1	1	no

XLOC_009292	g10494	457,531	800,061	0,80624	1,20902	0,0348	0,155688	no
XLOC_009293	g4227	13,388	17,3378	0,372984	0,545112	0,3404	0,600562	no
XLOC_009294	g4228	18,8883	16,4403	-0,200258	-0,264734	0,6496	0,827591	no
XLOC_009295	g4230	43,7243	66,5659	0,60635	0,936664	0,09885	0,301743	no
XLOC_009296	g4234	38,5133	42,9683	0,157915	0,269838	0,642	0,822712	no
XLOC_009297	g4226	128,033	104,714	-0,29006	-0,483181	0,40655	0,659969	no
XLOC_009298	g4229	71,641	13,2188	-2,4382	-3,66244	5,00E-05	0,00120049	yes
XLOC_009299	g4231	44,8507	94,1721	1,07017	1,55982	0,006	0,0465771	yes
XLOC_009300	g4232	41,7476	34,5853	-0,271535	-0,435602	0,4425	0,688832	no
XLOC_009301	g4233	198,906	209,83	0,0771342	0,130261	0,8217	0,918997	no
XLOC_009302	g4235	82,6755	54,9496	-0,589351	-0,985884	0,08565	0,27649	no
XLOC_009303	g10495	1504,74	937,299	-0,682937	-0,92554	0,106	0,315622	no
XLOC_009304	g10496	406,994	485,431	0,254259	0,326673	0,56835	0,776676	no
XLOC_009305	g10497	21,9997	47,2485	1,10279	1,71619	0,0038	0,0337091	yes
XLOC_009306	g10498	0	0	0	0	1	1	no
XLOC_009307	g4236	37,1968	39,2866	0,0788584	0,117896	0,8348	0,924522	no
XLOC_009308	g4237	43,2786	29,3911	-0,558272	-0,834957	0,1451	0,381188	no
XLOC_009309	g10499	0	0	0	0	1	1	no
XLOC_009310	g10500	93,7332	115,037	0,295461	0,486712	0,407	0,660291	no
XLOC_009311	g10501	0	0	0	0	1	1	no
XLOC_009312	g10502	0	0,00957733	inf	0	1	1	no
XLOC_009313	g10503	23,7254	18,5132	-0,357872	-0,539685	0,3462	0,606524	no
XLOC_009314	g715	2,22965	1,27486	-0,806483	-0,597297	0,3011	0,563145	no
XLOC_009315	g717	3,94739	1,67552	-1,23629	-1,26959	0,0591	0,218099	no
XLOC_009316	g718	93,5068	69,1946	-0,434412	-0,686369	0,22545	0,483494	no
XLOC_009317	g720	109,392	83,4775	-0,390044	-0,653053	0,24875	0,507526	no
XLOC_009318	g714	0,820745	0,543095	-0,595729	0	1	1	no
XLOC_009319	g716	36,7095	46,2039	0,331861	0,499721	0,36845	0,628593	no
XLOC_009320	g719	80,9666	92,8899	0,198194	0,332544	0,5643	0,773942	no
XLOC_009321	g721	760,79	559,58	-0,443153	-0,589112	0,31805	0,578273	no
XLOC_009322	g722	35,2872	56,4783	0,678551	1,12734	0,0525	0,203212	no
XLOC_009323	g10504	0	0	0	0	1	1	no
XLOC_009324	g4238	0	0	0	0	1	1	no
XLOC_009325	g10505	19,4671	19,6794	0,0156471	0,0192678	0,97195	0,987026	no
XLOC_009326	g10506	249,653	184,266	-0,43813	-0,605196	0,26025	0,520918	no
XLOC_009327	g10507	92,2029	100,784	0,128378	0,201578	0,7253	0,869807	no
XLOC_009328	g10508	0	0	0	0	1	1	no
XLOC_009329	g726	15,6679	19,781	0,336303	0,574248	0,30955	0,570529	no
XLOC_009330	g727	145,132	260,264	0,84261	1,38785	0,01685	0,0959229	no
XLOC_009331	g729	27,5166	24,7615	-0,152203	-0,260427	0,64	0,82122	no
XLOC_009332	g731	27,8831	21,9097	-0,347823	-0,578631	0,29915	0,561558	no
XLOC_009333	g734	81,6152	89,8338	0,13842	0,224146	0,7002	0,85752	no
XLOC_009334	g723	17,5871	20,3976	0,213884	0,265313	0,6453	0,82511	no
XLOC_009335	g724	29,678	26,4942	-0,163718	-0,280956	0,6196	0,808526	no
XLOC_009336	g725	2,92045	3,35398	0,19968	0,238693	0,6688	0,838776	no
XLOC_009337	g728	199,167	118,67	-0,747023	-1,26753	0,0243	0,12409	no
XLOC_009338	g730	217,8	184,577	-0,238782	-0,343521	0,54965	0,765215	no
XLOC_009339	g732	136,577	104,732	-0,383007	-0,553989	0,34475	0,604976	no
XLOC_009340	g733	197,262	282,25	0,516858	0,821992	0,18665	0,434852	no
XLOC_009341	g10509	3,09703	8,01752	1,37227	0,956839	0,1117	0,326554	no
XLOC_009342	g10510	0,553319	0	#NAME?	0	1	1	no
XLOC_009343	g10512	0,166585	0,0800579	-1,05714	0	1	1	no
XLOC_009344	g10514	7,71598	74,7764	3,27666	3,85879	5,00E-05	0,00120049	yes
XLOC_009345	g10516	7,81631	4,9617	-0,655652	-0,958421	0,0831	0,271381	no
XLOC_009346	g10517	2,99007	0,974566	-1,61735	-1,27592	0,11715	0,335495	no
XLOC_009347	g10511	0,173616	0,345761	0,993872	0	1	1	no
XLOC_009348	g10513	7,64965	9,15638	0,259384	0,368887	0,51695	0,741989	no
XLOC_009349	g10515	9,2612	12,2707	0,405943	0,504192	0,38435	0,640475	no
XLOC_009350	g10518	56,6578	43,8495	-0,369714	-0,627835	0,2616	0,521395	no
XLOC_009351	g4239	0	0	0	0	1	1	no
XLOC_009352	g4240	227,893	193,139	-0,23872	-0,378096	0,5076	0,734655	no
XLOC_009353	g4241	40,6899	26,6211	-0,612099	-1,02489	0,07485	0,252673	no
XLOC_009354	g4243	129,146	90,944	-0,505949	-0,772242	0,1748	0,420617	no
XLOC_009355	g4242	1,41712	2,80571	0,985403	0,676505	0,25855	0,518685	no
XLOC_009356	g4244	0	0	0	0	1	1	no
XLOC_009357	g735	0	0	0	0	1	1	no
XLOC_009358	g10519	24,1473	40,0351	0,729401	1,16614	0,0425	0,177068	no
XLOC_009359	g10520	0	0	0	0	1	1	no
XLOC_009360	g10521	45,6769	32,6301	-0,48526	-0,798349	0,14815	0,384876	no
XLOC_009361	g4246	70,4343	15,7342	-2,16238	-3,30654	5,00E-05	0,00120049	yes
XLOC_009362	g4249	24,2335	24,9717	0,0432925	0,063191	0,9119	0,962529	no
XLOC_009363	g4245	34,548	57,0249	0,722991	1,08074	0,0617	0,223825	no
XLOC_009364	g4247	22,7498	14,6223	-0,637681	-0,955284	0,09595	0,296019	no
XLOC_009365	g4248	105,206	63,1951	-0,73534	-1,19774	0,03345	0,151669	no
XLOC_009366	g4250	175,319	26,78	-2,71076	-3,69852	5,00E-05	0,00120049	yes
XLOC_009367	g4252	95,1068	98,7376	0,0540513	0,0823069	0,88755	0,950055	no
XLOC_009368	g4251	259,669	271,004	0,0616381	0,100232	0,8575	0,935212	no
XLOC_009369	g4253	68,8996	67,3449	-0,0329263	-0,0522375	0,92805	0,968074	no
XLOC_009370	g4254	21,644	26,6809	0,301841	0,398031	0,4933	0,72417	no
XLOC_009371	g10522	1,82317	0,81194	-1,16701	-0,857966	0,1321	0,361041	no
XLOC_009372	g10523	0	0	0	0	1	1	no
XLOC_009373	g4255	0	0	0	0	1	1	no
XLOC_009374	g4256	0,16582	19,1777	6,85367	2,32092	0,12375	0,347009	no
XLOC_009375	g10524	83,2352	116,361	0,483345	0,650835	0,27555	0,537409	no
XLOC_009376	g10525	45,0681	45,5493	0,0153228	0,0181342	0,98195	0,991184	no
XLOC_009377	g10526	0	0	0	0	1	1	no
XLOC_009378	g4257	0	0	0	0	1	1	no
XLOC_009379	g10527	15,2656	11,0698	-0,463657	-0,698461	0,23365	0,49121	no
XLOC_009380	g10528	0	0	0	0	1	1	no
XLOC_009381	g10530	109,858	78,1411	-0,491481	-0,846571	0,13805	0,370429	no
XLOC_009382	g10529	5,49326	7,31199	0,4126	0,533633	0,3594	0,619938	no
XLOC_009383	g10531	0	0	0	0	1	1	no

XLOC_009384	g10532	0	0	0	0	1	1	no
XLOC_009385	g10533	0	0	0	0	1	1	no
XLOC_009386	g4258	0	0	0	0	1	1	no
XLOC_009387	g4259	0	0	0	0	1	1	no
XLOC_009388	g4260	0	0	0	0	1	1	no
XLOC_009389	g4261	0	0	0	0	1	1	no
XLOC_009390	g4262	0	0	0	0	1	1	no
XLOC_009391	g4263	7,52001	12,4913	0,732117	1,04536	0,0734	0,250274	no
XLOC_009392	g10534	167,38	129,497	-0,370209	-0,529833	0,3632	0,6231	no
XLOC_009393	g10535	0	0	0	0	1	1	no
XLOC_009394	g4264	17,7266	15,8205	-0,164119	-0,236177	0,67735	0,844002	no
XLOC_009395	g4265	4,15893	4,41424	0,0859537	0,0782786	0,8823	0,947324	no
XLOC_009396	g10536	2,01057	6,63739	1,72301	2,76217	0,2235	0,480993	no
XLOC_009397	g4266	35,0811	40,7056	0,214534	0,341876	0,5422	0,760303	no
XLOC_009398	g4268	28,1893	23,7928	-0,244621	-0,408395	0,4773	0,713407	no
XLOC_009399	g4269	36,2126	57,2794	0,661526	1,09582	0,04595	0,185691	no
XLOC_009400	g4267	136,729	146,661	0,101165	0,168825	0,76205	0,887899	no
XLOC_009401	g4271	10,848	7,30514	-0,57044	-1,23822	0,68595	0,849222	no
XLOC_009402	g4270	0,390508	0,41873	0,100666	0	1	1	no
XLOC_009403	g4272	271,759	392,533	0,530484	0,82609	0,1437	0,379469	no
XLOC_009404	g10537	0,0545103	0	#NAME?	0	1	1	no
XLOC_009405	g4273	50,5115	48,6651	-0,053726	-0,0857084	0,87505	0,943667	no
XLOC_009406	g4276	25,9613	20,9964	-0,306225	-0,497783	0,3796	0,636665	no
XLOC_009407	g4274	81,3337	215,522	1,40591	2,24392	0,00015	0,00294012	yes
XLOC_009408	g4275	43,2439	27,166	-0,670694	-1,08134	0,053	0,204583	no
XLOC_009409	g4277	26,5952	35,3562	0,410797	0,687112	0,2325	0,490367	no
XLOC_009410	g4279	36,0736	61,2856	0,764605	1,08976	0,0605	0,221352	no
XLOC_009411	g4280	184,3	393,212	1,09325	1,86164	0,0014	0,016042	yes
XLOC_009412	g4283	58,1095	83,9843	0,531345	0,850278	0,1406	0,37407	no
XLOC_009413	g4285	18,5777	21,922	0,238813	0,375545	0,51965	0,743978	no
XLOC_009414	g4286	120,254	284,352	1,24159	2,08056	0,0002	0,00367103	yes
XLOC_009415	g4287	139,017	89,6754	-0,632477	-1,00436	0,0941	0,292703	no
XLOC_009416	g4278	37,252	51,8907	0,478158	0,75366	0,1793	0,426373	no
XLOC_009417	g4281	39,533	70,9501	0,843745	1,21371	0,02785	0,135591	no
XLOC_009418	g4282	69,6334	84,0493	0,271456	0,446929	0,42695	0,676561	no
XLOC_009419	g4284	21,1661	26,5578	0,327377	0,536483	0,3592	0,619938	no
XLOC_009420	g10538	268,418	306,021	0,18915	0,308158	0,5837	0,784861	no
XLOC_009421	g10539	29,3402	24,4743	-0,261613	-0,367574	0,51495	0,740924	no
XLOC_009422	g10540	0	0	0	0	1	1	no
XLOC_009423	g10541	54,2207	28,2287	-0,941679	-1,42016	0,02035	0,110224	no
XLOC_009424	g10542	234,571	186,696	-0,329334	-0,55389	0,33675	0,597018	no
XLOC_009425	g10543	0	0	0	0	1	1	no
XLOC_009426	g10544	18,3513	26,8229	0,547586	0,663169	0,2543	0,51341	no
XLOC_009427	g10545	267,087	155,331	-0,78196	-1,29524	0,0248	0,125793	no
XLOC_009428	g10546	28,6859	32,7866	0,192761	0,317551	0,5865	0,787238	no
XLOC_009429	g10547	0	0	0	0	1	1	no
XLOC_009430	g10548	6,25584	8,76722	0,486916	0,544289	0,36125	0,621601	no
XLOC_009431	g4288	0	0,242169	inf	0	1	1	no
XLOC_009432	g4290	0,378288	4,67232	3,62658	2,89738	0,00075	0,0100752	yes
XLOC_009433	g4289	0,201357	0	#NAME?	0	1	1	no
XLOC_009434	g10549	164,821	100,351	-0,71584	-1,01835	0,08845	0,281458	no
XLOC_009435	g4291	49,9495	24,3501	-1,03654	-1,53976	0,00675	0,0506379	no
XLOC_009436	g4293	638,624	1468,68	1,20148	1,17736	0,05735	0,21438	no
XLOC_009437	g4292	26,1969	30,0207	0,196561	0,300931	0,5975	0,794509	no
XLOC_009438	g4294	156,046	135,97	-0,198685	-0,288369	0,6049	0,79937	no
XLOC_009439	g4295	17,4303	19,1121	0,132893	0,214972	0,6956	0,855239	no
XLOC_009440	g10550	0	0	0	0	1	1	no
XLOC_009441	g4296	1,19843	0,515222	-1,21788	-0,803724	0,22055	0,477469	no
XLOC_009442	g4297	91,8767	57,9883	-0,663938	-1,1305	0,0525	0,203212	no
XLOC_009443	g4298	1,50392	1,06598	-0,496543	-0,452631	0,42045	0,671244	no
XLOC_009444	g10551	46,3908	31,5316	-0,55704	-0,940939	0,1002	0,303505	no
XLOC_009445	g10552	18,8232	73,6075	1,96734	2,70563	5,00E-05	0,00120049	yes
XLOC_009446	g10554	9,21022	8,5758	-0,102964	-0,121567	0,8241	0,919725	no
XLOC_009447	g10556	37,3703	51,2747	0,456353	0,715316	0,2139	0,469385	no
XLOC_009448	g10553	54,4587	28,7553	-0,921335	-1,43731	0,0086	0,0599801	no
XLOC_009449	g10555	6,08758	6,61259	0,119347	0,115844	0,8355	0,924776	no
XLOC_009450	g10557	14,6693	14,8757	0,0201522	0,0288008	0,95795	0,981385	no
XLOC_009451	g10558	178,284	130,075	-0,454833	-0,728844	0,212	0,467514	no
XLOC_009452	g10559	0	0	0	0	1	1	no
XLOC_009453	g10560	42,7175	37,121	-0,202594	-0,287543	0,6114	0,804495	no
XLOC_009454	g10561	7,09102	5,02575	-0,496654	-0,486047	0,40455	0,658274	no
XLOC_009455	g10562	6,69058	5,45889	-0,293525	-0,292793	0,6162	0,807415	no
XLOC_009456	g10565	25,1055	24,4411	-0,038693	-0,0577736	0,91705	0,963869	no
XLOC_009457	g10566	10,2713	5,02376	-1,03178	-1,36599	0,0152	0,0892194	no
XLOC_009458	g10563	24,1894	23,1942	-0,0606101	-0,0989341	0,8605	0,935634	no
XLOC_009459	g10564	13,0605	15,9833	0,291351	0,448071	0,4253	0,674709	no
XLOC_009460	g10567	20,2013	23,3676	0,210058	0,319934	0,56365	0,773375	no
XLOC_009461	g10568	42,389	52,503	0,30871	0,508319	0,3763	0,634489	no
XLOC_009462	g10569	0	0	0	0	1	1	no
XLOC_009463	g4299	57,4094	8,80711	-2,70455	-3,99992	5,00E-05	0,00120049	yes
XLOC_009464	g4300	4,45373	20,0205	2,16839	2,76944	0,0001	0,00209829	yes
XLOC_009465	g4302	115,603	87,8208	-0,396543	-0,685994	0,2332	0,49079	no
XLOC_009466	g4304	75,6206	66,1706	-0,192589	-0,326278	0,57065	0,777831	no
XLOC_009467	g4301	51,9133	62,779	0,274179	0,427635	0,4451	0,6915	no
XLOC_009468	g4303	167,282	100,866	-0,729843	-1,12322	0,04395	0,180506	no
XLOC_009469	g4305	22,9439	10,7624	-1,09211	-0,93973	0,35455	0,615463	no
XLOC_009470	g4308	12,2158	11,0704	-0,142031	-0,148273	0,85205	0,93347	no
XLOC_009471	g4306	2,72491	5,99938	1,1386	1,35488	0,0254	0,127912	no
XLOC_009472	g4307	38,7444	49,8831	0,364563	0,556876	0,31945	0,579959	no
XLOC_009473	g4309	818,636	1316,89	0,685838	0,878871	0,1386	0,371365	no
XLOC_009474	g4312	243,291	242,606	-0,00406831	-0,00686353	0,99	0,99501	no
XLOC_009475	g4310	29,6289	30,6871	0,050629	0,0842806	0,87875	0,945576	no

XLOC_009476	g4311	477,084	374,234	-0,350302	-0,589553	0,3	0,562214	no
XLOC_009477	g4314	29,9805	42,4786	0,502713	0,815508	0,14485	0,380837	no
XLOC_009478	g4313	111,301	146,706	0,398461	0,645695	0,2495	0,508092	no
XLOC_009479	g4315	84,3017	159,834	0,922939	1,52552	0,00825	0,0582842	no
XLOC_009480	g4316	360,881	685,36	0,925338	1,43394	0,0142	0,0854436	no
XLOC_009481	g4317	84,2341	47,3661	-0,830549	-1,33968	0,0242	0,123967	no
XLOC_009482	g4318	37,5516	16,6504	-1,17332	-1,94649	0,00055	0,00788467	yes
XLOC_009483	g10570	32,9446	31,3646	-0,0709078	-0,108176	0,8462	0,929972	no
XLOC_009484	g10571	58,3341	76,572	0,392477	0,645873	0,26525	0,526213	no
XLOC_009485	g10573	144,056	84,1925	-0,774868	-1,2956	0,02445	0,124597	no
XLOC_009486	g10575	61,9783	67,1702	0,116059	0,196907	0,72935	0,871694	no
XLOC_009487	g10577	24,5834	26,3422	0,0996942	0,167585	0,7728	0,894131	no
XLOC_009488	g10579	41,3302	75,1767	0,863091	1,36178	0,01465	0,0872426	no
XLOC_009489	g10581	92,9143	155,408	0,742089	1,26856	0,03205	0,147346	no
XLOC_009490	g10584	41,4638	54,2803	0,388575	0,629972	0,2691	0,530422	no
XLOC_009491	g10572	259,881	218,99	-0,246988	-0,386175	0,503	0,73123	no
XLOC_009492	g10574	72,0809	60,1371	-0,261361	-0,453443	0,432	0,679193	no
XLOC_009493	g10576	21,3116	23,8278	0,161002	0,249166	0,66385	0,835468	no
XLOC_009494	g10578	50,0499	35,3483	-0,501727	-0,820469	0,14925	0,386813	no
XLOC_009495	g10580	168,494	135,011	-0,319626	-0,540689	0,3453	0,605488	no
XLOC_009496	g10582	129,83	75,6906	-0,77844	-1,28138	0,0256	0,128392	no
XLOC_009497	g10583	41,8228	24,3133	-0,782546	-1,28695	0,01945	0,106823	no
XLOC_009498	g10585	189,842	182,104	-0,0600357	-0,100848	0,86355	0,936821	no
XLOC_009499	g10586	80,5868	5,33124	-3,918	-4,98453	5,00E-05	0,00120049	yes
XLOC_009500	g10587	4,8303	2,02502	-1,25418	-1,16943	0,05645	0,212798	no
XLOC_009501	g10588	59,796	42,8073	-0,482191	-0,785299	0,1673	0,410619	no
XLOC_009502	g10589	49,7665	66,6717	0,421898	0,72796	0,2025	0,455046	no
XLOC_009503	g10590	25,2788	8,21245	-1,62205	-2,44131	5,00E-05	0,00120049	yes
XLOC_009504	g10591	18,1531	36,6304	1,01283	1,17001	0,05045	0,19793	no
XLOC_009505	g10592	48,6232	48,1655	-0,013645	-0,0216486	0,9701	0,986679	no
XLOC_009506	g10593	20,3946	16,7177	-0,286813	-0,331309	0,55405	0,767387	no
XLOC_009507	g10594	16,8975	13,3295	-0,342184	-0,430766	0,4473	0,693445	no
XLOC_009508	g4319	45,4226	38,8705	-0,224736	-0,376533	0,50085	0,730196	no
XLOC_009509	g736	7,63349	14,92	0,966837	1,05324	0,07885	0,261767	no
XLOC_009510	g737	73,0296	39,5997	-0,882992	-1,4793	0,0097	0,0650198	no
XLOC_009511	g10595	17,2541	15,6435	-0,141378	-0,149304	0,80255	0,908624	no
XLOC_009512	g4320	25,5779	25,8114	0,0131108	0,0190499	0,9745	0,987624	no
XLOC_009513	g4321	45,3457	0	#NAME?	0	1	1	no
XLOC_009514	g4323	57,1715	154,195	1,43139	2,09916	0,0003	0,00496796	yes
XLOC_009515	g4322	8,89238	5,07178	-0,810077	-1,00367	0,0825	0,270501	no
XLOC_009516	g4324	48,6928	74,9445	0,622114	1,06615	0,0616	0,22371	no
XLOC_009517	g4325	42,8155	42,447	-0,0124703	-0,0205889	0,971	0,986977	no
XLOC_009518	g10596	2,0618	1,10404	-0,901113	-0,970242	0,08595	0,276731	no
XLOC_009519	g10597	93,503	90,0665	-0,054023	-0,0849301	0,88425	0,948388	no
XLOC_009520	g4327	0	0	0	0	1	1	no
XLOC_009521	g4328	0	0	0	0	1	1	no
XLOC_009522	g4326	0	0	0	0	1	1	no
XLOC_009523	g4329	46,0116	48,3505	0,0715332	0,121315	0,8288	0,921619	no
XLOC_009524	g4330	6,61179	30,7075	2,21548	2,68711	5,00E-05	0,00120049	yes
XLOC_009525	g10598	117,445	97,6974	-0,265596	-0,459111	0,4238	0,67309	no
XLOC_009526	g10600	107,157	149,842	0,483716	0,796904	0,17935	0,426373	no
XLOC_009527	g10599	47,9457	71,6081	0,578722	0,956517	0,08665	0,278073	no
XLOC_009528	g4331	0	0,635316	inf	0	1	1	no
XLOC_009529	g4332	271,352	90,8185	-1,57911	-2,3503	0,00025	0,00430702	yes
XLOC_009530	g4333	14,6098	12,8148	-0,189126	-0,199949	0,7259	0,869807	no
XLOC_009531	g4334	36,9132	21,8392	-0,75722	-1,25467	0,02625	0,130651	no
XLOC_009532	g4335	23,172	8,039	-1,52729	-2,08327	0,0007	0,00954722	yes
XLOC_009533	g10601	0	0	0	0	1	1	no
XLOC_009534	g10602	0	0	0	0	1	1	no
XLOC_009535	g10603	0	0	0	0	1	1	no
XLOC_009536	g10604	0	0	0	0	1	1	no
XLOC_009537	g10605	16,1409	37,7183	1,22454	0,738191	0,21215	0,467635	no
XLOC_009538	g10606	58,433	54,1137	-0,110789	-0,179796	0,75605	0,884596	no
XLOC_009539	g10608	28,795	31,6508	0,136423	0,185482	0,74595	0,880495	no
XLOC_009540	g10610	36,5097	39,2946	0,106052	0,177348	0,7527	0,883518	no
XLOC_009541	g10612	40,085	55,5508	0,470746	0,805955	0,1542	0,392088	no
XLOC_009542	g10613	31,5338	68,1514	1,11184	1,73157	0,0034	0,0310297	yes
XLOC_009543	g10616	51,4831	159,74	1,63355	2,64419	5,00E-05	0,00120049	yes
XLOC_009544	g10607	19,819	23,4518	0,242815	0,417095	0,46735	0,707046	no
XLOC_009545	g10609	43,6519	96,6405	1,14658	1,89925	0,00095	0,0120686	yes
XLOC_009546	g10611	55,6681	61,8203	0,151229	0,255358	0,65505	0,831084	no
XLOC_009547	g10614	226,311	180,877	-0,323297	-0,550985	0,3264	0,587257	no
XLOC_009548	g10615	22,1877	24,0445	0,115949	0,163159	0,774	0,894619	no
XLOC_009549	g10617	10,19	13,228	0,376448	0,622447	0,2628	0,523149	no
XLOC_009550	g4336	4,99225	7,44411	0,576411	0,759435	0,2006	0,452537	no
XLOC_009551	g10618	27,7181	35,0688	0,339363	0,48306	0,4062	0,659809	no
XLOC_009552	g10619	2,91707	1,6644	-0,809517	-0,827035	0,17945	0,426373	no
XLOC_009553	g10620	111,397	115,293	0,0495922	0,0820475	0,88635	0,949281	no
XLOC_009554	g10621	18,9248	26,5392	0,487848	0,648575	0,25785	0,517915	no
XLOC_009555	g10623	36,1789	45,0984	0,317928	0,526277	0,3602	0,620859	no
XLOC_009556	g10622	207,885	469,88	1,17651	1,9147	0,0014	0,016042	yes
XLOC_009557	g4337	0,79338	0,238019	-1,73693	0	1	1	no
XLOC_009558	g4338	131,991	177,555	0,427832	0,593703	0,29505	0,557058	no
XLOC_009559	g4341	155,903	159,923	0,0367286	0,0555771	0,9227	0,966188	no
XLOC_009560	g4343	9,4428	7,6284	-0,307835	-0,365839	0,5143	0,74064	no
XLOC_009561	g4345	47,3461	33,3924	-0,503725	-0,875977	0,1338	0,363462	no
XLOC_009562	g4339	10,2847	4,40908	-1,22194	-1,48375	0,01315	0,0811137	no
XLOC_009563	g4340	34,1578	22,2144	-0,620722	-0,898384	0,11355	0,330193	no
XLOC_009564	g4342	59,4633	59,5616	0,00238369	0,00375647	0,9949	0,997337	no
XLOC_009565	g4344	36,3653	51,0901	0,490481	0,824392	0,1498	0,387142	no
XLOC_009566	g4346	71,6491	73,2893	0,0326558	0,0555911	0,92355	0,966357	no
XLOC_009567	g4347	152,08	157,377	0,0494002	0,0779506	0,8884	0,950336	no

XLOC_009568	g4348	0	0	0	0	1	1	no
XLOC_009569	g10624	48,2225	276,18	2,51783	2,60695	5,00E-05	0,00120049	yes
XLOC_009570	g10625	86,0055	58,5781	-0,554069	-0,750854	0,19465	0,445978	no
XLOC_009571	g10626	32,7186	35,6714	0,124656	0,165465	0,77475	0,894748	no
XLOC_009572	g10627	23,0609	23,4925	0,026752	0,033757	0,9521	0,978762	no
XLOC_009573	g4350	27,3483	23,1823	-0,238427	-0,353059	0,5267	0,748509	no
XLOC_009574	g4349	53,1678	27,7878	-0,9361	-1,11239	0,0607	0,221589	no
XLOC_009575	g4351	48,847	91,6089	0,907216	1,3969	0,012	0,0759278	no
XLOC_009576	g10628	0	0	0	0	1	1	no
XLOC_009577	g4353	36,5184	27,4702	-0,410756	-0,68167	0,21365	0,469361	no
XLOC_009578	g4352	16,7047	25,115	0,588294	0,917458	0,10585	0,315366	no
XLOC_009579	g4355	4,26924	2,56461	-0,73524	-0,850548	0,14315	0,378394	no
XLOC_009580	g4354	3,10026	2,10308	-0,559887	-0,541859	0,34505	0,605394	no
XLOC_009581	g10629	13,4887	25,156	0,899148	1,22996	0,03405	0,153311	no
XLOC_009582	g10630	14,1001	31,7798	1,1724	1,76961	0,00175	0,0188639	yes
XLOC_009583	g10631	0	0	0	0	1	1	no
XLOC_009584	g4357	112,13	23,837	-2,2339	-3,13848	5,00E-05	0,00120049	yes
XLOC_009585	g4358	53,7155	54,2749	0,0149455	0,0217317	0,9714	0,986977	no
XLOC_009586	g4356	101,207	122,791	0,278899	0,455791	0,42985	0,678093	no
XLOC_009587	g4359	29,654	25,6324	-0,210262	-0,344221	0,5434	0,761117	no
XLOC_009588	g738	18,2022	16,9759	-0,100623	-0,143901	0,8082	0,911624	no
XLOC_009589	g10632	118,805	40,4465	-1,55451	-2,2588	0,00015	0,00294012	yes
XLOC_009590	g10633	44,6221	43,4228	-0,0393063	-0,0612532	0,91215	0,962531	no
XLOC_009591	g10637	87,2785	101,183	0,213276	0,367853	0,51475	0,740817	no
XLOC_009592	g10639	32,8822	37,7215	0,198082	0,322876	0,56935	0,777354	no
XLOC_009593	g10640	59,9455	92,3542	0,623525	1,01315	0,07795	0,259922	no
XLOC_009594	g10643	44,1689	46,0981	0,0616772	0,104837	0,8517	0,933294	no
XLOC_009595	g10646	249,823	248,885	-0,00543023	-0,00933354	0,98725	0,993619	no
XLOC_009596	g10647	19,0003	18,0127	-0,0770096	-0,104278	0,85825	0,935373	no
XLOC_009597	g10634	71,2437	85,7375	0,267163	0,404214	0,47555	0,712433	no
XLOC_009598	g10635	59,9063	70,3544	0,231932	0,401759	0,4897	0,722376	no
XLOC_009599	g10636	27,6275	32,4767	0,2333	0,397672	0,4806	0,715629	no
XLOC_009600	g10638	127,067	166,621	0,390972	0,640546	0,26395	0,524581	no
XLOC_009601	g10641	16,0895	9,02153	-0,834671	-1,31304	0,01985	0,108293	no
XLOC_009602	g10642	89,1301	81,1565	-0,135204	-0,197474	0,7242	0,86956	no
XLOC_009603	g10644	84,795	93,8993	0,147136	0,238043	0,6815	0,846595	no
XLOC_009604	g10645	63,826	56,6336	-0,172486	-0,278275	0,62145	0,809691	no
XLOC_009605	g4360	0,550994	0,259047	-1,08882	0	1	1	no
XLOC_009606	g10648	89,4821	81,1314	-0,14134	-0,217729	0,70285	0,859051	no
XLOC_009607	g10649	12,7654	23,8126	0,899484	1,1518	0,0438	0,18004	no
XLOC_009608	g10650	0	0	0	0	1	1	no
XLOC_009609	g4362	244,759	374,72	0,614451	0,938801	0,0967	0,297492	no
XLOC_009610	g4363	64,4762	72,5572	0,170351	0,280526	0,61775	0,808291	no
XLOC_009611	g4364	47,0839	86,6359	0,879731	1,49421	0,00825	0,0582842	no
XLOC_009612	g4361	46,8607	28,8023	-0,702196	-0,987368	0,08795	0,280412	no
XLOC_009613	g4365	143,404	96,1831	-0,576227	-0,888751	0,1116	0,326359	no
XLOC_009614	g10651	0	0	0	0	1	1	no
XLOC_009615	g10652	7,79212	3,46005	-1,17122	-1,4046	0,02405	0,123779	no
XLOC_009616	g10653	16,8822	10,7542	-0,650594	-0,773542	0,16815	0,410959	no
XLOC_009617	g10654	28,5371	17,4845	-0,706763	-1,14402	0,04855	0,192786	no
XLOC_009618	g10655	0	0	0	0	1	1	no
XLOC_009619	g10656	0	0	0	0	1	1	no
XLOC_009620	g739	21,4215	31,8246	0,571085	0,842053	0,13055	0,357801	no
XLOC_009621	g4368	21,1374	21,5989	0,0311637	0,0479028	0,93265	0,970604	no
XLOC_009622	g4370	44,2605	25,4895	-0,796113	-1,30336	0,0246	0,125037	no
XLOC_009623	g4372	17,2144	13,6544	-0,334249	-0,55448	0,3421	0,602155	no
XLOC_009624	g4366	0	0	0	0	1	1	no
XLOC_009625	g4367	6,95115	1,84665	-1,91234	-1,56079	0,1267	0,351269	no
XLOC_009626	g4369	5,56054	3,37273	-0,721309	-0,765799	0,171	0,415443	no
XLOC_009627	g4371	17,1963	14,0063	-0,296024	-0,44406	0,42875	0,677386	no
XLOC_009628	g4373	24,5035	39,1759	0,676976	0,946751	0,0845	0,274382	no
XLOC_009629	g10657	66,3759	74,267	0,162062	0,15749	0,78745	0,901779	no
XLOC_009630	g10658	11,0832	4,33032	-1,35583	-1,96505	0,0011	0,0134856	yes
XLOC_009631	g4374	157,316	142,411	-0,143606	-0,217232	0,70825	0,861576	no
XLOC_009632	g4378	51,7173	45,2888	-0,191493	-0,306445	0,5973	0,794351	no
XLOC_009633	g4379	54,7622	62,706	0,195421	0,332414	0,55085	0,765793	no
XLOC_009634	g4380	64,914	74,8553	0,205574	0,339193	0,5502	0,765215	no
XLOC_009635	g4382	63,2241	107,18	0,76149	1,22852	0,02885	0,137997	no
XLOC_009636	g4375	143,23	194,179	0,439052	0,580984	0,29855	0,560566	no
XLOC_009637	g4376	101,985	91,6575	-0,154031	-0,229911	0,678	0,844383	no
XLOC_009638	g4377	111,007	125,993	0,182685	0,317638	0,58575	0,786728	no
XLOC_009639	g4381	602,619	905,2	0,58699	0,967732	0,08295	0,271381	no
XLOC_009640	g4383	26,1879	30,4819	0,219052	0,361713	0,53635	0,755406	no
XLOC_009641	g4384	320,307	278,717	-0,200654	-0,308183	0,5773	0,781041	no
XLOC_009642	g10659	0	0	0	0	1	1	no
XLOC_009643	g10660	0	0	0	0	1	1	no
XLOC_009644	g10661	33,3552	13,7044	-1,28328	-1,47989	0,0138	0,0837035	no
XLOC_009645	g10662	8,92985	27,4668	1,62098	1,83215	0,00355	0,031924	yes
XLOC_009646	g10663	0	0	0	0	1	1	no
XLOC_009647	g10664	40,6876	57,0044	0,486485	0,72335	0,19615	0,447432	no
XLOC_009648	g10665	14,3986	26,5955	0,885254	1,17302	0,0408	0,172008	no
XLOC_009649	g10667	584,981	13202	4,49622	5,71383	5,00E-05	0,00120049	yes
XLOC_009650	g10666	105,103	267,004	1,34506	1,77869	0,00245	0,0243512	yes
XLOC_009651	g10668	41,5849	39,1117	-0,0884607	-0,148802	0,7917	0,904011	no
XLOC_009652	g10669	24,2889	19,3961	-0,324534	-0,337564	0,5567	0,769863	no
XLOC_009653	g10670	0	0	0	0	1	1	no
XLOC_009654	g10671	103,956	68,4419	-0,603019	-0,950282	0,0966	0,297371	no
XLOC_009655	g10672	68,9944	61,9978	-0,154262	-0,241983	0,67035	0,839755	no
XLOC_009656	g10673	0	0	0	0	1	1	no
XLOC_009657	g4385	0	0	0	0	1	1	no
XLOC_009658	g4386	53,5405	96,4532	0,849198	1,43166	0,0133	0,0817309	no
XLOC_009659	g4387	0	0	0	0	1	1	no

XLOC_009660	g4389	8,02266	7,59784	-0,0784912	-0,123229	0,8306	0,922276	no
XLOC_009661	g4388	74,2545	69,2604	-0,100447	-0,168541	0,7612	0,887449	no
XLOC_009662	g4390	22,0667	39,979	0,857375	1,31181	0,016	0,0925869	no
XLOC_009663	g10674	0	0	0	0	1	1	no
XLOC_009664	g10675	132,996	211,002	0,665877	1,04039	0,06285	0,226491	no
XLOC_009665	g10676	0	0	0	0	1	1	no
XLOC_009666	g10677	18,0348	49,0316	1,44293	1,96762	0,00125	0,0146655	yes
XLOC_009667	g10678	5,39627	9,08108	0,750903	0,73278	0,3728	0,631165	no
XLOC_009668	g10679	17,6394	22,9926	0,382368	0,596133	0,29525	0,55714	no
XLOC_009669	g10680	9,27957	6,94528	-0,418025	-0,569473	0,3032	0,564534	no
XLOC_009670	g10681	0	0	0	0	1	1	no
XLOC_009671	g10682	0,272236	0,311881	0,19614	0	1	1	no
XLOC_009672	g68	80,3483	68,7528	-0,224849	-0,358074	0,52425	0,746323	no
XLOC_009673	g71	12,9498	8,14062	-0,669717	-0,898211	0,12095	0,342878	no
XLOC_009674	g72	48,2987	230,564	2,25511	3,33128	5,00E-05	0,00120049	yes
XLOC_009675	g73	2161,01	1457,77	-0,567946	-0,526279	0,36135	0,621664	no
XLOC_009676	g75	51,9455	46,1389	-0,171016	-0,287881	0,6174	0,808291	no
XLOC_009677	g78	9,70505	19,0201	0,970715	1,55376	0,00765	0,0553596	no
XLOC_009678	g81	186,932	132,873	-0,492465	-0,792336	0,15575	0,39328	no
XLOC_009679	g83	15,6373	18,7931	0,265207	0,335552	0,5628	0,772925	no
XLOC_009680	g85	57,215	46,4699	-0,300095	-0,494437	0,3922	0,64686	no
XLOC_009681	g86	42,876	49,7995	0,21596	0,358372	0,5301	0,750804	no
XLOC_009682	g88	54,1628	60,9802	0,171039	0,29124	0,60635	0,800532	no
XLOC_009683	g91	38,7608	50,8321	0,391143	0,622047	0,2725	0,534007	no
XLOC_009684	g69	19,7009	17,1728	-0,198142	-0,327331	0,5522	0,766299	no
XLOC_009685	g70	105,403	93,0779	-0,179401	-0,308378	0,5898	0,790006	no
XLOC_009686	g74	64,1658	64,6947	0,0118438	0,020033	0,9716	0,986977	no
XLOC_009687	g76	36,5354	31,6893	-0,2053	-0,320773	0,57875	0,782073	no
XLOC_009688	g77	1067,3	1706,71	0,677254	0,883464	0,1133	0,329709	no
XLOC_009689	g79	11,7157	8,90908	-0,395091	-0,567843	0,31495	0,57568	no
XLOC_009690	g80	20,385	23,271	0,191031	0,297555	0,60255	0,798521	no
XLOC_009691	g82	95,5643	56,0038	-0,770947	-1,23822	0,02675	0,132468	no
XLOC_009692	g84	158,189	220,06	0,476253	0,644881	0,2504	0,508884	no
XLOC_009693	g87	33,9009	26,2294	-0,370141	-0,590165	0,3003	0,562226	no
XLOC_009694	g89	141,57	109,94	-0,364805	-0,62142	0,27915	0,540885	no
XLOC_009695	g90	158,095	112,31	-0,493303	-0,756666	0,1735	0,419132	no
XLOC_009696	g10683	0	0	0	0	1	1	no
XLOC_009697	g10684	79,3451	72,8874	-0,122471	-0,187952	0,7381	0,876358	no
XLOC_009698	g10685	0	0	0	0	1	1	no
XLOC_009699	g10686	152,868	42,5886	-1,84374	-2,76634	5,00E-05	0,00120049	yes
XLOC_009700	g10687	0	0	0	0	1	1	no
XLOC_009701	g10688	0	0	0	0	1	1	no
XLOC_009702	g10689	0	0	0	0	1	1	no
XLOC_009703	g4391	62,5417	44,78	-0,481965	-0,792361	0,1688	0,41183	no
XLOC_009704	g4392	559,858	220,339	-1,34534	-1,81969	0,0018	0,0192549	yes
XLOC_009705	g4394	36,8673	51,4885	0,481907	0,76794	0,17225	0,417447	no
XLOC_009706	g4393	58,3828	49,6371	-0,234124	-0,354528	0,53635	0,755406	no
XLOC_009707	g4395	244,896	221,541	-0,144599	-0,232278	0,6798	0,845936	no
XLOC_009708	g10690	0	0	0	0	1	1	no
XLOC_009709	g10691	0	0	0	0	1	1	no
XLOC_009710	g10692	13,969	7,18617	-0,958932	-1,31212	0,02535	0,127857	no
XLOC_009711	g10693	85,6784	121,089	0,499063	0,774008	0,17455	0,420427	no
XLOC_009712	g10694	10,9257	34,1862	1,64569	2,26291	0,0003	0,00496796	yes
XLOC_009713	g10697	123,53	122,041	-0,0174898	-0,0289842	0,9597	0,981898	no
XLOC_009714	g10698	8,10613	8,19926	0,0164796	0,0225399	0,9701	0,986679	no
XLOC_009715	g10695	20,9622	24,8952	0,24808	0,388715	0,5072	0,734473	no
XLOC_009716	g10696	4,05431	7,09384	0,80711	1,18929	0,04015	0,17046	no
XLOC_009717	g10699	53,342	103,704	0,959135	1,63763	0,00325	0,0301654	yes
XLOC_009718	g10700	947,01	260,363	-1,86286	-2,73634	5,00E-05	0,00120049	yes
XLOC_009719	g4396	0	0	0	0	1	1	no
XLOC_009720	g10701	0	0	0	0	1	1	no
XLOC_009721	g10702	0	0	0	0	1	1	no
XLOC_009722	g10703	0	0	0	0	1	1	no
XLOC_009723	g10704	11,3778	70,2572	2,62642	3,32277	5,00E-05	0,00120049	yes
XLOC_009724	g4397	0,0966341	0,0879028	-0,136622	0	1	1	no
XLOC_009725	g4399	0	0	0	0	1	1	no
XLOC_009726	g4398	0	0	0	0	1	1	no
XLOC_009727	g10705	57,0439	42,627	-0,420305	-0,606	0,29285	0,555278	no
XLOC_009728	g4401	16,6039	27,4733	0,726505	1,03877	0,0706	0,244203	no
XLOC_009729	g4403	40,1508	50,2461	0,323581	0,500963	0,38075	0,637613	no
XLOC_009730	g4400	30,8301	11,6975	-1,39814	-1,97292	0,0005	0,00733931	yes
XLOC_009731	g4402	1,02529	0,539479	-0,926393	0	1	1	no
XLOC_009732	g4404	32,3886	22,0191	-0,556731	-0,82055	0,1401	0,373549	no
XLOC_009733	g4405	21,2777	27,3444	0,3619	0,606089	0,29655	0,558627	no
XLOC_009734	g10706	22,6524	17,6282	-0,361778	-0,52414	0,3597	0,620238	no
XLOC_009735	g4407	53,8255	59,9105	0,154517	0,248407	0,64685	0,825909	no
XLOC_009736	g4409	111,404	209,167	0,908848	1,43279	0,01325	0,0814746	no
XLOC_009737	g4411	17,644	21,1513	0,261568	0,412368	0,46025	0,70181	no
XLOC_009738	g4413	50,0364	40,801	-0,294375	-0,451793	0,44035	0,686714	no
XLOC_009739	g4406	31,1342	26,7954	-0,216512	-0,322039	0,5755	0,780604	no
XLOC_009740	g4408	58,515	50,4795	-0,21311	-0,301563	0,58835	0,788966	no
XLOC_009741	g4410	190,995	200,93	0,073152	0,122352	0,82735	0,921258	no
XLOC_009742	g4412	41,2378	44,4208	0,10727	0,17867	0,748	0,881846	no
XLOC_009743	g4414	154,682	180,324	0,221282	0,381215	0,50795	0,734836	no
XLOC_009744	g4415	0	0	0	0	1	1	no
XLOC_009745	g4416	43,1802	34,212	-0,335866	-0,504027	0,3736	0,63195	no
XLOC_009746	g10707	253,392	260,649	0,0407377	0,0529294	0,92665	0,967539	no
XLOC_009747	g4417	18,6896	13,5233	-0,466788	-0,691504	0,2132	0,468687	no
XLOC_009748	g4418	0	0	0	0	1	1	no
XLOC_009749	g4421	56,4381	26,0337	-1,11629	-1,64394	0,0039	0,0342252	yes
XLOC_009750	g4419	79,0043	63,718	-0,31023	-0,53064	0,3445	0,604645	no
XLOC_009751	g4420	50,0025	38,1966	-0,388556	-0,645165	0,26765	0,528625	no

XLOC_009752	g10708	0	0	0	0	1	1	no
XLOC_009753	g4422	0	0	0	0	1	1	no
XLOC_009754	g10709	0	0	0	0	1	1	no
XLOC_009755	g10710	0	0	0	0	1	1	no
XLOC_009756	g10711	5,69836	4,16718	-0,451475	-0,552816	0,33825	0,598274	no
XLOC_009757	g4423	105,84	60,7177	-0,801691	-1,1657	0,0312	0,144862	no
XLOC_009758	g10712	26,5789	19,2904	-0,4624	-0,592335	0,3006	0,562587	no
XLOC_009759	g4424	2,17574	2,82356	0,376007	0,383281	0,5071	0,734472	no
XLOC_009760	g4425	25,8001	77,6713	1,59001	1,66396	0,00825	0,0582842	no
XLOC_009761	g4426	143,957	116,531	-0,304923	-0,499527	0,3793	0,636522	no
XLOC_009762	g4427	1,07276	4,27628	1,99503	2,2671	0,00065	0,00901554	yes
XLOC_009763	g4428	1,99162	4,14796	1,05846	0,687983	0,22555	0,483497	no
XLOC_009764	g4429	0,767992	1,444	0,910905	0,734767	0,22625	0,484363	no
XLOC_009765	g4430	2,53468	0	#NAME?	#NAME?	0,0148	0,0877633	no
XLOC_009766	g4431	2,74392	7,09701	1,37097	1,78281	0,0043	0,0368143	yes
XLOC_009767	g4432	235,933	20,668	-3,51291	-3,99984	5,00E-05	0,00120049	yes
XLOC_009768	g4433	88,7168	45,3724	-0,967391	-1,55903	0,0083	0,0585532	no
XLOC_009769	g10713	0	0	0	0	1	1	no
XLOC_009770	g4434	21,8905	19,8564	-0,140705	-0,227697	0,6825	0,847104	no
XLOC_009771	g10714	57,883	88,8421	0,618104	0,976191	0,08055	0,265972	no
XLOC_009772	g10715	0	0	0	0	1	1	no
XLOC_009773	g4435	13,9255	10,403	-0,420727	-0,606146	0,2867	0,549334	no
XLOC_009774	g4436	4,88369	4,91059	0,00792525	0,0101333	0,99025	0,99501	no
XLOC_009775	g4437	38,2425	34,676	-0,141239	-0,23813	0,67985	0,845936	no
XLOC_009776	g4438	17,5409	14,5461	-0,270086	-0,427425	0,445	0,6915	no
XLOC_009777	g10716	0,0285186	0,350891	3,62105	0	1	1	no
XLOC_009778	g10717	0	0	0	0	1	1	no
XLOC_009779	g4439	905,865	916,12	0,0162405	0,0226755	0,96725	0,985566	no
XLOC_009780	g4441	119,403	80,9887	-0,560046	-0,959161	0,0888	0,282115	no
XLOC_009781	g4440	117,839	11,913	-3,3062	-4,99807	5,00E-05	0,00120049	yes
XLOC_009782	g10718	14,5208	19,2057	0,403411	0,5285	0,3522	0,612793	no
XLOC_009783	g10719	0,751501	3,8097	2,34183	2,30737	0,14475	0,380837	no
XLOC_009784	g10720	0	0,231358	inf	0	1	1	no
XLOC_009785	g4442	3,93345	4,89777	0,31633	0,423249	0,4568	0,701012	no
XLOC_009786	g10721	0	0	0	0	1	1	no
XLOC_009787	g10722	0	0	0	0	1	1	no
XLOC_009788	g10723	4,73037	8,73242	0,884427	1,14855	0,0428	0,177622	no
XLOC_009789	g10724	20,4779	12,1668	-0,751124	-0,984243	0,08305	0,271381	no
XLOC_009790	g10725	28,1119	43,6086	0,633429	1,05195	0,06155	0,223694	no
XLOC_009791	g10726	125,759	60,1132	-1,06491	-1,79844	0,00225	0,0229201	yes
XLOC_009792	g10727	31,3277	38,6043	0,301322	0,379424	0,5099	0,736139	no
XLOC_009793	g10728	0	0	0	0	1	1	no
XLOC_009794	g10729	518,676	355,567	-0,544713	-0,853218	0,1215	0,343983	no
XLOC_009795	g10730	1,91861	0,962102	-0,995797	-0,924702	0,12785	0,353161	no
XLOC_009796	g10731	1,55446	2,42106	0,639228	0,556716	0,3938	0,648519	no
XLOC_009797	g10732	8,93915	14,6834	0,715976	0,646394	0,2588	0,518762	no
XLOC_009798	g4444	97,2125	93,2788	-0,0595937	-0,102315	0,86065	0,935634	no
XLOC_009799	g4443	55,2825	37,7405	-0,55071	-0,79229	0,16375	0,405862	no
XLOC_009800	g4445	46,4071	54,0402	0,219687	0,361095	0,53175	0,75231	no
XLOC_009801	g4446	129,008	196,646	0,608145	0,697504	0,2304	0,487719	no
XLOC_009802	g10733	132,656	112,808	-0,233821	-0,396167	0,49435	0,724879	no
XLOC_009803	g10735	35,1708	64,3341	0,871204	1,32258	0,0241	0,123907	no
XLOC_009804	g10734	13,3629	10,2124	-0,387907	-0,525756	0,3567	0,617124	no
XLOC_009805	g10736	0	0	0	0	1	1	no
XLOC_009806	g10737	62,2405	71,4103	0,198279	0,344555	0,55135	0,766024	no
XLOC_009807	g10738	49,3197	36,9861	-0,415179	-0,583803	0,31315	0,573795	no
XLOC_009808	g10739	314,732	243,654	-0,369293	-0,613059	0,27485	0,53647	no
XLOC_009809	g4448	45,0758	29,8173	-0,596206	-0,967932	0,0843	0,273843	no
XLOC_009810	g4447	23,6576	18,6065	-0,346495	-0,518938	0,36925	0,628841	no
XLOC_009811	g4449	9,47578	24,9129	1,39457	2,09072	0,00035	0,00552572	yes
XLOC_009812	g10740	14,9483	11,6771	-0,356301	-0,417103	0,45875	0,701036	no
XLOC_009813	g10741	0	0	0	0	1	1	no
XLOC_009814	g10742	0	0	0	0	1	1	no
XLOC_009815	g10744	15,9428	10,683	-0,577592	-0,913545	0,1081	0,319742	no
XLOC_009816	g10747	107,222	97,8306	-0,132246	-0,223003	0,6977	0,856213	no
XLOC_009817	g10743	2,97311	2,77302	-0,100511	0	1	1	no
XLOC_009818	g10745	32,2781	27,005	-0,257328	-0,425803	0,4577	0,701013	no
XLOC_009819	g10746	2,91702	2,99243	0,0368216	0,0400322	0,94375	0,975334	no
XLOC_009820	g10748	25,3103	38,3388	0,599081	0,65158	0,26575	0,526567	no
XLOC_009821	g4450	66,1536	90,2023	0,447345	0,72781	0,1906	0,440813	no
XLOC_009822	g10749	0	0	0	0	1	1	no
XLOC_009823	g10750	100,082	98,2474	-0,0266876	-0,0402294	0,94375	0,975334	no
XLOC_009824	g740	89,4948	435,74	2,28359	3,58522	5,00E-05	0,00120049	yes
XLOC_009825	g742	50,3173	143,851	1,51545	2,53903	5,00E-05	0,00120049	yes
XLOC_009826	g744	1,85164	2,62144	0,501555	0,499367	0,37865	0,636267	no
XLOC_009827	g747	182,9	123,608	-0,565285	-0,950244	0,10265	0,308736	no
XLOC_009828	g741	97,8957	36,1639	-1,4367	-1,75027	0,00235	0,0236687	yes
XLOC_009829	g743	13,9004	33,4764	1,26802	1,96836	0,0014	0,016042	yes
XLOC_009830	g745	73,0496	182,008	1,31705	2,00145	0,0008	0,0106019	yes
XLOC_009831	g746	4,36448	6,47044	0,568054	0,420258	0,46505	0,705296	no
XLOC_009832	g10751	26,1714	10,0932	-1,3746	-1,481	0,0106	0,0692562	no
XLOC_009833	g4451	22,3077	22,2255	-0,00532309	-0,00783262	0,98795	0,993718	no
XLOC_009834	g4453	30,4825	43,4829	0,512467	0,835251	0,14115	0,375025	no
XLOC_009835	g4455	77,2475	83,2511	0,107982	0,181636	0,7469	0,880922	no
XLOC_009836	g4452	35,2652	30,1253	-0,227266	-0,364299	0,51815	0,742916	no
XLOC_009837	g4454	104,853	127,67	0,284056	0,422541	0,46965	0,707649	no
XLOC_009838	g4456	155,045	160,153	0,0467634	0,0621451	0,91195	0,962529	no
XLOC_009839	g10752	0	0	0	0	1	1	no
XLOC_009840	g4457	5,25724	4,94773	-0,0875401	-0,0881136	0,87585	0,944007	no
XLOC_009841	g10753	0	0	0	0	1	1	no
XLOC_009842	g4459	0	0	0	0	1	1	no
XLOC_009843	g4460	0	0	0	0	1	1	no

XLOC_009844	g4458	0	0,0749712	inf	0	1	1	no
XLOC_009845	g748	6,03536	10,1761	0,753674	0,964067	0,09485	0,294197	no
XLOC_009846	g749	123,858	331,197	1,419	2,21246	0,00025	0,00430702	yes
XLOC_009847	g751	43,5571	65,0626	0,578919	0,992426	0,07875	0,261524	no
XLOC_009848	g752	151,008	353,723	1,228	2,0088	0,0006	0,00845337	yes
XLOC_009849	g753	214,301	712,1	1,73244	2,38398	0,00015	0,00294012	yes
XLOC_009850	g755	17,6396	12,4654	-0,500898	-0,646202	0,2669	0,527887	no
XLOC_009851	g750	163,253	214,961	0,396959	0,68397	0,2355	0,493515	no
XLOC_009852	g754	100,363	85,764	-0,226789	-0,347442	0,54525	0,761892	no
XLOC_009853	g10754	75,6334	52,541	-0,52558	-0,807573	0,16935	0,412659	no
XLOC_009854	g10755	30,8086	24,4986	-0,330633	-0,53328	0,35545	0,616046	no
XLOC_009855	g10756	12,7313	16,049	0,334107	0,525296	0,34265	0,602907	no
XLOC_009856	g10757	0,0109318	0,0573603	2,39152	0	1	1	no
XLOC_009857	g10758	0	0	0	0	1	1	no
XLOC_009858	g10759	51,2411	67,0833	0,388653	0,431374	0,45895	0,701036	no
XLOC_009859	g10760	0	0	0	0	1	1	no
XLOC_009860	g10761	1,25879	1,09359	-0,202974	-0,175356	0,756	0,884596	no
XLOC_009861	g4461	42,2413	31,8989	-0,405148	-0,671305	0,24455	0,503031	no
XLOC_009862	g4462	0	0	0	0	1	1	no
XLOC_009863	g4463	0	0	0	0	1	1	no
XLOC_009864	g10762	0	0	0	0	1	1	no
XLOC_009865	g10763	0	0	0	0	1	1	no
XLOC_009866	g4464	3,76094	4,53003	0,268427	0,29252	0,6204	0,808752	no
XLOC_009867	g10764	121,569	95,9917	-0,340795	-0,361666	0,54485	0,761892	no
XLOC_009868	g10765	1,2543	1,27106	0,0191502	0,0152534	0,94605	0,97576	no
XLOC_009869	g10766	80,5525	72,6732	-0,148506	-0,23547	0,67525	0,842561	no
XLOC_009870	g10768	73,9589	69,6659	-0,0862714	-0,149234	0,7939	0,904945	no
XLOC_009871	g10770	64,1192	90,9987	0,505089	0,804333	0,1532	0,391294	no
XLOC_009872	g10771	80,3575	58,0306	-0,469618	-0,79831	0,15055	0,387828	no
XLOC_009873	g10772	10,6515	11,795	0,147117	0,211738	0,7164	0,865607	no
XLOC_009874	g10774	122,347	161,601	0,401459	0,693448	0,2321	0,489945	no
XLOC_009875	g10767	45,0713	39,4357	-0,192709	-0,319584	0,5635	0,773346	no
XLOC_009876	g10769	102,554	58,7292	-0,804229	-1,29684	0,02355	0,122166	no
XLOC_009877	g10773	841,583	983,182	0,224353	0,331143	0,5625	0,772769	no
XLOC_009878	g10775	17,6836	31,8482	0,848796	1,02269	0,0772	0,257859	no
XLOC_009879	g10776	0	0	0	0	1	1	no
XLOC_009880	g4465	0	0	0	0	1	1	no
XLOC_009881	g10777	0	0	0	0	1	1	no
XLOC_009882	g4466	25,1533	27,5121	0,129315	0,172553	0,7613	0,88746	no
XLOC_009883	g4471	52,5917	70,1323	0,415244	0,693196	0,24155	0,499688	no
XLOC_009884	g4467	87,0805	30,5861	-1,50947	-2,51699	5,00E-05	0,00120049	yes
XLOC_009885	g4468	92,2579	92,1285	-0,00202499	-0,00320292	0,9953	0,997637	no
XLOC_009886	g4469	54,6716	66,6762	0,286382	0,467274	0,41995	0,671172	no
XLOC_009887	g4470	38,4265	52,2418	0,443104	0,750107	0,1948	0,446218	no
XLOC_009888	g4472	19,5427	17,5593	-0,154389	-0,242112	0,669	0,838813	no
XLOC_009889	g10778	96,0604	135,289	0,49403	0,784017	0,1623	0,404104	no
XLOC_009890	g10779	0	0	0	0	1	1	no
XLOC_009891	g4473	162,242	271,358	0,742049	1,22052	0,0322	0,147897	no
XLOC_009892	g4474	20,4912	24,1181	0,23511	0,342882	0,5598	0,771178	no
XLOC_009893	g4475	702,898	423,38	-0,731363	-1,0699	0,0577	0,214952	no
XLOC_009894	g10780	64,8699	79,0456	0,285137	0,451293	0,44675	0,692953	no
XLOC_009895	g4476	64,6695	118,576	0,874649	0,955172	0,09895	0,301861	no
XLOC_009896	g4478	0	0	0	0	1	1	no
XLOC_009897	g4477	0	0	0	0	1	1	no
XLOC_009898	g4479	0	0	0	0	1	1	no
XLOC_009899	g10781	10,3675	4,65895	-1,15399	-0,787257	0,2004	0,45251	no
XLOC_009900	g4481	76,8539	102,248	0,411884	0,711162	0,21105	0,466362	no
XLOC_009901	g4482	23,4203	28,844	0,300512	0,457325	0,4312	0,678624	no
XLOC_009902	g4480	89,2153	117,349	0,395437	0,690212	0,2359	0,493721	no
XLOC_009903	g10782	0	0	0	0	1	1	no
XLOC_009904	g10784	154,543	71,9934	-1,10207	-1,65097	0,0068	0,0508963	no
XLOC_009905	g10783	225,614	89,4101	-1,33534	-1,61623	0,0069	0,051293	no
XLOC_009906	g4484	0	0	0	0	1	1	no
XLOC_009907	g4483	3,57596	6,91569	0,951541	1,14409	0,0426	0,177109	no
XLOC_009908	g10785	3,86852	6,26997	0,696676	0,842272	0,15125	0,388714	no
XLOC_009909	g10786	31,2389	24,5215	-0,349297	-0,594315	0,29035	0,552994	no
XLOC_009910	g10787	37,0857	63,4164	0,773994	1,25412	0,0288	0,137959	no
XLOC_009911	g10788	12,8567	26,371	1,03643	1,44576	0,01465	0,0872426	no
XLOC_009912	g10789	6,16569	5,64877	-0,126327	-0,172647	0,7624	0,887899	no
XLOC_009913	g4487	26,3376	26,3435	0,000318967	0,000466386	0,996	0,997829	no
XLOC_009914	g4494	48,7549	82,4902	0,758675	1,25166	0,0268	0,132582	no
XLOC_009915	g4496	81,416	140,112	0,783198	1,2079	0,0393	0,168013	no
XLOC_009916	g4498	94,9262	122,972	0,373454	0,610169	0,2916	0,554382	no
XLOC_009917	g4485	190,306	152,14	-0,322922	-0,553443	0,3236	0,584128	no
XLOC_009918	g4486	100,194	85,7137	-0,225195	-0,276622	0,60855	0,80225	no
XLOC_009919	g4488	124,564	715,399	2,52186	3,02221	5,00E-05	0,00120049	yes
XLOC_009920	g4489	188,862	334,448	0,824448	1,43295	0,0124	0,0776582	no
XLOC_009921	g4490	18,3631	19,7801	0,107237	0,174973	0,7467	0,880922	no
XLOC_009922	g4491	31,8052	29,5306	-0,10705	-0,177493	0,7598	0,886717	no
XLOC_009923	g4492	12,2237	36,3186	1,57103	2,31183	0,00015	0,00294012	yes
XLOC_009924	g4493	159,796	109,27	-0,548332	-0,844142	0,1338	0,363462	no
XLOC_009925	g4495	45,5988	60,8639	0,416591	0,727937	0,2024	0,454925	no
XLOC_009926	g4497	195,505	610,236	1,64216	2,71914	5,00E-05	0,00120049	yes
XLOC_009927	g4500	159,958	65,9654	-1,27791	-2,03576	0,00035	0,00552572	yes
XLOC_009928	g4499	137,605	104,885	-0,39173	-0,607969	0,29515	0,557058	no
XLOC_009929	g757	84,7945	115,791	0,449477	0,742992	0,19205	0,442199	no
XLOC_009930	g759	32,5013	10,864	-1,58094	-2,43152	5,00E-05	0,00120049	yes
XLOC_009931	g756	116,751	45,2229	-1,3683	-2,32619	0,0001	0,00209829	yes
XLOC_009932	g758	240,652	87,4294	-1,46076	-2,25154	0,0001	0,00209829	yes
XLOC_009933	g10790	0	0	0	0	1	1	no
XLOC_009934	g10791	0	0	0	0	1	1	no
XLOC_009935	g4501	232,28	40,578	-2,5171	-3,49426	5,00E-05	0,00120049	yes

XLOC_009936	g4502	145,724	163,368	0,164886	0,272449	0,61625	0,807415	no
XLOC_009937	g4505	12,7301	19,6451	0,625927	0,969895	0,0943	0,292953	no
XLOC_009938	g4503	39,6126	42,9867	0,117931	0,19256	0,7294	0,871694	no
XLOC_009939	g4504	97,4071	167,921	0,785686	1,27489	0,0283	0,136697	no
XLOC_009940	g4506	337,579	275,026	-0,295658	-0,492306	0,38895	0,643879	no
XLOC_009941	g4507	0	0	0	0	1	1	no
XLOC_009942	g10792	50,7939	49,2434	-0,0447258	-0,0606676	0,91535	0,963526	no
XLOC_009943	g4508	13,9048	16,7128	0,265371	0,323243	0,5582	0,7703	no
XLOC_009944	g4509	154,738	94,3938	-0,713064	-0,977109	0,08695	0,27849	no
XLOC_009945	g4510	29,767	25,1932	-0,240683	-0,319316	0,5891	0,789433	no
XLOC_009946	g4511	56,4596	34,3834	-0,715506	-0,940749	0,09845	0,300802	no
XLOC_009947	g4512	36,0183	16,3294	-1,14126	-1,83114	0,0018	0,0192549	yes
XLOC_009948	g4513	0,668829	1,46933	1,13545	0,900971	0,12175	0,344351	no
XLOC_009949	g4514	4,92004	5,85177	0,250203	0,301382	0,60385	0,799232	no
XLOC_009950	g4515	131,052	78,0522	-0,747629	-1,23863	0,03115	0,144699	no
XLOC_009951	g10794	27,3727	21,8241	-0,326816	-0,496583	0,39345	0,64816	no
XLOC_009952	g10793	25,7479	23,1748	-0,1519	-0,197901	0,73515	0,874871	no
XLOC_009953	g760	10,2353	12,9279	0,336922	0,319081	0,5858	0,786728	no
XLOC_009954	g4518	15,4506	7,99287	-0,95088	-1,33814	0,02005	0,109202	no
XLOC_009955	g4519	232,136	309,255	0,41383	0,567536	0,3119	0,573032	no
XLOC_009956	g4516	36,7596	35,7992	-0,0381972	-0,0581554	0,91955	0,964535	no
XLOC_009957	g4517	179,483	143,961	-0,318168	-0,520521	0,37285	0,631165	no
XLOC_009958	g4520	75,3303	71,1542	-0,0822816	-0,129892	0,8173	0,917035	no
XLOC_009959	g10795	163,078	211,388	0,37433	0,599519	0,2785	0,540061	no
XLOC_009960	g10796	20,2254	91,2323	2,17337	3,00478	5,00E-05	0,00120049	yes
XLOC_009961	g4521	0	0	0	0	1	1	no
XLOC_009962	g10797	637,997	474,729	-0,426445	-0,628182	0,2738	0,535281	no
XLOC_009963	g10799	15,268	26,6179	0,801883	1,20684	0,0303	0,142503	no
XLOC_009964	g10798	38,7199	39,8407	0,041167	0,0714577	0,90095	0,956571	no
XLOC_009965	g10800	72,8577	54,89	-0,408538	-0,656404	0,2522	0,511378	no
XLOC_009966	g10801	41,4322	35,245	-0,233332	-0,262296	0,64115	0,821944	no
XLOC_009967	g4522	13,2168	8,66039	-0,609864	-0,905637	0,11045	0,32396	no
XLOC_009968	g4523	25,7891	18,6075	-0,470876	-0,709767	0,23265	0,490578	no
XLOC_009969	g10802	0	0	0	0	1	1	no
XLOC_009970	g10803	503,952	648,507	0,363835	0,525656	0,3495	0,610149	no
XLOC_009971	g10804	180,876	153,298	-0,238659	-0,403293	0,48915	0,721963	no
XLOC_009972	g10805	0	0	0	0	1	1	no
XLOC_009973	g10806	8,1316	7,10997	-0,193695	-0,208785	0,7105	0,862876	no
XLOC_009974	g10807	0	0	0	0	1	1	no
XLOC_009975	g761	0,395687	0,12648	-1,64545	0	1	1	no
XLOC_009976	g10808	0	0	0	0	1	1	no
XLOC_009977	g10809	0	0	0	0	1	1	no
XLOC_009978	g10810	4,16377	8,40809	1,01389	0,94497	0,2426	0,500911	no
XLOC_009979	g763	398,232	241,044	-0,724313	-1,21355	0,03425	0,153718	no
XLOC_009980	g766	74,934	62,2571	-0,267382	-0,42234	0,4606	0,701908	no
XLOC_009981	g770	33,6347	40,725	0,275964	0,463083	0,4204	0,671244	no
XLOC_009982	g771	13,1459	1,64168	-3,00137	-3,35222	5,00E-05	0,00120049	yes
XLOC_009983	g773	60,4051	56,181	-0,104589	-0,144812	0,79675	0,906252	no
XLOC_009984	g762	109,036	138,819	0,348396	0,586819	0,2994	0,561625	no
XLOC_009985	g764	173,958	148,1	-0,232167	-0,36922	0,5113	0,73751	no
XLOC_009986	g765	162,29	138,712	-0,226485	-0,387496	0,49835	0,728135	no
XLOC_009987	g767	57,4226	65,7297	0,194927	0,330952	0,55285	0,766659	no
XLOC_009988	g768	395,339	461,108	0,222014	0,331489	0,5583	0,7703	no
XLOC_009989	g769	81,7731	126,178	0,62576	0,864677	0,12935	0,355703	no
XLOC_009990	g772	141,172	194,438	0,461855	0,699591	0,21405	0,469524	no
XLOC_009991	g10811	8,53665	14,4226	0,756591	0,925326	0,11115	0,325333	no
XLOC_009992	g10812	50,1881	46,4299	-0,112291	-0,18774	0,7399	0,876968	no
XLOC_009993	g10813	57,6088	23,403	-1,29959	-1,45356	0,01435	0,0860825	no
XLOC_009994	g10814	4,97627	3,92399	-0,342745	-0,394689	0,47075	0,708578	no
XLOC_009995	g4524	0	0	0	0	1	1	no
XLOC_009996	g10815	53,4307	32,3259	-0,72498	-0,930412	0,1063	0,316323	no
XLOC_009997	g10816	0	0	0	0	1	1	no
XLOC_009998	g10817	0	0	0	0	1	1	no
XLOC_009999	g10818	18,328	13,115	-0,482825	-0,609006	0,29725	0,559302	no
XLOC_010000	g10819	1,50282	1,9359	0,365338	0,237494	0,7252	0,869807	no
XLOC_010001	g10820	0	0	0	0	1	1	no
XLOC_010002	g4525	0	0	0	0	1	1	no
XLOC_010003	g4526	0	0	0	0	1	1	no
XLOC_010004	g4527	38,8693	40,4565	0,0577412	0,0880874	0,87555	0,943891	no
XLOC_010005	g4528	0	0	0	0	1	1	no
XLOC_010006	g10821	0,382261	0,205362	-0,896388	0	1	1	no
XLOC_010007	g10822	41,8697	22,2462	-0,912346	-0,964979	0,1119	0,326749	no
XLOC_010008	g10823	0	0,318997	inf	0	1	1	no
XLOC_010009	g4531	9,63665	10,4982	0,123533	0,179785	0,7544	0,884245	no
XLOC_010010	g4532	64,1089	76,1725	0,248746	0,413414	0,46575	0,706248	no
XLOC_010011	g4533	14,0888	8,00597	-0,815397	-1,18045	0,03775	0,163956	no
XLOC_010012	g4529	1,57906	2,02536	0,359116	0,366831	0,5284	0,749406	no
XLOC_010013	g4530	13,9099	17,1073	0,298505	0,339544	0,5549	0,768239	no
XLOC_010014	g10824	0	0	0	0	1	1	no
XLOC_010015	g4535	42,8182	53,6041	0,324121	0,504687	0,3495	0,610149	no
XLOC_010016	g4534	428,334	503,649	0,233682	0,35042	0,53485	0,754197	no
XLOC_010017	g4536	4,04322	9,07303	1,16608	1,19101	0,0405	0,171279	no
XLOC_010018	g4537	1626,91	4039,74	1,31213	1,61638	0,0041	0,0354419	yes
XLOC_010019	g10825	0	0	0	0	1	1	no
XLOC_010020	g4538	30,7191	26,4319	-0,216852	-0,323738	0,56015	0,771227	no
XLOC_010021	g4539	113,624	93,0474	-0,288234	-0,47222	0,4106	0,663116	no
XLOC_010022	g10826	0	0	0	0	1	1	no
XLOC_010023	g10827	2,51685	0,811283	-1,63334	0	1	1	no
XLOC_010024	g4540	133,477	185,776	0,476973	0,736246	0,1921	0,442199	no
XLOC_010025	g4541	2,30778	0	#NAME?	#NAME?	0,00465	0,0389616	yes
XLOC_010026	g10828	48,6998	16,0611	-1,60034	-2,18857	0,00025	0,00430702	yes
XLOC_010027	g4542	0	0	0	0	1	1	no

XLOC_010028	g10829	110	135,353	0,299223	0,498197	0,37975	0,636808	no
XLOC_010029	g10830	107,829	142,498	0,402191	0,675972	0,24065	0,49898	no
XLOC_010030	g10831	94,1686	45,9708	-1,03453	-1,59525	0,00685	0,0511536	no
XLOC_010031	g4543	37,7822	40,7435	0,108863	0,189374	0,7449	0,879937	no
XLOC_010032	g4544	87,1883	123,829	0,506139	0,81606	0,14995	0,387278	no
XLOC_010033	g4545	19,7179	26,0253	0,400404	0,455865	0,4209	0,671416	no
XLOC_010034	g4546	15,1758	13,0652	-0,216049	-0,251445	0,6581	0,832266	no
XLOC_010035	g10833	177,861	153,794	-0,209752	-0,351882	0,53505	0,754371	no
XLOC_010036	g10834	40,0404	44,7111	0,15918	0,273518	0,6321	0,816117	no
XLOC_010037	g10832	118,624	116,024	-0,0319749	-0,0475348	0,93175	0,970131	no
XLOC_010038	g10835	602,564	332,921	-0,855932	-1,00477	0,09775	0,299843	no
XLOC_010039	g10836	0	0	0	0	1	1	no
XLOC_010040	g4549	36,6655	37,1467	0,0188136	0,0323637	0,95325	0,979073	no
XLOC_010041	g4547	27,8598	23,4534	-0,248387	-0,423619	0,44755	0,693614	no
XLOC_010042	g4548	505,007	357,15	-0,499773	-0,824803	0,15915	0,399502	no
XLOC_010043	g4550	35,5337	33,6436	-0,078853	-0,1202	0,8285	0,921598	no
XLOC_010044	g774	15,7385	3,32028	-2,24492	-2,58768	0,00495	0,0407111	yes
XLOC_010045	g775	15,4067	6,39756	-1,26796	-1,82475	0,0013	0,0151795	yes
XLOC_010046	g4551	212,764	407,338	0,936973	1,34535	0,019	0,104703	no
XLOC_010047	g10837	3,57193	17,3989	2,28422	2,12012	0,00105	0,0130684	yes
XLOC_010048	g4552	16,4378	9,14846	-0,845416	-1,21125	0,0391	0,167377	no
XLOC_010049	g10838	50,4549	42,4632	-0,248783	-0,426115	0,4542	0,698846	no
XLOC_010050	g10839	86,4278	55,9418	-0,627571	-0,99381	0,08595	0,276731	no
XLOC_010051	g10840	0	0	0	0	1	1	no
XLOC_010052	g10841	0	0	0	0	1	1	no
XLOC_010053	g10842	3,99617	2,29852	-0,797912	-0,768092	0,1777	0,424594	no
XLOC_010054	g10843	24,4011	29,6455	0,280867	0,339989	0,54715	0,76377	no
XLOC_010055	g10844	546,712	494,003	-0,146262	-0,186645	0,73725	0,875852	no
XLOC_010056	g10845	80,92	128,363	0,665659	1,02783	0,06905	0,240878	no
XLOC_010057	g10849	68,9938	73,7726	0,0966186	0,166813	0,7711	0,893264	no
XLOC_010058	g10846	754,478	918,34	0,28355	0,440349	0,44185	0,688366	no
XLOC_010059	g10847	26,4212	26,9629	0,0292798	0,0459137	0,93605	0,972082	no
XLOC_010060	g10848	45,0144	26,9342	-0,740945	-1,19373	0,04025	0,170663	no
XLOC_010061	g4553	28,9559	21,5208	-0,428122	-0,648628	0,25895	0,518851	no
XLOC_010062	g4554	36,099	32,2718	-0,161688	-0,267647	0,64155	0,822242	no
XLOC_010063	g4555	16,9527	91,2085	2,42766	3,81882	5,00E-05	0,00120049	yes
XLOC_010064	g4556	21,8946	17,3259	-0,337648	-0,524659	0,3593	0,619938	no
XLOC_010065	g10850	0	0	0	0	1	1	no
XLOC_010066	g10851	0,770597	0,7949	0,0447964	0	1	1	no
XLOC_010067	g10852	0,123182	0,875525	2,82936	0	1	1	no
XLOC_010068	g10853	9,17694	8,28864	-0,146878	-0,214357	0,7126	0,863918	no
XLOC_010069	g10854	69,1744	60,9737	-0,182053	-0,308742	0,60595	0,800219	no
XLOC_010070	g10855	7,55667	7,57607	0,00369964	0,00277913	0,9383	0,972362	no
XLOC_010071	g10856	99,2957	65,6397	-0,597162	-0,853672	0,1226	0,345759	no
XLOC_010072	g10857	2,47656	2,02322	-0,291686	-0,333424	0,5733	0,779376	no
XLOC_010073	g10858	1169,94	522,503	-1,16293	-1,73135	0,00265	0,0257909	yes
XLOC_010074	g10859	59,9102	67,9059	0,180736	0,288903	0,59295	0,791419	no
XLOC_010075	g10860	0,828082	0,552035	-0,585013	0	1	1	no
XLOC_010076	g4557	10,8984	6,97383	-0,644099	-0,784598	0,1856	0,433654	no
XLOC_010077	g10861	0	0,269438	inf	0	1	1	no
XLOC_010078	g10862	0,0382966	0	#NAME?	0	1	1	no
XLOC_010079	g10863	1,42685	1,91187	0,422154	0,409087	0,50705	0,734472	no
XLOC_010080	g4558	6,38725	3,01973	-1,08078	-1,33523	0,0259	0,1295	no
XLOC_010081	g10864	110,838	114,724	0,0497096	0,0615562	0,91425	0,963407	no
XLOC_010082	g10865	68,1018	79,4156	0,221729	0,27962	0,6146	0,806329	no
XLOC_010083	g10866	81,4448	87,2204	0,0988426	0,14776	0,7932	0,904567	no
XLOC_010084	g4559	20,2875	11,8173	-0,779691	-0,966075	0,08175	0,26867	no
XLOC_010085	g4560	0	0	0	0	1	1	no
XLOC_010086	g4561	0	0	0	0	1	1	no
XLOC_010087	g10867	2,30785	2,5017	0,116359	0,132059	0,8188	0,917355	no
XLOC_010088	g4563	19,9338	12,8507	-0,633367	-0,831783	0,14685	0,383835	no
XLOC_010089	g4562	5,67121	8,56744	0,595209	0,79756	0,1749	0,420754	no
XLOC_010090	g10869	11,4434	16,61	0,537537	0,822147	0,16295	0,405106	no
XLOC_010091	g10868	1,56085	1,68119	0,107152	0,0686006	0,89095	0,951302	no
XLOC_010092	g10870	0	0	0	0	1	1	no
XLOC_010093	g4564	111,038	76,2744	-0,541787	-0,865406	0,12765	0,352906	no
XLOC_010094	g4565	0,084619	0	#NAME?	0	1	1	no
XLOC_010095	g4566	0	0	0	0	1	1	no
XLOC_010096	g10871	0	0,0466014	inf	0	1	1	no
XLOC_010097	g4567	30,3512	41,3818	0,447243	0,746729	0,1893	0,439151	no
XLOC_010098	g4569	68,9209	50,9651	-0,435431	-0,728758	0,20045	0,45251	no
XLOC_010099	g4568	37,3025	4,57522	-3,02736	-3,22345	5,00E-05	0,00120049	yes
XLOC_010100	g4570	100,101	85,6335	-0,225206	-0,338243	0,54755	0,76377	no
XLOC_010101	g4571	0,231131	0,42414	0,875829	0	1	1	no
XLOC_010102	g4572	0,05671	0,0844231	0,574034	0	1	1	no
XLOC_010103	g10872	0	0	0	0	1	1	no
XLOC_010104	g4573	0	0	0	0	1	1	no
XLOC_010105	g4574	0	0	0	0	1	1	no
XLOC_010106	g4575	0	0,259047	inf	0	1	1	no
XLOC_010107	g10873	40,4276	93,7795	1,21393	1,81527	0,00145	0,0164423	yes
XLOC_010108	g10874	0	0	0	0	1	1	no
XLOC_010109	g4576	57,6484	61,8025	0,100387	0,173483	0,75785	0,8854	no
XLOC_010110	g4577	67,382	219,038	1,70074	2,79592	5,00E-05	0,00120049	yes
XLOC_010111	g4578	168,984	212,422	0,330048	0,568023	0,32415	0,584386	no
XLOC_010112	g4582	58,4783	54,3103	-0,106674	-0,17755	0,74975	0,881953	no
XLOC_010113	g4584	69,7868	105,157	0,591513	0,85398	0,12525	0,348824	no
XLOC_010114	g4586	64,22	61,8028	-0,0553488	-0,0917025	0,87205	0,942536	no
XLOC_010115	g4579	28,2396	39,6798	0,490688	0,83037	0,1501	0,387278	no
XLOC_010116	g4580	50,9413	21,2406	-1,26201	-1,88385	0,00135	0,0156148	yes
XLOC_010117	g4581	64,8258	65,8355	0,022297	0,0379896	0,94745	0,976281	no
XLOC_010118	g4583	58,6173	61,1568	0,0611862	0,0975396	0,8632	0,936801	no
XLOC_010119	g4585	122,578	143,996	0,232329	0,356286	0,53015	0,750804	no

XLOC_010120	g10875	3,2887	3,6236	0,139909	0,156105	0,7893	0,90274	no
XLOC_010121	g4587	0	0	0	0	1	1	no
XLOC_010122	g10876	0	0	0	0	1	1	no
XLOC_010123	g10877	0	0	0	0	1	1	no
XLOC_010124	g10878	32,6329	13,6397	-1,25852	-1,46399	0,0183	0,101932	no
XLOC_010125	g10879	1689,45	2168,73	0,360297	0,573425	0,3178	0,578273	no
XLOC_010126	g10880	60,9834	18,0782	-1,75416	-2,64097	5,00E-05	0,00120049	yes
XLOC_010127	g10881	2,24429	5,16716	1,20311	1,09152	0,1408	0,374399	no
XLOC_010128	g10882	33,7516	49,6441	0,556669	0,913652	0,10925	0,321786	no
XLOC_010129	g4588	2,02359	4,67114	1,20686	0,881433	0,16075	0,40208	no
XLOC_010130	g10883	0	0	0	0	1	1	no
XLOC_010131	g4589	15,1763	16,8322	0,149405	0,240015	0,66105	0,833956	no
XLOC_010132	g10884	0	0	0	0	1	1	no
XLOC_010133	g10885	31,3261	24,4843	-0,355505	-0,554338	0,32495	0,585506	no
XLOC_010134	g10886	7,32639	9,29122	0,342765	0,35631	0,6408	0,821603	no
XLOC_010135	g10887	0	0	0	0	1	1	no
XLOC_010136	g10888	0	0	0	0	1	1	no
XLOC_010137	g4590	73,2097	122,994	0,748477	1,11689	0,0481	0,191154	no
XLOC_010138	g10890	55,4652	57,6439	0,0555845	0,0810971	0,8845	0,948388	no
XLOC_010139	g10889	1,99054	4,22821	1,08688	1,22797	0,03795	0,164605	no
XLOC_010140	g4591	0	0	0	0	1	1	no
XLOC_010141	g10891	0	0	0	0	1	1	no
XLOC_010142	g4592	48,5889	109,708	1,17497	1,91927	0,00125	0,0146655	yes
XLOC_010143	g4593	123,61	77,011	-0,682661	-1,13194	0,04745	0,189337	no
XLOC_010144	g4594	66,7396	68,5002	0,0375645	0,0505551	0,92545	0,967112	no
XLOC_010145	g4595	1417,85	2432,9	0,778967	1,21873	0,0317	0,146422	no
XLOC_010146	g4596	55,0659	37,05	-0,571689	-0,756699	0,1994	0,451802	no
XLOC_010147	g10892	0	0	0	0	1	1	no
XLOC_010148	g4597	0	0	0	0	1	1	no
XLOC_010149	g10893	0	0	0	0	1	1	no
XLOC_010150	g10894	0	0	0	0	1	1	no
XLOC_010151	g10895	44,2195	56,443	0,352111	0,551809	0,33405	0,594729	no
XLOC_010152	g10896	55,893	66,6049	0,25296	0,43356	0,4484	0,694306	no
XLOC_010153	g10897	10,7102	4,27489	-1,32502	-1,22022	0,11815	0,337571	no
XLOC_010154	g10898	203,088	170,23	-0,254622	-0,423866	0,4489	0,694314	no
XLOC_010155	g10899	11,9156	13,1939	0,147022	0,209473	0,70365	0,859326	no
XLOC_010156	g4598	147,28	245,89	0,739451	0,97243	0,08505	0,275368	no
XLOC_010157	g4599	389,775	465,823	0,257142	0,414716	0,45645	0,701012	no
XLOC_010158	g4601	123,438	139,292	0,174333	0,287361	0,611	0,804448	no
XLOC_010159	g4600	1021,76	574,447	-0,830814	-0,799911	0,12735	0,352176	no
XLOC_010160	g4602	0	0	0	0	1	1	no
XLOC_010161	g4604	45,8775	35,2674	-0,37945	-0,627168	0,2747	0,5364	no
XLOC_010162	g4603	246,446	95,7474	-1,36397	-1,89309	0,0018	0,0192549	yes
XLOC_010163	g4605	11,3536	7,75124	-0,550646	-0,810134	0,13515	0,365713	no
XLOC_010164	g4606	22,8069	16,8149	-0,439728	-0,656228	0,2444	0,503031	no
XLOC_010165	g4607	2,09287	1,77746	-0,235667	-0,274258	0,62485	0,811966	no
XLOC_010166	g4608	61,5839	74,6485	0,27756	0,479306	0,4026	0,656406	no
XLOC_010167	g10900	0	0	0	0	1	1	no
XLOC_010168	g10901	14,6919	16,0621	0,128641	0,154024	0,7903	0,903358	no
XLOC_010169	g4609	38,1847	33,2281	-0,200594	-0,318505	0,57585	0,780604	no
XLOC_010170	g10902	42,3267	116,238	1,45744	2,38651	0,0001	0,00209829	yes
XLOC_010171	g4610	77,3239	84,769	0,132624	0,208363	0,71525	0,865069	no
XLOC_010172	g10903	18,0992	25,7409	0,508134	0,745358	0,1994	0,451802	no
XLOC_010173	g10904	0	0	0	0	1	1	no
XLOC_010174	g10905	0	0	0	0	1	1	no
XLOC_010175	g777	21,2497	51,3456	1,2728	1,96031	0,0009	0,0115984	yes
XLOC_010176	g779	20,777	34,5311	0,732909	1,10541	0,058	0,215579	no
XLOC_010177	g776	30,2196	32,5811	0,108554	0,173527	0,7544	0,884245	no
XLOC_010178	g778	49,9855	65,4123	0,388053	0,648656	0,24535	0,50394	no
XLOC_010179	g780	110,531	212,739	0,944633	1,54458	0,0075	0,0546771	no
XLOC_010180	g781	166,972	179,753	0,106409	0,183752	0,7556	0,884596	no
XLOC_010181	g10906	0	0	0	0	1	1	no
XLOC_010182	g10907	0	0	0	0	1	1	no
XLOC_010183	g10908	122,263	64,0649	-0,932383	-1,41026	0,01025	0,0677355	no
XLOC_010184	g10910	25,604	31,1899	0,284707	0,403934	0,48855	0,721546	no
XLOC_010185	g10909	76,6678	87,5175	0,190952	0,303067	0,60135	0,797684	no
XLOC_010186	g10912	19,0489	30,3067	0,669927	1,0208	0,08605	0,276941	no
XLOC_010187	g10911	5,56482	7,31426	0,394379	0,506147	0,3775	0,635531	no
XLOC_010188	g10913	1,24162	9,50485	2,93645	2,57777	0,00105	0,0130684	yes
XLOC_010189	g10914	5,55024	6,00529	0,113684	0,0895782	0,8852	0,94862	no
XLOC_010190	g10915	0	0	0	0	1	1	no
XLOC_010191	g10916	3,19642	23,3565	2,86929	3,39117	5,00E-05	0,00120049	yes
XLOC_010192	g10917	31,3074	32,6741	0,0616442	0,0736647	0,8976	0,954356	no
XLOC_010193	g4613	29,1362	19,5479	-0,575798	-0,855243	0,13485	0,365103	no
XLOC_010194	g4611	90,5872	120,977	0,417351	0,516765	0,3737	0,63195	no
XLOC_010195	g4612	14,952	6,29816	-1,24733	-1,00729	0,1078	0,319291	no
XLOC_010196	g10918	15,668	8,21194	-0,932022	-0,940928	0,0997	0,302738	no
XLOC_010197	g4614	20,023	17,592	-0,186738	-0,292764	0,6112	0,804448	no
XLOC_010198	g10919	46,9049	17,9633	-1,38469	-1,4915	0,01155	0,07365	no
XLOC_010199	g10920	1493,95	3051,57	1,03042	1,69414	0,00295	0,0280164	yes
XLOC_010200	g4615	0	0,0612657	inf	0	1	1	no
XLOC_010201	g10921	0	0	0	0	1	1	no
XLOC_010202	g4616	38,3748	40,349	0,0723717	0,123307	0,8264	0,920883	no
XLOC_010203	g4617	90,4298	204,484	1,17712	1,9739	0,00105	0,0130684	yes
XLOC_010204	g4618	113,114	333,283	1,55898	2,49045	5,00E-05	0,00120049	yes
XLOC_010205	g10922	0	0	0	0	1	1	no
XLOC_010206	g4619	0	0	0	0	1	1	no
XLOC_010207	g4620	0	0	0	0	1	1	no
XLOC_010208	g10923	1,88006	12,2195	2,70033	4,32867	0,16085	0,402227	no
XLOC_010209	g10924	1,6308	11,7644	2,85077	3,22127	5,00E-05	0,00120049	yes
XLOC_010210	g10925	20,0843	19,4494	-0,0463454	-0,066294	0,9101	0,961918	no
XLOC_010211	g4621	11,8807	13,4271	0,176523	0,259168	0,65565	0,831391	no

XLOC_010212	g10926	13,3081	18,4015	0,467517	0,47022	0,4098	0,662426	no
XLOC_010213	g4624	9,02346	11,79	0,385814	0,50258	0,3792	0,636522	no
XLOC_010214	g4622	1,55452	3,74606	1,26891	1,26191	0,03975	0,169274	no
XLOC_010215	g4623	2,44426	7,38443	1,59509	0	1	1	no
XLOC_010216	g4625	0,146073	0,267509	0,872898	0	1	1	no
XLOC_010217	g4626	0	0	0	0	1	1	no
XLOC_010218	g10927	0,0706988	0,554361	2,97107	0	1	1	no
XLOC_010219	g10928	0	0,16385	inf	0	1	1	no
XLOC_010220	g10929	0	0	0	0	1	1	no
XLOC_010221	g10930	19,5712	15,843	-0,304885	-0,435248	0,4287	0,677386	no
XLOC_010222	g782	85,4074	69,734	-0,292498	-0,489	0,37435	0,632807	no
XLOC_010223	g783	33,6434	84,4337	1,32749	2,16655	0,0001	0,00209829	yes
XLOC_010224	g4629	71,1827	75,6649	0,0880985	0,119636	0,8339	0,924151	no
XLOC_010225	g4627	81,2806	145,622	0,841248	1,39853	0,01885	0,10411	no
XLOC_010226	g4628	76,2755	74,4866	-0,0342399	-0,0574647	0,9173	0,964029	no
XLOC_010227	g4630	69,6239	40,7777	-0,771801	-1,22866	0,03285	0,149971	no
XLOC_010228	g4631	8,11107	6,21217	-0,384796	-0,516133	0,37605	0,634261	no
XLOC_010229	g4634	19,2552	29,3553	0,608374	1,01453	0,07385	0,251193	no
XLOC_010230	g4632	14,1263	19,4487	0,461293	0,634389	0,27295	0,53424	no
XLOC_010231	g4633	132,924	113,221	-0,231459	-0,362816	0,5164	0,741989	no
XLOC_010232	g10931	45,6179	135,609	1,57178	2,21224	0,0003	0,00496796	yes
XLOC_010233	g10932	118,393	111,721	-0,0836872	-0,138962	0,8094	0,912397	no
XLOC_010234	g4635	0,0716366	0	#NAME?	0	1	1	no
XLOC_010235	g10933	0	0	0	0	1	1	no
XLOC_010236	g10934	44,8312	31,7178	-0,499212	-0,763211	0,1664	0,409639	no
XLOC_010237	g10935	12,1405	20,489	0,755029	0,819267	0,15215	0,389904	no
XLOC_010238	g10936	72,1703	53,7678	-0,424665	-0,661123	0,25015	0,508587	no
XLOC_010239	g10937	21,8997	37,3704	0,770987	1,23913	0,0335	0,151669	no
XLOC_010240	g4636	53,011	57,955	0,128642	0,222281	0,68965	0,851227	no
XLOC_010241	g4637	57,0292	32,7118	-0,801891	-0,817089	0,1626	0,404543	no
XLOC_010242	g4638	3,04086	1,7749	-0,776737	-0,67034	0,332	0,592663	no
XLOC_010243	g784	0,171194	0	#NAME?	0	1	1	no
XLOC_010244	g10938	14,0594	14,6015	0,0545801	0,0781217	0,89045	0,951189	no
XLOC_010245	g10939	0	0	0	0	1	1	no
XLOC_010246	g785	139,691	110,046	-0,344126	-0,576557	0,32025	0,580544	no
XLOC_010247	g787	27,2911	50,0125	0,873862	1,48678	0,00985	0,0657113	no
XLOC_010248	g789	60,6706	60,2996	-0,00884814	-0,0135175	0,9809	0,990584	no
XLOC_010249	g786	2,77675	1,60796	-0,788166	-0,873443	0,1258	0,349959	no
XLOC_010250	g788	48,3738	93,9687	0,957954	1,64532	0,004	0,0348845	yes
XLOC_010251	g790	24,3913	30,1106	0,303909	0,479087	0,38465	0,64084	no
XLOC_010252	g791	27,3359	20,749	-0,397757	-0,661636	0,25125	0,510076	no
XLOC_010253	g4639	3,74491	3,2656	-0,197582	-0,19045	0,75105	0,882848	no
XLOC_010254	g4640	28,9677	26,8731	-0,108284	-0,169722	0,7684	0,892242	no
XLOC_010255	g4641	17,6911	28,5606	0,690998	1,11016	0,05505	0,208964	no
XLOC_010256	g4642	1,06919	0,876454	-0,286771	0	1	1	no
XLOC_010257	g10940	0	0	0	0	1	1	no
XLOC_010258	g4643	34,8857	40,7739	0,225009	0,372844	0,5163	0,741989	no
XLOC_010259	g4644	133,943	148,003	0,14401	0,216892	0,7044	0,859387	no
XLOC_010260	g4645	9,6716	18,8994	0,966516	1,26145	0,04085	0,172008	no
XLOC_010261	g4647	46,7744	66,0511	0,497864	0,837587	0,14295	0,378099	no
XLOC_010262	g4649	356,096	427,185	0,262592	0,257651	0,60705	0,800918	no
XLOC_010263	g4646	22,2133	22,8988	0,0438503	0,0745927	0,8952	0,953456	no
XLOC_010264	g4648	104,461	82,866	-0,334108	-0,562794	0,31845	0,578786	no
XLOC_010265	g4650	47,5748	100,913	1,08485	1,82994	0,00255	0,0250912	yes
XLOC_010266	g4651	63,6181	63,4206	-0,00448518	-0,00654751	0,99	0,99501	no
XLOC_010267	g4653	8,78917	9,3889	0,0952282	0,129851	0,82135	0,918849	no
XLOC_010268	g4652	11,8765	11,7511	-0,0153141	-0,022439	0,9718	0,986977	no
XLOC_010269	g10941	50,5622	46,7067	-0,114429	-0,191044	0,7424	0,878159	no
XLOC_010270	g10943	149,013	205,445	0,463314	0,75576	0,19315	0,443888	no
XLOC_010271	g10942	119,032	102,864	-0,210602	-0,367926	0,5206	0,744327	no
XLOC_010272	g10944	2,78927	2,67503	-0,0603331	-0,0710637	0,89695	0,954181	no
XLOC_010273	g10945	19,8011	16,9546	-0,223905	-0,326268	0,57015	0,777729	no
XLOC_010274	g4654	0	0	0	0	1	1	no
XLOC_010275	g4655	0	0	0	0	1	1	no
XLOC_010276	g4656	0	0	0	0	1	1	no
XLOC_010277	g4657	0	0	0	0	1	1	no
XLOC_010278	g4658	0	0	0	0	1	1	no
XLOC_010279	g10946	20,0802	16,0099	-0,326807	-0,475671	0,3971	0,651251	no
XLOC_010280	g10947	316,668	379,3	0,260366	0,446925	0,4283	0,677386	no
XLOC_010281	g10948	6,17764	5,10154	-0,276124	-0,312608	0,59005	0,790126	no
XLOC_010282	g10949	134,137	205,394	0,614693	1,02342	0,06665	0,235094	no
XLOC_010283	g10950	0	0	0	0	1	1	no
XLOC_010284	g10951	134,747	123,578	-0,124836	-0,193678	0,72795	0,870385	no
XLOC_010285	g10952	5,44936	7,70379	0,49948	0,518473	0,36575	0,625461	no
XLOC_010286	g10953	9,9991	16,9077	0,757807	0,991168	0,0817	0,26867	no
XLOC_010287	g10955	21,1094	23,2271	0,137925	0,236124	0,68025	0,846112	no
XLOC_010288	g10957	8,78085	5,96332	-0,558245	-0,78904	0,1604	0,401613	no
XLOC_010289	g10954	45,1124	38,0647	-0,245071	-0,413485	0,45855	0,701036	no
XLOC_010290	g10956	53,185	87,3789	0,716265	1,22241	0,03145	0,145541	no
XLOC_010291	g10958	0	0	0	0	1	1	no
XLOC_010292	g10959	0	0	0	0	1	1	no
XLOC_010293	g4659	33,9852	46,1435	0,44122	0,741212	0,19445	0,445832	no
XLOC_010294	g4661	243,055	202,017	-0,266803	-0,452962	0,4329	0,679955	no
XLOC_010295	g4660	59,0034	62,5695	0,0846611	0,143728	0,79805	0,907108	no
XLOC_010296	g4662	29,1121	16,0007	-0,863485	-1,2663	0,02865	0,137614	no
XLOC_010297	g10960	34,7379	60,3688	0,797293	1,27641	0,02405	0,123779	no
XLOC_010298	g10961	0,0986618	0,238444	1,27309	0	1	1	no
XLOC_010299	g10962	0	0	0	0	1	1	no
XLOC_010300	g792	92,7212	59,9056	-0,630208	-1,03297	0,07195	0,247391	no
XLOC_010301	g793	27,6827	25,8873	-0,0967369	-0,165249	0,7693	0,892759	no
XLOC_010302	g10963	7,04814	4,65784	-0,597584	-0,800728	0,16305	0,40515	no
XLOC_010303	g10964	0	5,60744	inf	#NAME?	0,013	0,0805426	no

XLOC_010304	g10965	0	0	0	0	1	1	no
XLOC_010305	g4663	0,0456865	0,897597	4,29623	0	1	1	no
XLOC_010306	g4664	0	0	0	0	1	1	no
XLOC_010307	g4665	0	0	0	0	1	1	no
XLOC_010308	g4666	27,3596	62,4441	1,19052	1,89738	0,0015	0,0168343	yes
XLOC_010309	g4667	82,2155	136,903	0,735667	1,2712	0,02725	0,133597	no
XLOC_010310	g4668	361,262	116,975	-1,62685	-2,16129	0,00015	0,00294012	yes
XLOC_010311	g4669	82,7869	17,2379	-2,26382	-2,67131	5,00E-05	0,00120049	yes
XLOC_010312	g4670	0	0	0	0	1	1	no
XLOC_010313	g4671	5,62395	2,69663	-1,06043	-1,06065	0,0646	0,230597	no
XLOC_010314	g4672	6,63898	3,51483	-0,917507	-1,17983	0,0417	0,174624	no
XLOC_010315	g4673	51,2865	45,7474	-0,164889	-0,256478	0,6574	0,832081	no
XLOC_010316	g4674	21,3924	11,6652	-0,874893	-1,45837	0,01085	0,070421	no
XLOC_010317	g10966	0	0	0	0	1	1	no
XLOC_010318	g4675	1,0884	1,39899	0,362177	0,316012	0,5901	0,790126	no
XLOC_010319	g10967	0,353241	0,299628	-0,237479	0	1	1	no
XLOC_010320	g10968	7,80034	3,87763	-1,00836	-1,21368	0,0426	0,177109	no
XLOC_010321	g10969	0	0	0	0	1	1	no
XLOC_010322	g10970	0	0,722003	inf	0	1	1	no
XLOC_010323	g4676	53,3354	81,4001	0,609938	0,528173	0,3839	0,640475	no
XLOC_010324	g4677	0	0	0	0	1	1	no
XLOC_010325	g10971	34,9071	24,4557	-0,513347	-0,647649	0,2655	0,52639	no
XLOC_010326	g10972	3,37933	11,8673	1,81219	0	1	1	no
XLOC_010327	g794	107,057	80,0289	-0,419785	-0,724119	0,2093	0,464271	no
XLOC_010328	g795	76,2996	100,632	0,399346	0,639505	0,263	0,523335	no
XLOC_010329	g797	96,152	96,9364	0,0117207	0,0200239	0,97265	0,987329	no
XLOC_010330	g798	10,1681	7,09264	-0,519649	-0,692037	0,2305	0,48772	no
XLOC_010331	g799	35,47	19,312	-0,877102	-1,1879	0,0387	0,166386	no
XLOC_010332	g802	505,505	1716,72	1,76386	2,74661	5,00E-05	0,00120049	yes
XLOC_010333	g796	3,80837	3,00277	-0,342878	-0,454061	0,44075	0,68701	no
XLOC_010334	g800	14,9431	13,479	-0,148764	-0,192907	0,7489	0,881846	no
XLOC_010335	g801	62,4555	57,1936	-0,126975	-0,209842	0,7108	0,862876	no
XLOC_010336	g10973	0	0	0	0	1	1	no
XLOC_010337	g4678	0	0	0	0	1	1	no
XLOC_010338	g4679	22,2853	11,4992	-0,954561	-1,41026	0,0135	0,082444	no
XLOC_010339	g803	0	0	0	0	1	1	no
XLOC_010340	g10974	10,8687	5,832	-0,898117	-1,13677	0,0539	0,206434	no
XLOC_010341	g10975	26,0892	20,9466	-0,31674	-0,402803	0,48935	0,722076	no
XLOC_010342	g10976	74,72	126,622	0,760965	1,13795	0,0487	0,193148	no
XLOC_010343	g4680	5,63444	2,68685	-1,06836	-1,31484	0,0293	0,139673	no
XLOC_010344	g10977	0	0	0	0	1	1	no
XLOC_010345	g804	10,7444	12,9593	0,270407	0,329862	0,5667	0,775285	no
XLOC_010346	g4681	59,844	93,446	0,642926	1,05242	0,07125	0,245586	no
XLOC_010347	g4682	2294,23	3935,75	0,778624	1,19986	0,03565	0,158051	no
XLOC_010348	g805	118,249	96,3864	-0,294923	-0,473346	0,4067	0,660022	no
XLOC_010349	g807	33,9327	30,7475	-0,142209	-0,198509	0,72045	0,86765	no
XLOC_010350	g810	317,368	437,296	0,462455	0,749832	0,19105	0,441334	no
XLOC_010351	g811	40,0111	36,8375	-0,119224	-0,200096	0,7273	0,870385	no
XLOC_010352	g813	29,5024	80,8677	1,45473	2,3283	0,0001	0,00209829	yes
XLOC_010353	g817	12,2574	20,0618	0,710799	1,07142	0,0637	0,228798	no
XLOC_010354	g819	69,2189	65,6709	-0,0759112	-0,131409	0,81985	0,918071	no
XLOC_010355	g820	127,089	119,598	-0,0876477	-0,147084	0,79635	0,906216	no
XLOC_010356	g822	95,2398	93,534	-0,0260741	-0,0459721	0,936	0,972082	no
XLOC_010357	g824	35,8896	14,4938	-1,30813	-1,95535	0,00085	0,011085	yes
XLOC_010358	g827	36,1526	35,1352	-0,0411816	-0,0705895	0,9025	0,957458	no
XLOC_010359	g828	5,23339	8,77126	0,745037	1,19053	0,0341	0,153466	no
XLOC_010360	g829	7,90619	11,314	0,517051	0,745215	0,1898	0,439584	no
XLOC_010361	g831	31,8816	34,5619	0,116458	0,188393	0,7357	0,874871	no
XLOC_010362	g837	44,8707	44,0252	-0,0274443	-0,0448405	0,93845	0,972415	no
XLOC_010363	g806	52,716	59,9579	0,185708	0,305173	0,59075	0,790268	no
XLOC_010364	g808	79,8224	37,6031	-1,08594	-1,74022	0,0027	0,0261737	yes
XLOC_010365	g809	126,054	69,5214	-0,85851	-1,29425	0,0273	0,133642	no
XLOC_010366	g812	38,2432	33,1981	-0,2041	-0,348042	0,5364	0,755406	no
XLOC_010367	g814	155,918	57,4585	-1,44019	-2,35207	5,00E-05	0,00120049	yes
XLOC_010368	g815	79,8772	47,5789	-0,747461	-1,22982	0,03005	0,141802	no
XLOC_010369	g816	36,8807	49,1937	0,415608	0,704278	0,20365	0,456689	no
XLOC_010370	g818	38,706	36,5018	-0,0845894	-0,139664	0,80595	0,910751	no
XLOC_010371	g821	5,72296	4,55085	-0,330627	-0,429639	0,454	0,698757	no
XLOC_010372	g823	30,0283	24,4778	-0,29485	-0,504329	0,37115	0,629674	no
XLOC_010373	g825	10,0545	12,4216	0,305012	0,429409	0,4523	0,696938	no
XLOC_010374	g826	4,9433	11,6586	1,23785	1,65218	0,0044	0,0374419	yes
XLOC_010375	g830	36,8929	20,1171	-0,874922	-1,39839	0,0166	0,09494	no
XLOC_010376	g832	9,23612	6,24576	-0,564409	-0,746039	0,1959	0,447173	no
XLOC_010377	g833	185,981	198,227	0,0920012	0,149444	0,78905	0,90274	no
XLOC_010378	g834	74,1356	86,457	0,221817	0,217287	0,7133	0,864233	no
XLOC_010379	g835	8,0501	50,1977	2,64054	4,01147	5,00E-05	0,00120049	yes
XLOC_010380	g836	12,4505	22,4046	0,847594	1,18539	0,043	0,177944	no
XLOC_010381	g838	238,107	319,573	0,424537	0,632275	0,2567	0,516345	no
XLOC_010382	g839	113,887	48,7071	-1,2254	-1,80203	0,00255	0,0250912	yes
XLOC_010383	g10978	6,4196	3,30193	-0,959175	-1,04178	0,15015	0,387306	no
XLOC_010384	g10979	24,3562	48,5607	0,995502	1,53728	0,009	0,0618908	no
XLOC_010385	g10980	20,9667	16,2888	-0,364221	-0,498433	0,3753	0,633565	no
XLOC_010386	g4683	10,7195	33,5918	1,64787	2,11527	0,00045	0,00669545	yes
XLOC_010387	g4685	101,532	189,282	0,898608	1,55712	0,0096	0,0644815	no
XLOC_010388	g4686	102,764	112,083	0,125232	0,215258	0,7058	0,860561	no
XLOC_010389	g4687	17,2878	13,679	-0,33779	-0,490003	0,3956	0,649604	no
XLOC_010390	g4689	4,32961	3,72616	-0,21655	-0,278828	0,6244	0,811876	no
XLOC_010391	g4684	8,7189	7,73848	-0,172096	-0,242762	0,6693	0,839037	no
XLOC_010392	g4688	7,17513	5,06385	-0,50277	-0,676107	0,24155	0,499688	no
XLOC_010393	g4690	0	0	0	0	1	1	no
XLOC_010394	g4691	0	0	0	0	1	1	no
XLOC_010395	g10981	0	3,23283	inf	#NAME?	0,00175	0,0188639	yes

XLOC_010396	g10982	0	0	0	0	1	1	no
XLOC_010397	g10983	6,18657	11,6439	0,912361	2,52336	0,5327	0,752895	no
XLOC_010398	g10984	0	0	0	0	1	1	no
XLOC_010399	g10985	0	0	0	0	1	1	no
XLOC_010400	g4692	3,30769	2,79365	-0,243671	-0,298702	0,59965	0,795859	no
XLOC_010401	g4695	20,3132	12,3969	-0,712439	-0,963591	0,0978	0,299843	no
XLOC_010402	g4693	67,0965	66,5845	-0,0110502	-0,0182784	0,9739	0,987624	no
XLOC_010403	g4694	54,472	59,9688	0,138697	0,232502	0,69325	0,853953	no
XLOC_010404	g4696	5,99334	4,57024	-0,39109	-0,404264	0,4829	0,716867	no
XLOC_010405	g4697	262,079	214,46	-0,289295	-0,375976	0,49795	0,727876	no
XLOC_010406	g10986	28,5741	26,5536	-0,105801	-0,154156	0,791	0,903527	no
XLOC_010407	g10987	2,41431	2,11037	-0,194113	-0,178729	0,75225	0,88326	no
XLOC_010408	g4698	21,6694	13,1055	-0,725492	-0,814005	0,15445	0,392419	no
XLOC_010409	g10988	30,1604	41,9033	0,474407	0,78327	0,16155	0,403259	no
XLOC_010410	g10989	12,3913	9,75734	-0,34477	-0,419183	0,44935	0,694572	no
XLOC_010411	g10990	66,5716	43,1718	-0,624818	-0,978557	0,07865	0,261524	no
XLOC_010412	g10991	12,6011	17,3385	0,460429	0,342547	0,7029	0,859051	no
XLOC_010413	g10992	0	0	0	0	1	1	no
XLOC_010414	g4699	62,7102	52,8688	-0,246284	-0,42331	0,4616	0,702668	no
XLOC_010415	g4700	27,0485	14,2619	-0,923381	-1,3162	0,0218	0,115905	no
XLOC_010416	g4702	78,7236	66,3125	-0,247514	-0,400542	0,4675	0,707046	no
XLOC_010417	g4704	39,5419	25,835	-0,614056	-1,02874	0,0698	0,242546	no
XLOC_010418	g4701	88,1632	142,772	0,695462	1,07761	0,05725	0,214333	no
XLOC_010419	g4703	28,6348	31,0751	0,117989	0,194937	0,73865	0,876455	no
XLOC_010420	g4705	20,2689	19,1634	-0,0809133	-0,119793	0,8362	0,925299	no
XLOC_010421	g4706	27,5191	36,7729	0,41821	0,655149	0,23935	0,497337	no
XLOC_010422	g10993	372,073	432,38	0,216712	0,344386	0,5363	0,755406	no
XLOC_010423	g10994	69,9357	71,8878	0,0397169	0,0466979	0,93725	0,972153	no
XLOC_010424	g10995	22,4733	54,7422	1,28444	1,36577	0,02535	0,127857	no
XLOC_010425	g10996	0	0,103007	inf	0	1	1	no
XLOC_010426	g10998	25,5779	9,1106	-1,48928	-2,19107	5,00E-05	0,00120049	yes
XLOC_010427	g10997	66,198	68,3947	0,0470977	0,073804	0,89845	0,95495	no
XLOC_010428	g10999	93,2746	9,46191	-3,30128	-2,74686	0,002	0,0208936	yes
XLOC_010429	g11000	0	0	0	0	1	1	no
XLOC_010430	g11001	0	0	0	0	1	1	no
XLOC_010431	g11002	115,653	38,7972	-1,57578	-1,83678	0,1271	0,351881	no
XLOC_010432	g11003	7,9353	5,67744	-0,483043	-0,520817	0,3761	0,634261	no
XLOC_010433	g4707	0	0,0729999	inf	0	1	1	no
XLOC_010434	g11004	0	0	0	0	1	1	no
XLOC_010435	g4708	0	0	0	0	1	1	no
XLOC_010436	g4709	75,5033	103,994	0,461894	0,796776	0,1666	0,409823	no
XLOC_010437	g4710	79,4397	68,31	-0,217764	-0,370883	0,5071	0,734472	no
XLOC_010438	g4711	79,3665	106,278	0,421239	0,66328	0,24415	0,503031	no
XLOC_010439	g4712	107,708	51,1677	-1,07382	-1,56573	0,00735	0,0538233	no
XLOC_010440	g11005	11,0147	33,6454	1,61098	0,871558	0,12185	0,344534	no
XLOC_010441	g11006	1052,65	22,9804	-5,51749	-5,82829	5,00E-05	0,00120049	yes
XLOC_010442	g840	0,416893	3,21576	2,94741	2,27045	0,00355	0,031924	yes
XLOC_010443	g11007	0	0	0	0	1	1	no
XLOC_010444	g11008	26,1826	41,7199	0,67213	0,770759	0,20225	0,454692	no
XLOC_010445	g841	1,31333	1,88836	0,523898	0,471406	0,4514	0,696316	no
XLOC_010446	g842	102,028	115,894	0,183835	0,298578	0,6074	0,801057	no
XLOC_010447	g11009	18,2608	13,1669	-0,47183	-0,556428	0,3252	0,585849	no
XLOC_010448	g11010	0	0	0	0	1	1	no
XLOC_010449	g4713	2,00692	9,88096	2,29967	1,13632	0,02205	0,116665	no
XLOC_010450	g4714	13,5026	23,2777	0,785708	1,18545	0,04165	0,174624	no
XLOC_010451	g844	0,459263	0,673717	0,552822	0	1	1	no
XLOC_010452	g843	8,56502	18,4323	1,1057	1,70223	0,0058	0,045492	yes
XLOC_010453	g4715	0	0,0213079	inf	0	1	1	no
XLOC_010454	g845	138,879	115,416	-0,266977	-0,41815	0,4875	0,720794	no
XLOC_010455	g11011	6,95409	13,8613	0,995128	1,16425	0,0442	0,180927	no
XLOC_010456	g4716	178,71	109,132	-0,711546	-1,13395	0,0426	0,177109	no
XLOC_010457	g4717	17,4771	12,8232	-0,446709	-0,736395	0,19735	0,449126	no
XLOC_010458	g4720	25,0274	19,7167	-0,344094	-0,450659	0,4311	0,678624	no
XLOC_010459	g4718	61,5288	26,9736	-1,18971	-1,63758	0,0065	0,0492896	yes
XLOC_010460	g4719	47,3847	24,0348	-0,979295	-1,5679	0,0077	0,0555169	no
XLOC_010461	g4721	8,32184	7,22313	-0,204279	-0,262947	0,65325	0,829883	no
XLOC_010462	g11012	56,9464	46,9381	-0,278845	-0,450481	0,4358	0,683059	no
XLOC_010463	g11014	897,027	332,736	-1,43077	-2,35617	5,00E-05	0,00120049	yes
XLOC_010464	g11015	3,65701	2,59736	-0,493618	-0,441408	0,44815	0,694183	no
XLOC_010465	g11013	65,8324	43,0851	-0,611608	-1,02044	0,0682	0,238931	no
XLOC_010466	g11016	17,6819	18,258	0,0462612	0,0594924	0,91565	0,963585	no
XLOC_010467	g11017	22,5505	18,5	-0,285637	-0,356138	0,52755	0,74938	no
XLOC_010468	g11019	4,78671	12,3417	1,36643	1,62562	0,00685	0,0511536	no
XLOC_010469	g11020	12,6413	9,0152	-0,487714	-0,713043	0,2125	0,468106	no
XLOC_010470	g11018	15,2551	21,9348	0,523932	0,592049	0,2917	0,554382	no
XLOC_010471	g4722	0	0	0	0	1	1	no
XLOC_010472	g4723	46,3772	52,0556	0,166638	0,276851	0,618	0,808291	no
XLOC_010473	g11021	88,1489	93,7858	0,0894261	0,152519	0,788	0,902094	no
XLOC_010474	g11022	57,9992	60,3061	0,0562702	0,0889287	0,87365	0,942981	no
XLOC_010475	g11023	55,2119	49,7814	-0,149373	-0,221863	0,6882	0,850292	no
XLOC_010476	g4724	0	0	0	0	1	1	no
XLOC_010477	g11024	46,2091	37,0614	-0,31826	-0,470639	0,39525	0,649464	no
XLOC_010478	g11025	63,6978	60,7757	-0,0677498	-0,11567	0,83915	0,92642	no
XLOC_010479	g11026	96,1767	345,621	1,84543	2,92333	5,00E-05	0,00120049	yes
XLOC_010480	g11027	12,6277	13,0664	0,0492683	0,07196	0,8997	0,955554	no
XLOC_010481	g11028	0	0	0	0	1	1	no
XLOC_010482	g11029	0	0	0	0	1	1	no
XLOC_010483	g4725	0	0	0	0	1	1	no
XLOC_010484	g11030	0,177959	0,164708	-0,111633	0	1	1	no
XLOC_010485	g11031	8,03347	4,21978	-0,928855	-1,35683	0,02945	0,140252	no
XLOC_010486	g4726	2,06712	2,62824	0,346479	0,406791	0,49335	0,72417	no
XLOC_010487	g4727	0	0	0	0	1	1	no

XLOC_010488	g4728	45,859	139,202	1,60191	2,69039	5,00E-05	0,00120049	yes
XLOC_010489	g4729	7,97668	10,2491	0,361631	0,383618	0,5049	0,732907	no
XLOC_010490	g4730	0	0	0	0	1	1	no
XLOC_010491	g11032	6,93438	14,8154	1,09526	1,44582	0,01225	0,0772112	no
XLOC_010492	g4731	0	0	0	0	1	1	no
XLOC_010493	g4732	22,5676	22,5497	-0,00114706	-0,00174471	0,9984	0,998705	no
XLOC_010494	g4733	28,4013	25,597	-0,149985	-0,255006	0,64855	0,827004	no
XLOC_010495	g4735	118,519	351,097	1,56675	2,34907	0,00025	0,00430702	yes
XLOC_010496	g4738	3,39139	0,956759	-1,82565	-1,78721	0,0105	0,06874	no
XLOC_010497	g4739	16,8233	12,7697	-0,397739	-0,574894	0,305	0,566474	no
XLOC_010498	g4734	17,0307	13,9343	-0,289498	-0,433832	0,4476	0,693614	no
XLOC_010499	g4736	16,3388	17,1694	0,0715429	0,106733	0,85175	0,933294	no
XLOC_010500	g4737	21,0265	13,7375	-0,614084	-0,879572	0,12365	0,347009	no
XLOC_010501	g11033	11,3315	4,27593	-1,40603	-1,51857	0,0804	0,265834	no
XLOC_010502	g4741	36,9741	28,865	-0,357195	-0,601736	0,293	0,555336	no
XLOC_010503	g4740	530,387	170,793	-1,63479	-2,692	5,00E-05	0,00120049	yes
XLOC_010504	g4742	4,65804	1,9909	-1,22631	-1,38446	0,0172	0,0974634	no
XLOC_010505	g4743	1,14209	0,331574	-1,78427	-1,68189	0,0091	0,0622298	no
XLOC_010506	g4745	230,229	176,975	-0,379526	-0,548139	0,33885	0,598992	no
XLOC_010507	g4747	16,1766	11,1324	-0,539141	-0,601207	0,4748	0,711946	no
XLOC_010508	g4744	90,6259	149,958	0,726565	1,15175	0,04945	0,195255	no
XLOC_010509	g4746	25,6773	10,6867	-1,26467	-1,75764	0,00405	0,0351645	yes
XLOC_010510	g11034	103,795	121,1	0,222458	0,298022	0,59945	0,795859	no
XLOC_010511	g11035	2,04864	2,65721	0,375243	0,346709	0,55435	0,767694	no
XLOC_010512	g4749	12,289	14,5919	0,247802	0,353382	0,53285	0,752918	no
XLOC_010513	g4748	3,36209	7,15942	1,09048	1,2675	0,03515	0,156826	no
XLOC_010514	g11036	5,09724	14,5214	1,5104	0,932171	0,18285	0,429978	no
XLOC_010515	g11037	0	0	0	0	1	1	no
XLOC_010516	g11038	0,0324397	0	#NAME?	0	1	1	no
XLOC_010517	g11039	0	0	0	0	1	1	no
XLOC_010518	g846	16,197	18,3408	0,179337	0,225655	0,69925	0,857151	no
XLOC_010519	g4750	0	0	0	0	1	1	no
XLOC_010520	g11040	5,07753	63,0767	3,63491	4,76973	5,00E-05	0,00120049	yes
XLOC_010521	g11041	0	0	0	0	1	1	no
XLOC_010522	g4751	3,79475	16,501	2,12047	2,11373	0,0011	0,0134856	yes
XLOC_010523	g4752	3,53934	2,64927	-0,417888	-0,522322	0,37155	0,630087	no
XLOC_010524	g11042	0	0	0	0	1	1	no
XLOC_010525	g11043	49,9872	68,1276	0,44668	0,595262	0,31215	0,573384	no
XLOC_010526	g11044	784,533	890,471	0,182734	0,282865	0,62735	0,813277	no
XLOC_010527	g11045	0	0	0	0	1	1	no
XLOC_010528	g11046	0	0,40735	inf	0	1	1	no
XLOC_010529	g11047	0,105283	0,575692	2,45102	0	1	1	no
XLOC_010530	g11048	25,8076	17,2894	-0,577908	-0,894878	0,11955	0,339989	no
XLOC_010531	g11049	0	0	0	0	1	1	no
XLOC_010532	g11050	0	0	0	0	1	1	no
XLOC_010533	g11051	0	0	0	0	1	1	no
XLOC_010534	g11052	0	0	0	0	1	1	no
XLOC_010535	g11053	0	0	0	0	1	1	no
XLOC_010536	g11054	0	0	0	0	1	1	no
XLOC_010537	g4753	0	0	0	0	1	1	no
XLOC_010538	g11055	131,252	222,597	0,762093	1,08944	0,0558	0,211077	no
XLOC_010539	g4754	0	0	0	0	1	1	no
XLOC_010540	g11056	10,3007	15,6561	0,603986	0,698015	0,23845	0,496518	no
XLOC_010541	g11057	29,24	40,8521	0,482468	0,643289	0,2754	0,537232	no
XLOC_010542	g4755	0	0,0221156	inf	0	1	1	no
XLOC_010543	g4756	0	0	0	0	1	1	no
XLOC_010544	g4757	0	0,516325	inf	0	1	1	no
XLOC_010545	g4758	200,804	109,619	-0,873288	-0,977014	0,0859	0,276731	no
XLOC_010546	g4760	767,998	326,096	-1,2358	-1,80038	0,00135	0,0156148	yes
XLOC_010547	g4764	45,7223	55,9966	0,292441	0,410413	0,4693	0,707556	no
XLOC_010548	g4759	56,2111	60,7669	0,112433	0,190413	0,7396	0,876968	no
XLOC_010549	g4761	56,8179	74,3106	0,387222	0,6497	0,2584	0,51859	no
XLOC_010550	g4762	39,6195	48,6442	0,296059	0,495694	0,3873	0,643156	no
XLOC_010551	g4763	88,2078	71,5108	-0,302745	-0,463547	0,4038	0,65738	no
XLOC_010552	g4765	0,306839	0,542784	0,822897	0	1	1	no
XLOC_010553	g4766	40,1511	25,2227	-0,670713	-1,05706	0,06885	0,240436	no
XLOC_010554	g4768	168,471	181,624	0,10846	0,184557	0,7493	0,881846	no
XLOC_010555	g4767	48,1318	54,4524	0,178003	0,301584	0,5893	0,789593	no
XLOC_010556	g4769	229,701	129,486	-0,826964	-1,36245	0,0186	0,103252	no
XLOC_010557	g4770	83,274	89,2096	0,0993325	0,131197	0,8014	0,908537	no
XLOC_010558	g11058	14,0975	53,3726	1,92066	2,50653	5,00E-05	0,00120049	yes
XLOC_010559	g11059	1,8617	1,59104	-0,226654	-0,253173	0,67185	0,840955	no
XLOC_010560	g11060	45,1936	21,4164	-1,0774	-1,41756	0,01705	0,0968369	no
XLOC_010561	g11061	366,379	120,324	-1,60642	-2,19452	0,0002	0,00367103	yes
XLOC_010562	g4771	0	0	0	0	1	1	no
XLOC_010563	g11062	0	0	0	0	1	1	no
XLOC_010564	g11063	7,25132	5,95449	-0,284266	-0,279935	0,6225	0,810521	no
XLOC_010565	g4773	5,77547	11,504	0,994131	1,12497	0,04925	0,1947	no
XLOC_010566	g4772	10,2203	9,15439	-0,158905	-0,25679	0,65345	0,829883	no
XLOC_010567	g11064	0	0	0	0	1	1	no
XLOC_010568	g11065	0	0	0	0	1	1	no
XLOC_010569	g11066	68,1168	47,3149	-0,525716	-0,860943	0,1332	0,362836	no
XLOC_010570	g4774	86,6306	145,115	0,744246	1,28098	0,02545	0,127967	no
XLOC_010571	g11067	37,1235	18,2735	-1,02258	-1,42299	0,0162	0,0931405	no
XLOC_010572	g11068	102,035	130,478	0,354734	0,521124	0,3716	0,630087	no
XLOC_010573	g4775	0	0	0	0	1	1	no
XLOC_010574	g4776	0	0,0326509	inf	0	1	1	no
XLOC_010575	g11069	105,221	53,5022	-0,975757	-1,3975	0,0149	0,0881965	no
XLOC_010576	g847	32,2594	50,9282	0,658746	0,89874	0,10835	0,319999	no
XLOC_010577	g848	192,806	412,367	1,09678	1,73708	0,00325	0,0301654	yes
XLOC_010578	g849	16,9859	26,9443	0,665638	0,964448	0,0937	0,291921	no
XLOC_010579	g852	21,1733	30,5172	0,527376	0,82832	0,13275	0,362113	no

XLOC_010580	g850	43,8036	37,8067	-0,212409	-0,365488	0,51015	0,736392	no
XLOC_010581	g851	11,2488	13,8957	0,304874	0,415367	0,463	0,704253	no
XLOC_010582	g92	20,5884	27,5115	0,418206	0,696578	0,22035	0,477352	no
XLOC_010583	g4777	16,2866	13,2372	-0,299084	-0,397576	0,496	0,726106	no
XLOC_010584	g4778	8,05462	11,4818	0,511459	0,469831	0,4114	0,663919	no
XLOC_010585	g4779	0	0	0	0	1	1	no
XLOC_010586	g11070	28,1561	25,9107	-0,119904	-0,161828	0,77285	0,894131	no
XLOC_010587	g11071	247,997	193,982	-0,354404	-0,484773	0,4037	0,657326	no
XLOC_010588	g11072	5,61849	10,9903	0,967977	1,13012	0,0539	0,206434	no
XLOC_010589	g4780	42,7466	15,8579	-1,43061	-2,02878	0,00075	0,0100752	yes
XLOC_010590	g11073	4,4936	6,10467	0,442042	0,561517	0,32945	0,590473	no
XLOC_010591	g11074	9,1668	6,39229	-0,520086	-0,702327	0,2246	0,482409	no
XLOC_010592	g11075	0	0	0	0	1	1	no
XLOC_010593	g4781	0	0	0	0	1	1	no
XLOC_010594	g4782	0	0	0	0	1	1	no
XLOC_010595	g11076	42,3863	46,4678	0,132633	0,20811	0,70315	0,859113	no
XLOC_010596	g4783	52,2886	28,6627	-0,867324	-1,35002	0,01965	0,10762	no
XLOC_010597	g4784	19,6034	280,061	3,83656	5,03419	5,00E-05	0,00120049	yes
XLOC_010598	g4785	23,7636	21,2128	-0,163816	-0,265086	0,64705	0,82595	no
XLOC_010599	g4786	216,865	60,1789	-1,84947	-2,89089	5,00E-05	0,00120049	yes
XLOC_010600	g4787	60,5907	87,009	0,522069	0,858722	0,12645	0,351012	no
XLOC_010601	g4788	8,67993	7,5206	-0,206835	-0,256511	0,6465	0,825677	no
XLOC_010602	g4790	3,51134	2,74081	-0,357422	-0,449506	0,41815	0,669829	no
XLOC_010603	g4789	36,5558	37,2914	0,0287428	0,0447848	0,93675	0,972153	no
XLOC_010604	g4791	50,8586	27,0188	-0,912526	-1,3856	0,01635	0,0937285	no
XLOC_010605	g4792	246,332	168,982	-0,543735	-0,934393	0,09765	0,299663	no
XLOC_010606	g4793	20,11	39,4799	0,973209	1,41274	0,01405	0,0848009	no
XLOC_010607	g4794	0	0	0	0	1	1	no
XLOC_010608	g11077	2,80769	1,52655	-0,879105	-0,791831	0,18545	0,4336	no
XLOC_010609	g4795	0	0	0	0	1	1	no
XLOC_010610	g4798	19,3581	15,4099	-0,32908	-0,468774	0,4203	0,671222	no
XLOC_010611	g4799	267,124	252,734	-0,0798903	-0,135761	0,8065	0,910938	no
XLOC_010612	g4801	10,2086	7,01168	-0,541957	-0,706125	0,21975	0,477108	no
XLOC_010613	g4802	3,68623	2,24168	-0,717562	-0,66447	0,24555	0,50414	no
XLOC_010614	g4796	0,873445	0,674766	-0,372329	0	1	1	no
XLOC_010615	g4797	5,53287	4,1191	-0,425697	-0,487517	0,3994	0,653222	no
XLOC_010616	g4800	47,3294	54,95	0,215382	0,349629	0,54535	0,761892	no
XLOC_010617	g11078	6,45833	4,98002	-0,375011	-0,470262	0,4336	0,68051	no
XLOC_010618	g11079	120,369	1016,15	3,07758	4,2762	5,00E-05	0,00120049	yes
XLOC_010619	g4803	3,81127	6,71402	0,816905	1,04727	0,0709	0,244896	no
XLOC_010620	g4804	20,4163	35,99	0,81788	1,27165	0,0224	0,118009	no
XLOC_010621	g4805	327,623	432,467	0,400554	0,544009	0,3384	0,598323	no
XLOC_010622	g11080	20,8097	27,779	0,416736	0,575757	0,3019	0,563717	no
XLOC_010623	g11081	383,982	330,559	-0,216133	-0,320756	0,55915	0,770892	no
XLOC_010624	g11082	24,4156	3,55861	-2,77841	-3,72672	5,00E-05	0,00120049	yes
XLOC_010625	g11083	115,163	43,5866	-1,40172	-2,34515	5,00E-05	0,00120049	yes
XLOC_010626	g4806	34,0042	43,8902	0,368186	0,629528	0,26055	0,521008	no
XLOC_010627	g4807	19,5738	15,2748	-0,357771	-0,592168	0,2841	0,546579	no
XLOC_010628	g4809	284,64	235,441	-0,273772	-0,453324	0,44985	0,694824	no
XLOC_010629	g4815	23,4665	26,0339	0,149785	0,225829	0,69445	0,854574	no
XLOC_010630	g4816	72,949	110,514	0,599263	1,02732	0,073	0,249343	no
XLOC_010631	g4819	7,80016	4,88172	-0,676114	-0,598817	0,4521	0,696739	no
XLOC_010632	g4822	190,116	224,409	0,239252	0,312576	0,58065	0,783024	no
XLOC_010633	g4808	21,1489	23,4957	0,151815	0,25272	0,65875	0,832458	no
XLOC_010634	g4810	24,9844	28,1729	0,17328	0,276064	0,62535	0,812208	no
XLOC_010635	g4811	10,3027	9,66655	-0,0919435	-0,130811	0,81655	0,916563	no
XLOC_010636	g4812	143,822	149,981	0,0605006	0,0843659	0,8757	0,943949	no
XLOC_010637	g4813	26,5207	39,8897	0,588899	0,983264	0,0921	0,289137	no
XLOC_010638	g4814	35,1617	40,6796	0,210301	0,346312	0,5457	0,762272	no
XLOC_010639	g4817	16,5159	32,1825	0,962423	1,52922	0,0069	0,051293	no
XLOC_010640	g4818	41,4274	29,9506	-0,468004	-0,785719	0,17245	0,417829	no
XLOC_010641	g4820	15,7794	16,5661	0,070192	0,109515	0,85095	0,933043	no
XLOC_010642	g4821	3,74751	4,89403	0,385089	0,510444	0,38545	0,641002	no
XLOC_010643	g4823	56,8362	55,4959	-0,0344272	-0,0562138	0,91795	0,964054	no
XLOC_010644	g11084	0	1,25497	inf	0	1	1	no
XLOC_010645	g11085	63,3697	52,6819	-0,266485	-0,426091	0,4579	0,701013	no
XLOC_010646	g11086	577,48	277,582	-1,05686	-1,64371	0,004	0,0348845	yes
XLOC_010647	g4825	948,756	1000,62	0,0767857	0,126022	0,8257	0,920778	no
XLOC_010648	g4824	10,8569	2,33392	-2,21779	-2,28362	0,00115	0,0138564	yes
XLOC_010649	g4826	0	0	0	0	1	1	no
XLOC_010650	g4827	101,409	80,9303	-0,325437	-0,504294	0,37735	0,635496	no
XLOC_010651	g11087	46,1633	26,7428	-0,787598	-1,20164	0,03305	0,150604	no
XLOC_010652	g11088	26,8103	31,0784	0,213125	0,35767	0,5301	0,750804	no
XLOC_010653	g4828	0	0	0	0	1	1	no
XLOC_010654	g11089	0	0	0	0	1	1	no
XLOC_010655	g4829	39,7121	32,0106	-0,311032	-0,526757	0,3594	0,619938	no
XLOC_010656	g4830	2513,77	1849,3	-0,442872	-0,502693	0,3851	0,640945	no
XLOC_010657	g11090	129,054	114,096	-0,17773	-0,260379	0,64065	0,821582	no
XLOC_010658	g4833	1428,22	904,431	-0,659138	-0,762187	0,19955	0,451933	no
XLOC_010659	g4835	5,83577	1,47499	-1,98422	-2,01657	0,00315	0,0293482	yes
XLOC_010660	g4836	1,66748	1,19198	-0,48431	-0,419191	0,4756	0,712433	no
XLOC_010661	g4831	36,0357	46,0658	0,354268	0,529053	0,33445	0,594729	no
XLOC_010662	g4832	53,8406	81,0488	0,590097	0,686479	0,2311	0,488674	no
XLOC_010663	g4834	0,637446	0	#NAME?	0	1	1	no
XLOC_010664	g11091	0	0	0	0	1	1	no
XLOC_010665	g4837	9,29991	5,9422	-0,646219	-0,644395	0,27435	0,536143	no
XLOC_010666	g11092	10,3874	9,63122	-0,10905	-0,145327	0,80125	0,908537	no
XLOC_010667	g11093	448,113	383,548	-0,224456	-0,38479	0,50095	0,730196	no
XLOC_010668	g11094	60,8872	28,7285	-1,08366	-1,60606	0,0062	0,0477522	yes
XLOC_010669	g4839	43,9187	28,7757	-0,609982	-0,980657	0,08565	0,27649	no
XLOC_010670	g4838	12,3706	16,8743	0,447912	0,703731	0,22835	0,486526	no
XLOC_010671	g4840	68,7523	274,974	1,99982	3,0826	5,00E-05	0,00120049	yes

XLOC_010672	g11095	0	0	0	0	1	1	no
XLOC_010673	g4842	28,4046	33,1361	0,222278	0,349001	0,5509	0,765793	no
XLOC_010674	g4841	64,0485	6,77986	-3,23984	-3,86318	5,00E-05	0,00120049	yes
XLOC_010675	g4843	1,91067	4,0919	1,09869	1,24479	0,0333	0,151392	no
XLOC_010676	g4844	0	0	0	0	1	1	no
XLOC_010677	g11096	22,5834	125,698	2,47663	3,3773	5,00E-05	0,00120049	yes
XLOC_010678	g11097	231,623	164,03	-0,497814	-0,809906	0,15175	0,389591	no
XLOC_010679	g4845	4,81788	2,89196	-0,736353	-0,888857	0,13415	0,36391	no
XLOC_010680	g4846	0	0	0	0	1	1	no
XLOC_010681	g4847	74,4292	85,5915	0,201599	0,319989	0,5654	0,774802	no
XLOC_010682	g11098	15,7295	27,2348	0,791978	0,915364	0,13175	0,360285	no
XLOC_010683	g11099	165,379	175,564	0,0862179	0,141889	0,8044	0,909628	no
XLOC_010684	g4848	67,8255	130,331	0,942285	1,55472	0,00705	0,0520143	no
XLOC_010685	g4849	86,5962	70,439	-0,29793	-0,510937	0,37765	0,635674	no
XLOC_010686	g4851	43,2657	50,6588	0,227589	0,364397	0,51995	0,744085	no
XLOC_010687	g4852	17,0789	15,0781	-0,179756	-0,252412	0,6635	0,835468	no
XLOC_010688	g4853	227,551	96,132	-1,2431	-1,96127	0,00085	0,011085	yes
XLOC_010689	g4854	31,3322	22,1373	-0,50117	-0,8406	0,1372	0,369327	no
XLOC_010690	g4855	282,684	260,029	-0,120519	-0,195095	0,7296	0,871721	no
XLOC_010691	g4858	21,1211	13,3923	-0,657282	-1,00217	0,08115	0,267593	no
XLOC_010692	g4859	111,368	103,929	-0,0997326	-0,133479	0,8186	0,917236	no
XLOC_010693	g4860	14,6232	11,1241	-0,394568	-0,574324	0,31655	0,577009	no
XLOC_010694	g4850	21,7751	7,50706	-1,53636	-2,18198	0,0002	0,00367103	yes
XLOC_010695	g4856	36,138	88,4634	1,29156	2,06067	0,00055	0,00788467	yes
XLOC_010696	g4857	36,3833	24,0655	-0,596312	-0,880546	0,1259	0,350039	no
XLOC_010697	g11100	0	0	0	0	1	1	no
XLOC_010698	g4862	103,769	147,591	0,508226	0,798815	0,16815	0,410959	no
XLOC_010699	g4861	6298	4260,61	-0,563834	-0,565971	0,52815	0,749406	no
XLOC_010700	g4863	3,73613	1,07496	-1,79727	-1,95879	0,00125	0,0146655	yes
XLOC_010701	g4864	25,95	31,6203	0,285117	0,460947	0,40795	0,660523	no
XLOC_010702	g4865	241,088	153,032	-0,655723	-1,10132	0,05085	0,198943	no
XLOC_010703	g4866	21,7373	37,6614	0,792916	1,25304	0,02635	0,131016	no
XLOC_010704	g4867	713,801	454,401	-0,651557	-0,62436	0,2942	0,556441	no
XLOC_010705	g11101	0	0	0	0	1	1	no
XLOC_010706	g854	9,40662	2,00779	-2,22807	-2,66228	5,00E-05	0,00120049	yes
XLOC_010707	g853	104,972	95,7578	-0,132537	-0,22573	0,69025	0,851647	no
XLOC_010708	g855	0,0647053	0,156627	1,27538	0	1	1	no
XLOC_010709	g856	22,5948	23,5375	0,0589667	0,100791	0,85585	0,934827	no
XLOC_010710	g11102	0	0	0	0	1	1	no
XLOC_010711	g4868	0	0	0	0	1	1	no
XLOC_010712	g11103	461,307	234,244	-0,977715	-1,53792	0,0069	0,051293	no
XLOC_010713	g11105	81,2021	100,614	0,309239	0,531588	0,34815	0,608658	no
XLOC_010714	g11108	44,3362	49,1031	0,147328	0,250071	0,6558	0,831391	no
XLOC_010715	g11110	24,2042	25,0146	0,0475101	0,0760469	0,89065	0,951189	no
XLOC_010716	g11104	264,578	208,149	-0,346076	-0,590049	0,29685	0,558763	no
XLOC_010717	g11106	79,832	122,842	0,621762	1,02355	0,068	0,238827	no
XLOC_010718	g11107	55,5838	63,8456	0,199923	0,342738	0,5436	0,76125	no
XLOC_010719	g11109	67,6723	72,3615	0,0966565	0,161523	0,7746	0,894738	no
XLOC_010720	g4869	16,0824	18,0747	0,168483	0,26673	0,638	0,81994	no
XLOC_010721	g11111	6,77669	11,8556	0,80692	0,823583	0,1648	0,406924	no
XLOC_010722	g4870	68,6106	103,533	0,593589	0,956923	0,10785	0,319291	no
XLOC_010723	g11112	2152,36	169,178	-3,6693	-3,4306	5,00E-05	0,00120049	yes
XLOC_010724	g11113	0	0	0	0	1	1	no
XLOC_010725	g11114	0	0	0	0	1	1	no
XLOC_010726	g11115	30,4759	22,8938	-0,412714	-0,533194	0,37165	0,630087	no
XLOC_010727	g11116	0,561209	1,2532	1,159	0	1	1	no
XLOC_010728	g11117	0	0	0	0	1	1	no
XLOC_010729	g11118	0,441936	0,897838	1,02262	0	1	1	no
XLOC_010730	g11119	41,4259	53,0874	0,357837	0,571041	0,32055	0,580544	no
XLOC_010731	g11120	27,1224	45,5419	0,747712	1,06432	0,05715	0,214203	no
XLOC_010732	g4871	91,5714	47,753	-0,939306	-1,29853	0,02425	0,12409	no
XLOC_010733	g11121	113,042	139,081	0,299075	0,485209	0,4018	0,655646	no
XLOC_010734	g4872	5,38066	23,9054	2,15148	2,99956	5,00E-05	0,00120049	yes
XLOC_010735	g4873	17,1164	16,9409	-0,0148617	-0,0207371	0,97035	0,98683	no
XLOC_010736	g4874	8,02377	14,9059	0,893531	1,15089	0,04375	0,179985	no
XLOC_010737	g11122	12,5614	11,4176	-0,137744	-0,12957	0,81385	0,915255	no
XLOC_010738	g4875	415,807	249,321	-0,73791	-1,09058	0,06425	0,229955	no
XLOC_010739	g4876	0,317491	1,42251	2,16365	1,46175	0,046	0,185789	no
XLOC_010740	g4877	0,391262	1,85091	2,24203	2,27397	0,00045	0,00669545	yes
XLOC_010741	g11123	50,5718	54,0173	0,0950892	0,160351	0,77025	0,893264	no
XLOC_010742	g4878	8,89284	9,60613	0,111311	0,157682	0,7893	0,90274	no
XLOC_010743	g11124	34,7749	29,1814	-0,252999	-0,416118	0,4693	0,707556	no
XLOC_010744	g11126	43,7077	50,5729	0,210475	0,353839	0,5393	0,758039	no
XLOC_010745	g11127	126,852	140,942	0,151955	0,241528	0,6738	0,8415	no
XLOC_010746	g11125	86,632	77,4512	-0,161612	-0,280236	0,62685	0,812951	no
XLOC_010747	g4880	0	0	0	0	1	1	no
XLOC_010748	g4879	1,26228	0,697433	-0,85591	-0,788532	0,1729	0,4183	no
XLOC_010749	g4881	0,0509232	0,309957	2,60567	0	1	1	no
XLOC_010750	g4882	82,58	87,5566	0,0844231	0,14584	0,7981	0,907108	no
XLOC_010751	g4883	122,5	120,633	-0,0221617	-0,034792	0,95115	0,978553	no
XLOC_010752	g4884	8,98191	1,30908	-2,77847	-3,17898	5,00E-05	0,00120049	yes
XLOC_010753	g11128	0	0	0	0	1	1	no
XLOC_010754	g11129	239,791	204,555	-0,229292	-0,377157	0,514	0,740425	no
XLOC_010755	g11130	101,15	95,0598	-0,0895903	-0,155277	0,78925	0,90274	no
XLOC_010756	g11131	16,7413	21,5069	0,361392	0,507777	0,38535	0,640945	no
XLOC_010757	g11132	0	0	0	0	1	1	no
XLOC_010758	g4886	89,5442	109,68	0,29263	0,482381	0,4114	0,663919	no
XLOC_010759	g4889	54,2566	60,9019	0,166687	0,285209	0,61245	0,805014	no
XLOC_010760	g4891	53,5544	60,1071	0,166529	0,269151	0,62355	0,811243	no
XLOC_010761	g4893	45,2074	46,1177	0,0287612	0,0464651	0,93505	0,971715	no
XLOC_010762	g4894	45,2021	57,0787	0,336562	0,5688	0,3319	0,592663	no
XLOC_010763	g4897	36,1133	41,3003	0,193621	0,331384	0,55985	0,771178	no

XLOC_010764	g4885	232,448	269,45	0,213108	0,362514	0,5254	0,74731	no
XLOC_010765	g4887	16,7421	21,5231	0,362406	0,544495	0,3356	0,595606	no
XLOC_010766	g4888	31,7262	44,1221	0,475827	0,799054	0,1463	0,383111	no
XLOC_010767	g4890	49,511	46,342	-0,0954274	-0,160207	0,77855	0,896711	no
XLOC_010768	g4892	39,7651	28,5516	-0,477931	-0,785394	0,16375	0,405862	no
XLOC_010769	g4895	80,8129	52,0618	-0,634362	-1,08279	0,05435	0,20743	no
XLOC_010770	g4896	340,769	286,674	-0,249382	-0,418216	0,4591	0,701036	no
XLOC_010771	g4898	51,1204	60,3765	0,240088	0,41353	0,46	0,701766	no
XLOC_010772	g4899	11,9928	16,6624	0,474432	0,764448	0,1879	0,436832	no
XLOC_010773	g4901	118,34	204,007	0,785678	1,10615	0,05145	0,200095	no
XLOC_010774	g4900	78,5179	114,26	0,541223	0,868337	0,14035	0,37381	no
XLOC_010775	g11133	0	0	0	0	1	1	no
XLOC_010776	g11134	100,904	58,5588	-0,78502	-0,713705	0,2443	0,503031	no
XLOC_010777	g4902	33,8369	20,263	-0,73975	-1,24547	0,0299	0,141434	no
XLOC_010778	g4903	15,1395	31,147	1,04078	1,69924	0,0044	0,0374419	yes
XLOC_010779	g4904	56,4902	112,213	0,99017	1,61934	0,0054	0,0429724	yes
XLOC_010780	g4906	2112,23	3562,62	0,754175	1,16043	0,0492	0,1947	no
XLOC_010781	g4907	31,6627	31,0126	-0,0299302	-0,0507954	0,9279	0,968021	no
XLOC_010782	g4905	157,976	223,68	0,501727	0,690729	0,2298	0,487612	no
XLOC_010783	g4908	125,19	140,109	0,162426	0,277665	0,6217	0,809909	no
XLOC_010784	g4910	25,5946	13,9623	-0,874299	-1,30196	0,02185	0,116108	no
XLOC_010785	g4909	46,4191	24,5724	-0,917677	-1,4982	0,00785	0,0561859	no
XLOC_010786	g11135	52,3529	49,3727	-0,084556	-0,14522	0,7982	0,907108	no
XLOC_010787	g11136	7,49974	16,2083	1,11182	1,23566	0,1158	0,333868	no
XLOC_010788	g11137	498,645	617,477	0,308373	0,520191	0,35205	0,612641	no
XLOC_010789	g4913	12,0903	24,3153	1,00801	1,54306	0,00955	0,0643217	no
XLOC_010790	g4915	1206,28	1093,79	-0,141222	-0,18618	0,7471	0,881052	no
XLOC_010791	g4911	38,4713	43,2296	0,168236	0,28055	0,6241	0,811851	no
XLOC_010792	g4912	5,21117	5,9618	0,194141	0,238746	0,66305	0,835468	no
XLOC_010793	g4914	12,6895	11,4671	-0,146138	-0,195704	0,7339	0,874259	no
XLOC_010794	g11138	35,9725	34,5966	-0,0562633	-0,0762788	0,8922	0,951863	no
XLOC_010795	g4917	131,129	144,777	0,142842	0,247118	0,6795	0,845777	no
XLOC_010796	g4916	135,582	100,956	-0,42544	-0,585325	0,3009	0,563041	no
XLOC_010797	g4918	164,12	46,1882	-1,82916	-2,54239	5,00E-05	0,00120049	yes
XLOC_010798	g857	8,5239	7,11039	-0,261585	-0,347423	0,5534	0,767027	no
XLOC_010799	g11139	0	0	0	0	1	1	no
XLOC_010800	g11140	1,45871	2,30884	0,662482	0,57747	0,31435	0,575151	no
XLOC_010801	g11141	9,90325	11,36	0,197993	0,262432	0,6402	0,821262	no
XLOC_010802	g4919	0	0	0	0	1	1	no
XLOC_010803	g4920	0	0	0	0	1	1	no
XLOC_010804	g4921	0	0	0	0	1	1	no
XLOC_010805	g4922	0	0	0	0	1	1	no
XLOC_010806	g11142	0	0	0	0	1	1	no
XLOC_010807	g11143	26,0661	67,1662	1,36556	2,09808	0,0004	0,0061761	yes
XLOC_010808	g11144	612,411	580,687	-0,0767403	-0,0898015	0,8748	0,9436	no
XLOC_010809	g4924	4,62585	3,50342	-0,400956	-0,488004	0,38195	0,638425	no
XLOC_010810	g4923	41,9887	33,5953	-0,321741	-0,418074	0,46675	0,706892	no
XLOC_010811	g4925	30,2144	29,1873	-0,049896	-0,0868878	0,8814	0,947078	no
XLOC_010812	g4926	8,24638	2,22004	-1,89318	-2,29177	0,0001	0,00209829	yes
XLOC_010813	g4927	47,4836	55,3969	0,222375	0,376799	0,50085	0,730196	no
XLOC_010814	g859	0	0	0	0	1	1	no
XLOC_010815	g858	11,7336	8,96	-0,389069	-0,394889	0,49015	0,722497	no
XLOC_010816	g860	3,99887	3,65457	-0,129891	-0,111287	0,84705	0,930483	no
XLOC_010817	g11145	0	0	0	0	1	1	no
XLOC_010818	g11146	27,5605	27,6876	0,00663761	0,0092916	0,9874	0,993619	no
XLOC_010819	g4929	151,773	105,266	-0,527879	-0,820558	0,1518	0,389617	no
XLOC_010820	g4928	261,365	138,615	-0,91498	-1,47371	0,01045	0,068504	no
XLOC_010821	g4930	0,126395	0,251396	0,992017	0	1	1	no
XLOC_010822	g4931	23,4841	20,1087	-0,223867	-0,33242	0,5631	0,772944	no
XLOC_010823	g4932	30,3663	29,4496	-0,0442223	-0,0746654	0,89685	0,954178	no
XLOC_010824	g11147	0,766528	0,725619	-0,0791261	0	1	1	no
XLOC_010825	g11148	45,7934	47,4657	0,0517474	0,0718836	0,89725	0,954356	no
XLOC_010826	g11149	18,5373	27,0778	0,546678	0,7156	0,2299	0,487612	no
XLOC_010827	g4933	6,77794	5,28503	-0,358935	-0,464798	0,42245	0,672657	no
XLOC_010828	g4934	0,680782	1,18276	0,796888	0,838255	0,15265	0,390472	no
XLOC_010829	g4935	26,4044	41,006	0,635057	0,794481	0,17	0,41373	no
XLOC_010830	g4936	0	0	0	0	1	1	no
XLOC_010831	g4939	65,1531	71,4547	0,133195	0,229111	0,6817	0,846629	no
XLOC_010832	g4941	59,8323	69,6257	0,218694	0,348577	0,54115	0,759699	no
XLOC_010833	g4943	36,5286	35,6283	-0,0360042	-0,0568565	0,91825	0,964054	no
XLOC_010834	g4944	92,8414	174,15	0,907494	1,50568	0,00695	0,0515476	no
XLOC_010835	g4946	37,3828	39,4373	0,077187	0,114962	0,83635	0,925299	no
XLOC_010836	g4948	129,71	100,714	-0,365035	-0,624958	0,28325	0,546037	no
XLOC_010837	g4949	165,537	101,444	-0,706471	-0,977712	0,09935	0,302236	no
XLOC_010838	g4952	51,2798	39,1787	-0,388323	-0,666783	0,24835	0,507023	no
XLOC_010839	g4954	125,879	145,099	0,204995	0,322614	0,5773	0,781041	no
XLOC_010840	g4958	905,777	5094,37	2,49168	3,65461	5,00E-05	0,00120049	yes
XLOC_010841	g4960	121,743	92,7239	-0,392822	-0,605504	0,28165	0,543914	no
XLOC_010842	g4937	19,9258	22,9003	0,200725	0,326832	0,55905	0,770892	no
XLOC_010843	g4938	106,277	137,235	0,368823	0,629392	0,28075	0,54302	no
XLOC_010844	g4940	38,6563	48,5175	0,327802	0,528271	0,33455	0,594729	no
XLOC_010845	g4942	75,8421	63,5262	-0,255648	-0,436824	0,4348	0,68174	no
XLOC_010846	g4945	77,6072	93,5858	0,270099	0,469244	0,41245	0,66501	no
XLOC_010847	g4947	69,0772	80,3962	0,218917	0,357144	0,5382	0,757073	no
XLOC_010848	g4950	292,178	209,258	-0,481566	-0,75466	0,1812	0,428871	no
XLOC_010849	g4951	90,5093	106,692	0,237309	0,393641	0,49295	0,724054	no
XLOC_010850	g4953	137,836	164,956	0,259128	0,427574	0,4515	0,696361	no
XLOC_010851	g4955	573,658	700,879	0,288973	0,470776	0,4187	0,670084	no
XLOC_010852	g4956	36,3556	30,6209	-0,247659	-0,391706	0,4878	0,720981	no
XLOC_010853	g4957	116,145	234,174	1,01165	1,72713	0,00415	0,0357482	yes
XLOC_010854	g4959	26,3127	31,7786	0,272295	0,388272	0,4783	0,713924	no
XLOC_010855	g4961	29,2447	38,5559	0,398775	0,607908	0,27165	0,532988	no

XLOC_010856	g11150	0	1,91959	inf	0	1	1	no
XLOC_010857	g11151	32,2999	28,0564	-0,203197	-0,275988	0,60705	0,800918	no
XLOC_010858	g11152	12,9928	6,45678	-1,00883	-1,35722	0,02205	0,116665	no
XLOC_010859	g4962	16,7069	8,1154	-1,04171	-1,45182	0,01365	0,0831532	no
XLOC_010860	g4964	390,634	284,614	-0,456812	-0,708756	0,2222	0,479456	no
XLOC_010861	g4963	29,6929	29,0088	-0,0336281	-0,0526381	0,92885	0,968394	no
XLOC_010862	g11153	0	0	0	0	1	1	no
XLOC_010863	g4965	0	0	0	0	1	1	no
XLOC_010864	g4966	0	0,00655276	inf	0	1	1	no
XLOC_010865	g11154	0	9,82798	inf	0	1	1	no
XLOC_010866	g4968	254,654	502,643	0,980994	1,5467	0,00685	0,0511536	no
XLOC_010867	g4967	174,11	134,984	-0,36721	-0,63301	0,261	0,521008	no
XLOC_010868	g11155	12,4942	26,3506	1,07657	1,39285	0,01235	0,0774438	no
XLOC_010869	g11156	0	0	0	0	1	1	no
XLOC_010870	g11157	12,4966	24,466	0,969242	1,12578	0,067	0,23599	no
XLOC_010871	g11158	86,2591	66,8989	-0,366695	-0,588385	0,3063	0,567308	no
XLOC_010872	g11159	30,0613	189,6	2,65697	4,02466	5,00E-05	0,00120049	yes
XLOC_010873	g4970	16,8741	21,6024	0,356378	0,521101	0,3708	0,629539	no
XLOC_010874	g4972	2,38731	6,33551	1,40807	1,95678	0,0008	0,0106019	yes
XLOC_010875	g4974	105,104	94,9022	-0,147306	-0,238504	0,6654	0,836176	no
XLOC_010876	g4976	54,4135	46,0117	-0,241966	-0,403428	0,4848	0,718602	no
XLOC_010877	g4977	80,7878	90,9474	0,170895	0,25893	0,64925	0,827531	no
XLOC_010878	g4978	67,8531	48,905	-0,472433	-0,812904	0,1553	0,392851	no
XLOC_010879	g4979	91,1138	84,05	-0,116421	-0,189364	0,7425	0,878158	no
XLOC_010880	g4969	53,9554	60,2078	0,158183	0,275028	0,6316	0,816117	no
XLOC_010881	g4971	2,86746	2,44093	-0,232341	-0,292456	0,6137	0,805977	no
XLOC_010882	g4973	91,7965	51,0001	-0,847939	-1,25796	0,02175	0,11589	no
XLOC_010883	g4975	40,9518	56,2892	0,458931	0,593875	0,30595	0,567194	no
XLOC_010884	g4980	83,4212	115,615	0,470838	0,773204	0,1804	0,427595	no
XLOC_010885	g11160	37,1161	33,806	-0,134766	-0,168572	0,7664	0,890609	no
XLOC_010886	g4981	11,7877	5,74846	-1,03604	-0,867365	0,12425	0,347518	no
XLOC_010887	g4982	4,16455	2,56545	-0,69895	-0,715711	0,2202	0,477349	no
XLOC_010888	g4983	9,21694	12,4554	0,434414	0,659609	0,2301	0,487612	no
XLOC_010889	g4985	0	0	0	0	1	1	no
XLOC_010890	g4984	0	0	0	0	1	1	no
XLOC_010891	g862	139,522	134,014	-0,0581077	-0,0996741	0,85745	0,935212	no
XLOC_010892	g863	20,4542	9,75026	-1,06888	-1,43489	0,01185	0,075124	no
XLOC_010893	g864	5,8473	4,59852	-0,346601	-0,501703	0,37215	0,630523	no
XLOC_010894	g861	38,0581	41,8173	0,135896	0,220762	0,70885	0,861925	no
XLOC_010895	g11161	0	0,420587	inf	0	1	1	no
XLOC_010896	g11162	0	0	0	0	1	1	no
XLOC_010897	g11163	0	0	0	0	1	1	no
XLOC_010898	g4986	156,218	189,672	0,279949	0,415337	0,4611	0,702451	no
XLOC_010899	g11164	0,248918	0,225779	-0,140759	0	1	1	no
XLOC_010900	g4987	0	0,0839901	inf	0	1	1	no
XLOC_010901	g11165	298,408	292,482	-0,0289406	-0,0392782	0,94395	0,975337	no
XLOC_010902	g4988	164,372	28,6869	-2,5185	-2,71062	5,00E-05	0,00120049	yes
XLOC_010903	g4989	52,2413	142,586	1,44857	2,24358	0,0001	0,00209829	yes
XLOC_010904	g4990	74,9192	37,0363	-1,0164	-1,40071	0,015	0,0885749	no
XLOC_010905	g11166	44,7558	44,1224	-0,020565	-0,0325629	0,95275	0,978815	no
XLOC_010906	g11167	8,08855	10,6727	0,399974	0,358259	0,5463	0,762785	no
XLOC_010907	g11168	62,2489	50,2206	-0,309767	-0,518459	0,3527	0,613315	no
XLOC_010908	g11169	0	0	0	0	1	1	no
XLOC_010909	g93	607,667	1450,35	1,25505	1,94802	0,0011	0,0134856	yes
XLOC_010910	g94	61,8942	58,756	-0,0750672	-0,127097	0,82345	0,919418	no
XLOC_010911	g96	21,2358	24,1137	0,183355	0,289191	0,61815	0,808291	no
XLOC_010912	g99	22,8175	31,9746	0,486787	0,777754	0,1748	0,420617	no
XLOC_010913	g101	75,6388	249,971	1,72456	2,42833	5,00E-05	0,00120049	yes
XLOC_010914	g102	71,9035	75,6736	0,0737283	0,124977	0,82895	0,921681	no
XLOC_010915	g104	99,276	159,583	0,684786	1,07435	0,06555	0,232467	no
XLOC_010916	g107	32,3491	51,7708	0,678414	1,15542	0,0424	0,176802	no
XLOC_010917	g110	68,355	84,2231	0,30117	0,515904	0,36425	0,62392	no
XLOC_010918	g112	3334,08	2809,72	-0,246864	-0,283284	0,6147	0,806352	no
XLOC_010919	g95	67,7907	118,723	0,808438	1,38622	0,0159	0,0920625	no
XLOC_010920	g97	11,5461	9,23877	-0,321634	-0,461385	0,41315	0,665649	no
XLOC_010921	g98	80,5533	85,9298	0,0932139	0,119396	0,83405	0,924164	no
XLOC_010922	g100	85,945	117,662	0,453164	0,760705	0,1793	0,426373	no
XLOC_010923	g103	2062,68	3202,58	0,634715	0,839623	0,13135	0,359392	no
XLOC_010924	g105	70,1845	49,8074	-0,494794	-0,82728	0,1437	0,379469	no
XLOC_010925	g106	77,3808	107,879	0,479371	0,810748	0,15405	0,39191	no
XLOC_010926	g108	83,8424	186,678	1,1548	1,61519	0,00395	0,0345713	yes
XLOC_010927	g109	86,2741	104,927	0,282392	0,406865	0,4701	0,708034	no
XLOC_010928	g111	175,523	193,34	0,139479	0,233088	0,67405	0,841661	no
XLOC_010929	g11170	34,8525	26,2852	-0,407016	-0,572063	0,31275	0,573414	no
XLOC_010930	g4991	1,98956	2,54463	0,355005	0,486309	0,38755	0,643188	no
XLOC_010931	g4993	9,95279	8,64824	-0,202694	-0,281813	0,6244	0,811876	no
XLOC_010932	g4992	18,6344	30,5827	0,714745	0,824225	0,1532	0,391294	no
XLOC_010933	g4994	735,189	1516,7	1,04475	1,74923	0,00195	0,0205021	yes
XLOC_010934	g4995	0,0546019	0	#NAME?	0	1	1	no
XLOC_010935	g11171	0	0	0	0	1	1	no
XLOC_010936	g4996	22,1771	34,3032	0,629271	1,04184	0,0747	0,252341	no
XLOC_010937	g4997	0,579866	0,195002	-1,57223	0	1	1	no
XLOC_010938	g4998	0	0	0	0	1	1	no
XLOC_010939	g11172	1,55778	4,47897	1,52368	1,00494	0,35635	0,61704	no
XLOC_010940	g5000	0	0	0	0	1	1	no
XLOC_010941	g5001	0	0	0	0	1	1	no
XLOC_010942	g5002	0	0	0	0	1	1	no
XLOC_010943	g4999	0	0	0	0	1	1	no
XLOC_010944	g11173	119,099	240,677	1,01494	1,14875	0,0576	0,214906	no
XLOC_010945	g11174	909,564	930,517	0,032857	0,0546066	0,92315	0,96635	no
XLOC_010946	g11175	0,0348519	0	#NAME?	0	1	1	no
XLOC_010947	g5003	0	0	0	0	1	1	no

XLOC_010948	g5004	0	0	0	0	1	1	no
XLOC_010949	g11176	793,222	614,204	-0,369007	-0,62872	0,279	0,54071	no
XLOC_010950	g11177	89,1833	79,1102	-0,17291	-0,267255	0,6343	0,817002	no
XLOC_010951	g11178	0	0	0	0	1	1	no
XLOC_010952	g11179	0	0	0	0	1	1	no
XLOC_010953	g5006	7,37149	16,984	1,20415	1,56392	0,00765	0,0553596	no
XLOC_010954	g5008	2,40238	6,78808	1,49854	1,78255	0,00355	0,031924	yes
XLOC_010955	g5005	29,4976	22,869	-0,367204	-0,578981	0,3073	0,568181	no
XLOC_010956	g5007	10,23	22,0185	1,10592	1,80823	0,00175	0,0188639	yes
XLOC_010957	g11180	6,54994	0	#NAME?	0	1	1	no
XLOC_010958	g5009	171,381	188,767	0,139403	0,150778	0,7674	0,891397	no
XLOC_010959	g5010	31,8476	129,815	2,0272	3,32987	5,00E-05	0,00120049	yes
XLOC_010960	g5013	0,343414	0,158705	-1,1136	0	1	1	no
XLOC_010961	g5015	12,0507	9,59466	-0,328813	-0,485544	0,37845	0,636208	no
XLOC_010962	g5017	33,9467	31,5245	-0,1068	-0,145416	0,78815	0,90216	no
XLOC_010963	g5011	0,295931	0,104458	-1,50234	0	1	1	no
XLOC_010964	g5012	60,2821	151,86	1,33294	2,17122	0,00035	0,00552572	yes
XLOC_010965	g5014	252,906	109,804	-1,20367	-1,89101	0,00105	0,0130684	yes
XLOC_010966	g5016	33,8643	24,1763	-0,486171	-0,751563	0,182	0,429625	no
XLOC_010967	g5018	10,8706	7,99333	-0,443561	-0,621485	0,2571	0,517043	no
XLOC_010968	g5019	0	0	0	0	1	1	no
XLOC_010969	g11181	13,2142	74,0059	2,48556	3,11705	5,00E-05	0,00120049	yes
XLOC_010970	g867	214,993	182,217	-0,238635	-0,378611	0,49895	0,728578	no
XLOC_010971	g869	32,4607	95,9383	1,56341	2,17202	0,0003	0,00496796	yes
XLOC_010972	g872	23,3145	37,2922	0,677645	1,14063	0,05265	0,203552	no
XLOC_010973	g874	4,67237	3,24392	-0,526415	-0,694894	0,22955	0,487604	no
XLOC_010974	g879	7,71314	12,1031	0,649991	0,681667	0,2475	0,506238	no
XLOC_010975	g880	90,9654	69,1924	-0,394705	-0,681777	0,23055	0,487721	no
XLOC_010976	g881	7,88819	10,8826	0,464263	0,689367	0,21755	0,474223	no
XLOC_010977	g883	111,329	56,5276	-0,977802	-1,62156	0,0047	0,03928	yes
XLOC_010978	g884	3,202	3,91384	0,28961	0,38205	0,4997	0,729131	no
XLOC_010979	g865	102,29	103,808	0,0212452	0,0361638	0,94805	0,976439	no
XLOC_010980	g866	26,1142	16,9653	-0,622245	-1,01473	0,07365	0,250691	no
XLOC_010981	g868	36,2213	31,0557	-0,22198	-0,357132	0,5283	0,749406	no
XLOC_010982	g870	34,3539	104,089	1,59928	2,53017	5,00E-05	0,00120049	yes
XLOC_010983	g871	33,8699	40,6763	0,264184	0,438224	0,43445	0,6813	no
XLOC_010984	g873	32,6757	63,3469	0,955057	1,55068	0,0087	0,0603347	no
XLOC_010985	g875	8,85161	8,81988	-0,0051797	-0,00718162	0,9933	0,99614	no
XLOC_010986	g876	16,8096	9,24016	-0,863298	-1,03742	0,0787	0,261524	no
XLOC_010987	g877	6,68413	9,62372	0,525854	0,807606	0,1464	0,383271	no
XLOC_010988	g878	25,9207	40,085	0,628954	0,941102	0,10195	0,307384	no
XLOC_010989	g882	73,3866	93,274	0,34596	0,595425	0,2964	0,558451	no
XLOC_010990	g11182	0	0	0	0	1	1	no
XLOC_010991	g11183	50,5438	55,4741	0,134281	0,218516	0,69625	0,855717	no
XLOC_010992	g11185	147,787	294,199	0,993268	1,63602	0,00485	0,0400901	yes
XLOC_010993	g11184	252,687	280,537	0,150836	0,246612	0,65755	0,832081	no
XLOC_010994	g11187	85,072	231,587	1,4448	2,3627	5,00E-05	0,00120049	yes
XLOC_010995	g11189	73,6248	64,6521	-0,187494	-0,32309	0,57545	0,780604	no
XLOC_010996	g11190	67,6167	82,0629	0,27935	0,447413	0,42105	0,671517	no
XLOC_010997	g11191	24,0362	35,2659	0,553063	0,890889	0,13435	0,364251	no
XLOC_010998	g11186	139,713	313,85	1,16761	1,44822	0,01555	0,0907314	no
XLOC_010999	g11188	7,17107	4,90772	-0,547137	-0,702495	0,221	0,477812	no
XLOC_011000	g11192	214,106	2877,16	3,74825	4,70688	5,00E-05	0,00120049	yes
XLOC_011001	g5021	27,802	7,21188	-1,94674	-2,4575	0,0001	0,00209829	yes
XLOC_011002	g5023	90,9429	30,885	-1,55805	-2,36845	0,0001	0,00209829	yes
XLOC_011003	g5025	50,4162	14,8506	-1,76337	-2,36346	5,00E-05	0,00120049	yes
XLOC_011004	g5020	23,7679	12,4797	-0,929434	-1,39153	0,0139	0,084206	no
XLOC_011005	g5022	28,2261	25,351	-0,154986	-0,245547	0,6635	0,835468	no
XLOC_011006	g5024	37,0985	34,2216	-0,116453	-0,187292	0,7276	0,870385	no
XLOC_011007	g5026	283,517	28,4893	-3,31494	-4,72975	5,00E-05	0,00120049	yes
XLOC_011008	g5028	0	0	0	0	1	1	no
XLOC_011009	g5029	0	0	0	0	1	1	no
XLOC_011010	g5027	0	0	0	0	1	1	no
XLOC_011011	g11193	215,448	151,328	-0,509661	-0,744147	0,16745	0,410802	no
XLOC_011012	g11194	499,366	600,664	0,26646	0,391974	0,49	0,722384	no
XLOC_011013	g5031	16,161	27,5779	0,770995	1,23072	0,034	0,153226	no
XLOC_011014	g5030	46,6832	44,4207	-0,0716736	-0,11958	0,83925	0,92642	no
XLOC_011015	g11195	3,05353	1,54197	-0,985705	-0,86975	0,1471	0,383977	no
XLOC_011016	g11196	64,9729	18,5986	-1,80464	-1,46683	0,0252	0,127231	no
XLOC_011017	g886	404,049	273,548	-0,562736	-0,781125	0,16325	0,405236	no
XLOC_011018	g888	10,3525	17,1297	0,726524	1,16374	0,0403	0,170801	no
XLOC_011019	g885	3131,81	4288,57	0,4535	0,592613	0,3003	0,562226	no
XLOC_011020	g887	32,0325	34,3178	0,0994187	0,124824	0,82985	0,922056	no
XLOC_011021	g889	52,2089	62,368	0,25651	0,434557	0,44405	0,690401	no
XLOC_011022	g890	43,2048	34,9867	-0,304382	-0,447461	0,43905	0,685309	no
XLOC_011023	g891	712,463	1014,6	0,51003	0,842152	0,1469	0,383863	no
XLOC_011024	g11197	0	0	0	0	1	1	no
XLOC_011025	g11198	267,092	339,691	0,346886	0,558769	0,3356	0,595606	no
XLOC_011026	g11199	0	0	0	0	1	1	no
XLOC_011027	g11200	106,887	76,9732	-0,473659	-0,80168	0,16015	0,401294	no
XLOC_011028	g11201	21,3145	13,8998	-0,616774	-0,908575	0,10185	0,307365	no
XLOC_011029	g5032	0	0	0	0	1	1	no
XLOC_011030	g11202	42,0562	65,8225	0,646262	1,082	0,0523	0,202678	no
XLOC_011031	g11203	0	0	0	0	1	1	no
XLOC_011032	g11204	0	0	0	0	1	1	no
XLOC_011033	g11205	55,2245	55,8135	0,0153037	0,0246403	0,96295	0,983686	no
XLOC_011034	g11206	0	0,351121	inf	0	1	1	no
XLOC_011035	g11207	0	0	0	0	1	1	no
XLOC_011036	g5033	3,03149	6,95695	1,19843	1,21336	0,04355	0,179539	no
XLOC_011037	g5034	19,207	17,5577	-0,129535	-0,211184	0,71615	0,865564	no
XLOC_011038	g5036	143,849	49,0023	-1,55363	-2,15985	0,00035	0,00552572	yes
XLOC_011039	g5037	59,2153	16,664	-1,82924	-2,40665	5,00E-05	0,00120049	yes

XLOC_011040	g5035	68,1144	13,7548	-2,30803	-3,16943	5,00E-05	0,00120049	yes
XLOC_011041	g5038	0	0	0	0	1	1	no
XLOC_011042	g5039	2,10544	2,52622	0,262862	0,329092	0,56875	0,777007	no
XLOC_011043	g5041	46,6072	171,049	1,87578	2,70256	5,00E-05	0,00120049	yes
XLOC_011044	g5040	34,2459	5,59835	-2,61286	-2,73126	0,0001	0,00209829	yes
XLOC_011045	g11208	88,2382	73,1426	-0,270691	-0,456353	0,4269	0,676561	no
XLOC_011046	g11209	30,603	30,8819	0,0130903	0,0187833	0,97215	0,987026	no
XLOC_011047	g11210	9,17209	16,0362	0,806009	1,12927	0,05155	0,200325	no
XLOC_011048	g892	4,20995	24,4091	2,53554	3,10917	5,00E-05	0,00120049	yes
XLOC_011049	g893	123,746	160,204	0,372525	0,594816	0,29665	0,558695	no
XLOC_011050	g5043	58,166	57,2363	-0,0232464	-0,0390752	0,9471	0,97628	no
XLOC_011051	g5042	90,6063	77,0809	-0,233239	-0,396772	0,49415	0,724879	no
XLOC_011052	g5044	1032,55	1013	-0,0275708	-0,0434077	0,9378	0,972153	no
XLOC_011053	g11211	412,679	131,45	-1,65051	-1,75505	0,00615	0,0475161	yes
XLOC_011054	g11212	26,3499	49,0649	0,896896	1,27748	0,03545	0,157663	no
XLOC_011055	g11213	301,43	177,926	-0,760549	-1,18057	0,0368	0,161185	no
XLOC_011056	g5046	6,04709	4,22486	-0,517338	-0,687773	0,2252	0,48338	no
XLOC_011057	g5045	17,9221	10,4239	-0,781843	-0,989169	0,09465	0,293855	no
XLOC_011058	g5047	0	0	0	0	1	1	no
XLOC_011059	g5048	0	0	0	0	1	1	no
XLOC_011060	g11214	24,3558	51,8133	1,08906	1,66535	0,00525	0,0423624	yes
XLOC_011061	g11215	108,442	228,388	1,07456	1,49948	0,00595	0,0463354	yes
XLOC_011062	g5051	23,8434	16,339	-0,545274	-0,878885	0,13405	0,363839	no
XLOC_011063	g5053	94,1146	119,441	0,343807	0,567599	0,31485	0,575652	no
XLOC_011064	g5056	61,7568	68,8354	0,156553	0,250339	0,6472	0,82599	no
XLOC_011065	g5058	42,8433	94,2451	1,13735	1,59486	0,005	0,0410192	yes
XLOC_011066	g5060	89,3977	37,4757	-1,25428	-2,11309	0,0001	0,00209829	yes
XLOC_011067	g5049	108,008	100,415	-0,105155	-0,165836	0,76415	0,889199	no
XLOC_011068	g5050	55,1974	55,7156	0,013482	0,0209103	0,96985	0,986679	no
XLOC_011069	g5052	94,8859	70,8004	-0,422436	-0,727434	0,2037	0,456697	no
XLOC_011070	g5054	20,0513	49,4493	1,30226	1,94969	0,0008	0,0106019	yes
XLOC_011071	g5055	127,089	204,245	0,684461	1,10753	0,0512	0,199517	no
XLOC_011072	g5057	24,3767	15,3919	-0,663334	-1,0733	0,0565	0,212905	no
XLOC_011073	g5059	67,9824	98,5241	0,535315	0,886751	0,1241	0,347197	no
XLOC_011074	g5061	88,7908	102,909	0,21289	0,310109	0,576	0,780604	no
XLOC_011075	g11216	47,3544	63,3558	0,419979	0,597449	0,304	0,565487	no
XLOC_011076	g11217	0	0	0	0	1	1	no
XLOC_011077	g11218	5,82871	9,58401	0,717454	0,833738	0,164	0,406174	no
XLOC_011078	g894	28,0929	30,8285	0,134055	0,199366	0,71515	0,865069	no
XLOC_011079	g897	45,8327	89,464	0,964931	1,57869	0,00525	0,0423624	yes
XLOC_011080	g899	143,899	121,534	-0,243704	-0,368686	0,51705	0,741989	no
XLOC_011081	g900	245,497	192,294	-0,352393	-0,593044	0,30035	0,562226	no
XLOC_011082	g901	17,0697	18,7882	0,13839	0,1776	0,7541	0,88421	no
XLOC_011083	g904	0,84	0,332273	-1,33802	0	1	1	no
XLOC_011084	g908	69,7455	97,1727	0,478451	0,799428	0,16065	0,401932	no
XLOC_011085	g911	56,9333	44,3355	-0,360812	-0,616727	0,2781	0,539702	no
XLOC_011086	g895	19,6308	19,0149	-0,0459863	-0,0610551	0,9183	0,964054	no
XLOC_011087	g896	52,648	39,3982	-0,418248	-0,667665	0,25805	0,518211	no
XLOC_011088	g898	25,3396	20,5388	-0,303042	-0,44511	0,42235	0,672637	no
XLOC_011089	g902	409,792	496,435	0,276714	0,399665	0,45705	0,701012	no
XLOC_011090	g903	65,5711	61,9096	-0,0828973	-0,133455	0,81015	0,912557	no
XLOC_011091	g905	15,6899	6,69139	-1,22946	-1,48781	0,05675	0,213437	no
XLOC_011092	g906	7,79966	11,4013	0,547709	0,811155	0,1479	0,384532	no
XLOC_011093	g907	504,887	487,741	-0,0498437	-0,0801494	0,8856	0,948788	no
XLOC_011094	g909	94,8913	112,506	0,245653	0,427527	0,4614	0,702668	no
XLOC_011095	g910	107,729	178,472	0,728294	1,194	0,0351	0,156745	no
XLOC_011096	g113	11,7283	64,8579	2,46729	3,2202	5,00E-05	0,00120049	yes
XLOC_011097	g5062	7,74723	7,45093	-0,0562606	-0,0530801	0,92435	0,966785	no
XLOC_011098	g5063	0	0	0	0	1	1	no
XLOC_011099	g5064	0	0	0	0	1	1	no
XLOC_011100	g5065	0	0	0	0	1	1	no
XLOC_011101	g5066	0,0770648	0,0698162	-0,14251	0	1	1	no
XLOC_011102	g5067	2,06942	1,3428	-0,623984	-0,558458	0,3144	0,575151	no
XLOC_011103	g11219	249,694	240,733	-0,052728	-0,0900463	0,87255	0,942536	no
XLOC_011104	g5068	0	0	0	0	1	1	no
XLOC_011105	g11220	85,0007	159,539	0,90836	1,4964	0,0096	0,0644815	no
XLOC_011106	g11221	0	0	0	0	1	1	no
XLOC_011107	g11222	28,6169	19,5225	-0,551734	-0,789313	0,1628	0,404836	no
XLOC_011108	g5070	0,539902	0,784292	0,538692	0	1	1	no
XLOC_011109	g5069	0	0	0	0	1	1	no
XLOC_011110	g5071	10,8718	5,40669	-1,00777	-1,27072	0,028	0,135891	no
XLOC_011111	g11223	25,5748	14,5055	-0,818123	-1,29601	0,0214	0,11446	no
XLOC_011112	g11224	80,7084	104,301	0,369969	0,498944	0,38855	0,643542	no
XLOC_011113	g912	0	0	0	0	1	1	no
XLOC_011114	g11225	0	0	0	0	1	1	no
XLOC_011115	g5072	3,05918	5,42951	0,827677	0,833225	0,1396	0,372621	no
XLOC_011116	g11226	12,1025	39,8391	1,71888	1,98684	0,00115	0,0138564	yes
XLOC_011117	g5073	1,59877	0,75806	-1,07658	-0,749835	0,2447	0,503131	no
XLOC_011118	g5074	0,54689	2,53116	2,21047	0	1	1	no
XLOC_011119	g913	37,6582	29,7758	-0,338826	-0,539266	0,347	0,607296	no
XLOC_011120	g915	65,1552	42,1603	-0,627996	-1,01395	0,0828	0,271213	no
XLOC_011121	g917	73,7478	100,378	0,444777	0,745034	0,20065	0,452545	no
XLOC_011122	g914	188,099	48,4058	-1,95824	-2,88401	5,00E-05	0,00120049	yes
XLOC_011123	g916	70,3618	73,3606	0,0602133	0,104001	0,85675	0,935212	no
XLOC_011124	g5075	20,1545	30,588	0,601867	0,393921	0,4786	0,714229	no
XLOC_011125	g5076	26,7704	19,1348	-0,484438	-0,782739	0,1754	0,421441	no
XLOC_011126	g11227	434,022	375,466	-0,209085	-0,292758	0,60095	0,797261	no
XLOC_011127	g5077	80,7452	111,831	0,469868	0,74003	0,2015	0,453629	no
XLOC_011128	g5078	15,1951	13,6769	-0,15186	-0,179187	0,7582	0,885423	no
XLOC_011129	g5079	6,86884	5,76325	-0,253185	-0,279644	0,63065	0,815832	no
XLOC_011130	g11228	4,76797	2,79804	-0,76896	-0,604386	0,28455	0,546579	no
XLOC_011131	g5080	344,88	248,478	-0,472977	-0,783968	0,1673	0,410619	no

XLOC_011132	g5082	26,8718	30,8909	0,201092	0,326241	0,57645	0,780604	no
XLOC_011133	g5081	42,9364	68,1886	0,667329	1,04791	0,07045	0,24377	no
XLOC_011134	g5083	133,537	121,802	-0,132704	-0,229356	0,6943	0,854574	no
XLOC_011135	g5085	5,98396	10,2455	0,775813	1,14222	0,05255	0,203246	no
XLOC_011136	g5084	49,3437	62,6976	0,345544	0,589456	0,28945	0,552637	no
XLOC_011137	g918	546,874	454,585	-0,266658	-0,419739	0,4583	0,701013	no
XLOC_011138	g919	7,42766	3,89083	-0,932831	-0,966771	0,1022	0,307854	no
XLOC_011139	g920	114,487	45,35	-1,33601	-2,15794	5,00E-05	0,00120049	yes
XLOC_011140	g923	49,2156	11,8494	-2,05431	-3,00961	5,00E-05	0,00120049	yes
XLOC_011141	g921	34,6541	13,4561	-1,36476	-1,91015	0,0014	0,016042	yes
XLOC_011142	g922	304,468	118,133	-1,36588	-2,27784	5,00E-05	0,00120049	yes
XLOC_011143	g924	0,200493	0,825199	2,04119	0	1	1	no
XLOC_011144	g11229	0	0	0	0	1	1	no
XLOC_011145	g11230	0	0	0	0	1	1	no
XLOC_011146	g11231	0	0,402425	inf	0	1	1	no
XLOC_011147	g925	0	0	0	0	1	1	no
XLOC_011148	g5086	183,615	156,533	-0,230216	-0,37386	0,51695	0,741989	no
XLOC_011149	g927	27,1279	27,5414	0,0218209	0,0322108	0,9539	0,979536	no
XLOC_011150	g926	18,9149	12,7354	-0,570681	-0,861792	0,13455	0,364693	no
XLOC_011151	g11232	0	0	0	0	1	1	no
XLOC_011152	g5087	37,5536	39,3542	0,0675631	0,113987	0,8405	0,926758	no
XLOC_011153	g5088	41,5349	53,5688	0,367068	0,57256	0,3166	0,577009	no
XLOC_011154	g11233	0	0	0	0	1	1	no
XLOC_011155	g11234	32,7616	34,1815	0,0612105	0,0842704	0,87955	0,945715	no
XLOC_011156	g928	123,876	150,814	0,283871	0,486516	0,38845	0,643542	no
XLOC_011157	g930	13,5347	5,01382	-1,43268	-2,05579	0,00085	0,011085	yes
XLOC_011158	g929	8,38218	7,63936	-0,133873	-0,135014	0,80985	0,912533	no
XLOC_011159	g931	321,242	514,128	0,678465	0,963687	0,0931	0,290512	no
XLOC_011160	g115	88,3355	142,094	0,685775	0,894295	0,11085	0,324842	no
XLOC_011161	g114	12,6309	12,1742	-0,0531387	-0,0802542	0,88875	0,950504	no
XLOC_011162	g5089	142,844	75,7895	-0,914368	-1,26662	0,02355	0,122166	no
XLOC_011163	g5091	45,1588	38,4549	-0,231841	-0,401282	0,48385	0,717735	no
XLOC_011164	g5092	189,408	168,246	-0,170924	-0,288335	0,61825	0,808291	no
XLOC_011165	g5093	185,585	271,087	0,546678	0,901319	0,1217	0,344308	no
XLOC_011166	g5090	94,6636	66,4556	-0,510419	-0,752796	0,1965	0,447606	no
XLOC_011167	g5094	34,5783	27,3692	-0,337316	-0,474821	0,39675	0,650975	no
XLOC_011168	g5096	20,1923	17,0339	-0,245395	-0,365897	0,5026	0,730938	no
XLOC_011169	g5095	45,2464	14,4896	-1,64279	-2,29734	0,00015	0,00294012	yes
XLOC_011170	g11235	0	0	0	0	1	1	no
XLOC_011171	g11236	107,837	92,2809	-0,224752	-0,382985	0,49275	0,724047	no
XLOC_011172	g5098	1184,28	2011,49	0,764255	1,01951	0,07295	0,249343	no
XLOC_011173	g5097	61,0381	67,2439	0,139694	0,230082	0,6901	0,851568	no
XLOC_011174	g5099	12,5551	38,8582	1,62995	1,99917	0,00305	0,028799	yes
XLOC_011175	g932	153,105	131,741	-0,216817	-0,35015	0,52715	0,748931	no
XLOC_011176	g5102	46,1768	49,0298	0,0864899	0,150418	0,7964	0,906216	no
XLOC_011177	g5103	14,5788	14,1225	-0,045874	-0,0603631	0,9161	0,963749	no
XLOC_011178	g5104	30,0968	30,9718	0,0413417	0,0716719	0,90305	0,957458	no
XLOC_011179	g5105	86,4318	76,8745	-0,169057	-0,277746	0,62545	0,812208	no
XLOC_011180	g5106	46,0957	37,9811	-0,279348	-0,44522	0,42825	0,677386	no
XLOC_011181	g5108	29,1764	26,6344	-0,131514	-0,203668	0,72525	0,869807	no
XLOC_011182	g5100	5,99704	2,39882	-1,32193	-1,39981	0,0217	0,115749	no
XLOC_011183	g5101	41,3973	27,6873	-0,580312	-0,863942	0,12325	0,346994	no
XLOC_011184	g5107	113,821	160,082	0,492044	0,692773	0,2276	0,485982	no
XLOC_011185	g5109	25,1825	21,9183	-0,200281	-0,314216	0,57775	0,781152	no
XLOC_011186	g5110	31,4635	24,7606	-0,345637	-0,49714	0,3989	0,652731	no
XLOC_011187	g5111	37,2886	55,8395	0,582553	0,934714	0,0847	0,274597	no
XLOC_011188	g5112	69,3303	175,112	1,33672	1,5844	0,0065	0,0492896	yes
XLOC_011189	g5114	6,75919	10,7043	0,663269	0,732111	0,2231	0,480342	no
XLOC_011190	g5113	52,3362	121,99	1,22088	1,81028	0,0019	0,0201056	yes
XLOC_011191	g11237	56,1213	56,0372	-0,00216503	-0,00318222	0,9972	0,998008	no
XLOC_011192	g5115	308,41	391,511	0,344203	0,519068	0,3592	0,619938	no
XLOC_011193	g5116	34,8736	32,9674	-0,0810954	-0,127688	0,8269	0,921071	no
XLOC_011194	g5118	397,328	848,695	1,09491	1,62873	0,00455	0,0383857	yes
XLOC_011195	g5119	62,9077	28,3316	-1,15083	-1,88224	0,00135	0,0156148	yes
XLOC_011196	g5120	115,176	138,364	0,264638	0,439993	0,43065	0,678592	no
XLOC_011197	g5117	394,281	420,383	0,0924796	0,143347	0,8012	0,908537	no
XLOC_011198	g5121	26,087	34,2994	0,394851	0,580588	0,29685	0,558763	no
XLOC_011199	g933	191,891	189,929	-0,0148264	-0,0238287	0,96845	0,986226	no
XLOC_011200	g935	48,3668	42,2067	-0,196542	-0,283629	0,628	0,813648	no
XLOC_011201	g938	151,137	204,595	0,436914	0,747434	0,1849	0,433346	no
XLOC_011202	g940	33,4605	22,2776	-0,586864	-0,909655	0,1127	0,328304	no
XLOC_011203	g943	35,4209	27,7717	-0,350986	-0,59915	0,29385	0,556101	no
XLOC_011204	g934	0,21425	1,35205	2,65778	1,2717	0,24695	0,505742	no
XLOC_011205	g936	61,9297	90,5838	0,548622	0,932679	0,09535	0,295281	no
XLOC_011206	g937	34,4217	98,4121	1,51552	1,90343	0,00115	0,0138564	yes
XLOC_011207	g939	1,62733	2,28289	0,488349	0,628246	0,26875	0,529838	no
XLOC_011208	g941	1,95531	1,16473	-0,747402	-0,848386	0,14775	0,384374	no
XLOC_011209	g942	26,6665	39,675	0,573202	0,840447	0,14745	0,384102	no
XLOC_011210	g944	62,7242	60,311	-0,0566012	-0,0837649	0,88645	0,949285	no
XLOC_011211	g945	4,42136	3,15943	-0,484826	-0,634369	0,2639	0,524581	no
XLOC_011212	g946	61,3151	48,3116	-0,343874	-0,552256	0,321	0,581038	no
XLOC_011213	g947	84,7543	108,321	0,353955	0,611294	0,27805	0,539702	no
XLOC_011214	g11238	35,7411	32,8953	-0,119703	-0,153746	0,7963	0,906216	no
XLOC_011215	g11239	2,85485	2,17629	-0,391543	-0,550965	0,3374	0,597377	no
XLOC_011216	g11240	2,24395	1,789	-0,326888	-0,423626	0,4662	0,706494	no
XLOC_011217	g11241	8,4578	6,10715	-0,469783	-0,750797	0,1901	0,440071	no
XLOC_011218	g11242	145,593	159,324	0,130025	0,222425	0,69485	0,854745	no
XLOC_011219	g948	0	0,0492801	inf	0	1	1	no
XLOC_011220	g11243	5,99863	7,45624	0,313816	0,389179	0,49945	0,728948	no
XLOC_011221	g949	0,0224814	0	#NAME?	0	1	1	no
XLOC_011222	g5122	3,89187	2,05267	-0,922965	-0,836898	0,2438	0,502649	no
XLOC_011223	g11244	0	0	0	0	1	1	no

XLOC_011224	g11245	159,395	121,072	-0,396737	-0,686961	0,2361	0,494034	no
XLOC_011225	g11246	22,0347	32,2612	0,550025	0,660087	0,26565	0,526567	no
XLOC_011226	g11247	89,2758	236,874	1,40778	2,1816	0,00035	0,00552572	yes
XLOC_011227	g11248	0	0	0	0	1	1	no
XLOC_011228	g5123	0	0	0	0	1	1	no
XLOC_011229	g5124	58,0353	65,4816	0,17416	0,287531	0,6112	0,804448	no
XLOC_011230	g5125	25,0882	15,1804	-0,724798	-1,14906	0,04315	0,178414	no
XLOC_011231	g5126	52,4673	40,1551	-0,385834	-0,589246	0,28995	0,552768	no
XLOC_011232	g5127	4,39791	6,45433	0,553449	0,76971	0,18565	0,433654	no
XLOC_011233	g11249	0	0,949072	inf	0	1	1	no
XLOC_011234	g5128	0	0	0	0	1	1	no
XLOC_011235	g11250	0	0	0	0	1	1	no
XLOC_011236	g5130	38,6019	53,3889	0,467867	0,78906	0,16825	0,411101	no
XLOC_011237	g5129	36,8323	49,7328	0,433226	0,734446	0,19415	0,445456	no
XLOC_011238	g5131	6,76057	5,27746	-0,357302	-0,470696	0,40785	0,660523	no
XLOC_011239	g5132	77,9454	53,9292	-0,531398	-0,867607	0,13615	0,367609	no
XLOC_011240	g11251	259,072	176,117	-0,556823	-0,946696	0,0993	0,302177	no
XLOC_011241	g11252	208,149	244,285	0,230947	0,365078	0,53185	0,752343	no
XLOC_011242	g11253	8,58983	0,860292	-3,31973	-2,19499	0,1155	0,333246	no
XLOC_011243	g5133	0	0	0	0	1	1	no
XLOC_011244	g5134	11,7091	11,2291	-0,060384	-0,080546	0,88765	0,950055	no
XLOC_011245	g5135	52,3595	42,2709	-0,308786	-0,483984	0,3849	0,640945	no
XLOC_011246	g5136	24,0387	17,6112	-0,448863	-0,595636	0,3294	0,590473	no
XLOC_011247	g5137	6,30194	9,70031	0,622235	0,766708	0,18675	0,434879	no
XLOC_011248	g11254	0	0	0	0	1	1	no
XLOC_011249	g5138	2,26101	9,52542	2,07481	1,86909	0,00535	0,0426783	yes
XLOC_011250	g950	0	0	0	0	1	1	no
XLOC_011251	g11255	152,431	124,433	-0,292784	-0,447922	0,4288	0,677386	no
XLOC_011252	g11257	28,5911	43,3406	0,600154	1,00212	0,07175	0,246963	no
XLOC_011253	g11258	2,97359	12,4343	2,06404	2,42977	5,00E-05	0,00120049	yes
XLOC_011254	g11256	44,3212	51,5985	0,219334	0,362399	0,524	0,746323	no
XLOC_011255	g11259	0	0	0	0	1	1	no
XLOC_011256	g5139	10,9978	8,76574	-0,327271	-0,520426	0,35075	0,611245	no
XLOC_011257	g5140	81,2563	57,6683	-0,494701	-0,824411	0,13905	0,372164	no
XLOC_011258	g11260	160,246	161,443	0,0107328	0,0184859	0,97445	0,987624	no
XLOC_011259	g11261	58,5598	55,4289	-0,0792721	-0,136288	0,8154	0,916054	no
XLOC_011260	g11262	74,3672	64,3669	-0,208346	-0,334806	0,57165	0,77837	no
XLOC_011261	g951	17,5615	46,5678	1,40691	1,42764	0,01475	0,0876786	no
XLOC_011262	g952	1,57853	3,42105	1,11586	1,15463	0,0469	0,187983	no
XLOC_011263	g953	62,6724	88,6245	0,499876	0,857996	0,13415	0,36391	no
XLOC_011264	g955	16,4318	20,2724	0,303028	0,463512	0,40955	0,662373	no
XLOC_011265	g954	91,4748	85,9072	-0,090594	-0,150406	0,79075	0,903527	no
XLOC_011266	g5141	30,4082	16,7959	-0,856349	-1,28368	0,0272	0,133552	no
XLOC_011267	g5143	182,778	182,08	-0,00551794	-0,00883562	0,98585	0,992622	no
XLOC_011268	g5142	18,8245	10,3719	-0,859931	-1,29495	0,02595	0,129618	no
XLOC_011269	g956	97,2	76,2031	-0,351107	-0,523268	0,35475	0,615463	no
XLOC_011270	g957	52,478	50,0272	-0,0689994	-0,101172	0,8582	0,935373	no
XLOC_011271	g11263	29,1991	26,2432	-0,153982	-0,189884	0,7521	0,88326	no
XLOC_011272	g5144	4,20082	3,96334	-0,0839546	-0,103838	0,86065	0,935634	no
XLOC_011273	g11264	79,4793	48,1781	-0,722202	-1,18945	0,0401	0,170395	no
XLOC_011274	g11265	9,65731	16,1853	0,744991	1,07453	0,0681	0,238841	no
XLOC_011275	g11267	0	0	0	0	1	1	no
XLOC_011276	g11266	0	0	0	0	1	1	no
XLOC_011277	g11268	39,1288	34,359	-0,187541	-0,314685	0,56645	0,775267	no
XLOC_011278	g5145	76,3359	51,9267	-0,555885	-0,889268	0,10915	0,321685	no
XLOC_011279	g5146	62,7	268,881	2,10043	2,7221	0,0001	0,00209829	yes
XLOC_011280	g5147	12	13,7896	0,200547	0,286286	0,6046	0,79937	no
XLOC_011281	g11269	1157,72	1490,92	0,364919	0,594743	0,2946	0,556907	no
XLOC_011282	g11270	0	0	0	0	1	1	no
XLOC_011283	g11271	27,1734	157,048	2,53094	3,75883	5,00E-05	0,00120049	yes
XLOC_011284	g11275	41,473	55,6959	0,425399	0,71172	0,2137	0,469366	no
XLOC_011285	g11272	13,5159	55,4407	2,03629	2,47771	0,00025	0,00430702	yes
XLOC_011286	g11273	35,2472	30,1196	-0,226806	-0,376665	0,51055	0,736753	no
XLOC_011287	g11274	72,3134	62,9238	-0,200656	-0,338148	0,5442	0,761477	no
XLOC_011288	g5149	73,7289	95,7296	0,376734	0,650444	0,261	0,521008	no
XLOC_011289	g5148	115,359	62,4314	-0,885781	-1,43639	0,01445	0,0864711	no
XLOC_011290	g5150	0	0	0	0	1	1	no
XLOC_011291	g11276	114,665	63,3077	-0,856966	-1,35651	0,01995	0,108718	no
XLOC_011292	g11278	565,109	1541,88	1,44809	2,29522	0,0002	0,00367103	yes
XLOC_011293	g11277	6105,08	4982,77	-0,293063	-0,348845	0,54525	0,761892	no
XLOC_011294	g11279	295,458	258,96	-0,190219	-0,283259	0,61185	0,804764	no
XLOC_011295	g5151	276,301	251,226	-0,137251	-0,21855	0,6959	0,855501	no
XLOC_011296	g5152	25,6754	34,6253	0,431441	0,668319	0,2327	0,490578	no
XLOC_011297	g11280	1,8737	0,628221	-1,57655	-1,32024	0,0359	0,158373	no
XLOC_011298	g11281	84,55	43,9694	-0,943304	-1,47021	0,01365	0,0831532	no
XLOC_011299	g5153	35,8098	10,3448	-1,79146	-2,68041	5,00E-05	0,00120049	yes
XLOC_011300	g958	10,3618	21,8877	1,07885	0,846767	0,15515	0,392851	no
XLOC_011301	g959	93,6165	126,818	0,43793	0,651489	0,2357	0,493721	no
XLOC_011302	g961	140,264	116,757	-0,264629	-0,456797	0,42205	0,672377	no
XLOC_011303	g960	7,66707	8,76329	0,192798	0,254001	0,6576	0,832081	no
XLOC_011304	g962	132,839	181,839	0,452989	0,620842	0,2682	0,529179	no
XLOC_011305	g11282	219,09	307,552	0,489309	0,767489	0,18735	0,435863	no
XLOC_011306	g5154	304,523	299,045	-0,0261885	-0,0417944	0,9411	0,973928	no
XLOC_011307	g11283	144,407	204,735	0,503617	0,793768	0,16055	0,401887	no
XLOC_011308	g11284	142,781	42,5778	-1,74563	-2,6468	0,0001	0,00209829	yes
XLOC_011309	g11285	616,365	342,046	-0,849595	-0,989559	0,09305	0,290512	no
XLOC_011310	g5156	27,5916	18,9106	-0,545034	-0,876624	0,1265	0,351012	no
XLOC_011311	g5157	8,94317	11,3187	0,339849	0,380554	0,5063	0,733855	no
XLOC_011312	g5155	43,8935	38,864	-0,175571	-0,206479	0,7272	0,870385	no
XLOC_011313	g5158	40,8455	32,5707	-0,326604	-0,530042	0,3548	0,615463	no
XLOC_011314	g5160	73,1927	50,2136	-0,543622	-0,841498	0,14455	0,380558	no
XLOC_011315	g5159	91,6687	102,951	0,167451	0,287211	0,6157	0,807125	no

XLOC_011316	g5161	5,36712	7,37999		0,459471	0,60467	0,29475	0,556907	no
XLOC_011317	g11286	2,72942	4,35751		0,674911	0,667568	0,2322	0,49005	no
XLOC_011318	g11287	0	0		0	0	1	1	no
XLOC_011319	g5162	8,84668	4,36963		-1,01762	-1,40267	0,01515	0,0890856	no
XLOC_011320	g5163	165,273	325,551		0,978033	1,63848	0,0049	0,0404013	yes
XLOC_011321	g5164	19,3859	13,1253		-0,562659	-0,869112	0,1229	0,346406	no
XLOC_011322	g5165	3,74545	5,24695		0,486339	0,511161	0,3617	0,621784	no
XLOC_011323	g5166	74,668	82,2962		0,140337	0,221308	0,6921	0,853072	no
XLOC_011324	g11288	0	1,82672	inf		0	1	1	no
XLOC_011325	g5167	131,836	132,249		0,0045064	0,00679139	0,9906	0,995057	no
XLOC_011326	g5168	227,954	230,211		0,0142114	0,0245591	0,9636	0,983889	no
XLOC_011327	g5170	16,8797	33,0227		0,968166	1,58346	0,0084	0,0588359	no
XLOC_011328	g5171	8,7363	5,17742		-0,754789	-0,960058	0,0802	0,265262	no
XLOC_011329	g5169	50,8981	60,4322		0,247706	0,349585	0,5475	0,76377	no
XLOC_011330	g5172	0	0		0	0	1	1	no
XLOC_011331	g5173	168,544	222,155		0,398442	0,650331	0,264	0,524581	no
XLOC_011332	g5174	4,48502	4,73822		0,0792331	0,105839	0,8471	0,930483	no
XLOC_011333	g5176	6,18111	2,00095		-1,62718	-2,10237	0,0003	0,00496796	yes
XLOC_011334	g5175	85,1978	114,774		0,42991	0,66449	0,2373	0,495382	no
XLOC_011335	g5177	82,1838	102,472		0,318304	0,515209	0,36795	0,627849	no
XLOC_011336	g5178	26,6687	35,5156		0,413303	0,673615	0,24395	0,502852	no
XLOC_011337	g5179	201,694	120,463		-0,743577	-1,20004	0,0405	0,171279	no
XLOC_011338	g11289	129,721	118,161		-0,134661	-0,196304	0,7255	0,869807	no
XLOC_011339	g11292	36,0136	38,9944		0,114724	0,195477	0,73245	0,873744	no
XLOC_011340	g11293	27,2752	51,7943		0,925204	1,49598	0,0087	0,0603347	no
XLOC_011341	g11295	74,1163	45,4559		-0,705322	-1,15129	0,0417	0,174624	no
XLOC_011342	g11297	3,6234	4,361		0,267314	0,257083	0,65735	0,832081	no
XLOC_011343	g11299	15,3855	11,0624		-0,475904	-0,733902	0,2086	0,463556	no
XLOC_011344	g11290	40,4534	41,3207		0,030603	0,0518693	0,92645	0,967433	no
XLOC_011345	g11291	1312,54	2181,57		0,733005	1,13251	0,04535	0,184252	no
XLOC_011346	g11294	47,6789	68,4287		0,52125	0,903334	0,1154	0,333204	no
XLOC_011347	g11296	91,588	82,9458		-0,142989	-0,231124	0,68105	0,846464	no
XLOC_011348	g11298	26,6375	23,7992		-0,162543	-0,246829	0,66095	0,833937	no
XLOC_011349	g11300	8,63552	3,87232		-1,15708	-1,59102	0,00505	0,0412571	yes
XLOC_011350	g963	106,125	82,9672		-0,355151	-0,613333	0,28655	0,549334	no
XLOC_011351	g964	141,677	133,23		-0,0886846	-0,130764	0,818	0,917235	no
XLOC_011352	g5180	63,008	47,7246		-0,400804	-0,654577	0,22965	0,487604	no
XLOC_011353	g5182	18,8848	45,2082		1,25936	1,99494	0,0006	0,00845337	yes
XLOC_011354	g5183	17,1984	23,2351		0,434037	0,674229	0,2281	0,486204	no
XLOC_011355	g5186	24,8598	28,5734		0,200858	0,323984	0,5624	0,772769	no
XLOC_011356	g5181	12,0628	15,1985		0,333363	0,564318	0,30775	0,568599	no
XLOC_011357	g5184	332,238	457,382		0,461183	0,523115	0,36765	0,627446	no
XLOC_011358	g5185	0,649476	1,11743		0,782836	0,713241	0,22855	0,486741	no
XLOC_011359	g5187	76,4725	51,9728		-0,557184	-0,925204	0,09555	0,295435	no
XLOC_011360	g5188	114,209	107,409		-0,0885611	-0,150927	0,78795	0,902094	no
XLOC_011361	g5190	11,4538	46,9021		2,03382	1,39248	0,0333	0,151392	no
XLOC_011362	g5191	1,1631	0	#NAME?		0	1	1	no
XLOC_011363	g5193	39,5385	48,629		0,298559	0,514642	0,3693	0,628841	no
XLOC_011364	g5189	85,017	99,563		0,227859	0,362037	0,52175	0,74532	no
XLOC_011365	g5192	87,3046	89,3581		0,0335403	0,0578189	0,9189	0,964163	no
XLOC_011366	g5194	104,098	195,152		0,90666	1,52458	0,0086	0,0599801	no
XLOC_011367	g11301	95,8363	149,746		0,643868	0,613873	0,29205	0,554618	no
XLOC_011368	g11302	0	0,757956	inf		0	1	1	no
XLOC_011369	g5196	42,7039	87,0635		1,0277	1,67429	0,00535	0,0426783	yes
XLOC_011370	g5195	42,0948	54,613		0,375602	0,611406	0,2729	0,53424	no
XLOC_011371	g11303	0	0		0	0	1	1	no
XLOC_011372	g5197	16,4945	21,0768		0,353667	0,542407	0,32265	0,583181	no
XLOC_011373	g11304	247,631	812,892		1,71487	2,68118	5,00E-05	0,00120049	yes
XLOC_011374	g11305	28,7211	43,3684		0,594532	0,900559	0,1234	0,347009	no
XLOC_011375	g11306	46,2494	65,8911		0,510649	0,82442	0,1363	0,367811	no
XLOC_011376	g11307	0	0		0	0	1	1	no
XLOC_011377	g5198	0	0		0	0	1	1	no
XLOC_011378	g11309	39,9977	34,2906		-0,222105	-0,346755	0,5496	0,765215	no
XLOC_011379	g11308	277,718	148,493		-0,903225	-1,32376	0,023	0,120011	no
XLOC_011380	g5199	610,944	819,918		0,42444	0,570292	0,2961	0,5581	no
XLOC_011381	g5200	119,655	94,1662		-0,345605	-0,59277	0,30935	0,570452	no
XLOC_011382	g5202	135,028	164,03		0,280699	0,401879	0,4659	0,706334	no
XLOC_011383	g5204	267,984	199,266		-0,427447	-0,667375	0,23875	0,496827	no
XLOC_011384	g5201	186,066	145,013		-0,359637	-0,575835	0,3032	0,564534	no
XLOC_011385	g5203	24,3355	41,093		0,755832	1,21138	0,03715	0,162067	no
XLOC_011386	g5205	60,1081	96,3159		0,680216	1,10543	0,055	0,208964	no
XLOC_011387	g11310	0	0		0	0	1	1	no
XLOC_011388	g5206	45,4296	12,042		-1,91556	-2,42979	0,0001	0,00209829	yes
XLOC_011389	g5207	1,65054	2,47638		0,585292	0,576606	0,32045	0,580544	no
XLOC_011390	g11311	3,21884	2,85209		-0,174522	-0,185085	0,7489	0,881846	no
XLOC_011391	g5208	15,8762	19,9413		0,328897	0,41882	0,45885	0,701036	no
XLOC_011392	g5209	26,0783	18,9029		-0,464242	-0,707232	0,2237	0,481107	no
XLOC_011393	g5210	57,6306	40,5646		-0,506615	-0,822226	0,15525	0,392851	no
XLOC_011394	g5211	0	0		0	0	1	1	no
XLOC_011395	g117	617,093	1434,98		1,21747	1,54786	0,0059	0,0459825	yes
XLOC_011396	g119	13,2451	9,78856		-0,436295	-0,680989	0,2204	0,477355	no
XLOC_011397	g122	14,1131	7,84405		-0,847361	-1,34768	0,01775	0,0996029	no
XLOC_011398	g125	200,326	140,695		-0,509781	-0,857608	0,12865	0,354275	no
XLOC_011399	g116	50,6158	61,6375		0,284222	0,434092	0,46425	0,705281	no
XLOC_011400	g118	231,545	248,013		0,0991241	0,167208	0,772	0,89378	no
XLOC_011401	g120	35,9882	33,7356		-0,0932536	-0,153362	0,78065	0,898182	no
XLOC_011402	g121	4874,53	3434,42		-0,505194	-0,708108	0,21225	0,46775	no
XLOC_011403	g123	301,485	126,744		-1,25016	-1,72075	0,00465	0,0389616	yes
XLOC_011404	g124	37,5306	37,2401		-0,0112129	-0,0190199	0,97455	0,987624	no
XLOC_011405	g126	7,74261	8,02454		0,0515984	0,0714357	0,9044	0,958369	no
XLOC_011406	g15	12,7403	18,8755		0,56712	0,756333	0,1883	0,437348	no
XLOC_011407	g11312	0	0		0	0	1	1	no

XLOC_011408	g11313	1083,16	3092,21	1,51339	2,32644	5,00E-05	0,00120049	yes
XLOC_011409	g965	18,1844	16,7814	-0,115842	-0,175079	0,7514	0,883013	no
XLOC_011410	g966	0	0,0169023	inf	0	1	1	no
XLOC_011411	g967	0	0	0	0	1	1	no
XLOC_011412	g11314	277,563	375,727	0,436867	0,73904	0,1964	0,447606	no
XLOC_011413	g11315	0	0	0	0	1	1	no
XLOC_011414	g5212	78,764	67,9783	-0,212462	-0,326083	0,5475	0,76377	no
XLOC_011415	g11316	0	0	0	0	1	1	no
XLOC_011416	g5213	0	0	0	0	1	1	no
XLOC_011417	g5214	0	0	0	0	1	1	no
XLOC_011418	g968	1,88864	0,777476	-1,28048	-1,00077	0,15895	0,39933	no
XLOC_011419	g5215	27,2436	39,0851	0,520698	0,810547	0,1565	0,394565	no
XLOC_011420	g11317	2098,92	1760,72	-0,25348	-0,388504	0,49145	0,723327	no
XLOC_011421	g11318	181,643	76,6899	-1,244	-2,05759	0,00045	0,00669545	yes
XLOC_011422	g5216	123,262	36,2059	-1,76743	-2,51204	5,00E-05	0,00120049	yes
XLOC_011423	g969	33,2041	29,8289	-0,154647	-0,243369	0,67645	0,843308	no
XLOC_011424	g970	31,4226	21,9059	-0,520482	-0,85065	0,12875	0,354351	no
XLOC_011425	g5217	99,5623	68,9884	-0,529247	-0,865815	0,12535	0,348905	no
XLOC_011426	g11319	715,118	611,973	-0,224713	-0,360738	0,506	0,733528	no
XLOC_011427	g11320	0	0	0	0	1	1	no
XLOC_011428	g5218	0	0	0	0	1	1	no
XLOC_011429	g5219	0	0	0	0	1	1	no
XLOC_011430	g5220	0	0	0	0	1	1	no
XLOC_011431	g11321	32,9593	31,1129	-0,0831723	-0,1405	0,80215	0,908624	no
XLOC_011432	g11322	292,081	248,399	-0,233708	-0,370694	0,51305	0,739382	no
XLOC_011433	g5221	12,1138	8,43992	-0,521352	-0,765287	0,17025	0,41403	no
XLOC_011434	g11323	16,1097	13,7037	-0,233368	-0,294799	0,59595	0,793092	no
XLOC_011435	g11324	696,285	263,211	-1,40346	-2,15996	0,0003	0,00496796	yes
XLOC_011436	g5222	4,76325	6,95711	0,546541	0,663023	0,26765	0,528625	no
XLOC_011437	g11325	0	0	0	0	1	1	no
XLOC_011438	g5223	0,0374001	0	#NAME?	0	1	1	no
XLOC_011439	g11326	0	0	0	0	1	1	no
XLOC_011440	g5224	0	0	0	0	1	1	no
XLOC_011441	g5225	0	0	0	0	1	1	no
XLOC_011442	g5226	16,9117	18,8657	0,157748	0,258582	0,6536	0,829883	no
XLOC_011443	g5228	38,3857	24,763	-0,632385	-0,744149	0,193	0,443751	no
XLOC_011444	g5227	18,5957	19,6405	0,0788572	0,0994016	0,86175	0,936105	no
XLOC_011445	g11327	0	0	0	0	1	1	no
XLOC_011446	g971	0	0	0	0	1	1	no
XLOC_011447	g5229	76,0114	79,5447	0,0655499	0,0985301	0,8635	0,936821	no
XLOC_011448	g5232	23,5516	45,333	0,944739	1,61297	0,00525	0,0423624	yes
XLOC_011449	g5234	157,588	219,663	0,479138	0,728931	0,1819	0,429595	no
XLOC_011450	g5235	96,4698	72,3179	-0,415725	-0,663817	0,2315	0,489204	no
XLOC_011451	g5230	78,3209	71,9078	-0,123249	-0,197144	0,7331	0,873988	no
XLOC_011452	g5231	384,275	451,294	0,231931	0,367442	0,5157	0,741858	no
XLOC_011453	g5233	202,335	243,089	0,26474	0,418463	0,45495	0,699375	no
XLOC_011454	g5236	41,6328	55,6919	0,419749	0,657769	0,2463	0,505152	no
XLOC_011455	g5237	92,9229	113,287	0,285883	0,460952	0,4156	0,667815	no
XLOC_011456	g11328	11,5186	26,6224	1,20867	3,99691	0,3036	0,565064	no
XLOC_011457	g11329	27,296	21,8365	-0,321945	-0,330614	0,5611	0,772142	no
XLOC_011458	g11330	9,48193	4,92653	-0,94461	-1,11074	0,1459	0,382369	no
XLOC_011459	g11331	9,87308	8,97465	-0,137644	-0,137031	0,8089	0,912195	no
XLOC_011460	g5238	30,2943	13,5416	-1,16165	-1,57277	0,0084	0,0588359	no
XLOC_011461	g972	7,02872	10,3914	0,564057	0,490044	0,3921	0,646804	no
XLOC_011462	g973	16,5818	16,575	-0,000594248	-0,000725503	0,9901	0,99501	no
XLOC_011463	g974	7,75851	7,13076	-0,121724	-0,1784	0,7555	0,884596	no
XLOC_011464	g976	3,25088	32,4213	3,31804	4,12885	5,00E-05	0,00120049	yes
XLOC_011465	g977	23,8324	42,2892	0,827363	1,33455	0,01585	0,0918813	no
XLOC_011466	g978	130,993	157,37	0,26468	0,341867	0,5448	0,761892	no
XLOC_011467	g975	1,61908	3,83276	1,24321	0	1	1	no
XLOC_011468	g979	185,799	153,236	-0,277987	-0,376477	0,4952	0,725692	no
XLOC_011469	g980	87,7923	91,9594	0,0669031	0,103093	0,8561	0,934827	no
XLOC_011470	g981	111,46	114,094	0,0336965	0,0572629	0,9165	0,963809	no
XLOC_011471	g5242	12,6189	8,74197	-0,529556	-0,745835	0,199	0,451802	no
XLOC_011472	g5239	7,58397	3,78538	-1,00251	-1,49944	0,00915	0,0624413	no
XLOC_011473	g5240	14,9514	11,3466	-0,398022	-0,592187	0,28955	0,552637	no
XLOC_011474	g5241	28,0443	17,6776	-0,665788	-1,04526	0,06275	0,226296	no
XLOC_011475	g5243	12,2471	6,52881	-0,907551	-1,12607	0,0612	0,223082	no
XLOC_011476	g5244	0	0	0	0	1	1	no
XLOC_011477	g11332	20,1078	19,705	-0,0291928	-0,0425318	0,9409	0,973926	no
XLOC_011478	g11333	151,57	111,909	-0,437657	-0,685666	0,22675	0,485222	no
XLOC_011479	g5245	28,1355	16,5872	-0,762316	-1,18154	0,0405	0,171279	no
XLOC_011480	g983	15,3545	16,0982	0,0682331	0,0966024	0,8558	0,934827	no
XLOC_011481	g982	39,7396	36,0961	-0,138733	-0,211718	0,70925	0,861985	no
XLOC_011482	g984	167,473	71,7095	-1,22369	-1,82663	0,0015	0,0168343	yes
XLOC_011483	g5248	53,1024	48,1539	-0,141124	-0,240291	0,66435	0,83565	no
XLOC_011484	g5246	122,523	90,519	-0,436762	-0,738088	0,204	0,457265	no
XLOC_011485	g5247	126,676	102,319	-0,30807	-0,420254	0,44985	0,694824	no
XLOC_011486	g5249	60,8079	34,7513	-0,807192	-1,13335	0,05175	0,200943	no
XLOC_011487	g11335	0,293216	0,129913	-1,17442	0	1	1	no
XLOC_011488	g11334	3,90753	9,42802	1,2707	1,58101	0,0061	0,0471669	yes
XLOC_011489	g11336	236,668	275,574	0,219574	0,374482	0,50435	0,732434	no
XLOC_011490	g11337	1,99106	2,02443	0,0239794	0,0230386	0,95955	0,981847	no
XLOC_011491	g11338	0,104742	0	#NAME?	0	1	1	no
XLOC_011492	g11339	34,0885	24,6476	-0,467835	-0,545577	0,3302	0,591007	no
XLOC_011493	g5250	86,5901	66,0678	-0,390255	-0,645614	0,2525	0,511771	no
XLOC_011494	g5252	2468,38	3524,83	0,513989	0,740821	0,1825	0,42989	no
XLOC_011495	g5253	106,638	73,0194	-0,546363	-0,89934	0,12205	0,344901	no
XLOC_011496	g5257	0	0	0	0	1	1	no
XLOC_011497	g5251	85,6856	105,839	0,304746	0,511674	0,3578	0,618591	no
XLOC_011498	g5254	633,004	454,84	-0,476854	-0,780513	0,1802	0,427225	no
XLOC_011499	g5255	65,4748	64,9751	-0,0110533	-0,0166403	0,97345	0,98758	no

XLOC_011500	g5256	89,5567	171,353	0,936099	1,51753	0,0062	0,0477522	yes
XLOC_011501	g5258	0,23511	0	#NAME?	0	1	1	no
XLOC_011502	g11340	261,491	4566,2	4,12616	4,8022	5,00E-05	0,00120049	yes
XLOC_011503	g5259	0	0	0	0	1	1	no
XLOC_011504	g11341	0	0	0	0	1	1	no
XLOC_011505	g11342	144,192	44,6345	-1,69176	-2,44274	5,00E-05	0,00120049	yes
XLOC_011506	g11343	80,7187	77,5575	-0,0576362	-0,098267	0,86385	0,936935	no
XLOC_011507	g11345	47,1122	47,6879	0,0175201	0,0300929	0,9581	0,981385	no
XLOC_011508	g11346	184,497	253,302	0,457263	0,783883	0,17165	0,41661	no
XLOC_011509	g11347	30,12	34,7242	0,20522	0,348766	0,5409	0,759565	no
XLOC_011510	g11349	63,7584	89,0468	0,481949	0,747697	0,19425	0,445582	no
XLOC_011511	g11351	120,013	137,92	0,200635	0,261539	0,6478	0,826477	no
XLOC_011512	g11344	79,3402	66,7448	-0,249397	-0,427136	0,44525	0,6915	no
XLOC_011513	g11348	131,842	77,6147	-0,76441	-1,12209	0,0481	0,191154	no
XLOC_011514	g11350	389,839	320,847	-0,28099	-0,481126	0,39875	0,65273	no
XLOC_011515	g11352	23,889	25,0818	0,070294	0,113911	0,84535	0,929972	no
XLOC_011516	g11353	130,055	123,648	-0,0728833	-0,114972	0,83705	0,925448	no
XLOC_011517	g5264	20,4772	19,3609	-0,0808709	-0,118563	0,84165	0,927714	no
XLOC_011518	g5266	74,867	27,294	-1,45575	-2,41388	0,0001	0,00209829	yes
XLOC_011519	g5267	41,4284	29,1715	-0,506059	-0,851455	0,1414	0,375486	no
XLOC_011520	g5268	105,047	135,244	0,364532	0,6002	0,2975	0,559437	no
XLOC_011521	g5269	112,329	76,2213	-0,559459	-0,93137	0,0934	0,291263	no
XLOC_011522	g5271	147,229	47,3804	-1,6357	-2,62751	5,00E-05	0,00120049	yes
XLOC_011523	g5260	17,1253	13,5941	-0,333156	-0,508356	0,3477	0,608067	no
XLOC_011524	g5261	60,3337	43,3207	-0,477908	-0,775901	0,162	0,403766	no
XLOC_011525	g5262	3,5648	4,90489	0,460402	0,357255	0,5937	0,791923	no
XLOC_011526	g5263	15,9497	20,5072	0,362599	0,520393	0,3556	0,616198	no
XLOC_011527	g5265	43,052	38,478	-0,162048	-0,277263	0,6265	0,812884	no
XLOC_011528	g5270	1559,2	1992,47	0,353751	0,510073	0,3705	0,629539	no
XLOC_011529	g11354	0,66025	1,48693	1,17125	0,815205	0,19135	0,441819	no
XLOC_011530	g5272	31,1473	37,2668	0,258784	0,428433	0,4649	0,705296	no
XLOC_011531	g5273	30,1646	34,181	0,180337	0,309024	0,58065	0,783024	no
XLOC_011532	g5274	151,298	156,027	0,0443997	0,0761953	0,89285	0,951986	no
XLOC_011533	g985	9,71886	5,74359	-0,758834	-1,06847	0,05755	0,214906	no
XLOC_011534	g11355	140,375	76,2943	-0,879642	-1,39076	0,01335	0,0818844	no
XLOC_011535	g11356	7,2583	9,46877	0,383544	0,434831	0,4573	0,701012	no
XLOC_011536	g11357	9,04865	6,30833	-0,520443	-0,567853	0,33035	0,591007	no
XLOC_011537	g986	18,485	28,3804	0,618539	0,978047	0,0744	0,252007	no
XLOC_011538	g11358	33,3387	104,202	1,64411	2,57341	5,00E-05	0,00120049	yes
XLOC_011539	g5275	46,8762	50,9452	0,12009	0,202511	0,72225	0,868752	no
XLOC_011540	g5276	93,2079	146,336	0,650765	1,11542	0,0506	0,19836	no
XLOC_011541	g5278	36,8216	36,721	-0,00394863	-0,00638467	0,99135	0,995293	no
XLOC_011542	g5279	221,115	233,839	0,0807169	0,121085	0,82265	0,919263	no
XLOC_011543	g5280	1221,42	1347,22	0,141434	0,213854	0,7125	0,863903	no
XLOC_011544	g5281	173,309	164,797	-0,0726565	-0,0992639	0,8604	0,935634	no
XLOC_011545	g5283	423,315	806,196	0,929399	1,5815	0,0057	0,0448151	yes
XLOC_011546	g5284	56,7636	57,8701	0,0278532	0,0444981	0,9399	0,973199	no
XLOC_011547	g5277	204,196	176,987	-0,206315	-0,331536	0,57235	0,778999	no
XLOC_011548	g5282	268,005	207,188	-0,371317	-0,542491	0,3348	0,594958	no
XLOC_011549	g11359	0	0	0	0	1	1	no
XLOC_011550	g5285	0	0	0	0	1	1	no
XLOC_011551	g5286	58,5725	43,3264	-0,434976	-0,679497	0,20965	0,464522	no
XLOC_011552	g11360	2,60723	0,642634	-2,02045	-0,844253	0,1565	0,394565	no
XLOC_011553	g5287	0	0	0	0	1	1	no
XLOC_011554	g11361	0	0	0	0	1	1	no
XLOC_011555	g5288	190,61	66,0323	-1,52938	-1,75489	0,00115	0,0138564	yes
XLOC_011556	g11362	0	0,389739	inf	0	1	1	no
XLOC_011557	g11363	69,719	41,8457	-0,736473	-1,2351	0,0314	0,145447	no
XLOC_011558	g11364	41,7853	47,7233	0,191698	0,312869	0,58405	0,784915	no
XLOC_011559	g11365	7,36206	11,189	0,603901	0,714803	0,2081	0,462759	no
XLOC_011560	g11366	1,32183	22,3505	4,0797	3,33966	0,12395	0,347197	no
XLOC_011561	g11367	34,8567	9,90038	-1,81588	-2,34394	0,0004	0,0061761	yes
XLOC_011562	g11368	0	0,195002	inf	0	1	1	no
XLOC_011563	g5289	38,5813	37,033	-0,0590892	-0,0998169	0,86085	0,935644	no
XLOC_011564	g11369	0	0	0	0	1	1	no
XLOC_011565	g5290	0,727841	0,640937	-0,183442	0	1	1	no
XLOC_011566	g11370	0	0	0	0	1	1	no
XLOC_011567	g11371	3,44641	13,0397	1,91974	3,21933	0,3123	0,573414	no
XLOC_011568	g5291	8,09945	8,80605	0,12067	0,141436	0,803	0,908624	no
XLOC_011569	g5293	40,4959	74,9195	0,887565	1,52401	0,0091	0,0622298	no
XLOC_011570	g5294	222,947	235,644	0,0799071	0,136123	0,81045	0,912686	no
XLOC_011571	g5296	22,537	46,1267	1,03331	1,67145	0,00425	0,0364498	yes
XLOC_011572	g5298	284,756	735,995	1,36997	1,97074	0,0007	0,00954722	yes
XLOC_011573	g5299	69,0245	56,3548	-0,29257	-0,508136	0,3888	0,643822	no
XLOC_011574	g5292	39,2803	61,5577	0,648133	1,11865	0,0514	0,200059	no
XLOC_011575	g5295	50,5066	56,7725	0,168719	0,284143	0,6177	0,808291	no
XLOC_011576	g5297	42,4126	38,866	-0,125984	-0,207265	0,7277	0,870385	no
XLOC_011577	g5300	5,15118	10,5882	1,03948	1,51744	0,00835	0,0587371	no
XLOC_011578	g5301	44,4278	37,1066	-0,259784	-0,444774	0,4297	0,678074	no
XLOC_011579	g5302	58,4893	57,868	-0,015407	-0,0257552	0,9644	0,984142	no
XLOC_011580	g5303	20,2223	15,2882	-0,403524	-0,653647	0,23965	0,497749	no
XLOC_011581	g5304	35,7295	36,2581	0,02119	0,0317811	0,95635	0,980718	no
XLOC_011582	g5305	96,7094	199,88	1,04741	1,49897	0,01365	0,0831532	no
XLOC_011583	g11372	0	1,28435	inf	0	1	1	no
XLOC_011584	g5307	9,04821	10,0085	0,145519	0,230941	0,689	0,850853	no
XLOC_011585	g5308	31,8756	37,0384	0,216566	0,355002	0,52565	0,747557	no
XLOC_011586	g5306	30,3183	39,2597	0,372858	0,625244	0,26955	0,530883	no
XLOC_011587	g5309	0	0,120928	inf	0	1	1	no
XLOC_011588	g5310	31,436	34,8147	0,14728	0,249427	0,66625	0,836968	no
XLOC_011589	g5311	11,7299	7,27408	-0,689359	-1,04533	0,0739	0,251193	no
XLOC_011590	g11373	1358,65	1004,09	-0,436289	-0,445885	0,4536	0,698284	no
XLOC_011591	g11374	1144,91	716,817	-0,675564	-0,988293	0,0814	0,268148	no

XLOC_011592	g11375	24,0907	18,7	-0,36544	-0,589135	0,3051	0,566474	no
XLOC_011593	g5312	8,29646	11,8497	0,514283	0,42705	0,44885	0,694314	no
XLOC_011594	g5313	0	0	0	0	1	1	no
XLOC_011595	g5314	0	0	0	0	1	1	no
XLOC_011596	g11376	0	0	0	0	1	1	no
XLOC_011597	g11377	24,3221	16,6746	-0,544614	-0,705879	0,21555	0,471321	no
XLOC_011598	g5316	9,91609	10,1979	0,0404296	0,0596963	0,91555	0,963585	no
XLOC_011599	g5315	30,8022	36,2845	0,236318	0,360539	0,53085	0,751362	no
XLOC_011600	g5317	74,9684	101,608	0,43866	0,728281	0,1919	0,442199	no
XLOC_011601	g5319	27,7514	45,3511	0,708576	1,19317	0,03975	0,169274	no
XLOC_011602	g5320	5,84525	3,09903	-0,915445	-0,815475	0,3542	0,615379	no
XLOC_011603	g5318	73,6524	257,977	1,80844	2,27928	0,0003	0,00496796	yes
XLOC_011604	g11378	0	0	0	0	1	1	no
XLOC_011605	g5321	0	0	0	0	1	1	no
XLOC_011606	g11379	64,5358	82,7707	0,359022	0,621712	0,2803	0,542577	no
XLOC_011607	g5322	0	0	0	0	1	1	no
XLOC_011608	g5323	12,2626	14,3945	0,231258	0,286727	0,6247	0,811943	no
XLOC_011609	g5324	0	0	0	0	1	1	no
XLOC_011610	g5325	75,1361	59,6375	-0,333285	-0,434407	0,4376	0,68432	no
XLOC_011611	g5326	5,20734	4,25747	-0,290548	-0,319618	0,58065	0,783024	no
XLOC_011612	g11380	17,7043	9,76448	-0,858487	-1,21721	0,0319	0,146794	no
XLOC_011613	g11381	564,145	645,464	0,194272	0,278869	0,6316	0,816117	no
XLOC_011614	g5327	13,7713	23,8928	0,794916	1,36244	0,0162	0,0931405	no
XLOC_011615	g11382	44,9827	54,528	0,277626	0,448553	0,41415	0,666219	no
XLOC_011616	g11383	0	0	0	0	1	1	no
XLOC_011617	g11384	0	0	0	0	1	1	no
XLOC_011618	g987	0	0	0	0	1	1	no
XLOC_011619	g5328	12,4323	22,0111	0,824139	0,837282	0,1652	0,407706	no
XLOC_011620	g11385	0	0	0	0	1	1	no
XLOC_011621	g5329	30,8401	44,8817	0,541319	0,924844	0,1021	0,307647	no
XLOC_011622	g5331	36,9408	40,9114	0,147287	0,245442	0,6677	0,83795	no
XLOC_011623	g5330	11,4343	12,2786	0,102789	0,144309	0,797	0,906374	no
XLOC_011624	g5332	12,2256	13,9237	0,187636	0,265093	0,62735	0,813277	no
XLOC_011625	g11387	1445,25	770,636	-0,907197	-0,949618	0,10705	0,318169	no
XLOC_011626	g11389	323,455	280,267	-0,206763	-0,338604	0,5395	0,758065	no
XLOC_011627	g11391	91,8713	97,6736	0,0883544	0,14839	0,7968	0,906252	no
XLOC_011628	g11392	15,5425	14,6309	-0,0872057	-0,14058	0,809	0,912203	no
XLOC_011629	g11386	74,7247	51,6521	-0,532758	-0,856903	0,12295	0,346447	no
XLOC_011630	g11388	132,05	152,055	0,203507	0,343005	0,55	0,765215	no
XLOC_011631	g11390	50,2765	58,0685	0,207873	0,358316	0,5252	0,747134	no
XLOC_011632	g11393	41,0815	37,204	-0,14303	-0,244555	0,6635	0,835468	no
XLOC_011633	g11394	34,3253	19,5976	-0,808599	-1,062	0,08505	0,275368	no
XLOC_011634	g5333	0	0	0	0	1	1	no
XLOC_011635	g11395	161,905	136,469	-0,246577	-0,410713	0,46835	0,707556	no
XLOC_011636	g11396	137,816	127,999	-0,106611	-0,178705	0,74915	0,881846	no
XLOC_011637	g11397	6,50505	6,77282	0,0581965	0,0729794	0,89815	0,954734	no
XLOC_011638	g11398	56,8621	42,378	-0,42415	-0,697744	0,22075	0,477586	no
XLOC_011639	g11399	137,904	50,0469	-1,46232	-2,20531	0,0001	0,00209829	yes
XLOC_011640	g11400	5,06094	5,89204	0,219363	0,309757	0,5834	0,784861	no
XLOC_011641	g11401	30,2633	29,0414	-0,0594547	-0,0944516	0,86755	0,939446	no
XLOC_011642	g5334	351,903	611,66	0,797553	1,23531	0,03575	0,158066	no
XLOC_011643	g5335	23,0321	17,4599	-0,399599	-0,654919	0,24925	0,508092	no
XLOC_011644	g5336	1,39168	0,567766	-1,29346	-1,55099	0,0123	0,0774269	no
XLOC_011645	g5337	0	0	0	0	1	1	no
XLOC_011646	g5338	2,19649	2,54347	0,211596	0,173759	0,74975	0,881953	no
XLOC_011647	g5339	29,73	56,24	0,919675	1,52438	0,0056	0,0440994	yes
XLOC_011648	g11402	32,7288	30,8971	-0,0830913	-0,109867	0,8484	0,931286	no
XLOC_011649	g5340	0	0	0	0	1	1	no
XLOC_011650	g5342	47,6021	125,034	1,39322	2,26733	0,0002	0,00367103	yes
XLOC_011651	g5341	32,5108	44,1776	0,442396	0,739038	0,20035	0,45251	no
XLOC_011652	g11403	201,587	623,675	1,62939	2,42142	5,00E-05	0,00120049	yes
XLOC_011653	g11404	12,8653	9,085	-0,501924	-0,693058	0,21765	0,474223	no
XLOC_011654	g11405	6,97019	7,42326	0,0908551	0,104255	0,85555	0,934827	no
XLOC_011655	g11406	7867,03	295,847	-4,73289	-6,06196	5,00E-05	0,00120049	yes
XLOC_011656	g11407	24,312	14,392	-0,756406	-1,26696	0,027	0,133036	no
XLOC_011657	g989	43,8597	13,9979	-1,64769	-1,80822	0,00405	0,0351645	yes
XLOC_011658	g988	202,55	220,98	0,125638	0,202448	0,7274	0,870385	no
XLOC_011659	g990	20,412	124,04	2,60331	3,99027	5,00E-05	0,00120049	yes
XLOC_011660	g11408	13,7614	14,6548	0,090744	0,12423	0,82355	0,919425	no
XLOC_011661	g11409	161,927	225,036	0,474811	0,818017	0,15475	0,392851	no
XLOC_011662	g5343	0	0	0	0	1	1	no
XLOC_011663	g5344	24,5451	34,8113	0,504124	0,756123	0,1742	0,420013	no
XLOC_011664	g5345	3,24805	5,42733	0,740671	0,798067	0,17995	0,426853	no
XLOC_011665	g11410	63,6862	54,2858	-0,230406	-0,351863	0,52155	0,74518	no
XLOC_011666	g991	3,96501	4,53866	0,194942	0,194369	0,71925	0,867399	no
XLOC_011667	g992	2,9262	3,08989	0,0785273	0,0986651	0,8629	0,936801	no
XLOC_011668	g994	38,8858	111,198	1,51582	1,9195	0,00185	0,01964	yes
XLOC_011669	g995	46,4773	125,861	1,43723	1,60002	0,01145	0,0731073	no
XLOC_011670	g996	26,8563	52,8641	0,97703	1,16183	0,0476	0,189628	no
XLOC_011671	g997	5,12005	4,37726	-0,226131	-0,296147	0,57315	0,779376	no
XLOC_011672	g998	219,092	166,602	-0,395134	-0,58762	0,2949	0,556907	no
XLOC_011673	g993	15,569	22,7847	0,549394	0,772676	0,1861	0,434086	no
XLOC_011674	g999	23,9698	33,2301	0,471269	0,783146	0,164	0,406174	no
XLOC_011675	g11411	66,6998	85,5472	0,359037	0,612357	0,27455	0,536213	no
XLOC_011676	g5347	136,981	220,6	0,68745	0,993076	0,0707	0,244463	no
XLOC_011677	g5346	74,0565	134,399	0,859826	1,38973	0,01545	0,0903627	no
XLOC_011678	g5348	61,1462	70,4028	0,20337	0,330709	0,558	0,77025	no
XLOC_011679	g5349	35,6886	21,6249	-0,722767	-1,21479	0,0362	0,159196	no
XLOC_011680	g5351	8,4349	2,91633	-1,53222	-1,93688	0,00105	0,0130684	yes
XLOC_011681	g5350	26,2918	27,6141	0,0707908	0,102879	0,85835	0,935373	no
XLOC_011682	g5352	1344,84	3196,74	1,24917	1,49061	0,00575	0,045172	yes
XLOC_011683	g5353	842,424	544,404	-0,629869	-0,970233	0,0847	0,274597	no

XLOC_011684	g5355	7,20511	5,60804	-0,361525	-0,498165	0,3802	0,637127	no
XLOC_011685	g5354	8,93899	9,42955	0,0770783	0,0826644	0,88455	0,948387	no
XLOC_011686	g5356	49,1699	80,7309	0,715347	1,10204	0,05195	0,201481	no
XLOC_011687	g5358	132,395	64,2933	-1,04211	-1,76463	0,0025	0,0247231	yes
XLOC_011688	g5357	33,413	47,5686	0,5096	0,822957	0,15405	0,39191	no
XLOC_011689	g5359	386,081	512,893	0,409752	0,677498	0,2278	0,485987	no
XLOC_011690	g1002	24,191	41,3982	0,775096	1,29285	0,02255	0,118481	no
XLOC_011691	g1000	5,61728	21,3582	1,92684	2,77179	5,00E-05	0,00120049	yes
XLOC_011692	g1001	13,7459	38,3219	1,47917	1,54281	0,0117	0,0743169	no
XLOC_011693	g11412	0	0	0	0	1	1	no
XLOC_011694	g11413	0	0	0	0	1	1	no
XLOC_011695	g11414	0	0	0	0	1	1	no
XLOC_011696	g11415	0	0	0	0	1	1	no
XLOC_011697	g11416	49,1698	47,6891	-0,044113	-0,0494939	0,9245	0,966785	no
XLOC_011698	g5360	135,851	136,502	0,00689706	0,0106354	0,98575	0,992622	no
XLOC_011699	g1006	21,3849	24,0609	0,170103	0,217201	0,70945	0,862121	no
XLOC_011700	g1008	34,0724	40,7464	0,258069	0,436002	0,45345	0,698162	no
XLOC_011701	g1011	79,3705	60,0541	-0,402341	-0,674505	0,23185	0,489627	no
XLOC_011702	g1013	33,7737	66,4158	0,975624	1,59169	0,0063	0,0482574	yes
XLOC_011703	g1016	12,2597	15,1357	0,304029	0,383589	0,4876	0,720794	no
XLOC_011704	g1017	1850,72	2190,83	0,243391	0,348801	0,5396	0,758065	no
XLOC_011705	g1021	1254,5	1339,41	0,0944898	0,117203	0,83145	0,922581	no
XLOC_011706	g1023	25,8459	32,0075	0,30847	0,507463	0,3814	0,637999	no
XLOC_011707	g1026	47,3612	51,5044	0,12099	0,206558	0,71695	0,865633	no
XLOC_011708	g1027	19,5173	23,1158	0,24413	0,265631	0,63215	0,816117	no
XLOC_011709	g1029	40,1249	51,1964	0,351547	0,596687	0,3	0,562214	no
XLOC_011710	g1030	9,89186	9,47406	-0,0622577	-0,0901175	0,87	0,941318	no
XLOC_011711	g1031	13,9287	6,56794	-1,08455	-1,512	0,01065	0,0695366	no
XLOC_011712	g1032	17,2964	10,2081	-0,760761	-1,22816	0,0325	0,148719	no
XLOC_011713	g1003	8,79614	4,30489	-1,03089	-1,27257	0,0296	0,140761	no
XLOC_011714	g1004	54,4316	110,955	1,02745	1,67316	0,0039	0,0342252	yes
XLOC_011715	g1005	36,1259	63,7122	0,818536	1,27718	0,0238	0,122944	no
XLOC_011716	g1007	24,8841	37,4726	0,590611	0,999053	0,0825	0,270501	no
XLOC_011717	g1009	21,2737	33,7783	0,667026	1,02808	0,0683	0,238941	no
XLOC_011718	g1010	22,1565	26,5172	0,259201	0,433767	0,4395	0,685606	no
XLOC_011719	g1012	75,7645	101,918	0,427813	0,709058	0,2116	0,467156	no
XLOC_011720	g1014	8,85116	9,29417	0,0704601	0,0784841	0,8961	0,95369	no
XLOC_011721	g1015	140,557	95,9107	-0,551397	-0,832692	0,1505	0,387828	no
XLOC_011722	g1018	49,6748	39,8889	-0,316524	-0,536424	0,3516	0,612183	no
XLOC_011723	g1019	314,012	343,329	0,128775	0,200599	0,7154	0,865069	no
XLOC_011724	g1020	56,529	57,2294	0,0177653	0,0298114	0,9589	0,981744	no
XLOC_011725	g1022	74,5528	62,1274	-0,263033	-0,424682	0,44885	0,694314	no
XLOC_011726	g1024	310,898	325,753	0,0673351	0,11441	0,8346	0,924405	no
XLOC_011727	g1025	29,6434	45,2282	0,609513	0,851591	0,1164	0,334616	no
XLOC_011728	g1028	51,6837	46,5016	-0,152427	-0,263515	0,64525	0,82511	no
XLOC_011729	g1033	17,4543	22,9208	0,393072	0,656202	0,24685	0,505646	no
XLOC_011730	g5361	12,1293	19,4447	0,680879	0,957447	0,1019	0,307384	no
XLOC_011731	g5362	1,83472	23,05	3,65113	2,42365	0,00535	0,0426783	yes
XLOC_011732	g5363	18,5827	24,4598	0,396454	0,503821	0,37715	0,635486	no
XLOC_011733	g11417	50,8972	55,4598	0,123857	0,191384	0,7437	0,879048	no
XLOC_011734	g5364	2,56229	3,37839	0,398901	0,477229	0,40615	0,659809	no
XLOC_011735	g1034	2544,17	11905,7	2,22639	2,36685	0,0003	0,00496796	yes
XLOC_011736	g5365	83,4238	31,2135	-1,41829	-2,26281	0,0001	0,00209829	yes
XLOC_011737	g5366	4,80328	5,57944	0,2161	0,201226	0,7148	0,865069	no
XLOC_011738	g5367	110,777	196,621	0,827758	1,11228	0,0468	0,187812	no
XLOC_011739	g5368	27,8002	31,2756	0,169939	0,22142	0,69475	0,854729	no
XLOC_011740	g11418	0	1,02782	inf	0	1	1	no
XLOC_011741	g11419	66,2473	117,47	0,826358	1,26948	0,03165	0,146329	no
XLOC_011742	g11420	0	0	0	0	1	1	no
XLOC_011743	g1035	246,096	40,8389	-2,59121	-3,18294	5,00E-05	0,00120049	yes
XLOC_011744	g5370	68,7819	86,9978	0,33895	0,497299	0,3953	0,649464	no
XLOC_011745	g5371	5,94107	7,79983	0,392719	0,378689	0,50265	0,730938	no
XLOC_011746	g5372	0	0	0	0	1	1	no
XLOC_011747	g5369	20,8155	18,6731	-0,156699	-0,202994	0,72985	0,871761	no
XLOC_011748	g5373	40,9547	56,6763	0,468717	0,418085	0,4857	0,719067	no
XLOC_011749	g5374	84,0805	52,8261	-0,670521	-1,11508	0,05375	0,206101	no
XLOC_011750	g5375	2118,96	823,681	-1,3632	-1,3057	0,04185	0,175029	no
XLOC_011751	g5376	418,006	334,462	-0,321679	-0,514533	0,3652	0,624894	no
XLOC_011752	g5377	81,2852	37,0462	-1,13367	-1,55187	0,0078	0,0560322	no
XLOC_011753	g5378	27,5488	11,611	-1,2465	-1,21414	0,0434	0,179071	no
XLOC_011754	g11421	9,05595	16,2877	0,846847	0,961187	0,08575	0,276611	no
XLOC_011755	g5379	77,1304	84,6128	0,133578	0,216765	0,69895	0,85689	no
XLOC_011756	g5380	3,9859	2,97814	-0,420491	-0,455549	0,4375	0,68432	no
XLOC_011757	g5381	29,3708	17,6117	-0,737851	-0,774318	0,19835	0,451297	no
XLOC_011758	g5382	2,7606	10,2155	1,8877	1,52689	0,0147	0,0874343	no
XLOC_011759	g11422	0	0	0	0	1	1	no
XLOC_011760	g11423	0	0	0	0	1	1	no
XLOC_011761	g11424	0,00848888	0,00722666	-0,232244	0	1	1	no
XLOC_011762	g11425	118,079	98,846	-0,2565	-0,42856	0,46045	0,701864	no
XLOC_011763	g5384	43,6966	58,6358	0,424261	0,630868	0,27485	0,53647	no
XLOC_011764	g5383	429,964	320,455	-0,424091	-0,639269	0,25375	0,513038	no
XLOC_011765	g11426	0	0	0	0	1	1	no
XLOC_011766	g11427	88,8297	73,1188	-0,280799	-0,428751	0,44735	0,693445	no
XLOC_011767	g5385	12,6318	7,10181	-0,830804	-1,07455	0,06635	0,234381	no
XLOC_011768	g5386	12,2645	7,42904	-0,723243	-1,00967	0,0836	0,27247	no
XLOC_011769	g11428	0	0	0	0	1	1	no
XLOC_011770	g5387	176,034	284,685	0,693511	1,02116	0,0727	0,249098	no
XLOC_011771	g11429	29,2581	39,8763	0,446698	0,580716	0,3082	0,569216	no
XLOC_011772	g11431	0	0,0889086	inf	0	1	1	no
XLOC_011773	g11430	0	0	0	0	1	1	no
XLOC_011774	g11432	105,074	95,1415	-0,143256	-0,179701	0,75735	0,8854	no
XLOC_011775	g5388	0	0	0	0	1	1	no

XLOC_011776	g1038	33,1118	44,1823	0,416124	0,671552	0,2255	0,483496	no
XLOC_011777	g1036	20,8499	8,69488	-1,2618	-1,60455	0,00745	0,0543933	no
XLOC_011778	g1037	112,651	137,345	0,285946	0,448759	0,41575	0,667866	no
XLOC_011779	g1039	3,64951	13,1661	1,85105	2,76521	5,00E-05	0,00120049	yes
XLOC_011780	g11434	188,525	26,4421	-2,83385	-4,29357	5,00E-05	0,00120049	yes
XLOC_011781	g11433	22,3818	8,87209	-1,33498	-1,41596	0,0209	0,112459	no
XLOC_011782	g5389	4,82936	9,26124	0,939373	1,27967	0,0245	0,124723	no
XLOC_011783	g5390	33,7633	34,7009	0,0395157	0,0610133	0,91265	0,962645	no
XLOC_011784	g5391	0	0	0	0	1	1	no
XLOC_011785	g5392	0	0	0	0	1	1	no
XLOC_011786	g11435	26,3922	22,5264	-0,228495	-0,318983	0,56815	0,776676	no
XLOC_011787	g5393	0	0	0	0	1	1	no
XLOC_011788	g5394	0	0	0	0	1	1	no
XLOC_011789	g11436	66,3251	88,9086	0,422768	0,537858	0,3475	0,607846	no
XLOC_011790	g11437	0	0	0	0	1	1	no
XLOC_011791	g5395	0	0	0	0	1	1	no
XLOC_011792	g1040	235,232	740,898	1,65519	2,60121	5,00E-05	0,00120049	yes
XLOC_011793	g1042	4,00787	12,9107	1,68766	2,21056	0,00025	0,00430702	yes
XLOC_011794	g1044	29,5969	33,7158	0,18798	0,32295	0,57155	0,77837	no
XLOC_011795	g1046	112,213	170,367	0,602412	1,05378	0,0726	0,248842	no
XLOC_011796	g1041	45,6662	103,221	1,17654	1,89766	0,00125	0,0146655	yes
XLOC_011797	g1043	123,924	211,492	0,771145	1,27598	0,028	0,135891	no
XLOC_011798	g1045	142,402	183,978	0,36956	0,611654	0,2898	0,552768	no
XLOC_011799	g5396	73,1153	96,764	0,404296	0,692879	0,22625	0,484363	no
XLOC_011800	g5398	502,22	648,229	0,368183	0,511245	0,36395	0,623733	no
XLOC_011801	g5399	12,362	20,8306	0,752797	0,643339	0,4604	0,701864	no
XLOC_011802	g5400	71,4241	97,5283	0,449409	0,740379	0,1858	0,433695	no
XLOC_011803	g5402	464,758	671,569	0,531058	0,699962	0,22775	0,485987	no
XLOC_011804	g5397	140,283	135,928	-0,0455042	-0,0697058	0,90485	0,958435	no
XLOC_011805	g5401	132,924	142,754	0,102933	0,169795	0,7667	0,890794	no
XLOC_011806	g11438	10,2382	6,74478	-0,602115	-0,906814	0,1108	0,324793	no
XLOC_011807	g5403	0	0	0	0	1	1	no
XLOC_011808	g11439	514,435	2305,32	2,1639	3,01228	5,00E-05	0,00120049	yes
XLOC_011809	g1047	2,70798	2,65462	-0,0287144	-0,0317563	0,9516	0,978709	no
XLOC_011810	g1048	43,2378	30,9001	-0,484679	-0,809648	0,1496	0,387107	no
XLOC_011811	g5404	0,716607	0,263848	-1,44148	0	1	1	no
XLOC_011812	g5405	26,2644	38,0343	0,534191	0,855418	0,13615	0,367609	no
XLOC_011813	g5406	0	0,31344	inf	0	1	1	no
XLOC_011814	g5408	88,0255	77,498	-0,183763	-0,301928	0,5835	0,784861	no
XLOC_011815	g5410	102,358	128,368	0,326671	0,522293	0,3542	0,615379	no
XLOC_011816	g5407	59,8863	93,703	0,64587	1,08877	0,0606	0,221553	no
XLOC_011817	g5409	90,7392	52,8097	-0,780922	-1,24679	0,0299	0,141434	no
XLOC_011818	g5411	39,2111	19,7552	-0,989033	-1,61492	0,0041	0,0354419	yes
XLOC_011819	g5412	0	0	0	0	1	1	no
XLOC_011820	g11440	0	0	0	0	1	1	no
XLOC_011821	g5413	79,8203	87,1596	0,126904	0,208345	0,7124	0,863888	no
XLOC_011822	g1051	39,0001	31,0704	-0,327938	-0,561078	0,3205	0,580544	no
XLOC_011823	g1054	191,866	596,982	1,63759	2,40307	5,00E-05	0,00120049	yes
XLOC_011824	g1055	55,6235	41,0918	-0,436843	-0,751364	0,19055	0,440813	no
XLOC_011825	g1057	198,48	221,975	0,161402	0,263172	0,64825	0,826837	no
XLOC_011826	g1059	41,0598	47,6109	0,213565	0,331883	0,5583	0,7703	no
XLOC_011827	g1060	60,3492	61,9183	0,0370313	0,05956	0,91385	0,963393	no
XLOC_011828	g1049	68,0978	58,1384	-0,228118	-0,391925	0,4892	0,721963	no
XLOC_011829	g1050	57,4933	59,6059	0,0520599	0,088512	0,87165	0,942376	no
XLOC_011830	g1052	372,782	210,337	-0,825634	-1,12941	0,04605	0,185789	no
XLOC_011831	g1053	126,563	86,901	-0,542412	-0,9328	0,0991	0,301943	no
XLOC_011832	g1056	15,5132	160,928	3,37484	4,79651	5,00E-05	0,00120049	yes
XLOC_011833	g1058	42,8846	41,2468	-0,0561747	-0,0954779	0,86255	0,936559	no
XLOC_011834	g1061	12,236	20,4226	0,73903	1,1344	0,0504	0,197813	no
XLOC_011835	g1062	2,39838	1,85948	-0,367159	-0,314174	0,6191	0,808526	no
XLOC_011836	g1063	33,9998	52,7903	0,634747	0,98519	0,08355	0,272397	no
XLOC_011837	g11441	12,7475	7,74091	-0,71964	-0,919202	0,1118	0,3267	no
XLOC_011838	g5415	14,272	5,71771	-1,31968	-1,86359	0,0034	0,0310297	yes
XLOC_011839	g5416	32,4778	32,4491	-0,00127309	-0,00217111	0,99655	0,997966	no
XLOC_011840	g5414	55,0087	60,6668	0,141247	0,243251	0,6652	0,836076	no
XLOC_011841	g5417	122,093	215,382	0,818916	1,20446	0,0481	0,191154	no
XLOC_011842	g5419	13,4187	20,1098	0,583657	0,853776	0,13735	0,369528	no
XLOC_011843	g5420	17,5234	17,5619	0,00316622	0,00414417	0,99175	0,995293	no
XLOC_011844	g5422	68,3989	22,7054	-1,59093	-2,4623	5,00E-05	0,00120049	yes
XLOC_011845	g5418	86,8374	148,219	0,771338	0,969105	0,08335	0,271836	no
XLOC_011846	g5421	25,6176	20,0111	-0,356334	-0,567809	0,31405	0,574832	no
XLOC_011847	g5423	3,47329	5,25452	0,597255	0,472576	0,3833	0,639811	no
XLOC_011848	g5424	199,503	257,086	0,365842	0,586322	0,29585	0,557736	no
XLOC_011849	g5425	469,272	627,729	0,419716	0,6124	0,28415	0,546579	no
XLOC_011850	g5426	25,7411	27,568	0,0989203	0,164669	0,76905	0,89268	no
XLOC_011851	g5427	15,6573	11,6586	-0,425433	-0,644211	0,2686	0,529649	no
XLOC_011852	g5429	1,62724	11,8405	2,86323	2,52793	0,04175	0,174759	no
XLOC_011853	g5428	1439,35	3560,75	1,30677	1,065	0,17755	0,424426	no
XLOC_011854	g5430	11,194	4,56191	-1,29501	-1,59724	0,0096	0,0644815	no
XLOC_011855	g5431	0	0	0	0	1	1	no
XLOC_011856	g5432	6,57864	22,5634	1,77812	2,33241	5,00E-05	0,00120049	yes
XLOC_011857	g11442	1818,7	118,189	-3,94374	-4,35175	5,00E-05	0,00120049	yes
XLOC_011858	g11443	174,576	158,039	-0,14357	-0,244256	0,67475	0,842151	no
XLOC_011859	g11445	173,351	449,722	1,37533	2,11414	0,00045	0,00669545	yes
XLOC_011860	g11444	27,815	26,8729	-0,0497116	-0,08571	0,88515	0,94862	no
XLOC_011861	g11446	1252,87	5675,65	2,17955	3,32545	5,00E-05	0,00120049	yes
XLOC_011862	g11447	0,62654	0	#NAME?	0	1	1	no
XLOC_011863	g11448	6,73297	5,27952	-0,350836	-0,394477	0,48265	0,716713	no
XLOC_011864	g5433	39,5965	48,0684	0,279715	0,332354	0,5667	0,775285	no
XLOC_011865	g11450	62,6423	159,579	1,34906	2,25294	0,0001	0,00209829	yes
XLOC_011866	g11454	60,7237	59,3723	-0,0324713	-0,0551971	0,92495	0,966895	no
XLOC_011867	g11456	131,615	387,729	1,55872	2,38706	5,00E-05	0,00120049	yes

XLOC_011868	g11459	57,5935	51,2555	-0,168199	-0,266672	0,63295	0,816463	no
XLOC_011869	g11461	2058,25	3073,58	0,578502	0,87917	0,13365	0,363462	no
XLOC_011870	g11462	207,53	77,7569	-1,41628	-2,40856	5,00E-05	0,00120049	yes
XLOC_011871	g11464	83,53	73,4414	-0,185701	-0,288022	0,61965	0,808526	no
XLOC_011872	g11466	404,897	677,213	0,742056	0,990016	0,0805	0,265972	no
XLOC_011873	g11468	47,5612	68,2821	0,521721	0,754378	0,1793	0,426373	no
XLOC_011874	g11449	225,542	223,67	-0,0120231	-0,0188292	0,97295	0,987532	no
XLOC_011875	g11451	66,9429	54,0382	-0,308954	-0,516932	0,36075	0,621176	no
XLOC_011876	g11452	55,6507	61,6372	0,147402	0,235197	0,6824	0,847104	no
XLOC_011877	g11453	215,489	195,028	-0,143932	-0,237243	0,67785	0,844303	no
XLOC_011878	g11455	237,511	172,387	-0,462337	-0,745922	0,18535	0,433572	no
XLOC_011879	g11457	38,6473	25,8447	-0,5805	-0,917498	0,10035	0,303772	no
XLOC_011880	g11458	31,0196	27,9095	-0,152424	-0,224787	0,70015	0,85752	no
XLOC_011881	g11460	2939,31	2113,04	-0,476158	-0,45191	0,41885	0,670105	no
XLOC_011882	g11463	296,792	450,201	0,601119	0,852909	0,1247	0,348082	no
XLOC_011883	g11465	146,723	167,973	0,195134	0,329253	0,5497	0,765215	no
XLOC_011884	g11467	669,883	1104,82	0,721827	1,05395	0,06475	0,230629	no
XLOC_011885	g11469	115,636	105,347	-0,134441	-0,178797	0,76205	0,887899	no
XLOC_011886	g5434	61,2607	64,3799	0,0716472	0,114347	0,8407	0,926771	no
XLOC_011887	g11470	12,007	6,13081	-0,969725	-1,12854	0,0529	0,204278	no
XLOC_011888	g5435	831,153	813,287	-0,0313487	-0,0419154	0,94475	0,975496	no
XLOC_011889	g5436	0	0	0	0	1	1	no
XLOC_011890	g11471	330,579	125,573	-1,39647	-1,52721	0,0166	0,09494	no
XLOC_011891	g11474	22,1036	9,0231	-1,29258	-1,6481	0,0064	0,048795	yes
XLOC_011892	g11472	4,28467	5,88062	0,456784	0,626849	0,26845	0,52946	no
XLOC_011893	g11473	19,9544	20,927	0,0686588	0,107154	0,8538	0,934498	no
XLOC_011894	g5437	13,5865	3,43989	-1,98175	-2,47576	0,00025	0,00430702	yes
XLOC_011895	g5438	6,10525	3,19447	-0,934475	-1,14524	0,05715	0,214203	no
XLOC_011896	g11475	59,2825	62,4932	0,0760947	0,115938	0,84455	0,92945	no
XLOC_011897	g5439	58,7251	60,2791	0,0376816	0,0629024	0,9116	0,962529	no
XLOC_011898	g5441	12,0853	5,18275	-1,22146	-1,5127	0,01205	0,0761951	no
XLOC_011899	g5440	63,7321	78,6411	0,303262	0,485193	0,37915	0,636522	no
XLOC_011900	g11476	20,7875	26,5814	0,3547	0,475819	0,4231	0,672849	no
XLOC_011901	g5442	14,7488	14,9955	0,0239353	0,0375152	0,9481	0,976439	no
XLOC_011902	g11477	0	0	0	0	1	1	no
XLOC_011903	g5443	8,00328	14,3646	0,843854	1,18763	0,0423	0,17661	no
XLOC_011904	g5444	140,426	14,1338	-3,31259	-3,52284	5,00E-05	0,00120049	yes
XLOC_011905	g5446	40,7985	47,6482	0,223905	0,350282	0,52835	0,749406	no
XLOC_011906	g5445	9,49645	3,71215	-1,35513	-1,87527	0,00175	0,0188639	yes
XLOC_011907	g5447	12,8582	10,0546	-0,35484	-0,519102	0,37075	0,629539	no
XLOC_011908	g11479	2640,78	1937,38	-0,446861	-0,518653	0,35175	0,612336	no
XLOC_011909	g11478	40,4961	22,8539	-0,82534	-1,32888	0,0168	0,0958605	no
XLOC_011910	g1064	0	0	0	0	1	1	no
XLOC_011911	g1065	0	0	0	0	1	1	no
XLOC_011912	g1066	0	0	0	0	1	1	no
XLOC_011913	g5448	108,529	46,2756	-1,22975	-1,19635	0,0573	0,214357	no
XLOC_011914	g5449	36,7315	27,6584	-0,4093	-0,485853	0,4081	0,660548	no
XLOC_011915	g5450	112,528	74,0248	-0,604199	-0,749446	0,19995	0,452439	no
XLOC_011916	g11480	655,534	1250,98	0,932318	1,51146	0,0069	0,051293	no
XLOC_011917	g5451	180,864	93,0351	-0,95906	-1,45814	0,00925	0,0629487	no
XLOC_011918	g11482	50,091	30,2667	-0,726818	-1,10265	0,05335	0,205128	no
XLOC_011919	g11481	60,5515	22,2125	-1,44679	-1,9886	0,00135	0,0156148	yes
XLOC_011920	g1067	0	0	0	0	1	1	no
XLOC_011921	g11483	91,9257	127,93	0,47681	0,715867	0,20585	0,460047	no
XLOC_011922	g5452	281,987	227,103	-0,312283	-0,504763	0,37045	0,629539	no
XLOC_011923	g5453	245,452	238,028	-0,0443066	-0,0719296	0,8987	0,955112	no
XLOC_011924	g5454	9,85801	16,3094	0,726334	0,991052	0,084	0,273411	no
XLOC_011925	g1069	24,6219	26,4926	0,105645	0,184674	0,7501	0,882048	no
XLOC_011926	g1071	16,3694	17,742	0,116166	0,194084	0,73335	0,874074	no
XLOC_011927	g1072	19,4273	29,1269	0,584271	0,858542	0,1395	0,372621	no
XLOC_011928	g1074	55,1694	79,6846	0,530432	0,903212	0,10835	0,319999	no
XLOC_011929	g1076	19,7982	24,5708	0,311574	0,488858	0,4008	0,654912	no
XLOC_011930	g1077	32,658	59,1049	0,855841	1,27591	0,0271	0,133261	no
XLOC_011931	g1079	17,59	11,9959	-0,552213	-0,780477	0,1646	0,406737	no
XLOC_011932	g1068	55,2301	51,8617	-0,0907855	-0,155831	0,7884	0,902341	no
XLOC_011933	g1070	51,9496	35,2551	-0,559282	-0,904556	0,12255	0,345717	no
XLOC_011934	g1073	6,29672	4,01355	-0,649721	-0,93182	0,10415	0,3121	no
XLOC_011935	g1075	8,62565	11,2086	0,377904	0,553114	0,33745	0,597377	no
XLOC_011936	g1078	0,456768	13,9811	4,93587	4,65083	5,00E-05	0,00120049	yes
XLOC_011937	g5455	6,75423	13,241	0,971154	1,35678	0,01775	0,0996029	no
XLOC_011938	g11484	0	0	0	0	1	1	no
XLOC_011939	g11485	0	0,0727859	inf	0	1	1	no
XLOC_011940	g11486	0	0	0	0	1	1	no
XLOC_011941	g11488	121,196	147,926	0,287533	0,429144	0,44245	0,688832	no
XLOC_011942	g11487	47,8662	51,9624	0,118461	0,201522	0,7194	0,867399	no
XLOC_011943	g5456	334,071	130,716	-1,35372	-1,92018	0,0006	0,00845337	yes
XLOC_011944	g1081	47,0577	54,0244	0,19918	0,321886	0,56225	0,772769	no
XLOC_011945	g1080	29,168	26,1458	-0,157809	-0,186679	0,7472	0,881064	no
XLOC_011946	g1082	76,4722	176,201	1,20422	1,60308	0,00585	0,0457017	yes
XLOC_011947	g1083	53,5631	24,7586	-1,11331	-1,78179	0,00155	0,0171215	yes
XLOC_011948	g5457	33,9928	28,2308	-0,26796	-0,418272	0,4721	0,709631	no
XLOC_011949	g5459	34,368	34,9659	0,0248795	0,0416431	0,94045	0,973666	no
XLOC_011950	g5458	35,5945	31,9029	-0,157969	-0,219683	0,6973	0,856211	no
XLOC_011951	g11489	84,0185	50,8545	-0,72433	-1,21419	0,03555	0,15775	no
XLOC_011952	g11491	29,0189	21,7323	-0,417154	-0,672902	0,24235	0,500816	no
XLOC_011953	g11490	36,2371	15,7438	-1,20269	-1,7876	0,00165	0,0180837	yes
XLOC_011954	g11492	20,1731	35,2835	0,806561	1,2876	0,0238	0,122944	no
XLOC_011955	g1084	1,30259	1,47609	0,180397	0,181722	0,7552	0,884596	no
XLOC_011956	g5460	0	0	0	0	1	1	no
XLOC_011957	g5461	0	0	0	0	1	1	no
XLOC_011958	g11493	1770,38	178,361	-3,31119	-5,24636	0,0094	0,0637927	no
XLOC_011959	g5462	69,9554	59,0046	-0,245608	-0,426678	0,4588	0,701036	no

XLOC_011960	g5464	16,1482	8,93835	-0,853295	-1,07834	0,06945	0,241672	no
XLOC_011961	g5466	48,3116	299,065	2,63002	3,46991	5,00E-05	0,00120049	yes
XLOC_011962	g5463	36,7467	54,9486	0,580471	0,954105	0,0943	0,292953	no
XLOC_011963	g5465	17,4748	15,5395	-0,169343	-0,285731	0,6167	0,807789	no
XLOC_011964	g1085	53,1101	61,6142	0,214278	0,356683	0,5258	0,747625	no
XLOC_011965	g1086	79,613	96,17	0,272583	0,436275	0,43585	0,683059	no
XLOC_011966	g5468	18,1646	17,8355	-0,0263786	-0,043636	0,93765	0,972153	no
XLOC_011967	g5469	31,8789	35,4469	0,153057	0,254104	0,65245	0,8295	no
XLOC_011968	g5467	52,5811	69,677	0,406139	0,584001	0,30295	0,564534	no
XLOC_011969	g5470	14,9398	23,6512	0,662755	1,01701	0,07815	0,2605	no
XLOC_011970	g5471	828,964	1108,73	0,419523	0,65875	0,2381	0,496104	no
XLOC_011971	g5472	1784,06	426,802	-2,06353	-2,15641	0,0001	0,00209829	yes
XLOC_011972	g5473	0	0,0177876	inf	0	1	1	no
XLOC_011973	g5474	0	0	0	0	1	1	no
XLOC_011974	g11494	0	0	0	0	1	1	no
XLOC_011975	g5476	0	0	0	0	1	1	no
XLOC_011976	g5477	0	0	0	0	1	1	no
XLOC_011977	g5475	0	0	0	0	1	1	no
XLOC_011978	g11495	2389,56	12301,1	2,36397	3,94612	5,00E-05	0,00120049	yes
